# Supplementary material for: Synthesis of substituted pyridines with diverse functional groups via the remodeling of (Aza)indole/Benzofuran skeletons
Source: Commun Chem. 2023 Jun 7;6:112. doi: 10.1038/s42004-023-00914-5 (PMC10247795; doi:10.1038/s42004-023-00914-5)
Supplement: Supplementary file 2 — supplementary material [file 42004_2023_914_MOESM2_ESM.pdf]

< Supplementary Information >

Synthesis of Substituted Pyridines with Diverse Functional Groups *via* the Remodeling of the (Aza)indole/Benzofuran Skeleton

CRI Center for Chemical Proteomics, Department of Chemistry

Seoul National University, Seoul 08826 (Korea)

*Corresponding author. E-mail: sbpark@snu.ac.kr

Contents

I. Supplementary Figures and Tables 2

Supplementary Methods

II. General Information 13

III. General Synthetic Procedures and Characterization of All Starting Materials 14

IV. Experimental Synthetic Procedures and Characterization of the Final Products 43

V. Synthetic Application for the Synthesis of Privileged Pyridine Scaffold Containing Bio-relevant Molecules 84

VI. Supplementary References 88

I. Supplementary Figures and Tables

(1) Investigation of hypothesis and optimizing the reaction conditions

Initial test reactions were performed on the basis of the hypothesis for the synthesis of substituted pyridines from either (aza)indoles or benzofurans *via* the ring cleavage reaction. As shown in Supplementary Figure 1, the reaction of ammonium acetate with carbonyl compounds (**I**) containing an electron-withdrawing group at the α-position allows the *in situ* formation of enamine (**II**). The resulting enamine **II** undergoes an Aldol-type reaction with 3-formyl (aza)indoles/benzofurans (**III**) to furnish the intermediate **IV**. The dehydration of the intermediate **IV** would provide the amine intermediate **V**, which undergoes intramolecular cyclization followed by the subsequent re-aromatization *via* C–N bond cleavage. These transformations provided *meta*-aminoaryl/phenol-conjugated *ortho*-substituted nicotinates, 3-pyridyl sulfones, or 3-pyridyl phosphonates (**VII**).

Supplementary Figure 1. Working hypothesis and plausible mechanism for synthesizing substituted pyridines *via* the ring cleavage of (aza)indoles and benzofurans.

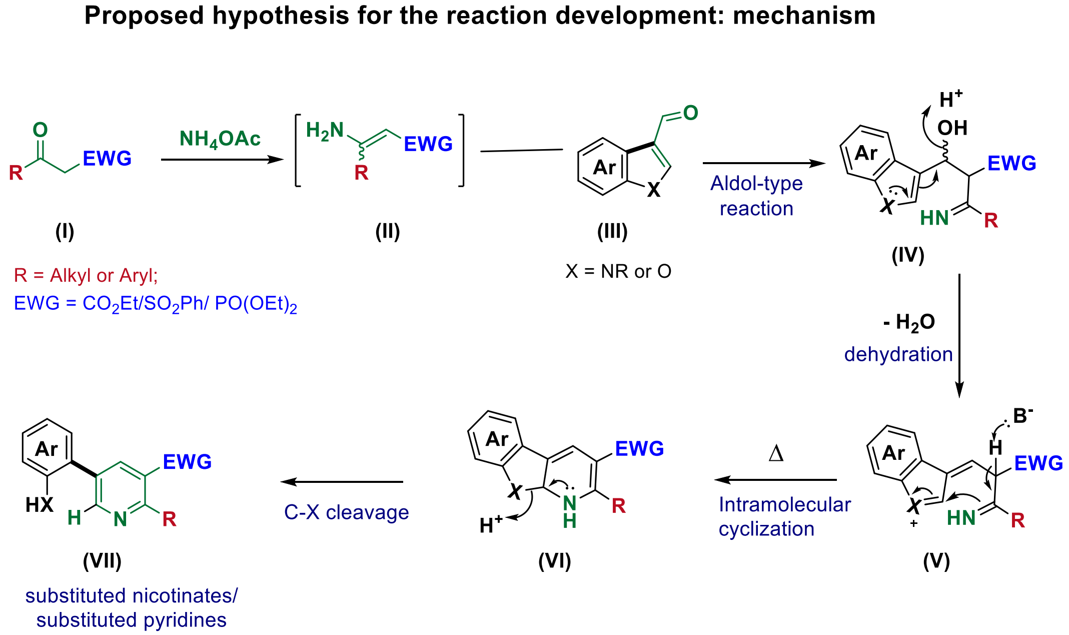

(2) Reaction optimization

All screening experiments for the reaction optimization were performed in a 4.0-mL sealed vial equipped with a magnetic bar and a Teflon-lined screwed cap. The yield of each reaction was confirmed by crude ^{1}H NMR analysis. All ^{1}H NMR yields were quantified using 1,3,5-trimethoxybenzene as an internal standard. Values given in the parenthesis are isolated yields

Supplementary Table 1. Reaction optimization by solvent for *N*-phenylsulfonyl 3-formyl-7-azaindole (**1b**)

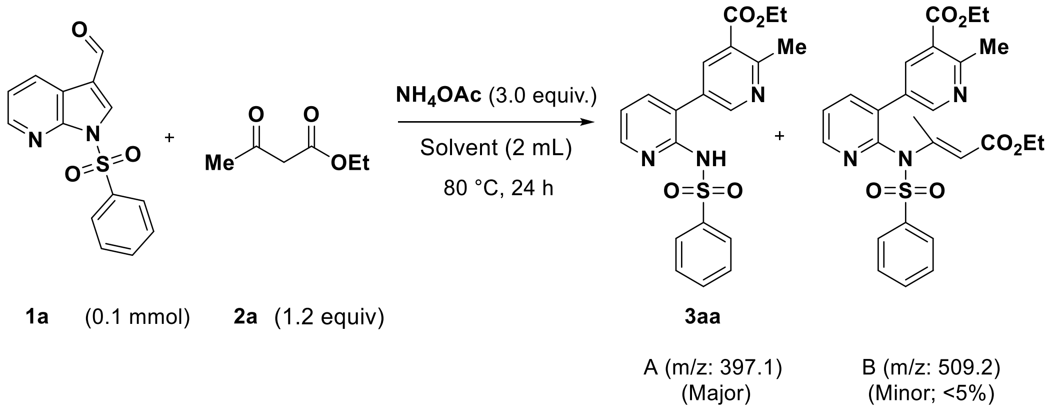

Entry	Solvent	LC/MS (5 h)			NMR (24 h)	
		result	A	B	% SM conversion	% Yield (A) [a]
1	EtOH	incomplete	major	major	100%	32%
2	CH_3CN	incomplete	trace	trace	100%	50%
3	DCE	incomplete	trace	major	97%	34%
4	dioxane	incomplete	minor	major	97%	14%
5	THF	incomplete	trace	major	16%	11%
6	DMF	incomplete	minor	major	97%	12%
7	DMSO	incomplete	minor	major	100%	6%
8	toluene	incomplete	minor	major	96%	20%
9	water	no reaction	-	-	-	-

Notes: [a]Crude NMR yields with respect to the methyl peak at δ ~2.81 ppm using 1,3,5-trimethoxy benzene as an internal reference.

Among various solvents, we found that ethanol (EtOH), acetonitrile (CH_3CN), and 1,2-dichloroethane (DCE) are suitable solvents for this methodology. We then optimized the reaction conditions by changing these solvents with various temperatures.

Supplementary Table 2. Reaction optimization by temperature and solvent for *N*-phenylsulfonyl 3-formyl 7-azaindoles.

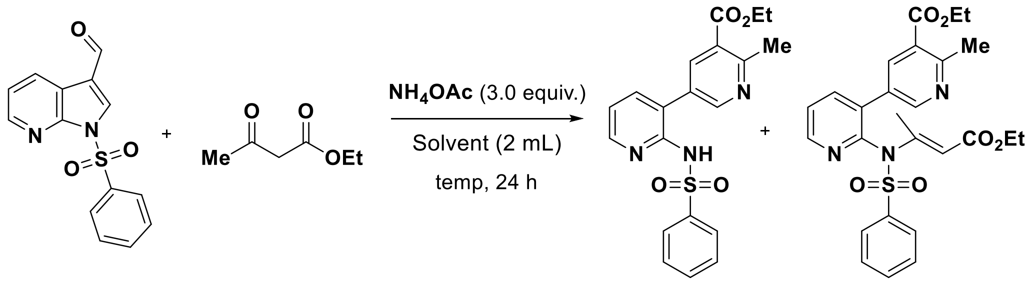

1a (0.1 mmol) **2a** (1.2 equiv)

3aa

A (m/z: 397.1) (Major) B (m/z: 509.2) (Minor; <5%)

Entry	Solvent	Temp (°C)	A (LC/MS)	B (LC/MS)	% Yield (A)[a]
1	EtOH	100	major	minor	49%
2	EtOH	120	major	minor	49%
3	CH_3CN	100	major	trace	53%
4	CH_3CN	120	major	trace	54%
5	DCE	100	major	trace	53%
6	**DCE**	**120**	**major**	**trace**	**69%**

Notes: [a]Crude NMR yields with respect to the methyl peak at ~δ 2.81 ppm using 1,3,5-trimethoxy benzene as an internal reference.

Based on the above screening results, we selected DCE as the best solvent for this transformation at 120 °C as the optimal temperature.

Supplementary Table 3. Reaction optimization concerning time and equivalence of ammonium acetate.

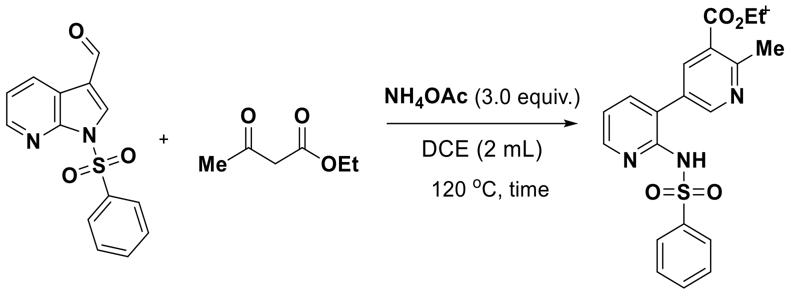

Entry	NH_4OAc (equiv.)	catalyst	time	Yield (A)[a]
1	3.0	none	12	52%
2	5.0	none	12	74%
3	5.0	none	8	63%
4	5.0	none	16	75%
5	5.0	none	24	71%
6[b]	5.0	none	16	76%
7	5.0	$BF_3{\cdot}OEt_2$ (1.0 equiv.)	8	71%
8	5.0	$BF_3{\cdot}OEt_2$ (1.0 equiv.)	12	74%
9	5.0	$BF_3{\cdot}OEt_2$ (1.0 equiv.)	16	81%
10	5.0	$BF_3{\cdot}OEt_2$ (2.0 equiv.)	16	83%
11	5.0	$Zn(OTf)_2$ (10 mol%)	16	73%
12	5.0	$Yb(OTf)_3$ (10 mol%)	16	82%
13	5.0	$AgCO_3$ (10 mol%)	16	92% (79%)
	Deviation from entry 3			
**14**	**no catalyst, NH_4OAc (6.0 equiv.)**		**16**	**89% (80%)**

Notes: [a]Crude NMR yields with respect to the peak at δ ~6.7 ppm using 1,3,5-trimethoxy benzene as an internal reference (isolated yields were written in the parenthesis); [b]2.0 equivalent of *β*-ketoester (**2a**).

Optimized condition in respect of ethyl acetoacetate (2a): A 4-mL vial equipped with a magnetic bar and a Teflon-lined screwed cap was charged with **1** (0.2 mmol), ethyl acetoacetate (1.2 equiv.), and NH_4OAc (92.53 mg, 6.0 equiv.) in DCE (2.0 mL). The vial was then sealed and heated at 120 ℃ for 16 h. Upon reaction completion checked by LC-MS and TLC analysis, the reaction mixture was diluted with dichloromethane (DCM), quenched with saturated aqueous $NaHCO_3$ solution, and extracted with DCM (3 × 10 mL). The combined organic fractions were dried over anhydrous Na_2SO_4(s), filtered, and concentrated under reduced pressure. Finally, the crude mixture was purified by silica-gel flash column chromatography to obtain the desired product.

The scope of this methodology was examined with various (aza)indole 3-carboxaldehydes with ethyl acetoacetate under the optimized condition, and we observed the formation of substituted pyridines *via* the ring cleavage of (aza)indoles in good to excellent yields. But changing the *β*-ketoester from ethyl acetoacetate (**2a**) to ethyl butyrylacetate (**2b**) significantly reduced the yield of the reaction down to ~30%, indicating that we needed further optimization in the case of ethyl butyrylacetate (**2b**) to synthesize diverse alkyl/aryl *o*-substituted pyridines. In general, 3-formyl azaindoles are more reactive than 3-formyl indoles. Hence, the reaction optimization was performed with *N*-phenylsulfonyl 3-formyl indole (**1a'**) with ethyl butyrylacetate (**2b**) as the model system

Supplementary Table 4. Reaction optimization for *N*-phenylsulfonyl 3-formyl indole (**1a'**) with ethyl butyrylacetate (**2b**)

Entry	NH_4OAc	catalyst	time	LC/MS yield	NMR yield[a]
1	4.0 equiv.	none	8 h	~36%	~29%
2	5.0 equiv.	none	8 h	~36%	~30%
3	6.0 equiv.	none	8 h	~36%	~30%
4	4.0 equiv.	$Zn(OTf)_2$ (30 mol%)	8 h	59%	47%
5	4.0 equiv.	$Zn(OTf)_2$ (30 mol%)	16 h	55%	50%
6	4.0 equiv.	$AlCl_3$ (30 mol%)	16 h	33%	-
7	4.0 equiv.	$AuCl_3$ (30 mol%)	16 h	33%	-
8	4.0 equiv.	$BF_3{\cdot}OEt_2$ (30 mol%)	16 h	34%	-
9	4.0 equiv.	No catalyst	16 h	41%	-
10	4.0 equiv.	AcOH (1.0 equiv.)	16 h	65%	55%
**11**	**4.0 equiv.**	**TFA (1.0 equiv.)**	**16 h**	**80%**	**74% (69%)**
12[b]	4.0 equiv.	TFA (1.0 equiv.)	16 h	88%	80% (70%)

Notes: [a]Crude NMR yields with respect to the peak at δ ~6.7 ppm using 1,3,5-trimethoxy benzene as an internal reference (isolated yields were written in the parenthesis); [b]The reaction of *N*-phenylsulfonyl 3-formyl azaindole (**1a**) with ethyl butyrylacetate (**2b**).

General procedure for the reaction of *N*-substituted 3-formyl (aza)indoles with diverse *β*-ketoesters (2a–2f): A 4-mL vial equipped with a magnetic bar and a Teflon-lined screwed cap was charged with **1** (0.2 mmol), *β*-ketoesters (**2a**–**2f**, 1.2 equiv.), trifluoroacetic acid (TFA, 22.80 mg, 14.86 μL, 1.0 equiv.), and NH_4OAc (61.66 mg, 4.0 equiv.) in DCE (2.0 mL). The vial was then sealed and heated at 120 °C for 16 h. Upon reaction completion checked by LC-MS and TLC analysis, the reaction mixture was diluted with DCM, quenched with saturated aqueous $NaHCO_3$ solution, and extracted with DCM (3 × 10 mL). The combined organic layer was washed with brine (10 mL), dried over anhydrous Na_2SO_4(s), filtered, and concentrated under reduced pressure. The crude mixture was purified by silica-gel flash column chromatography to obtain the desired product.

Note: The general procedure for the above methodology was slightly modified in the case of ethyl benzoylacetate (**2f**); the reaction was performed in ethanol without TFA.

(3) Use of *β*-keto sulfones/phosphonates as the enamine precursors:

After we successfully synthesized *meta*-arylamino-conjugated substituted nicotinates from various *β*-ketoesters and 3-formyl (aza)indoles *via* the ring cleavage reaction, we turned our attention to the synthesis of substituted pyridine analogs containing sulfone and phosphonate group at its *meta* position of the pyridine with our ring-cleavage methodology. For instance, we performed the reaction of *N*-phenylsulfonyl 3-formyl 7-azaindole (**1a**) with phenylsulfonylacetone (**4a**) under the optimized reaction conditions that were used for *β*-ketoesters (**2a–2f**), which led to the formation of the desired ring cleavage product (**6aa**) along with its regioisomer (**6aa'**). The regioisomer **6aa'** can be formed *via* this methodology reaction of 3-formyl 7-azaindole (**1a**) with a terminal enamine obtained as a major enamine from phenylsulfonylacetone (**4a**) (see Supplementary Figure 2). A similar reactivity pattern of (aza)indole cleavage reaction was observed with 3-formyl 7-azaindole (**1a**) in the case of diethyl (2-oxopropyl)phosphonate (**5a**), which also provided the regioisomer **7aa'** along with our desired product (**7aa**) (see Supplementary Figure 3). The *in situ* enamine formation from diethyl (2-oxopropyl)phosphonate (**5a**) in the presence of ammonium acetate allowed the formation of terminal enamine as a major form along with internal enamine as a minor form.

Supplementary Figure 2. The ring cleavage reaction of *N*-phenylsulfonyl 3-formyl 7-azaindole (**1b**) with phenylsulfonylacetone (**4a**)

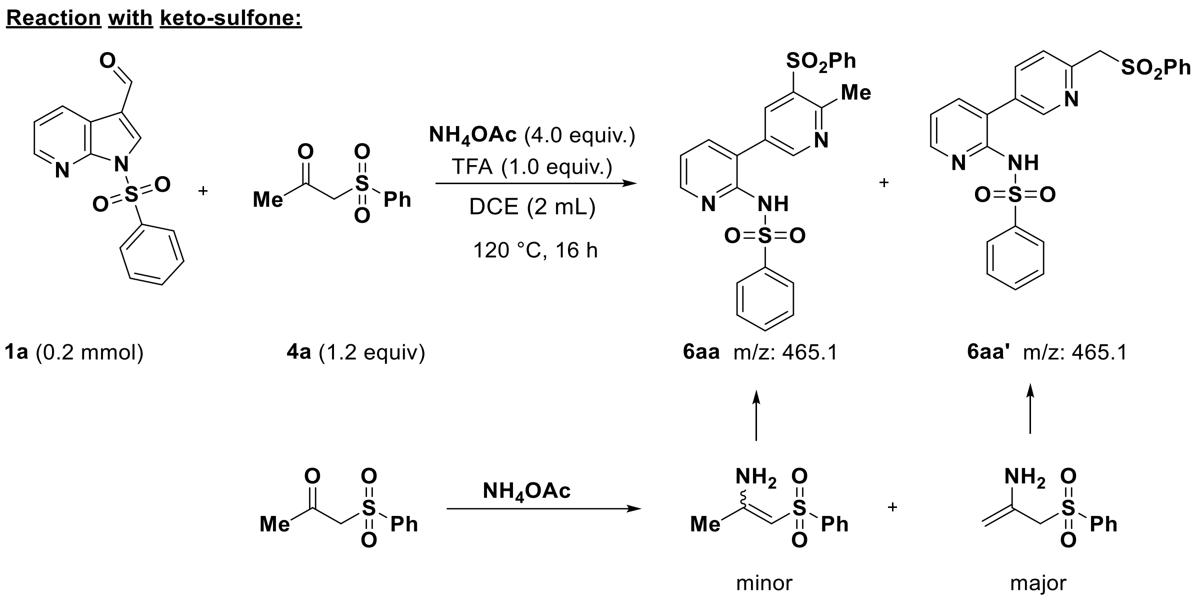

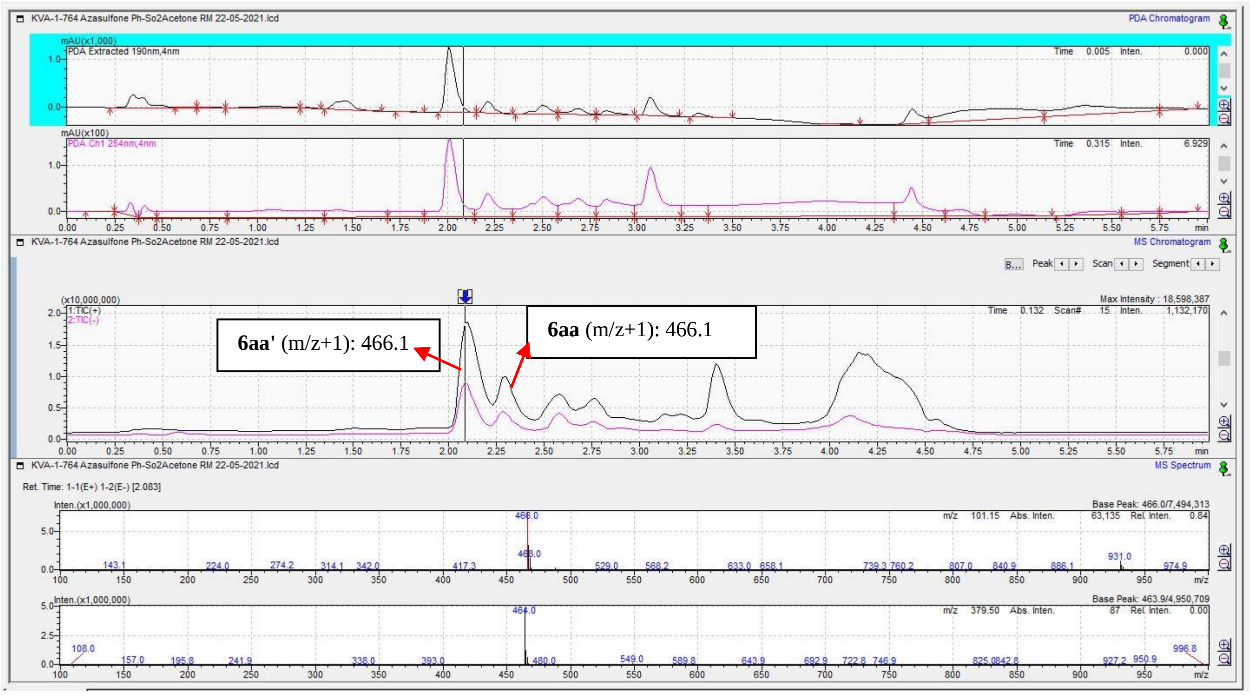

Supplementary Figure 3. The ring cleavage reaction of *N*-phenylsulfonyl 3-formyl 7-azaindole (**1b**) with diethyl (2-oxopropyl)phosphonate (**5a**).

Reaction with keto-phosphonate:

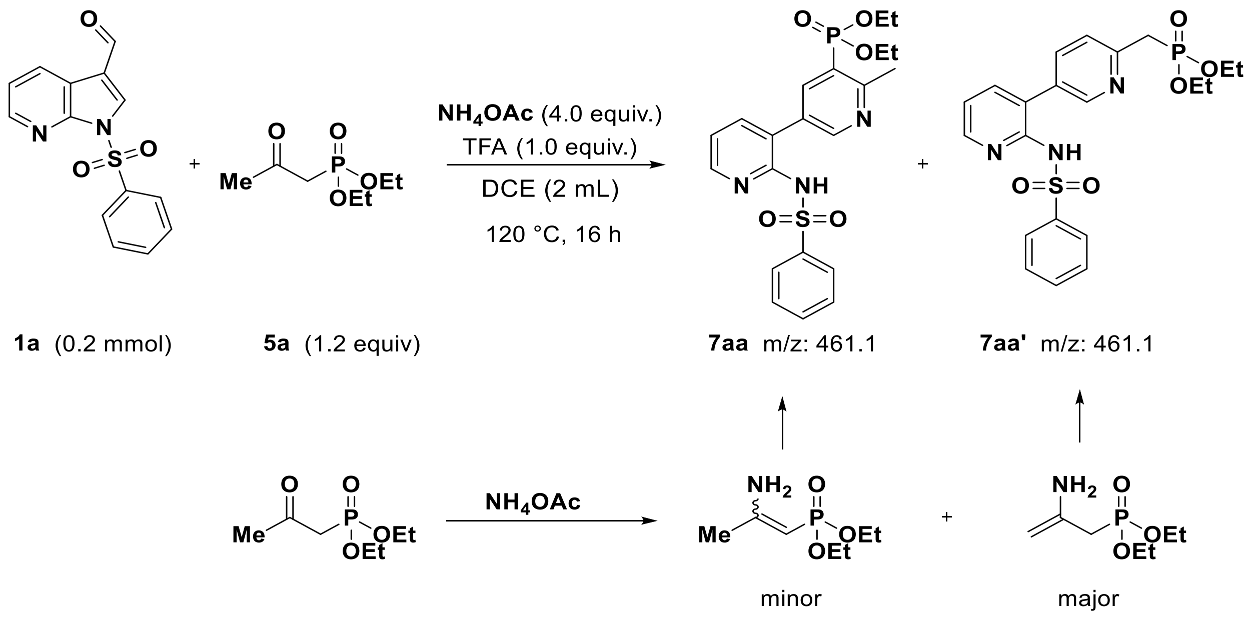

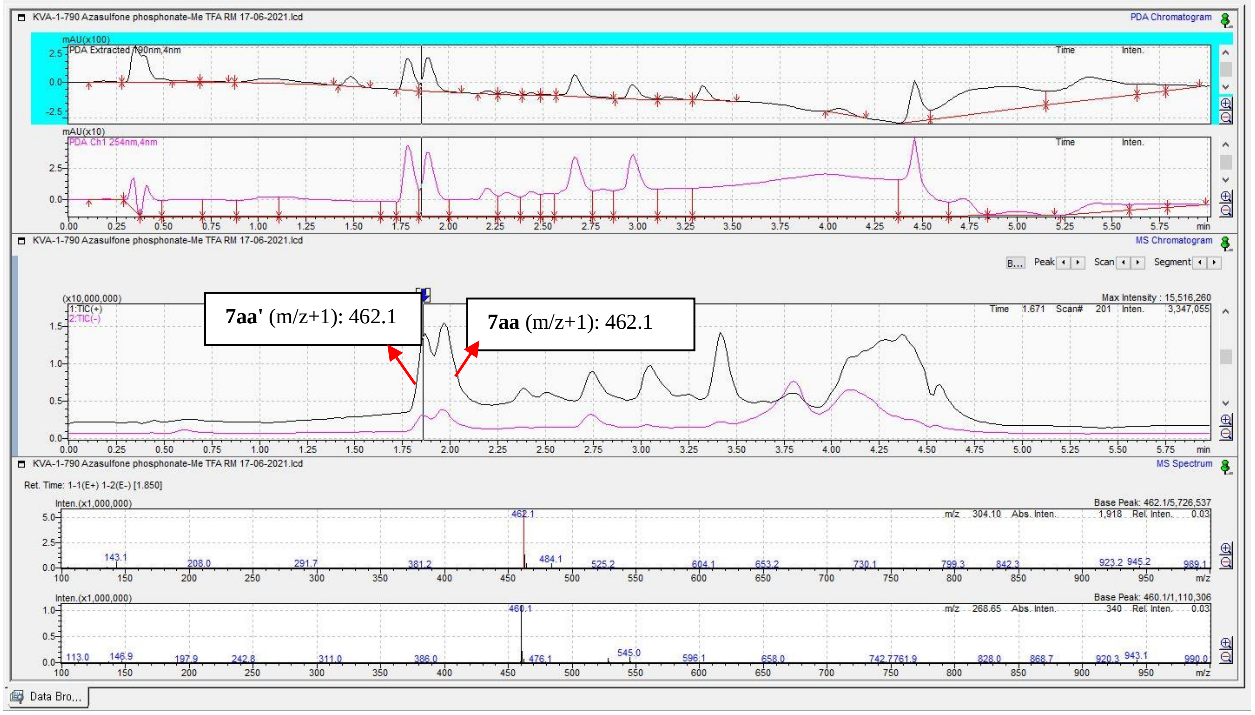

Unlike *β*-ketoesters, *β*-ketosulfones and *β*-ketophosphonates caused the regioselectivity issue in the *in situ* enamine formation leading to the generation of regioisomeric mixtures along with low yields and slow reaction kinetics. To overcome this regioselectivity issue of *in situ* enamine formation, we used **4b** (-*i*Pr) and **4c** (-Ph) for *β*-ketosulfones and **5b** (-*i*Pr) and **5c** (-Ph) for *β*-ketophosphonates. Accordingly, the ring cleavage reaction pattern of 3-formyl (aza)indoles was examined with *β*-ketosulfones (**4b** and **4c**) and *β*-ketophosphonates (**5b** and **5c**). Importantly, when we increased the equivalence of ammonium acetate, the reaction rate was significantly improved. The extended reaction time allowed the completion of the ring cleavage reaction. In the case of *β*-ketosulfones (**4b** and **4c**), the reaction concentration was increased from 0.1 M to 0.2 M due to their slow reaction kinetics compared to that of *β*-ketophosphonates (**5b** and **5c**).

Supplementary Figure 4. Ring cleavage reaction of *N*-phenylsulfonyl 3-formyl 7-azaindoles with *β*-ketosulfones (**4b** and **4c**)/*β*-ketophosphonates (**5b** and **5c**).

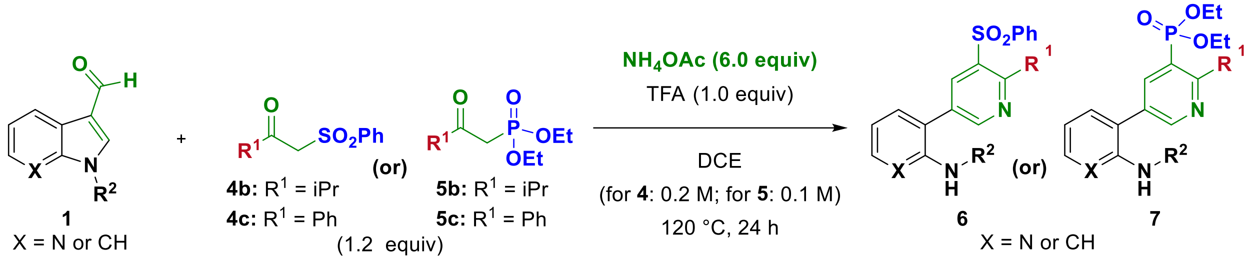

General procedure for the reaction of *N*-substituted 3-formyl (aza)indole with *β*-ketosulfones (4a–b)/*β*-ketophosphonates (5a–b): A 4-mL vial equipped with a magnetic bar and a Teflon-lined screwed cap was charged with **1** (0.2 mmol), *β*-keto sulfones/phosphonates (**4b–c/5b–c**, 1.2 equiv.), TFA (22.80 mg, 14.86 µL, 1.0 equiv.), and NH_4OAc (92.50 mg, 6.0 equiv.) in DCE (1.0 mL (**4b–c**)/2.0 mL (**5b–c**)). The vial was then sealed and heated at 120 °C for 16 h to 48 h. Upon reaction completion checked by LC-MS and TLC analysis, the reaction mixture was diluted with DCM, quenched with saturated aqueous $NaHCO_3$ solution, and extracted with DCM (3 × 10 mL). The combined organic fraction was washed with brine (10 mL), dried over anhydrous Na_2SO_4(s), filtered, and concentrated under reduced pressure. The crude compound was purified by silica-gel flash column chromatography to obtain the desired product bearing 3-pyridylsulfones (**6**)/3-pyridyl phosphonates (**7**).

(4) Extension of ring cleavage methodology toward benzofuran analogs:

We extended the scope of our ring cleavage methodology to 3-formyl benzofurans for the synthesis of *meta*-*o*-phenol-conjugated substituted nicotinates. The reaction was studied with commercially available representative *β*-ketoesters (ethyl acetoacetate **2a**, ethyl 3-cyclopropyl-3-oxopropionate **2d**, and ethyl benzoylacetate **2f**) under the optimized conditions to furnish the ring cleavage products in good to excellent yields (**9**) from substituted 3-formyl benzofurans (**8a–8j**).

Supplementary Figure 5. The reaction of substituted 3-formyl benzofurans with *β*-ketoesters.

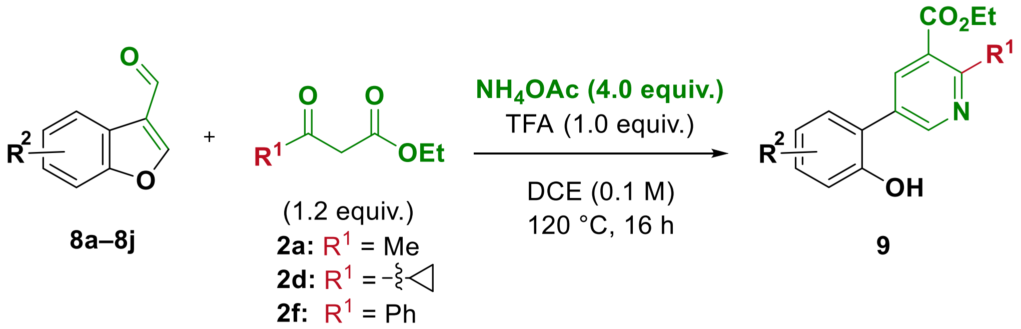

General procedure for the reaction of benzofuran-3-carboxaldehydes with *β*-ketoesters. A 4-mL vial equipped with a magnetic bar and a Teflon-lined screwed cap was charged with **8** (0.2 mmol), *β*-ketoesters (**2a**, **2d**, or **2f**, 1.2 equiv.), TFA (22.80 mg, 14.86 μL, 1.0 equiv.), and NH_4OAc (61.66 mg, 4.0 equiv.) in DCE (2.0 mL). The vial was then sealed and heated at 120 ℃ for 16 h. Upon reaction completion checked by LC-MS and TLC analysis, the reaction mixture was diluted with DCM, quenched with saturated aqueous $NaHCO_3$ solution, and extracted with DCM (3 × 10 mL). The combined organic fraction was washed with brine (10 mL), dried over anhydrous Na_2SO_4(s), filtered, and concentrated under reduced pressure. The crude compound was purified by silica-gel flash column chromatography to obtain the desired phenol-conjugated product (**9**).

Supplementary Methods

II. General Information

NMR spectra were obtained on an Agilent 400-MR DD2 Nuclear Magnetic Resonance System (400 MHz, Agilent, USA), Varian/Oxford Unity Inova 500 MHz (Varian Assoc., Palo Alto, USA), and Bruker AVANCE 500 (500 MHz, Bruker, Germany). Chemical shifts values were recorded as parts per million (δ), referenced to tetramethylsilane (TMS) as the internal standard or to the residual solvent peak ($CDCl_3$ ^{13}C: 77.16; CD_2Cl_2 ^{13}C: 53.84; CD_3OD ^{13}C: 49.00; DMSO-d_6 ^{13}C: 39.52). Multiplicities were indicated as follows: s (singlet), d (doublet), t (triplet), q (quartet), m (multiplet), dd (doublet of doublet), dt (doublet of triplet), td (triplet of doublets), br. s (broad singlet), and so on. Coupling constants were reported in hertz (Hz). Low-resolution mass spectrometry (LRMS) of all synthesized compounds was obtained with LC/MS-2020 [Shimadzu]. High-resolution mass spectrometry (HRMS) of final compounds was obtained with ThermoFisher Scientific (Orbitrap Exploris 120) from the Chemistry department at Seoul National University. All commercially available reagents were used without further purification unless noted otherwise. Commercially available reagents were obtained from Sigma-Aldrich, TCI, Acros, or Alfa Aesar. All anhydrous solvents were obtained through activated alumina columns of solvent purification systems from Glass Contour. Analytical thin-layer chromatography (TLC) was performed using Merck Kiselgel 60 F_{254} plates. The components in TLC were visualized by observation under UV light (254 and 365 nm) or by treating the plates with ninhydrin or $KMnO_4$ followed by thermal visualization. Flash column chromatography was performed on Merck Kieselgel 60 (230-400 mesh) Biotage® Selekt and Isolera One (ZIP® KP-Sil and Sfär columns were used). Microwave reactions were performed using the Biotage® Robot Eight [Biotage] and microwave reaction conditions were as indicated in the Experimental Section. Infrared (IR) spectra were recorded on a PerkinElmer Spectrum IR Version 10.6.0. as a neat sample and are reported in the frequency of absorption (cm^{-1}). HPLC purification was performed on an Agilent 1260 Infinity system [Agilent] with a YMC-Pack silica column (SL12S05-2520WTX, 250 × 20 mm, 5 μm).

III. General Synthetic Procedures and Characterization of All Starting Materials

Supplementary Figure 6. Starting materials used in this methodology

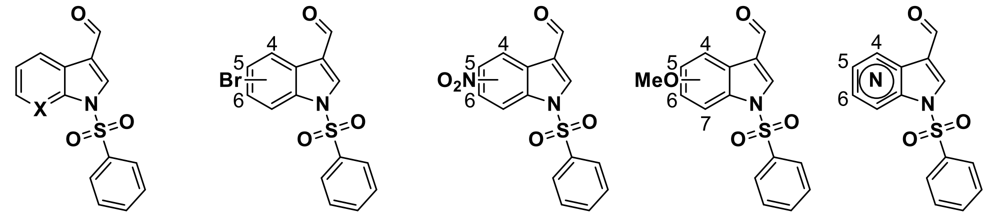

1a: X = N
1a': X = CH

1b: R = 4-Br
1c: R = 5-Br
1d: R = 6-Br

1e: R = 4-NO_2
1f: R = 5-NO_2
1g: R = 6-NO_2

1h: R = 4-MeO
1i: R = 5-MeO
1j: R = 6-MeO
1k: R = 7-MeO

1l: 4-N
1m: 5-N
1n: 6-N

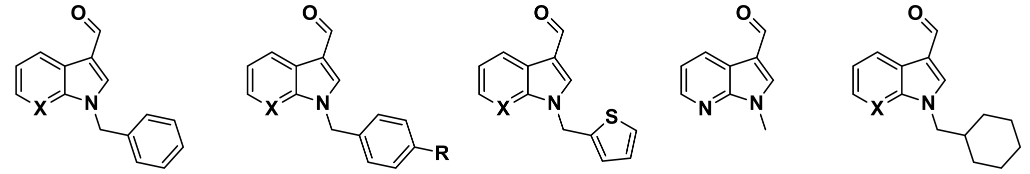

1o: X = N
1o': X = CH

1p: X = N; R = *p*-Cl
1q: X = N; R = *p*-OMe
1q': X = CH; R = *p*-OMe

1r: X = N
1r': X = CH

1s

1t: X = N
1t': X = CH

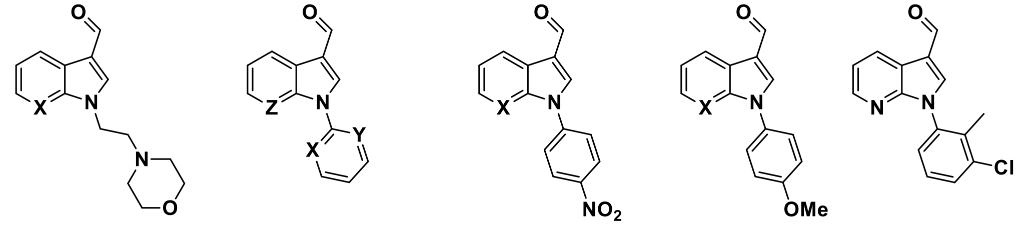

1u: X = N
1u': X = CH

1v: X = Y = CH; Z = N
1w: X = CH;Y = N; Z = N
1x: X = Y = N; Z = N
1x': X = Y = N; Z = CH

1y: X = N
1y': X = CH

1z: X = N
1z': X = CH

1aa

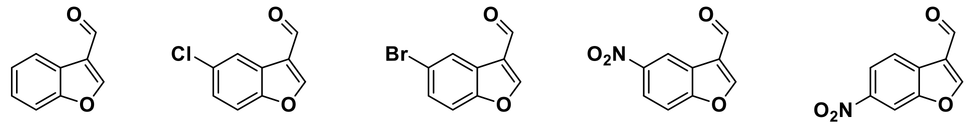

8a **8b** **8c** **8d** **8e**

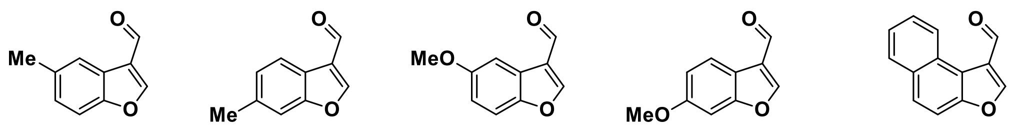

8f **8g** **8h** **8i** **8j**

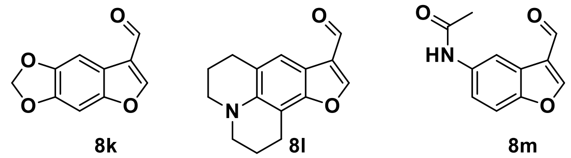

8k **8l** **8m**

Supplementary Figure 7. *β*-keto esters/sulfones/phosphonates used in this methodology.

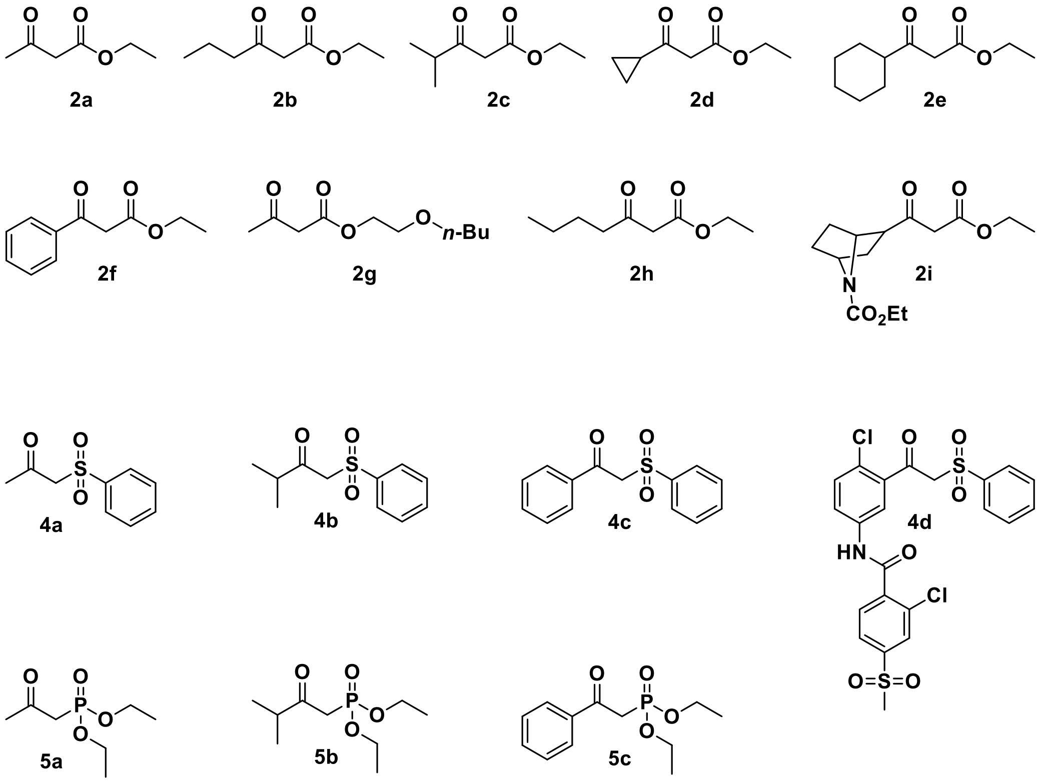

Supplementary Figure 8. The following compounds (starting materials) were synthesized using the procedure reported in the literature[1]:

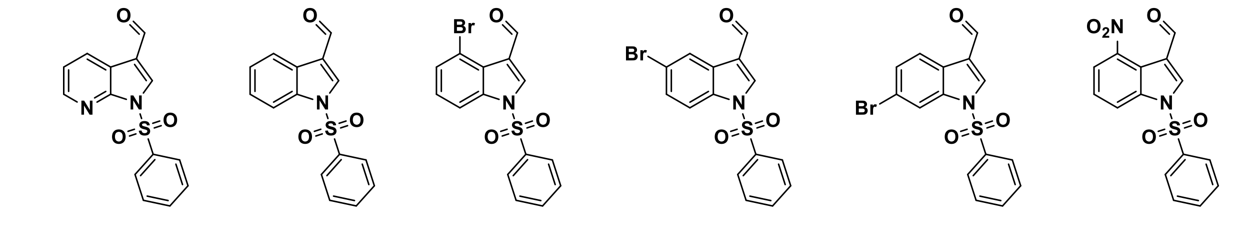

1a 1a' (Commercially Available) 1b 1c 1d 1e

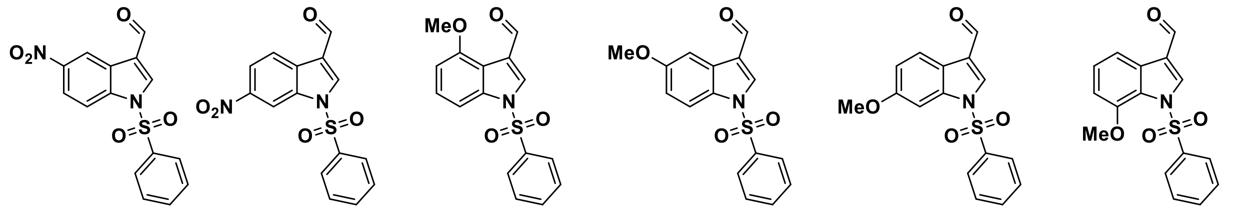

1f 1g 1h 1i 1j 1k

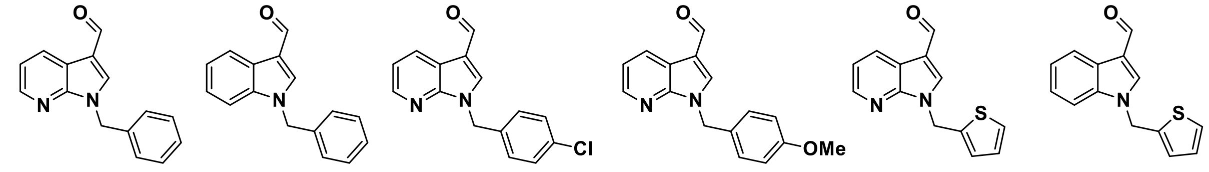

1o 1o' 1p 1q 1r 1r'

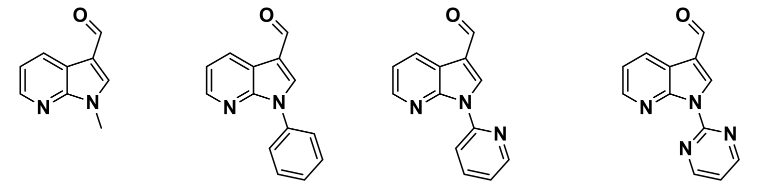

1s 1v 1w 1x

Supplementary Figure 9. Syntheses of *N*-phenylsulfonyl azaindole-3-carboxaldehydes (**1l–1n**)

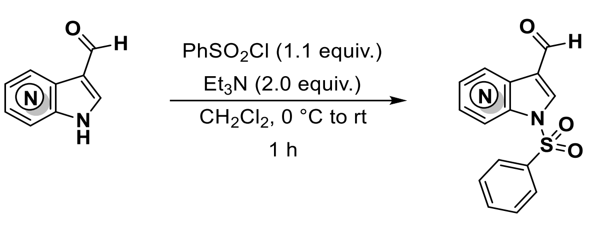

General Procedure: A round bottom flask placed under the ice bath, equipped with a magnetic bar, was charged with azaindole-3-carbaldehyde derivative (1.0 equiv.), Et_3N (2.0 equiv.), and DCM at 0 °C. To this cold suspension, benzenesulfonyl chloride (1.1 equiv.) was added dropwise, and the reaction mixture was

stirred at r.t. for 1 h. The progress of the reaction was monitored by TLC and LC-MS. After completion of the reaction, the reaction mixture was diluted with water and extracted with DCM (3 × 20 mL). The combined organic layer was washed with brine, dried over anhydrous Na_2SO_4(s), filtered, and evaporated to give a crude product. The crude product was purified by silica-gel flash column chromatography using EtOAc/hexanes mixture as eluent.

Compound 1l: 1-(phenylsulfonyl)-1*H*-pyrrolo[3,2-*b*]pyridine-3-carbaldehyde

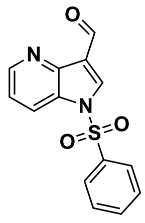

1l

Following the general procedure described above with 1*H*-pyrrolo[3,2-*b*]pyridine-3-carbaldehyde (3-formyl 4-azaindole)[2] (0.12 g, 0.82 mmol) provided **1l** in 94% yield (0.22 g) as off-white solid; ^{1}H NMR (400 MHz, $CDCl_3$): δ 10.38 (s, 1H), 8.71 (d, *J* = 4.8 Hz, 1H), 8.46 (s, 1H), 8.30 (d, *J* = 8.4 Hz, 1H), 7.97 (d, *J* = 7.6 Hz, 2H), 7.66 (t, *J* = 7.6 Hz, 1H), 7.55 (t, *J* = 7.6 Hz, 2H), 7.37 (dd, *J* = 8.8, 4.8 Hz, 1H); ^{13}C NMR (100 MHz, $CDCl_3$): 184.94, 147.90, 145.03, 137.18, 135.26, 134.73, 130.03, 128.87, 127.24, 121.66, 121.25, 120.53; LRMS (ESI): *m/z* calcd for $C_{14}H_{11}N_2O_3S$ $[M+H]^+$: 287.05; Found: 287.00.

Compound 1m: 1-(phenylsulfonyl)-1*H*-pyrrolo[3,2-*c*]pyridine-3-carbaldehyde

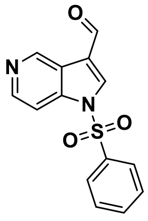

1m

Following the general procedure described above with 1*H*-pyrrolo[3,2-*c*]pyridine-3-carbaldehyde (3-formyl 5-azaindole)[2] (0.126 g, 0.86 mmol) provided **1m** in 81% yield (0.20 g) as off-white solid; ^{1}H NMR (400 MHz, $CDCl_3$): δ 10.12 (s, 1H), 9.52 (s, 1H), 8.62 (d, *J* = 6.0 Hz, 1H), 8.26 (s, 1H), 8.00 (dd, *J* = 7.6, 1.6 Hz, 2H), 7.92–7.86 (m, 1H), 7.68 (t, *J* = 7.6 Hz, 1H), 7.57 (t, *J* = 7.6 Hz, 2H); ^{13}C NMR (100 MHz, $CDCl_3$): 184.54, 145.83, 145.59, 139.99, 137.07, 136.06, 135.47, 130.16, 127.39, 122.70, 121.98, 108.32; LRMS (ESI): *m/z* calcd for $C_{14}H_{11}N_2O_3S$ $[M+H]^+$: 287.05; Found: 287.00.

Compound 1n: 1-(phenylsulfonyl)-1*H*-pyrrolo[2,3-*c*]pyridine-3-carbaldehyde

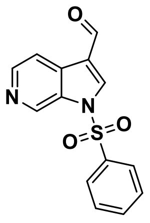
1n

Following the general procedure described above with 1*H*-pyrrolo[2,3-*c*]pyridine-3-carbaldehyde (3-formyl 6-azaindole)[2] (0.12 g, 0.82 mmol) provided **1n** in 76% yield (0.18 g) as white solid; ^{1}H NMR (400 MHz, $CDCl_3$): δ 10.13 (s, 1H), 9.34 (s, 1H), 8.56 (d, *J* = 5.2 Hz, 1H), 8.34 (s, 1H), 8.14 (d, *J* = 5.2 Hz, 1H), 8.03 (d, *J* = 7.6 Hz, 2H), 7.68 (t, *J* = 7.6 Hz, 1H), 7.56 (t, *J* = 7.6 Hz, 2H); ^{13}C NMR (100 MHz, $CDCl_3$): 184.76, 144.65, 137.92, 137.02, 135.94, 135.42, 132.21, 130.16, 127.47, 121.60, 116.89; LRMS (ESI): *m/z* calcd for $C_{14}H_{11}N_2O_3S$ $[M+H]^+$: 287.05; Found: 287.00.

Compound 1q': 1-(4-methoxybenzyl)-1*H*-indole-3-carbaldehyde

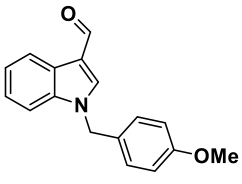
1q'

Compound **1q'** was prepared using the procedure reported in the literature.[3]

Supplementary Figure 10. Syntheses of *N*-cyclohexylmethyl (aza)indole-3-carboxaldehydes (**1t, 1t'**)

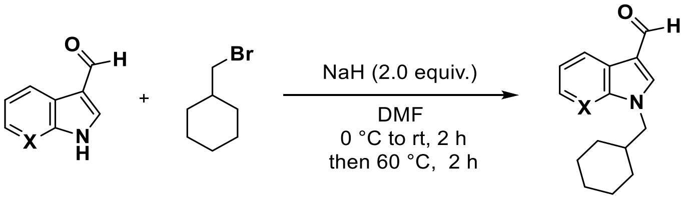

General procedure: To a pre-cooled solution of (aza)indole-3-carboxaldehyde (1.0 equiv.) in anhydrous DMF at 0 °C, was added NaH (2.0 equiv., 60% suspension in mineral oil) in a portion-wise under argon atmosphere. After the complete addition of NaH, the reaction mixture was warmed to r.t. and stirred 0.5 h. After the reaction mixture was cooled to 0 °C, cyclohexylmethyl bromide (1.1 equiv.) was added dropwise, and the reaction mixture was allowed warm to r.t. and stirred for 2 h. The progress of the reaction was monitored by TLC and LC-MS. When the full conversion was not observed, the reaction mixture was

heated at 60 ℃ for 2 h. After completion of the reaction (monitored by TLC and LC-MS), the reaction mixture was diluted with ice-cold water (20 mL) and extracted with EtOAc (3 × 20 mL). The combined organic layer was washed with brine, dried over anhydrous Na_2SO_4(s), filtered, and evaporated to give a crude product. The crude product was purified by silica-gel flash column chromatography using EtOAc/hexanes mixture as eluent.

Compound 1t: 1-(cyclohexylmethyl)-1*H*-pyrrolo[2,3-*b*]pyridine-3-carbaldehyde

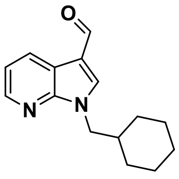

1t

Following the general procedure described above with 7-azaindole-3-carboxaldehyde (0.51 g, 3.42 mmol) provided **1t** in 70% yield (0.58 g, 2.39 mmol) as off-white solid; ^{1}H NMR (400 MHz, $CDCl_3$): δ 9.98 (s, 1H), 8.55 (d, *J* = 8.0 Hz, 1H), 8.42 (d, *J* = 4.8 Hz, 1H), 7.83 (s, 1H), 7.32–7.19 (m, 1H), 4.18 (d, *J* = 7.2 Hz, 2H), 2.03–1.90 (m, 1H), 1.78–1.59 (m, 5H), 1.30–1.14 (m, 3H), 1.10–0.95 (m, 2H); ^{13}C NMR (100 MHz, $CDCl_3$): 184.63, 148.80, 145.09, 138.72, 130.48, 118.86, 117.73, 116.11, 51.79, 38.33, 30.79, 26.26, 25.64; LRMS (ESI): *m/z* calcd for $C_{15}H_{19}N_2O$ $[M+H]^+$: 243.15; Found: 243.1.

Compound 1t': 1-(cyclohexylmethyl)-1*H*-indole-3-carbaldehyde

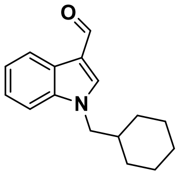

1t'

Following the general procedure described above with indole-3-carboxaldehyde (0.51 g, 3.44 mmol) provided **1t'** in 99% yield (0.83 g) as pink solid; ^{1}H NMR (400 MHz, $CDCl_3$): δ 9.99 (s, 1H), 8.31 (d, *J* = 7.2 Hz, 1H), 7.64 (s, 1H), 7.41–7.26 (m, 3H), 3.97 (d, *J* = 7.2 Hz, 2H), 1.94–1.79 (m, 1H), 1.75–1.58 (m, 5H), 1.28–1.10 (m, 3H), 1.09–0.93 (m, 2H); ^{13}C NMR (100 MHz, $CDCl_3$): 184.57, 139.01, 137.61, 125.48, 123.91, 122.84, 122.14, 117.91, 110.42, 53.82, 38.36, 31.00, 26.20, 25.65; LRMS (ESI): *m/z* calcd for $C_{16}H_{20}NO$ $[M+H]^+$: 242.15; Found: 242.2.

Supplementary Figure 11. Syntheses of *N*-morpholinoethyl protected (aza)indole-3-carboxaldehydes (**1u, 1u'**)

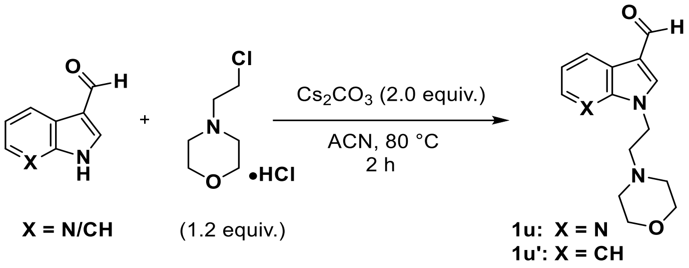

General procedure: 20-mL sealed vial was charged with (aza)indole-3-carboxaldehyde (1.0 equiv.), *N*-(2-chloroethyl) morpholine hydrochloride (1.2 equiv.), and cesium carbonate (2.0 equiv.) in ACN (20 mL). The reaction mixture was heated at 80 ℃ for 2 h. After completion of the reaction (monitored by TLC and LC-MS), the resulting mixture was diluted with ice-cold water (20 mL) and extracted with EtOAc (3 × 20 mL). The combined organic layer was washed with brine, dried over anhydrous Na_2SO_4(s), filtered, and evaporated to give a crude product. The crude product was purified by silica-gel flash column chromatography using EtOAc/hexanes mixture as eluent.

Compound 1u: 1-(2-morpholinoethyl)-1*H*-pyrrolo[2,3-*b*]pyridine-3-carbaldehyde

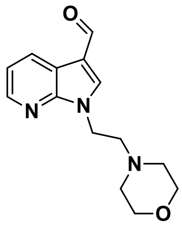

1u

Following the general procedure described above with 7-azaindole-3-carboxaldehyde (0.5 g, 3.42 mmol) provided **1u** in 82% yield (0.73 g) as white crystalline solid; ^{1}H NMR (400 MHz, $CDCl_3$): δ 9.99 (s, 1H), 8.55 (d, *J* = 8.0 Hz, 1H), 8.41 (d, *J* = 4.8 Hz, 1H), 8.00 (s, 1H), 7.26 (dd, *J* = 8.4, 4.8 Hz, 1H), 4.47 (t, *J* = 6.0 Hz, 2H), 3.69 (t, *J* = 4.4 Hz, 4H), 2.83 (t, *J* = 6.0 Hz, 2H), 2.53 (t, *J* = 4.4 Hz, 4H); ^{13}C NMR (100 MHz, $CDCl_3$): 184.76, 148.56, 145.02, 138.97, 130.59, 119.00, 117.70, 116.41, 67.04, 57.93, 53.71, 42.18; LRMS (ESI): *m/z* calcd for $C_{14}H_{18}N_3O_2$ $[M+H]^+$: 260.14; Found: 260.1.

Compound 1u': 1-(2-morpholinoethyl)-1*H*-indole-3-carbaldehyde

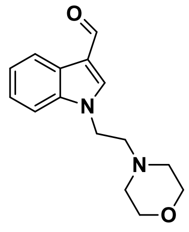

1u'

Following the general procedure described above with indole-3-carboxaldehyde (0.5 g, 3.44 mmol) provided **1u'** in 100% yield (0.89 g) as off-white solid; ^{1}H NMR (400 MHz, $CDCl_3$): δ 10.00 (s, 1H), 8.37–8.27 (m, 1H), 7.81 (s, 1H), 7.42–7.27 (m, 3H), 4.27 (t, J = 6.4 Hz, 2H), 3.69 (t, J = 4.4 Hz, 4H), 2.79 (t, J = 6.4 Hz, 2H), 2.49 (t, J = 4.4 Hz, 4H); ^{13}C NMR (100 MHz, $CDCl_3$): 184.66, 138.99, 137.26, 125.44, 124.07, 123.04, 122.30, 118.31, 109.91, 67.01, 57.69, 53.87, 44.58; LRMS (ESI): *m/z* calcd for $C_{15}H_{19}N_2O_2$ $[M+H]^+$: 181.60; Found: 181.10.

Supplementary Figure 12. Synthesis of *N*-*p*-NO_2-phenyl (aza)indole-3-carboxaldehydes (**1y, 1y'**)

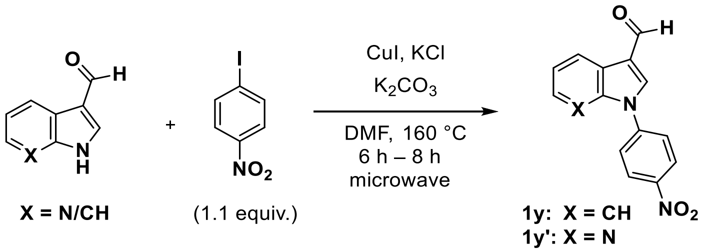

General procedure: 10-mL microwave vial was charged with (aza)indole-3-carboxaldehdye (1.0 mmol), KCl (1.0 equiv.), K_2CO_3 (3.0 equiv.), 1-iodo-4-nitro-benzene (1.1 equiv.), and CuI (0.1 equiv.) in DMF (4 mL). The reaction mixture was stirred at 160 ℃ for 6 h (8h for **1y'**) in a microwave reactor. After completion of the reaction (monitored by TLC), the resulting mixture was diluted with saturated NH_4Cl solution (10 mL) and extracted with EtOAc (2 × 10 mL). The organic extracts were washed with brine (10 mL), dried over Na_2SO_4(s), filtered, and evaporated to give a crude product. The crude product was purified by silica-gel flash column chromatography using EtOAc and hexanes (1:3) as an eluent furnishing the title compound **1y** (68%) and **1y'** (65%)

Compound 1y': 1-(4-nitrophenyl)-1*H*-pyrrolo[2,3-*b*]pyridine-3-carbaldehyde

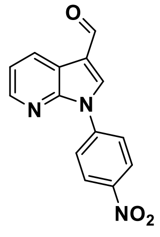

1y

^{1}H NMR (500 MHz, DMSO-d_6): δ 10.08 (s, 1H), 9.11 (s, 1H), 8.55 (dd, *J* = 8.0, 2.0 Hz, 1H), 8.51 (dd, *J* = 4.4, 1.5 Hz, 1H), 8.47 (d, *J* = 9.0 Hz, 2H), 8.35 (d, *J* = 9.0 Hz, 2H), 7.48 (dd, *J* = 8.0, 4.5 Hz, 1H); ^{13}C NMR (125 MHz, DMSO-d_6): δ 185.89, 147.89, 145.48, 142.13, 139.41, 130.31, 124.90, 124.10, 120.18, 118.08, 117.76; LRMS (ESI): *m/z* calcd for $C_{14}H_{10}N_3O_3$ [M+H]$^+$: 268.07; Found: 268.1.

Compound 1y: 1-(4-nitrophenyl)-1*H*-indole-3-carbaldehyde

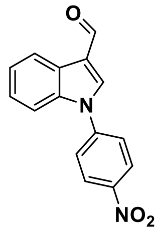

1y'

^{1}H NMR (500 MHz, CD_2Cl_2): δ 10.06 (s, 1H), 8.38 (d, J = 9.0 Hz, 2H), 8.30–8.24 (m, 1H), 7.93 (s, 1H), 7.70 (d, J = 8.5 Hz, 2H), 7.56–7.49 (m, 1H), 7.37–7.29 (m, 2H); ^{13}C NMR (125 MHz, CD_2Cl_2): δ 185.29, 147.06, 143.97, 137.84, 137.24, 126.33, 126.00, 125.60, 125.26, 124.39, 122.75, 121.28, 111.31; LRMS (ESI): *m/z* calcd for $C_{15}H_{11}N_2O_3$ [M+H]$^+$: 267.08; Found: 267.1.

Supplementary Figure 13. Synthesis of *N*-*p*-OMe-phenyl (aza)indole-3-carboxaldehydes (**1z, 1z'**)

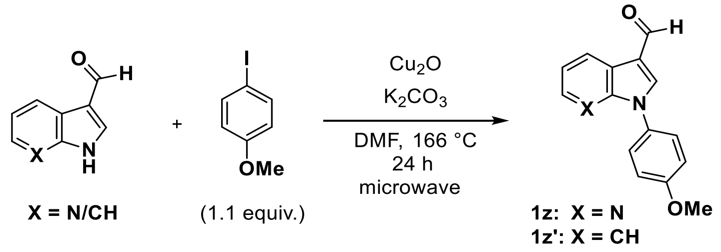

General procedure: To a stirred solution of (aza)indole 3-carboxaldehyde (5.0 mmol) in dry DMF (10 mL) were added dicopper oxide (217.7 mg, 1.50 mmol, 0.3 equiv.), potassium carbonate (1.38 g, 10.00 mmol), and 1-iodo-4-methoxy-benzene (2.34 g, 10.00 mmol, 2.0 equiv.) sequentially in a microwave vial. The reaction mixture was heated at 166 °C for 24 h in a microwave reactor. After completion of the reaction (monitored by TLC), the reaction mixture was cooled to r.t. and filtrated through a short pad of Celite eluting with EtOAc. The filtrate was washed with brine, and the organic phase was dried over Na_2SO_4(s) and concentrated. The crude residue was purified by silica-gel flash column chromatography (hexanes/EtOAc = 8:1) to provide the desired product **1z** (33%, off-white solid) and **1z'** (72%, off-white solid).

Compound 1z: 1-(4-methoxyphenyl)-1*H*-pyrrolo[2,3-*b*]pyridine-3-carbaldehyde

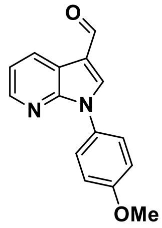

1z

^{1}H NMR (400 MHz, $CDCl_3$): δ 10.07 (s, 1H), 8.63 (dt, *J* = 8.0, 1.6 Hz, 1H), 8.45 (dd, *J* = 4.8, 1.6 Hz, 1H), 8.05 (s, 1H), 7.61 (d, *J* = 9.2 Hz, 2H), 7.31 (dd, *J* = 8.0, 4.8 Hz, 1H), 7.08 (d, *J* = 8.8 Hz, 2H), 3.88 (s, 3H); ^{13}C NMR (100 MHz, $CDCl_3$): 184.97, 159.41, 148.86, 145.85, 138.07, 130.90, 130.01, 126.25, 119.50, 118.14, 117.35, 114.98, 55.77; LRMS (ESI): *m/z* calcd for $C_{15}H_{13}N_2O_2$ $[M+H]^+$: 253.10; Found: 253.0.

Compound 1z': 1-(4-methoxyphenyl)-1*H*-indole-3-carbaldehyde

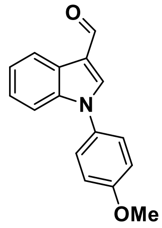

1z'

Compound **1va** was prepared using the above procedure and the NMR was matched with the data reported in the literature[4].

Compound 1aa: 1-(3-chloro-2-methylphenyl)-1*H*-pyrrolo[2,3-*b*]pyridine-3-carbaldehyde

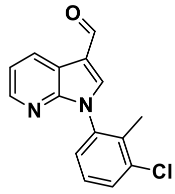

1aa

Compound **1aa** was prepared using the above procedure (2.0 mmol, 292 mg) in 12% yield (65mg) as a white solid. ^{1}H NMR (400 MHz, $CDCl_3$): δ 10.08 (s, 1H), 8.66 (dd, *J* = 8.0, 1.6 Hz, 1H), 8.43 (dd, *J* = 4.8, 1.6 Hz, 1H), 7.91 (s, 1H), 7.57 (dd, *J* = 7.6, 1.6 Hz, 1H), 7.37–7.25 (m, 3H), 2.11 (s, 3H); ^{13}C NMR (100 MHz, $CDCl_3$): δ 185.03, 149.25, 146.26, 138.65, 137.09, 136.26, 134.81, 131.17, 130.68, 127.50, 126.71, 119.56, 117.80, 117.44, 15.77; LRMS (ESI): *m/z* calcd for $C_{15}H_{12}ClN_2O$ $[M+H]^+$: 271.06; Found: 271.0.

Compound 8a: benzofuran-3-carbaldehyde

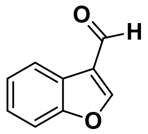

8a

Compound **8a** (148 mg, 49%) was prepared from commercially available 3-methyl benzofuran (264 mg, 2.0 mmol) using the procedure reported in the literature[5].

Supplementary Figure 14. Synthesis of benzofuran-3-carboxaldehydes as starting materials

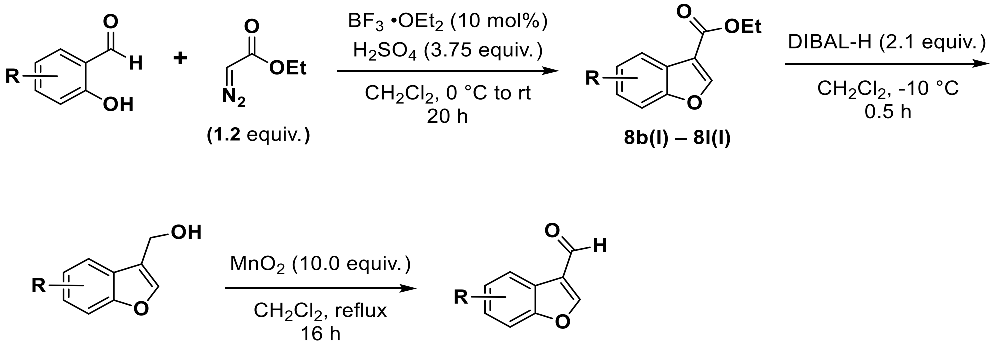

General Synthetic Procedure for 8b–8l:

1) Substituted benzofuran-3-ester, **8b(I)–8l(I)**, were synthesized using the general procedure reported in the literature[6]. In a typical experiment,

To the pre-cooled solution of commercially available salicylaldehyde (6.0 mmol) in CH_2Cl_2 (12 mL; 0.5 M) at 0 ℃, $BF_3{\cdot}OEt_2$ (10 mol%) was added and allowed to stir for 5 min. Then, ethyl diazoacetate (1.2 equiv. diluted with DCM about 0.5 M; 14 mL) was added dropwise to the reaction mixture. The resulting mixture was then warmed to room temperature and stirred for 12 h. After the reaction completion monitored by TLC and LC-MS, the volatiles were removed under reduced pressure. Then, concentrated H_2SO_4 (3.5 equiv.) solution was added dropwise to the remaining condensed residue and stirred for 30 min. The reaction mixture was diluted with CH_2Cl_2 and quenched with saturated aqueous $NaHCO_3$ solution until p*H* 7. The resulting mixture was extracted with DCM (2 × 10 mL). The combined organic layer was washed with brine, dried over anhydrous Na_2SO_4(s), filtered, and evaporated to provide a crude product. The desired products were isolated by silica-gel flash column chromatography using 5–15% ethyl acetate (EtOAc) in hexanes as eluents.

2) Substituted benzofuran-3-carbinol **8b(II)–8l(II)**: DIBAL-H reduction

To a stirred solution of substituted benzofuran 3-ester (5.0 mmol) obtained above in DCM (20 mL) was added a 1.0 M solution of DIBAL-H in toluene (10.5 mmol; 2.1 equiv.) at –10 °C under nitrogen atmosphere. The reaction mixture was stirred at the same temperature for 0.5 h and diluted with EtOAc (20 mL). After the reaction completion by monitoring the disappearance of starting material with TLC and LC-MS, the reaction mixture was quenched with saturated sodium potassium tartrate solution and 10 mL of diethyl ether. Then, the reaction mixture was stirred for an additional hour to ensure that both organic and aqueous layers were separated. The separated organic layer was diluted with EtOAc (10 mL), washed with water and brine, dried over anhydrous Na_2SO_4(s), filtered, and evaporated to provide the crude product. The resulting crude mixture was purified by silica-gel flash column chromatography eluting with EtOAc in hexanes to provide the desired alcohols, **8b(II)–8l(II)**.

3) Substituted benzofuran-3-carboxaldehyde (**8b–8l**): MnO_2 oxidation

A mixture of substituted benzofuran-3-yl methanol (1.0 equiv.) and manganese dioxide (MnO_2, 10.0 equiv.) in CH_2Cl_2 (0.1 M) was heated at 50 ℃ for 16 h. Then, the insoluble portion was filtered off with a short pad of Celite, and the filtrate was diluted with DCM. The combined organic layer was washed with water and brine, dried over anhydrous Na_2SO_4(s), filtered, and evaporated under reduced pressure to provide the crude product. The resulting residue was purified by silica-gel flash column chromatography (10% EtOAc in hexanes) to provide the desired benzofuran-3-carbaldehydes (**8b–8l**).

Compound 8b(I): ethyl 5-chlorobenzofuran-3-carboxylate

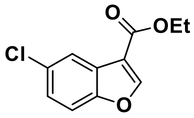
8b(I)

Following the general procedure described above with 5-chloro-2-hydroxy-benzaldehyde (0.934 g, 6.0 mmol) provided **8b(I)** in 81% yield (1.09 g) as off-white solid; ^{1}H NMR (400 MHz, $CDCl_3$): δ 8.25 (s, 1H), 8.03 (d, *J* = 2.4 Hz, 1H), 7.48–7.39 (m, 1H), 7.31 (dd, *J* = 8.8, 2.4 Hz, 1H), 4.41 (q, *J* = 7.2 Hz, 2H), 1.43 (t, *J* = 7.2 Hz, 3H); ^{13}C NMR (100 MHz, $CDCl_3$): δ 162.98, 154.04, 152.11, 130.12, 126.12, 125.76, 121.91, 114.66, 112.81, 60.91, 14.50; LRMS (ESI): *m/z* calcd for $C_{12}H_9ClO_3$ $[M+H]^+$: 225.03; Found: 225.0.

Compound 8b(II): (5-chlorobenzofuran-3-yl)methanol

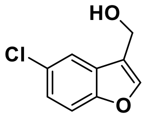
8b(II)

Following the general procedure described above with ester **8b(I)** (1.09 g, 4.85 mmol) provided **8b(II)** in 82% yield (0.726 g) as off-white solid; ^{1}H NMR (400 MHz, $CDCl_3$): δ 7.74–7.51 (m, 2H), 7.40 (d, *J* = 8.8 Hz, 1H), 7.27 (dd, *J* = 8.8, 2.4 Hz, 1H), 4.81 (s, 2H), 1.71 (s, 1H); ^{13}C NMR (100 MHz, $CDCl_3$): δ 154.14, 143.74, 128.62, 128.25, 125.06, 120.31, 119.93, 112.73, 55.88; LRMS (ESI): *m/z* calcd for $C_9H_8ClO_2$ $[M+H]^+$: 183.02; Found: not ionised.

Compound 8b: 5-chlorobenzofuran-3-carbaldehyde

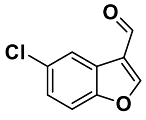
8b

Following the general procedure described above with alcohol **8b(II)** (0.5 g, 2.74 mmol) provided **8b** in 56% yield (0.276 g) as white solid; ^{1}H NMR (500 MHz, $CDCl_3$): δ 10.14 (s, 1H), 8.29 (d, *J* = 2.0 Hz, 1H), 8.17 (s, 1H), 7.47 (dd, *J* = 8.5, 2.5 Hz, 1H), 7.37 (d, *J* = 8.0 Hz, 1H); ^{13}C NMR (125 MHz, $CDCl_3$): δ 184.40, 156.15, 154.36, 130.83, 126.76, 124.39, 123.30, 122.43, 112.81; LRMS (ESI): *m/z* calcd for $C_9H_6ClO_2$ $[M+H]^+$: 181.01; Found: 181.0.

Compound 8c(I): ethyl 5-bromobenzofuran-3-carboxylate

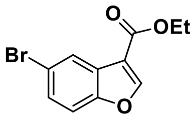
8c(I)

Following the general procedure described above with 5-bromo-2-hydroxy-benzaldehyde (1.21 g, 6.0 mmol) provided **8c(I)** in 83% yield (1.34 g) as white solid; NMR data were consistent with literature values[7].

Compound 8c(II): (5-bromobenzofuran-3-yl)methanol

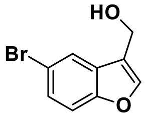

8c(II)

Following the general procedure described above with ester **8c(I)** (0.8 g, 2.87 mmol) provided **8c (II)** in 89% yield (0.6 g) as white solid; ^{1}H NMR (400 MHz, $CDCl_3$): δ 7.79 (d, *J* = 2.0 Hz, 1H), 7.57 (s, 1H), 7.43–7.31 (m, 2H), 4.77 (s, 2H), 2.01 (s, 1H); ^{13}C NMR (100 MHz, $CDCl_3$): δ 154.43, 143.53, 128.80, 127.68, 122.92, 120.09, 116.01, 113.17, 55.75; LRMS (ESI): *m/z* calcd for $C_9H_8BrO_2$ $[M+H]^+$: 226.97; Found: 227.1.

Compound 8c: 5-bromobenzofuran-3-carbaldehyde

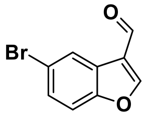

8c

Following the general procedure described above with alcohol **8c(II)** (0.5 g, 2.2 mmol) provided **8c** in 61% yield (0.3 g) as off-white solid; ^{1}H NMR (400 MHz, $CDCl_3$): δ 10.15 (s, 1H), 8.34 (d, *J* = 2.0 Hz, 1H), 8.27 (s, 1H), 7.51 (dd, *J* = 8.8, 2.0 Hz, 1H), 7.43 (d, *J* = 8.8 Hz, 1H); ^{13}C NMR (100 MHz, $CDCl_3$): δ 184.44, 156.00, 154.76, 129.51, 125.50, 124.94, 123.16, 118.34, 113.28; LRMS (ESI): *m/z* calcd for $C_9H_6BrO_2$ $[M+H]^+$: 224.96; Found: 224.0.

Compound 8d(I): ethyl 5-nitrobenzofuran-3-carboxylate

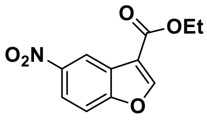

8d(I)

Following the general procedure described above with 2-hydroxy-5-nitro-benzaldehyde (1.0 g, 6.0 mmol) provided **8d(I)** in 65% yield (0.92 g) as off-white solid; NMR data were consistent with literature values[2].

Compound 8d(II): (5-nitrobenzofuran-3-yl)methanol

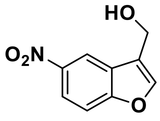

8d(II)

Following the general procedure described above with ester **8d(I)** (0.7 g, 2.98 mmol) provided **8d(II)** in 82% yield (0.474 g) as yellow solid; ^{1}H NMR (400 MHz, CD_3OD): δ 8.63 (d, *J* = 2.8 Hz, 1H), 8.23 (dd, *J* = 9.2, 2.4 Hz, 1H), 7.92 (s, 1H), 7.63 (d, *J* = 9.2 Hz, 1H), 4.89 (s, 2H); ^{13}C NMR (100 MHz, CD_3OD): δ 159.77, 146.84, 145.38, 128.88, 123.38, 121.25, 117.85, 112.92, 55.18; LRMS (ESI): *m/z* calcd for $C_9H_8NO_4$ $[M+H]^+$: 194.05; Found: 194.2.

Compound 8d: 5-nitrobenzofuran-3-carbaldehyde

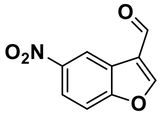
8d

Following the general procedure described above with alcohol **8d(II)** (0.4 g, 2.07 mmol) provided **8d** in 51% yield (0.2 g) as yellow solid; ^{1}H NMR (400 MHz, $CDCl_3$): δ 10.23 (s, 1H), 9.11 (d, *J* = 2.4 Hz, 1H), 8.46 (s, 1H), 8.36 (dd, *J* = 9.2, 2.4 Hz, 1H), 7.69 (d, *J* = 9.2 Hz, 1H); ^{13}C NMR (100 MHz, $CDCl_3$): δ 183.96, 158.35, 157.17, 123.90, 123.79, 122.29, 119.46, 112.49; LRMS (ESI): *m/z* calcd for $C_9H_6NO_4$ $[M+H]^+$: 192.03; Found: 192.0.

Compound 8e(I): ethyl 6-nitrobenzofuran-3-carboxylate

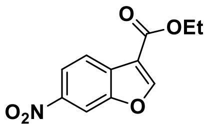
8e(I)

Following the general procedure described above with 2-hydroxy-4-nitro-benzaldehyde (1.0 g, 6.0 mmol) provided **8e(I)** in 54% yield (0.76 g) as yellow solid; ^{1}H NMR (400 MHz, $CDCl_3$): δ 8.48 (s, 1H), 8.45 (d, *J* = 2.0 Hz, 1H), 8.29 (dd, *J* = 8.8, 2.0 Hz, 1H), 8.20 (d, *J* = 8.4 Hz, 1H), 4.45 (q, *J* = 7.2 Hz, 2H), 1.45 (t, *J* = 7.2 Hz, 3H); ^{13}C NMR (100 MHz, $CDCl_3$): δ 162.38, 155.17, 154.30, 145.86, 130.54, 122.47, 119.86, 115.24, 108.40, 61.30, 14.46; LRMS (ESI): *m/z* calcd for $C_{11}H_{10}NO_5$ $[M+H]^+$: 236.06; Found: 236.1.

Compound 8e(II): (6-nitrobenzofuran-3-yl)methanol

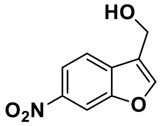
8e(II)

Following the general procedure described above with ester **8e(I)** (0.7 g, 2.98 mmol) provided **8e(II)** in 96% yield (0.55 g) as yellow solid; ^{1}H NMR (400 MHz, $CDCl_3$): δ 8.39 (d, *J* = 2.0 Hz, 1H), 8.19 (dd, *J* = 8.8, 2.0 Hz, 1H), 7.87 (s, 1H), 7.78 (d, *J* = 8.4 Hz, 1H), 4.89 (s, 2H); ^{13}C NMR (100 MHz, $CDCl_3$): δ 154.41, 147.33, 145.48, 132.75, 121.06, 120.35, 118.66, 108.23, 55.70; LRMS (ESI): *m/z* calcd for $C_9H_8NO_4$ $[M+H]^+$: 194.05; Found: 194.1.

Compound 8e: 6-nitrobenzofuran-3-carbaldehyde

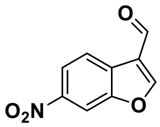
8e

Following the general procedure described above with alcohol **8e(II)** (0.51 g, 2.64 mmol) provided **8e** in 44% yield (0.22 g) as yellow solid; ^{1}H NMR (400 MHz, $CDCl_3$): δ 10.24 (s, 1H), 8.54 (s, 1H), 8.49 (s, 1H), 8.39–8.29 (m, 2H); ^{13}C NMR (100 MHz, $CDCl_3$): 184.13, 158.72, 154.69, 146.48, 128.81, 123.32, 123.09, 120.57, 108.40; LRMS (ESI): *m/z* calcd for $C_9H_6NO_4$ $[M+H]^+$: 192.03; Found: 192.1.

Compound 8f(I): ethyl 5-methylbenzofuran-3-carboxylate

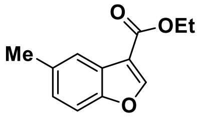

8f(I)

Following the general procedure described above with 2-hydroxy-5-methylbenzaldehyde (0.817 g, 6.0 mmol) provided **8f(I)** in 73% yield (0.9 g) as *colorless* gum; ^{1}H NMR (400 MHz, $CDCl_3$): δ 8.22 (s, 1H), 7.85 (s, 1H), 7.40 (d, *J* = 8.4 Hz, 1H), 7.17 (dd, *J* = 8.4, 1.6 Hz, 1H), 4.41 (q, *J* = 7.2 Hz, 2H), 2.48 (s, 3H), 1.43 (t, *J* = 7.2 Hz, 3H); ^{13}C NMR (100 MHz, $CDCl_3$): δ 163.74, 154.20, 151.20, 133.95, 126.64, 124.81, 121.86, 114.52, 111.28, 60.65, 21.57, 14.55; LRMS (ESI): *m/z* calcd for $C_{12}H_{13}O_3$ $[M+H]^+$: 205.09; Found: 205.1.

Compound 8f(II): (5-methylbenzofuran-3-yl)methanol

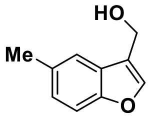

8f(II)

Following the general procedure described above with ester **8f(I)** (0.8 g, 3.92 mmol) provided **8f(II)** in 82% yield (0.52 g) as white solid; ^{1}H NMR (500 MHz, $CDCl_3$): 7.48 (s, 1H), 7.40 (s, 1H), 7.33 (d, *J* = 8.0 Hz, 1H), 7.09 (dd, *J* = 8.0, 2.0 Hz, 1H), 4.71 (s, 2H), 2.42 (s, 3H); ^{13}C NMR (125 MHz, $CDCl_3$): δ 154.08, 142.51, 132.34, 126.81, 125.94, 120.14, 119.74, 111.11, 55.81, 21.34; LRMS (ESI): *m/z* calcd for $C_{10}H_{11}O_2$ $[M+H]^+$: 163.08; Found: 163.2.

Compound 8f: 5-methylbenzofuran-3-carbaldehyde

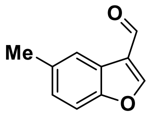

8f

Following the general procedure described above with alcohol **8f(II)** (0.42 g, 2.59 mmol) provided **8f** in 55% yield (0.16 g) as off-white solid; ^{1}H NMR (400 MHz, $CDCl_3$): δ 10.15 (s, 1H), 8.23 (s, 1H), 7.98 (s, 1H), 7.42 (d, *J* = 8.4 Hz, 1H), 7.21(dd, *J* = 8.4. 2.0 Hz, 1H), 2.47 (s, 3H); ^{13}C NMR (100 MHz, $CDCl_3$): δ 185.05, 155.72, 154.59, 134.83, 127.63, 123.65, 123.06, 122.44, 111.25, 21.45; LRMS (ESI): *m/z* calcd for $C_{10}H_9O_2$ $[M+H]^+$: 161.06; Found: 161.1.

Compound 8g(I): ethyl 6-methylbenzofuran-3-carboxylate

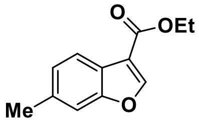

8g(I)

Following the general procedure described above with 2-hydroxy-4-methylbenzaldehyde (0.82 g, 6.0 mmol) provided **8g(I)** in 41% yield (0.5 g) as off-white solid; ^{1}H NMR (400 MHz, $CDCl_3$): δ 8.18 (s, 1H), 7.91 (d, *J* = 8.0 Hz, 1H), 7.32 (s, 1H), 7.17 (d, *J* = 8.0 Hz, 1H), 4.39 (q, *J* = 7.2 Hz, 2H), 2.48 (s, 3H), 1.42 (t, *J* = 7.2 Hz, 3H); ^{13}C

NMR (100 MHz, $CDCl_3$): δ 163.69, 156.13, 150.51, 135.73, 125.69, 122.21, 121.54, 114.73, 111.90, 60.59, 21.76, 14.50; LRMS (ESI): *m/z* calcd for $C_{12}H_{13}O_3$ $[M+H]^+$: 205.06; Found: 205.2.

Compound 8g(II): (6-methylbenzofuran-3-yl)methanol

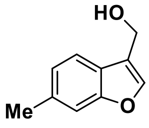
8g(II)

Following the general procedure described above with ester **8g(I)** (0.8 g, 3.92 mmol) provided **8g(II)** in 89% yield (0.57 g) as yellow solid; ^1H NMR (400 MHz, $CDCl_3$): δ 7.56–7.40 (m, 2H), 7.27 (s, 1H), 7.07 (d, *J* = 8.0 Hz, 1H), 4.75 (s, 2H), 2.46 (s, 3H), 1.99 (s, 1H); ^{13}C NMR (100 MHz, $CDCl_3$): δ 156.13, 141.85, 135.01, 124.28, 124.24, 120.34, 119.45, 111.86, 56.00, 21.75; LRMS (ESI): *m/z* calcd for $C_{10}H_{11}O_2$ $[M+H]^+$: 163.08; Found: 163.2.

Compound 8g: 6-methylbenzofuran-3-carbaldehyde

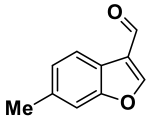
8g

Following the general procedure described above with alcohol **8g(II)** (0.52 g, 3.22 mmol) provided **8g** in 67% yield (0.35 g) as orange solid; ^1H NMR (400 MHz, $CDCl_3$): δ 10.14 (s, 1H), 8.20 (s, 1H), 8.04 (d, *J* = 8.0 Hz, 1H), 7.35 (s, 1H), 7.21 (d, *J* = 8.0 Hz, 1H), 2.49 (s, 3H); ^{13}C NMR (100 MHz, $CDCl_3$): 184.95, 156.56, 155.11, 136.92, 126.39, 123.84, 122.08, 120.45, 111.89, 21.86; LRMS (ESI): *m/z* calcd for $C_{10}H_9O_2$ $[M+H]^+$: 161.06; Found: 161.1.

Compound 8h(I): ethyl 5-methoxybenzofuran-3-carboxylate

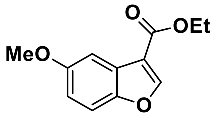
8h(I)

Following the general procedure described above with 2-hydroxy-5-methoxy-benzaldehyde (0.91 g, 6.0 mmol) provided **8h(I)** in 41% yield (0.54 g) as off- white solid; ^1H NMR (400 MHz, $CDCl_3$): δ 8.21 (s, 1H), 7.52 (d, *J* = 2.8 Hz, 1H), 7.40 (d, *J* = 9.2 Hz, 1H), 6.95 (dd, *J* = 9.2, 2.8 Hz, 1H), 4.40 (q, *J* = 7.2 Hz, 2H), 3.88 (s, 3H), 1.42 (t, *J* = 7.2 Hz, 3H); ^{13}C NMR (100 MHz, $CDCl_3$): δ 163.63, 157.05, 151.59, 150.60, 125.50, 114.71, 114.59, 112.31, 103.82, 60.60, 55.96, 14.49; LRMS (ESI): *m/z* calcd for $C_{12}H_{13}O_4$ $[M+H]^+$: 221.08; Found: 221.1.

Compound 8h(II): (5-methoxybenzofuran-3-yl)methanol

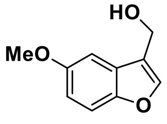
8h(II)

Following the general procedure described above with ester **8h(I)** (0.5 g, 2.27 mmol) provided **8h(II)** in 91% yield (0.37 g) as colorless liquid; ^{1}H NMR (400 MHz, $CDCl_3$): δ 7.56 (s, 1H), 7.36 (d, *J* = 8.8 Hz, 1H), 7.10 (d, *J* = 2.8 Hz, 1H), 6.91 (dd, *J* = 8.8, 2.8 Hz, 1H), 4.82–4.76 (m, 2H), 3.84 (s, 3H), 1.86 (s, 1H); ^{13}C NMR (100 MHz, $CDCl_3$): δ 156.08, 150.66, 143.29, 127.33, 120.56, 113.72, 112.20, 102.25, 56.06, 56.00; LRMS (ESI): *m/z* calcd for $C_{10}H_{11}O_3$ $[M+H]^+$: 179.07; Found: not ionised.

Compound 8h: 5-methoxybenzofuran-3-carbaldehyde

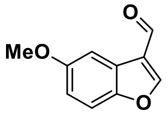
8h

Following the general procedure described above with alcohol **8h(II)** (0.35 g, 1.96 mmol) provided **8h** in 61% yield (0.21 g) as off-white solid; ^{1}H NMR (400 MHz, $CDCl_3$): δ 10.14 (s, 1H), 8.24 (s, 1H), 7.63 (d, *J* = 2.8 Hz, 1H), 7.43 (d, *J* = 9.2 Hz, 1H), 6.99 (dd, *J* = 9.2, 2.8 Hz, 1H), 3.88 (s, 3H); ^{13}C NMR (100 MHz, $CDCl_3$): δ 185.04, 157.59, 156.14, 150.94, 123.92, 123.66, 115.87, 112.38, 104.06, 56.05; LRMS (ESI): *m/z* calcd for $C_{10}H_9O_3$ $[M+H]^+$: 177.06; Found: 177.1.

Compound 8i(I): ethyl 6-methoxybenzofuran-3-carboxylate

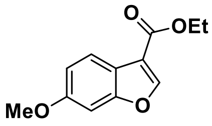
8i(I)

Following the general procedure described above with 2-hydroxy-4-methyl-benzaldehyde (0.82 g, 6.0 mmol) provided **8i(I)** in 41% yield (0.5 g) as white solid; NMR data were consistent with literature values [8].

Compound 8i(II): (6-methoxybenzofuran-3-yl)methanol

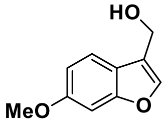
8i(II)

Following the general procedure described above with ester **8i(I)** (0.6 g, 2.72 mmol) provided **8i(II)** in 78% yield (0.38 g) as off-white solid; ^{1}H NMR (400 MHz, $CDCl_3$): δ 7.57–7.48 (m, 2H), 7.01 (d, *J* = 2.4 Hz, 1H), 6.90 (dd, *J* = 8.4, 2.4 Hz, 1H), 4.79 (s, 2H), 3.85 (s, 3H), 1.75 (s, 1H); ^{13}C NMR (100 MHz, $CDCl_3$): δ 158.40, 156.77, 141.52, 120.42, 120.17, 120.13, 111.99, 96.22, 56.13, 55.85; LRMS (ESI): *m/z* calcd for $C_{10}H_{11}O_3$ $[M+H]^+$: 179.07; Found: 179.2.

Compound 8i: 6-methoxybenzofuran-3-carbaldehyde

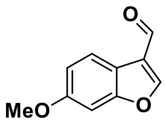

8i

Following the general procedure described above with alcohol **8i(II)** (0.35 g, 1.97 mmol) provided **8i** in 46% yield (0.16 g) as red solid; ^{1}H NMR (400 MHz, $CDCl_3$): δ 10.11 (s, 1H), 8.17 (s, 1H), 8.03 (d, *J* = 8.8 Hz, 1H), 7.05 (d, *J* = 2.4 Hz, 1H), 7.00 (dd, *J* = 8.8, 2.4 Hz, 1H), 3.87 (s, 3H); ^{13}C NMR (100 MHz, $CDCl_3$): 184.91, 159.33, 157.23, 154.74, 123.89, 122.79, 116.14, 113.85, 96.17, 55.86; LRMS (ESI): *m/z* calcd for $C_{10}H_9O_3$ $[M+H]^+$: 177.06; Found: 177.1.

Compound 8j(I): ethyl naphtho[2,1-*b*]furan-1-carboxylate

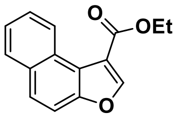

8j(I)

Following the general procedure described above with 2-hydroxy-5-methoxy-benzaldehyde (1.03 g, 6.0 mmol) provided **8j(I)** in 68% yield (0.96 g) as a white crystalline solid; ^{1}H NMR (500 MHz, $CDCl_3$): δ 9.48 (d, *J* = 8.5 Hz, 1H), 8.37 (s, 1H), 7.92 (d, *J* = 8.0 Hz, 1H), 7.78 (d, *J* = 9.0 Hz, 1H), 7.67–7.60 (m, 2H), 7.52 (t, *J* = 7.5 Hz, 1H), 4.44 (q, *J* = 7.0 Hz, 2H), 1.44 (t, *J* = 7.0 Hz, 3H); ^{13}C NMR (125 MHz, $CDCl_3$): δ 163.82, 154.07, 151.26, 131.38, 128.70, 128.26, 127.48, 126.90, 126.81, 125.28, 119.44, 116.89, 112.28, 60.95, 14.51 ; LRMS (ESI): *m/z* calcd for $C_{15}H_{13}O_3$ $[M+H]^+$: 241.09; Found: 241.1.

Compound 8j(II): naphtho[2,1-*b*]furan-1-ylmethanol

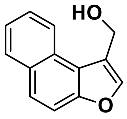

8j(II)

Following the general procedure described above with ester **8j(I)** (0.86 g, 3.64 mmol) provided **8j(II)** in 70% yield (0.51 g) as a red solid; ^{1}H NMR (400 MHz, $CDCl_3$): 8.33 (d, *J* = 8.4 Hz, 1H), 7.92 (d, *J* = 8.0 Hz, 1H), 7.70 (d, *J* = 8.8 Hz, 1H), 7.65 (s, 1H), 7.62–7.54 (m, 2H), 7.47 (t, *J* = 7.6 Hz, 1H), 5.00 (d, *J* = 4.4 Hz, 2H), 2.03 (t, *J* = 4.8 Hz, 1H); ^{13}C NMR (100 MHz, $CDCl_3$): δ 153.88, 142.30, 130.73, 128.96, 128.20, 126.77, 126.09, 124.57, 124.27, 122.05, 120.75, 112.69, 56.92; LRMS (ESI): *m/z* calcd for $C_{13}H_{11}O_2$ $[M+H]^+$: 199.08; Found: 199.1.

Compound 8j: naphtho[2,1-*b*]furan-1-carbaldehyde

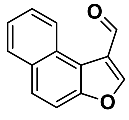

8j

Following the general procedure described above with alcohol **8j(II)** (0.35 g, 1.96 mmol) provided **8j** in 61% yield (0.21 g) as pale brown solid; ^{1}H NMR (400 MHz, $CDCl_3$): δ 10.16 (s, 1H), 9.36 (d, *J* = 8.4 Hz, 1H), 8.36 (s, 1H), 7.93 (d, *J* = 8.0 Hz, 1H), 7.82 (d, *J* = 8.8 Hz, 1H), 7.72–7.60 (m, 2H), 7.55 (t, *J* = 7.6 Hz, 1H); ^{13}C NMR (100 MHz, $CDCl_3$): δ 184.05, 157.53, 154.85, 131.41, 128.60, 128.32, 128.27, 127.66, 127.16, 126.82, 125.73, 118.49, 112.09 ; LRMS (ESI): *m/z* calcd for $C_{13}H_9O_2$ $[M+H]^+$: 197.06; Found: 197.1.

Compound 8k(I): ethyl [1,3]dioxolo[4,5-*f*]benzofuran-7-carboxylate

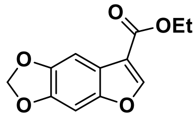

8k(I)

Following the general procedure described above with 6-hydroxy-1,3-benzodioxole-5-carbaldehyde[9] (0.996 g, 6.0 mmol) provided **8k(I)** in 14% yield (0.202 g) as white solid; ^{1}H NMR (400 MHz, $CDCl_3$): δ 8.14 (s, 1H), 7.41 (s, 1H), 6.98 (s, 1H), 6.02 (s, 2H), 4.38 (q, *J* = 7.2 Hz, 2H), 1.41 (t, *J* = 7.2 Hz, 3H); ^{13}C NMR (100 MHz, $CDCl_3$): δ 163.59, 150.96, 150.18, 146.97, 145.78, 118.36, 115.15, 101.75, 100.49, 93.70, 60.63, 14.50; LRMS (ESI): *m/z* calcd for $C_{12}H_{11}O_5$ $[M+H]^+$: 235.06; Found: 235.1.

Compound 8k(II): [1,3]dioxolo[4,5-*f*]benzofuran-7-ylmethanol

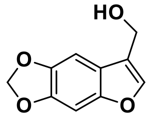

8k(II)

Following the general procedure described above with ester **8k(I)** (0.18 g, 0.77 mmol) provided **8k(II)** in 81% yield (0.12 g) as off-white solid; ^{1}H NMR (400 MHz, $CDCl_3$): δ 7.50 (s, 1H), 7.01 (s, 1H), 6.96 (s, 1H), 5.98 (s, 2H), 4.74 (s, 2H), 1.79 (s, 1H); ^{13}C NMR (100 MHz, $CDCl_3$): δ 150.92, 146.56, 144.72, 141.91, 120.85, 120.07, 101.49, 98.54, 93.83, 56.02; LRMS (ESI): *m/z* calcd for $C_{10}H_9O_4$ $[M+H]^+$: 193.05; Found: 193.1.

Compound 8k: [1,3]dioxolo[4,5-*f*]benzofuran-7-carbaldehyde

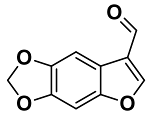

8k

Following the general procedure described above with alcohol **8k(II)** (0.105 g, 0.55 mmol) provided **8k** in 48% yield (0.05 g) as off-white solid; ^{1}H NMR (400 MHz, $CDCl_3$): δ 10.09 (s, 1H), 8.16 (s, 1H), 7.54 (s, 1H), 7.01 (s, 1H), 6.03 (s, 2H); ^{13}C NMR (100 MHz, $CDCl_3$):

184.85, 154.61, 151.50, 147.72, 146.29, 124.23, 116.47, 101.95, 100.95, 93.72; LRMS (ESI): *m/z* calcd for $C_{10}H_7O_4$ [M+H]$^+$: 191.03; Found: 191.1.

Compound 8l(I): ethyl 2,3,6,7-tetrahydro-1*H*,5*H*-furo[2,3-*f*]pyrido[3,2,1-*ij*]quinoline-9-carboxylate

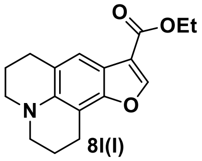
8l(I)

Following the general procedure described above with 6-hydroxy-1,3-benzodioxole-5-carbaldehyde (1.3 g, 6.0 mmol) provided **8l(I)** in 47% yield (0.80 g) as off-white solid; ^{1}H NMR (400 MHz, $CDCl_3$): δ 8.01 (s, 1H), 7.41 (s, 1H), 4.36 (q, *J* = 7.2 Hz, 2H), 3.16 (q, *J* = 6.0 Hz, 4H), 2.91 (dt, *J* = 18.4, 6.4 Hz, 4H), 2.08–1.95 (m, 4H), 1.40 (t, *J* = 7.2 Hz, 3H); ^{13}C NMR (100 MHz, $CDCl_3$): δ 164.27, 154.04, 148.39, 141.42, 120.25, 118.55, 114.62, 113.60, 104.52, 60.34, 50.47, 50.13, 28.37, 22.43, 21.39, 20.79, 14.55; LRMS (ESI): *m/z* calcd for $C_{17}H_{20}NO_3$ [M+H]$^+$: 286.14; Found: 286.1.

Compound 8l(II): (2,3,6,7-tetrahydro-1*H*,5*H*-furo[2,3-*f*]pyrido[3,2,1-*ij*]quinolin-9-yl)methanol

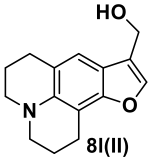
8l(II)

Following the general procedure described above with ester **8l(I)** (0.18 g, 0.77 mmol) provided **8l(II)** in 81% yield (0.12 g) as brown solid; ^{1}H NMR (400 MHz, $CDCl_3$): δ 7.36 (s, 1H), 7.06 (s, 1H), 4.71 (d, *J* = 5.2 Hz, 2H), 3.14 (q, *J* = 5.6 Hz, 4H), 2.93 (t, *J* = 6.8 Hz, 2H), 2.86 (t, *J* = 6.8 Hz, 2H), 2.07–1.95 (m, 4H), 1.59 (t, *J* = 5.2 Hz, 1H); ^{13}C NMR (100 MHz, $CDCl_3$): δ 153.93, 141.14, 139.82, 120.42, 118.98, 116.67, 115.93, 105.01, 56.34, 50.59, 50.28, 28.28, 22.57, 21.55, 20.89; LRMS (ESI): *m/z* calcd for $C_{15}H_{18}NO_2$ [M+H]$^+$: 244.13; Found: 244.1.

Compound 8l: 2,3,6,7-tetrahydro-1*H*,5*H*-furo[2,3-*f*]pyrido[3,2,1-*ij*]quinoline-9-carbaldehyde

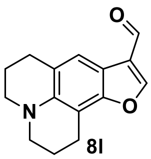
8l

Following the general procedure described above with alcohol **8l(II)** (0.22 g, 0.904 mmol) provided **8l** in 23% yield (0.05 g) as brown solid; ^{1}H NMR (400 MHz, $CDCl_3$): δ 10.04 (s, 1H), 8.01 (s, 1H), 7.53 (s, 1H), 3.20–3.14 (m, 4H), 2.93 (t, *J* = 6.8 Hz, 2H), 2.87 (t, *J* = 6.8 Hz, 2H), 2.07–1.94 (m, 4H); ^{13}C NMR (100 MHz, $CDCl_3$): 185.33, 154.59, 153.58, 142.12, 124.29, 120.73, 119.11, 111.36, 104.26, 50.37, 50.01, 28.26, 22.25, 21.24, 20.78; LRMS (ESI): *m/z* calcd for $C_{15}H_{16}NO_2$ [M+H]$^+$: 242.12; Found: 242.1.

Supplementary Figure 15. Synthesis of starting material benzofuran-3-carboxaldehydes **8m:**

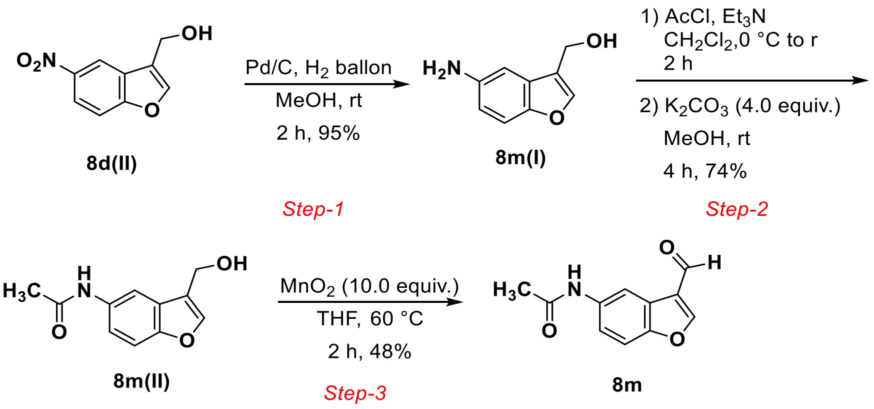

Step-1: To a stirred solution of (5-nitrobenzofuran-3-yl)methanol **8d(II)** (140 mg, 0.724 mmol) in methanol (7 mL) was added palladium 10% on activated carbon (23.14 mg, 0.22 mmol). The reaction mixture was purged with hydrogen and reacted in the presence of a hydrogen balloon at r.t. for 1 h. After completion of the reaction monitored by TLC, the reaction mixture was filtered, and the filter cake was washed with EtOAc. The filtrate was concentrated to dryness to obtain the crude product. The crude product was purified by silica-gel flash column chromatography using EtOAc and hexanes as eluent to provide **8m(I)** in 94% yield (0.112 g) as white solid.

Compound 8m(I): (5-aminobenzofuran-3-yl)methanol

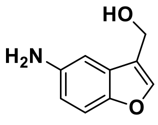

8m(I)

^{1}H NMR (400 MHz, DMSO-d_6): δ 7.64 (s, 1H), 7.18 (d, J = 8.8 Hz, 1H), 6.77 (d, J = 2.0 Hz, 1H), 6.58 (dd, J = 8.8, 2.0 Hz, 1H), 4.99 (t, J = 5.2 Hz, 1H), 4.82 (s, 2H), 4.52 (d, J = 5.2 Hz, 2H); ^{13}C NMR (100 MHz, DMSO-d_6): δ 148.14, 144.23, 142.19, 127.58, 120.84, 112.68, 110.99, 103.24, 54.05; LRMS (ESI): m/z calcd for $C_9H_{10}NO_2$ $[M+H]^+$: 164.07; Found: 164.1.

Step-2: To a pre-cooled stirred solution of (5-aminobenzofuran-3-yl)methanol **8m(I)** (94 mg, 0.58 mmol) in DCM (6 mL) at 0 °C, DIPEA (297.8 mg, 2.30 mmol, 401.4 μL) and acetyl chloride (94.9 mg, 1.21 mmol, 86.3 μL) were added at the same temperature. Then, the reaction solution was warmed to r.t. and stirred for 0.5 h, and the volatiles was removed *in vacuo*. The obtained crude residue was diluted with methanol, added with K_2CO_3 (318.5 mg, 2.30 mmol), and stirred for additional 4 h. After completion of the reaction

(monitored by TLC and LC-MS), the reaction mixture was concentrated under reduced pressure. The resulting residue was diluted with water and extracted with EtOAc (2 × 10 mL). The combined organic layer was washed with brine, dried over anhydrous Na_2SO_2(s), filtered, and evaporated to give a crude product. The crude product was purified by silica-gel flash column chromatography using DCM and MeOH as eluent to furnish **8m(II)** in 74% yield (88 mg) as a white solid.

Compound 8m(II): *N*-(3-(hydroxymethyl)benzofuran-5-yl)acetamide

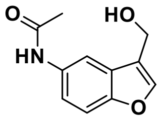

8m(II)

^{1}H NMR (400 MHz, DMSO-d_6): δ 9.96 (s, 1H), 7.99 (d, J = 2.0 Hz, 1H), 7.83 (s, 1H), 7.47 (d, J = 8.8 Hz, 1H), 7.40 (dd, J = 8.8, 2.0 Hz, 1H), 5.13 (t, J = 5.2 Hz, 1H), 4.59 (d, J = 5.2 Hz, 2H), 2.05 (s, 3H); ^{13}C NMR (100 MHz, DMSO-d_6): δ 167.99, 151.13, 143.11, 134.53, 127.00, 121.52, 116.84, 111.07, 110.55, 53.87, 23.95; LRMS (ESI): *m/z* calcd for $C_{11}H_{12}NO_3$ $[M+H]^+$: 206.08; Found: 206.1.

Step-3: To a stirred solution of *N*-[3-(hydroxymethyl)benzofuran-5-yl]acetamide **8m(II)** (78 mg, 0.38 mmol) in THF (4 mL), manganese dioxide (338.1 mg, 3.80 mmol) was added. Then, the reaction mixture was heated at 60 °C for 1 h. After the disappearance of starting material monitored by TLC and LC-MS, the reaction mixture was filtered with a short pad of Celite® and washed with EtOAc. The combined organic layer was concentrated under reduced pressure to provide give a crude product which was purified by silica-gel flash column chromatography (hexanes and EtOAc as eluent) to furnish *N*-(3-formylbenzofuran-5-yl)acetamide **8m** (44 mg, 216.5 µmol, 57% yield) as a white solid.

Compound 8m: *N*-(3-formylbenzofuran-5-yl)acetamide

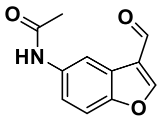

8m

^{1}H NMR (400 MHz, DMSO-d_6): δ 10.12 (s, 1H), 10.10 (s, 1H), 8.95 (s, 1H), 8.39 (d, J = 2.0 Hz, 1H), 7.70–7.56 (m, 2H), 2.07 (s, 3H); ^{13}C NMR (100 MHz, DMSO-d_6): 186.27, 168.22, 158.83, 151.31, 136.63, 122.86, 122.65, 118.13, 111.82, 111.57, 24.00; LRMS (ESI): *m/z* calcd for $C_{11}H_{10}NO_3$ $[M+H]^+$: 204.07; Found: 204.1.

Supplementary Figure 16. Commercially available *β*-ketoesters used in this methodology.

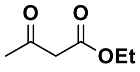

ethyl 3-oxobutanoate

2a

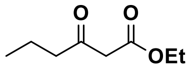

ethyl 3-oxohexanoate

2b

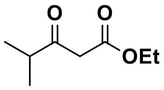

ethyl 4-methyl-3-oxopentanoate

2c

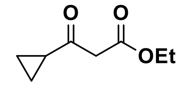

ethyl 3-cyclopropyl-3-oxopropanoate

2d

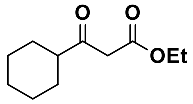

ethyl 3-cyclohexyl-3-oxopropanoate

2e

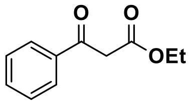

ethyl 3-oxo-3-phenylpropanoate

2f

Supplementary Figure 17. Synthesis of *β*-keto ester (**2g**)

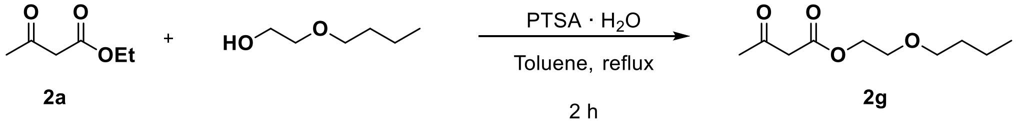

To a stirred solution of ethyl 3-oxobutanoate **2a** (260.28 mg, 2 mmol, 0.26 mL) and 2-butoxyethanol (259.98 mg, 2.20 mmol, 0.29 mL) in toluene, was added a catalytic amount of *p*-toluenesulfonic acid monohydrate (PTSA·H_2O; 38.04 mg, 0.2 mmol), and the reaction mixture was heated at 140 ℃ in a 4 mL sealed vial for 2 h. After completion of the reaction monitored by TLC, saturated aqueous $NaHCO_3$ solution was added and stirring was continued for 10 min. The organic solvents were evaporated, and the aqueous remaining was diluted with water and extracted three times with diethyl ether. The combined ethereal extracts were washed with brine, dried over anhydrous Na_2SO_4(s), filtered, and evaporated. The crude product was purified by silica-gel flash column chromatography using EtOAc/hexanes as an eluent to furnish **2g** in 42% yield (168 mg) as a colorless liquid.

Compound 2g: 2-butoxyethyl 3-oxobutanoate

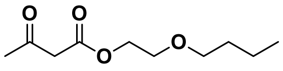

2g

^{1}H NMR (400 MHz, $CDCl_3$): δ 4.32–4.26 (m, 2H), 3.67–3.60 (m, 2H), 3.52–3.43 (m, 4H), 2.28 (s, 3H), 1.56 (p, *J* = 7.2 Hz, 2H), 1.36 (sextet, *J* = 7.2 Hz, 2H), 0.92 (t, *J* = 7.2 Hz, 3H); ^{13}C NMR (100 MHz, $CDCl_3$): 200.58, 167.27, 71.27, 68.41, 64.57, 50.13, 31.75, 30.21, 19.34, 14.00; LRMS (ESI): *m/z* calcd for $C_{10}H_{19}O_4$ $[M+H]^+$: 203.13; Found: 203.2.

Supplementary Figure 18. Synthesis of *β*-keto ester (**2h**)

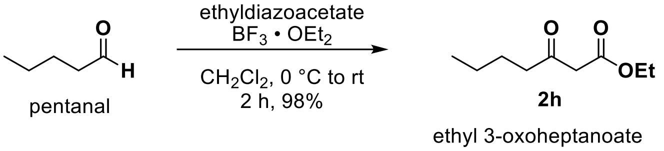

To a pre-cooled solution of ethyldiazoacetate (125.5 mg, 1.10 mmol, 116.2 μL) in DCM (10 mL) at 0 °C, was added anhydrous $BF_3·OEt_2$ (14.2 mg, 0.1 mmol, 12.3 μL) dropwise. To this suspension, a few drops of pentanal (86.1 mg, 1 mmol, 0.1 mL) in 2 mL of DCM was added. When nitrogen evolution began, the remaining solution of aldehyde was added dropwise over 10 min. After nitrogen evolution had stopped (~1 h), the reaction was transferred to a separatory funnel with brine (10 mL) and extracted with diethyl ether (2 × 20 mL). The organic layers were combined and dried over anhydrous $MgSO_4$(s), and the volatiles were removed under reduced pressure. The crude product was purified by silica-gel column chromatography (hexanes/EtOAc) to furnish the desired ketoester **2h** in 98% (170 mg) as a colorless oil.

Compound 2h: ethyl 3-oxoheptanoate

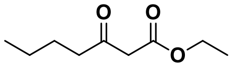

2h

The NMR of the keto ester **2h** was in good agreement with the reported values in the literature[10].

Supplementary Figure 19. Synthesis of *β*-ketoester (**2i**)

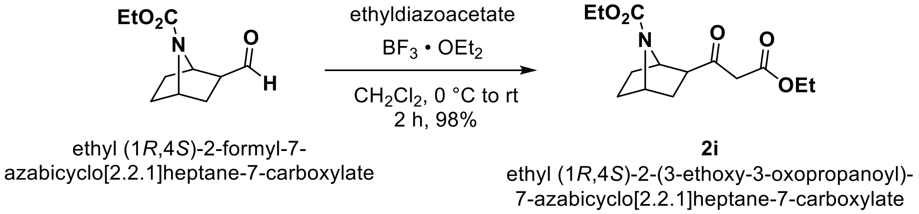

To a pre-cooled solution of ethyldiazoacetate (125.51 mg, 1.10 mmol, 116.21 µL) in DCM (10 mL) at 0 °C, was added anhydrous $BF_3{\cdot}OEt_2$ (14.19 mg, 0.1 mmol, 12.34 µL) dropwise. To this suspension, a few drops of aldehyde[11] (86.13 mg, 1 mmol, 0.1 mL) in 2 mL of DCM was added. When nitrogen evolution began, the remaining solution of aldehyde was added dropwise over 10 min. After nitrogen evolution had stopped (~1 h), the reaction was transferred to a separatory funnel with brine (10 mL) and extracted with diethyl ether (2 × 20 mL). The organic layers were combined and dried over anhydrous $MgSO_4$(s), and the volatiles were removed under reduced pressure. The crude product was purified by silica-gel flash column chromatography (hexanes/EtOAc) to furnish the desired ketoester **2i** in 98% (170 mg) as a colorless oil.

Compound 2i: ethyl (1*R*,4*S*)-2-(3-ethoxy-3-oxopropanoyl)-7-azabicyclo[2.2.1]heptane-7-carboxylate

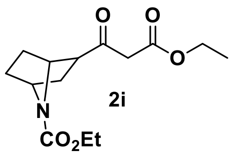

2i

^{1}H NMR (500 MHz, $CDCl_3$): δ 4.59–4.49 (m, 1H), 4.40–4.32 (m, 1H), 4.20 (q, J = 7.0 Hz, 2H), 4.11–4.03 (m, 2H), 3.68–3.49 (m, 2H), 2.82 (dd, J = 8.5, 5.0 Hz, 1H), 2.34–2.25 (m, 1H), 1.92–1.84 (m, 1H), 1.83–1.75 (m, 1H), 1.57–1.50 (m, 2H), 1.48–1.42 (m, 1H), 1.28 (t, J = 7.0 Hz, 3H), 1.23 (t, J = 7.0 Hz, 3H); ^{13}C NMR (125 MHz, $CDCl_3$): 201.28, 167.42, 155.56, 61.57, 61.39, 58.25, 56.13, 55.24, 47.86, 32.04, 29.79, 29.16, 14.59, 14.24; LRMS (ESI): *m/z* calcd for $C_{14}H_{22}NO_5$ $[M+H]^+$: 284.15; Found: 284.1.

Supplementary Figure 20. Commercially available *β*-keto sulfones/phosphonates.

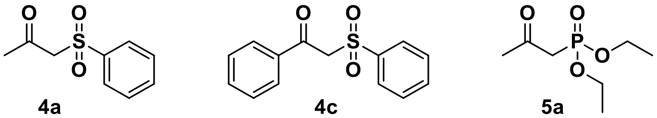

Compound 4b: 3-methyl-1-(phenylsulfonyl)butan-2-one

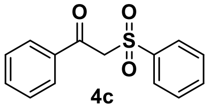

Compound **4b** was prepared from commercially available methylsulfonylbenzene using the procedure reported in the literature[12].

Supplementary Figure 21. Synthesis of *β*-keto sulfone (**4d**)

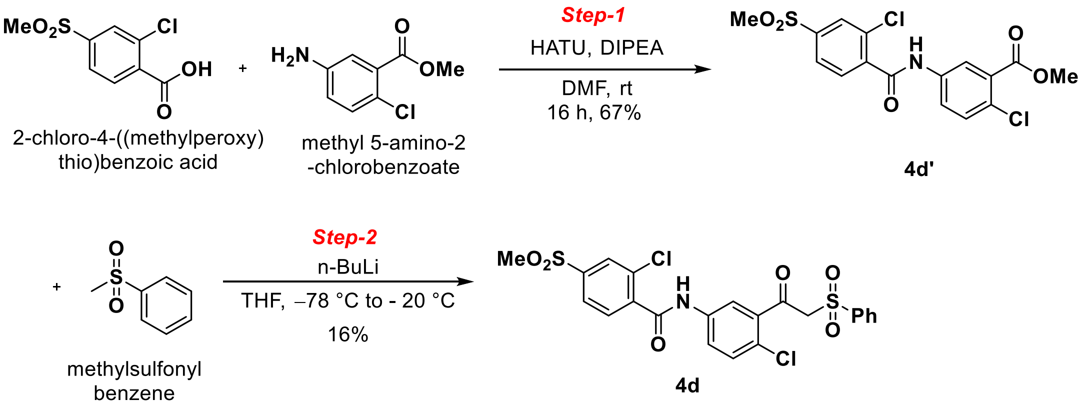

***Step-1*: Synthesis of amide 4d′**: Methyl 5-amino-2-chlorobenzoate (0.74 g, 4.00 mmol) and 2-chloro-4-methylsulfonylbenzoic acid (0.94 g, 4.00 mmol) were dissolved in DMF (16 mL) and stirred at r.t. for 16 h under argon atmosphere with an excess of 1-[bis(dimethylamino)methylene]-1*H*-1,2,3-triazolo[4,5-*b*]pyridinium 3-oxidehexafluorophosphate (HATU, 1.52 g, 4.00 mmol) and DIPEA (2.58 g, 20.00 mmol, 3.48 mL). After completion of the reaction monitored by TLC, the reaction mixture was diluted with water and extracted with EtOAc (2 × 20 mL). The combined organic layers were washed with brine (10 mL), dried over anhydrous Na_2SO_4(s), filtered, and evaporated to give a crude product. The crude product was purified by silica-gel flash column chromatography (hexanes/EtOAc) to furnish the desired amide.

Compound 4d': methyl 2-chloro-5-(2-chloro-4-(methylsulfonyl)benzamido)benzoate

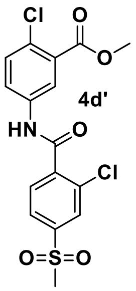

Compound **4d'** was prepared using the above procedure (4.0 mmol, 740 mg) in 67% yield (1.08 g) as a white solid. ^{1}H NMR (400 MHz, DMSO-d_6): δ 11.02 (s, 1H), 8.28 (d, *J* = 2.4 Hz, 1H), 8.15 (d, *J* = 1.6 Hz, 1H), 8.02 (dd, *J* = 8.0, 1.6 Hz, 1H), 7.93 (d, *J* = 8.0 Hz, 1H), 7.82 (dd, *J* = 8.8, 2.8 Hz, 1H), 7.60 (d, *J* = 8.8 Hz, 1H), 3.89 (s, 3H), 3.36 (s, 3H); ^{13}C NMR (100 MHz, DMSO-d_6): 165.14, 164.00, 143.25, 140.63, 137.54, 131.48, 130.98, 130.11, 130.00, 128.15, 126.52, 125.96, 123.88, 121.61, 52.70, 43.08; LRMS (ESI): *m/z* calcd for $C_{16}H_{14}Cl_2NO_5$ $[M+H]^+$: 402.00; Found: 402.0.

***Step-2*: Synthesis of amide 4d**: To a mixture of methylsulfonylbenzene (51.55 mg, 330.00 µmol) and THF (6.0 mL) cooled to –78 °C was added *n*-BuLi (2.5 M, 0.3 mL). After stirring for 1 h, methyl 2-chloro-5-[(2-chloro-4-methylsulfonyl-benzoyl)amino]benzoate (**4d′**, 120.7 mg, 0.3 mmol) was added to this solution at –78 °C. Stirring was continued for 1 h and then slowly warmed to –20 °C, and stirred for another 1 h. After completion of the reaction monitored by TLC, the reaction mixture was poured into a saturated aqueous NH_4Cl solution (10 mL) and extracted with EtOAc (2 × 20 mL). The combined organic layer was washed with brine, dried over anhydrous Na_2SO_4(s), filtered, and concentrated *in vacuo*. The residue was purified by silica-gel flash column chromatography (hexanes/EtOAc) to furnish the desired *β*-keto sulfone.

Compound 4d: 2-chloro-N-(4-chloro-3-(2-(phenylsulfonyl)acetyl)phenyl)-4-(methylsulfonyl)benzamide

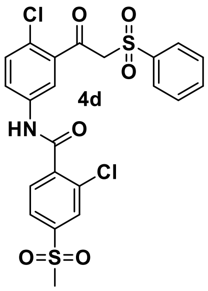

Compound **4d'** was prepared using the above procedure (0.33 mmol, 51.55 mg) in 16% yield (25 mg) as a white solid. ^{1}H NMR (400 MHz, $CDCl_3$): δ 8.91 (s, 1H), 8.05 (dd, *J* = 8.4, 2.4 Hz, 1H), 7.89–7.82 (m, 3H), 7.78–7.74 (m, 3H), 7.67 (t, *J* = 7.6 Hz, 1H), 7.56 (t, *J* = 7.6 Hz, 2H), 7.38 (d, *J* = 8.8 Hz, 1H), 4.83 (s, 2H), 3.09 (s, 3H); ^{13}C NMR (100 MHz, $CDCl_3$): 189.56, 163.78, 142.88, 140.41, 138.67, 137.38, 137.04, 134.59, 132.65, 131.64, 130.65, 129.54, 129.13, 128.53, 127.04, 125.97, 124.79, 121.81, 66.27, 44.58; LRMS (ESI): *m/z* calcd for $C_{22}H_{18}Cl_2NO_6S_2$ $[M+H]^+$: 526.00; Found: 526.0.

Supplementary Figure 22. Synthesis of β-keto phosphonate(**5b**)

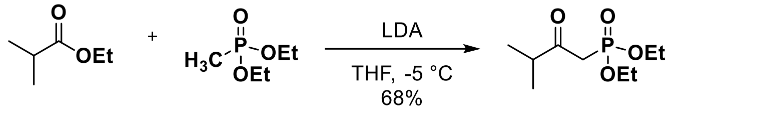

A two-necked, round-bottomed flask equipped with a nitrogen inlet adapter and magnetic bar was charged with the 1-[ethoxy(methyl)phosphoryl]oxyethane (1.83 g, 12 mmol, 1.75 mL) in THF. The reaction mixture was cooled at –5 °C, while LDA (2 M in THF/heptane/ethylbenzene, 6.50 mL) was added dropwise keeping the internal temperature below 0 ℃. After the addition, the reaction mixture was stirred at –5 ℃ for 30 min, then ethyl 2-methylpropanoate (1.16 g, 10.00 mmol, 1.34 mL) was added and stirred for 2 h at the same temperature. The reaction mixture was then carefully quenched with 6 M HCl to adjust the p*H* to 4–5 and diluted with EtOAc. The aqueous layer was separated and extracted twice with EtOAc. The combined organic layers were washed with water and brine, dried over anhydrous Na_2SO_4(s), filtered, and evaporated *in vacuo*. The residue was purified by silica-gel flash column chromatography with EtOAc/hexanes to give the desired β-keto phosphonate.

Compound 5b: diethyl (3-methyl-2-oxobutyl)phosphonate

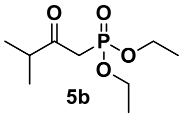
5b

The NMR of the **5b** was in good agreement with the reported values in the literature [13]. ^{1}H NMR (500 MHz, $CDCl_3$): δ 4.21–4.09 (m, 4H), 3.13 (dd, *J* = 22.5, 2.5 Hz, 2H), 2.92–2.77 (m, 1H), 1.33 (td, *J* = 7.0, 3.0 Hz, 6H), 1.12 (dt, *J* = 7.0, 2.0 Hz, 6H); ^{13}C NMR (125 MHz, $CDCl_3$): δ 206.20, 62.62 (d, *J* = 6.4 Hz), 41.84, 40.26 (d, *J* = 128.5 Hz), 18.07, 16.45 (d, *J* = 6.2 Hz); LRMS (ESI): *m/z* calcd for $C_9H_{20}O_4P$ $[M+H]^+$: 223.11; Found: 223.1.

Compound 5c: diethyl (2-oxo-2-phenylethyl)phosphonate

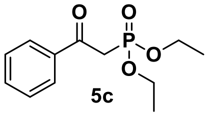
5c

Compound **5c** was prepared using the procedure reported in the literature[14].

IV. Experimental Synthetic Procedures and Characterization of the Final Products

Compound 3aa: ethyl 6-methyl-2'-(phenylsulfonamido)-[3,3'-bipyridine]-5-carboxylate

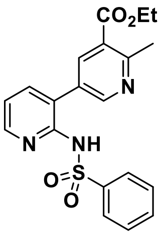

3aa

Brown solid; Yield: 70% (56 mg) ; ^{1}H NMR (500 MHz, $CDCl_3$): δ 8.67 (d, *J* = 3.0 Hz, 1H), 8.33 (d, *J* = 2.5 Hz, 1H), 7.99 (d, *J* = 7.5 Hz, 2H), 7.81 (d, *J* = 6.5 Hz, 1H), 7.63 (d, *J* = 7.0 Hz, 1H), 7.55–7.44 (m, 3H), 6.76 (t, *J* = 7.0 Hz, 1H), 4.37 (q, *J* = 7.0 Hz, 2H), 2.83 (s, 3H), 1.37 (t, *J* = 7.0 Hz, 3H); ^{13}C NMR (125 MHz, $CDCl_3$): δ 166.26, 159.50, 151.68, 151.38, 142.85, 141.09, 139.05, 136.22, 132.12, 129.21, 128.85, 127.58, 126.33, 125.22, 112.82, 61.47, 24.65, 14.34; HRMS (ESI+): *m/z* calcd for $C_{20}H_{21}N_3O_4S$ $[M+H]^+$: 398.1175; Found: 398.1178.

Compound 3a'a: ethyl 2-methyl-5-(2-(phenylsulfonamido)phenyl)nicotinate

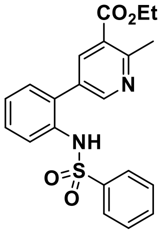

3a'a

Brown solid; Yield: 69% (55 mg); ^{1}H NMR (400 MHz, $CDCl_3$): δ 8.17 (d, *J* = 2.4 Hz, 1H), 7.83 (d, *J* = 2.4 Hz, 1H), 7.66 (dd, *J* = 8.0, 1.2 Hz, 1H), 7.61–7.57 (m, 2H), 7.55–7.49 (m, 1H), 7.43–7.35 (m, 3H), 7.28–7.21 (m, 1H), 7.13 (dd, *J* = 7.6, 1.6 Hz, 1H), 7.12 (br. s, 1H), 4.39 (q, *J* = 7.2 Hz, 2H), 2.81 (s, 3H), 1.41 (t, *J* = 7.2 Hz, 3H); ^{13}C NMR (100 MHz, $CDCl_3$): δ 166.05, 159.17, 151.31, 139.31, 138.77, 133.86, 133.15, 131.18, 131.02, 130.88, 129.76, 129.15, 127.12, 126.23, 125.69, 124.04, 61.67, 24.58, 14.39; LRMS (ESI): *m/z* calcd for $C_{21}H_{21}N_2O_4S$ $[M+H]^+$: 397.12; Found: 397.05.

Compound 3ab: ethyl 2'-(phenylsulfonamido)-6-propyl-[3,3'-bipyridine]-5-carboxylate

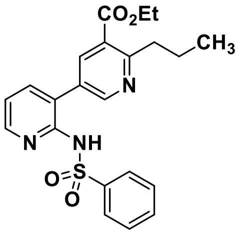

3ab

Off-white solid; Yield: 60% (51 mg); ^{1}H NMR (400 MHz, $CDCl_3$): δ 8.69 (d, *J* = 2.4 Hz, 1H), 8.30 (d, *J* = 2.4 Hz, 1H), 7.99 (d, *J* = 7.2 Hz, 2H), 7.79 (d, *J* = 6.4 Hz, 1H), 7.64 (dd, *J* = 7.2, 2.0 Hz, 1H), 7.55–7.43 (m, 3H), 6.76 (t, *J* = 6.8 Hz, 1H), 4.37 (q, *J* = 7.2 Hz, 2H), 3.20–3.06 (m, 2H), 1.84–1.70 (m, 2H), 1.38 (t, *J* = 7.2 Hz, 3H), 1.03 (t, *J* = 7.2 Hz, 3H); ^{13}C NMR (100 MHz, $CDCl_3$): δ 166.51, 163.06, 151.74, 151.36, 142.86, 141.17, 139.11, 135.80, 132.15, 128.98, 128.88, 128.07, 126.31, 125.30, 112.16, 61.55, 38.94, 23.47, 14.36, 14.32; LRMS (ESI): *m/z* calcd for $C_{22}H_{24}N_3O_4S$ $[M+H]^+$: 426.15; Found: 426.15.

Compound 3a'b: ethyl 5-(2-(phenylsulfonamido)phenyl)-2-propylnicotinate

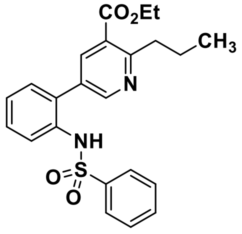

3a'b

Off-white solid; Yield: 65% (55 mg) ; ^{1}H NMR (400 MHz, $CDCl_3$): δ 8.17 (d, *J* = 2.4 Hz, 1H), 7.73 (d, *J* = 2.0 Hz, 1H), 7.67 (dd, *J* = 8.0, 1.2 Hz, 1H), 7.57–7.46 (m, 3H), 7.44–7.39 (m, 1H), 7.39–7.33 (m, 2H), 7.26 (td, *J* = 7.6, 1.2 Hz, 1H), 7.13 (dd, *J* = 7.6, 1.6 Hz, 1H), 6.94 (s, 1H), 4.40 (q, *J* = 7.2 Hz, 2H), 3.17–3.05 (m, 2H), 1.82–1.70 (m, 2H), 1.42 (t, *J* = 7.2 Hz, 3H), 1.04 (t, *J* = 7.2 Hz, 3H); ^{13}C NMR (100 MHz, $CDCl_3$): δ 166.27, 162.72, 151.24, 139.20, 138.65, 133.69, 133.15, 131.55, 130.82, 130.74, 129.74, 129.15, 127.06, 126.41, 125.75, 124.56, 61.72, 38.83, 23.51, 14.38, 14.35; HRMS (ESI+): *m/z* calcd for $C_{23}H_{25}N_2O_4S$ $[M+H]^+$: 425.1535; Found: 425.1544.

Compound 3ac: ethyl 6-isopropyl-2'-(phenylsulfonamido)-[3,3'-bipyridine]-5-carboxylate

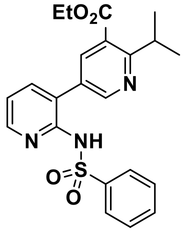

3ac

Brown solid; Yield: 39% (33 mg); ^{1}H NMR (500 MHz, $CDCl_3$): δ 8.72 (d, *J* = 2.5 Hz, 1H), 8.20 (d, *J* = 2.0 Hz, 1H), 8.03–7.96 (m, 2H), 7.80 (d, *J* = 6.0 Hz, 1H), 7.63 (dd, *J* = 7.0, 2.0 Hz, 1H), 7.56–7.51 (m, 1H), 7.50–7.45 (m, 2H), 6.77 (t, *J* = 6.5 Hz, 1H), 4.37 (q, *J* = 7.0 Hz, 2H), 3.85 (hept, *J* = 6.5 Hz, 1H), 1.38 (t, *J* = 7.0 Hz, 3H), 1.33 (d, *J* = 6.5 Hz, 6H); ^{13}C NMR (125 MHz, $CDCl_3$): δ 167.05, 166.96, 151.72, 151.23, 142.83, 141.08, 138.57, 136.66, 132.17, 128.86, 128.64, 128.05, 126.39, 125.18, 112.85, 61.60, 32.37, 22.37, 14.31; HRMS (ESI+): *m/z* calcd for $C_{22}H_{24}N_3O_4S$ $[M+H]^+$: 426.1488; Found: 426.1494.

Compound 3a'c: ethyl 2-isopropyl-5-(2-(phenylsulfonamido)phenyl)nicotinate

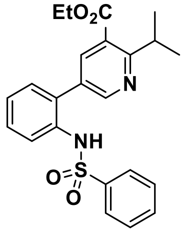

3a'c

Pale-yellow solid; Yield: 59% (50 mg); ^{1}H NMR (500 MHz, $CDCl_3$): δ 8.21 (d, *J* = 2.0 Hz, 1H), 7.70–7.65 (m, 1H), 7.59 (d, *J* = 2.0 Hz, 1H), 7.56–7.49 (m, 3H), 7.44–7.33 (m, 3H), 7.24 (t, *J* = 8.0 Hz, 1H), 7.11 (dd, *J* = 7.5, 2.5 Hz, 1H), 6.68 (s, 1H), 4.40 (q, *J* = 7.5 Hz, 2H), 3.83 (hept, *J* = 6.5 Hz, 1H), 1.42 (t, *J* = 7.5 Hz, 3H), 1.33 (d, *J* = 6.5 Hz, 6H); ^{13}C NMR (125 MHz, $CDCl_3$): δ 166.74, 166.66, 151.21, 139.04, 137.93, 133.66, 133.21, 131.30, 130.80, 130.25, 129.68, 129.17, 127.05, 126.29, 125.57, 124.22, 61.77, 32.35, 22.40, 14.33; LRMS (ESI): *m/z* calcd for $C_{23}H_{25}N_2O_4S$ $[M+H]^+$: 425.15; Found: 425.10.

Compound 3ad: ethyl 6-cyclopropyl-2'-(phenylsulfonamido)-[3,3'-bipyridine]-5-carboxylate

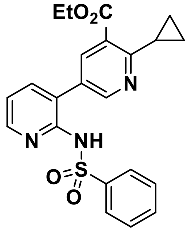

3ad

Off-white solid; Yield: 61% (52 mg); ^{1}H NMR (500 MHz, $CDCl_3$): δ 12.76 (br. s, 1H), 8.58 (d, *J* = 2.5 Hz, 1H), 8.23 (d, *J* = 2.0 Hz, 1H), 7.98 (d, *J* = 7.5 Hz, 2H), 7.77 (s, 1H), 7.63–7.57 (m, 1H), 7.56–7.50 (m, 1H), 7.49–7.43 (m, 2H), 6.75 (t, *J* = 7.0 Hz, 1H), 4.38 (q, *J* = 7.0 Hz, 2H), 3.05 (tt, *J* = 8.0, 5.0 Hz, 1H), 1.37 (t, *J* = 7.0 Hz, 3H), 1.22–1.17 (m, 2H), 1.08–1.02 (m, 2H); ^{13}C NMR (125 MHz, $CDCl_3$): δ 166.92, 163.59, 151.94, 151.56, 142.86, 140.87, 138.51, 134.05, 132.14, 128.85, 128.15, 127.56, 126.37, 125.06, 112.45, 61.52, 14.38 (2C), 11.50 (2C); LRMS (ESI): *m/z* calcd for $C_{22}H_{22}N_3O_4S$ $[M+H]^+$: 424.13; Found: 424.05.

Compound 3a'd: ethyl 2-cyclopropyl-5-(2-(phenylsulfonamido)phenyl)nicotinate

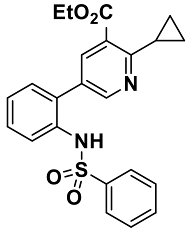

3a'd

Pale-yellow solid; Yield: 63% (53 mg); ^{1}H NMR (400 MHz, $CDCl_3$): δ 8.05 (d, *J* = 2.4 Hz, 1H), 7.70–7.61 (m, 2H), 7.57–7.48 (m, 3H), 7.42–7.33 (m, 3H), 7.23 (td, *J* = 7.6, 1.2 Hz, 1H), 7.09 (dd, *J* = 7.6, 1.6 Hz, 1H), 6.69 (s, 1H), 4.40 (q, *J* = 7.2 Hz, 2H), 3.02 (tt, *J* = 8.0, 4.8 Hz, 1H), 1.41 (t, *J* = 7.2 Hz, 3H), 1.22–1.16 (m, 2H), 1.11–1.01 (m, 2H); ^{13}C NMR (125 MHz, $CDCl_3$): δ 166.70, 163.17, 151.44, 139.09, 137.99, 133.69, 133.15, 131.47, 130.76, 129.57, 129.17, 129.11, 127.05, 126.26, 125.43, 124.19, 61.66, 14.38, 14.27, 11.54 (2C); HRMS (ESI+): *m/z* calcd for $C_{23}H_{23}N_2O_4S$ $[M+H]^+$: 423.1379; Found: 423.1386.

Compound 3ae: ethyl 6-cyclohexyl-2'-(phenylsulfonamido)-[3,3'-bipyridine]-5-carboxylate

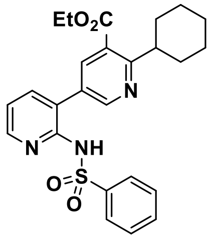

3ae

White solid; Yield: 57% (53 mg); ^{1}H NMR (500 MHz, $CDCl_3$): δ 8.69 (d, *J* = 2.5 Hz, 1H), 8.20 (d, *J* = 2.5 Hz, 1H), 8.00 (d, *J* = 7.5 Hz, 2H), 7.79 (s, 1H), 7.66–7.56 (m, 1H), 7.53 (td, *J* = 7.5, 1.5 Hz, 1H), 7.49–7.44 (m, 2H), 6.76 (t, *J* = 7.0 Hz, 1H), 4.37 (q, *J* = 7.0 Hz, 2H), 3.51–3.39 (m, 1H), 1.90–1.83 (m, 4H), 1.77–1.67 (m, 3H), 1.45–1.32 (m, 6H); ^{13}C NMR (125 MHz, $CDCl_3$): δ 167.02, 166.24, 151.77, 151.13, 142.88, 141.12, 138.68, 135.89, 132.16, 128.87, 128.54, 128.52, 126.35, 125.28, 112.55, 61.57, 42.85, 32.48, 26.68, 26.14, 14.34; HRMS (ESI+): *m/z* calcd for $C_{25}H_{28}N_2O_4S$ $[M+H]^+$: 466.1801; Found: 466.1808.

Compound 3a'e: ethyl 2-cyclohexyl-5-(2-(phenylsulfonamido)phenyl)nicotinate

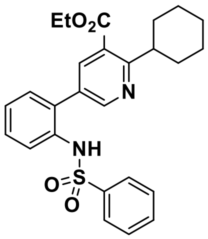

3a'e

White solid; Yield: 55% (51 mg); ^{1}H NMR (400 MHz, $CDCl_3$): δ 8.19 (d, *J* = 2.4 Hz, 1H), 7.69 (d, *J* = 8.4 Hz, 1H), 7.61–7.47 (m, 4H), 7.43–7.32 (m, 3H), 7.24 (t, *J* = 7.6 Hz, 1H), 7.10 (d, *J* = 7.6 Hz, 1H), 6.53 (s, 1H), 4.41 (q, *J* = 7.2 Hz, 2H), 3.51–3.39 (m, 1H), 1.93–1.84 (m, 4H), 1.70–1.66 (m, 3H), 1.50–1.32 (m, 6H); ^{13}C NMR (100 MHz, $CDCl_3$): δ 166.82, 165.99, 151.13, 139.06, 138.01, 133.74, 133.27, 131.14, 130.83, 130.12, 129.73, 129.22, 127.11, 126.24, 125.79, 124.00, 61.79, 42.89, 32.57, 26.73, 26.17, 14.41; LRMS (ESI): *m/z* calcd for $C_{26}H_{29}N_2O_4S$ $[M+H]^+$: 465.18; Found: 465.15.

Compound 3af: ethyl 6-phenyl-2'-(phenylsulfonamido)-[3,3'-bipyridine]-5-carboxylate

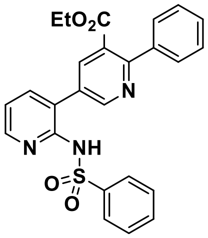

3af

White solid; Yield: 44% (41 mg); ^{1}H NMR (500 MHz, $CDCl_3$): δ 12.83 (br. s, 1H), 8.83 (d, *J* = 2.5 Hz, 1H), 8.22 (d, *J* = 2.5 Hz, 1H), 8.01 (d, *J* = 7.0 Hz, 2H), 7.82–7.64 (m, 2H), 7.57–7.47 (m, 5H), 7.45–7.40 (m, 3H), 6.76 (s, 1H), 4.16 (q, *J* = 7.0 Hz, 2H), 1.05 (t, *J* = 7.0 Hz, 3H); ^{13}C NMR (125 MHz, $CDCl_3$): δ 167.84, 158.46, 151.99, 150.96, 142.95, 141.37, 139.84, 138.57, 134.08, 132.22, 129.79, 128.98, 128.91, 128.72, 128.38, 128.27, 126.92, 126.31, 111.95, 61.71, 13.77; LRMS (ESI): *m/z* calcd for $C_{25}H_{22}N_3O_4S$ $[M+H]^+$: 460.13; Found: 460.05.

Compound 3a'f: ethyl 2-phenyl-5-(2-(phenylsulfonamido)phenyl)nicotinate

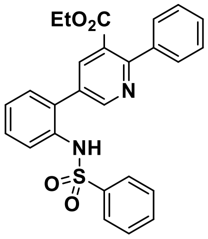

3a'f

Pale-yellow solid; Yield: 70% (63 mg); ^{1}H NMR (400 MHz, $CDCl_3$): δ 8.35 (d, *J* = 2.4 Hz, 1H), 7.69–7.49 (m, 7H), 7.48–7.35 (m, 6H), 7.28 (t, *J* = 7.6 Hz, 1H), 7.17 (d, *J* = 7.6 Hz, 1H), 6.84 (s, 1H), 4.17 (q, *J* = 7.2 Hz, 2H), 1.07 (t, *J* = 7.2 Hz, 3H); ^{13}C NMR (100 MHz, $CDCl_3$); δ 167.68, 158.17, 150.87, 139.60, 139.12, 138.15, 133.74, 133.34, 131.62, 131.22, 130.89, 129.97, 129.24, 129.08, 128.72, 128.38, 127.41, 127.15, 126.51, 124.61, 61.88, 13.79; HRMS (ESI+): *m/z* calcd for $C_{26}H_{23}N_2O_4S$ $[M+H]^+$: 459.1379; Found: 459.1385.

Compound 3ba: ethyl 5-(2-bromo-6-(phenylsulfonamido)phenyl)-2-methylnicotinate

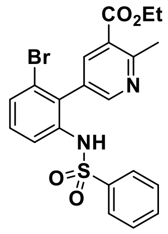

3ba

Off-white solid; Yield: 53% (50 mg); ^{1}H NMR (400 MHz, $CDCl_3$): δ 7.92 (d, *J* = 2.0 Hz, 1H), 7.76–7.68 (m, 2H), 7.64–7.56 (m, 3H), 7.50–7.43 (m, 3H), 7.26 (t, *J* = 8.0 Hz, 1H), 6.57 (br. s, 1H), 4.39 (q, *J* = 7.2 Hz, 2H), 2.86 (s, 3H), 1.41 (t, *J* = 7.2 Hz, 3H); ^{13}C NMR (100 MHz, $CDCl_3$); δ 165.87, 160.39, 151.96, 139.61, 138.89, 136.17, 133.57, 130.68, 130.38, 129.58, 129.49, 129.40, 127.14, 125.96, 124.87, 120.97, 61.73, 24.83, 14.39; HRMS (ESI): *m/z* calcd for $C_{21}H_{20}BrN_2O_4S$ $[M+H]^+$: 475.0327; Found: 475.0339.

Compound 3ca: ethyl 5-(5-bromo-2-(phenylsulfonamido)phenyl)-2-methylnicotinate

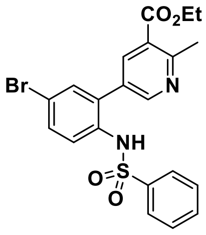

3ca

White solid; Yield: 63% (60 mg); ^{1}H NMR (400 MHz, $CDCl_3$): δ 8.45 (d, *J* = 2.4 Hz, 1H), 8.11 (d, *J* = 2.4 Hz, 1H), 7.69 (s, 1H), 7.59–7.45 (m, 4H), 7.39 (t, *J* = 8.0 Hz, 2H), 7.36–7.29 (m, 2H), 4.42 (q, *J* = 7.2 Hz, 2H), 2.89 (s, 3H), 1.42 (t, *J* = 7.2 Hz, 3H); ^{13}C NMR (100 MHz, $CDCl_3$); δ 164.68, 157.92, 148.65, 141.58, 139.09, 133.44, 133.31, 133.21, 133.18, 133.06, 131.74, 129.27, 127.20, 127.09, 120.23, 62.27, 22.65, 14.33; LRMS (ESI): *m/z* calcd for $C_{21}H_{20}BrN_2O_4S$ $[M+H]^+$: 475.03; Found: 475.0.

Compound 3da: ethyl 5-(4-bromo-2-(phenylsulfonamido)phenyl)-2-methylnicotinate

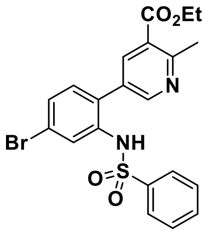

3da

White solid; Yield: 60% (57 mg); ^{1}H NMR (400 MHz, $CDCl_3$): δ 8.21 (d, *J* = 2.8 Hz, 1H), 7.86 (d, *J* = 2.4 Hz, 1H), 7.82 (d, *J* = 2.0 Hz, 1H), 7.66 (d, *J* = 7.6 Hz, 3H), 7.56 (t, *J* = 7.6 Hz, 1H), 7.44 (t, *J* = 7.6 Hz, 2H), 7.36 (dd, *J* = 8.0, 2.4 Hz, 1H), 7.00 (dd, *J* = 8.0, 2.4 Hz, 1H), 4.38 (q, *J* = 7.2 Hz, 2H), 2.75 (s, 3H), 1.41 (t, *J* = 7.2 Hz, 3H); ^{13}C NMR (100 MHz, $CDCl_3$); δ 165.62, 159.07, 150.78, 138.97, 138.94, 135.20, 133.29, 131.92, 130.27, 129.35, 129.20, 128.97, 127.02, 126.25, 125.82, 123.20, 61.68, 24.21, 14.23; LRMS (ESI): *m/z* calcd for $C_{21}H_{20}BrN_2O_4S$ $[M+H]^+$: 475.03; Found: 475.0.

Compound 3ea: ethyl 2-methyl-5-(2-nitro-6-(phenylsulfonamido)phenyl)nicotinate

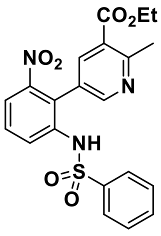

3ea

Yellow solid; Yield: 54% (48 mg); ^{1}H NMR (500 MHz, $CDCl_3$): δ 8.03 (d, *J* = 8.5 Hz, 1H), 7.97–7.90 (m, 1H), 7.79–7.71 (m, 2H), 7.58 (dd, *J* = 24.6, 8.3 Hz, 4H), 7.48 (t, *J* = 8.0 Hz, 2H), 6.77 (s, 1H), 4.37 (q, *J* = 7.0 Hz, 2H), 2.84 (s, 3H), 1.40 (t, *J* = 7.0 Hz, 3H); ^{13}C NMR (125 MHz, $CDCl_3$); δ 165.58, 160.96, 150.90, 150.03, 138.94, 138.60, 136.87, 133.88, 130.26, 129.58, 127.08, 126.04, 125.96, 125.21, 123.73, 120.74, 61.81, 24.84, 14.35; LRMS (ESI): *m/z* calcd for $C_{21}H_{20}N_3O_6S$ $[M+H]^+$: 442.11; Found: 442.00.

Compound 3fa: ethyl 2-methyl-5-(5-nitro-2-(phenylsulfonamido)phenyl)nicotinate

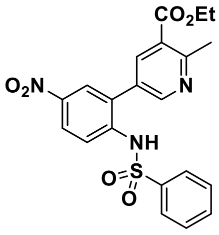

3fa

Off-white solid; Yield: 65% (57 mg); ^{1}H NMR (500 MHz, $CDCl_3$): δ 8.38 (s, 1H), 8.24 (dd, *J* = 9.0, 3.0 Hz, 1H), 8.04–7.97 (m, 2H), 7.87 (d, *J* = 9.5 Hz, 1H), 7.84 (d, *J* = 7.5 Hz, 2H), 7.80 (br. s, 1H), 7.64 (t, *J* = 7.5 Hz, 1H), 7.54 (t, *J* = 7.5 Hz, 2H), 4.42 (q, *J* = 7.0 Hz, 2H), 2.78 (s, 3H), 1.43 (t, *J* = 7.0 Hz, 3H);^{13}C NMR (125 MHz, $CDCl_3$); δ 165.51, 160.62, 151.24, 143.97, 140.55, 139.08, 138.76, 134.10, 129.74, 128.92, 128.54, 127.32, 126.60, 126.42, 125.26, 119.76, 62.02, 24.72, 14.40; HRMS (ESI): *m/z* calcd for $C_{21}H_{20}N_3O_6S$ $[M+H]^+$: 442.1073; Found: 442.1078.

Compound 3ga: ethyl 2-methyl-5-(4-nitro-2-(phenylsulfonamido)phenyl)nicotinate

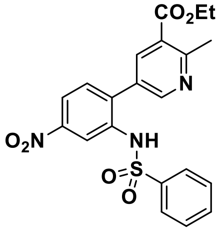

3ga

Off-white solid; Yield: 66% (58 mg); ^{1}H NMR (500 MHz, $CDCl_3$): δ 8.52 (s, 1H), 8.33 (s, 1H), 8.04 (d, *J* = 8.5 Hz, 1H), 7.96 (s, 1H), 7.78 (d, *J* = 7.5 Hz, 2H), 7.61 (t, *J* = 7.5 Hz, 1H), 7.55–7.47 (m, 3H), 7.32 (d, *J* = 8.5 Hz, 1H), 4.41 (q, *J* = 7.0 Hz, 2H), 2.80 (s, 3H), 1.43 (t, *J* = 7.0 Hz, 3H); ^{13}C NMR (125 MHz, $CDCl_3$); δ 165.65, 160.53, 150.91, 148.64, 138.78, 138.70, 135.72, 135.50, 133.90, 131.87, 129.64, 129.24, 127.39, 126.21, 119.94, 116.73, 62.00, 24.74, 14.40; LRMS (ESI): *m/z* calcd for $C_{21}H_{20}N_3O_6S$ $[M+H]^+$: 442.11; Found: 442.05.

Compound 3ha: ethyl 5-(2-methoxy-6-(phenylsulfonamido)phenyl)-2-methylnicotinate

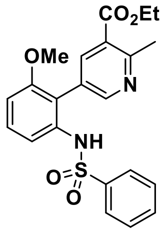

3ha

Off-white solid; Yield: 42% (36 mg); ^{1}H NMR (500 MHz, $CDCl_3$): δ 7.97 (d, *J* = 2.0 Hz, 1H), 7.73 (d, *J* = 2.0 Hz, 1H), 7.66–7.60 (m, 2H), 7.59–7.54 (m, 1H), 7.44 (t, *J* = 8.0 Hz, 2H), 7.38–7.32 (m, 2H), 6.75 (dd, *J* = 7.5, 1.5 Hz, 1H), 6.33 (s, 1H), 4.40 (q, *J* = 7.0 Hz, 2H), 3.64 (s, 3H), 2.87 (s, 3H), 1.42 (t, *J* = 7.0 Hz, 3H);^{13}C NMR (125 MHz, $CDCl_3$);δ 166.23, 159.53, 157.56, 152.84, 140.17, 139.03, 135.47, 133.35, 130.28, 129.26, 127.22, 126.41, 125.91, 118.45, 114.12, 107.74, 61.61, 55.86, 24.81, 14.44; LRMS (ESI): *m/z* calcd for $C_{22}H_{23}N_2O_5S$ $[M+H]^+$: 427.13; Found: 427.10.

Compound 3ia: ethyl 5-(5-methoxy-2-(phenylsulfonamido)phenyl)-2-methylnicotinate

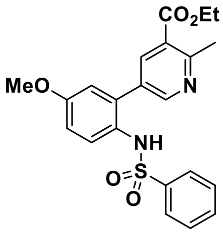

3ia

Pale-yellow solid; Yield: 60% (51 mg); ^{1}H NMR (400 MHz, $CDCl_3$): δ 8.13 (d, *J* = 2.4 Hz, 1H), 7.73 (d, *J* = 2.4 Hz, 1H), 7.53–7.45 (m, 2H), 7.45–7.38 (m, 2H), 7.34–7.26 (m, 2H), 6.98–6.89 (m, 2H), 6.67 (d, *J* = 2.8 Hz, 1H), 4.39 (q, *J* = 7.2 Hz, 2H), 3.81 (s, 3H), 2.83 (s, 3H), 1.42 (t, *J* = 7.2 Hz, 3H); ^{13}C NMR (100 MHz, $CDCl_3$): δ 166.10, 158.97, 158.46, 151.02, 139.31, 138.49, 135.03, 132.88, 131.27, 128.97, 128.92, 127.07, 125.91, 125.50, 116.07, 114.69, 61.65, 55.72, 24.59, 14.43; LRMS (ESI): *m/z* calcd for $C_{22}H_{23}N_2O_5S$ $[M+H]^+$: 427.13; Found: 427.10.

Compound 3ja: ethyl 5-(4-methoxy-2-(phenylsulfonamido)phenyl)-2-methylnicotinate

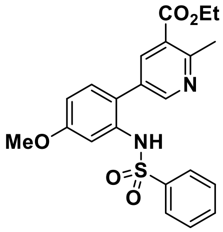

3ja

Pale-yellow solid; Yield: 60% (51 mg); ^{1}H NMR (400 MHz, $CDCl_3$): δ 8.15 (d, *J* = 2.4 Hz, 1H), 7.80 (d, *J* = 2.4 Hz, 1H), 7.70–7.61 (m, 2H), 7.57–7.50 (m, 1H), 7.45–7.37 (m, 2H), 7.24 (d, *J* = 2.8 Hz, 1H), 7.09 (s, 1H), 7.02 (d, *J* = 8.4 Hz, 1H), 6.77 (dd, *J* = 8.4, 2.8 Hz, 1H), 4.39 (q, *J* = 7.2 Hz, 2H), 3.84 (s, 3H), 2.79 (s, 3H), 1.41 (t, *J* = 7.2 Hz, 3H); ^{13}C NMR (100 MHz, $CDCl_3$): δ 166.12, 160.55, 158.84, 151.62, 139.25, 138.94, 134.98, 133.25, 131.63, 130.84, 129.21, 127.20, 125.71, 122.77, 112.02, 108.51, 61.65, 55.67, 24.54, 14.39; HRMS (ESI): *m/z* calcd for $C_{22}H_{23}N_2O_5S$ $[M+H]^+$: 427.1328; Found: 427.1333.

Compound 3ka: ethyl 5-(3-methoxy-2-(phenylsulfonamido)phenyl)-2-methylnicotinate

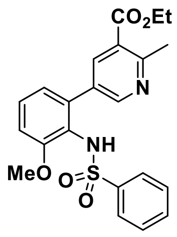

3ka

Yellow solid; Yield: 60% (43 mg); ^{1}H NMR (400 MHz, $CDCl_3$): δ 8.70 (d, *J* = 2.0 Hz, 1H), 8.25 (d, *J* = 2.4 Hz, 1H), 7.50–7.40 (m, 3H), 7.33–7.26 (m, 3H), 7.08 (s, 1H), 6.95 (d, *J* = 7.6 Hz, 1H), 6.80 (d, *J* = 8.4 Hz, 1H), 4.38 (q, *J* = 7.2 Hz, 2H), 3.48 (s, 3H), 2.83 (s, 3H), 1.42 (t, *J* = 7.2 Hz, 3H); ^{13}C NMR (100 MHz, $CDCl_3$): δ 166.49, 158.19, 155.40, 152.04, 140.27, 139.13, 137.61, 132.81, 132.26, 128.92, 128.35, 127.21, 125.06, 122.98, 122.70, 110.80, 61.33, 55.59, 24.61, 14.44; LRMS (ESI): *m/z* calcd for $C_{22}H_{23}N_2O_5S$ $[M+H]^+$: 427.13; Found: 427.10.

Compound 3la: ethyl 6'-methyl-3-(phenylsulfonamido)-[2,3'-bipyridine]-5'-carboxylate

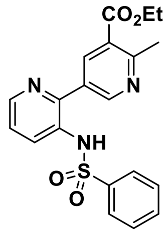

3la

Off-white solid; Yield: 45% (36 mg); ^{1}H NMR (400 MHz, $CDCl_3$): δ 8.50 (dd, *J* = 4.4, 1.6 Hz, 1H), 8.44 (t, *J* = 2.4 Hz, 1H), 8.08 (d, *J* = 2.4 Hz, 1H), 8.02 (dd, *J* = 8.4, 1.6 Hz, 1H), 7.67–7.60 (m, 2H), 7.55 (t, *J* = 7.6 Hz, 1H), 7.41 (t, *J* = 7.6 Hz, 2H), 7.34 (dd, *J* = 8.4, 4.4 Hz, 1H), 4.39 (q, *J* = 7.2 Hz, 2H), 2.81 (s, 3H), 1.41 (t, *J* = 7.2 Hz, 3H); ^{13}C NMR (100 MHz, $CDCl_3$):δ 165.76, 159.68, 150.89, 148.56, 146.93, 139.26, 139.10, 133.43, 131.72, 131.58, 130.87, 129.37, 127.14, 125.85, 123.93, 61.66, 24.46, 14.35; LRMS (ESI): *m/z* calcd for $C_{20}H_{20}N_3O_4S$ $[M+H]^+$: 398.12; Found: 398.05.

Compound 3ma: ethyl 6-methyl-4'-(phenylsulfonamido)-[3,3'-bipyridine]-5-carboxylate

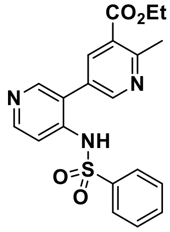

3ma

White solid; Yield: 36% (29 mg); ^{1}H NMR (500 MHz, DMSO-d_6): δ 13.05 (br. s, 1H), 8.75 (d, *J* = 2.0 Hz, 1H), 8.40 (d, *J* = 2.5 Hz, 1H), 8.21 (d, *J* = 1.5 Hz, 1H), 8.01 (dd, *J* = 7.0, 1.5 Hz, 1H), 7.82–7.76 (m, 2H), 7.54–7.46 (m, 3H), 7.41 (d, *J* = 7.5 Hz, 1H), 4.33 (q, *J* = 7.0 Hz, 2H), 2.75 (s, 3H), 1.32 (t, *J* = 7.0 Hz, 3H); ^{13}C NMR (125 MHz, DMSO-d_6): δ 165.99, 160.13, 157.44, 151.58, 144.05, 138.79, 138.01 (2C; merged), 131.13, 128.64 (2C), 128.56, 125.75 (2C), 124.31, 123.99, 112.61, 61.11, 24.10, 14.05; LRMS (ESI): *m/z* calcd for $C_{20}H_{20}N_3O_4S$ $[M+H]^+$: 398.12; Found: 398.15.

Compound 3na: ethyl 6-methyl-3'-(phenylsulfonamido)-[3,4'-bipyridine]-5-carboxylate

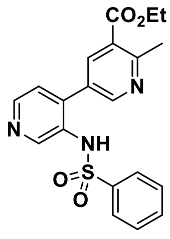

3na

White solid; Yield: 45% (36 mg); ^{1}H NMR (400 MHz, $CDCl_3$): δ 8.75 (s, 1H), 8.52 (d, *J* = 5.2 Hz, 1H), 8.32 (d, *J* = 2.4 Hz, 1H), 7.95 (d, *J* = 2.8 Hz, 1H), 7.66–7.50 (m, 4H), 7.40 (t, *J* = 8.0 Hz, 2H), 7.15 (d, *J* = 4.4 Hz, 1H), 4.41 (q, *J* = 7.2 Hz, 2H), 2.82 (s, 3H), 1.43 (t, *J* = 7.2 Hz, 3H); ^{13}C NMR (100 MHz, $CDCl_3$): δ 165.79, 160.23, 150.61, 147.73, 146.93, 140.09, 139.19, 138.42, 133.43, 130.46, 129.36, 128.90, 127.18, 125.95, 124.69, 61.90, 24.67, 14.40; LRMS (ESI): *m/z* calcd for $C_{20}H_{20}N_3O_4S$ $[M+H]^+$: 398.12; Found: 398.05.

Compound 3oa: ethyl 2'-(benzylamino)-6-methyl-[3,3'-bipyridine]-5-carboxylate

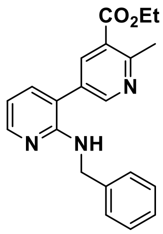

3oa

Yellow solid; Yield: 44% (31 mg); ^{1}H NMR (400 MHz, $CDCl_3$): δ 8.67 (d, *J* = 2.4 Hz, 1H), 8.27 (d, *J* = 2.4 Hz, 1H), 8.21 (dd, *J* = 5.2, 1.6 Hz, 1H), 7.34–7.28 (m, 5H), 7.26–7.22 (m, 1H), 6.72 (dd, *J* = 7.6, 5.2 Hz, 1H), 4.65 (s, 3H), 4.38 (q, *J* = 7.2 Hz, 2H), 2.86 (s, 3H), 1.39 (t, *J* = 7.2 Hz, 3H); ^{13}C NMR (100 MHz, $CDCl_3$): δ 166.26, 159.36, 155.54, 151.76, 148.38, 139.61, 138.81, 137.80, 131.22, 128.71, 127.75, 127.31, 126.05, 117.70, 113.37, 61.62, 45.79, 24.70, 14.39; LRMS (ESI): *m/z* calcd for $C_{21}H_{22}N_3O_2$ $[M+H]^+$: 348.17; Found: 348.10.

Compound 3o'a: ethyl 5-(2-(benzylamino)phenyl)-2-methylnicotinate

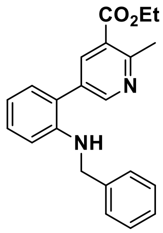

3o'a

Yellow solid; Yield: 29% (20 mg); ^{1}H NMR (400 MHz, $CDCl_3$): δ 8.72 (d, *J* = 2.4 Hz, 1H), 8.33 (d, *J* = 2.4 Hz, 1H), 7.35–7.28 (m, 4H), 7.27–7.22 (m, 2H), 7.09 (dd, *J* = 7.6, 1.6 Hz, 1H), 6.82 (td, *J* = 7.6, 1.2 Hz, 1H), 6.72 (d, *J* = 8.0 Hz, 1H), 4.39 (q, *J* = 7.2 Hz, 2H), 4.32 (d, *J* = 5.2 Hz, 2H), 4.12 (t, *J* = 5.2 Hz, 1H), 2.87 (s, 3H), 1.40 (t, *J* = 7.2 Hz, 3H); ^{13}C NMR (100 MHz, $CDCl_3$): δ 166.52, 158.73, 152.26, 145.24, 139.28, 139.06, 132.61, 130.59, 129.91, 128.81, 127.42, 127.35, 125.85, 123.02, 117.79, 111.32, 61.50, 48.29, 24.67, 14.40; LRMS (ESI): *m/z* calcd for $C_{22}H_{23}N_2O_2$ $[M+H]^+$: 347.18; Found: 347.2.

Compound 3pa: ethyl 2'-((4-chlorobenzyl)amino)-6-methyl-[3,3'-bipyridine]-5-carboxylate

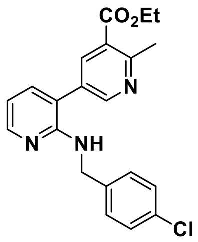

3pa

Yellow solid; Yield: 50% (38 mg); ^{1}H NMR (400 MHz, $CDCl_3$): δ 8.66 (d, *J* = 2.4 Hz, 1H), 8.25 (d, *J* = 2.4 Hz, 1H), 8.19 (dd, *J* = 5.2, 2.0 Hz, 1H), 7.31 (dd, *J* = 7.6, 2.0 Hz, 1H), 7.28–7.21 (m, 4H), 6.73 (dd, *J* = 7.2, 5.2 Hz, 1H), 4.73 (d, *J* = 5.6 Hz, 1H), 4.61 (d, *J* = 5.6 Hz, 2H), 4.39 (q, *J* = 7.2 Hz, 2H), 2.85 (s, 3H), 1.39 (t, *J* = 7.2 Hz, 3H); ^{13}C NMR (100 MHz, $CDCl_3$): δ 166.23, 159.36, 155.32, 151.71, 148.34, 138.78, 138.32, 137.84, 132.94, 131.13, 129.04, 128.75, 126.09, 117.71, 113.57, 61.66, 44.94, 24.69, 14.38; HRMS (ESI): *m/z* calcd for $C_{21}H_{21}ClN_3O_2$ $[M+H]^+$: 382.1322; Found: 382.1325.

Compound 3qa: ethyl 2'-((4-methoxybenzyl)amino)-6-methyl-[3,3'-bipyridine]-5-carboxylate

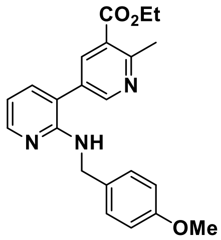

3qa

White solid; Yield: 27% (20 mg); ^{1}H NMR (400 MHz, $CDCl_3$): δ 8.65 (d, *J* = 2.4 Hz, 1H), 8.25 (d, *J* = 2.4 Hz, 1H), 8.21 (dd, *J* = 5.2, 1.6 Hz, 1H), 7.30 (dd, *J* = 7.2, 1.6 Hz, 1H), 7.25–7.21 (m, 2H), 6.86–6.80 (m, 2H), 6.71 (dd, *J* = 7.2, 5.2 Hz, 1H), 4.60 (t, *J* = 5.2 Hz, 1H), 4.56 (d, *J* = 5.2 Hz, 2H), 4.38 (q, *J* = 7.2 Hz, 2H), 3.77 (s, 3H), 2.85 (s, 3H), 1.39 (t, *J* = 7.2 Hz, 3H); ^{13}C NMR (100 MHz, $CDCl_3$): δ 166.24, 159.27, 158.93, 155.53, 151.72, 148.35, 138.78, 137.74, 131.59, 131.24, 129.09, 126.02, 117.66, 114.09, 113.25, 61.59, 55.37, 45.31, 24.67, 14.37; LRMS (ESI): *m/z* calcd for $C_{22}H_{24}N_3O_3$ $[M+H]^+$: 378.18; Found: 378.2.

Compound 3ra: ethyl 6-methyl-2'-((thiophen-2-ylmethyl)amino)-[3,3'-bipyridine]-5-carboxylate

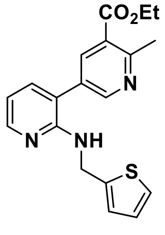

3ra

Yellow solid; Yield: 47% (33 mg); ^{1}H NMR (400 MHz, $CDCl_3$): δ 8.65 (d, *J* = 2.4 Hz, 1H), 8.27 (d, *J* = 2.4 Hz, 1H), 8.23 (dd, *J* = 5.2, 2.0 Hz, 1H), 7.32 (dd, *J* = 7.2, 2.0 Hz, 1H), 7.16 (dd, *J* = 4.8, 1.2 Hz, 1H), 6.96 (dd, *J* = 3.6, 1.2 Hz, 1H), 6.91 (dd, *J* = 5.2, 3.6 Hz, 1H), 6.75 (dd, *J* = 7.2, 4.8 Hz, 1H), 4.82 (d, *J* = 5.6 Hz, 2H), 4.76 (t, *J* = 5.6 Hz, 1H), 4.39 (q, *J* = 7.2 Hz, 2H), 2.85 (s, 3H), 1.40 (t, *J* = 7.2 Hz, 3H); ^{13}C NMR (100 MHz, $CDCl_3$): δ 166.24, 159.33, 154.99, 151.75, 148.23, 142.87, 138.77, 137.83, 131.05, 126.81, 125.99, 125.42, 124.73, 117.89, 113.71, 61.60, 40.62, 24.66, 14.39; LRMS (ESI): *m/z* calcd for $C_{19}H_{20}N_3O_2S$ $[M+H]^+$: 354.13; Found: 354.1.

Compound 3r'a: ethyl 2-methyl-5-(2-((thiophen-2-ylmethyl)amino)phenyl)nicotinate

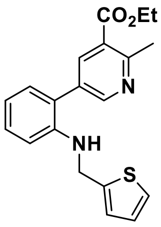

3r'a

Yellow solid; Yield: 40% (28 mg); ^{1}H NMR (400 MHz, $CDCl_3$): δ 8.70 (d, *J* = 2.4 Hz, 1H), 8.31 (d, *J* = 2.4 Hz, 1H), 7.28 (td, *J* = 8.0, 1.6 Hz, 1H), 7.18 (dd, *J* = 4.8, 1.2 Hz, 1H), 7.10 (dd, *J* = 7.6, 1.6 Hz, 1H), 6.99–6.90 (m, 2H), 6.85 (td, *J* = 7.6, 1.2 Hz, 1H), 6.81 (d, *J* = 8.4 Hz, 1H), 4.50 (d, *J* = 4.4 Hz, 2H), 4.39 (q, *J* = 7.2 Hz, 2H), 4.15 (t, *J* = 6.0 Hz, 1H), 2.87 (s, 3H), 1.40 (t, *J* = 7.2 Hz, 3H); ^{13}C NMR (100 MHz, $CDCl_3$): δ 166.51, 158.77, 152.24, 144.68, 142.81, 139.27, 132.43, 130.64, 129.87, 127.00, 125.81, 125.00, 124.73, 123.39, 118.28, 111.49, 61.50, 43.48, 24.65, 14.42; HRMS (ESI): *m/z* calcd for $C_{20}H_{21}N_2O_2S$ $[M+H]^+$: 353.1324; Found: 353.1326.

Compound 3sa: ethyl 6-methyl-2'-(methylamino)-[3,3'-bipyridine]-5-carboxylate

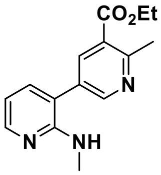

3sa

Yellow solid; Yield: 45% (24 mg); ^{1}H NMR (400 MHz, $CDCl_3$): δ 8.64 (d, *J* = 2.0 Hz, 1H), 8.29–8.10 (m, 2H), 7.28–7.25 (m, 1H), 6.68 (dd, *J* = 7.2, 4.8 Hz, 1H), 4.46–4.35 (m, 3H), 2.96 (d, *J* = 4.8 Hz, 3H), 2.87 (s, 3H), 1.41 (t, *J* = 7.2 Hz, 3H); ^{13}C NMR (100 MHz, $CDCl_3$): δ 166.39, 159.22, 156.49, 151.87, 148.37, 138.86, 137.47, 131.35, 126.11, 117.85, 112.74, 61.68, 28.82, 24.68, 14.39; LRMS (ESI): *m/z* calcd for $C_{15}H_{18}N_3O_2$ $[M+H]^+$: 272.14; Found: 272.10.

Compound 3ta: ethyl 2'-((cyclohexylmethyl)amino)-6-methyl-[3,3'-bipyridine]-5-carboxylate

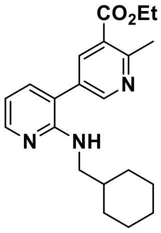

3ta

Pale-yellow solid; Yield: 62% (44 mg); ^{1}H NMR (500 MHz, $CDCl_3$): δ 8.67 (d, *J* = 2.5 Hz, 1H), 8.27 (d, *J* = 2.0 Hz, 1H), 8.17 (dd, *J* = 5.0, 1.5 Hz, 1H), 7.26–7.23 (m, 1H), 6.65 (dd, *J* = 7.5, 5.0 Hz, 1H), 4.43–4.36 (m, 3H), 3.26 (t, *J* = 6.0 Hz, 2H), 2.90 (s, 3H), 1.75–1.69 (m, 4H), 1.64–1.58 (m, 1H), 1.56–1.50 (m, 1H), 1.40 (t, *J* = 7.0 Hz, 3H), 1.25–1.16 (m, 3H), 0.98–0.90 (m, 2H); ^{13}C NMR (125 MHz, $CDCl_3$): δ 166.33, 159.31, 156.00, 151.81, 148.37, 138.73, 137.61, 131.50, 126.05, 117.50, 112.63, 61.65, 48.12, 37.76, 31.29, 26.68, 26.07, 24.71, 14.41; HRMS (ESI): *m/z* calcd for $C_{21}H_{28}N_3O_2$ $[M+H]^+$: 354.2182; Found: 354.2184.

Compound 3t'a: ethyl 5-(2-((cyclohexylmethyl)amino)phenyl)-2-methylnicotinate

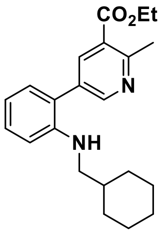

3t'a

White solid; Yield: 60% (42 mg); ^{1}H NMR (400 MHz, $CDCl_3$): δ 8.69 (d, *J* = 2.4 Hz, 1H), 8.30 (d, *J* = 2.4 Hz, 1H), 7.27 (t, *J* = 8.0 Hz, 1H), 7.06 (dd, *J* = 7.6, 1.6 Hz, 1H), 6.77 (t, *J* = 7.2 Hz, 1H), 6.71 (d, *J* = 8.0 Hz, 1H), 4.38 (q, *J* = 7.2 Hz, 2H), 3.76 (t, *J* = 5.6 Hz, 1H), 2.93 (t, *J* = 6.0 Hz, 3H), 2.89 (s, 3H), 1.80–1.65 (m, 5H), 1.58–1.47 (m, 1H), 1.39 (t, *J* = 7.2 Hz, 3H), 1.28–1.12 (m, 3H), 0.99–0.87 (m, 2H); ^{13}C NMR (100 MHz, $CDCl_3$): δ 166.53, 158.65, 152.27, 145.61, 139.21, 132.76, 130.59, 129.89, 125.72, 122.54, 116.98, 110.71, 61.48, 50.56, 37.28, 31.41, 26.64, 26.04, 24.69, 14.39; LRMS (ESI): *m/z* calcd for $C_{22}H_{29}N_2O_2$ $[M+H]^+$: 353.22; Found: 353.2.

Compound 3ua: ethyl 6-methyl-2'-((2-morpholinoethyl)amino)-[3,3'-bipyridine]-5-carboxylate

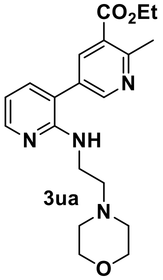

Yellow solid; Yield: 51% (38 mg); ^{1}H NMR (500 MHz, $CDCl_3$): δ 8.66 (d, *J* = 2.5 Hz, 1H), 8.24 (d, *J* = 2.5 Hz, 1H), 8.18 (dd, *J* = 5.0, 2.0 Hz, 1H), 7.29 (dd, *J* = 7.0, 1.5 Hz, 1H), 6.68 (dd, *J* = 7.0, 5.0 Hz, 1H), 5.23 (s, 1H), 4.41 (q, *J* = 7.0 Hz, 2H), 3.55 (t, *J* = 5.0 Hz, 4H), 3.50–3.43 (m, 2H), 2.90 (s, 3H), 2.55 (t, *J* = 6.0 Hz, 2H), 2.40 (t, *J* = 5.0 Hz, 4H), 1.41 (t, *J* = 7.0 Hz, 3H); ^{13}C NMR (125 MHz, $CDCl_3$): δ 166.47, 159.00, 155.85, 151.87, 148.34, 138.63, 137.45, 131.38, 126.01, 117.82, 112.77, 67.05, 61.69, 56.71, 53.15, 37.73, 24.71, 14.38; LRMS (ESI): *m/z* calcd for $C_{20}H_{27}N_4O_3$ $[M+H]^+$: 371.21; Found: 371.2.

Compound 3u'a: ethyl 2-methyl-5-(2-((2-morpholinoethyl)amino)phenyl)nicotinate

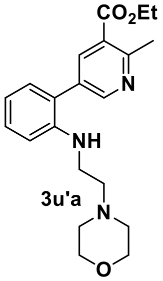

Yellow solid; Yield: 44% (33 mg); ^{1}H NMR (400 MHz, $CDCl_3$): δ 8.68 (d, *J* = 2.4 Hz, 1H), 8.26 (d, *J* = 2.4 Hz, 1H), 7.30 (t, *J* = 8.0 Hz, 2H), 7.09 (d, *J* = 7.2 Hz, 1H), 6.80 (t, *J* = 7.2 Hz, 1H), 6.71 (d, *J* = 8.0 Hz, 1H), 4.60 (s, 1H), 4.39 (q, *J* = 7.2 Hz, 2H), 3.51 (t, *J* = 4.4 Hz, 4H), 3.14 (q, *J* = 5.2 Hz, 2H), 2.89 (s, 3H), 2.56 (t, *J* = 6.0 Hz, 2H), 2.35 (t, *J* = 4.8 Hz, 4H), 1.40 (t, *J* = 7.2 Hz, 3H); ^{13}C NMR (100 MHz, $CDCl_3$): δ 166.70, 158.41, 152.37, 145.76, 139.12, 132.75, 130.42, 129.94, 125.79, 123.08, 117.33, 111.15, 67.02, 61.56, 56.49, 53.09, 40.00, 24.66, 14.41; LRMS (ESI): *m/z* calcd for $C_{21}H_{28}N_3O_3$ $[M+H]^+$: 370.21; Found: 370.2.

Compound 3va: ethyl 6-methyl-2'-(phenylamino)-[3,3'-bipyridine]-5-carboxylate

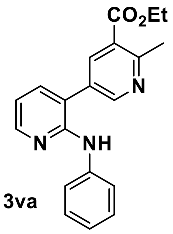

3va

Off-white solid; Yield: 63% (42 mg); ^{1}H NMR (500 MHz, $CDCl_3$): δ 8.70 (d, J = 2.0 Hz, 1H), 8.31–8.25 (m, 2H), 7.47 (d, J = 8.0 Hz, 2H), 7.41 (dd, J = 7.5, 2.0 Hz, 1H), 7.29–7.25 (m, 2H), 6.99 (t, J = 7.5 Hz, 1H), 6.87 (dd, J = 7.5, 5.0 Hz, 1H), 6.38 (s, 1H), 4.40 (q, J = 7.0 Hz, 2H), 2.88 (s, 3H), 1.40 (t, J = 7.0 Hz, 3H); ^{13}C NMR (125 MHz, $CDCl_3$): δ 166.20, 159.59, 153.01, 151.82, 148.15, 140.27, 138.87, 138.51, 130.94, 128.95, 126.21, 122.57, 120.02, 119.16, 115.48, 61.71, 24.67, 14.36; LRMS (ESI): m/z calcd for $C_{20}H_{20}N_3O_2$ $[M+H]^+$: 334.16; Found: 334.2.

Compound 3wa: ethyl 6-methyl-2'-(pyridin-2-ylamino)-[3,3'-bipyridine]-5-carboxylate

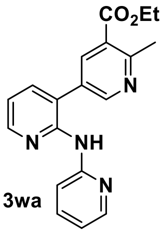

3wa

Yellow solid; Yield: 46% (31 mg); ^{1}H NMR (400 MHz, $CDCl_3$): δ 8.70 (d, J = 2.8 Hz, 1H), 8.43 (d, J = 8.4 Hz, 1H), 8.35 (d, J = 5.2 Hz, 1H), 8.28 (d, J = 2.4 Hz, 1H), 8.14 (d, J = 5.2 Hz, 1H), 7.66 (t, J = 8.0 Hz, 1H), 7.47 (d, J = 7.2 Hz, 1H), 7.12 (s, 1H), 7.01–6.93 (m, 1H), 6.87 (t, J = 6.0 Hz, 1H), 4.39 (q, J = 7.2 Hz, 2H), 2.91 (s, 3H), 1.39 (t, J = 7.2 Hz, 3H); ^{13}C NMR (100 MHz, $CDCl_3$): 166.15, 159.99, 153.29, 151.87, 151.61, 147.84, 139.03, 138.72, 137.97, 130.43, 126.15, 120.18, 117.45, 116.42, 112.67, 61.67, 24.80, 14.35; HRMS (ESI): m/z calcd for $C_{19}H_{19}N_4O_2$ $[M+H]^+$: 335.1508; Found: 335.1508.

Compound 3xa: ethyl 6-methyl-2'-(pyrimidin-2-ylamino)-[3,3'-bipyridine]-5-carboxylate

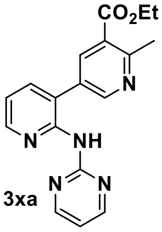

3xa

Yellow solid; Yield: 66% (44 mg); ^{1}H NMR (400 MHz, $CDCl_3$): δ 8.72 (d, J = 2.4 Hz, 1H), 8.53 (dd, J = 4.4, 2.4 Hz, 1H), 8.46–8.07 (m, 4H), 7.67 (dd, J = 7.6, 2.0 Hz, 1H), 7.26–7.18 (m, 1H), 6.71–6.61 (m, 1H), 4.35 (q, J = 7.2 Hz, 2H), 2.80 (s, 3H), 1.36 (t, J = 7.2, Hz, 3H); ^{13}C NMR (100 MHz, $CDCl_3$): 166.19, 159.26, 158.82, 157.94, 151.06, 150.10, 148.52, 139.30, 137.98, 131.84, 125.67, 125.39, 120.15, 113.61, 61.42, 24.49, 14.26; LRMS (ESI): m/z calcd for $C_{18}H_{18}N_5O_2$ $[M+H]^+$: 336.15; Found: 336.05.

Compound 6ab: *N*-(6'-isopropyl-5'-(phenylsulfonyl)-[3,3'-bipyridin]-2-yl)benzenesulfonamide

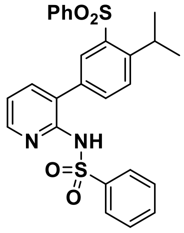

6ab

White solid; Yield: 41% (40 mg); ^{1}H NMR (500 MHz, $CDCl_3$): δ 8.84 (d, *J* = 2.4 Hz, 1H), 8.81 (d, *J* = 2.2 Hz, 1H), 8.08–8.00 (m, 2H), 7.85 (d, *J* = 8.0 Hz, 2H), 7.80–7.71 (m, 2H), 7.62 (t, *J* = 7.5 Hz, 1H), 7.57–7.48 (m, 5H), 6.79 (t, *J* = 6.5 Hz, 1H), 3.79 (hept, *J* = 6.5 Hz, 1H), 1.04 (d, *J* = 6.5 Hz, 6H); ^{13}C NMR (125 MHz, $CDCl_3$): δ 165.67, 152.49, 152.05, 142.78, 141.68, 141.05, 138.92, 135.87, 134.67, 133.72, 132.39, 129.81, 129.54, 129.20, 127.84, 127.80, 126.23, 112.28, 31.99, 21.72; HRMS (ESI+): *m/z* calcd for $C_{25}H_{24}N_3O_4S_2$ $[M+H]^+$: 494.1208; Found: 494.1213.

Compound 6ac: *N*-(6'-phenyl-5'-(phenylsulfonyl)-[3,3'-bipyridin]-2-yl)benzenesulfonamide

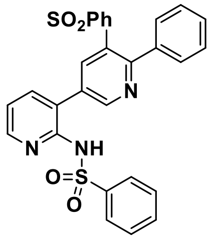

6ac

Yellow solid; Yield: 35% (37 mg); ^{1}H NMR (500 MHz, $CDCl_3$): δ 12.88 (br. s, 1H), 8.91 (d, *J* = 2.5 Hz, 1H), 8.88 (d, *J* = 2.0 Hz, 1H), 8.10–8.04 (m, 2H), 7.84–7.72 (m, 2H), 7.55–7.49 (m, 3H), 7.45–7.41 (m, 1H), 7.38 (t, *J* = 7.5 Hz, 1H), 7.29–7.21 (m, 6H), 7.17–7.14 (m, 2H), 6.79 (t, *J* = 7.0 Hz, 1H); ^{13}C NMR (125 MHz, $CDCl_3$): δ 158.39, 152.43, 152.18, 142.95, 142.06, 139.67, 137.79, 137.55, 136.69, 135.02, 133.25, 132.35, 130.75, 129.69, 129.22, 129.02, 128.67, 128.18, 128.10, 127.72, 126.16, 111.72; LRMS (ESI): *m/z* calcd for $C_{28}H_{22}N_3O_4S_2$ $[M+H]^+$: 528.11; Found: 528.1.

Compound 6a'b: *N*-(2-(6-isopropyl-5-(phenylsulfonyl)pyridin-3-yl)phenyl)benzenesulfonamide

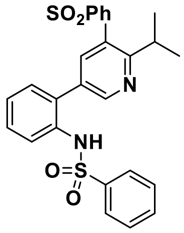

6a'b

Off-white solid; Yield: 43% (42 mg); ^{1}H NMR (400 MHz, $CDCl_3$): δ 8.40 (d, *J* = 2.0 Hz, 1H), 8.23 (d, *J* = 2.4 Hz, 1H), 7.90 (d, *J* = 7.6 Hz, 2H), 7.68–7.49 (m, 7H), 7.44–7.36 (m, 3H), 7.30 (t, *J* = 7.6 Hz, 1H), 7.21 (dd, *J* = 7.6, 1.6 Hz, 1H), 6.69 (br. s, 1H), 3.75 (hept, *J* = 6.8 Hz, 1H), 1.05 (d, *J* = 6.4 Hz, 6H); ^{13}C NMR (100 MHz, $CDCl_3$): δ 165.46, 153.27, 141.12, 138.87, 137.40, 134.76, 133.75, 133.61, 133.50, 131.70, 131.61, 131.00, 130.06, 129.56, 129.35, 127.81, 127.13, 127.01, 125.40, 32.13, 21.90; LRMS (ESI): *m/z* calcd for $C_{26}H_{25}N_2O_4S_2$ $[M+H]^+$: 493.13; Found: 493.1.

Compound 6a'c: *N*-(2-(6-phenyl-5-(phenylsulfonyl)pyridin-3-yl)phenyl)benzenesulfonamide

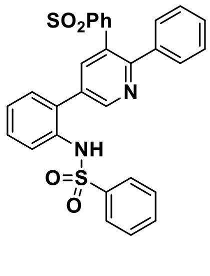

6a'c

Yellow solid; Yield: 36% (38 mg); ^{1}H NMR (500 MHz, $CDCl_3$): δ 8.49–8.44 (m, 2H), 7.64–7.55 (m, 3H), 7.49–7.37 (m, 6H), 7.35 (td, J = 7.5, 1.5 Hz, 1H), 7.30–7.22 (m, 7H), 7.19–7.15 (m, 2H), 4.76 (br. s, 1H); ^{13}C NMR (100 MHz, $CDCl_3$): δ 157.88, 152.45, 139.54, 138.98, 137.50, 137.33, 137.14, 133.59, 133.47, 133.32, 133.19, 131.97, 131.06, 130.24, 129.77, 129.36, 129.11, 128.72, 128.08, 127.78, 127.23, 127.17, 125.94; LRMS (ESI): *m/z* calcd for $C_{19}H_{23}N_2O_4S_2$ $[M+H]^+$: 527.11; Found: 527.1.

Compound 6qb: 6'-isopropyl-*N*-(4-methoxybenzyl)-5'-(phenylsulfonyl)-[3,3'-bipyridin]-2-amine

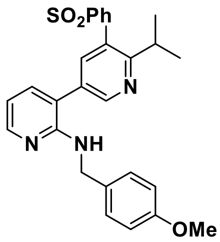

6qb

Yellow gum; Yield: 40% (38 mg); ^{1}H NMR (400 MHz, $CDCl_3$): δ 8.81 (d, J = 2.0 Hz, 1H), 8.61 (d, J = 2.0 Hz, 1H), 8.25 (dd, J = 4.8, 1.6 Hz, 1H), 7.79 (d, J = 8.0 Hz, 2H), 7.61 (t, J = 7.2 Hz, 1H), 7.49 (t, J = 7.6 Hz, 2H), 7.48–7.28 (m, 3H), 6.88 (d, J = 8.4 Hz, 2H), 6.75 (t, J = 6.8 Hz, 1H), 4.59 (s, 3H), 3.81–3.73 (m, 4H), 1.04 (d, J = 6.8 Hz, 6H); ^{13}C NMR (100MHz, $CDCl_3$): 165.50, 159.03, 155.39, 153.51, 148.77, 141.02, 138.02, 137.19, 135.39, 133.74, 131.82, 131.48, 129.49, 129.30, 127.85, 116.88, 114.23, 113.54, 55.41, 45.47, 32.17, 21.92; LRMS (ESI): *m/z* calcd for $C_{27}H_{28}N_3O_3S$ $[M+H]^+$: 474.19; Found: 474.1.

Compound 6qc: *N*-(4-methoxybenzyl)-6'-phenyl-5'-(phenylsulfonyl)-[3,3'-bipyridin]-2-amine

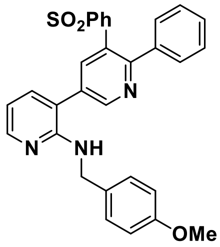

6qc

Yellow gum; Yield: 38% (39 mg); ^{1}H NMR (400 MHz, $CDCl_3$): δ 8.88 (d, J = 2.0 Hz, 1H), 8.84 (d, J = 2.4 Hz, 1H), 8.32–8.24 (m, 1H), 7.47–7.36 (m, 3H), 7.35–7.23 (m, 4H), 7.23–7.10 m, 6H), 6.88 (d, J = 8.0 Hz, 2H), 6.82–6.76 (m, 1H), 4.67 (t, J = 5.2 Hz, 1H), 4.61 (d, J = 5.2 Hz, 2H), 3.77 (s, 3H); ^{13}C NMR (100 MHz, $CDCl_3$): δ 159.04, 158.00, 155.32, 152.58, 149.01, 139.50, 138.12, 137.68, 137.46, 137.17, 133.31, 133.18, 131.38, 129.70, 129.31, 129.09, 128.64, 128.10, 127.76, 116.58, 114.22, 113.59, 55.40, 45.52; HRMS (ESI+): *m/z* calcd for $C_{30}H_{26}N_3O_3S$ $[M+H]^+$: 508.1695; Found: 508.1698.

Compound 6q'b: 2-(6-isopropyl-5-(phenylsulfonyl)pyridin-3-yl)-*N*-(4-methoxybenzyl)aniline

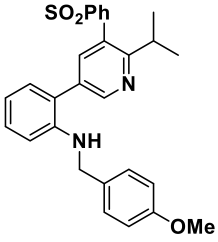

6q'b

Orange solid; Yield: 32% (30 mg); ^{1}H NMR (400 MHz, $CDCl_3$): δ 8.85 (d, *J* = 2.4 Hz, 1H), 8.66 (d, *J* = 2.4 Hz, 1H), 7.80 (d, *J* = 7.6 Hz, 2H), 7.60 (t, *J* = 7.2 Hz, 1H), 7.49 (t, *J* = 7.6 Hz, 2H), 7.34–7.27 (m, 3H), 7.12 (dd, *J* = 7.6, 1.6 Hz, 1H), 6.91–6.82 (m, 3H), 6.77 (d, *J* = 8.4 Hz, 1H), 4.28 (s, 2H), 4.04 (s, 1H), 3.84–3.72 (m, 4H), 1.06 (d, *J* = 6.8 Hz, 6H); ^{13}C NMR (100 MHz, $CDCl_3$): δ 164.80, 159.09, 153.99, 145.28, 141.27, 137.60, 135.04, 133.61, 133.19, 130.89, 130.71, 130.31, 129.44, 128.89, 127.85, 122.20, 118.04, 114.33, 111.63, 55.43, 47.89, 32.10, 21.95; LRMS (ESI): *m/z* calcd for $C_{28}H_{29}N_2O_3S$ $[M+H]^+$: 473.19; Found: 471.1.

Compound 6q'c: *N*-(4-methoxybenzyl)-2-(6-phenyl-5-(phenylsulfonyl)pyridin-3-yl)aniline

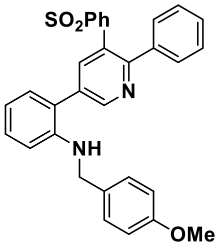

6q'c

Orange solid; Yield: 20% (20 mg); ^{1}H NMR (400 MHz, $CDCl_3$): δ 8.96–8.87 (m, 2H), 7.45–7.36 (m, 2H), 7.35–7.29 m, 3H), 7.26 (d, *J* = 7.6 Hz, 2H), 7.23–7.14 (m, 7H), 6.92–6.86 (m, 3H), 6.82 (d, *J* = 8.4 Hz, 1H), 4.30 (s, 2H), 4.08 (br. s, 1H), 3.78 (s, 3H); ^{13}C NMR (100 MHz, $CDCl_3$): δ 159.15, 157.47, 153.12, 145.28, 139.84, 137.76, 137.58, 137.36, 134.59, 133.22, 130.82, 130.78, 130.58, 129.80, 128.98, 128.63, 128.14, 127.76, 121.93, 118.19, 114.37, 111.79, 55.44, 47.98; LRMS (ESI): *m/z* calcd for $C_{31}H_{27}N_2O_3S$ $[M+H]^+$: 507.17; Found: 507.1.

Compound 6ub: 6'-isopropyl-*N*-(2-morpholinoethyl)-5'-(phenylsulfonyl)-[3,3'-bipyridin]-2-amine

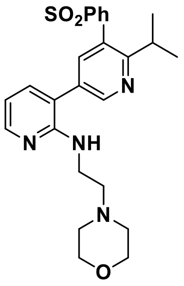

6ub

Yellow gum; Yield: 45% (42 mg); ^{1}H NMR (400 MHz, $CDCl_3$): δ 8.84 (d, *J* = 2.0 Hz, 1H), 8.58 (d, *J* = 2.4 Hz, 1H), 8.20 (dd, *J* = 4.8, 1.6 Hz, 1H), 7.89 (d, *J* = 7.6 Hz, 2H), 7.64 (t, *J* = 7.6 Hz, 1H), 7.56 (t, *J* = 7.6 Hz, 2H), 7.34 (dd, *J* = 7.2, 1.6 Hz, 1H), 6.70 (dd, *J* = 7.2, 4.8 Hz, 1H), 5.35 (t, *J* = 4.4 Hz, 1H), 3.79 (hept, *J* = 6.8 Hz, 1H), 3.58–3.45 (m, 6H), 2.59 (t, *J* = 6.0 Hz, 2H), 2.41 (t, *J* = 4.8 Hz, 4H), 1.07 (d, *J* = 6.8 Hz, 6H); ^{13}C NMR (100 MHz, $CDCl_3$): δ 165.40, 155.76, 153.63, 148.78, 141.22, 137.61, 137.24, 135.33, 133.73, 131.96, 129.46, 127.73, 117.04, 112.91, 67.07, 56.69, 53.15, 37.64, 32.19, 21.88; LRMS (ESI): *m/z* calcd for $C_{25}H_{31}N_4O_3S$ $[M+H]^+$: 467.21; Found: 467.2.

Compound 6uc: *N*-(2-morpholinoethyl)-6'-phenyl-5'-(phenylsulfonyl)-[3,3'-bipyridin]-2-amine

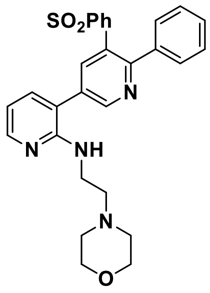
6uc

Yellow gum; Yield: 42% (42 mg); ^{1}H NMR (400 MHz, $CDCl_3$): δ 8.90 (d, *J* = 2.0 Hz, 1H), 8.85 (d, *J* = 2.4 Hz, 1H), 8.23 (d, *J* = 4.8 Hz, 1H), 7.46–7.36 (m, 3H), 7.30–7.16 (m, 8H), 6.74 (dd, *J* = 7.2, 5.2 Hz, 1H), 5.42 (t, *J* = 4.8 Hz, 1H), 3.65–3.48 (m, 6H), 2.62 (t, *J* = 6.0 Hz, 2H), 2.45 (t, *J* = 4.4 Hz, 4H); ^{13}C NMR (100 MHz, $CDCl_3$): δ 157.89, 155.66, 152.71, 149.02, 139.54, 137.72, 137.61, 137.47, 136.95, 133.26, 133.24, 129.64, 129.03, 128.58, 127.97, 127.78, 116.62, 112.95, 67.08, 56.78, 53.19, 37.68; LRMS (ESI): *m/z* calcd for $C_{28}H_{29}N_4O_3S$ $[M+H]^+$: 501.20; Found: 501.2.

Compound 6u'b: 2-(6-isopropyl-5-(phenylsulfonyl)pyridin-3-yl)-*N*-(2-morpholinoethyl)aniline

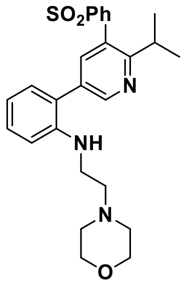
6u'b

Yellow gum; Yield: 42% (39 mg); ^{1}H NMR (400 MHz, $CDCl_3$): δ 8.85 (d, *J* = 2.0 Hz, 1H), 8.60 (d, *J* = 2.0 Hz, 1H), 7.89 (d, *J* = 7.6 Hz, 2H), 7.62 (t, *J* = 7.6 Hz, 1H), 7.54 (t, *J* = 7.6 Hz, 2H), 7.32 (t, *J* = 8.0 Hz, 1H), 7.13 (d, *J* = 7.2 Hz, 1H), 6.82 (t, *J* = 7.2 Hz, 1H), 6.72 (d, *J* = 8.4 Hz, 1H), 4.73 (br. s, 1H), 3.80 (hept, *J* = 6.4 Hz, 1H), 3.48 (t, *J* = 4.4 Hz, 4H), 3.15 (t, *J* = 5.6 Hz, 2H), 2.60 (t, *J* = 5.6 Hz, 2H), 2.37 (t, *J* = 4.8 Hz, 4H), 1.08 (d, *J* = 6.4 Hz, 6H); ^{13}C NMR (100 MHz, $CDCl_3$): δ 164.75, 154.10, 145.80, 141.51, 137.73, 135.11, 133.63, 133.34, 130.51, 130.35, 129.44, 127.78, 122.29, 117.56, 111.47, 67.07, 56.52, 53.11, 39.96, 32.16, 21.97; HRMS (ESI+): *m/z* calcd for $C_{26}H_{32}N_3O_3S$ $[M+H]^+$: 466.2164; Found: 466.2163.

Compound 6u'c: *N*-(2-morpholinoethyl)-2-(6-phenyl-5-(phenylsulfonyl)pyridin-3-yl)aniline

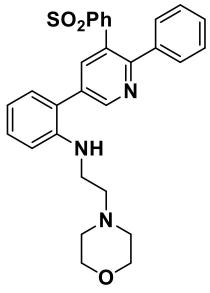
6u'c

Yellow gum; Yield: 20% (20 mg); ^{1}H NMR (400 MHz, $CDCl_3$): δ 8.91 (d, *J* = 2.4 Hz, 1H), 8.88 (d, *J* = 2.4 Hz, 1H), 7.46–7.32 (m, 3H), 7.31–7.25 (m, 4H), 7.24–7.16 (m, 5H), 6.86 (t, *J* = 7.6 Hz, 1H), 6.76 (d, *J* = 8.0 Hz, 1H), 4.81 (br. s, 1H), 3.54 (t, *J* = 4.4 Hz, 4H), 3.19 (t, *J* = 6.0 Hz, 2H), 2.64 (t, *J* = 6.0 Hz, 2H), 2.41 (t, *J* = 4.4 Hz, 4H); ^{13}C NMR (100 MHz, $CDCl_3$): δ 157.39, 153.23, 145.79, 139.93, 137.82, 137.51, 137.39, 134.70, 133.21, 130.62, 130.58, 129.77, 128.97, 128.63, 128.07, 127.82, 121.91, 117.69, 111.61, 67.15, 56.59, 53.17, 39.99; LRMS (ESI): *m/z* calcd for $C_{29}H_{30}N_3O_3S$ $[M+H]^+$: 500.20; Found: 500.1.

Compound 6xb: 6'-isopropyl-5'-(phenylsulfonyl)-*N*-(pyrimidin-2-yl)-[3,3'-bipyridin]-2-amine

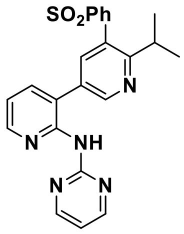

6xb

Yellow solid; Yield: 36% (26 mg); ^{1}H NMR (400 MHz, $CDCl_3$): δ 8.85 (d, *J* = 2.4 Hz, 1H), 8.56 (dd, *J* = 4.8, 2.0 Hz, 1H), 8.52 (d, *J* = 2.0 Hz, 1H), 8.31–8.19 (m, 3H), 7.82–7.70 (m, 3H), 7.61 (t, *J* = 7.6 Hz, 1H), 7.52 (t, *J* = 7.6 Hz, 2H), 7.30–7.24 (m, 1H), 6.60 (t, *J* = 4.8 Hz, 1H), 3.72 (hept, *J* = 6.4 Hz, 1H), 0.99 (d, *J* = 6.4 Hz, 6H); ^{13}C NMR (100 MHz, $CDCl_3$): δ 165.05, 159.00, 157.88, 152.99, 150.13, 148.95, 141.08, 139.33, 136.27, 134.76, 133.59, 132.67, 129.38, 127.80, 125.39, 120.57, 113.60, 32.04, 21.89; LRMS (ESI): *m/z* calcd for $C_{23}H_{22}N_5O_2S$ $[M+H]^+$: 432.15; Found: 432.1.

Compound 6xc: 6'-phenyl-5'-(phenylsulfonyl)-*N*-(pyrimidin-2-yl)-[3,3'-bipyridin]-2-amine

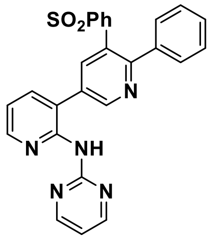

6xc

Off-white solid; Yield: 47% (44 mg); ^{1}H NMR (400 MHz, $CDCl_3$): δ 8.91 (s, 1H), 8.81 (d, *J* = 2.0 Hz, 1H), 8.59 (s, 2H), 8.36–8.20 (m, 2H), 7.83 (d, *J* = 7.6 Hz, 1H), 7.46–7.29 (m, 3H), 7.28–7.15 (m, 6H), 7.11–7.05 (m, 2H), 6.65 (t, *J* = 4.8 Hz, 1H); ^{13}C NMR (100 MHz, $CDCl_3$): δ 159.03, 157.87, 157.41, 151.93, 150.29, 149.10, 139.71, 139.40, 137.57, 136.89, 136.15, 134.32, 133.12, 129.63, 128.90, 128.53, 127.96, 127.63, 125.43, 120.63, 113.47; HRMS (ESI+): *m/z* calcd for $C_{26}H_{20}N_5O_2S$ $[M+H]^+$: 466.1338; Found: 466.1339.

Compound 6x'b: *N*-(2-(6-isopropyl-5-(phenylsulfonyl)pyridin-3-yl)phenyl)pyrimidin-2-amine

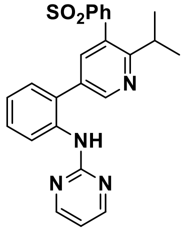

6x'b

Yellow solid; Yield: 50% (43 mg); ^{1}H NMR (400 MHz, $CDCl_3$): δ 8.82 (d, *J* = 2.4 Hz, 1H), 8.52 (d, *J* = 2.0 Hz, 1H), 8.34 (d, *J* = 4.8 Hz, 2H), 8.08 (d, *J* = 8.0 Hz, 1H), 7.87 (d, *J* = 7.6 Hz, 2H), 7.61 (t, *J* = 7.2 Hz, 1H), 7.56–7.46 (m, 3H), 7.37–7.33 (m, 1H), 7.28–7.24 (m, 1H), 6.89 (s, 1H), 6.69 (t, *J* = 4.8 Hz, 1H), 3.80 (hept, *J* = 6.8 Hz, 1H), 1.06 (d, *J* = 6.8 Hz, 6H); ^{13}C NMR (100 MHz, $CDCl_3$): δ 165.12, 160.45, 158.25, 153.75, 141.19, 137.60, 136.47, 135.00, 133.60, 132.80, 130.66, 129.79, 129.44, 129.42, 127.92, 124.98, 123.82, 113.02, 32.12, 21.95; LRMS (ESI): *m/z* calcd for $C_{24}H_{23}N_4O_2S$ $[M+H]^+$: 431.15; Found: 431.1.

Compound 6x'c: *N*-(2-(6-phenyl-5-(phenylsulfonyl)pyridin-3-yl)phenyl)pyrimidin-2-amine

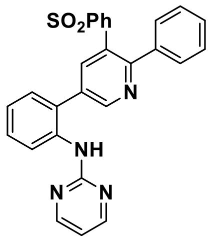

6x'c

Yellow solid; Yield: 52% (48 mg); ^{1}H NMR (400 MHz, $CDCl_3$): δ 8.88 (d, *J* = 2.0 Hz, 1H), 8.78 (d, *J* = 2.4 Hz, 1H), 8.35 (d, *J* = 4.8 Hz, 2H), 8.07 (d, *J* = 8.4 Hz, 1H), 7.51 (t, *J* = 8.0 Hz, 1H), 7.46–7.36 (m, 3H), 7.34–7.31 (m, 1H), 7.30–7.23 (m, 5H), 7.20 (t, *J* = 8.4 Hz, 3H), 7.00 (s, 1H), 6.70 (t, *J* = 4.8 Hz, 1H); ^{13}C NMR (100 MHz, $CDCl_3$): δ 160.53, 158.29, 157.73, 152.90, 139.83, 137.70, 137.53, 137.25, 136.52, 134.24, 133.21, 130.74, 130.07, 129.79, 129.53, 128.98, 128.64, 128.16, 127.71, 125.22, 124.24, 113.04; LRMS (ESI): *m/z* calcd for $C_{27}H_{21}N_4O_2S$ $[M+H]^+$: 465.14; Found: 465.1.

Compound 6yb: 6'-isopropyl-*N*-(4-nitrophenyl)-5'-(phenylsulfonyl)-[3,3'-bipyridin]-2-amine

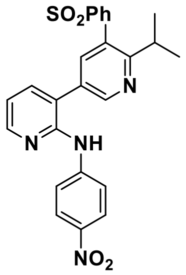

6yb

Yellow solid; Yield: 42% (40 mg); ^{1}H NMR (400 MHz, $CDCl_3$): δ 8.85 (d, *J* = 2.0 Hz, 1H), 8.55 (d, *J* = 2.0 Hz, 1H), 8.40 (d, *J* = 5.2 Hz, 1H), 8.12 (d, *J* = 8.8 Hz, 2H), 7.87 (d, *J* = 7.6 Hz, 2H), 7.68–7.51 (m, 6H), 7.12 (dd, *J* = 8.0, 4.8 Hz, 1H), 6.96 (s, 1H), 3.81 (hept, *J* = 6.8 Hz, 1H), 1.07 (d, *J* = 6.8 Hz, 6H); ^{13}C NMR (100 MHz, $CDCl_3$): δ 166.44, 153.31, 151.03, 148.13, 146.27, 141.72, 140.74, 139.71, 137.29, 135.77, 134.01, 130.58, 129.60, 127.81, 125.31, 120.36, 118.08, 118.00, 32.32, 21.90; LRMS (ESI): *m/z* calcd for $C_{25}H_{23}N_4O_4S$ $[M+H]^+$: 475.14; Found: 475.1.

Compound 6yc: *N*-(4-nitrophenyl)-6'-phenyl-5'-(phenylsulfonyl)-[3,3'-bipyridin]-2-amine

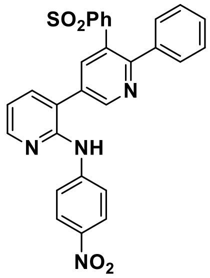

6yc

Yellow solid; Yield: 48% (47 mg); ^{1}H NMR (400 MHz, $CDCl_3$): δ 8.89 (s, 1H), 8.79 (d, *J* = 2.4 Hz, 1H), 8.43 (d, *J* = 5.2 Hz, 1H), 8.13 (d, *J* = 8.8 Hz, 2H), 7.67 (d, *J* = 7.6 Hz, 1H), 7.58 (d, *J* = 8.8 Hz, 2H), 7.49–7.40 (m, 1H), 7.39 (t, *J* = 7.6 Hz, 1H), 7.29–7.20 (m, 6H), 7.19–7.09 (m, 4H); ^{13}C NMR (100 MHz, $CDCl_3$): δ 158.69, 152.38, 151.04, 148.42, 146.35, 141.71, 139.80, 139.19, 137.95, 137.32, 137.13, 133.56, 132.01, 129.60, 129.32, 128.76, 128.04, 127.83, 125.30, 120.13, 118.17, 118.06; LRMS (ESI): *m/z* calcd for $C_{28}H_{21}N_4O_4S$ $[M+H]^+$: 509.13; Found: 509.0.

Compound 6y'b: 2-(6-isopropyl-5-(phenylsulfonyl)pyridin-3-yl)-*N*-(4-nitrophenyl)aniline

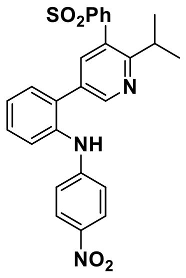

6y'b

Yellow solid; Yield: 50% (47 mg); ^{1}H NMR (400 MHz, $CDCl_3$): δ 8.78 (d, *J* = 2.0 Hz, 1H), 8.50 (d, *J* = 2.0 Hz, 1H), 8.05 (d, *J* = 8.4 Hz, 2H), 7.78–7.70 (m, 2H), 7.60 (t, *J* = 7.6 Hz, 1H), 7.53–7.44 (m, 5H), 7.42–7.36 (m, 1H), 6.74 (d, *J* = 8.8 Hz, 2H), 6.08 (s, 1H), 3.71 (hept, *J* = 6.8 Hz, 1H), 1.00 (d, *J* = 6.8 Hz, 6H); ^{13}C NMR (100 MHz, $CDCl_3$): δ 165.38, 153.24, 150.38, 140.98, 140.08, 137.06, 136.99, 134.95, 133.82, 132.27, 131.89, 131.51, 130.45, 129.45, 127.63, 126.88, 126.23, 125.55, 113.86, 32.15, 21.84; LRMS (ESI): *m/z* calcd for $C_{26}H_{24}N_3O_4S$ $[M+H]^+$: 474.15; Found: 474.1.

Compound 6y'c: *N*-(4-nitrophenyl)-2-(6-phenyl-5-(phenylsulfonyl)pyridin-3-yl)aniline

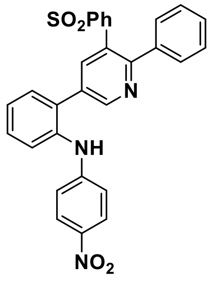

6y'c

Off-white solid; Yield: 48% (50 mg); ^{1}H NMR (400 MHz, $CDCl_3$): δ 8.79 (d, *J* = 2.4 Hz, 1H), 8.76 (d, *J* = 2.0 Hz, 1H), 8.03 (d, *J* = 8.8 Hz, 2H), 7.51 (t, *J* = 7.6 Hz, 2H), 7.45–7.37 (m, 3H), 7.34 (t, *J* = 7.6 Hz, 1H), 7.23–7.13 (m, 4H), 7.12–7.05 (m, 4H), 6.71 (d, *J* = 8.8 Hz, 2H), 6.50 (s, 1H); ^{13}C NMR (100 MHz, $CDCl_3$): δ 157.72, 152.25, 150.55, 139.86, 139.39, 137.27, 137.16, 137.07, 137.01, 133.75, 133.40, 131.73, 131.46, 130.73, 129.59, 129.09, 128.64, 127.85, 127.75, 127.02, 126.20, 125.99, 113.74; HRMS (ESI+): *m/z* calcd for $C_{29}H_{22}N_3O_4S$ $[M+H]^+$: 508.1331; Found: 508.1334.

Compound 6zb: 6'-isopropyl-*N*-(4-methoxyphenyl)-5'-(phenylsulfonyl)-[3,3'-bipyridin]-2-amine

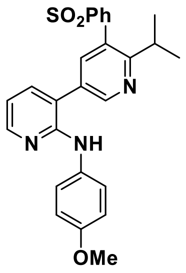

6zb

Yellow solid; Yield: 35% (32 mg); ^{1}H NMR (400 MHz, $CDCl_3$): δ 11.09 (br. s, 1H), 8.47 (d, *J* = 2.0 Hz, 1H), 8.15 (d, *J* = 2.0 Hz, 1H), 8.07 (dd, *J* = 6.0, 1.6 Hz, 1H), 7.83–7.74 (m, 3H), 7.64 (t, *J* = 7.6 Hz, 1H), 7.56 (t, *J* = 7.6 Hz, 2H), 7.06 (t, *J* = 6.8 Hz, 1H), 6.79 (d, *J* = 8.4 Hz, 2H), 6.51 (d, *J* = 8.4 Hz, 2H), 3.63 (s, 3H), 3.57 (hept, *J* = 6.8 Hz, 1H), 0.92 (d, *J* = 6.8 Hz, 6H); ^{13}C NMR (100 MHz, $CDCl_3$): δ 165.93, 157.67, 152.28, 151.71, 146.12, 141.00, 139.43, 136.10, 134.01, 133.82, 129.55, 129.52, 129.36, 127.64, 125.53, 121.94, 114.41, 114.17, 55.30, 32.12, 21.59; HRMS (ESI+): *m/z* calcd for $C_{26}H_{26}N_3O_3S$ $[M+H]^+$: 460.1695; Found: 460.1697.

Compound 6zc: *N*-(4-methoxyphenyl)-6'-phenyl-5'-(phenylsulfonyl)-[3,3'-bipyridin]-2-amine

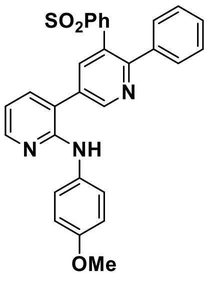

6zc

Yellow solid; Yield: 41% (40 mg); ^{1}H NMR (400 MHz, $CDCl_3$): δ 11.50 (s, 1H), 8.51 (d, *J* = 2.0 Hz, 1H), 8.35 (d, *J* = 2.0 Hz, 1H), 8.09 (d, *J* = 6.0 Hz, 1H), 7.94–7.85 (m, 1H), 7.43 (t, *J* = 7.2 Hz, 1H), 7.37 (t, *J* = 7.6 Hz, 1H), 7.28–7.18 (m, 4H), 7.15–7.07 (m, 3H), 7.02–6.95 (m, 2H), 6.82 (d, *J* = 8.4 Hz, 2H), 6.59 (d, *J* = 8.4 Hz, 2H), 3.70 (s, 3H); ^{13}C NMR (100 MHz, $CDCl_3$): δ 158.33, 157.93, 151.61, 151.03, 146.67, 139.29, 139.14, 137.00, 136.27, 135.89, 133.40, 130.45, 129.49, 129.44, 129.20, 128.67, 128.01, 127.76, 125.83, 121.80, 114.68, 114.10, 55.41; LRMS (ESI): *m/z* calcd for $C_{29}H_{24}N_3O_3S$ $[M+H]^+$: 493.15; Found: 493.1.

Compound 6z'b: 2-(6-isopropyl-5-(phenylsulfonyl)pyridin-3-yl)-*N*-(4-methoxyphenyl)aniline

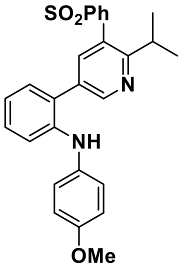

6z'b

Brown gum; Yield: 30% (28 mg); ^{1}H NMR (400 MHz, $CDCl_3$): δ 8.88 (d, *J* = 2.4 Hz, 1H), 8.56 (d, *J* = 2.4 Hz, 1H), 7.82 (d, *J* = 7.6 Hz, 2H), 7.60 (t, *J* = 7.6 Hz, 1H), 7.50 (t, *J* = 7.6 Hz, 2H), 7.31–7.19 (m, 2H), 7.09 (d, *J* = 8.0 Hz, 1H), 7.03–6.91 (m, 3H), 6.85–6.78 (m, 2H), 4.76 (br. s, 1H), 3.79 (hept, *J* = 6.8 Hz, 1H), 3.77 (s, 3H), 1.07 (d, *J* = 6.8 Hz, 6H); ^{13}C NMR (100 MHz, $CDCl_3$): δ 164.72, 155.74, 153.70, 142.80, 141.21, 137.51, 135.56, 135.13, 133.62, 133.24, 131.11, 130.00, 129.44, 127.81, 125.16, 122.83, 120.96, 117.22, 114.85, 55.69, 32.10, 21.92; LRMS (ESI): *m/z* calcd for $C_{27}H_{27}N_2O_3S$ $[M+H]^+$: 459.17; Found: 459.1.

Compound 6z'c: *N*-(4-methoxyphenyl)-2-(6-phenyl-5-(phenylsulfonyl)pyridin-3-yl)aniline

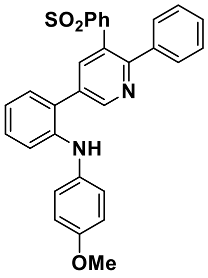

6z'c

Brown gum; Yield: 40% (39 mg); ^{1}H NMR (400 MHz, $CDCl_3$): δ 8.92 (d, *J* = 2.0 Hz, 1H), 8.83 (d, *J* = 2.0 Hz, 1H), 7.43–7.34 (m, 2H), 7.32–7.15 (m, 10H), 7.12 (d, *J* = 8.4 Hz, 1H), 7.03 (t, *J* = 7.2 Hz, 1H), 6.96 (d, *J* = 8.8 Hz, 2H), 6.82 (d, *J* = 8.8 Hz, 2H), 5.08 (br. s, 1H), 3.77 (s, 3H); ^{13}C NMR (100 MHz, $CDCl_3$): δ 157.31, 155.72, 152.85, 142.81, 139.82, 137.73, 137.38, 137.24, 135.56, 134.58, 133.17, 131.17, 130.24, 129.72, 128.92, 128.61, 128.03, 127.69, 124.97, 122.80, 121.12, 117.52, 114.82, 55.66; LRMS (ESI): *m/z* calcd for $C_{30}H_{25}N_2O_3S$ $[M+H]^+$: 493.15; Found: 493.1.

Compound 7ab: diethyl (6-isopropyl-2'-(phenylsulfonamido)-[3,3'-bipyridin]-5-yl)phosphonate

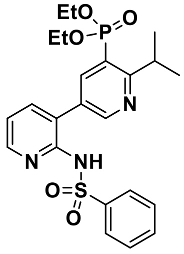

7ab

Off-white solid; Yield: 56% (55 mg); ^{1}H NMR (500 MHz, $CDCl_3$): δ 12.82 (br. s, 1H), 8.78 (s, 1H), 8.33 (d, *J* = 15.0 Hz, 1H), 7.99 (d, *J* = 7.5 Hz, 2H), 7.75 (s, 1H), 7.64 (d, *J* = 7.5 Hz, 1H), 7.57–7.45 (m, 3H), 6.76 (s, 1H), 4.23–4.08 (m, 4H), 3.74 (hept, *J* = 7.0 Hz, 1H), 1.37–1.29 (m, 12H); ^{13}C NMR (125 MHz, $CDCl_3$): δ 170.01 (d, *J* = 13.0 Hz), 152.38, 151.95, 143.28, 142.54 (d, *J* = 10.0 Hz), 141.35, 134.13, 132.18, 128.95, 128.53 (d, *J* = 11.4 Hz), 126.32, 121.63 (d, *J* = 184.1 Hz), 111.92, 62.64 (d, *J* = 5.6 Hz), 33.86, 22.50, 16.46 (d, *J* = 6.6 Hz); ^{31}P NMR (162 MHz, $CDCl_3$) δ 17.41; HRMS (ESI+): *m/z* calcd for $C_{23}H_{29}N_3O_5PS$ $[M+H]^+$: 490.1566; Found: 490.1566.

Compound 7ac: diethyl (6-phenyl-2'-(phenylsulfonamido)-[3,3'-bipyridin]-5-yl)phosphonate

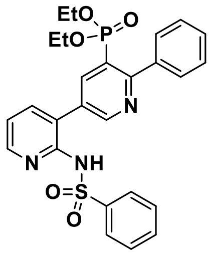

7ac

Off-white solid; Yield: 65% (68 mg); ^{1}H NMR (500 MHz, $CDCl_3$): δ 8.91 (t, *J* = 2.5 Hz, 1H), 8.56 (dd, *J* = 15.0, 2.5 Hz, 1H), 8.02–7.96 (m, 2H), 7.79–7.70 (m, 2H), 7.69–7.62 (m, 2H), 7.56–7.42 (m, 6H), 6.78 (t, *J* = 6.5 Hz, 1H), 4.06–3.94 (m, 2H), 3.93–3.82 (m, 2H), 1.13 (t, *J* = 7.0 Hz, 6H); ^{13}C NMR (125 MHz, $CDCl_3$): δ 161.40 (d, *J* = 11.7 Hz), 151.94, 151.27 (d, *J* = 1.9 Hz), 143.62 (d, *J* = 9.1 Hz), 142.83, 141.76, 139.45, 136.25, 132.26, 130.02 (d, *J* = 11.4 Hz), 129.30, 129.22, 129.04, 127.95, 127.86, 126.23, 123.48 (d, *J* = 187.9 Hz), 112.47, 62.91 (d, *J* = 6.0 Hz), 16.12 (d, *J* = 6.8 Hz); ^{31}P NMR (162 MHz, $CDCl_3$) δ 15.06; LRMS (ESI): *m/z* calcd for $C_{26}H_{27}N_3O_5PS$ $[M+H]^+$: 524.14; Found: 524.2.

Compound 7a'b: diethyl (2-isopropyl-5-(2-(phenylsulfonamido)phenyl)pyridin-3-yl)phosphonate

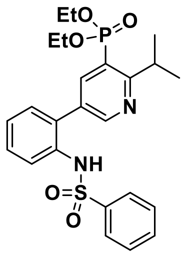

7a'b

Yellow solid; Yield: 47% (46 mg); ^{1}H NMR (400 MHz, $CDCl_3$): δ 8.27 (t, *J* = 2.4 Hz, 1H), 7.92 (dd, *J* = 14.4, 2.4 Hz, 1H), 7.63–7.51 (m, 4H), 7.43–7.34 (m, 3H), 7.23 (t, *J* = 7.6 Hz, 1H), 7.12 (dd, *J* = 8.0, 1.6 Hz, 1H), 6.82–6.73 (m, 1H), 4.28–4.11 (m, 4H), 3.73 (hept, *J* = 6.8 Hz, 1H), 1.41–1.31 (m, 12H); ^{13}C NMR (100 MHz, $CDCl_3$): δ 169.64 (d, *J* = 12.7 Hz), 152.27 (d, *J* = 1.9 Hz), 152.26, 142.13 (d, *J* = 9.7 Hz), 139.05, 133.68, 133.34, 131.47, 130.96, 130.32 (d, *J* = 11.5 Hz), 129.64, 129.22, 127.10, 126.27, 124.04, 122.23 (d, *J* = 185.0 Hz), 62.71 (d, *J* = 5.9 Hz), 33.87, 22.53, 16.50 (d, *J* = 6.6 Hz); ^{31}P NMR (162 MHz, $CDCl_3$) δ 17.00; LRMS (ESI): *m/z* calcd for $C_{24}H_{30}N_2O_5PS$ $[M+H]^+$: 489.16; Found: 489.1.

Compound 7a'c: diethyl (2-phenyl-5-(2-(phenylsulfonamido)phenyl)pyridin-3-yl)phosphonate

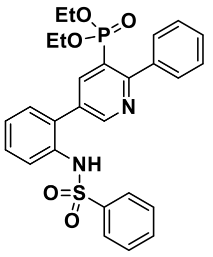

7a'c

Yellow solid; Yield: 50% (50 mg); ^{1}H NMR (500 MHz, $CDCl_3$): 8.48 (s, 1H), 8.21 (dd, *J* = 15.0, 2.0 Hz, 1H), 7.72–7.66 (m, 2H), 7.62–7.56 (m, 3H), 7.53 (t, *J* = 7.5 Hz, 1H), 7.48–7.41 (m, 4H), 7.40–7.34 (m, 3H), 7.29 (t, *J* = 7.5 Hz, 1H), 7.25–7.20 (m, 1H), 4.04–3.96 (m, 2H), 3.94–3.85 (m, 2H), 1.15 (t, *J* = 7.0 Hz, 6H); ^{13}C NMR (125 MHz, $CDCl_3$): δ 160.85, 151.18, 143.60, 139.53, 139.39, 133.75, 133.23, 132.29 (d, *J* = 10.8 Hz), 132.09, 130.95, 129.94, 129.39, 129.22, 129.16, 128.00, 127.16, 126.82, 125.49, 124.05 (d, *J* = 187.1 Hz), 62.84 (d, *J* = 6.1 Hz), 16.18 (d, *J* = 6.8 Hz); ^{31}P NMR (162 MHz, $CDCl_3$) δ 15.16LRMS (ESI): *m/z* calcd for $C_{27}H_{28}N_2O_5PS$ $[M+H]^+$: 523.15; Found: 523.1.

Compound 7qb: diethyl (6-isopropyl-2'-((4-methoxybenzyl)amino)-[3,3'-bipyridin]-5-yl)phosphonate

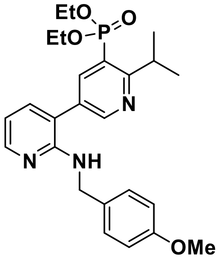

7qb

Yellow gum; Yield: 57% (54 mg); ^{1}H NMR (400 MHz, $CDCl_3$): δ 8.77 (t, *J* = 2.4 Hz, 1H), 8.30–8.18 (m, 2H), 7.30 (dd, *J* = 7.2, 2.0 Hz, 1H), 7.26 (d, *J* = 8.4 Hz, 2H), 6.84 (d, *J* = 8.4 Hz, 2H), 6.71 (dd, *J* = 7.2, 5.2 Hz, 1H), 4.64–4.54 (m, 3H), 4.22–4.07 (m, 4H), 3.77 (s, 3H), 3.75–3.68 (m, 1H), 1.38–1.30 (m, 12H); ^{13}C NMR (100 MHz, $CDCl_3$): δ 169.55 (d, *J* = 13.0 Hz), 158.91, 155.49, 152.51 (d, *J* = 1.9 Hz), 148.28, 142.07 (d, *J* = 9.4 Hz), 137.89, 131.65, 130.70 (d, *J* = 11.2 Hz), 129.10, 122.66 (d, *J* = 184.5 Hz), 117.75, 114.06, 113.32, 62.62 (d, *J* = 5.8 Hz), 55.38, 45.25, 33.90, 22.52, 16.47 (d, *J* = 6.6 Hz); ^{31}P NMR (162 MHz, $CDCl_3$) δ 17.06; LRMS (ESI): *m/z* calcd for $C_{25}H_{33}N_3O_4P$ $[M+H]^+$: 470.22; Found: 470.3.

Compound 7qc: diethyl (2'-((4-methoxybenzyl)amino)-6-phenyl-[3,3'-bipyridin]-5-yl)phosphonate

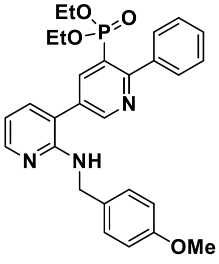

7qc

Yellow gum; Yield: 62% (63 mg); ^{1}H NMR (400 MHz, $CDCl_3$): δ 8.87 (t, *J* = 2.4 Hz, 1H), 8.40 (dd, *J* = 14.8, 2.4 Hz, 1H), 8.24 (dd, *J* = 4.8, 1.6 Hz, 1H), 7.75–7.65 (m, 2H), 7.52–7.42 (m, 3H), 7.37 (dd, *J* = 7.2, 2.0 Hz, 1H), 7.31–7.22 (m, 2H), 6.84 (d, *J* = 8.2 Hz, 2H), 6.75 (dd, *J* = 7.2, 5.2 Hz, 1H), 4.66 (t, *J* = 5.2 Hz, 1H), 4.59 (d, *J* = 5.2 Hz, 2H), 4.02–3.79 (m, 4H), 3.77 (s, 3H), 1.11 (t, *J* = 7.2 Hz, 6H); ^{13}C NMR (100 MHz, $CDCl_3$): δ 161.51 (d, *J* = 11.3 Hz), 158.92, 155.42, 151.90 (d, *J* = 1.7 Hz), 148.55, 142.64 (d, *J* = 8.4 Hz), 140.32, 137.96, 131.77 (d, *J* = 10.7 Hz), 131.51, 129.28, 129.16, 128.98, 127.93, 124.22 (d, *J* = 186.7 Hz), 117.40, 114.09, 113.38, 62.61 (d, *J* = 6.1 Hz), 55.36, 45.32, 16.14

(d, J = 6.6 Hz); ^{31}P NMR (162 MHz, $CDCl_3$) δ 15.34; HRMS (ESI+): *m/z* calcd for $C_{28}H_{31}N_3O_4P$ $[M+H]^+$: 504.2052; Found: 504.2055.

Compound 7q'b: diethyl (2-isopropyl-5-(2-((4-methoxybenzyl)amino)phenyl)pyridin-3-yl)phosphonate

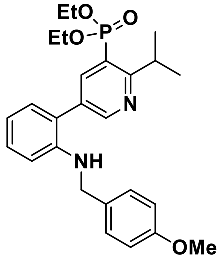

7q'b

Colorless gum; Yield: 54% (51 mg); ^{1}H NMR (400 MHz, $CDCl_3$): δ 8.82 (t, J = 2.4 Hz, 1H), 8.30 (dd, J = 14.8, 2.4 Hz, 1H), 7.28–7.23 (m, 3H), 7.08 (d, J = 7.6 Hz, 1H), 6.88–6.78 (m, 3H), 6.71 (d, J = 8.4 Hz, 1H), 4.26 (s, 2H), 4.22–4.04 (m, 5H), 3.82–3.70 (m, 4H), 1.37–1.30 (m, 12H); ^{13}C NMR (100 MHz, $CDCl_3$): δ 168.92 (d, J = 13.2 Hz), 158.98, 153.03, 145.31, 142.44 (d, J = 9.1 Hz), 132.10 (d, J = 11.2 Hz), 131.08, 130.70, 129.84, 128.65, 123.07, 122.23 (d. J = 184.4 Hz), 117.74, 114.19, 111.31, 62.57 (d, J = 4.9 Hz), 55.37, 47.76, 33.86, 22.58, 16.50 (d, J = 5.1 Hz); ^{31}P NMR (162 MHz, $CDCl_3$) δ 17.44; LRMS (ESI): *m/z* calcd for $C_{26}H_{34}N_2O_4P$ $[M+H]^+$: 469.23; Found: 469.2.

Compound 7q'c: diethyl (5-(2-((4-methoxybenzyl)amino)phenyl)-2-phenylpyridin-3-yl)phosphonate

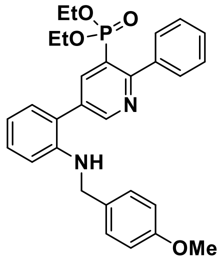

7q'c

Yellow solid; Yield: 30% (30 mg); ^{1}H NMR (400 MHz, $CDCl_3$): 8.91 (t, J = 2.4 Hz, 1H), 8.45 (dd, J = 14.8, 2.4 Hz, 1H), 7.76–7.66 (m, 2H), 7.49–7.41 (m, 3H), 7.32–7.23 (m, 3H), 7.15 (d, J = 7.2 Hz, 1H), 6.90–6.81 (m, 3H), 6.76 (d, J = 8.0 Hz, 1H), 4.28 (s, 2H), 4.11 (br. s, 1H), 4.02–3.83 (m, 4H), 3.78 (s, 3H), 1.12 (t, J = 7.2 Hz, 6H); ^{13}C NMR (100 MHz, $CDCl_3$): δ 161.01 (d, J = 11.3 Hz), 159.01, 152.45 (d, J = 1.9 Hz), 145.30, 142.96 (d, J = 8.4 Hz), 140.59, 133.19 (d, J = 10.9 Hz), 130.94, 130.73, 130.10, 129.38, 128.87, 128.70, 127.92, 123.90 (d, J = 186.6 Hz), 122.74, 117.86, 114.23, 111.44, 62.56 (d, J = 6.2 Hz), 55.39, 47.81, 16.18 (d, J = 6.5 Hz); ^{31}P NMR (162 MHz, $CDCl_3$) δ 15.71; LRMS (ESI): *m/z* calcd for $C_{29}H_{32}N_2O_4P$ $[M+H]^+$: 503.21; Found: 503.1.

Compound 7ub: diethyl (6-isopropyl-2'-((2-morpholinoethyl)amino)-[3,3'-bipyridin]-5-yl)phosphonate

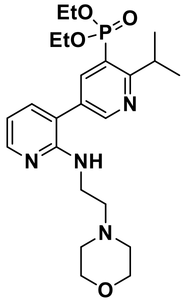

7ub

Yellow gum; Yield: 52% (48 mg); ^{1}H NMR (400 MHz, $CDCl_3$): δ 8.78 (t, *J* = 2.4 Hz, 1H), 8.27–8.12 (m, 2H), 7.35–7.22 (m, 1H), 6.67 (dd, *J* = 7.2, 4.8 Hz, 1H), 5.28 (t, *J* = 4.8 Hz, 1H), 4.31–4.09 (m, 4H), 3.76 (hept, *J* = 6.8 Hz, 1H), 3.60–3.38 (m, 6H), 2.56 (t, *J* = 6.0 Hz, 2H), 2.39 (t, *J* = 4.8 Hz, 4H), 1.43–1.32 (m, 12H); ^{13}C NMR (100 MHz, $CDCl_3$): δ 169.42 (d, *J* = 12.8 Hz), 155.87, 152.59 (d, *J* = 1.9 Hz), 148.24, 142.15 (d, *J* = 9.5 Hz), 137.48, 130.78 (d, *J* = 11.4 Hz), 122.59 (d, *J* = 185.4 Hz), 118.02, 112.73, 67.00, 62.54 (d, *J* = 6.0 Hz), 56.61, 53.09, 37.63, 33.88, 22.48, 16.43 (d, *J* = 6.5 Hz); ^{31}P NMR (162 MHz, $CDCl_3$) δ 17.31; LRMS (ESI): *m/z* calcd for $C_{23}H_{36}N_4O_4P$ $[M+H]^+$: 463.25; Found: 463.2.

Compound 7uc: diethyl (2'-((2-morpholinoethyl)amino)-6-phenyl-[3,3'-bipyridin]-5-yl)phosphonate

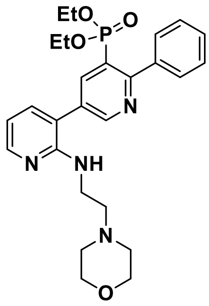

7uc

Yellow gum; Yield: 56% (56 mg); ^{1}H NMR (400 MHz, $CDCl_3$): δ 8.87 (t, *J* = 2.4 Hz, 1H), 8.40 (dd, *J* = 14.8, 2.4 Hz, 1H), 8.20 (dd, *J* = 4.8, 1.6 Hz, 1H), 7.77–7.66 (m, 2H), 7.52–7.42 (m, 3H), 7.35 (dd, *J* = 7.2, 1.6 Hz, 1H), 6.71 (dd, *J* = 7.2, 4.8 Hz, 1H), 5.29 (t, *J* = 4.4 Hz, 1H), 4.07–3.83 (m, 4H), 3.57 (t, *J* = 4.4 Hz, 4H), 3.50 (q, *J* = 5.6 Hz, 2H), 2.58 (t, *J* = 6.0 Hz, 2H), 2.42 (t, *J* = 4.4 Hz, 4H), 1.14 (t, *J* = 7.2 Hz, 6H); ^{13}C NMR (100 MHz, $CDCl_3$): δ 161.57 (d, *J* = 11.3 Hz), 155.89, 152.04 (d, *J* = 1.9 Hz), 148.60, 142.74 (d, *J* = 8.6 Hz), 140.46, 137.63, 131.97 (d, *J* = 11.0 Hz), 129.29, 128.99, 127.98, 124.21 (d, *J* = 187.2 Hz), 117.67, 112.89, 67.13, 62.62 (d, *J* = 6.3 Hz), 56.81, 53.21, 37.81, 16.18 (d, *J* = 6.6 Hz); ^{31}P NMR (162 MHz, $CDCl_3$) δ 15.48; LRMS (ESI): *m/z* calcd for $C_{26}H_{34}N_4O_4P$ $[M+H]^+$: 497.23; Found: 497.2.

Compound 7u'b: diethyl (2-isopropyl-5-(2-((2-morpholinoethyl)amino)phenyl)pyridin-3-yl)phosphonate

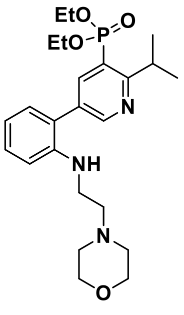

7u'b

Colorless gum; Yield: 38% (35 mg); ^{1}H NMR (400 MHz, $CDCl_3$): δ 8.80 (t, *J* = 2.4 Hz, 1H), 8.22 (dd, *J* = 14.8, 2.4 Hz, 1H), 7.31–7.26 (m, 1H), 7.08 (dd, *J* = 7.6, 1.6 Hz, 1H), 6.79 (t, *J* = 7.2 Hz, 1H), 6.70 (d, *J* = 8.0 Hz, 1H), 4.66 (s, 1H), 4.29–4.12 (m, 4H), 3.77 (p, *J* = 6.8 Hz, 1H), 3.50 (t, *J* = 4.4 Hz, 4H), 3.14 (t, *J* = 6.0 Hz, 2H), 2.57 (t, *J* = 6.0 Hz, 2H), 2.34 (t, *J* = 4.4 Hz, 4H), 1.41–1.32 (m, 12H); ^{13}C NMR (100 MHz, $CDCl_3$): δ 168.84 (d, *J* = 13.0 Hz), 153.07 (d, *J* = 1.9 Hz), 145.84, 142.52 (d, *J* = 9.4 Hz), 132.17 (d, *J* = 11.4 Hz), 130.53, 129.84, 123.34, 122.32 (d, *J* = 185.2 Hz), 117.35, 111.21, 67.02, 62.52 (d, *J* = 5.9 Hz), 56.43, 53.06, 40.05, 33.89, 22.58, 16.50 (d, *J* = 6.4 Hz); ^{31}P NMR (162 MHz, $CDCl_3$) δ 17.71; HRMS (ESI+): *m/z* calcd for $C_{24}H_{37}N_3O_4P$ $[M+H]^+$: 462.2522; Found: 462.2525.

Compound 7u'c: diethyl (5-(2-((2-morpholinoethyl)amino)phenyl)-2-phenylpyridin-3-yl)phosphonate

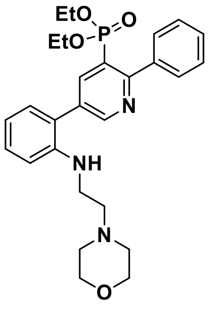

7u'c

Yellow gum; Yield: 50% (49 mg); ^{1}H NMR (400 MHz, $CDCl_3$): 8.88 (t, *J* = 2.4 Hz, 1H), 8.41 (dd, *J* = 14.8, 2.4 Hz, 1H), 7.72 (dd, *J* = 7.6, 2.4 Hz, 2H), 7.50–7.44 (m, 3H), 7.32 (td, *J* = 8.0, 1.6 Hz, 1H), 7.15 (dd, *J* = 7.6, 1.6 Hz, 1H), 6.83 (t, *J* = 7.2 Hz, 1H), 6.73 (d, *J* = 8.4 Hz, 1H), 4.70 (s, 1H), 4.05–3.94 (m, 2H), 3.92–3.83 (m, 2H), 3.53 (t, *J* = 4.4 Hz, 4H), 3.16 (t, *J* = 6.0 Hz, 2H), 2.59 (t, *J* = 6.0 Hz, 2H), 2.38 (t, *J* = 4.8 Hz, 4H), 1.14 (t, *J* = 7.2 Hz, 6H); ^{13}C NMR (100 MHz, $CDCl_3$): δ 161.01 (d, *J* = 11.5 Hz), 152.52 (d, *J* = 1.8 Hz), 145.84, 143.02 (d, *J* = 8.5 Hz), 140.68, 133.29 (d, *J* = 11.0 Hz), 130.53, 130.13, 129.34, 128.86, 127.97, 123.86 (d, *J* = 187.4 Hz), 122.92, 117.49, 111.33, 67.09, 62.54 (d, *J* = 6.2 Hz), 56.55, 53.12, 40.11, 16.20 (d, *J* = 6.6 Hz); ^{31}P NMR (162 MHz, $CDCl_3$) δ 15.85; LRMS (ESI): *m/z* calcd for $C_{27}H_{35}N_3O_4P$ $[M+H]^+$: 496.24; Found: 496.2.

Compound 7xb: diethyl (6-isopropyl-2'-(pyrimidin-2-ylamino)-[3,3'-bipyridin]-5-yl)phosphonate

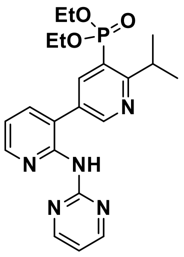

7xb

Yellow solid; Yield: 47% (41 mg); ^{1}H NMR (400 MHz, $CDCl_3$): δ 8.82 (t, *J* = 2.4 Hz, 1H), 8.53 (d, *J* = 5.2 Hz, 1H), 8.28 (d, *J* = 4.8 Hz, 2H), 8.23 (dd, *J* = 14.8, 2.4 Hz, 1H), 8.10 (s, 1H), 7.69 (dd, *J* = 7.2, 1.6 Hz, 1H), 7.25–7.18 (m, 1H), 6.66 (t, *J* = 4.8 Hz, 1H), 4.27–3.93 (m, 4H), 3.71 (hept, *J* = 6.8 Hz, 1H), 1.36–1.24 (m, 12H); ^{13}C NMR (100 MHz, $CDCl_3$): δ 169.37 (d, *J* = 13.0 Hz), 159.14, 157.91, 152.11 (d, *J* = 1.8 Hz), 150.07, 148.57, 141.24 (d, *J* = 9.5 Hz), 139.30, 131.19 (d, *J* = 11.4 Hz), 125.52, 121.91 (d, *J* = 184.7 Hz), 120.16, 113.58, 62.52 (d, *J* = 5.8 Hz), 33.76, 22.48, 16.41 (d, *J* = 6.7 Hz); ^{31}P NMR (162 MHz, $CDCl_3$) δ 17.07; LRMS (ESI): *m/z* calcd for $C_{21}H_{27}N_5O_3P$ $[M+H]^+$: 428.19; Found: 428.1.

Compound 7xc: diethyl (6-phenyl-2'-(pyrimidin-2-ylamino)-[3,3'-bipyridin]-5-yl)phosphonate

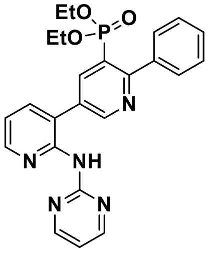

7xc

Off-white solid; Yield: 52% (48 mg); ^{1}H NMR (400 MHz, $CDCl_3$): δ 8.90 (t, *J* = 2.4 Hz, 1H), 8.56 (d, *J* = 4.8 Hz, 1H), 8.41 (dd, *J* = 14.8, 2.4 Hz, 1H), 8.35–8.23 (m, 3H), 7.75 (dd, *J* = 7.2, 1.6 Hz, 1H), 7.68–7.59 (m, 2H), 7.49–7.38 (m, 3H), 7.31–7.20 m, 1H), 6.67 (t, *J* = 4.8 Hz, 1H), 4.06–3.72 (m, 4H), 1.10 (t, *J* = 7.2 Hz, 6H); ^{13}C NMR (100 MHz, $CDCl_3$): δ 161.23 (d, *J* = 11.2 Hz), 159.17, 157.97, 151.36 (d, *J* = 1.8 Hz), 150.13, 148.81, 141.78 (d, *J* = 8.6 Hz), 140.32, 139.38, 132.44 (d, *J* = 11.1 Hz), 129.26, 128.89, 127.85, 125.48, 123.40 (d, *J* = 186.4 Hz), 120.30, 113.69, 62.51 (d, *J* = 6.1 Hz), 16.10 (d, *J* = 6.6 Hz); ^{31}P NMR (162 MHz, $CDCl_3$) δ 15.40; LRMS (ESI): *m/z* calcd for $C_{24}H_{25}N_5O_3P$ $[M+H]^+$: 462.17; Found: 462.1.

Compound 7x'b: diethyl (2-isopropyl-5-(2-(pyrimidin-2-ylamino)phenyl)pyridin-3-yl)phosphonate

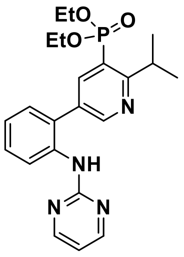

7x'b

Yellow solid; Yield: 66% (56 mg); ^{1}H NMR (400 MHz, $CDCl_3$): δ 8.79 (t, *J* = 2.4 Hz, 1H), 8.32 (d, *J* = 4.8 Hz, 2H), 8.22 (dd, *J* = 14.8, 2.4 Hz, 1H), 8.12 (d, *J* = 8.4 Hz, 1H), 7.44 (t, *J* = 7.6 Hz, 1H), 7.30 (d, *J* = 6.8 Hz, 1H), 7.22 (t, *J* = 7.6 Hz, 1H), 6.99 (s, 1H), 6.68 (t, *J* = 4.8 Hz, 1H), 4.26–4.04 (m, 4H), 3.76 (hept, *J* = 6.0 Hz, 1H), 1.39–1.27 (m, 12H); ^{13}C NMR (100 MHz, $CDCl_3$): δ 169.21 (d, *J* = 13.2 Hz), 160.36, 158.09, 152.79 (d, *J* = 1.8 Hz), 142.33 (d, *J* = 9.4 Hz), 136.41, 131.57 (d, *J* = 11.0 Hz), 130.67, 129.65, 129.23, 124.42, 123.06, 122.09 (d, *J* = 184.4 Hz), 112.76, 62.50 (d, *J* = 5.8 Hz), 33.78,

22.51, 16.38 (d, *J* = 6.6 Hz); ^{31}P NMR (162 MHz, $CDCl_3$) δ 17.23; LRMS (ESI): *m/z* calcd for $C_{22}H_{28}N_4O_3P$ $[M+H]^+$: 427.19; Found: 427.1

Compound 7x'c: diethyl (2-phenyl-5-(2-(pyrimidin-2-ylamino)phenyl)pyridin-3-yl)phosphonate

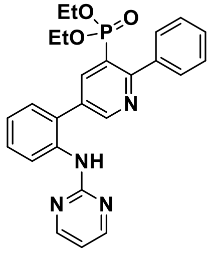

7x'c

Off-white solid; Yield: 52% (48 mg); ^{1}H NMR (400 MHz, $CDCl_3$): 8.87 (t, *J* = 2.4 Hz, 1H), 8.39 (dd, *J* = 14.8, 2.4 Hz, 1H), 8.34 (d, *J* = 5.2 Hz, 2H), 8.14 (d, *J* = 8.0 Hz, 1H), 7.77–7.65 (m, 2H), 7.51–7.41 (m, 4H), 7.36 (d, *J* = 8.0 Hz, 1H), 7.26 (d, *J* = 7.6 Hz, 1H), 7.02 (s, 1H), 6.69 (t, *J* = 4.8 Hz, 1H), 4.03–3.78 (m, 4H), 1.10 (t, *J* = 7.2 Hz, 6H); ^{13}C NMR (100 MHz, $CDCl_3$): δ 161.26 (d, *J* = 11.3 Hz), 160.42, 158.15, 152.20 (d, *J* = 1.8 Hz), 142.87 (d, *J* = 8.4 Hz), 140.46, 136.50, 132.69 (d, *J* = 11.0 Hz), 130.69, 129.54, 129.49, 129.35, 128.84, 127.85, 124.59, 123.66 (d, *J* = 185.1 Hz), 123.30, 112.88, 62.52 (d, *J* = 6.2 Hz), 16.09 (d, *J* = 6.7 Hz); ^{31}P NMR (162 MHz, $CDCl_3$) δ 15.50; HRMS (ESI+): *m/z* calcd for $C_{25}H_{26}N_4O_3P$ $[M+H]^+$: 461.1743; Found: 461.1746.

Compound 7yb: diethyl (6-isopropyl-2'-((4-nitrophenyl)amino)-[3,3'-bipyridin]-5-yl)phosphonate

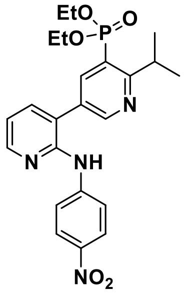

7yb

Yellow solid; Yield: 60% (56 mg); ^{1}H NMR (400 MHz, $CDCl_3$): δ 8.80 (t, *J* = 2.8 Hz, 1H), 8.39 (d, *J* = 4.8 Hz, 1H), 8.30 (dd, *J* = 14.8, 2.4 Hz, 1H), 8.12 (d, *J* = 8.8 Hz, 2H), 7.62 (d, *J* = 8.8 Hz, 2H), 7.54 (d, *J* = 7.2 Hz, 1H), 7.11–7.05 (m, 1H), 7.01 (s, 1H), 4.31–4.11 (m, 4H), 3.78 (hept, *J* = 6.8 Hz, 1H), 1.44–1.29 (m, 12H); ^{13}C NMR (100 MHz, $CDCl_3$): δ 170.08 (d, *J* = 13.4 Hz), 151.88, 151.15, 147.97, 146.49, 142.75, 141.54, 139.43, 129.83 (d, *J* = 11.3 Hz), 125.26, 123.49 (d, *J* = 185.8 Hz), 120.84, 117.88, 62.90 (d, *J* = 6.0 Hz), 33.94, 22.44, 16.47 (d, *J* = 6.4 Hz); ^{31}P NMR (162 MHz, $CDCl_3$) δ 16.15; LRMS (ESI): *m/z* calcd for $C_{23}H_{28}N_4O_5P$ $[M+H]^+$: 471.18; Found: 471.1.

Compound 7yc: diethyl (2'-((4-nitrophenyl)amino)-6-phenyl-[3,3'-bipyridin]-5-yl)phosphonate

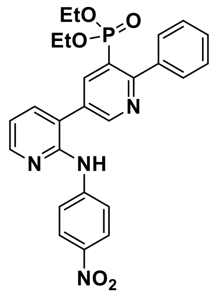

7yc

Yellow solid; Yield: 57% (58 mg); ^{1}H NMR (500 MHz, $CDCl_3$): δ 8.84 (t, *J* = 2.5 Hz, 1H), 8.47–8.37 (m, 2H), 8.14 (d, *J* = 9.0 Hz, 2H), 7.73–7.66 (m, 2H), 7.64 (d, *J* = 9.0 Hz, 2H), 7.60 (dd, *J* = 7.5, 2.0 Hz, 1H), 7.49–7.40 (m, 3H), 7.28 (br. s, 1H), 7.10 (dd, *J* = 7.5, 5.0 Hz, 1H), 4.06–3.80 (m, 4H), 1.11 (t, *J* = 7.0 Hz, 6H); ^{13}C NMR (125 MHz, $CDCl_3$): δ 161.91 (d, *J* = 11.3 Hz), 151.43, 151.22, 148.09, 146.65, 143.09, 141.49, 139.63, 139.44, 130.85 (d, *J* = 11.3 Hz), 129.33, 129.27, 127.99, 125.27, 124.67 (d, *J* = 187.6 Hz), 120.73, 117.91, 117.85, 62.88 (d, *J* = 6.1 Hz), 16.10 (d, *J* = 6.8 Hz); ^{31}P NMR (162 MHz, $CDCl_3$) δ 14.62; LRMS (ESI): *m/z* calcd for $C_{26}H_{26}N_4O_5P$ $[M+H]^+$: 505.16; Found: 505.1.

Compound 7y'b: diethyl (2-isopropyl-5-(2-((4-nitrophenyl)amino)phenyl)pyridin-3-yl)phosphonate

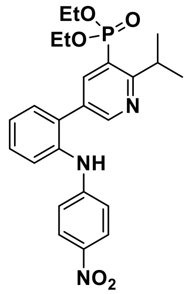

7y'b

Yellow solid; Yield: 42% (40 mg); ^{1}H NMR (400 MHz, $CDCl_3$): δ 8.80 (t, *J* = 2.4 Hz, 1H), 8.24 (dd, *J* = 14.8, 2.4 Hz, 1H), 7.99 (d, *J* = 8.8 Hz, 2H), 7.50–7.39 (m, 3H), 7.35 (t, *J* = 7.2 Hz, 1H), 6.72 (d, *J* = 8.8 Hz, 2H), 6.61 (s, 1H), 4.19–3.97 (m, 4H), 3.67 (hept, *J* = 6.8 Hz, 1H), 1.32–1.22 (m, 12H); ^{13}C NMR (100 MHz, $CDCl_3$): δ 168.70 (d, *J* = 13.6 Hz), 151.59, 150.83, 142.83 (d, *J* = 9.5 Hz), 139.66, 137.13, 132.31, 131.83 (d, *J* = 11.4 Hz), 131.36, 130.13, 126.64, 126.09, 125.58, 122.46 (d, *J* = 186.0 Hz), 113.69, 62.83 (d, *J* = 5.8 Hz), 33.65, 22.24, 16.34 (d, *J* = 6.5 Hz); ^{31}P NMR (162 MHz, $CDCl_3$) δ 16.14; LRMS (ESI): *m/z* calcd for $C_{24}H_{29}N_3O_5P$ $[M+H]^+$: 470.18; Found: 470.1.

Compound 7y'c: diethyl (5-(2-((4-nitrophenyl)amino)phenyl)-2-phenylpyridin-3-yl)phosphonate

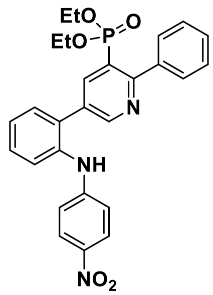

7y'c

Yellow solid; gum: 49% (49 mg); ^{1}H NMR (400 MHz, $CDCl_3$): 8.72 (s, 1H), 8.33 (dd, *J* = 14.8, 2.0 Hz, 1H), 8.01 (d, *J* = 8.4 Hz, 2H), 7.54 (d, *J* = 7.2 Hz, 2H), 7.49–7.32 (m, 7H), 7.16 (s, 1H), 6.76 (d, *J* = 8.8 Hz, 2H), 3.92–3.80 (m, 2H), 3.80–3.66 (m, 2H), 1.03 (t, *J* = 7.2 Hz, 6H); ^{13}C NMR (100 MHz, $CDCl_3$): δ 160.71 (d, *J* = 11.5 Hz), 151.18, 151.09, 143.20 (d, *J* = 8.6 Hz), 139.51, 139.44, 137.42, 132.92 (d, *J* = 11.1 Hz), 132.15, 131.29, 130.22, 129.18, 127.92, 126.51, 126.21, 125.57, 123.89 (d, *J* = 187.3 Hz), 113.53, 62.71 (d, *J* = 6.2 Hz), 16.03 (d, *J* = 6.6 Hz); ^{31}P NMR (162 MHz, $CDCl_3$) δ 14.92; HRMS (ESI+): *m/z* calcd for $C_{27}H_{27}N_3O_5P$ $[M+H]^+$: 504.1688; Found: 504.1692.

Compound 7zb: diethyl (6-isopropyl-2'-((4-methoxyphenyl)amino)-[3,3'-bipyridin]-5-yl)phosphonate

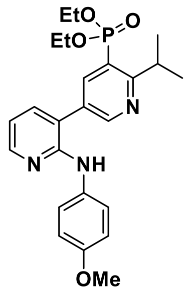

7zb

Off-white solid; Yield: 36% (33 mg); ^{1}H NMR (500 MHz, $CDCl_3$): δ 11.26 (s, 1H), 8.46 (t, *J* = 2.5 Hz, 1H), 8.03 (d, *J* = 5.5 Hz, 1H), 7.93 (dd, *J* = 15.0, 2.5 Hz, 1H), 7.79 (d, *J* = 7.0 Hz, 1H), 7.05 (t, *J* = 6.5 Hz, 1H), 6.75 (d, *J* = 8.5 Hz, 2H), 6.51 (d, *J* = 8.5 Hz, 2H), 4.24–4.03 (m, 4H), 3.65 (s, 3H), 3.53 (hept, *J* = 6.5 Hz, 1H), 1.37 (t, *J* = 7.0 Hz, 6H), 1.20 (d, *J* = 6.5 Hz, 6H); ^{13}C NMR (125 MHz, $CDCl_3$): δ 169.65 (d, *J* = 12.8 Hz), 157.79, 151.42, 151.09, 146.69, 141.67 (d, *J* = 9.8 Hz), 138.25, 129.27, 128.44 (d, *J* = 12.1 Hz), 125.72, 122.95, 121.73 (d, *J* = 187.1 Hz), 114.32, 114.13, 62.93 (d, *J* = 5.9 Hz), 55.26, 33.73, 22.07, 16.45 (d, *J* = 6.6 Hz); ^{31}P NMR (162 MHz, $CDCl_3$) δ 16.15; LRMS (ESI): *m/z* calcd for $C_{24}H_{31}N_3O_4P$ $[M+H]^+$: 459.21; Found: 456.2.

Compound 7zc: diethyl (2'-((4-methoxyphenyl)amino)-6-phenyl-[3,3'-bipyridin]-5-yl)phosphonate

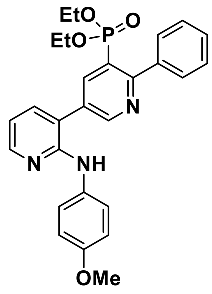

7zc

Yellow gum; Yield: 37% (36 mg); ^{1}H NMR (400 MHz, $CDCl_3$): δ 8.91 (t, *J* = 2.4 Hz, 1H), 8.43 (dd, *J* = 14.8, 2.4 Hz, 1H), 8.29–8.21 (m, 1H), 7.78–7.68 (m, 2H), 7.52–7.42 (m, 4H), 7.32 (d, *J* = 8.8 Hz, 2H), 6.89–6.79 (m, 3H), 6.23 (s, 1H), 4.05–3.84 (m, 4H), 3.78 (s, 3H), 1.14 (t, *J* = 7.2 Hz, 6H); ^{13}C NMR (100 MHz, $CDCl_3$): δ 161.75 (d, *J* = 11.2 Hz), 155.93, 153.76, 152.01 (d, *J* = 1.9 Hz), 148.50, 142.75 (d, *J* = 8.4 Hz), 140.34, 138.69, 133.12, 131.69 (d, *J* = 11.0 Hz), 129.36, 129.08, 127.98, 124.32 (d, *J* = 187.2 Hz), 123.26, 118.38, 115.08, 114.33, 62.72 (d, *J* = 6.3 Hz), 55.63, 16.17 (d, *J* = 6.8 Hz); ^{31}P NMR (162 MHz, $CDCl_3$) δ 14.92; LRMS (ESI): *m/z* calcd for $C_{27}H_{29}N_3O_4P$ $[M+H]^+$: 490.19; Found: 490.2.

Compound 7z'b: diethyl (2-isopropyl-5-(2-((4-methoxyphenyl)amino)phenyl)pyridin-3-yl)phosphonate

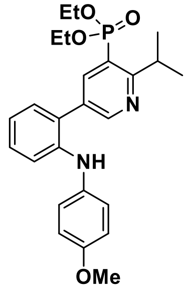

7z'b

Brown gum; Yield: 31% (28 mg); ^{1}H NMR (400 MHz, $CDCl_3$): δ 8.88 (t, *J* = 2.4 Hz, 1H), 8.30 (dd, *J* = 14.8, 2.4 Hz, 1H), 7.25 (d, *J* = 9.6 Hz, 1H), 7.19 (d, *J* = 7.6 Hz, 1H), 7.08 (d, *J* = 8.4 Hz, 1H), 7.00–6.91 (m, 3H), 6.81 (d, *J* = 8.8 Hz, 2H), 5.20 (br. s, 1H), 4.25–4.07 (m, 4H), 3.82–3.71 (m, 4H), 1.39–1.29 (m, 12H); ^{13}C NMR (100 MHz, $CDCl_3$): δ 168.65 (d, *J* = 13.5 Hz), 155.72, 152.46 (d, *J* = 1.9 Hz), 142.87, 142.75 (d, *J* = 9.2 Hz), 135.64, 132.35 (d, *J* = 11.3 Hz), 131.09, 129.64, 125.55, 123.03, 122.55 (d, *J* = 185.7 Hz), 120.54, 116.54, 114.80, 62.71 (d, *J* = 5.8 Hz), 55.67, 33.80, 22.46, 16.44 (d, *J* = 6.5 Hz);

^{31}P NMR (162 MHz, $CDCl_3$) δ 17.06; HRMS (ESI+): *m/z* calcd for $C_{25}H_{32}N_2O_4P$ $[M+H]^+$: 455.2100; Found: 455.2102.

Compound 7z'c: diethyl (5-(2-((4-methoxyphenyl)amino)phenyl)-2-phenylpyridin-3-yl)phosphonate

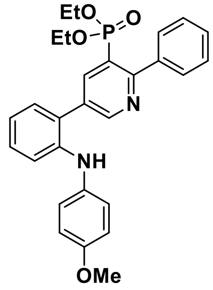

7z'c

Brown gum; Yield: 41% (41 mg); ^1H NMR (400 MHz, $CDCl_3$): 8.93 (t, J = 2.4 Hz, 1H), 8.46 (dd, J = 14.8, 2.4 Hz, 1H), 7.79–7.68 (m, 2H), 7.51–7.40 (m, 3H), 7.28–7.21 (m, 2H), 7.12 (d, J = 8.0 Hz, 1H), 7.04–6.95 (m, 3H), 6.87–6.78 (m, 2H), 5.04 (br. s, 1H), 4.01–3.81 (m, 4H), 3.78 (s, 3H), 1.11 (t, J = 7.2 Hz, 6H); ^{13}C NMR (100 MHz, $CDCl_3$): δ 160.93 (d, J = 11.4 Hz), 155.73, 152.19 (d, J = 1.8 Hz), 142.99, 142.90, 140.33, 135.55, 133.17 (d, J = 10.8 Hz), 131.05, 129.79, 129.36, 128.93, 127.92, 125.32, 123.85 (d, J = 187.4 Hz), 122.95, 120.56, 116.48, 114.83, 62.63 (d, J = 6.2 Hz), 55.65, 16.11 (d, J = 6.8 Hz); ^{31}P NMR (162 MHz, $CDCl_3$) δ 15.61; LRMS (ESI): *m/z* calcd for $C_{28}H_{30}N_2O_4P$ $[M+H]^+$: 489.19; Found: 489.1.

Compound 9aa: ethyl 5-(2-hydroxyphenyl)-2-methylnicotinate

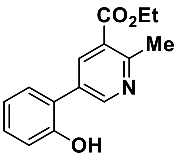

9aa

Off-white solid; Yield: 60% (31 mg); ^1H NMR (400 MHz, $CDCl_3$): δ 8.81 (d, J = 2.4 Hz, 1H), 8.40 (d, J = 2.4 Hz, 1H), 7.32–7.25 (m, 3H), 7.02 (td, J = 7.6, 0.8 Hz, 1H), 6.97 (d, J = 8.0 Hz, 1H), 4.40 (q, J = 7.2 Hz, 2H), 2.84 (s, 3H), 1.40 (t, J = 7.2 Hz, 3H); ^{13}C NMR (100 MHz, $CDCl_3$): δ 166.59, 158.06, 153.53, 151.86, 139.22, 131.68, 130.65, 130.06, 125.69, 124.11, 121.18, 116.65, 61.59, 24.34, 14.41; HRMS (ESI): *m/z* calcd for $C_{15}H_{16}NO_3$ $[M+H]^+$: 258.1130; Found: 258.1126.

Compound 9ad: ethyl 5-(2-hydroxyphenyl)-2-methylnicotinate

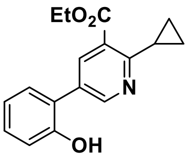

9ad

Off-white solid; Yield: 55% (31 mg); ^1H NMR (400 MHz, $CDCl_3$): δ 8.72 (d, J = 2.0 Hz, 1H), 8.27 (d, J = 2.4 Hz, 1H), 7.28–7.23 (m, 2H), 7.01 (t, J = 7.6 Hz, 1H), 6.93 (d, J = 8.0 Hz, 1H), 5.95 (br. s, 1H), 4.41 (q, J = 7.2 Hz, 2H), 3.04 (tt, J = 8.4, 4.4 Hz, 1H), 1.40 (t, J = 7.2 Hz, 3H), 1.25–1.18 (m, 2H), 1.10–1.03 (m, 2H); ^{13}C NMR (100 MHz, $CDCl_3$): δ 167.36, 162.24, 153.16, 152.12, 138.55, 130.60, 129.85, 129.76, 125.64, 124.32, 121.33, 116.40, 61.64, 14.41, 14.38, 11.20; LRMS (ESI): *m/z* calcd for $C_{17}H_{18}NO_3$ $[M+H]^+$: 284.13; Found: 284.1.

Compound 9af: ethyl 5-(2-hydroxyphenyl)-2-phenylnicotinate

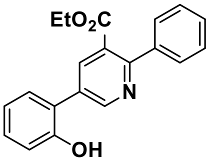

9af

Off-white solid; Yield: 40% (25.3 mg); ^{1}H NMR (400 MHz, $CDCl_3$): δ 9.01 (d, *J* = 2.4 Hz, 1H), 8.30 (d, *J* = 2.0 Hz, 1H), 7.61–7.52 (m, 2H), 7.47–7.38 (m, 3H), 7.33 (dd, *J* = 7.6, 1.6 Hz, 1H), 7.20 (td, *J* = 8.0, 1.6 Hz, 1H), 7.00 (t, *J* = 7.2 Hz, 1H), 6.78 (d, *J* = 8.0 Hz, 1H), 4.17 (q, *J* = 7.2 Hz, 2H), 1.05 (t, *J* = 7.2 Hz, 3H); ^{13}C NMR (100 MHz, $CDCl_3$): δ 168.36, 156.96, 153.64, 151.49, 139.76, 138.57, 132.58, 130.57, 130.12, 128.83, 128.77, 128.30, 127.25, 123.72, 121.13, 116.68, 61.80, 13.76; LRMS (ESI): *m/z* calcd for $C_{20}H_{18}NO_3$ $[M+H]^+$: 320.13; Found: 320.1.

Compound 9ba: ethyl 5-(5-chloro-2-hydroxyphenyl)-2-methylnicotinate

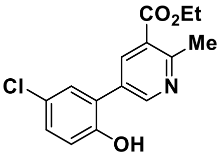

9ba

White solid; Yield: 62% (36 mg); ^{1}H NMR (400 MHz, DMSO-d_6): δ 10.15 (s, 1H), 8.78 (d, *J* = 2.4 Hz, 1H), 8.33 (d, *J* = 2.0 Hz, 1H), 7.41 (d, *J* = 2.4 Hz, 1H), 7.28 (dd, *J* = 8.8, 2.8 Hz, 1H), 6.99 (d, *J* = 8.8 Hz, 1H), 4.34 (q, *J* = 7.2 Hz, 2H), 2.74 (s, 3H), 1.34 (t, *J* = 7.2 Hz, 3H); ^{13}C NMR (100 MHz, DMSO-d_6): δ 166.09, 156.79, 153.55, 151.34, 137.91, 130.25, 129.30, 129.14, 125.00, 124.66, 123.12, 117.68, 61.09, 23.99, 14.04; LRMS (ESI): *m/z* calcd for $C_{15}H_{15}ClNO_3$ $[M+H]^+$: 292.07; Found: 292.05.

Compound 9bd: ethyl 5-(5-chloro-2-hydroxyphenyl)-2-cyclopropylnicotinate

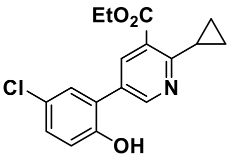

9bd

Colorless gum; Yield: 58% (37 mg); ^{1}H NMR (400 MHz, $CDCl_3$): δ 8.69 (d, *J* = 2.4 Hz, 1H), 8.23 (d, *J* = 2.4 Hz, 1H), 7.23 (d, *J* = 2.4 Hz, 1H), 7.17 (dd, *J* = 8.8, 2.8 Hz, 1H), 6.93 (br. s, 1H), 6.85 (d, *J* = 8.8 Hz, 1H), 4.42 (q, *J* = 7.2 Hz, 2H), 3.00 (tt, *J* = 8.4, 4.8 Hz, 1H), 1.41 (t, *J* = 7.2 Hz, 3H), 1.22–1.14 (m, 2H), 1.10–0.99 (m, 2H); ^{13}C NMR (100 MHz, $CDCl_3$): δ 167.27, 162.54, 152.14, 151.88, 138.56, 130.06, 129.49, 129.02, 125.93, 125.87, 125.81, 117.76, 61.83, 14.56, 14.40, 11.26; HRMS (ESI): *m/z* calcd for $C_{17}H_{17}ClNO_3$ $[M+H]^+$: 318.0897; Found: 318.0898.

Compound 9bf: ethyl 5-(5-chloro-2-hydroxyphenyl)-2-phenylnicotinate

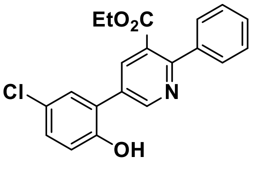

9bf

Off-white solid; Yield: 30% (21 mg); ^{1}H NMR (400 MHz, $CDCl_3$): δ 9.03 (d, *J* = 2.0 Hz, 1H), 8.24 (d, *J* = 2.0 Hz, 1H), 7.58–7.49 m, 2H), 7.47–7.37 (m, 3H), 7.30–7.26 (m, 1H), 7.04 (dd, *J* = 8.8, 2.4 Hz, 1H), 6.52 (d, *J* = 8.8 Hz, 1H), 4.17 (q, *J* = 7.2 Hz, 2H), 1.05 (t, *J* = 7.2 Hz, 3H); ^{13}C NMR (100 MHz, $CDCl_3$): δ 167.99, 157.08, 152.72, 151.31, 139.25, 138.62, 131.97, 129.87, 129.69, 129.06, 128.75, 128.37, 127.50, 125.40, 124.94, 117.98, 61.95, 13.75; LRMS (ESI): *m/z* calcd for $C_{20}H_{17}ClNO_3$ $[M+H]^+$: 354.09; Found: 354.1.

Compound 9ca: ethyl 5-(5-bromo-2-hydroxyphenyl)-2-methylnicotinate

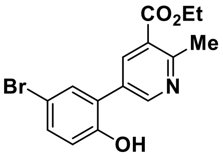

9ca

White solid; Yield: 68% (46 mg); ^{1}H NMR (400 MHz, DMSO-d_6): δ 10.19 (s, 1H), 8.77 (d, *J* = 2.4 Hz, 1H), 8.32 (d, *J* = 2.4 Hz, 1H), 7.52 (d, *J* = 2.4 Hz, 1H), 7.40 (dd, *J* = 8.4, 2.8 Hz, 1H), 6.94 (d, *J* = 8.4 Hz, 1H), 4.34 (q, *J* = 7.2 Hz, 2H), 2.74 (s, 3H), 1.34 (t, *J* = 7.2 Hz, 3H); ^{13}C NMR (100 MHz, DMSO-d_6): δ 166.11, 156.82, 154.02, 151.37, 137.95, 132.11, 132.08, 130.19, 125.60, 124.68, 118.19, 110.70, 61.13, 24.03, 14.07; LRMS (ESI): *m/z* calcd for $C_{15}H_{15}BrNO_3$ $[M+H]^+$: 336.02; Found: 336.0.

Compound 9cd: ethyl 5-(5-bromo-2-hydroxyphenyl)-2-cyclopropylnicotinate

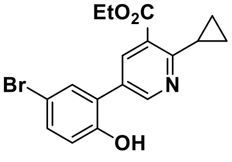

9cd

White solid; Yield: 64% (46 mg); ^{1}H NMR (400 MHz, $CDCl_3$): δ 8.68 (d, *J* = 2.0 Hz, 1H), 8.22 (d, *J* = 2.4 Hz, 1H), 7.37 (d, *J* = 2.4 Hz, 1H), 7.31 (dd, *J* = 8.4, 2.4 Hz, 1H), 6.89 (br. s, 1H), 6.81 (d, *J* = 8.8 Hz, 1H), 4.42 (q, *J* = 7.2 Hz, 2H), 3.00 (tt, *J* = 8.8, 5.2 Hz, 1H), 1.41 (t, *J* = 7.2 Hz, 3H), 1.21–1.15 (m, 2H), 1.09– 1.00 (m, 2H); ^{13}C NMR (100 MHz, $CDCl_3$): 167.25, 162.59, 152.64, 151.89, 138.53, 132.93, 132.43, 128.86, 126.36, 125.91, 118.20, 113.02, 61.82, 14.55, 14.41, 11.29; LRMS (ESI): *m/z* calcd for $C_{17}H_{17}BrNO_3$ $[M+H]^+$: 362.04; Found: 362.1.

Compound 9cf: ethyl 5-(5-bromo-2-hydroxyphenyl)-2-phenylnicotinate

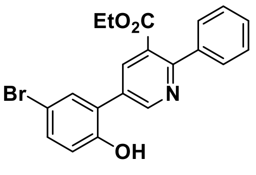

9cf

Off-white solid; Yield: 39% (31 mg); ^{1}H NMR (400 MHz, $CDCl_3$): δ 9.26 (br. s, 1H), 9.03 (d, *J* = 2.4 Hz, 1H), 8.23 (d, *J* = 2.0 Hz, 1H), 7.57–7.47 (m, 2H), 7.45–7.36 (m, 4H), 7.13 (dd, *J* = 8.8, 2.4 Hz, 1H), 6.39 (d, *J* = 8.8 Hz, 1H), 4.17 (q, *J* = 7.2 Hz, 2H), 1.05 (t, *J* = 7.2 Hz, 3H); ^{13}C NMR (100 MHz, $CDCl_3$): δ 167.93, 156.99, 153.35, 151.27, 139.13, 138.68, 132.70, 132.60, 132.04, 129.08, 128.74, 128.38, 127.58, 125.42, 118.39, 112.39, 61.97, 13.75; LRMS (ESI): *m/z* calcd for $C_{20}H_{17}BrNO_3$ $[M+H]^+$: 398.04; Found: 398.0.

Compound 9da: ethyl 5-(2-hydroxy-5-nitrophenyl)-2-methylnicotinate

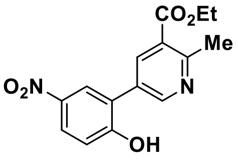

9da

Yellow solid; Yield: 60% (36 mg); ^{1}H NMR (400 MHz, $CDCl_3$): δ 8.96 (d, *J* = 2.0 Hz, 1H), 8.45 (d, *J* = 2.4 Hz, 1H), 8.28 (d, *J* = 2.8 Hz, 1H), 8.17 (dd, *J* = 8.0, 2.8 Hz, 1H), 7.06 (d, *J* = 8.8 Hz, 1H), 4.45 (q, *J* = 7.2 Hz, 2H), 2.88 (s, 3H), 1.45 (t, *J* = 7.2 Hz, 3H); ^{13}C NMR (125 MHz, $CDCl_3$): δ 165.73, 160.89, 158.04, 151.29, 141.12, 139.89, 131.05, 126.88, 126.76, 126.29, 124.39, 116.40, 62.10, 23.72, 14.44; LRMS (ESI): *m/z* calcd for $C_{15}H_{15}N_2O_5$ $[M+H]^+$: 303.10; Found: 303.1.

Compound 9dd: ethyl 2-cyclopropyl-5-(2-hydroxy-5-nitrophenyl)nicotinate

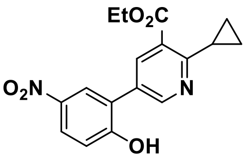

9dd

Off-white solid; Yield: 40% (26 mg); ^{1}H NMR (500 MHz, $CDCl_3$): δ 8.76 (d, *J* = 2.5 Hz, 1H), 8.30 (d, *J* = 2.0 Hz, 1H), 8.22 (d, *J* = 2.5 Hz, 1H), 8.13 (dd, *J* = 9.0, 2.5 Hz, 1H), 7.02 (d, *J* = 9.0 Hz, 1H), 4.46 (q, *J* = 7.0 Hz, 2H), 3.02 (tt, *J* = 8.0, 5.0 Hz, 1H), 1.44 (t, *J* = 7.0 Hz, 3H), 1.20–1.15 (m, 2H), 1.12–1.06 (m, 2H); ^{13}C NMR (125 MHz, $CDCl_3$): δ 167.14, 162.95, 159.71, 151.71, 141.53, 138.80, 128.43, 126.57, 126.38, 125.84, 124.85, 116.52, 62.10, 14.71, 14.39, 11.41; LRMS (ESI): *m/z* calcd for $C_{17}H_{15}N_2O_5$ $[M+H]^+$: 329.11; Found: 329.2.

Compound 9df: ethyl 5-(2-hydroxy-5-nitrophenyl)-2-phenylnicotinate

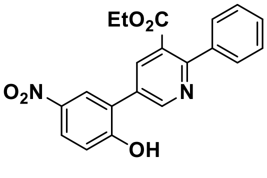

9df

Off-white solid; Yield: 30% (22 mg); ^{1}H NMR (500 MHz, $CDCl_3$): δ 9.19 (d, *J* = 2.0 Hz, 1H), 8.34 (d, *J* = 2.0 Hz, 1H), 8.27 (d, *J* = 2.5 Hz, 1H), 7.91 (dd, *J* = 9.0, 2.5 Hz, 1H), 7.59–7.53 (m, 2H), 7.48–7.41 (m, 3H), 6.44 (d, *J* = 9.0 Hz, 1H), 4.21 (q, *J* = 7.0 Hz, 2H), 1.08 (t, *J* = 7.0 Hz, 3H); ^{13}C NMR (125 MHz, $CDCl_3$): δ 167.33, 160.35, 157.41, 151.08, 141.17, 139.13, 138.67, 131.65, 129.48, 128.74, 128.61, 128.23, 126.52, 126.00, 123.71, 116.58, 62.22, 13.80; HRMS (ESI): *m/z* calcd for $C_{20}H_{17}N_2O_5$ $[M+H]^+$: 365.1137; Found: 365.1136.

Compound 9ea: ethyl 5-(2-hydroxy-4-nitrophenyl)-2-methylnicotinate

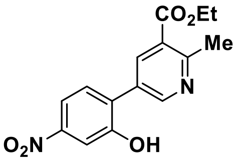

9ea

Yellow solid; Yield: 70% (42 mg); ^{1}H NMR (400 MHz, DMSO-d_6): δ 10.96 (s, 1H), 8.83 (d, *J* = 2.0 Hz, 1H), 8.38 (d, *J* = 2.4 Hz, 1H), 7.80–7.73 (m, 2H), 7.64 (d, *J* = 8.4 Hz, 1H), 4.34 (q, *J* = 7.2 Hz, 2H), 2.75 (s, 3H), 1.33 (t, *J* = 7.2 Hz, 3H); ^{13}C NMR (100 MHz, DMSO-d_6): δ 165.93, 157.69, 155.28, 151.34, 147.82, 138.02, 131.12, 130.20, 129.65, 124.75, 114.38, 110.29, 61.20, 24.11, 14.04; LRMS (ESI): *m/z* calcd for $C_{15}H_{15}N_2O_5$ $[M+H]^+$: 303.10; Found: 303.2.

Compound 9ed: ethyl 2-cyclopropyl-5-(2-hydroxy-4-nitrophenyl)nicotinate

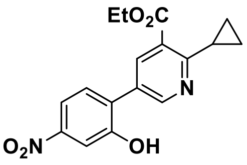

9ed

Yellow solid; Yield: 50% (33 mg); ^{1}H NMR (400 MHz, $CDCl_3$): δ 8.76 (d, *J* = 2.4 Hz, 1H), 8.35 (d, *J* = 2.0 Hz, 1H), 8.17 (br. s, 1H), 7.89–7.78 (m, 2H), 7.45 (d, *J* = 8.0 Hz, 1H), 4.47 (q, *J* = 7.2 Hz, 2H), 3.01 (tt, *J* = 8.4, 4.4 Hz, 1H), 1.44 (t, *J* = 7.2 Hz, 3H), 1.23–1.17 (m, 2H), 1.14–1.06 (m, 2H); ^{13}C NMR (100 MHz, $CDCl_3$): δ 167.55, 163.22, 154.34, 151.67, 148.44, 138.78, 130.98, 130.95, 128.50, 126.03, 115.97, 111.38, 62.20, 14.81, 14.38, 11.52; LRMS (ESI): *m/z* calcd for $C_{17}H_{15}N_2O_5$ $[M+H]^+$: 329.11; Found: 329.1.

Compound 9ef: ethyl 5-(2-hydroxy-4-nitrophenyl)-2-phenylnicotinate

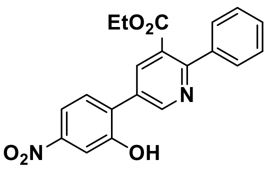

9ef

Yellow solid; Yield: 43% (31 mg); ^{1}H NMR (500 MHz, $CDCl_3$): δ 9.94 (br. s, 1H), 9.15 (d, *J* = 2.0 Hz, 1H), 8.35 (d, *J* = 2.0 Hz, 1H), 7.79 (dd, *J* = 8.4, 2.0 Hz, 1H), 7.60–7.53 (m, 2H), 7.49 (d, *J* = 8.4 Hz, 1H), 7.45–7.35 (m, 4H), 4.20 (q, *J* = 7.2 Hz, 2H), 1.05 (t, *J* = 7.2 Hz, 3H); ^{13}C NMR (100 MHz, $CDCl_3$): δ 167.88, 157.93, 154.89, 151.10, 148.73, 139.07, 138.82, 131.43, 130.89, 129.97, 129.33, 128.72, 128.53, 127.86, 115.52, 111.56, 62.22, 13.73; LRMS (ESI): *m/z* calcd for $C_{20}H_{17}N_2O_5$ $[M+H]^+$: 365.11; Found: 365.1.

Compound 9fa: ethyl 5-(2-hydroxy-5-methylphenyl)-2-methylnicotinate

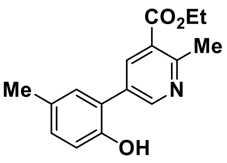

9fa

Off-white solid; Yield: 63% (34.4 mg); ^{1}H NMR (400 MHz, $CDCl_3$): δ 8.80 (d, *J* = 2.0 Hz, 1H), 8.41 (d, *J* = 2.4 Hz, 1H), 8.41 (br. s, 1H), 7.08 (d, *J* = 2.0 Hz, 1H), 7.03 (dd, *J* = 8.4, 2.4 Hz, 1H), 6.86 (d, *J* = 8.0 Hz, 1H), 4.38 (q, *J* = 7.2 Hz, 2H), 2.81 (s, 3H), 2.31 (s, 3H), 1.39 (t, *J* = 7.2 Hz, 2H); ^{13}C NMR (100 MHz, $CDCl_3$): δ 166.58, 157.45, 151.81, 151.69, 139.40, 132.38, 130.91, 130.50, 130.00, 125.70, 123.77, 116.65, 61.58, 24.00, 20.58, 14.38; HRMS (ESI): *m/z* calcd for $C_{16}H_{18}NO_3$ $[M+H]^+$: 272.1287; Found: 272.1284.

Compound 9fd: ethyl 2-cyclopropyl-5-(2-hydroxy-5-methylphenyl)nicotinate

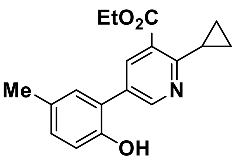

9fd

Colorless gum; Yield: 57% (34 mg); ^{1}H NMR (400 MHz, $CDCl_3$): δ 8.71 (d, *J* = 2.4 Hz, 1H), 8.25 (d, *J* = 2.4 Hz, 1H), 7.08–7.00 (m, 2H), 6.82 (d, *J* = 8.0 Hz, 1H), 6.02 (br. s, 1H), 4.40 (q, *J* = 7.2 Hz, 2H), 3.02 (tt, *J* = 8.4, 4.8 Hz, 1H), 2.31 (s, 3H), 1.40 (t, *J* = 7.2 Hz, 3H), 1.23–1.17 (m, 2H), 1.08–1.01 (m, 2H); ^{13}C NMR (100 MHz, $CDCl_3$): δ 167.40, 162.01, 152.11, 150.97, 138.53, 130.96, 130.49, 130.27, 130.03, 125.62, 124.01, 116.29, 61.62, 20.60, 14.40, 14.38, 11.13; LRMS (ESI): *m/z* calcd for $C_{18}H_{20}NO_3$ $[M+H]^+$: 298.14; Found: 298.2.

Compound 9ff: ethyl 5-(2-hydroxy-5-methylphenyl)-2-phenylnicotinate

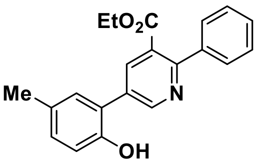

9ff

Yellow solid; Yield: 33% (22 mg); ^{1}H NMR (400 MHz, $CDCl_3$): δ 9.00 (d, *J* = 2.0 Hz, 1H), 8.28 (d, *J* = 2.0 Hz, 1H), 7.58–7.52 (m, 2H), 7.50–7.35 (m, 4H), 7.12 (d, *J* = 2.4 Hz, 1H), 6.96 (dd, *J* = 8.0, 2.4 Hz, 1H), 6.62 (d, *J* = 8.4 Hz, 1H), 4.16 (q, *J* = 7.2 Hz, 2H), 2.30 (s, 3H), 1.03 (t, *J* = 7.2 Hz, 3H); ^{13}C NMR (100 MHz, $CDCl_3$): δ 168.39, 156.71, 151.49, 139.70, 138.55, 132.89, 130.87, 130.55, 130.18, 128.79, 128.76, 128.26, 127.22, 123.36, 116.59, 61.76, 20.58, 13.74; LRMS (ESI): *m/z* calcd for $C_{21}H_{20}NO_3$ $[M+H]^+$: 334.14; Found: 334.2.

Compound 9ga: ethyl 5-(2-hydroxy-4-methylphenyl)-2-methylnicotinate

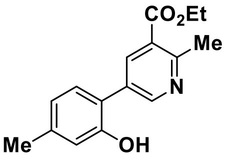

9ga

White solid; Yield: 81% (44 mg); ^{1}H NMR (400 MHz, $CDCl_3$): δ 8.83 (d, *J* = 2.4 Hz, 1H), 8.75 (br. s, 1H), 8.42 (d, *J* = 2.0 Hz, 1H), 7.18 (d, *J* = 8.0 Hz, 1H), 6.84–6.75 (m, 2H), 4.38 (q, *J* = 7.2 Hz, 2H), 2.81 (s, 3H), 2.31 (s, 3H), 1.39 (t, *J* = 7.0 Hz, 3H); ^{13}C NMR (100 MHz, $CDCl_3$): δ 166.58, 157.18, 154.04, 151.63, 140.40, 139.38, 132.40, 130.27, 125.73, 121.61, 121.13, 117.35, 61.56, 23.93, 21.36, 14.37; LRMS (ESI): *m/z* calcd for $C_{16}H_{18}NO_3$ $[M+H]^+$: 272.13; Found: 272.2.

Compound 9gd: ethyl 2-cyclopropyl-5-(2-hydroxy-4-methylphenyl)nicotinate

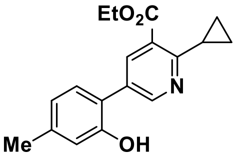

9gd

Yellow solid; Yield: 64% (378 mg); ^{1}H NMR (400 MHz, $CDCl_3$): δ 8.71 (d, *J* = 2.4 Hz, 1H), 8.26 (d, *J* = 2.4 Hz, 1H), 7.15 (d, *J* = 7.6 Hz, 1H), 6.82 (d, *J* = 7.6 Hz, 1H), 6.75 (s, 1H), 6.21 (br. s, 1H), 4.40 (q, *J* = 7.2 Hz, 2H), 3.02 (tt, *J* = 8.0, 4.8 Hz, 1H), 2.32 (s, 3H), 1.40 (t, *J* = 7.2 Hz, 3H), 1.22–1.17 (m, 2H), 1.08–1.01 (m, 2H); ^{13}C NMR (100 MHz, $CDCl_3$): δ 167.44, 161.80, 153.09, 152.08, 140.20, 138.51, 130.30, 129.98, 125.62, 122.09, 121.38, 117.03, 61.61, 21.31, 14.39, 14.36, 11.09; LRMS (ESI): *m/z* calcd for $C_{18}H_{20}NO_3$ $[M+H]^+$: 298.14; Found: 298.2.

Compound 9gf: ethyl 5-(2-hydroxy-4-methylphenyl)-2-phenylnicotinate

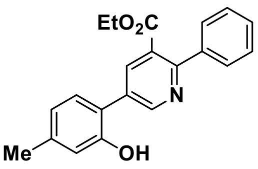

9gf

Yellow solid; Yield: 35% (23 mg); ^{1}H NMR (400 MHz, $CDCl_3$): δ 9.06 (d, *J* = 2.0 Hz, 1H), 8.30 (d, *J* = 2.4 Hz, 1H), 8.05 (br. s, 1H), 7.60–7.51 (m, 2H), 7.46–7.39 (m, 3H), 7.21 (d, *J* = 7.6 Hz, 1H), 6.77 (d, *J* = 8.0 Hz, 1H), 6.47 (s, 1H), 4.16 (q, *J* = 7.2 Hz, 2H), 2.21 (s, 3H), 1.03 (t, *J* = 7.2 Hz, 3H); ^{13}C NMR (100 MHz, $CDCl_3$): δ 168.38, 156.42, 153.75, 151.50, 140.45, 139.72, 138.43, 133.01, 130.19, 128.78, 128.77, 128.29, 127.28, 121.73, 120.66, 117.29, 61.75, 21.28, 13.75; LRMS (ESI): *m/z* calcd for $C_{21}H_{20}NO_3$ $[M+H]^+$: 334.14; Found: 334.2.

Compound 9ha: ethyl 5-(2-hydroxy-5-methoxyphenyl)-2-methylnicotinate

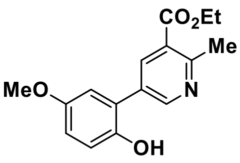

9ha

Off-white solid; Yield: 67% (40 mg); ^{1}H NMR (400 MHz, DMSO-*d$_6$*): δ 9.36 (s, 1H), 8.79 (d, *J* = 2.0 Hz, 1H), 8.35 (d, *J* = 2.0 Hz, 1H), 6.97–6.88 (m, 2H), 6.84 (dd, *J* = 8.8, 2.8 Hz, 1H), 4.34 (q, *J* = 7.2 Hz, 2H), 3.73 (s, 3H), 2.74 (s, 3H), 1.34 (t, *J* = 7.2 Hz, 3H); ^{13}C NMR (100 MHz, DMSO-*d$_6$*): δ 166.24, 156.32, 152.56, 151.44, 148.33, 137.94, 131.55, 124.58, 123.58, 116.90, 115.09, 114.95, 61.09, 55.48, 24.03, 14.07; LRMS (ESI): *m/z* calcd for $C_{16}H_{18}NO_4$ $[M+H]^+$: 288.12; Found: 288.1.

Compound 9hd: ethyl 2-cyclopropyl-5-(2-hydroxy-5-methoxyphenyl)nicotinate

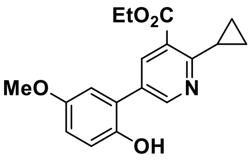

9hd

Colorless gum; Yield: 56% (35 mg); ^{1}H NMR (400 MHz, $CDCl_3$): δ 8.71 (d, *J* = 2.4 Hz, 1H), 8.27 (d, *J* = 2.4 Hz, 1H), 6.89–6.77 (m, 3H), 6.16 (br. s, 1H), 4.40 (q, *J* = 7.2 Hz, 2H), 3.78 (s, 3H), 3.01 (tt, *J* = 8.4, 4.8 Hz, 1H), 1.40 (t, *J* = 7.2 Hz, 3H), 1.22–1.15 (m, 2H), 1.08–1.00 (m, 2H); ^{13}C NMR (100 MHz, $CDCl_3$): δ 167.36, 162.16, 153.88, 152.04, 147.25, 138.56, 129.99, 125.63, 125.00, 117.34, 115.64, 115.00, 61.65, 55.99, 14.40, 14.38, 11.16; HRMS (ESI): *m/z* calcd for $C_{18}H_{20}NO_4$ $[M+H]^+$: 314.1392; Found: 314.1392.

Compound 9hf: ethyl 5-(2-hydroxy-5-methoxyphenyl)-2-phenylnicotinate

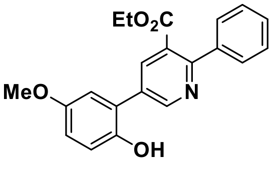

9hf

Yellow solid; Yield: 47% (43 mg); ^{1}H NMR (400 MHz, $CDCl_3$): δ 8.99 (d, *J* = 2.4 Hz, 1H), 8.27 (d, *J* = 2.0 Hz, 1H), 7.59–7.49 (m, 2H), 7.45–7.38 (m, 3H), 7.33 (br. s, 1H), 6.85 (d, *J* = 3.2 Hz, 1H), 6.73 (dd, *J* = 8.8, 2.8 Hz, 1H), 6.65 (d, *J* = 8.8 Hz, 1H), 4.16 (q, *J* = 7.2 Hz, 2H), 3.78 (s, 3H), 1.04 (t, *J* = 7.2 Hz, 3H); ^{13}C NMR (100 MHz, $CDCl_3$): δ 168.30, 156.92, 153.81, 151.45, 147.63, 139.60, 138.55, 132.69, 128.84, 128.76, 128.26, 127.23, 124.33, 117.72, 115.47, 115.42, 61.80, 56.02, 13.74; LRMS (ESI): *m/z* calcd for $C_{21}H_{20}NO_4$ $[M+H]^+$: 350.14; Found: 350.2.

Compound 9ia: ethyl 5-(2-hydroxy-4-methoxyphenyl)-2-methylnicotinate

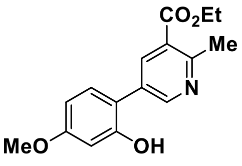

9ia

Yellow solid; Yield: 45% (26 mg); ^{1}H NMR (400 MHz, CD_3OD): δ 8.72 (d, *J* = 2.0 Hz, 1H), 8.43 (d, *J* = 2.4 Hz, 1H), 7.24 (d, *J* = 8.4 Hz, 1H), 6.54 (dd, *J* = 8.4, 2.4 Hz, 1H), 6.50 (d, *J* = 2.4 Hz, 1H), 4.39 (q, *J* = 7.2 Hz, 2H), 3.79 (s, 3H), 2.78 (s, 3H), 1.40 (t, *J* = 7.2 Hz, 3H); ^{13}C NMR (100 MHz, CD_3OD): δ 167.82, 162.68, 157.20, 157.00, 151.98, 140.07, 134.08, 131.79, 126.84, 117.26, 106.92, 102.77, 62.54, 55.70, 23.80, 14.54; LRMS (ESI): *m/z* calcd for $C_{16}H_{18}NO_4$ $[M+H]^+$: 288.12; Found: 288.2.

Compound 9gd: ethyl 2-cyclopropyl-5-(2-hydroxy-4-methoxyphenyl)nicotinate

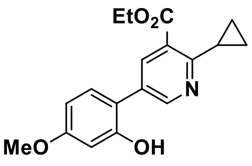

9id

Yellow solid; Yield: 35% (22 mg); ^{1}H NMR (400 MHz, $CDCl_3$): δ 8.68 (d, *J* = 2.4 Hz, 1H), 8.22 (d, *J* = 2.4 Hz, 1H), 7.17 (d, *J* = 8.4 Hz, 1H), 6.58 (dd, *J* = 8.4, 2.4 Hz, 1H), 6.51 (d, *J* = 2.8 Hz, 1H), 4.41 (q, *J* = 7.2 Hz, 2H), 3.81 (s, 3H), 3.02 (tt, *J* = 8.4, 4.8 Hz, 1H), 1.40 (t, *J* = 7.2 Hz, 3H), 1.22–1.17 (m, 2H), 1.09–1.01 (m, 2H); ^{13}C NMR (100 MHz, $CDCl_3$): δ 167.39, 161.77, 161.04, 154.14, 152.08, 138.38, 131.17, 129.59, 125.67, 117.02, 107.03, 102.29, 61.61, 55.57, 14.41, 14.33, 11.11; LRMS (ESI): *m/z* calcd for $C_{18}H_{20}NO_4$ $[M+H]^+$: 314.14; Found: 314.2.

Compound 9gf: ethyl 5-(2-hydroxy-4-methoxyphenyl)-2-phenylnicotinate

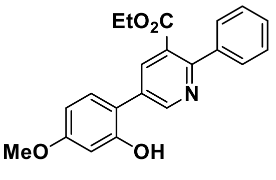

9if

Yellow solid; Yield: 20% (14 mg); ^{1}H NMR (400 MHz, $CDCl_3$): δ 9.00 (d, *J* = 2.0 Hz, 1H), 8.25 (d, *J* = 2.4 Hz, 1H), 7.58–7.51 (m, 2H), 7.45–7.37 (m, 3H), 7.24 (d, *J* = 8.4 Hz, 1H), 6.55 (dd, *J* = 8.4, 2.4 Hz, 1H), 6.35 (d, *J* = 2.4 Hz, 1H), 4.16 (q, *J* = 7.2 Hz, 2H), 3.71 (s, 3H), 1.04 (t, *J* = 7.2 Hz, 3H); ^{13}C NMR (100 MHz, $CDCl_3$): δ 168.45, 161.25, 156.18, 154.90, 151.30, 139.61, 138.32, 132.70, 131.13, 128.80, 128.75, 128.29, 127.32, 116.41, 106.99, 102.45, 61.78, 55.48, 29.84, 13.75.; LRMS (ESI): *m/z* calcd for $C_{21}H_{20}NO_4$ $[M+H]^+$: 350.14; Found: 350.2.

Compound 9ja: ethyl 5-(2-hydroxynaphthalen-1-yl)-2-methylnicotinate

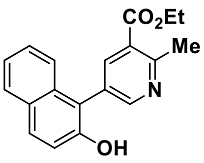

9ja

Off-white solid; Yield: 62% (38 mg); ^{1}H NMR (400 MHz, $CDCl_3$): δ 8.53 (d, *J* = 2.4 Hz, 1H), 8.31 (d, *J* = 2.0 Hz, 1H), 7.85–7.77 (m, 2H), 7.38–7.20 (m, 4H), 4.38 (q, *J* = 7.2 Hz, 2H), 2.74 (s, 3H), 1.37 (t, *J* = 7.2 Hz, 3H); ^{13}C NMR (100 MHz, $CDCl_3$): δ 165.99, 158.71, 153.75, 152.33, 141.82, 133.58, 130.63, 129.76, 128.83, 128.35, 127.03, 126.09, 123.92, 123.56, 119.01, 116.26, 61.73, 24.04, 14.35; LRMS (ESI): *m/z* calcd for $C_{19}H_{18}NO_3$ $[M+H]^+$: 308.13; Found: 308.1.

Compound 9jd: ethyl 2-cyclopropyl-5-(2-hydroxynaphthalen-1-yl)nicotinate

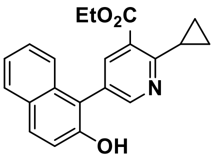

9jd

Colorless gum; Yield: 51% (34 mg); ^{1}H NMR (400 MHz, $CDCl_3$): δ 8.54 (d, *J* = 2.4 Hz, 1H), 8.19 (d, *J* = 2.4 Hz, 1H), 7.81 (d, *J* = 8.0 Hz, 2H), 7.39–7.28 (m, 3H), 7.24 (d, *J* = 8.8 Hz, 1H), 6.19 (br. s, 1H), 4.37 (q, *J* = 7.2 Hz, 2H), 3.08 (tt, *J* = 8.4, 4.8 Hz, 1H), 1.36 (t, *J* = 7.2 Hz, 3H), 1.26–1.21 (m, 2H), 1.14–1.04 (m, 2H); ^{13}C NMR (100 MHz, $CDCl_3$): δ 166.96, 163.32, 154.05, 151.41, 141.04, 133.57, 130.46, 128.98, 128.33, 127.07, 126.96, 126.06, 124.14, 123.69, 118.07, 116.77, 61.72, 14.54, 14.34, 11.66, 11.14; LRMS (ESI): *m/z* calcd for $C_{21}H_{20}NO_3$ $[M+H]^+$: 334.14; Found: 334.2.

Compound 9jf: ethyl 5-(2-hydroxynaphthalen-1-yl)-2-phenylnicotinate

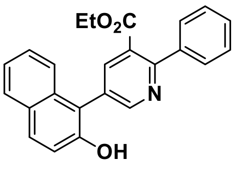

9jf

Pale-brown gum; Yield: 38% (28 mg); ^{1}H NMR (500 MHz, $CDCl_3$): δ 8.80 (d, *J* = 2.0 Hz, 1H), 8.18 (d, *J* = 2.0 Hz, 1H), 7.80 (d, *J* = 8.0 Hz, 1H), 7.75 (d, *J* = 9.0 Hz, 1H), 7.63–7.59 (m, 2H), 7.48–7.41 (m, 4H), 7.40–7.32 (m, 2H), 7.11 (d, *J* = 9.0 Hz, 1H), 6.83 (br. s, 1H), 4.17 (q, *J* = 7.0 Hz, 2H), 1.05 (t, *J* = 7.0 Hz, 3H); ^{13}C NMR (125 MHz, $CDCl_3$): δ 168.05, 157.90, 153.38, 151.48, 140.96, 139.58, 133.38, 130.59, 129.97, 129.03, 128.98, 128.86, 128.40, 128.35, 127.64, 127.21, 124.00, 123.70, 118.14, 116.49, 61.90, 13.77; HRMS (ESI): *m/z* calcd for $C_{24}H_{20}NO_3$ $[M+H]^+$: 370.1443; Found: 370.1444.

V. Synthetic Application for the Synthesis of Privileged Pyridine Scaffold Containing Bio-relevant Molecules

Compound 10a: ethyl 2'-((3-chloro-2-methylphenyl)amino)-6-methyl-[3,3'-bipyridine]-5-carboxylate

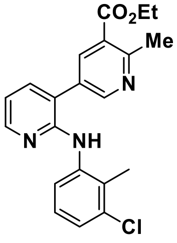

10a
Clonixin analog

Colorless gum; Yield: 59% (46 mg); ^{1}H NMR (500 MHz, $CDCl_3$): δ 9.00 (br. s, 1H), 8.54 (d, *J* = 2.5 Hz, 1H), 8.16 (dd, *J* = 5.5, 2.0 Hz, 1H), 7.99 (d, *J* = 2.5 Hz, 1H), 7.65 (dd, *J* = 7.5, 2.0 Hz, 1H), 7.06–6.95 (m, 3H), 6.85 (t, *J* = 8.0 Hz, 1H), 4.39 (q, *J* = 7.0 Hz, 2H), 2.80 (s, 3H), 2.27 (s, 3H), 1.41 (t, *J* = 7.0 Hz, 3H); ^{13}C NMR (125 MHz, $CDCl_3$): δ 165.51, 159.72, 152.50, 150.34, 143.19, 142.76, 138.26, 138.05, 135.35, 130.36, 129.78, 126.67, 126.23, 125.11, 122.45, 121.14, 115.21, 61.84, 24.42, 15.20, 14.37; HRMS (ESI+): *m/z* calcd for $C_{21}H_{21}ClN_3O_2$ $[M+H]^+$: 382.1322; Found: 382.1325.

Compound 10b: 2-butoxyethyl 2-methyl-5-(2-(phenylsulfonamido)phenyl)nicotinate

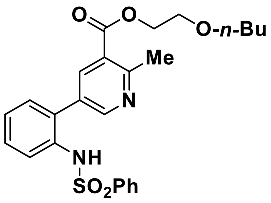

10b
Nicoboxil analog

White solid; Yield: 78% (73 mg); ^{1}H NMR (500 MHz, $CDCl_3$): δ 8.36 (d, *J* = 2.5 Hz, 1H), 8.02 (d, *J* = 2.5 Hz, 1H), 7.60–7.47 (m, 5H), 7.42–7.33 (m, 3H), 7.28 (t, *J* = 7.5 Hz, 1H), 7.16 (dd, *J* = 7.5, 1.5 Hz, 1H), 4.48 (t, *J* = 4.5 Hz, 2H), 3.81–3.70 (m, 2H), 3.50 (t, *J* = 6.5 Hz, 2H), 2.85 (s, 3H), 1.63–1.50 (m, 2H), 1.40–1.30 (m, 2H), 0.88 (t, *J* = 7.5 Hz, 3H); ^{13}C NMR (125 MHz, $CDCl_3$): δ 165.42, 158.06, 149.90, 140.54, 139.29, 133.70, 133.09, 132.21, 131.59, 130.80, 129.98, 129.11, 127.05, 126.77, 126.20, 125.47, 71.24, 68.36, 64.92, 31.71, 23.37, 19.32, 13.93; HRMS (ESI+): *m/z* calcd for $C_{25}H_{29}N_2O_5S$ $[M+H]^+$: 469.1797; Found: 469.1801.

Compound 10c: ethyl 2-butyl-5-(2-(phenylsulfonamido)phenyl)nicotinate

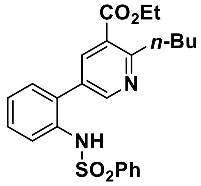

10c
Fusaric acid analog

White solid; Yield: 60% (53 mg); ^{1}H NMR (400 MHz, $CDCl_3$): δ 8.16 (d, *J* = 2.4 Hz, 1H), 7.72 (d, *J* = 2.4 Hz, 1H), 7.68 (d, *J* = 8.0 Hz, 1H), 7.58–7.49 (m, 3H), 7.44–7.34 m, 3H), 7.27–7.22 (m, 1H), 7.12 (d, *J* = 7.6 Hz, 1H), 6.71 (s, 1H), 4.40 (q, *J* = 7.2 Hz, 2H), 3.22–3.12 (m, 2H), 1.79–1.66 (m, 2H), 1.52–1.39 (m, 5H), 0.98 (t, *J* = 7.2 Hz, 3H); ^{13}C NMR (100 MHz, $CDCl_3$): δ 166.31, 163.04, 151.26, 139.13, 138.65, 133.72, 133.23, 131.27, 130.83, 130.63, 129.79, 129.19, 127.11, 126.33, 125.75, 124.24, 61.77, 36.73, 32.40, 23.10, 14.39, 14.13; HRMS (ESI+): *m/z* calcd for $C_{24}H_{27}N_2O_4S$ $[M+H]^+$: 439.1692; Found: 439.1696.

Compound 10d: ethyl (1*R*,4*S*)-2-(3-(ethoxycarbonyl)-5-(2-(phenylsulfonamido)phenyl)pyridin-2-yl)-7-azabicyclo[2.2.1]heptane-7-carboxylate

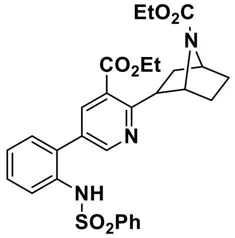

10d
Epibatidine analog

white solid; Yield: 60% (66 mg); ^{1}H NMR (500 MHz, CD_2Cl_2): δ 8.22 (d, *J* = 2.0 Hz, 1H), 7.82–7.69 (m, 1H), 7.60 (d, *J* = 8.0 Hz, 1H), 7.57–7.49 (m, 3H), 7.44–7.34 (m, 3H), 7.26 (t, *J* = 7.5, 1.5 Hz, 1H), 7.14 (dd, *J* = 7.5, 1.5 Hz, 1H), 6.80 (br. s, 1H), 4.47–4.37 (m, 3H), 4.32 (s, 1H), 4.04–3.94 (m, 1H), 3.86 (dd, *J* = 8.5, 5.0 Hz, 1H), 2.95–2.78 (m, 1H), 1.92–1.82 (m, 2H), 1.81–1.76 (m, 1H), 1.70 (dd, *J* = 12.0, 8.5 Hz, 1H), 1.61–1.52 (m, 1H), 1.42 (t, *J* = 7.1 Hz, 3H), 1.34–0.90 (m, 4H); ^{13}C NMR (125 MHz, CD_2Cl_2): δ 166.54, 162.27, 155.92, 150.83, 139.49, 138.97, 134.12, 133.46, 131.84, 131.17, 131.15, 129.87, 129.48, 127.38, 126.57, 125.94, 124.55, 63.05, 62.07, 60.99, 56.90, 47.84, 34.46, 30.08, 29.85, 14.83, 14.51; HRMS (ESI+): *m/z* calcd for $C_{29}H_{32}N_3O_6S$ $[M+H]^+$: 550.2012; Found: 550.2019.

Compound 10e: 2-chloro-*N*-(4-chloro-3-(3-((phenylperoxy)thio)-5-(2-(phenylsulfonamido)phenyl) pyridin-2-yl)phenyl)-4-(methylsulfonyl)benzamide

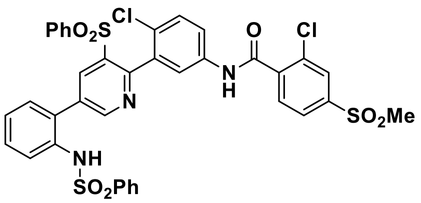

10e
Vismodegib analog

White solid; Yield: 33% (11 mg); ^{1}H NMR (500 MHz, CD_2Cl_2): δ 8.57 (s, 1H), 8.48 (d, *J* = 2.0 Hz, 1H), 8.42 (d, *J* = 2.0 Hz, 1H), 7.95 (d, *J* = 1.5 Hz, 1H), 7.83 (dd, *J* = 8.0, 2.0 Hz, 1H), 7.79–7.74 (m, 2H), 7.56–7.50 (m, 3H), 7.50–7.45 (m, 1H), 7.41–7.36 (m, 2H), 7.36–7.26 (m, 9H), 7.18 (d, *J* = 9.0 Hz, 1H), 7.06 (s, 1H), 3.00 (s, 3H); ^{13}C NMR (125 MHz, CD_2Cl_2): δ 163.67, 154.55, 153.01, 143.74, 140.66, 139.70, 139.48, 138.17, 137.31, 136.65, 136.08, 134.88, 134.18, 133.94, 133.82, 132.66, 132.61, 131.44, 131.07, 130.52, 130.10, 129.72, 129.70, 129.66, 129.47, 128.59, 127.66, 127.47, 126.55, 126.44, 123.41, 122.40, 44.70; HRMS (ESI+): *m/z* calcd for $C_{37}H_{28}Cl_2N_3O_7S_3$ $[M+H]^+$: 792.0466; Found: 792.0478.

Compound 10f: ethyl 5-(6-hydroxybenzo[*d*][1,3]dioxol-5-yl)-2-methylnicotinate

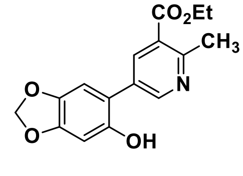

10f
Sesamol:*sub*-nicotinate

Off-white solid; Yield: 65% (36 mg); ^{1}H NMR (500 MHz, DMSO-d_6): δ 9.59 (s, 1H), 8.72 (d, *J* = 2.5 Hz, 1H), 8.29 (d, *J* = 2.0 Hz, 1H), 6.97 (s, 1H), 6.59 (s, 1H), 5.98 (s, 2H), 4.33 (q, *J* = 7.0 Hz, 2H), 2.71 (s, 3H), 1.33 (t, *J* = 7.0 Hz, 3H); ^{13}C NMR (125 MHz, DMSO-d_6): δ 166.27, 155.60, 151.40, 149.65, 147.74, 140.65, 137.81, 131.63, 124.49, 114.72, 108.86, 101.07, 98.15, 61.02, 23.94, 14.06; HRMS (ESI+): *m/z* calcd for $C_{16}H_{16}NO_5$ $[M+H]^+$: 302.1028; Found: 302.1025.

Compound 10g: ethyl 5-(8-hydroxy-2,3,6,7-tetrahydro-1*H*,5*H*-pyrido[3,2,1-*ij*]quinolin-9-yl)-2-methylnicotinate

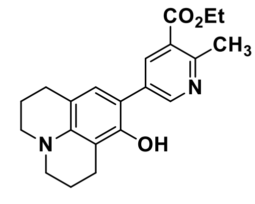

10g
Julolidine:*sub*-nicotinate

brown solid; Yield: 58% (41 mg); ^{1}H NMR (500 MHz, $CDCl_3$): δ 8.63 (d, *J* = 2.0 Hz, 1H), 8.23 (d, *J* = 2.5 Hz, 1H), 6.68 (s, 1H), 6.47 (s, 1H), 4.35 (q, *J* = 7.0 Hz, 2H), 3.15 (dt, *J* = 8.0, 5.0 Hz, 4H), 2.75 (q, *J* = 7.0 Hz, 4H), 2.66 (s, 3H), 2.05–1.96 (m, 4H), 1.38 (t, *J* = 7.0 Hz, 3H); ^{13}C NMR (125 MHz, $CDCl_3$): δ 166.54, 156.69, 151.98, 149.54, 144.32, 138.81, 132.59, 127.75, 125.46, 114.58, 111.82, 108.70, 61.39, 50.20, 49.48, 27.26, 24.12, 22.38, 21.71, 21.57, 14.37; HRMS (ESI+): *m/z* calcd for $C_{21}H_{25}N_2O_3$ $[M+H]^+$: 353.1865; Found: 353.1866.

Compound 10h: ethyl 5-(5-acetamido-2-hydroxyphenyl)-2-methylnicotinate

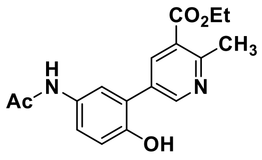

10h

Paracetamol: *sub*-nicotinate

Off-white solid; Yield: 51% (32 mg); ^{1}H NMR (400 MHz, CD_3OD): δ 8.77 (d, *J* = 2.0 Hz, 1H), 8.47 (d, *J* = 2.4 Hz, 1H), 7.50 (d, *J* = 2.8 Hz, 1H), 7.38 (dd, *J* = 8.8, 2.4 Hz, 1H), 6.89 (d, *J* = 8.4 Hz, 1H), 4.39 (q, *J* = 7.2 Hz, 2H), 2.80 (s, 3H), 2.11 (s, 3H), 1.40 (t, *J* = 7.2 Hz, 3H); ^{13}C NMR (100 MHz, CD_3OD): δ 171.46, 167.69, 158.19, 152.64, 152.22, 140.30, 133.70, 132.48, 126.90, 124.54, 123.51, 123.49, 117.23, 62.58, 23.91, 23.58, 14.53; HRMS (ESI+): *m/z* calcd for $C_{17}H_{19}N_2O_4$ $[M+H]^+$: 315.1345; Found: 315.1345.

VI. Supplementary References

1. Varun, B. V., Vaithegi, K., Yi, S. & Park, S. B. Nature-inspired remodeling of (aza)indoles to meta-aminoaryl nicotinates for late-stage conjugation of vitamin B3 to (hetero)arylamines. *Nat. Commun. 2020 111* **11**, 1–9 (2020).

2. Kil'Met'Ev, A. S., Shul'Ts, E. E., Shakirov, M. M., Rybalova, T. V. & Tolstikov, G. A. Diels-alder reactions with ethyl 1-benzofuran-3-carboxylates. *Russ. J. Org. Chem.* **49**, 872–885 (2013).

3. Costi, R. *et al.* New nucleotide-competitive non-nucleoside inhibitors of terminal deoxynucleotidyl transferase: Discovery, characterization, and crystal structure in complex with the target. *J. Med. Chem.* **56**, 7431–7441 (2013).

4. Echeverry-Gonzalez, C. A., Ortiz Villamizar, M. C. & Kouznetsov, V. V. The remarkable selectivity of the 2-arylquinoline-based acyl hydrazones toward copper salts: exploration of their catalytic applications in the copper catalysed N -arylation of indole derivatives and C1-alkynylation of tetrahydroisoquinolines via the A 3 reaction. *New J. Chem.* **45**, 243–250 (2021).

5. Ando, K. *et al.* Preparation of 2-, 3-, 4- and 7-(2-alkylcarbamoyl-1-alkylvinyl)benzo[b]furans and their BLT 1 and/or BLT 2 inhibitory activities. *Org. Biomol. Chem.* **6**, 296–307 (2008).

6. Ahmed, S. A., Hinz, D. J., Jellen, M. J. & Hossain, M. M. A Concise Synthesis of Potential COX Inhibitor BRL-37959 and Analogs Involving Bismuth(III) Catalyzed Friedel−Crafts Acylation. *Chem. Biodivers.* **15**, e1800334 (2018).

7. He, S. *et al.* Facile functionalization at the C2 position of a highly substituted benzofuran. *Tetrahedron Lett.* **55**, 2212–2216 (2014).

8. Fowler, K. J., Ellis, J. L. & Morrow, G. W. 6-endo heck cyclization of 3-(2-iodophenoxy)methylbenzofurans: A useful approach to pterocarpenes. *Synth. Commun.* **43**, 1676–1682 (2013).

9. Buravlev, E. V., Shevchenko, O. G. & Suponitsky, K. Y. Synthesis and Antioxidant Capacity of Some Derivatives of Sesamol at the C-6 Position. *Chem. Biodivers.* **18**, e2100221 (2021).

10. Ortiz Zacarías, N. V. *et al.* Synthesis and Pharmacological Evaluation of Triazolopyrimidinone Derivatives as Noncompetitive, Intracellular Antagonists for CC Chemokine Receptors 2 and 5. *J. Med. Chem.* **62**, 11035–11053 (2019).

11. Che, D. *et al.* exo-2-(pyridazin-4-yl)-7-azabicyclo[2.2.1]heptanes: Syntheses and nicotinic acetylcholine receptor agonist activity of potent pyridazine analogues of (±)-epibatidine. *J. Med. Chem.* **44**, 47–57 (2001).

12. Hiraoka, C. *et al.* Screening, substrate specificity and stereoselectivity of yeast strains, which reduce sterically hindered isopropyl ketones. *Tetrahedron: Asymmetry* **17**, 3358–3367 (2006).

13. Li, L. *et al.* Silver-Catalyzed Oxidative C(sp3)−P Bond Formation through C−C and P−H Bond Cleavage. *Angew. Chemie Int. Ed.* **56**, 10539–10544 (2017).

14. Chen, G. *et al.* Synthesis and antiproliferative evaluation of new zampanolide mimics. *Org. Biomol. Chem.* **17**, 3830–3844 (2019).
