# Supplementary material for: Synthesis of substituted pyridines with diverse functional groups via the remodeling of (Aza)indole/Benzofuran skeletons
Source: Commun Chem. 2023 Jun 7;6:112. doi: 10.1038/s42004-023-00914-5 (PMC10247795; doi:10.1038/s42004-023-00914-5)
Supplement: Supplementary file 3 — Description of Additional Supplementary Files [file 42004_2023_914_MOESM3_ESM.pdf]

Description of Additional Supplementary Files

File name: Supplementary Data 1

Description: NMR spectra for 1 – ^{1}H, ^{13}C, ^{31}P
