# Supplementary material for: Synthesis of substituted pyridines with diverse functional groups via the remodeling of (Aza)indole/Benzofuran skeletons
Source: Commun Chem. 2023 Jun 7;6:112. doi: 10.1038/s42004-023-00914-5 (PMC10247795; doi:10.1038/s42004-023-00914-5)
Supplement: Supplementary file 4 — Supplementary Data 1 [file 42004_2023_914_MOESM4_ESM.pdf]

^{1}H, ^{13}C, and ^{31}P NMR Spectra

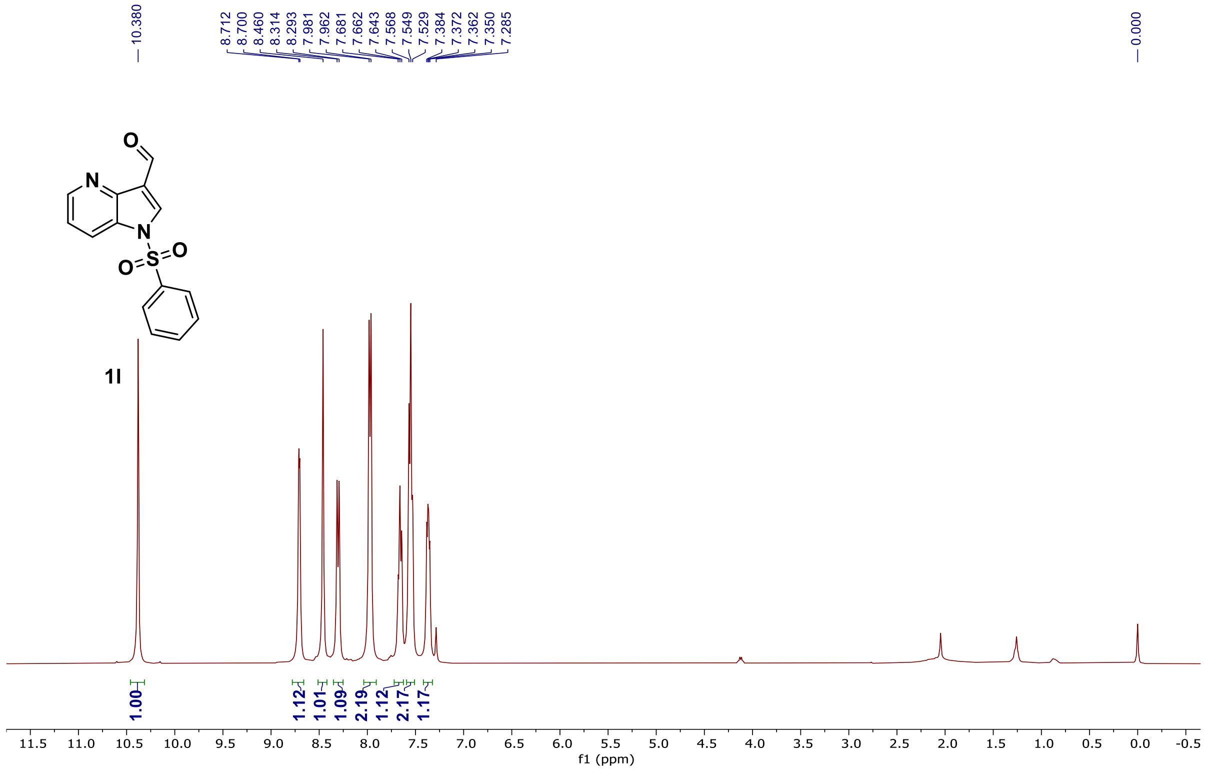

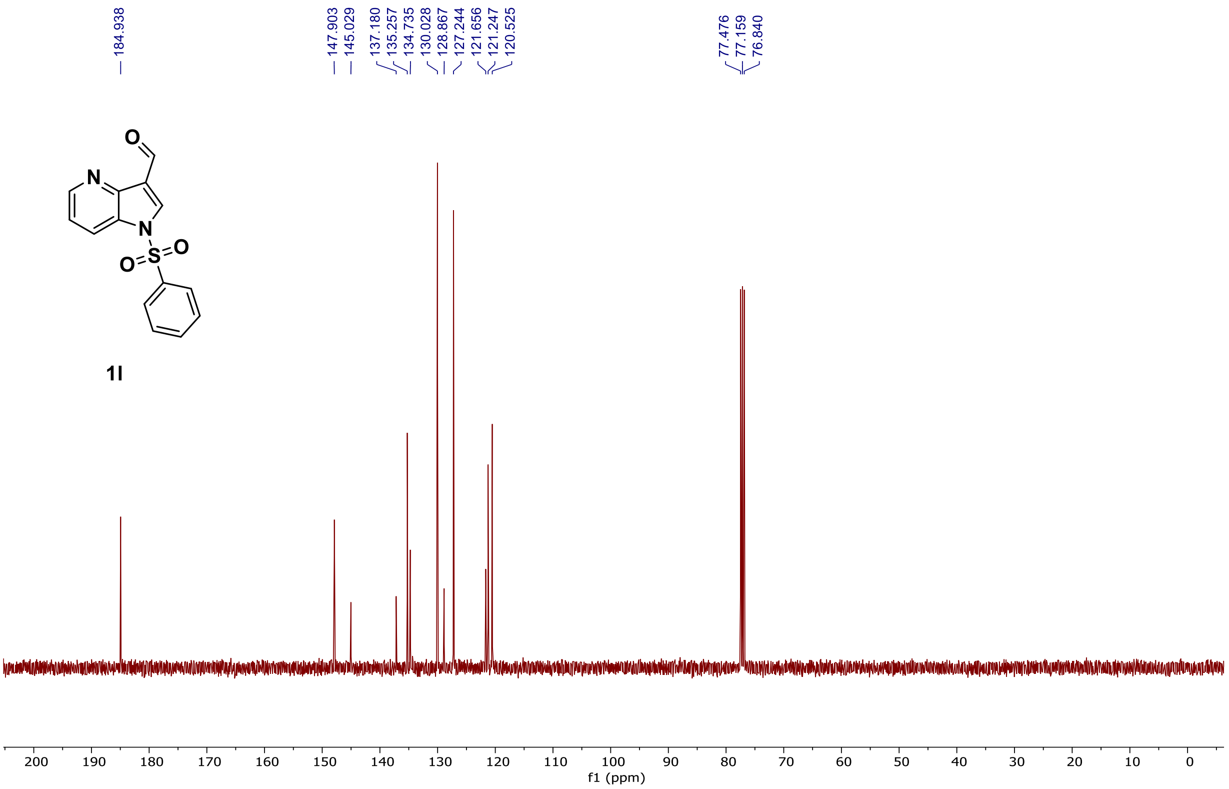

^{1}H and ^{13}C NMR Spectrum of **1l** in $CDCl_3$

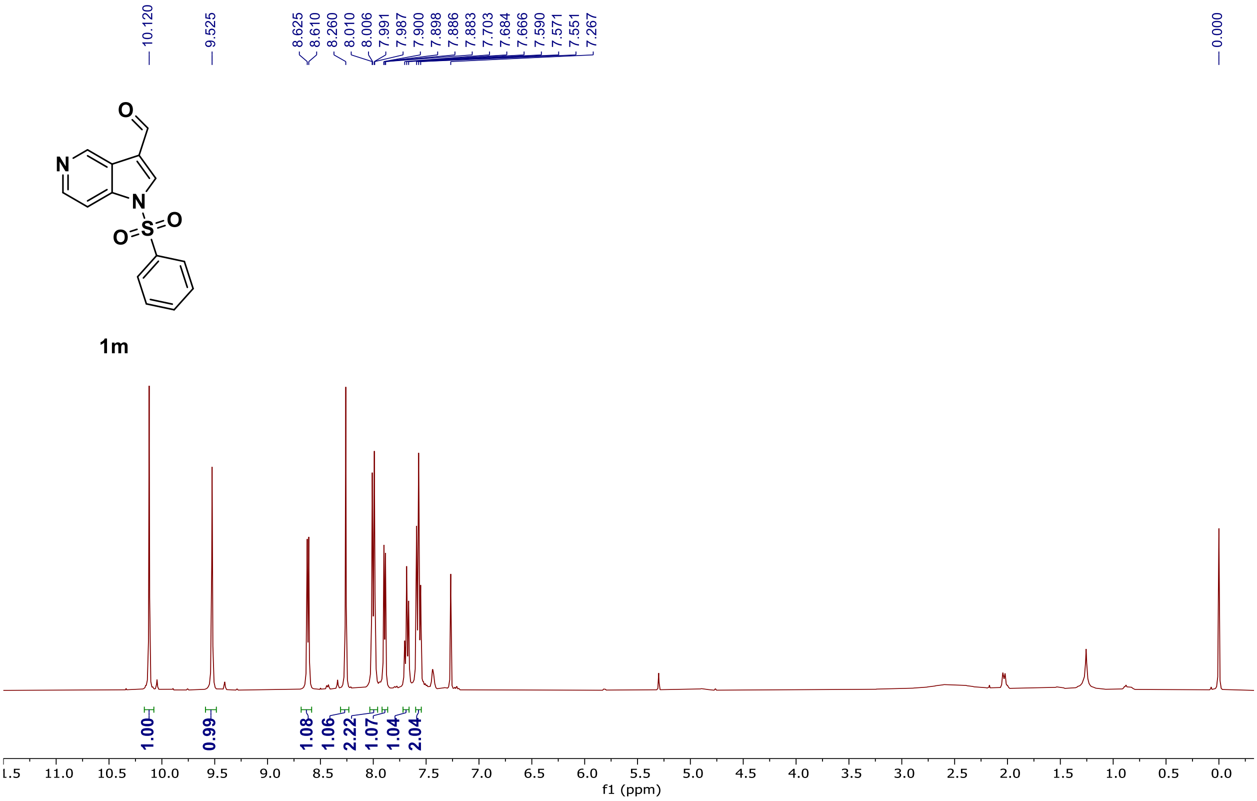

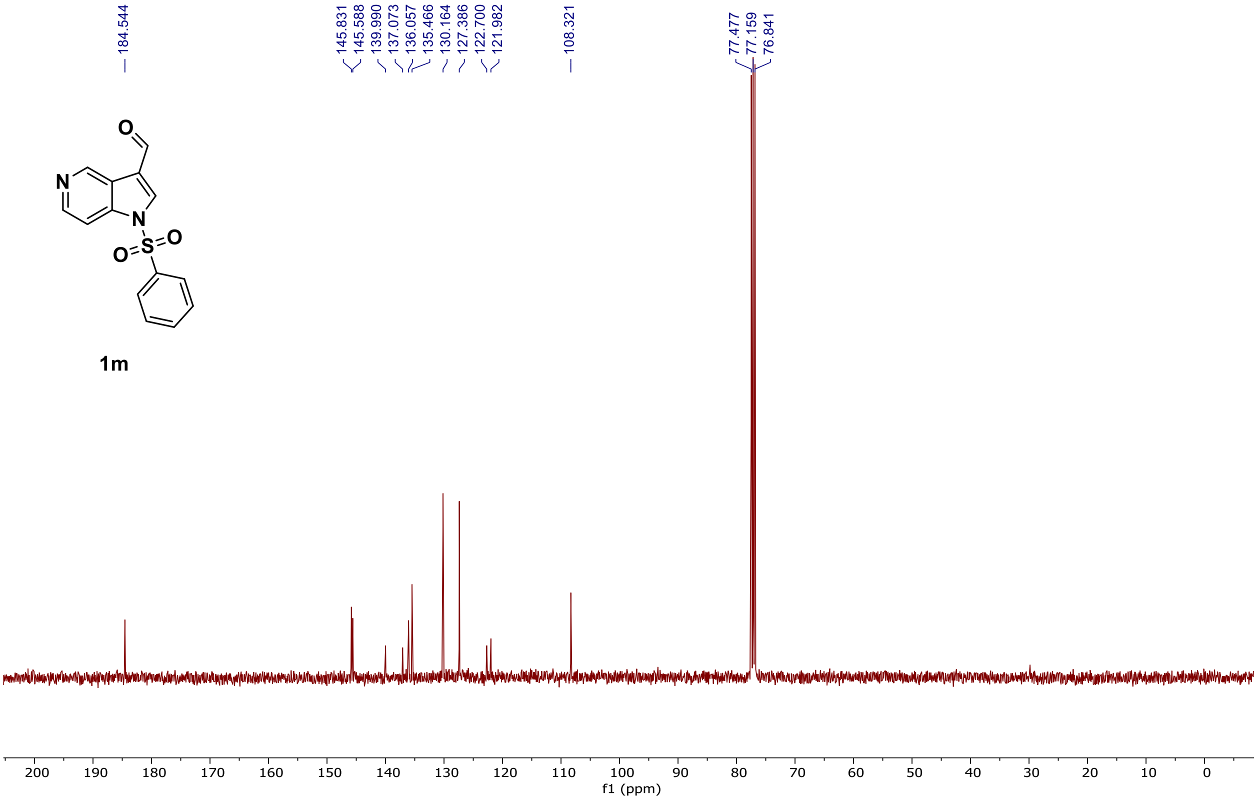

^{1}H and ^{13}C NMR Spectrum of **1m** in $CDCl_3$

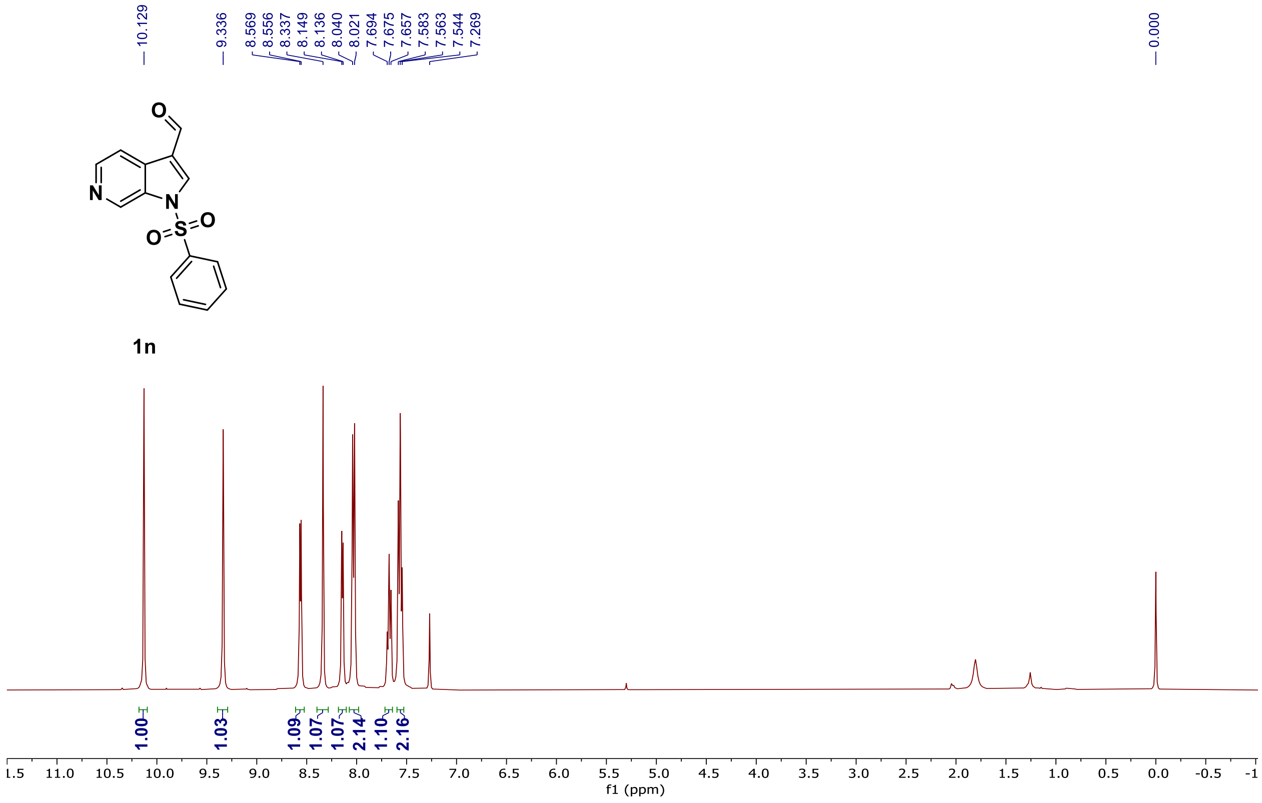

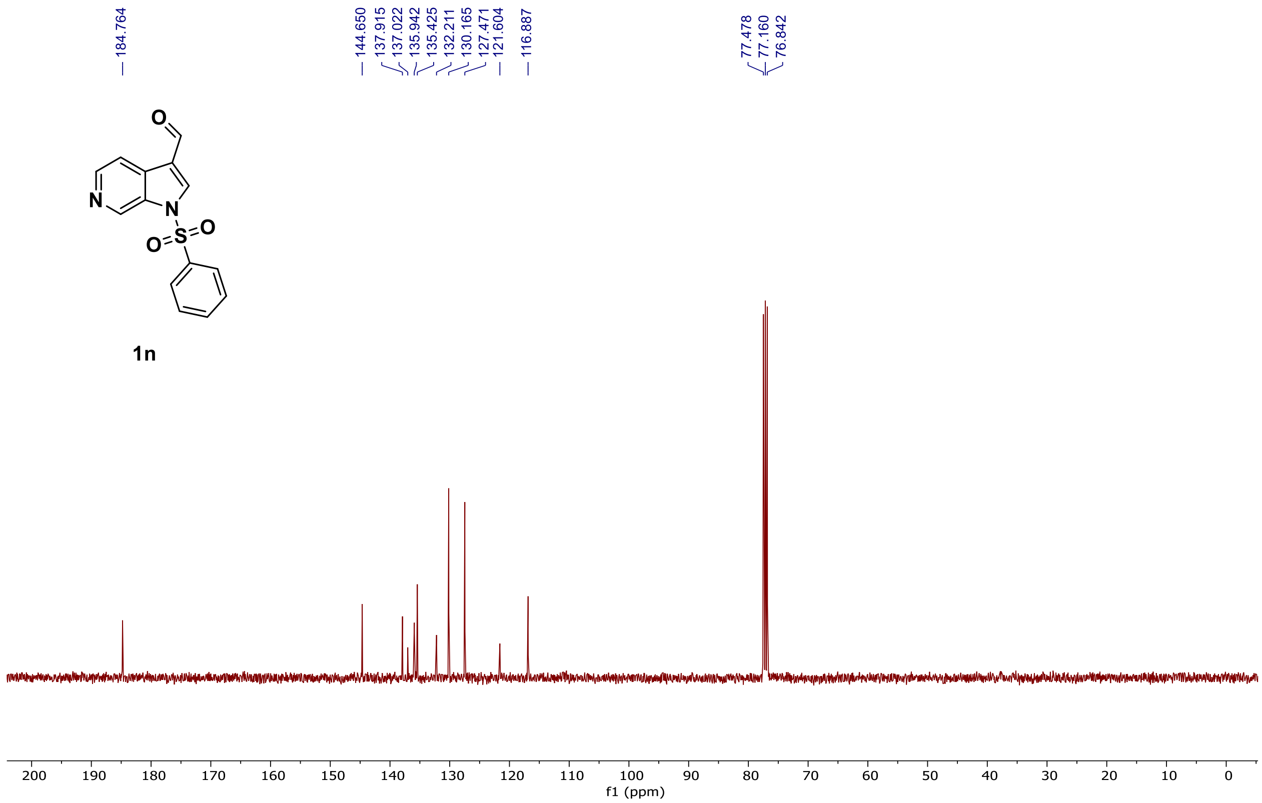

^{1}H and ^{13}C NMR Spectrum of **1n** in $CDCl_3$

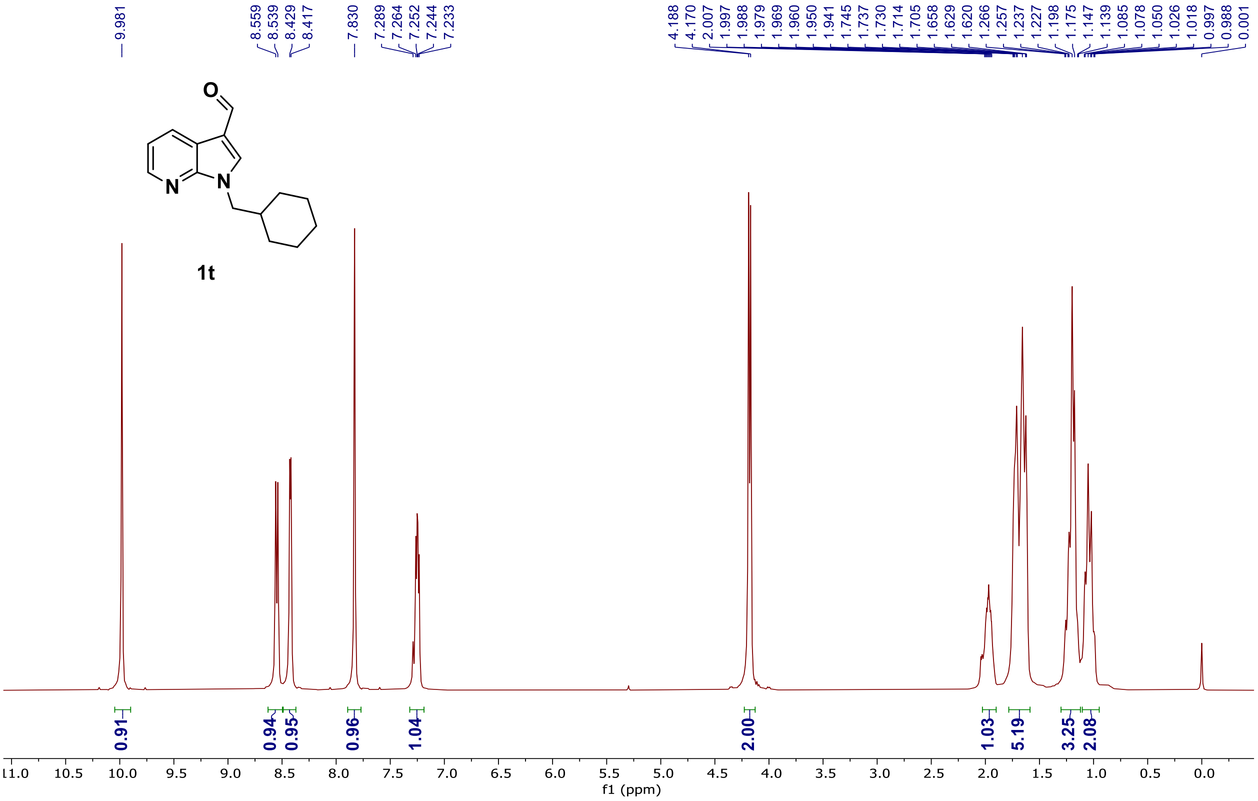

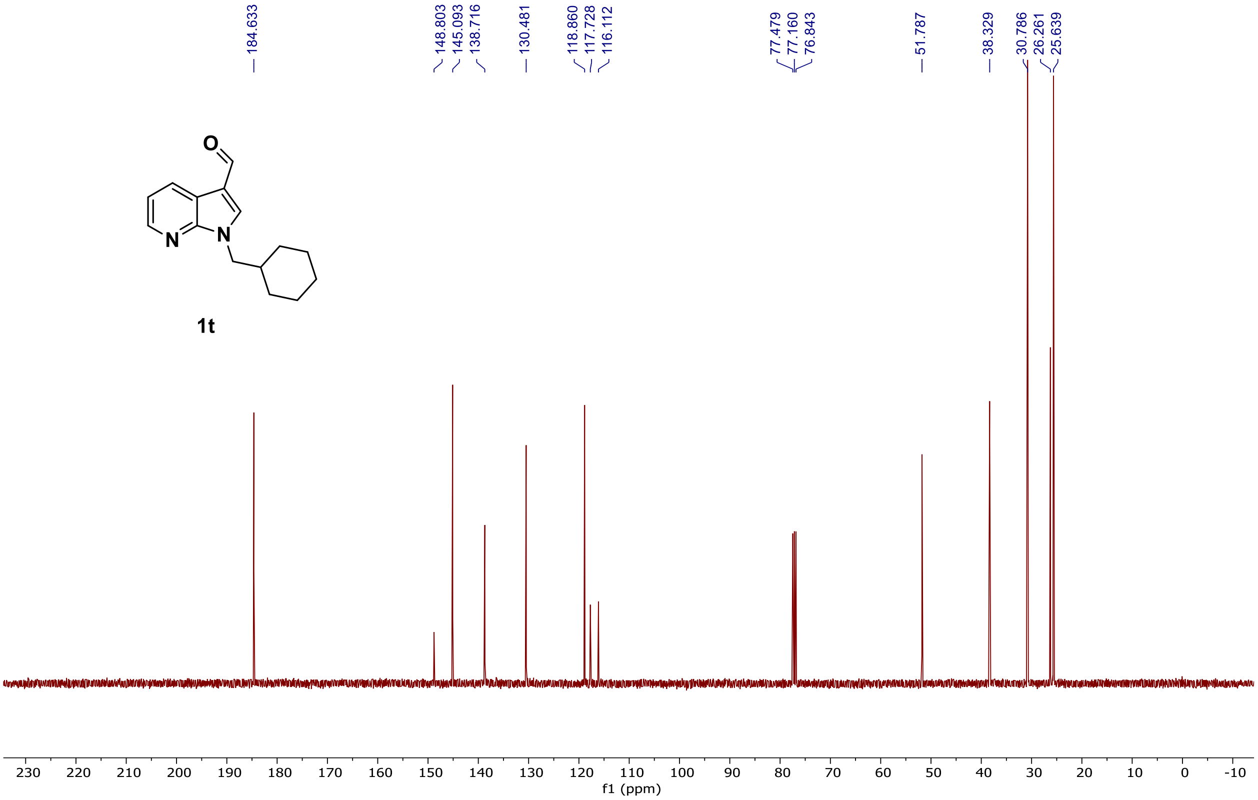

^{1}H and ^{13}C NMR Spectrum of **1t** in $CDCl_3$

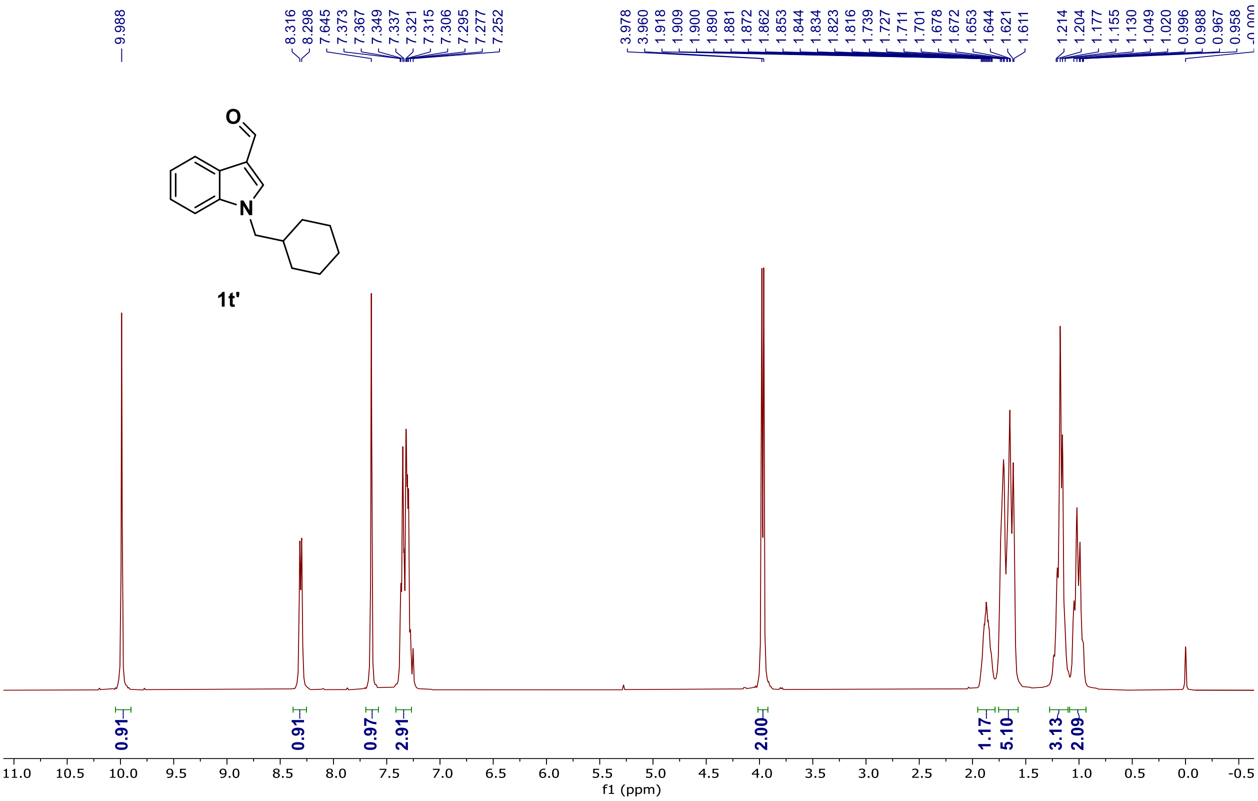

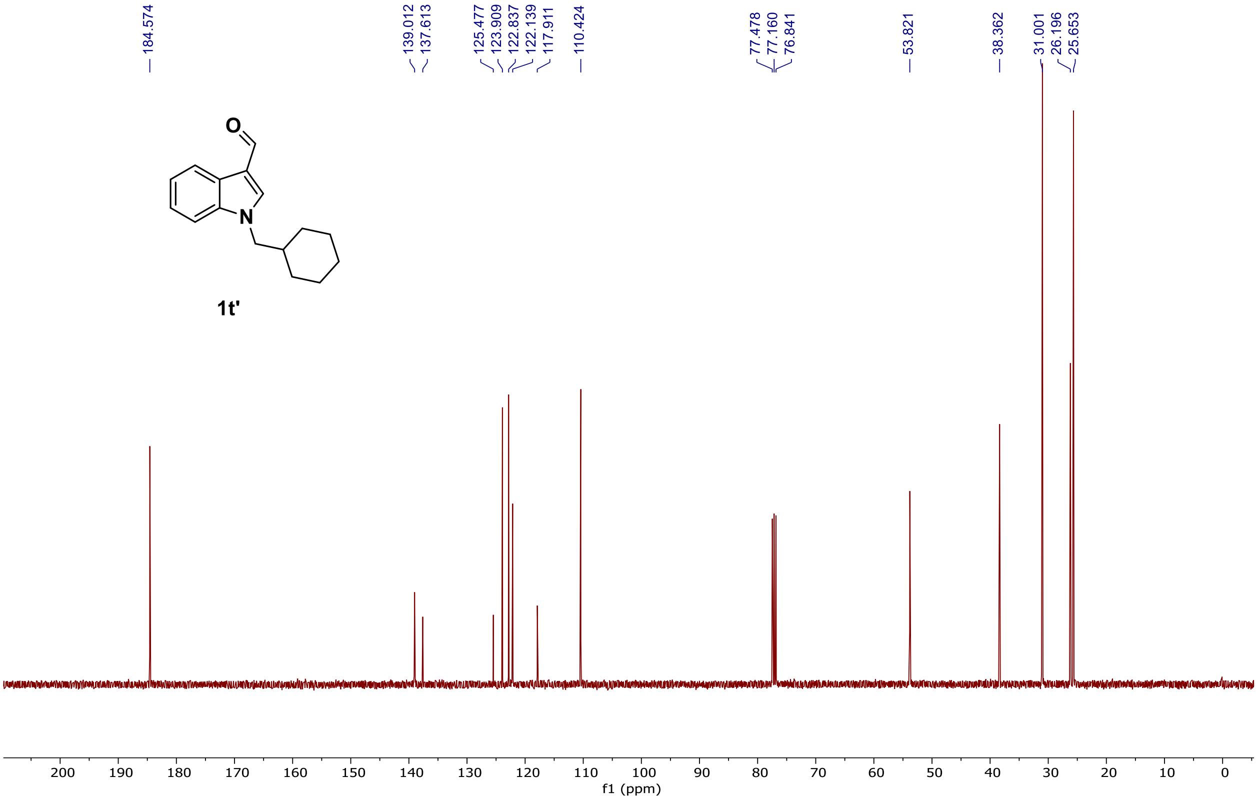

^{1}H and ^{13}C NMR Spectrum of **1t'** in $CDCl_3$

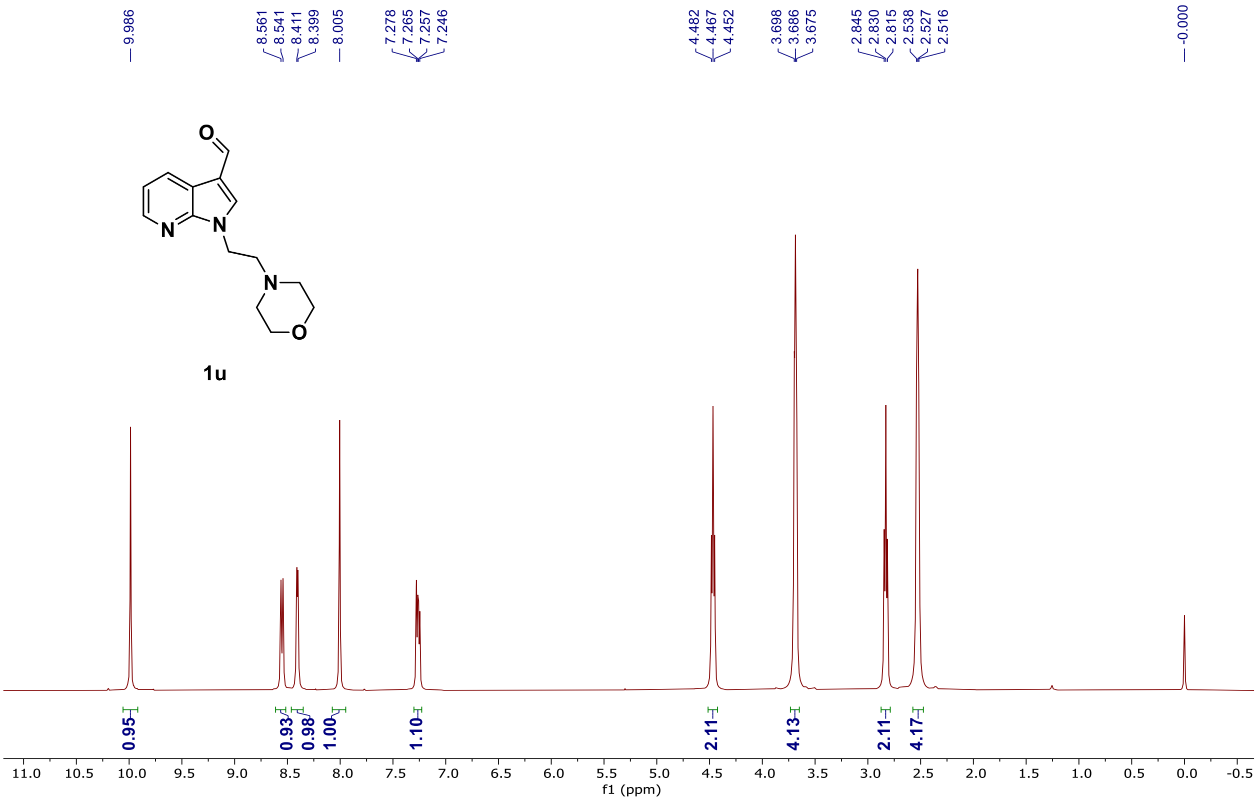

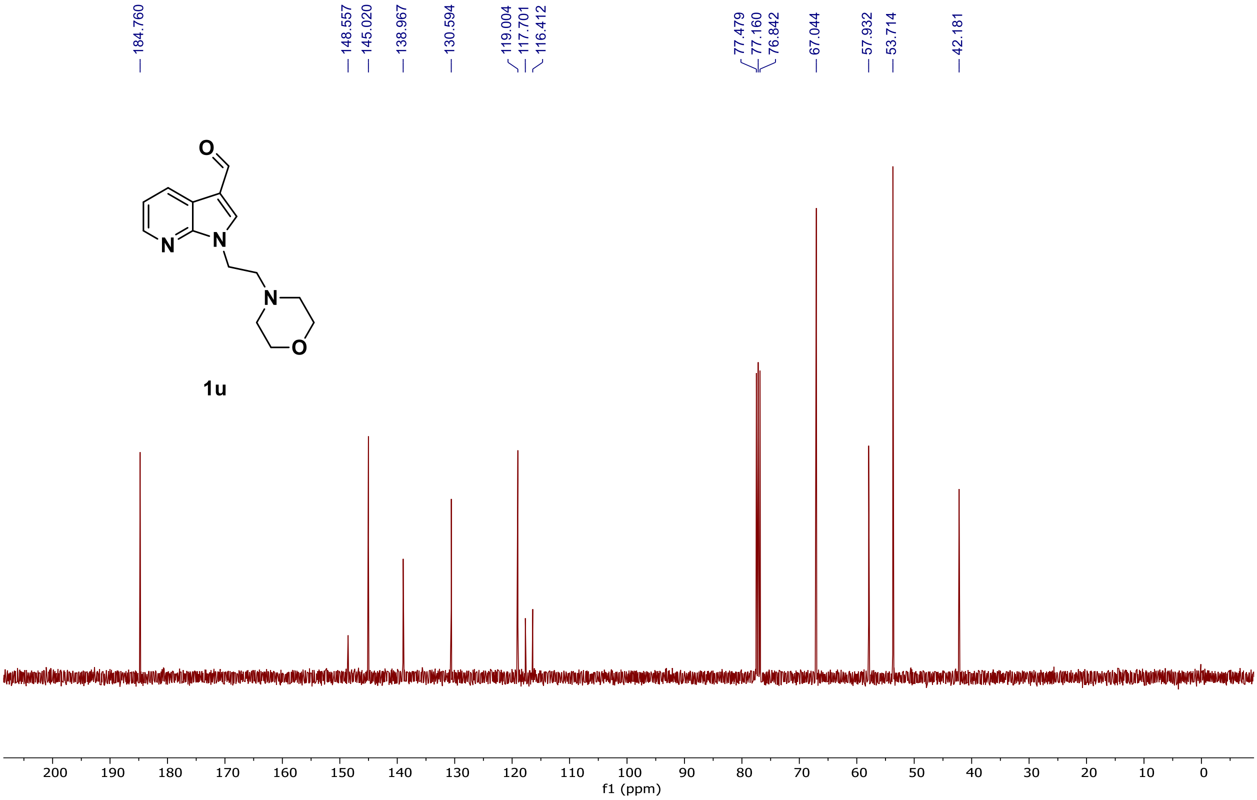

^{1}H and ^{13}C NMR Spectrum of **1u** in $CDCl_3$

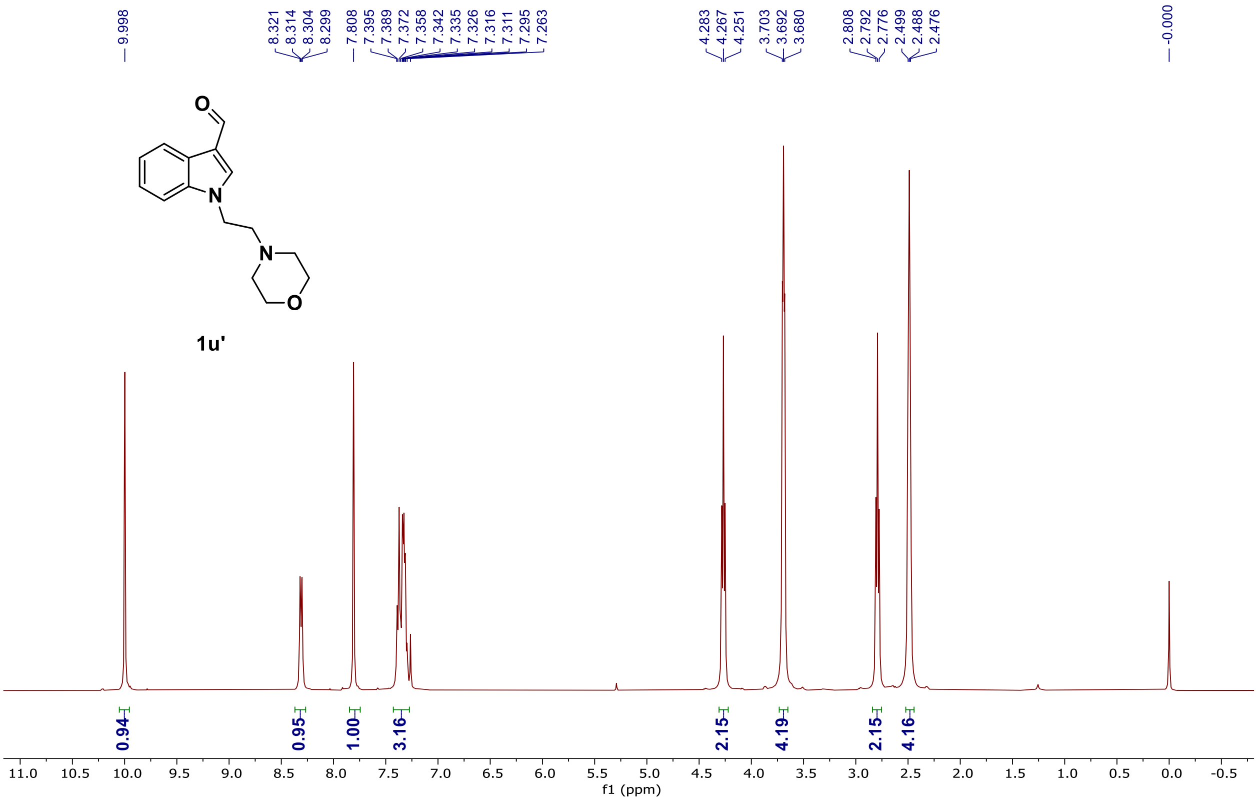

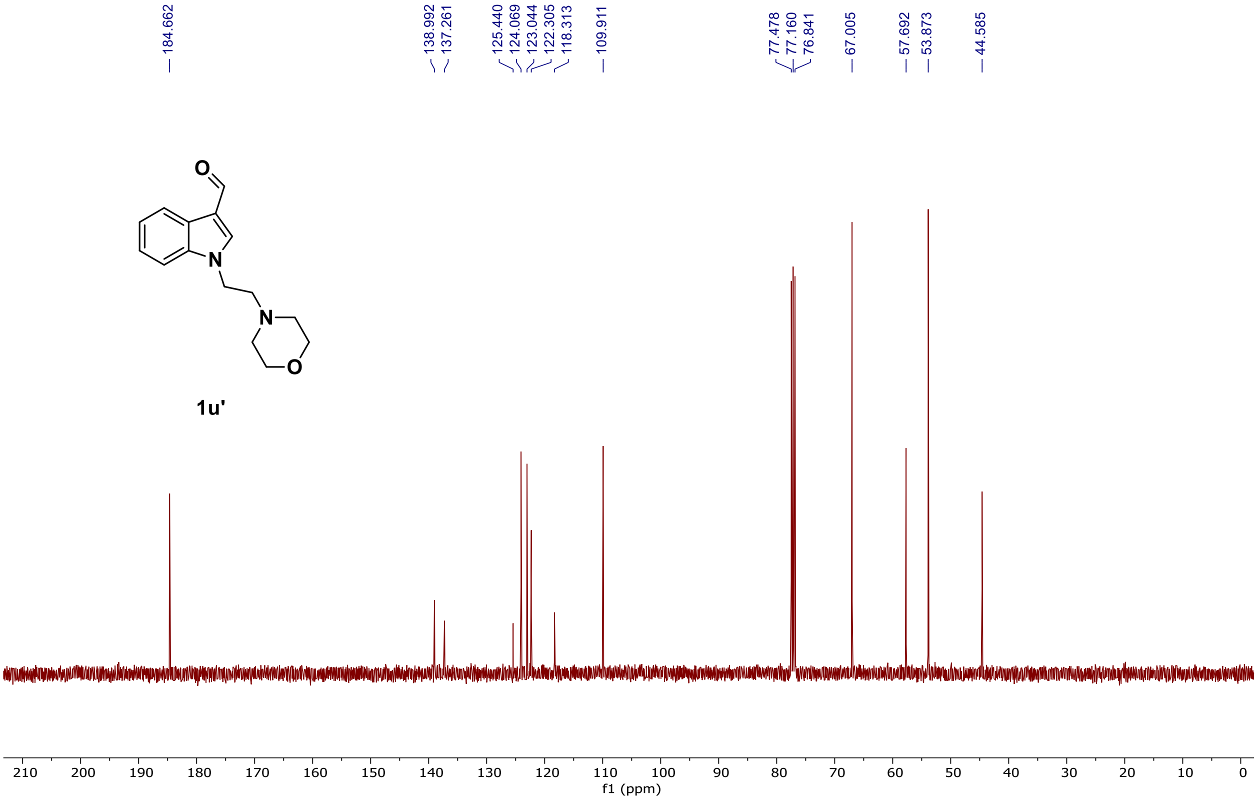

^{1}H and ^{13}C NMR Spectrum of **1u'** in $CDCl_3$

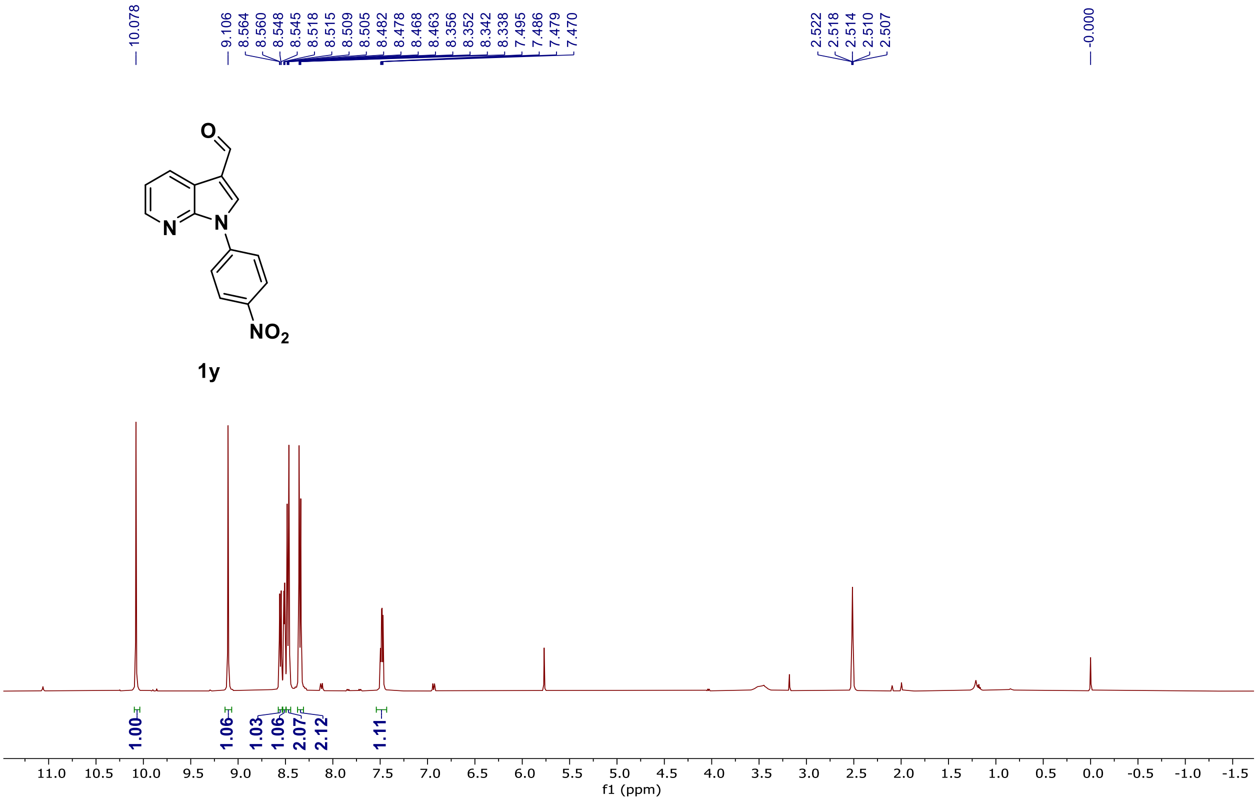

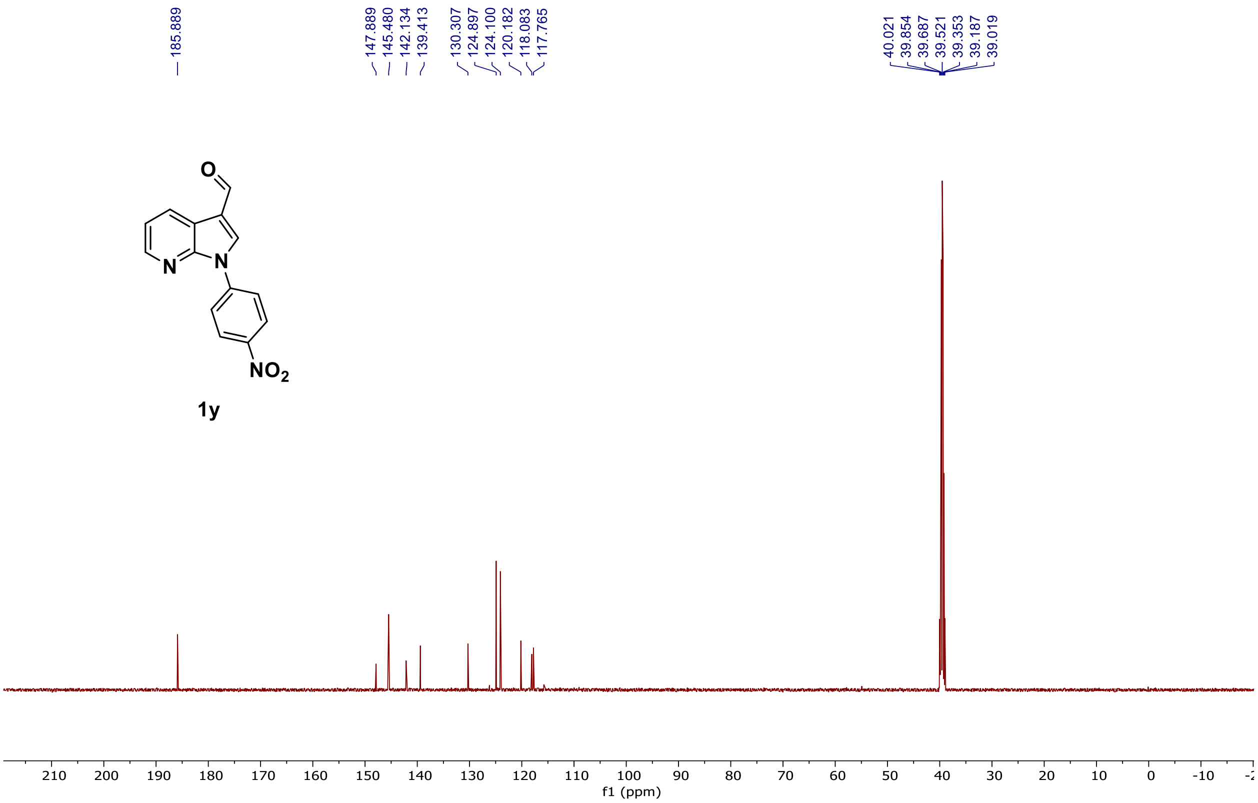

^{1}H and ^{13}C NMR Spectrum of **1y** in DMSO-d_6

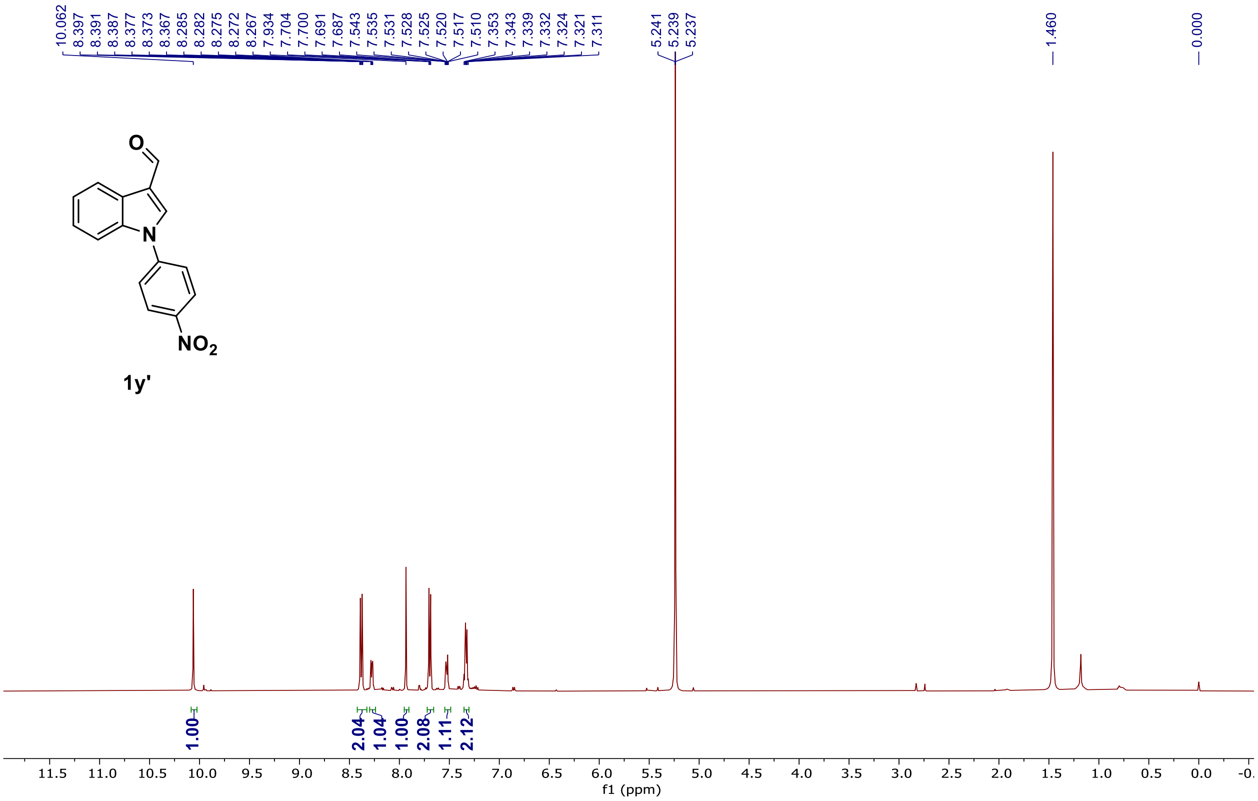

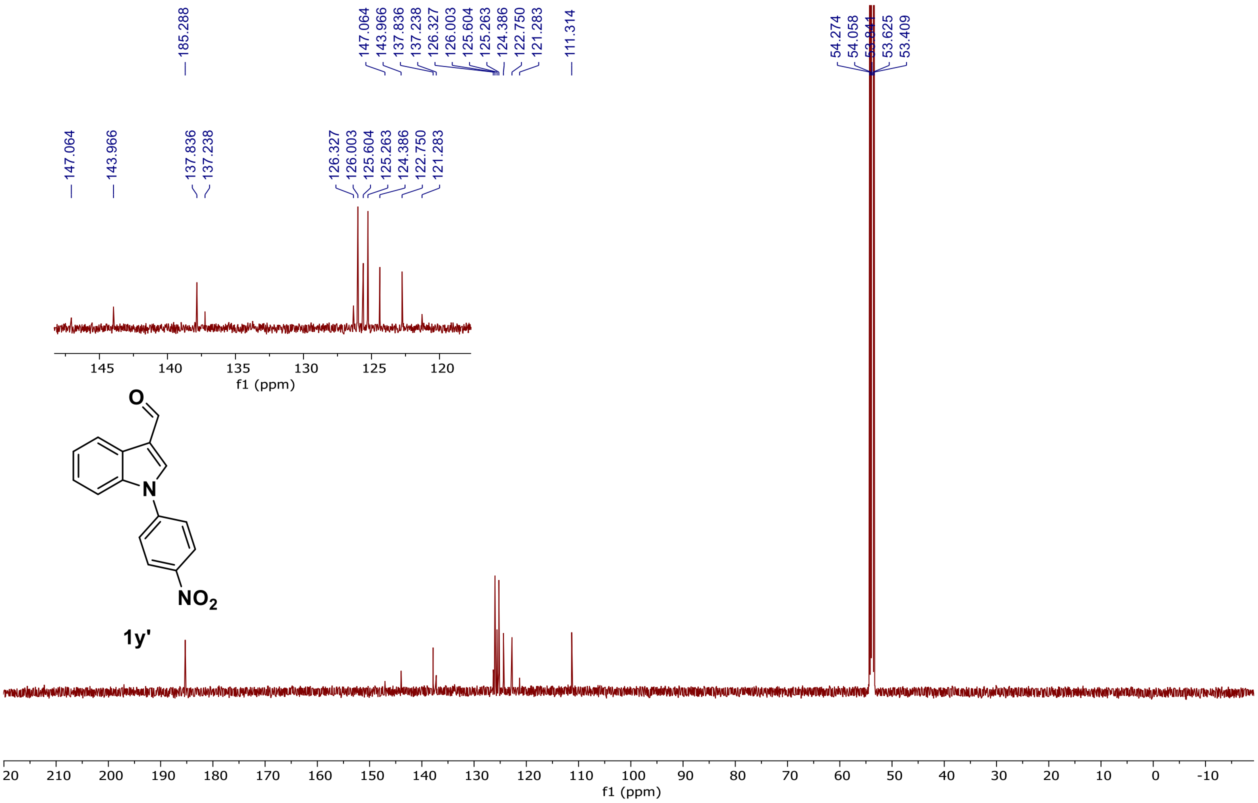

^{1}H and ^{13}C NMR Spectrum of **1y'** in CD_2Cl_2

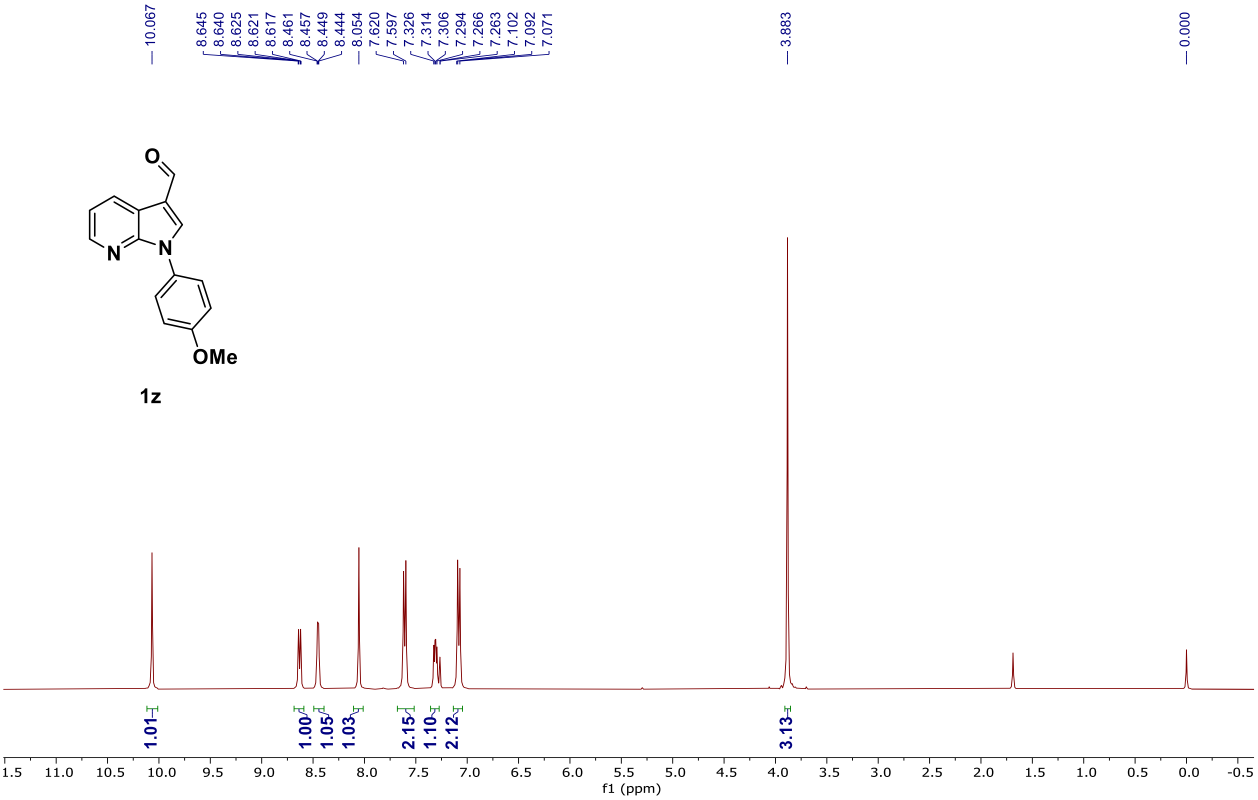

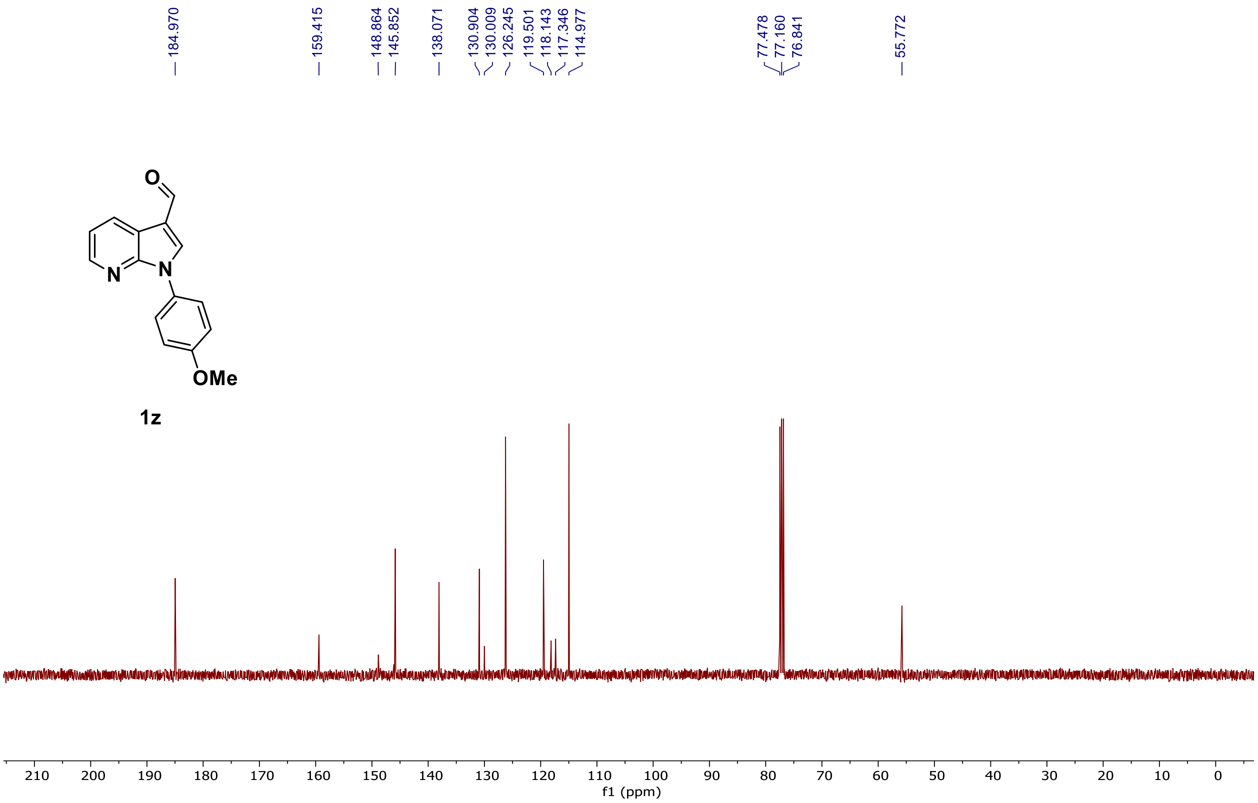

^{1}H and ^{13}C NMR Spectrum of **1z** in $CDCl_3$

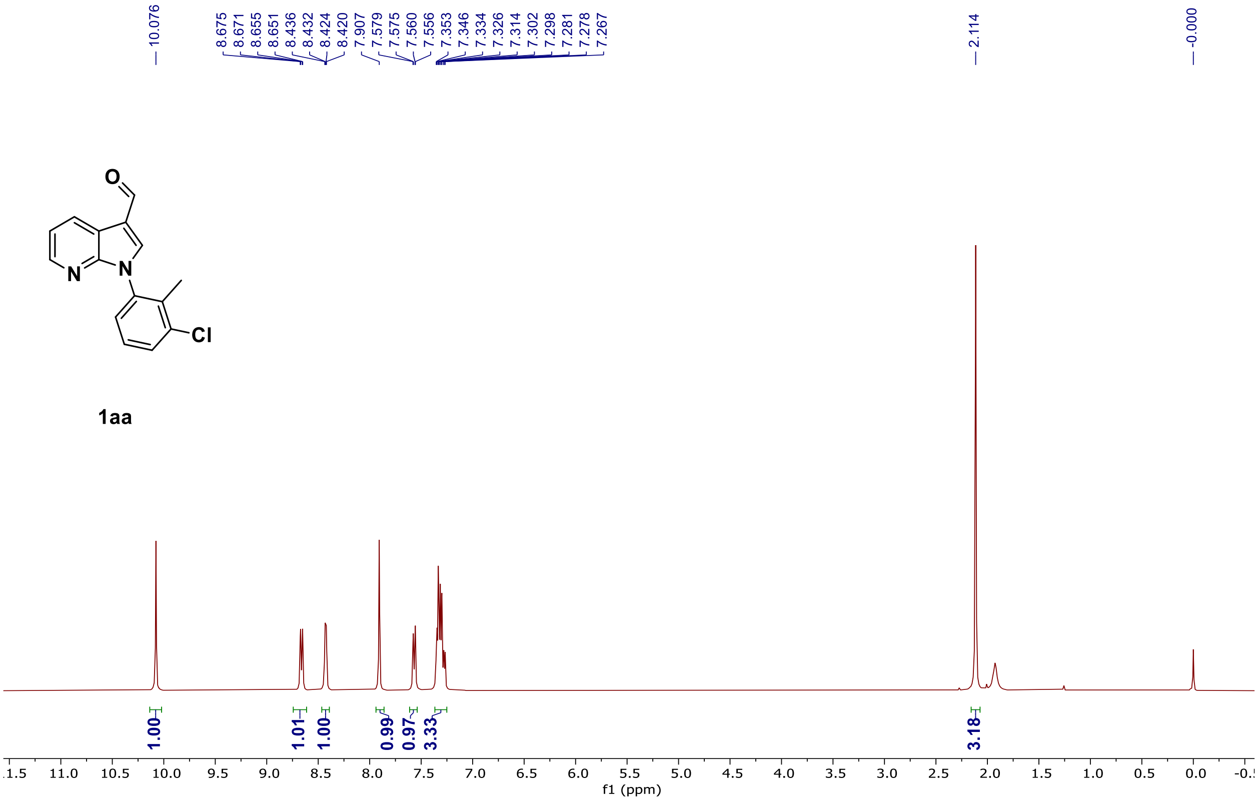

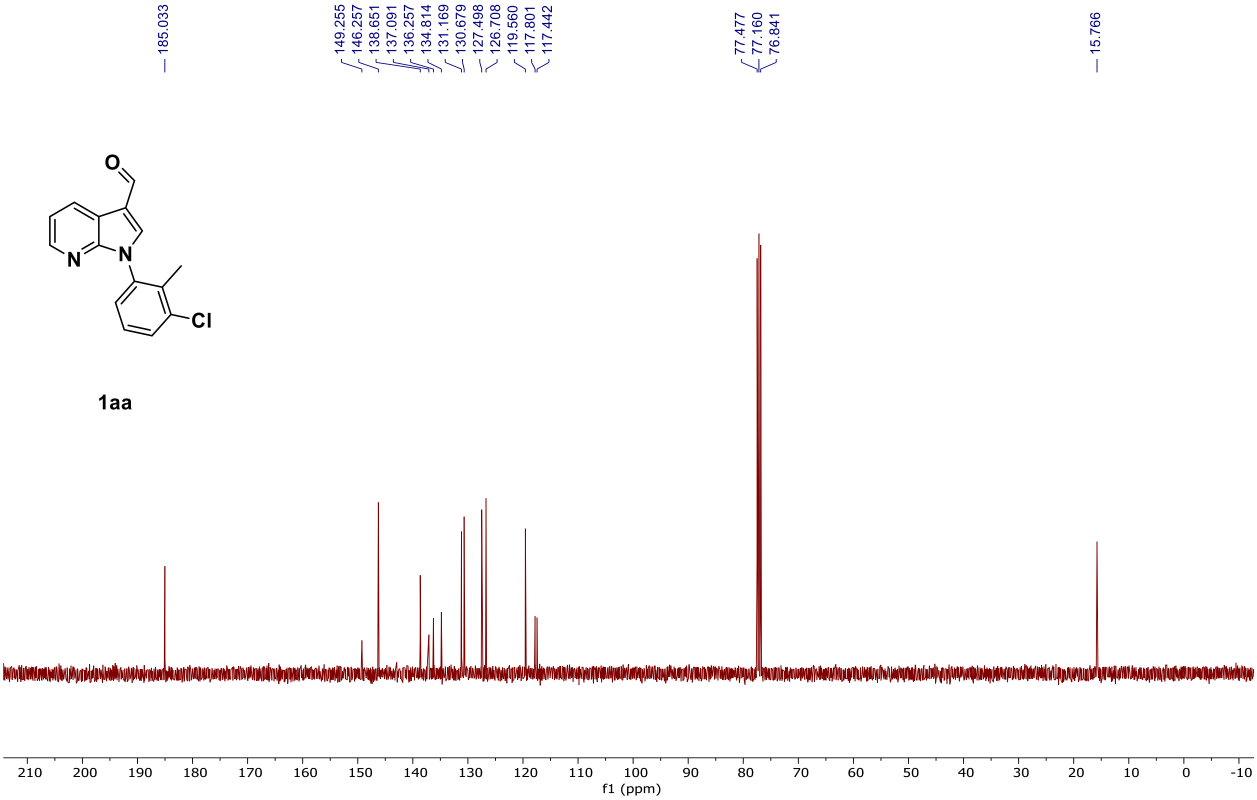

^{1}H and ^{13}C NMR Spectrum of **1aa** in $CDCl_3$

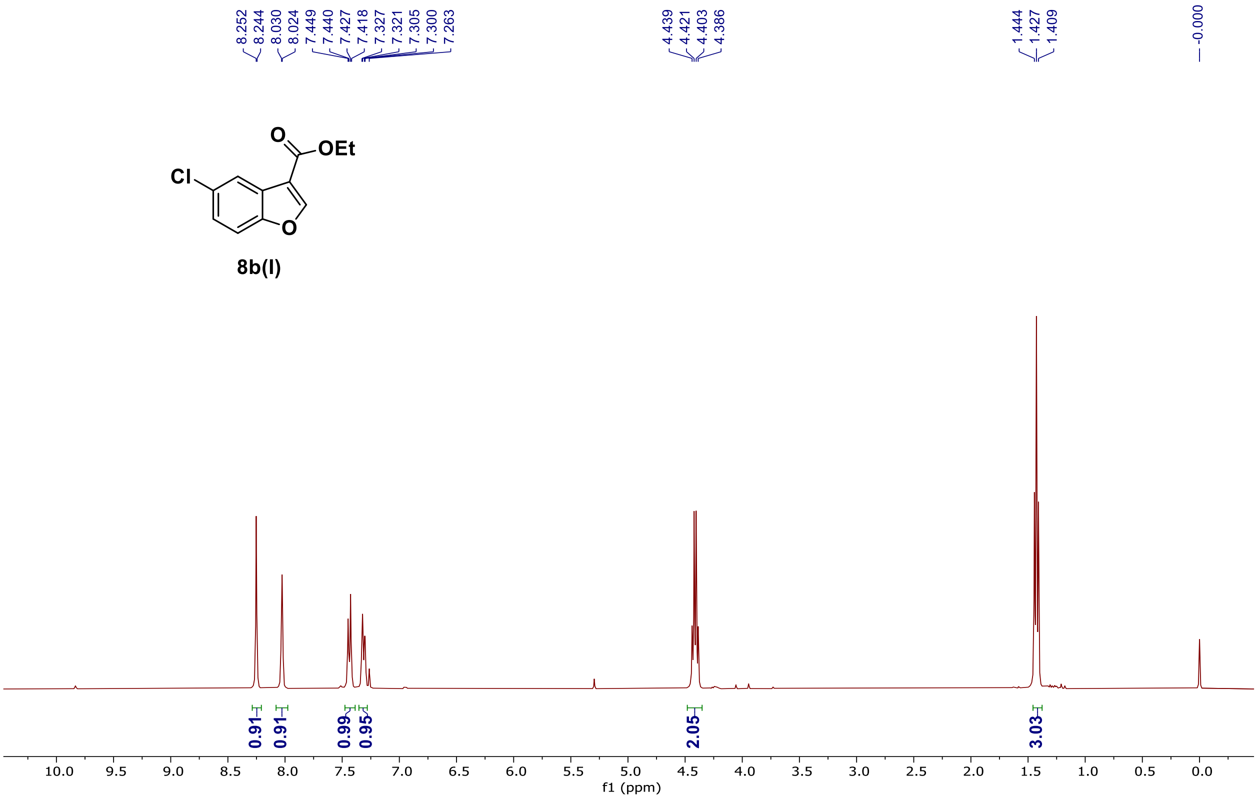

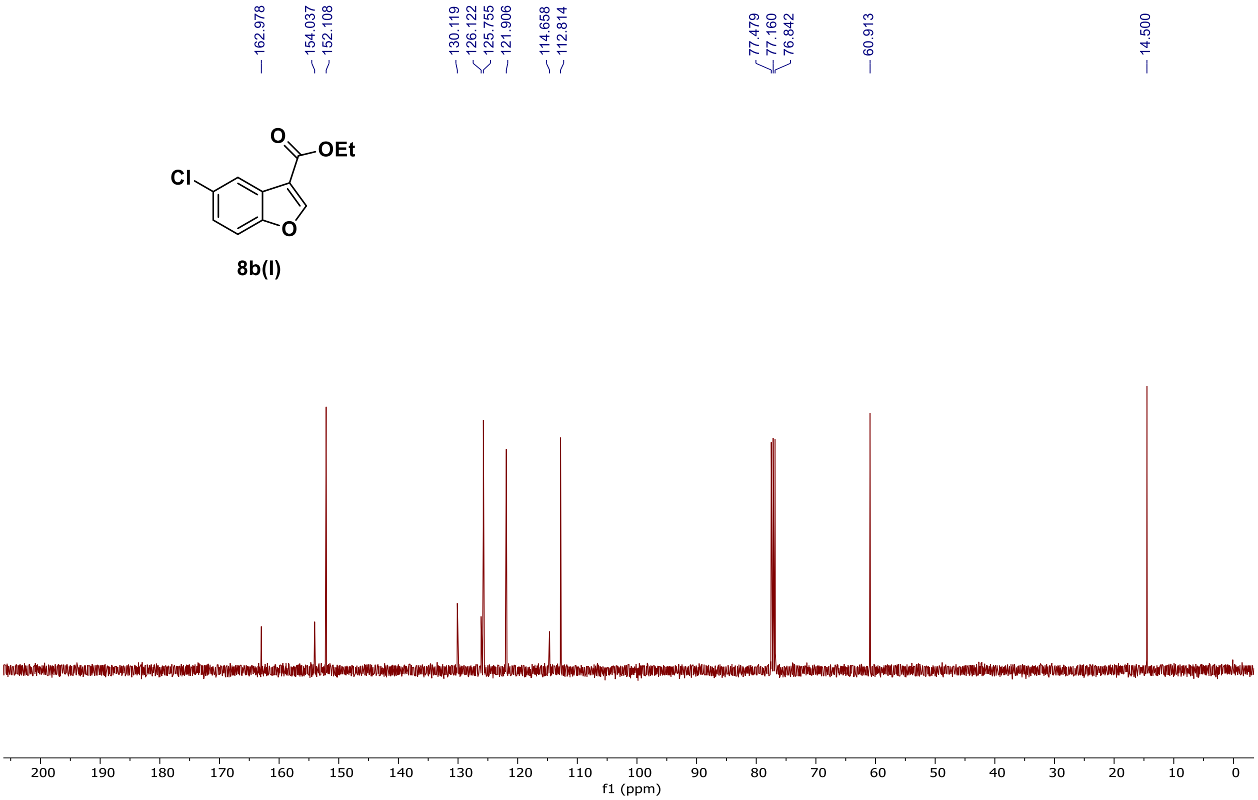

^{1}H and ^{13}C NMR Spectrum of **8b(I)** in $CDCl_3$

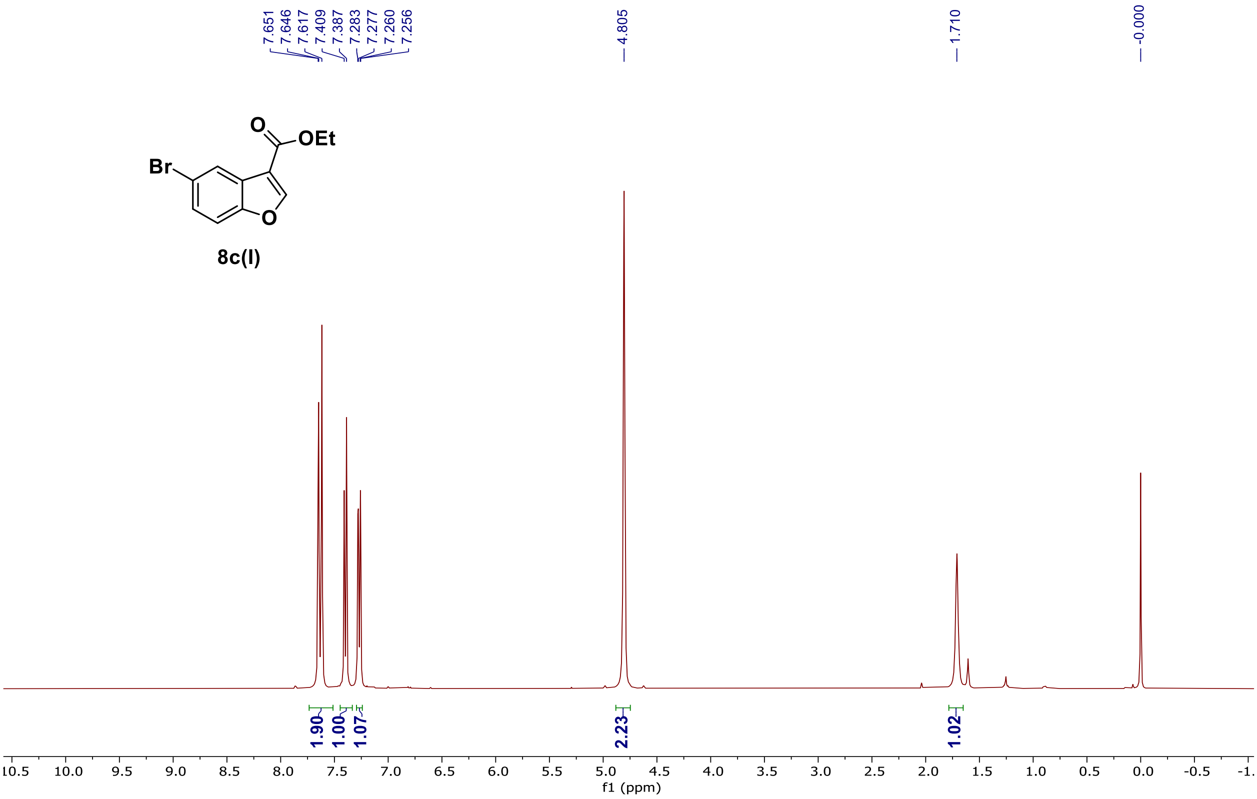

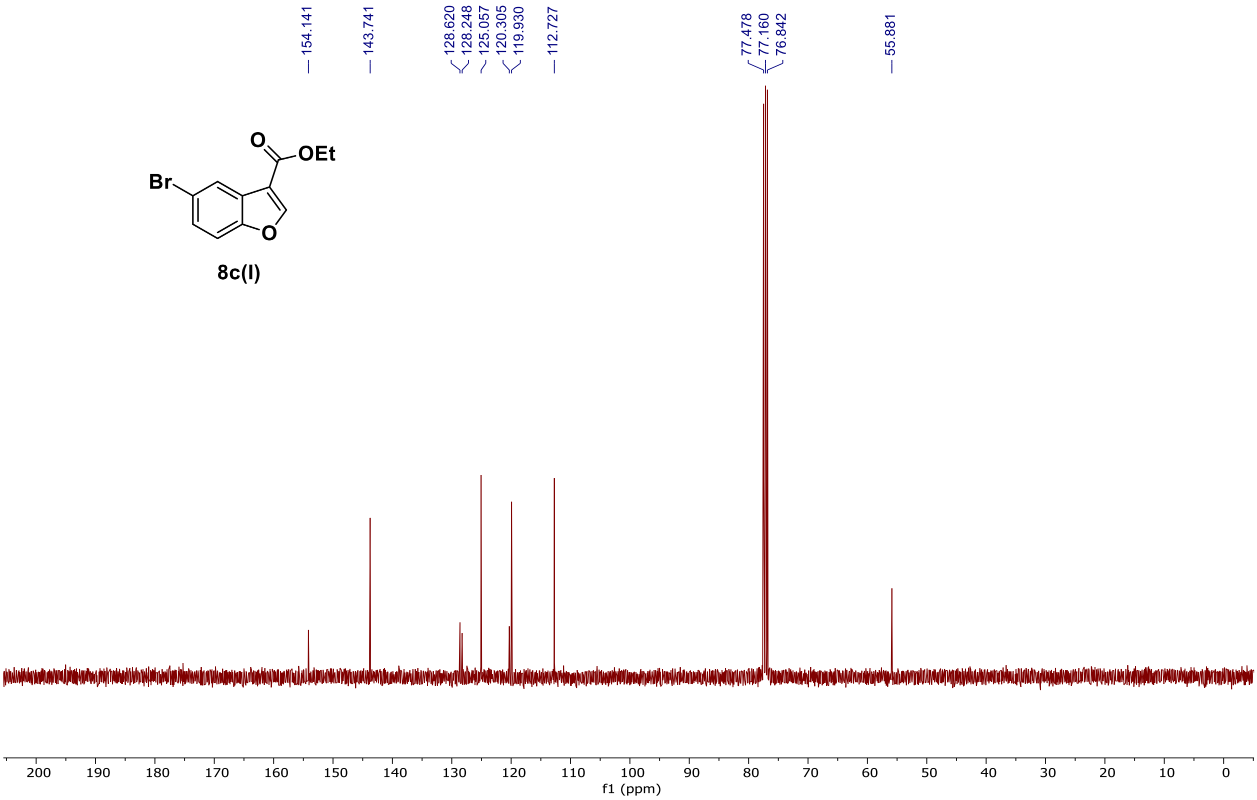

^{1}H and ^{13}C NMR Spectrum of **8b(II)** in $CDCl_3$

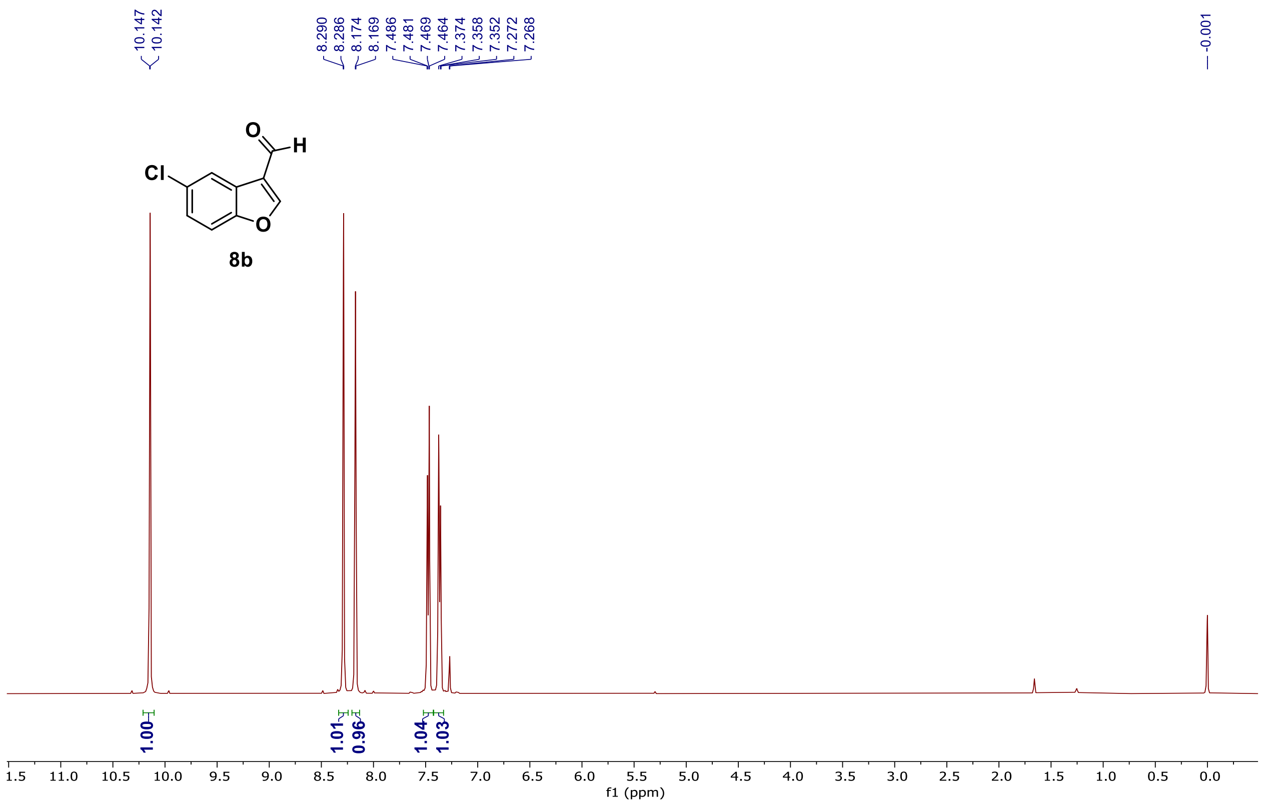

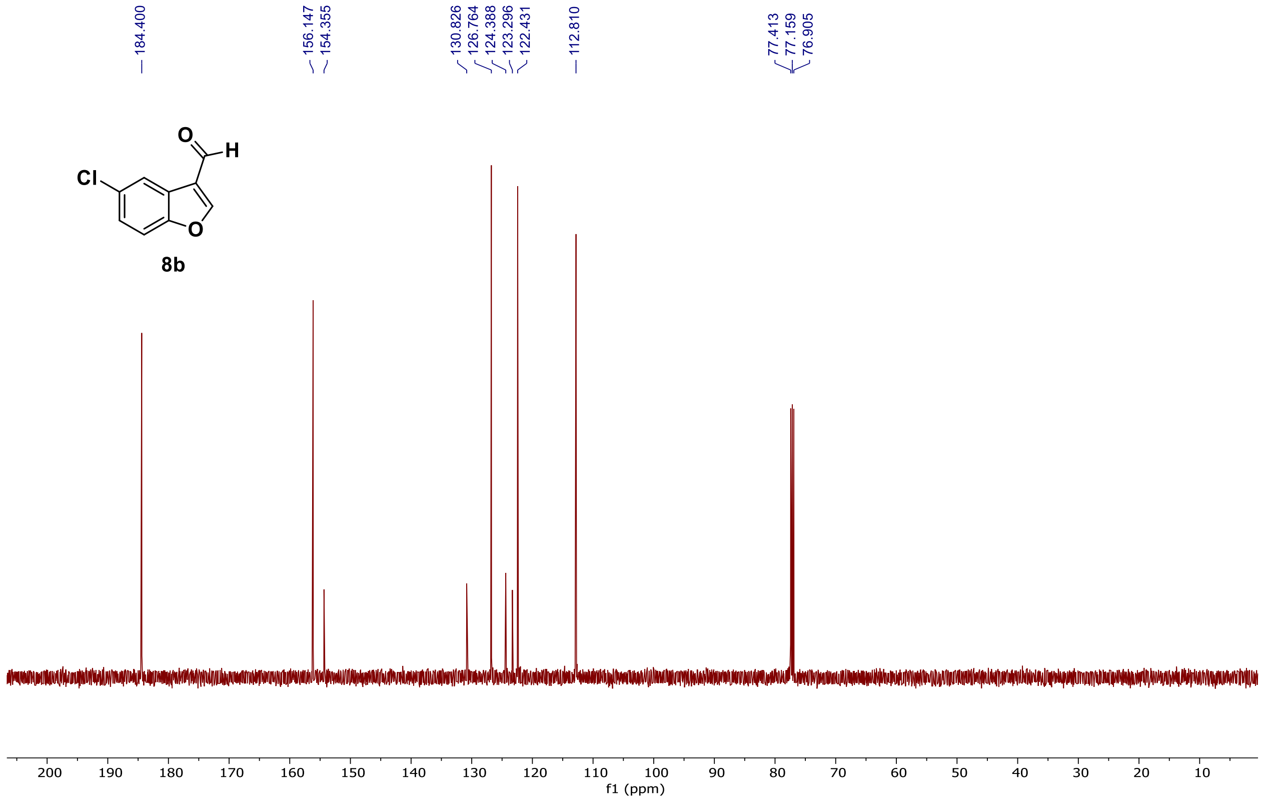

^{1}H and ^{13}C NMR Spectrum of **8b** in $CDCl_3$

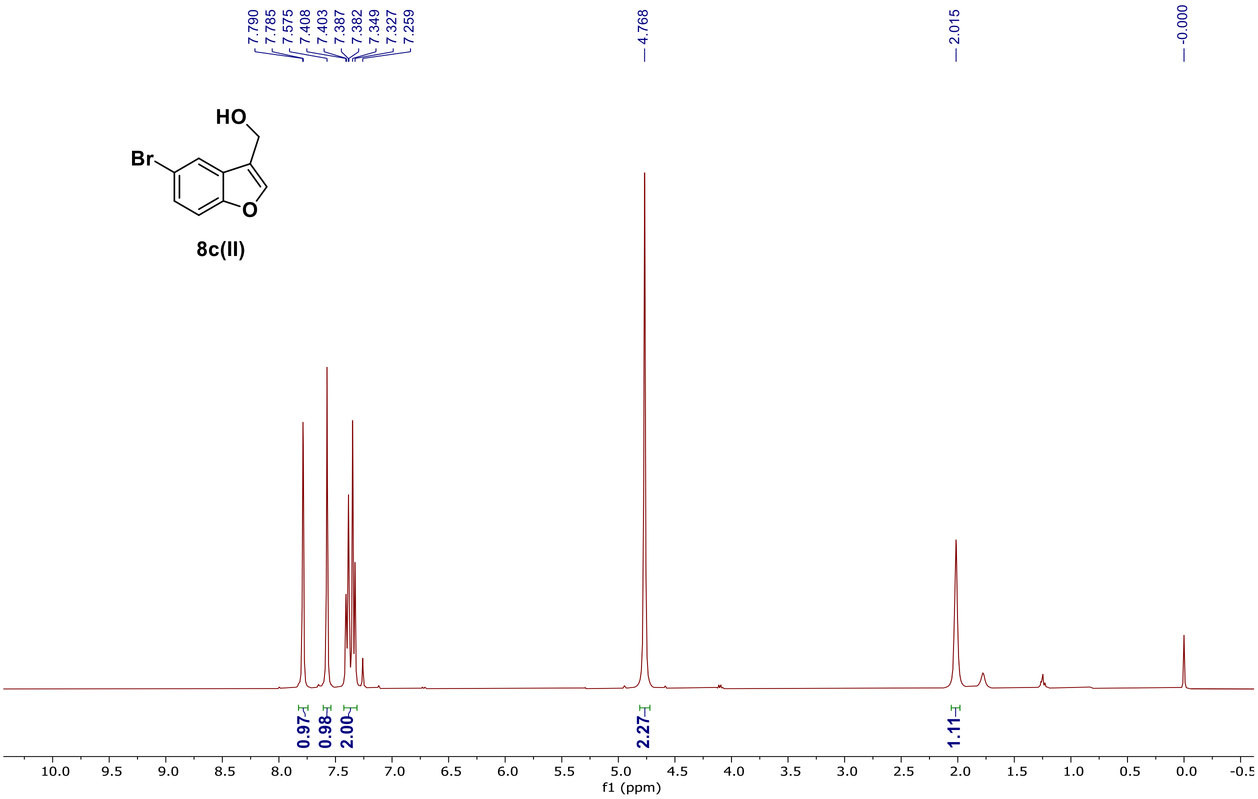

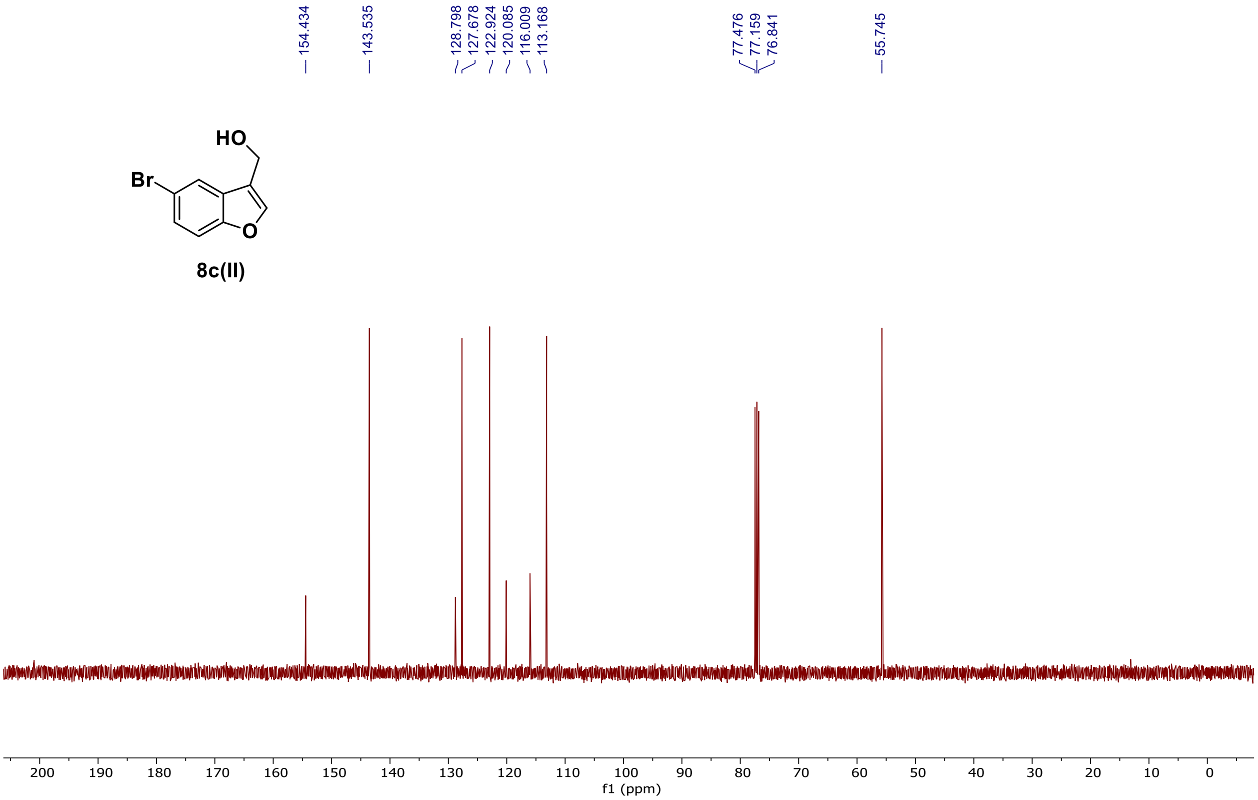

^{1}H and ^{13}C NMR Spectrum of **8c(II)** in $CDCl_3$

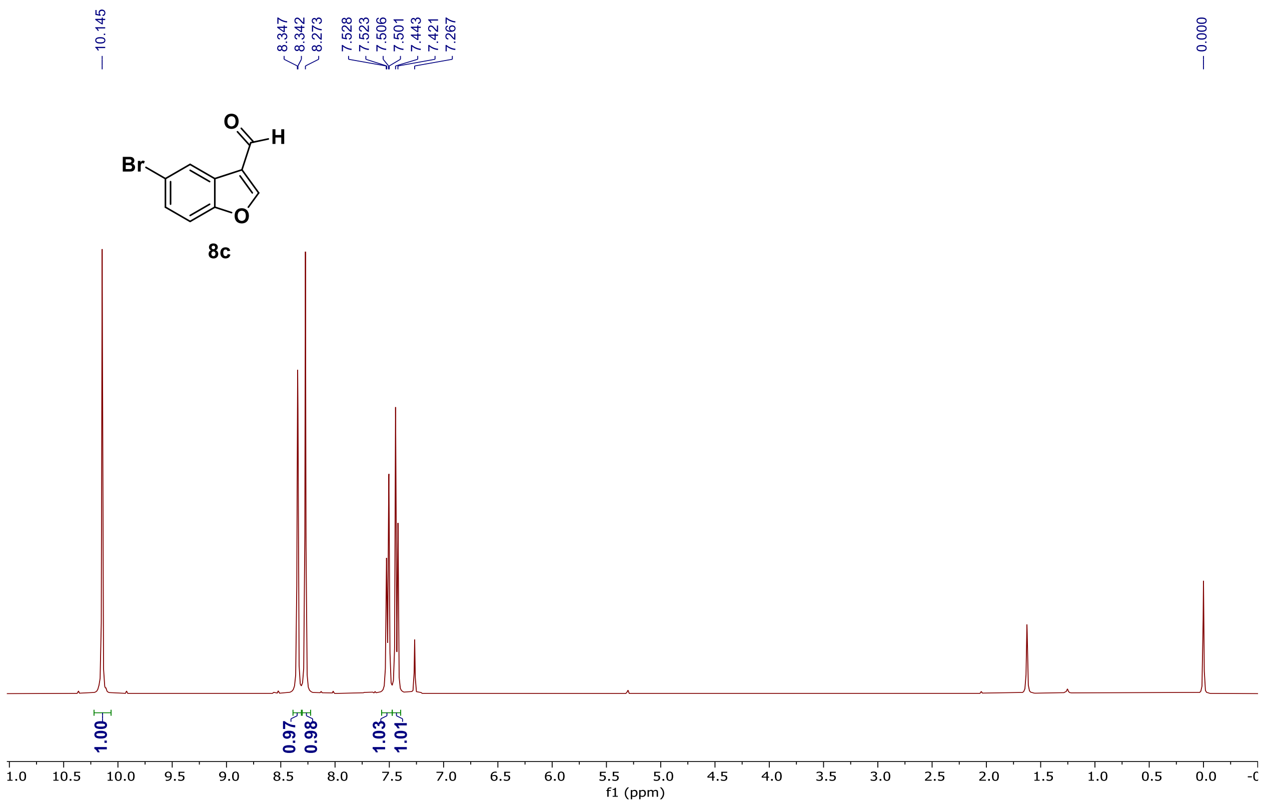

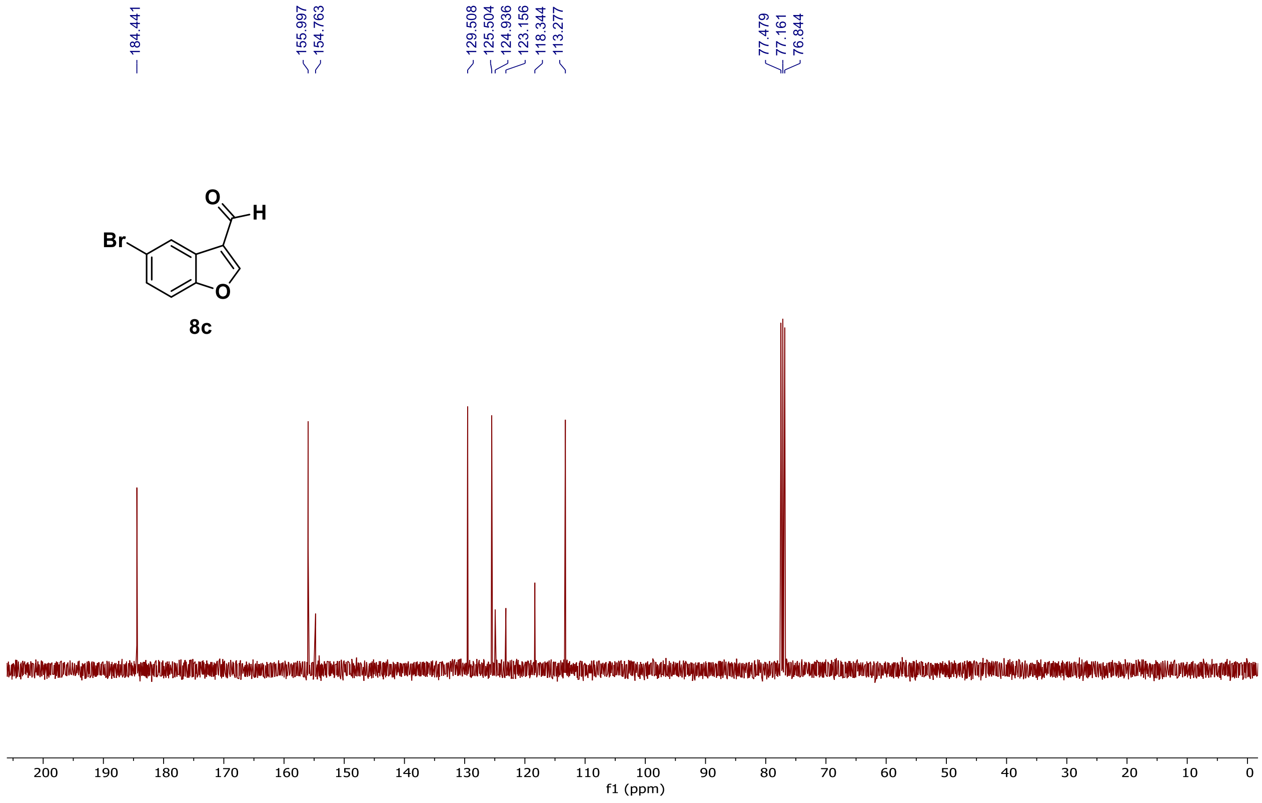

^{1}H and ^{13}C NMR Spectrum of **8c** in $CDCl_3$

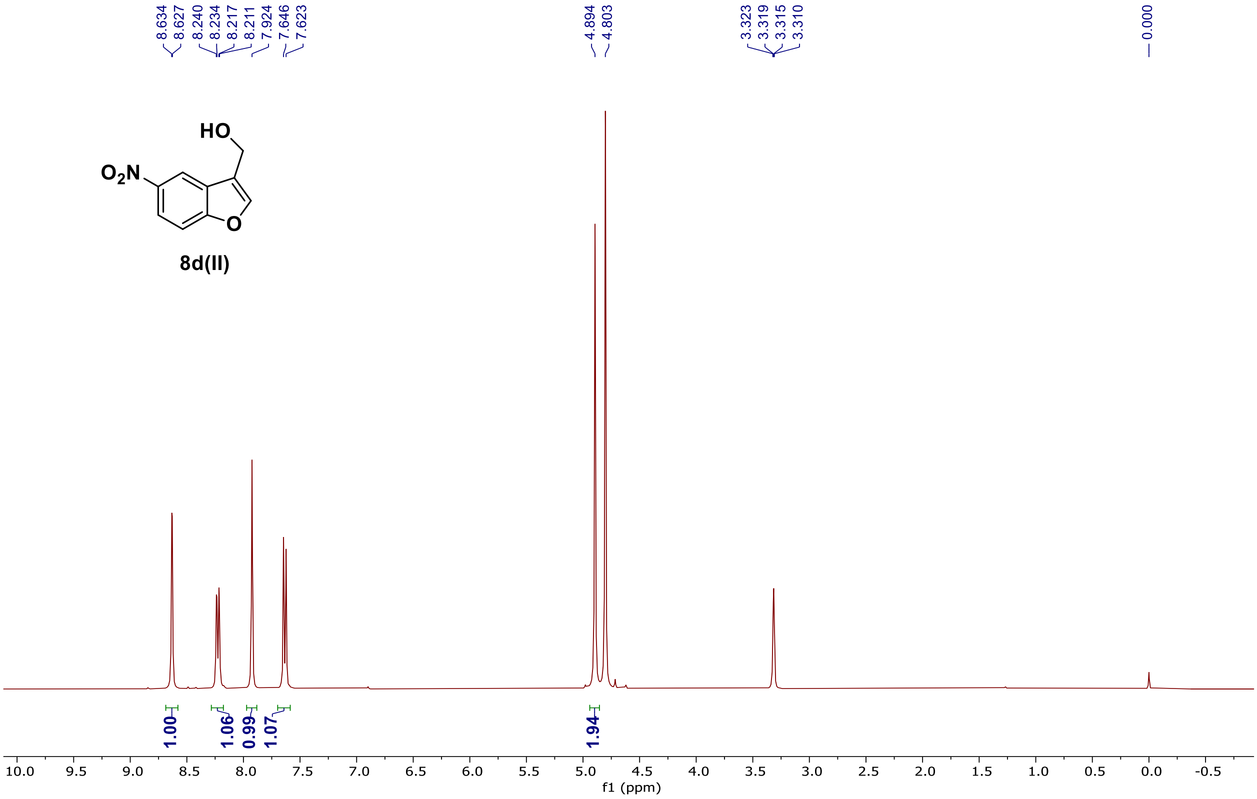

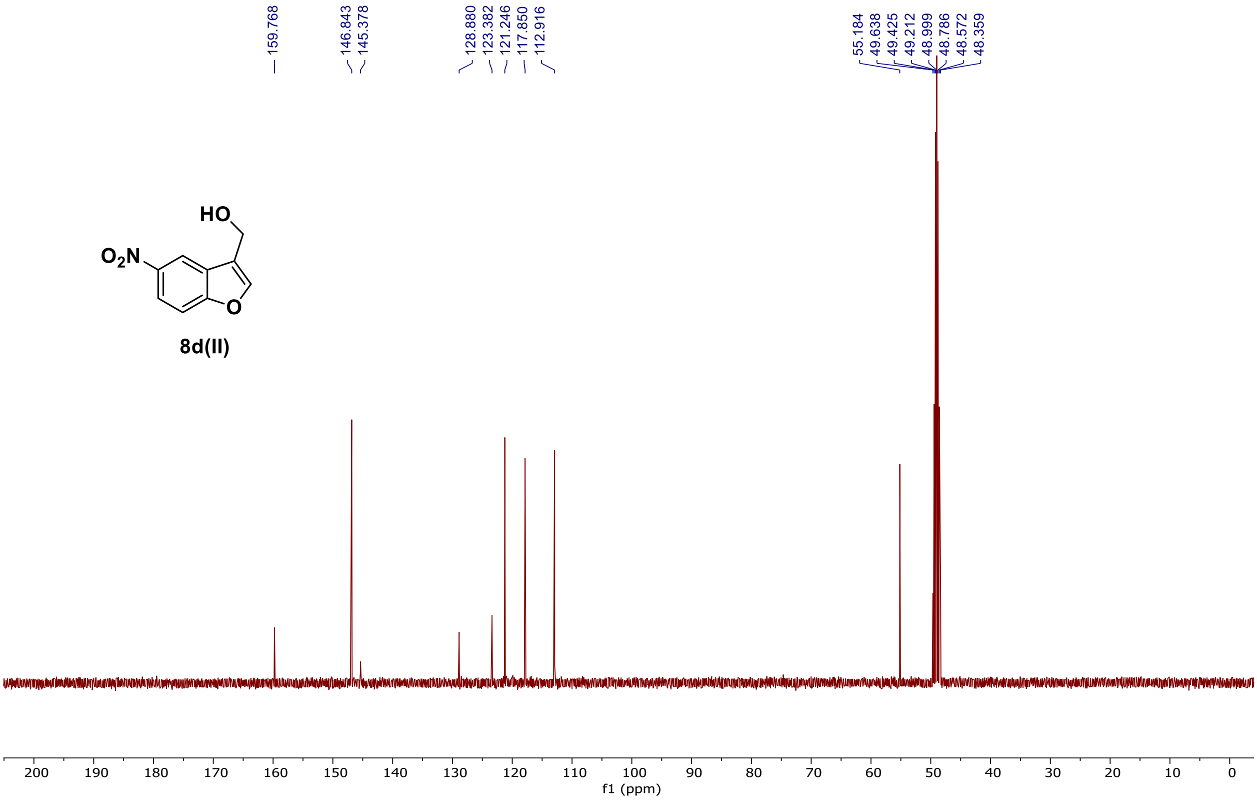

^{1}H and ^{13}C NMR Spectrum of **8d(II)** in CD_3OD

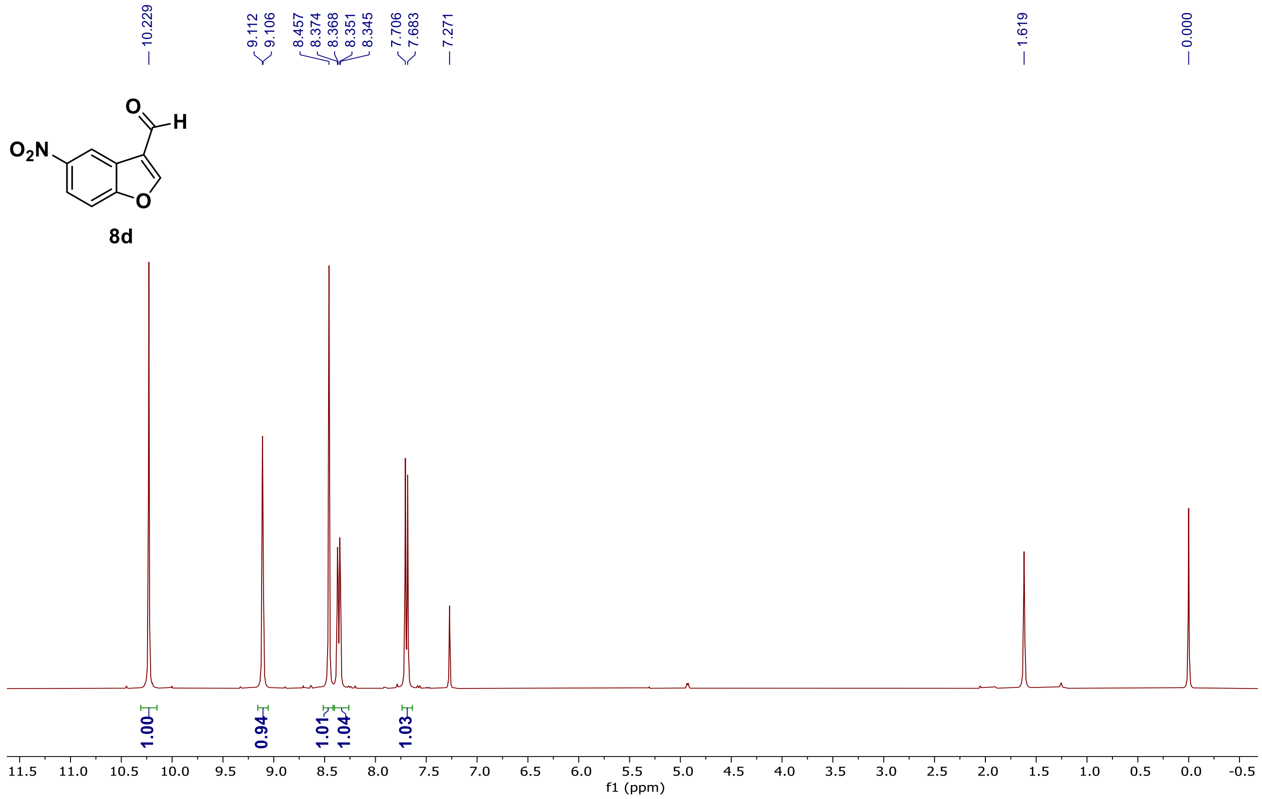

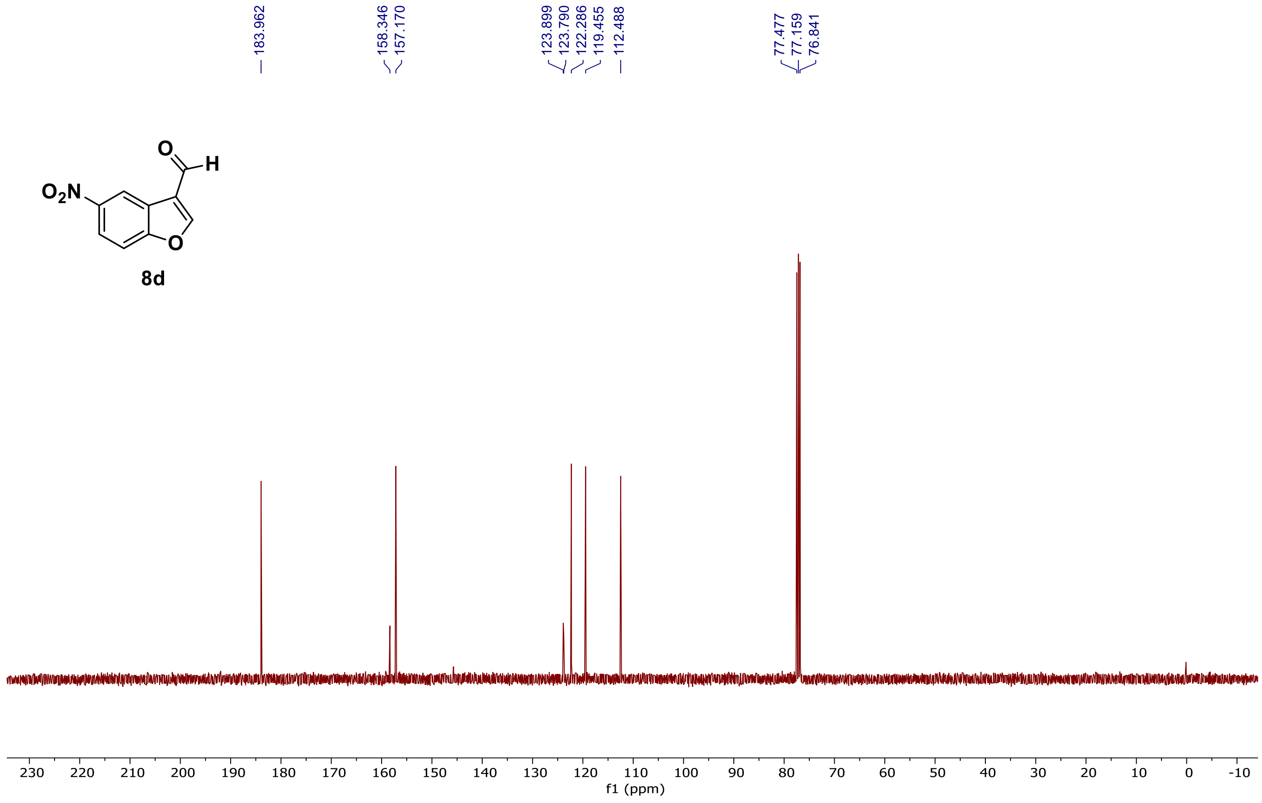

^{1}H and ^{13}C NMR Spectrum of **8d** in $CDCl_3$

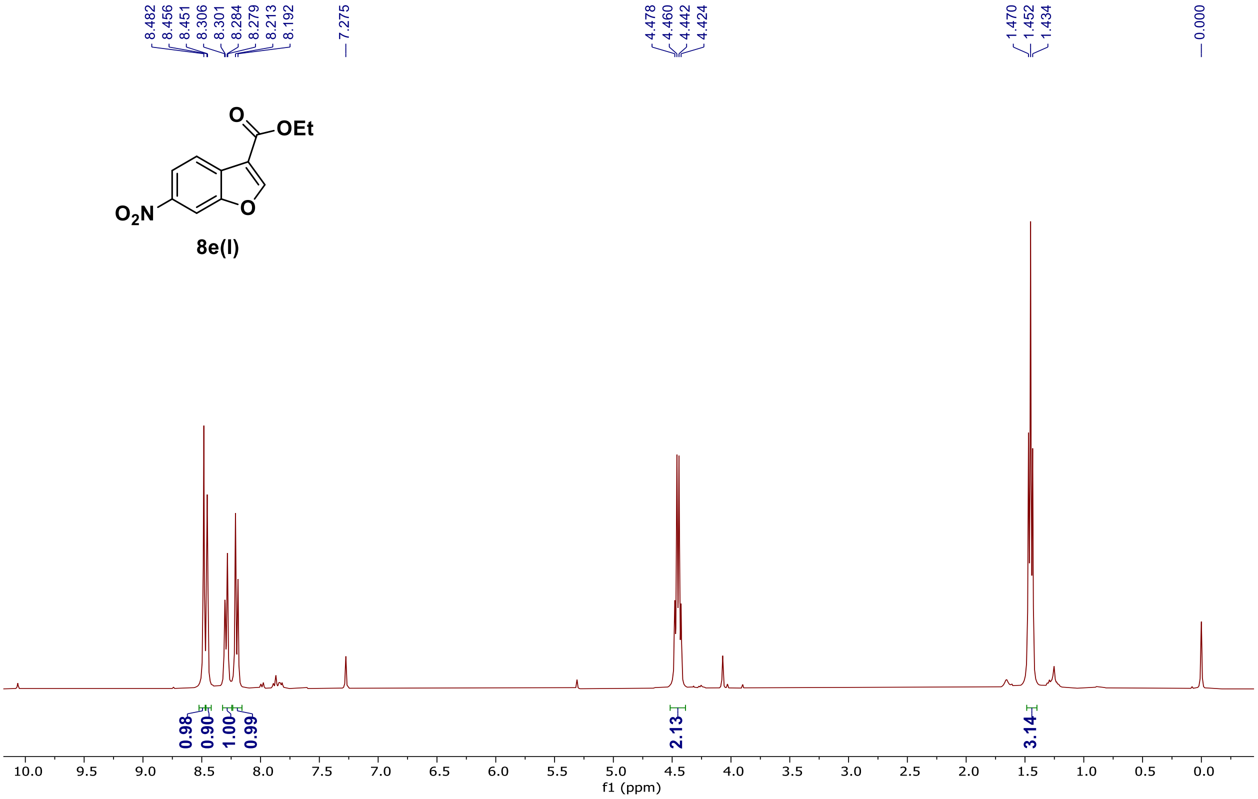

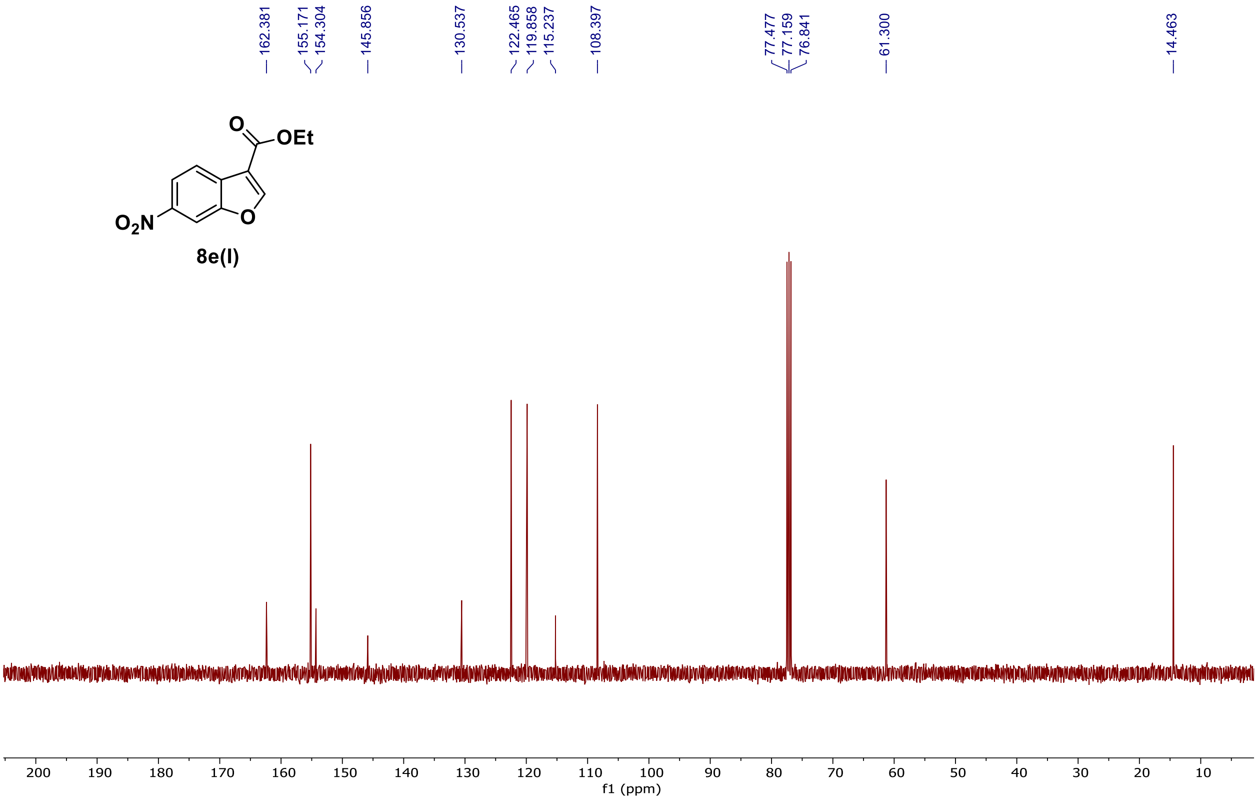

^{1}H and ^{13}C NMR Spectrum of **8e(I)** in $CDCl_3$

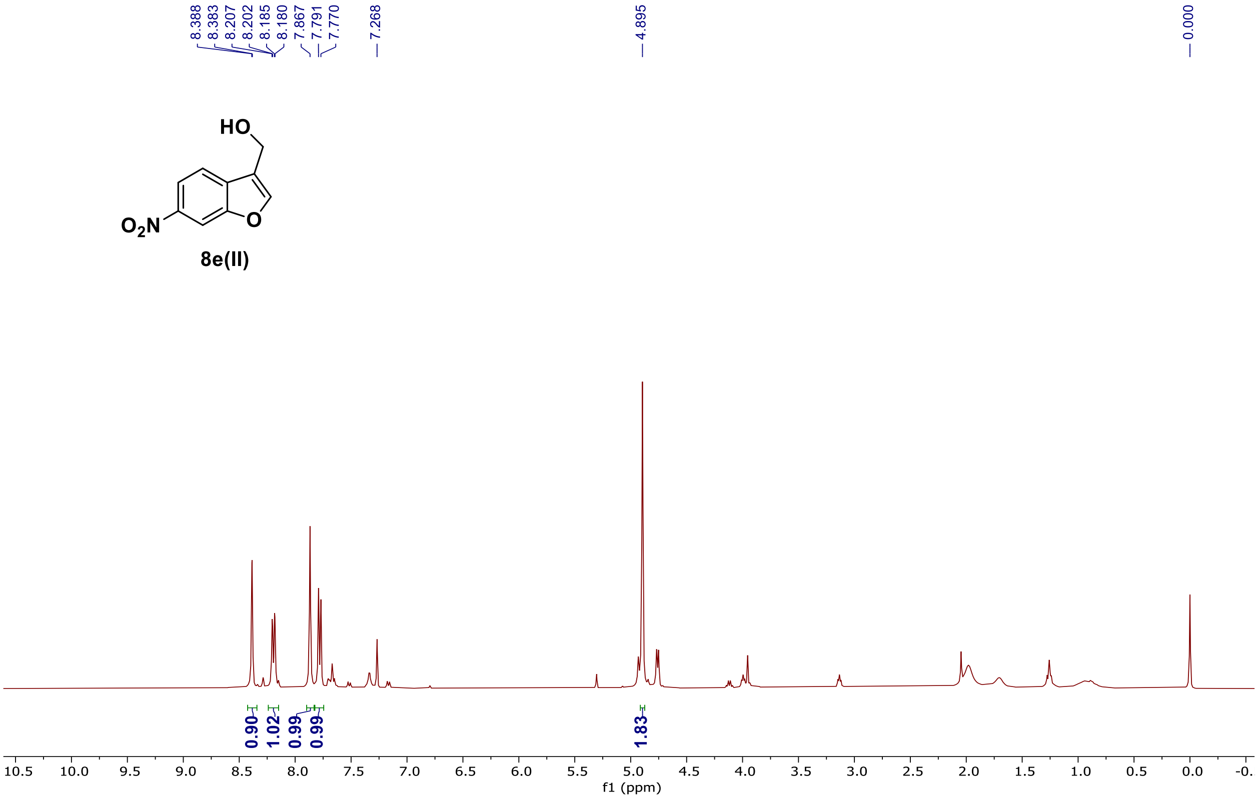

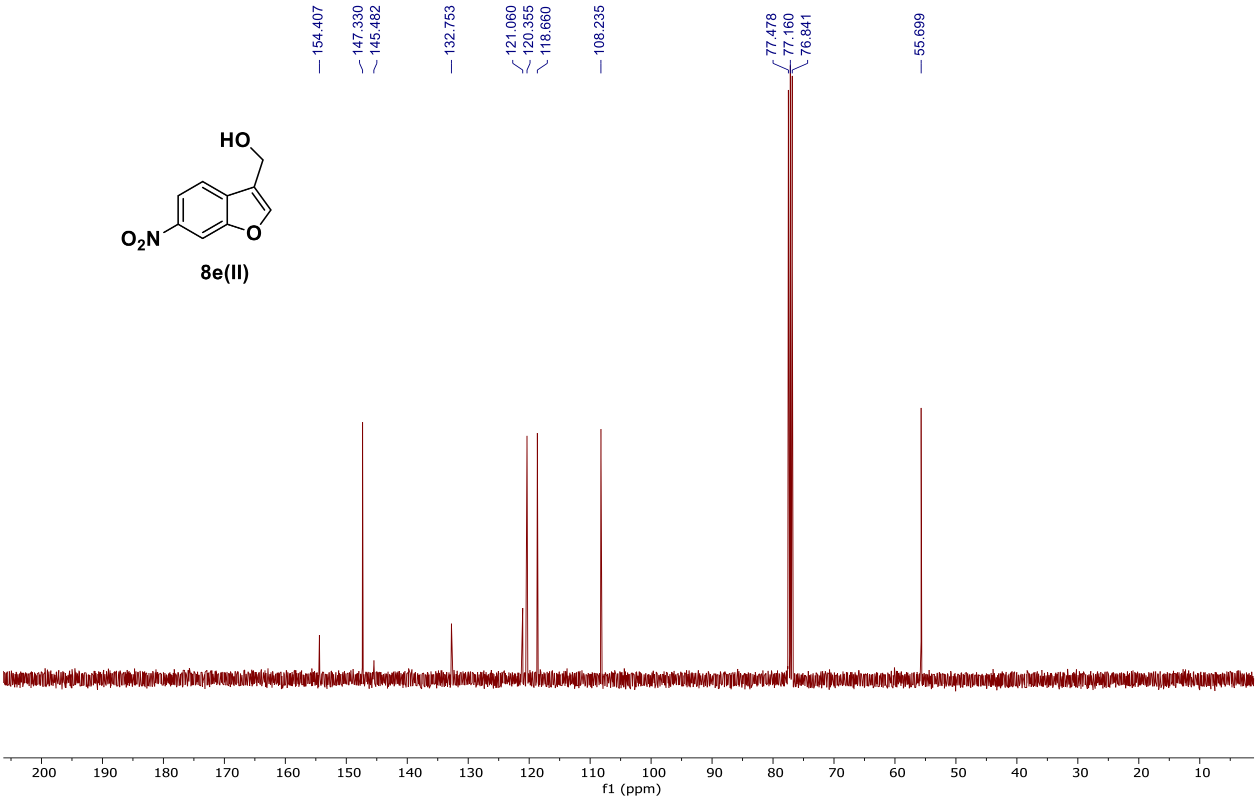

^{1}H and ^{13}C NMR Spectrum of **8e(II)** in $CDCl_3$

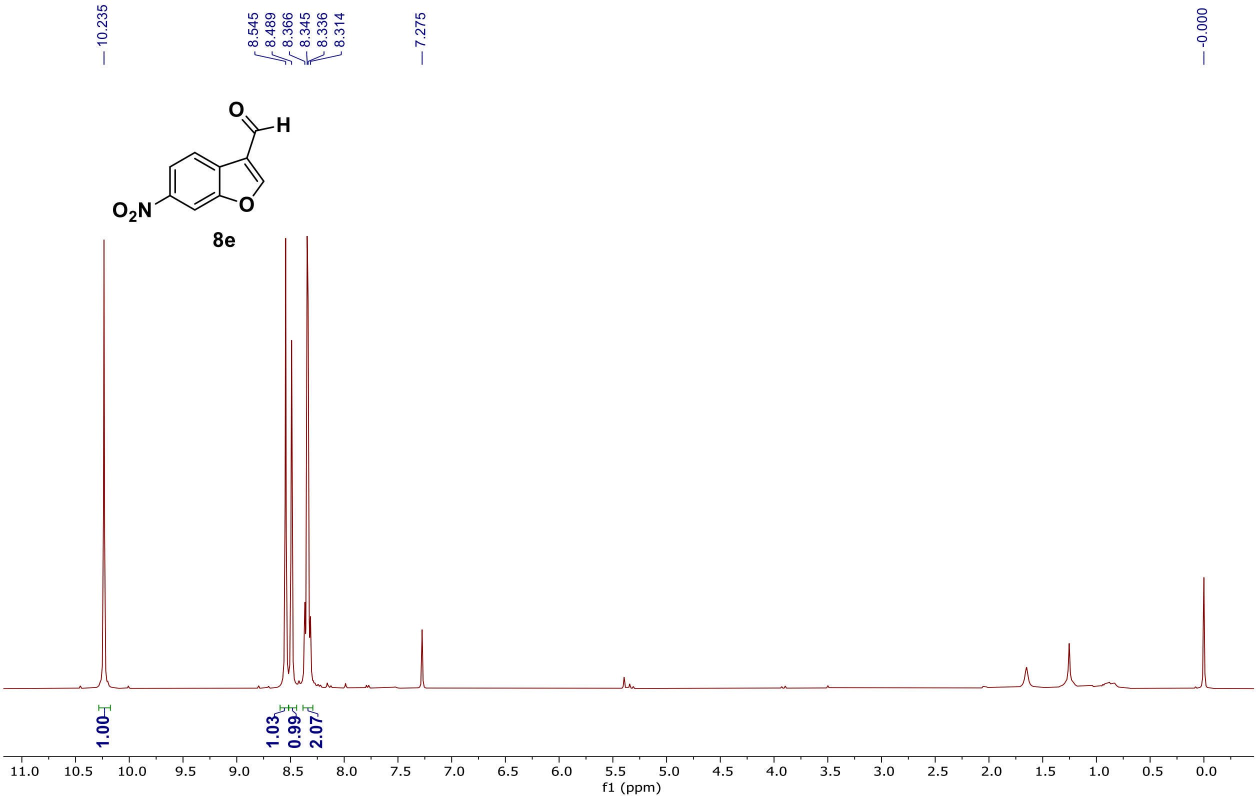

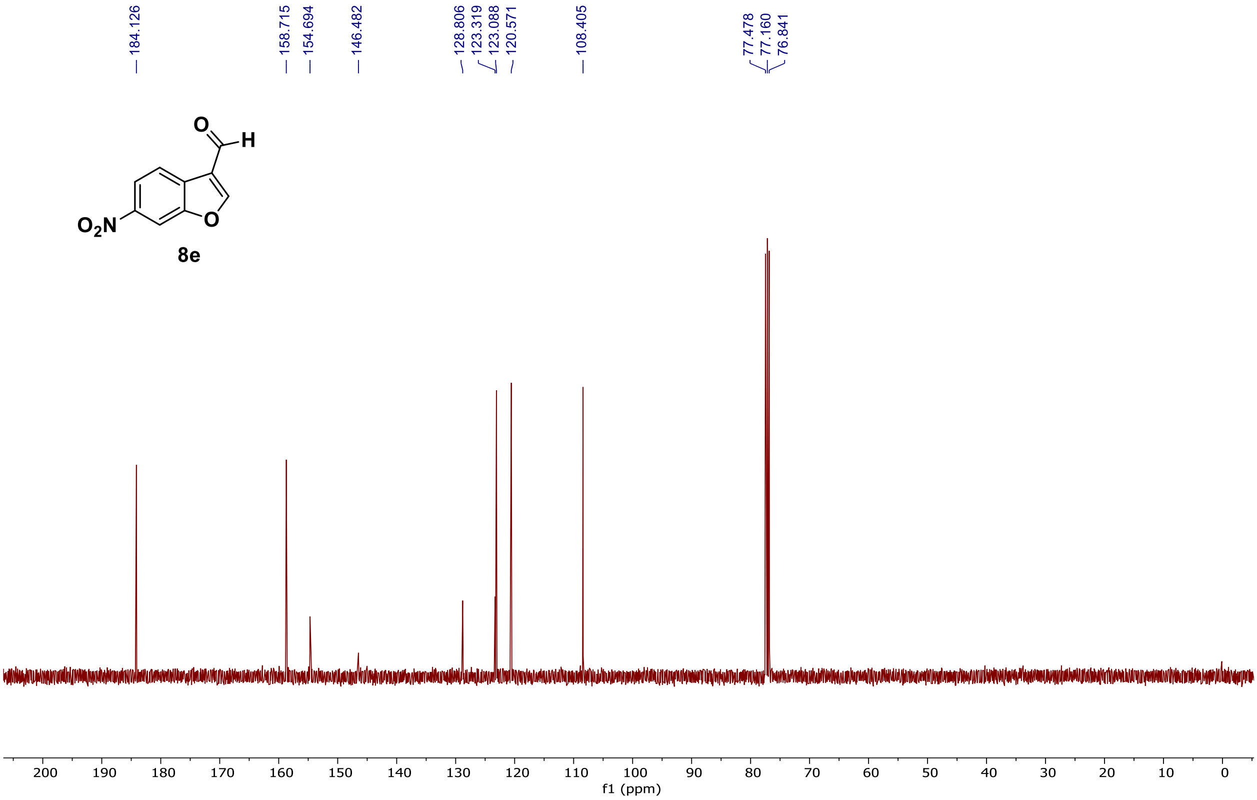

^{1}H and ^{13}C NMR Spectrum of **8e** in $CDCl_3$

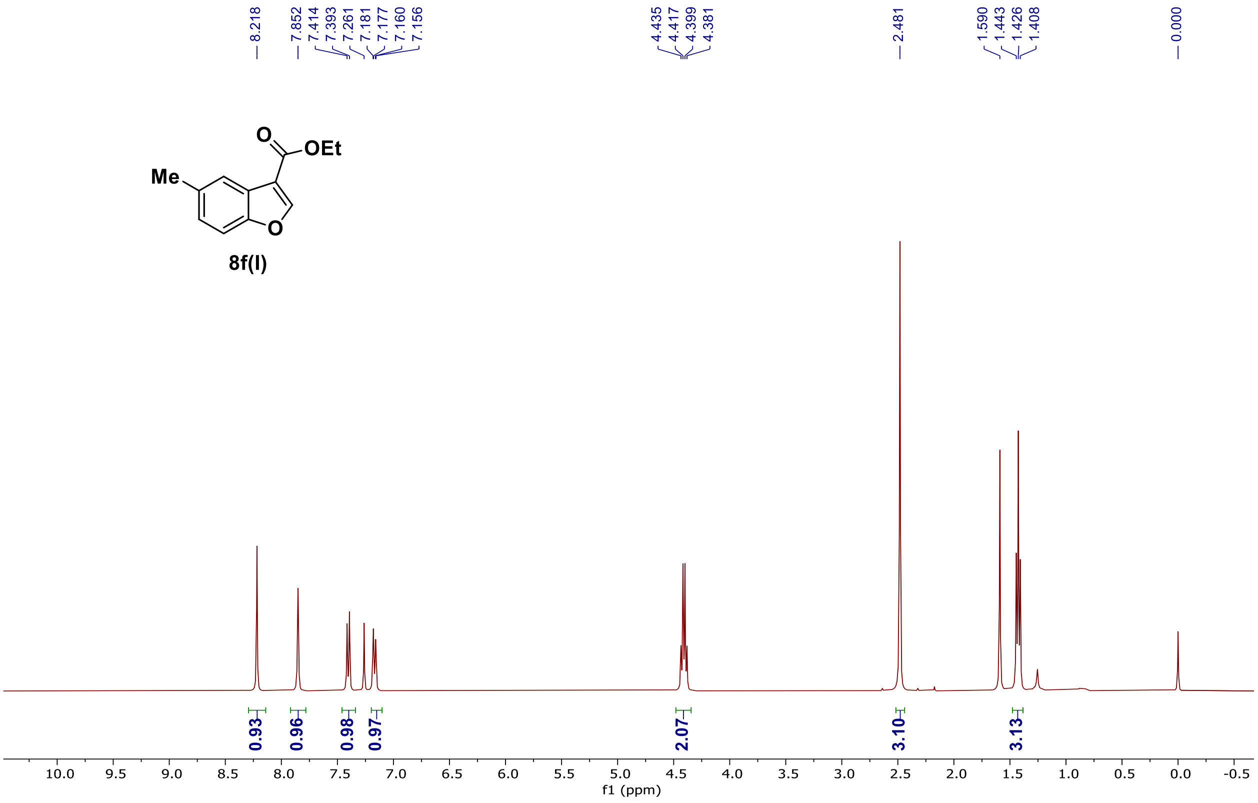

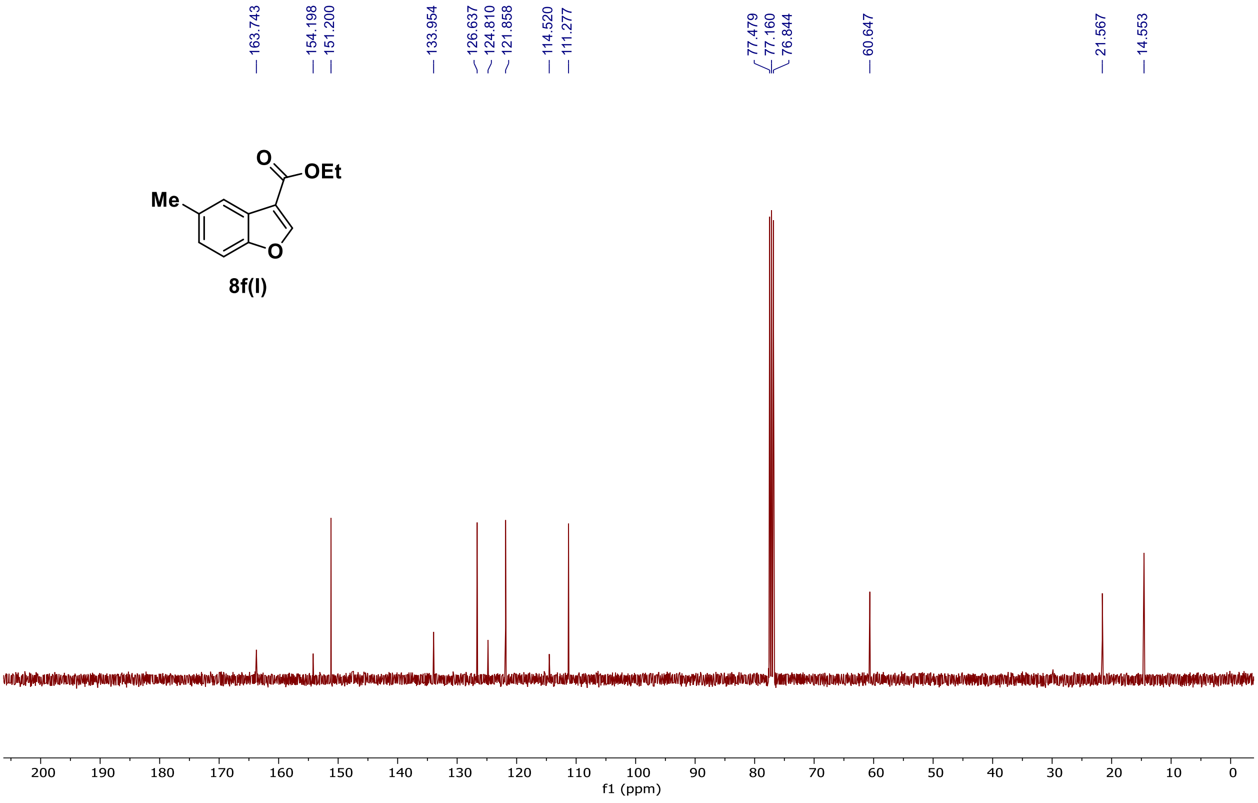

^{1}H and ^{13}C NMR Spectrum of **8f(I)** in $CDCl_3$

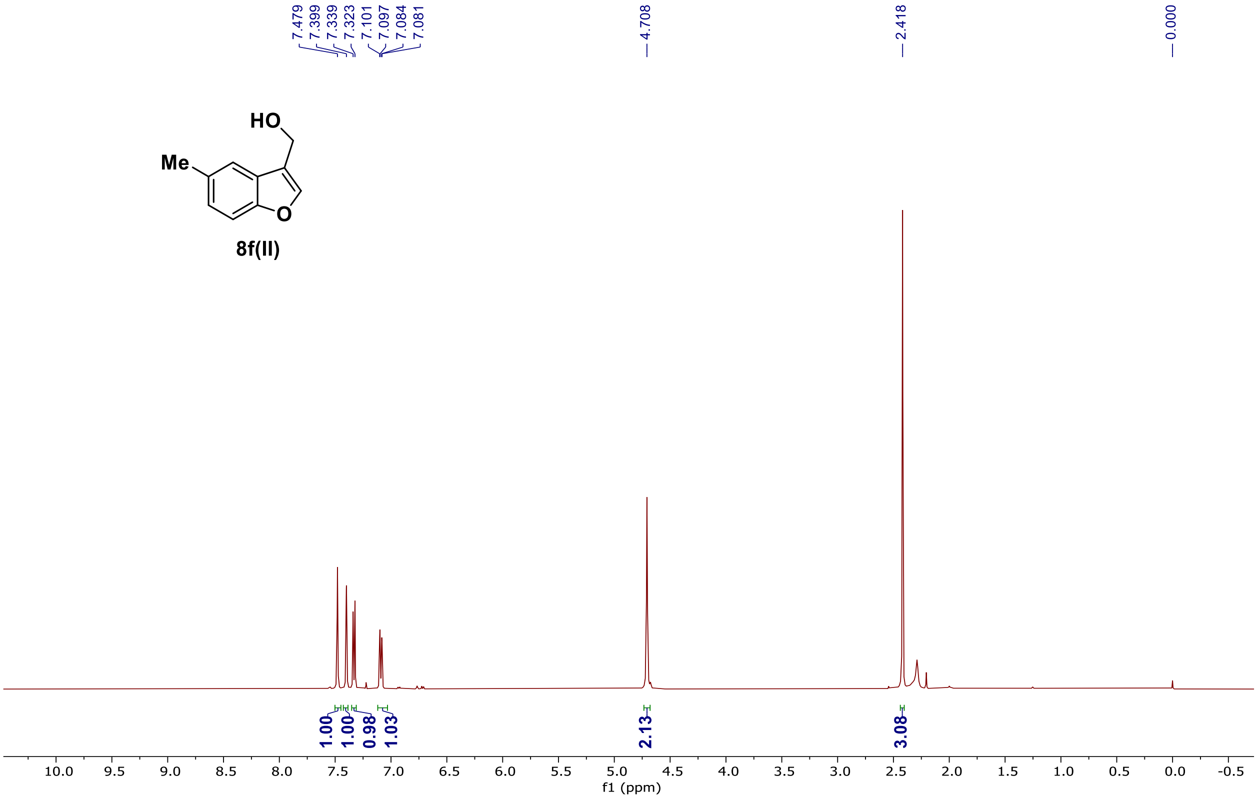

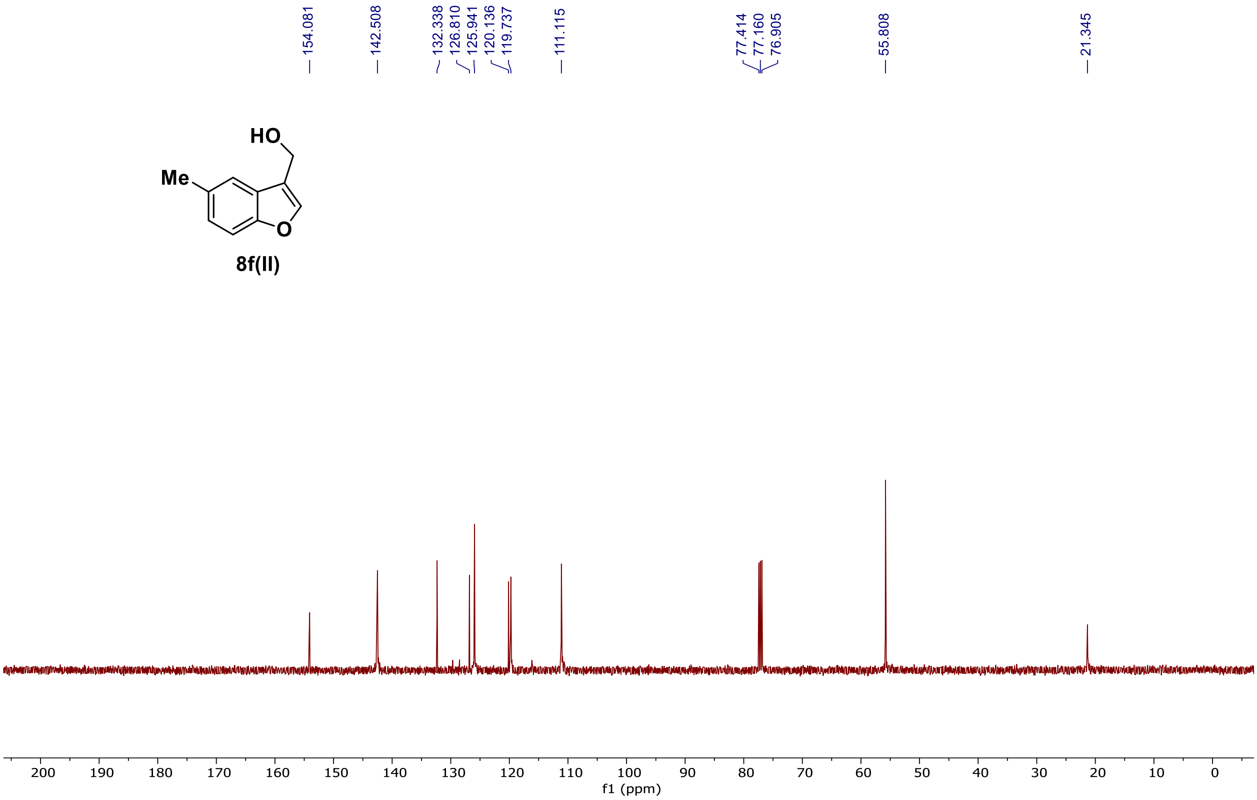

^{1}H and ^{13}C NMR Spectrum of **8f(II)** in $CDCl_3$

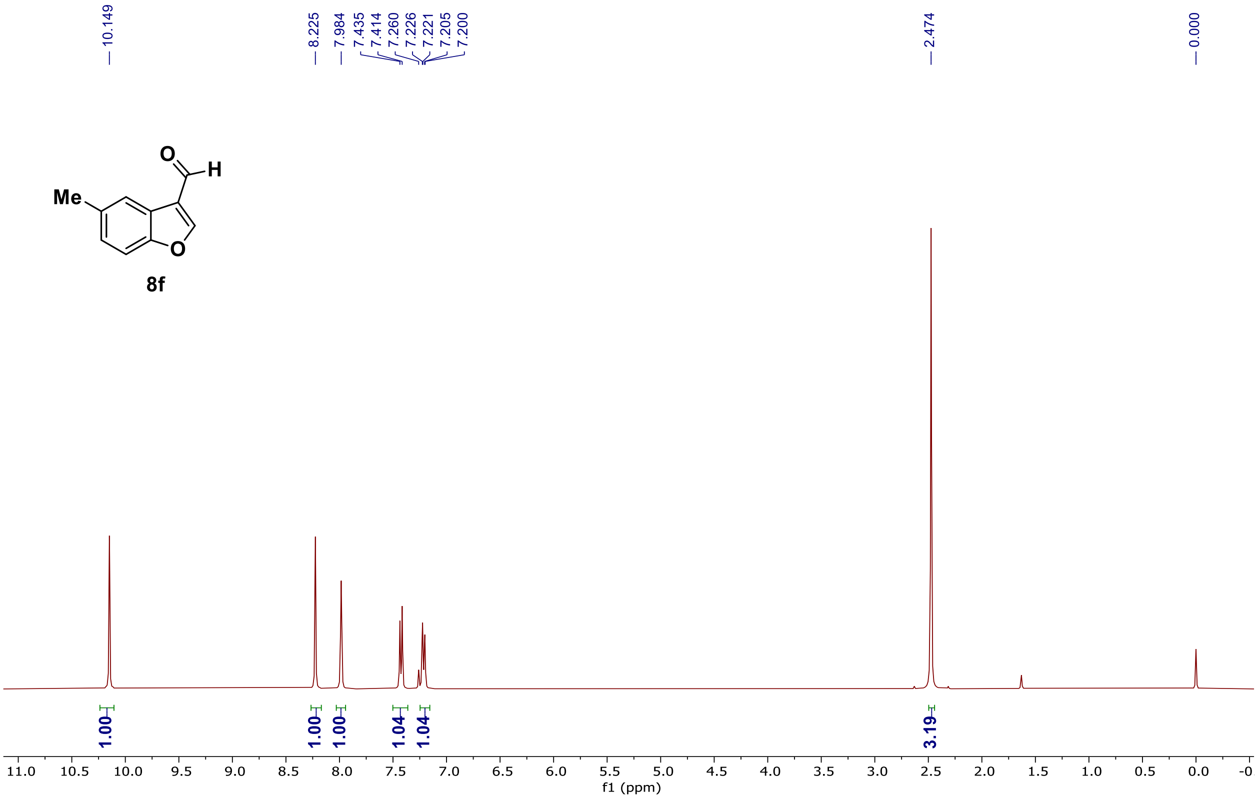

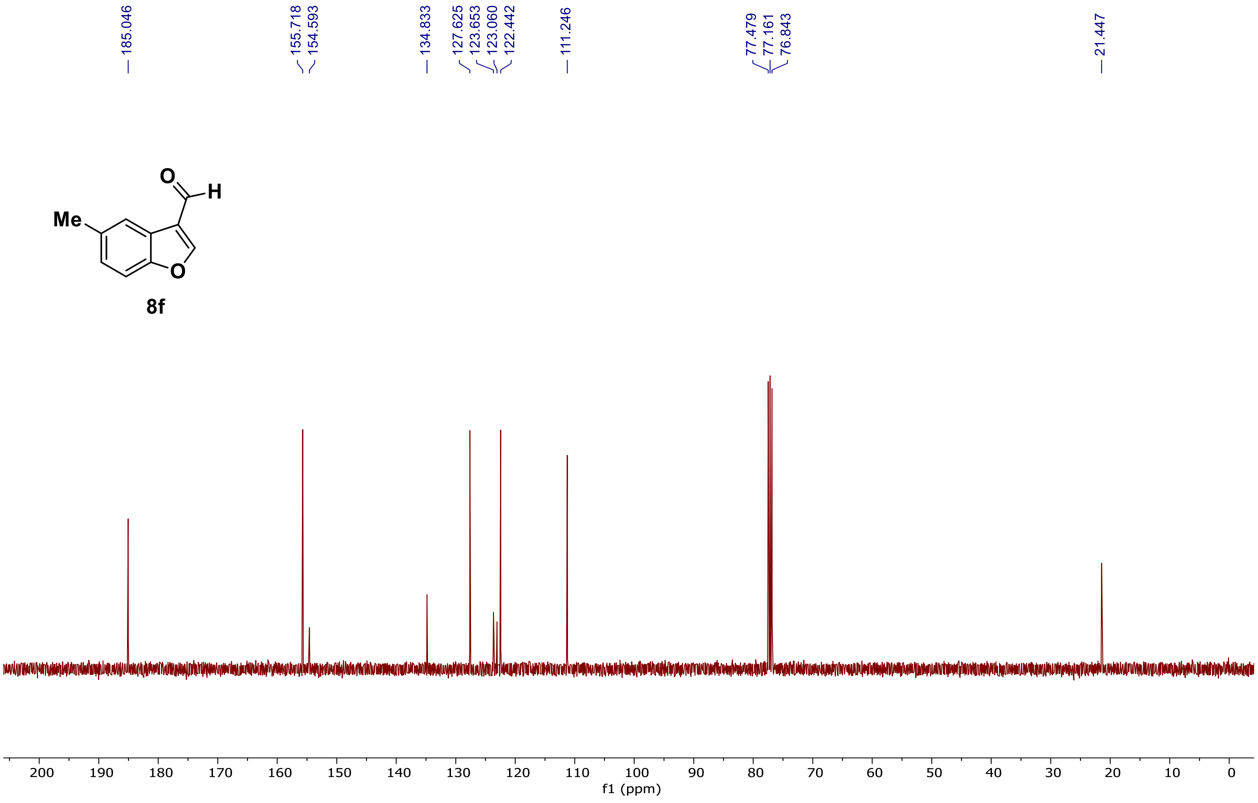

^{1}H and ^{13}C NMR Spectrum of **8f** in $CDCl_3$

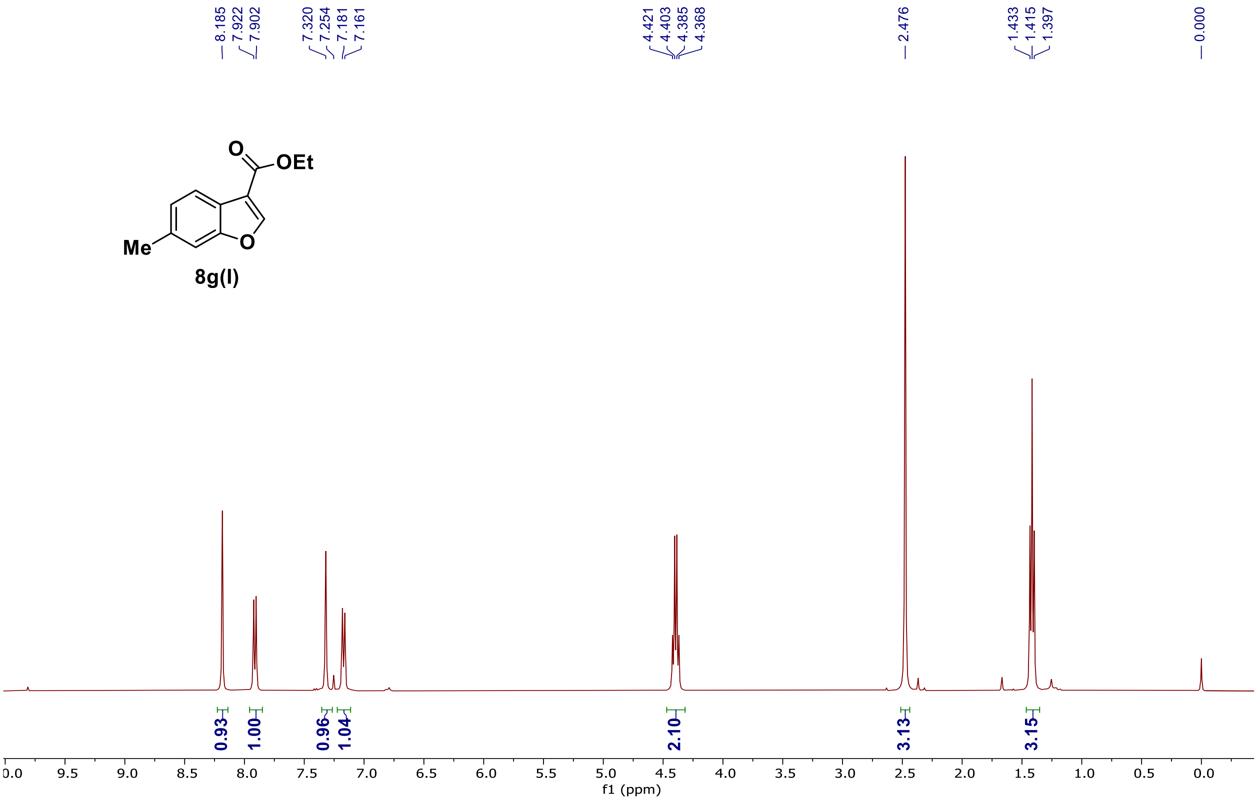

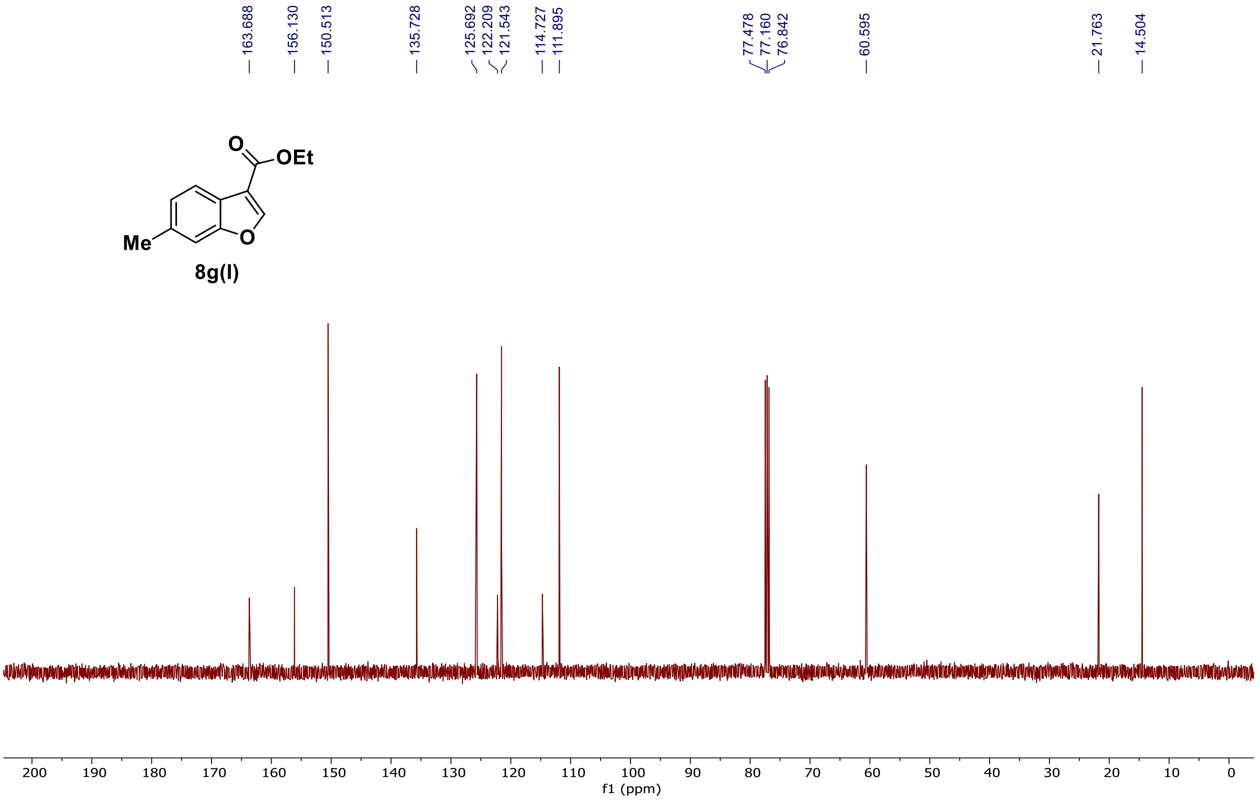

^{1}H and ^{13}C NMR Spectrum of **8g(I)** in $CDCl_3$

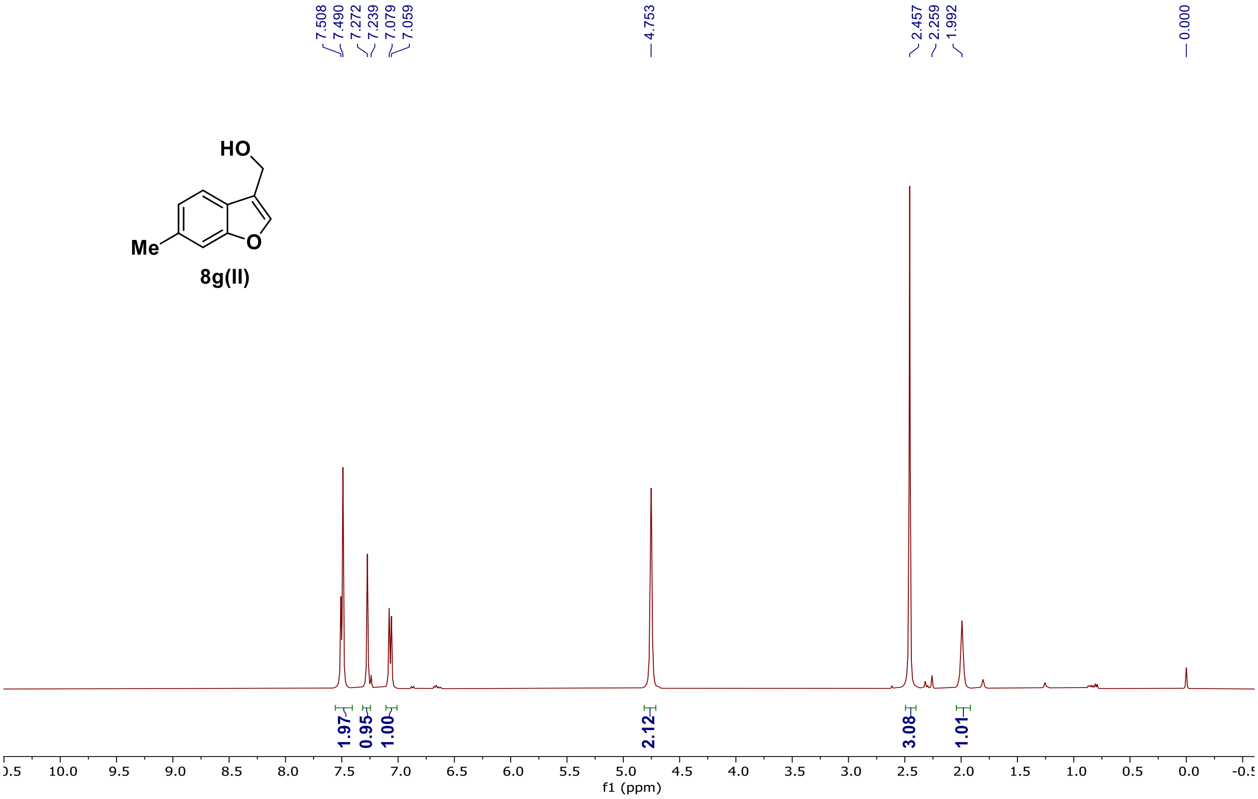

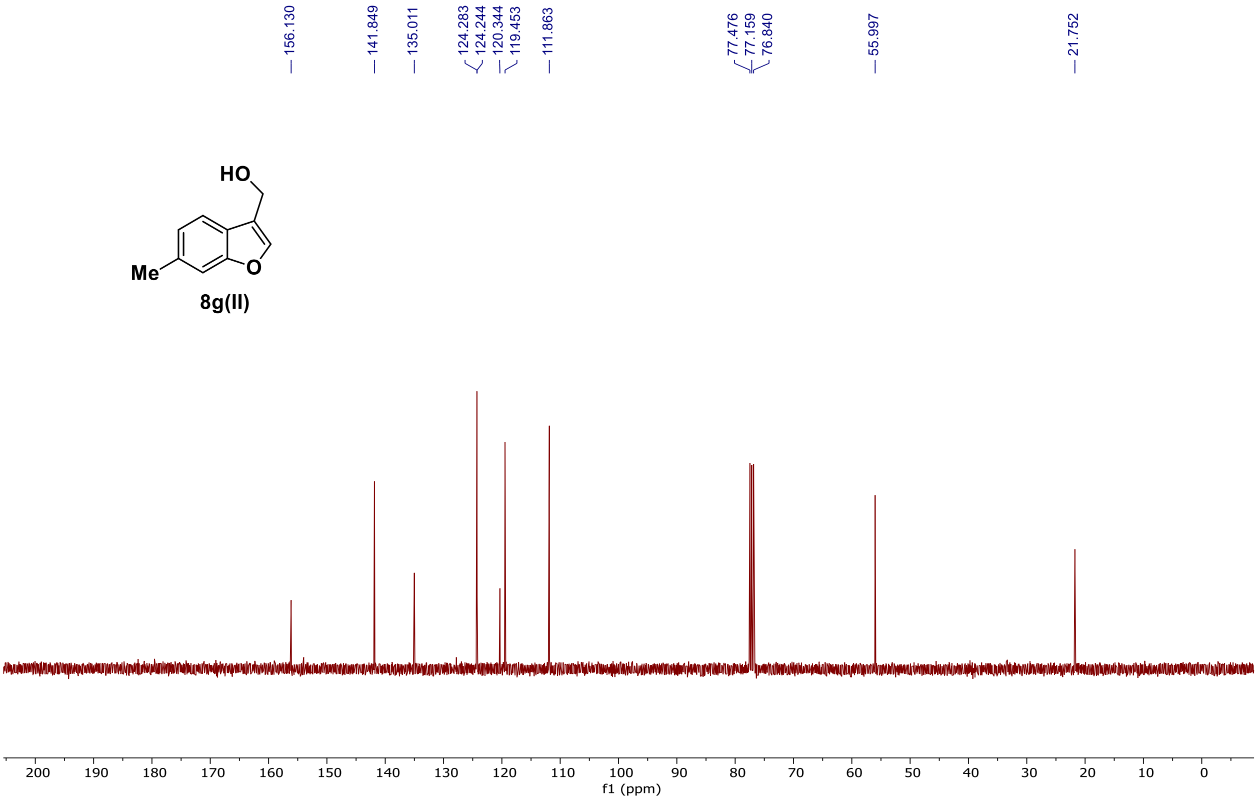

^{1}H and ^{13}C NMR Spectrum of **8g(II)** in $CDCl_3$

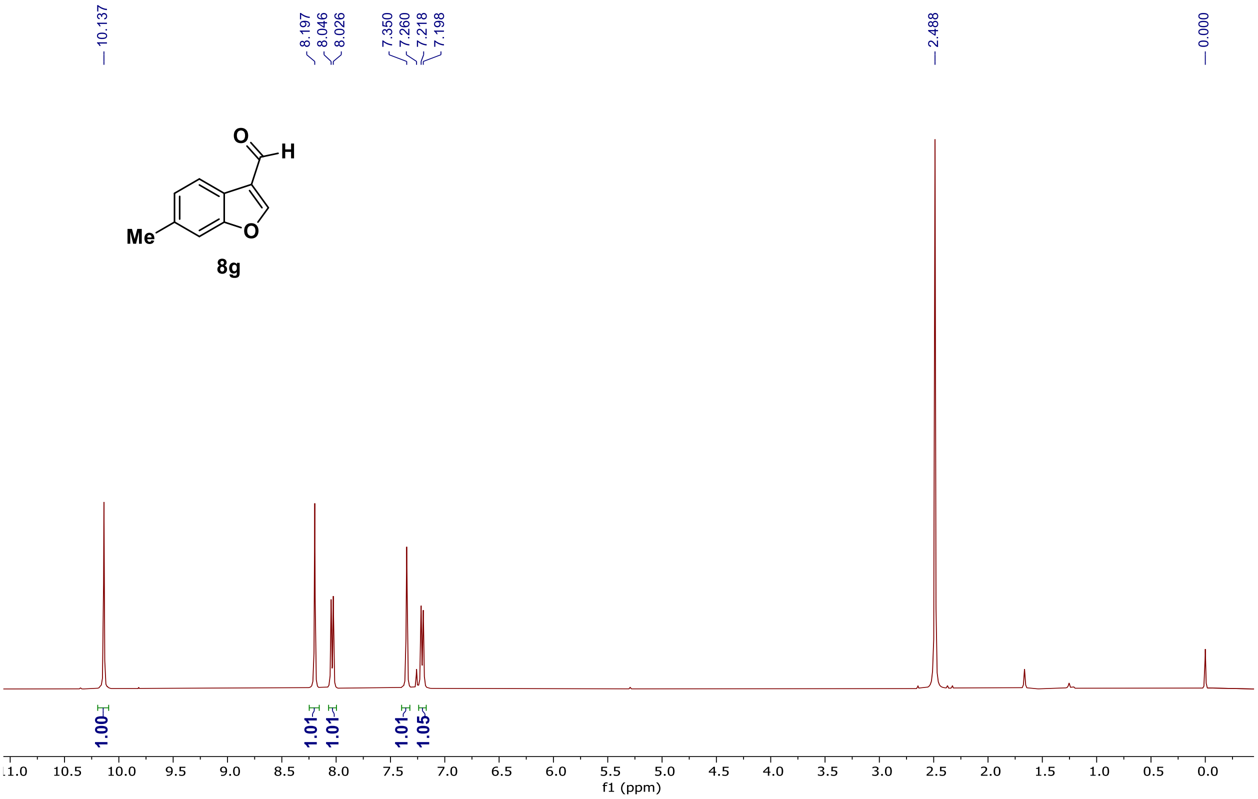

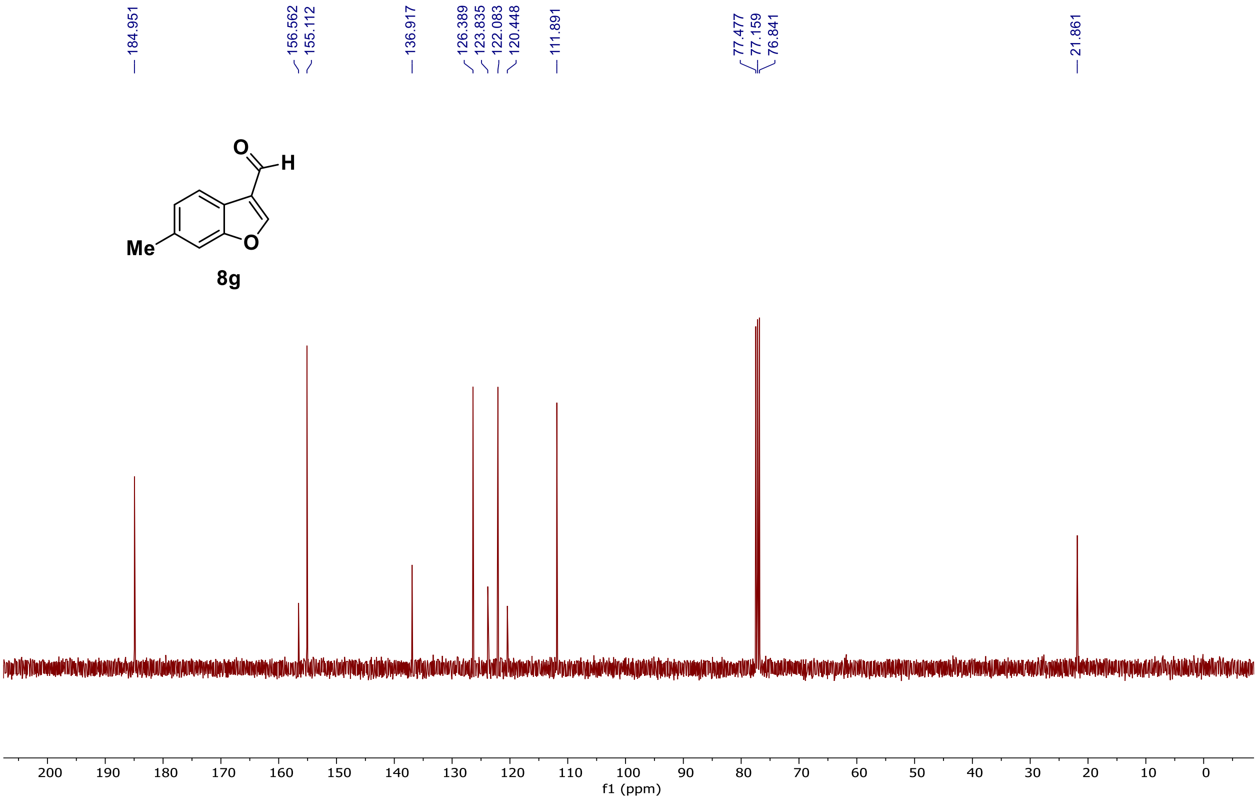

^{1}H and ^{13}C NMR Spectrum of **8g** in $CDCl_3$

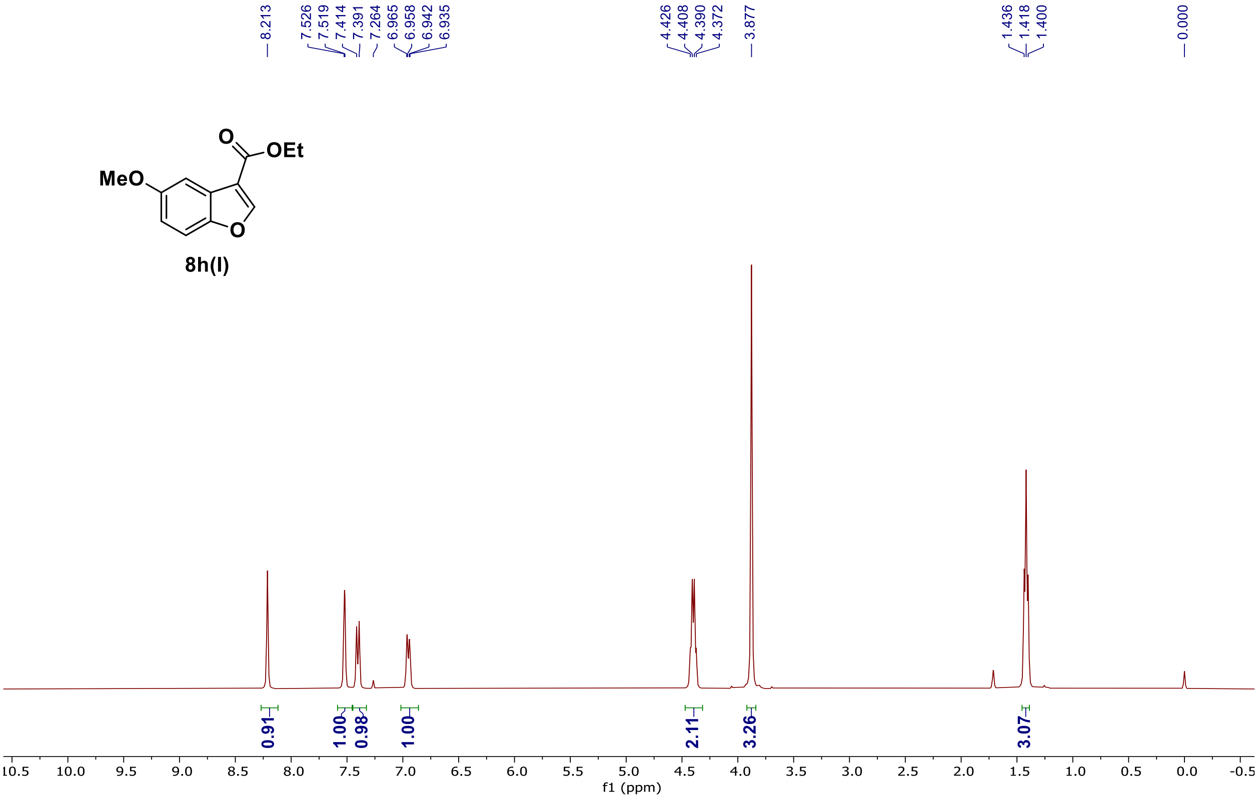

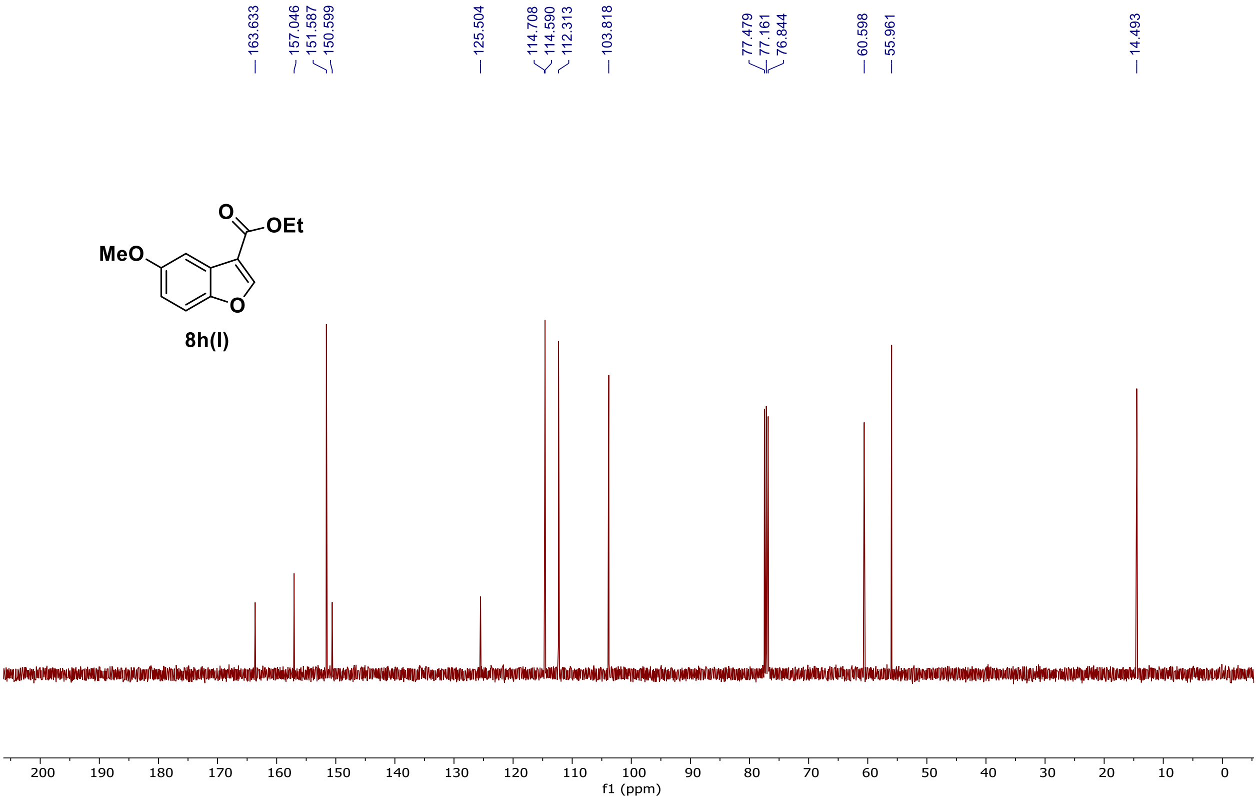

^{1}H and ^{13}C NMR Spectrum of **8h(I)** in $CDCl_3$

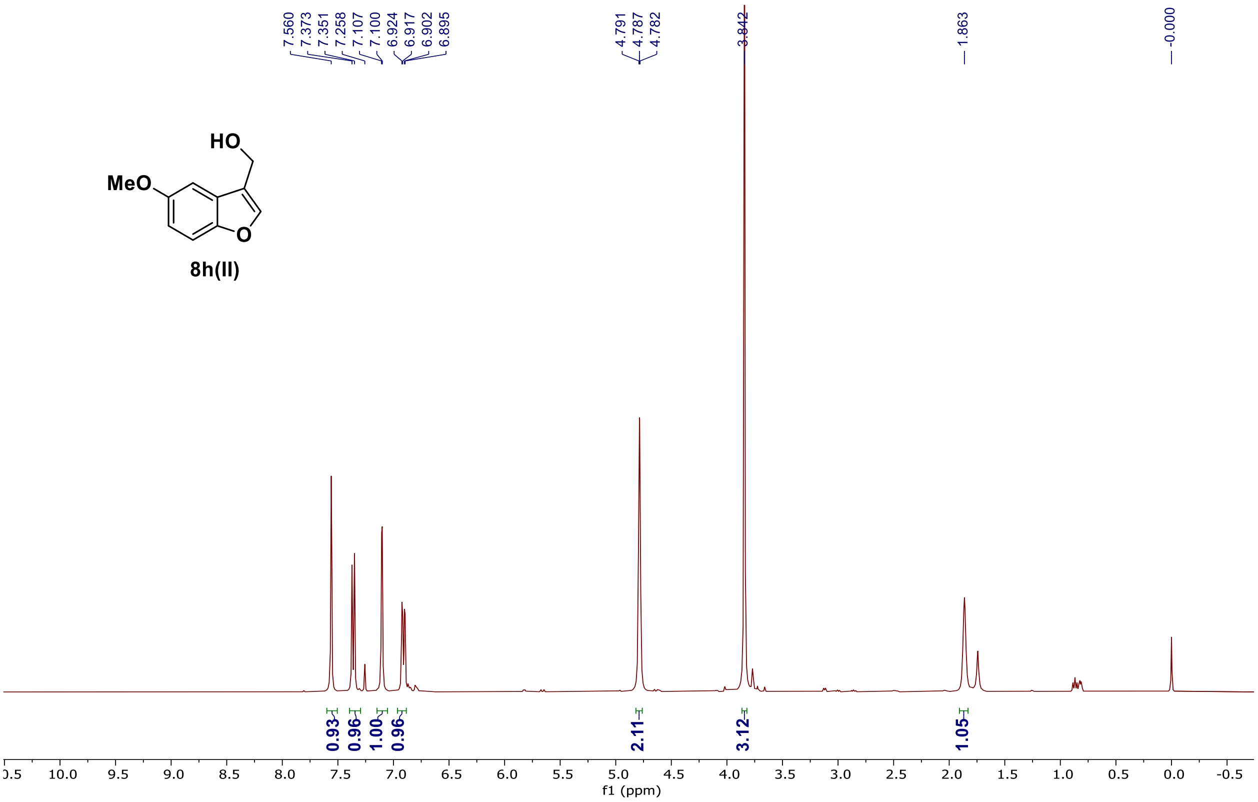

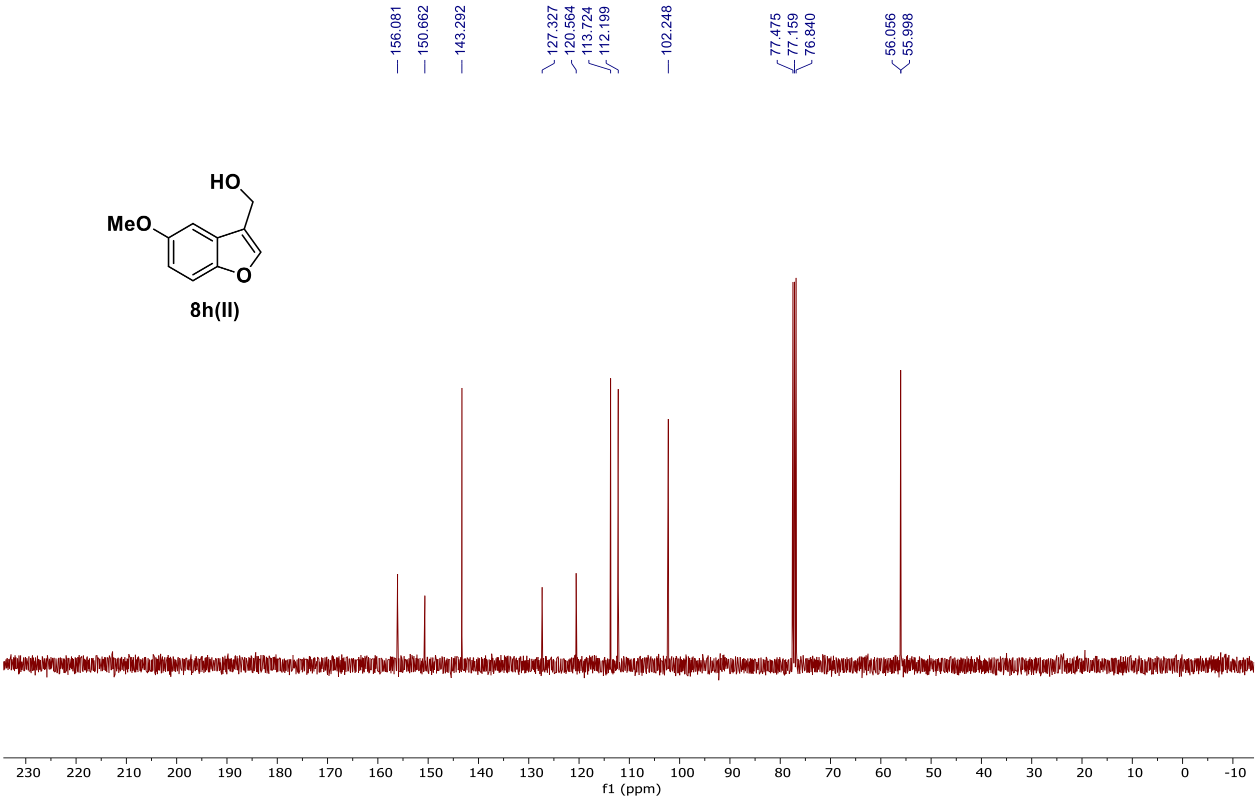

^{1}H and ^{13}C NMR Spectrum of **8h(II)** in $CDCl_3$

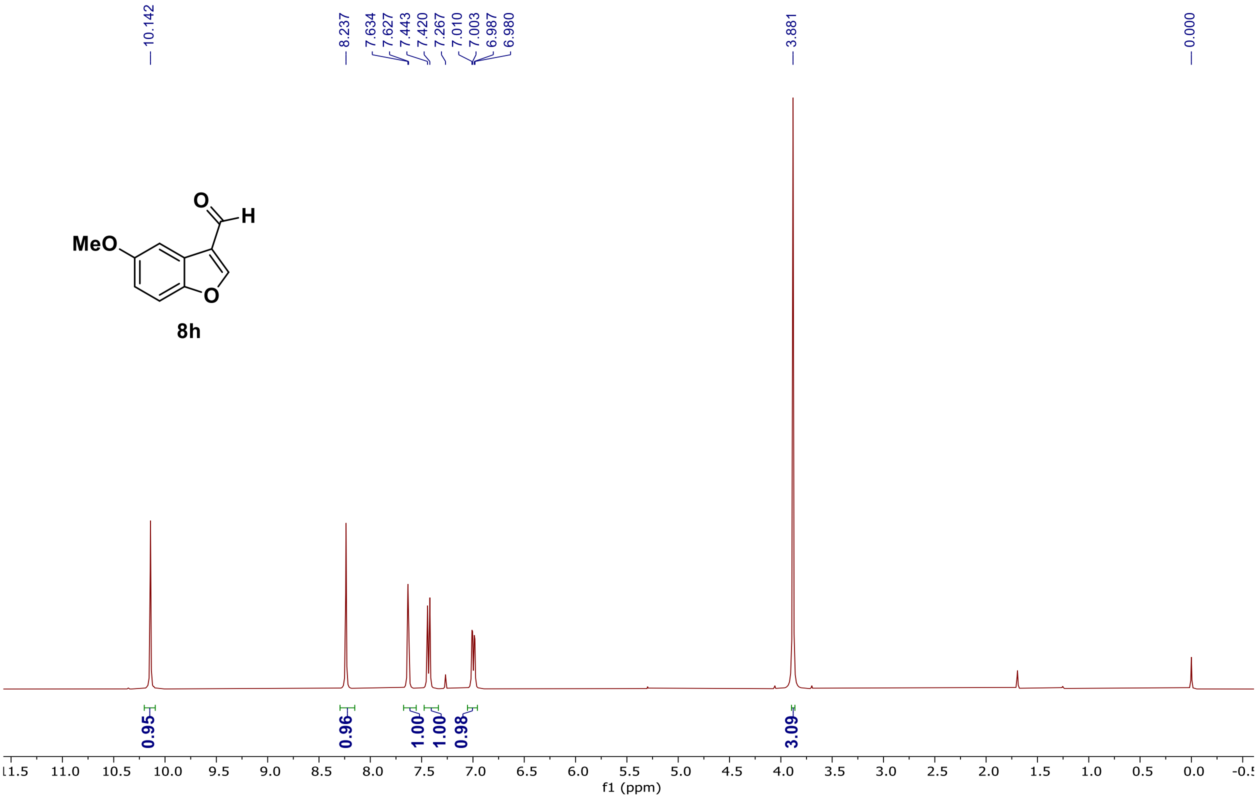

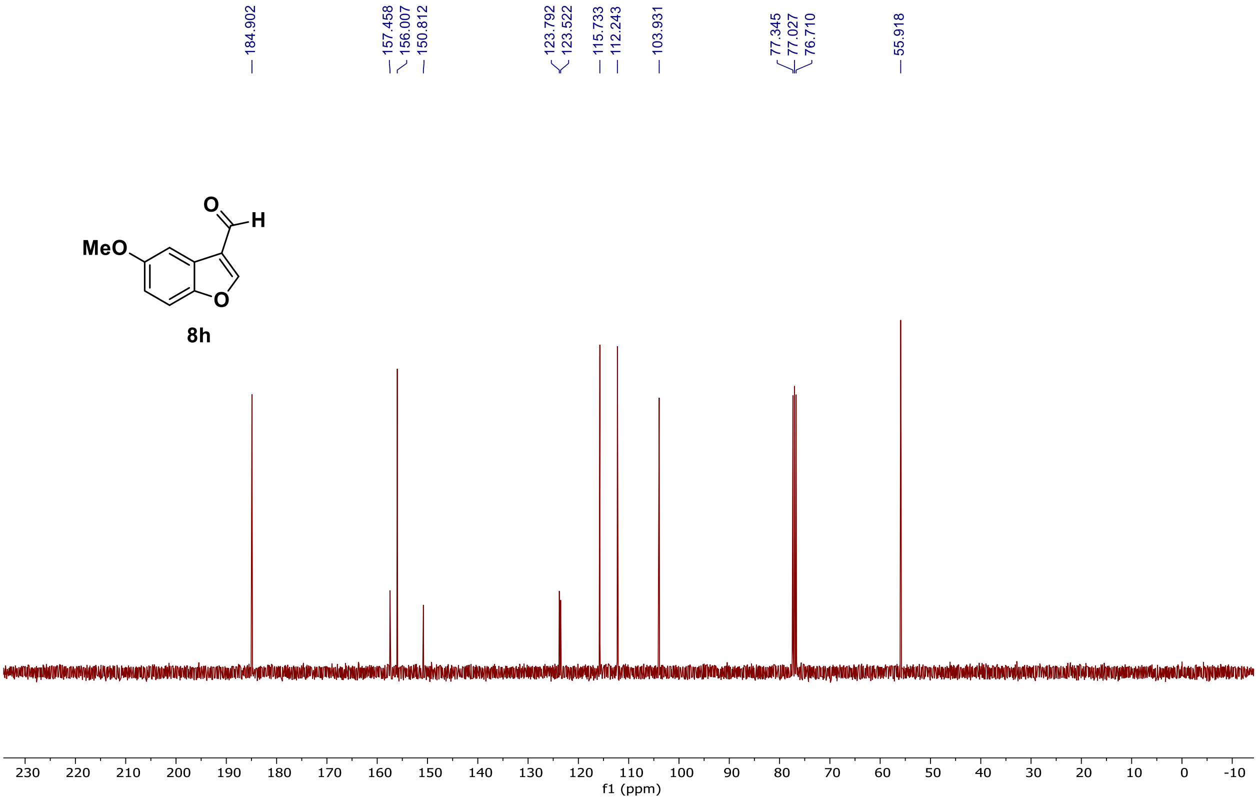

^{1}H and ^{13}C NMR Spectrum of **8h** in $CDCl_3$

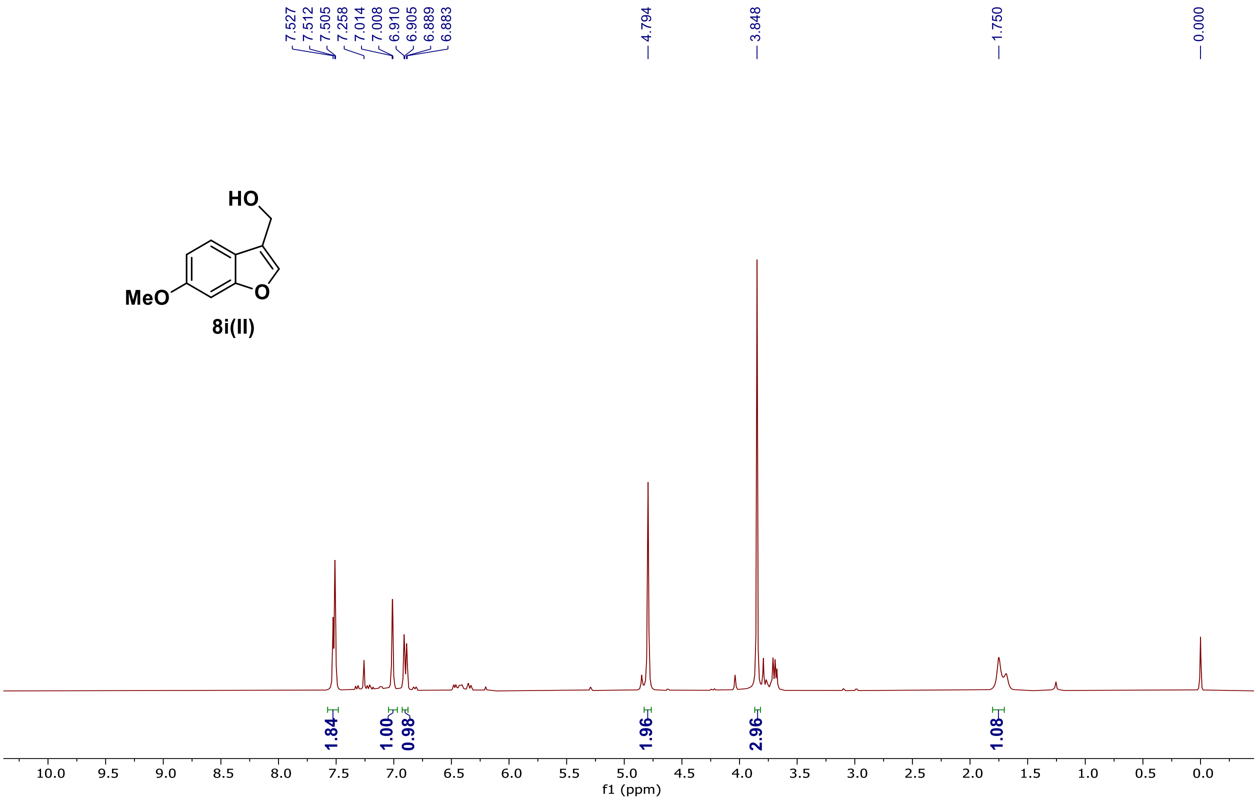

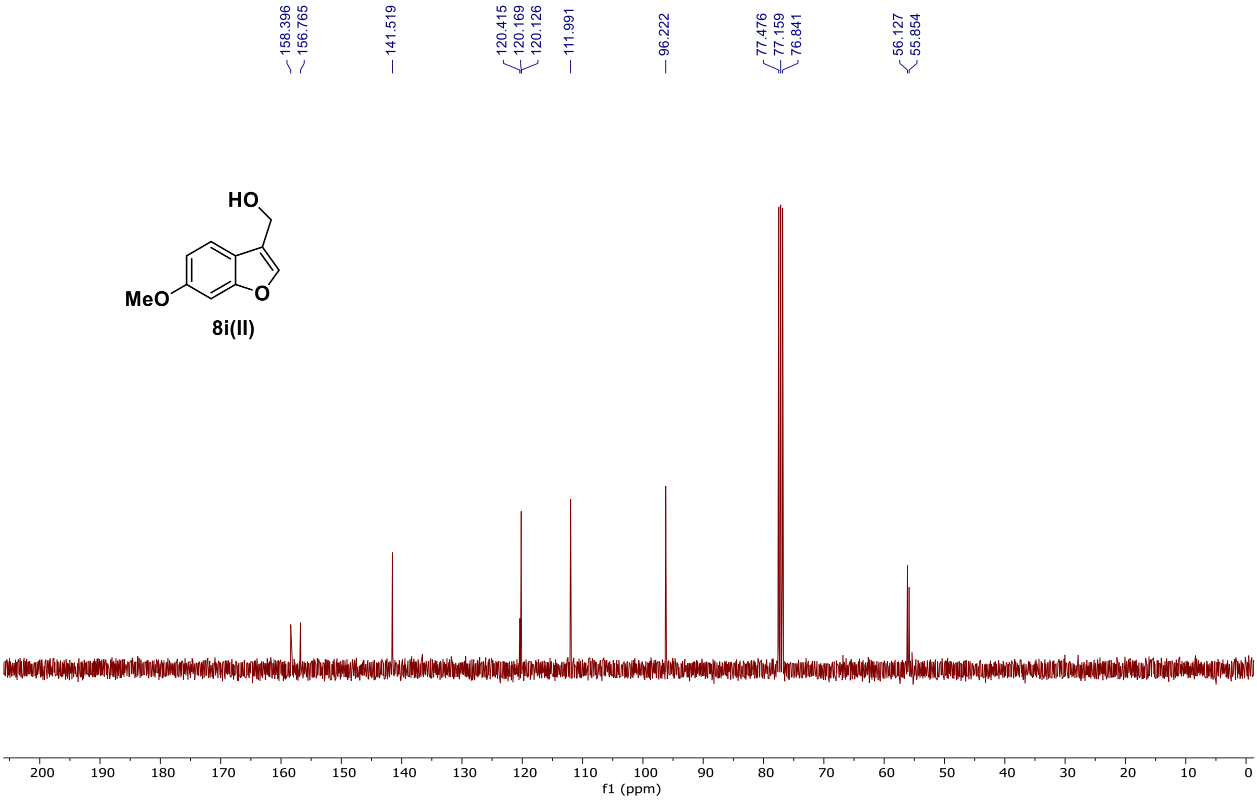

^{1}H and ^{13}C NMR Spectrum of **8i(II)** in $CDCl_3$

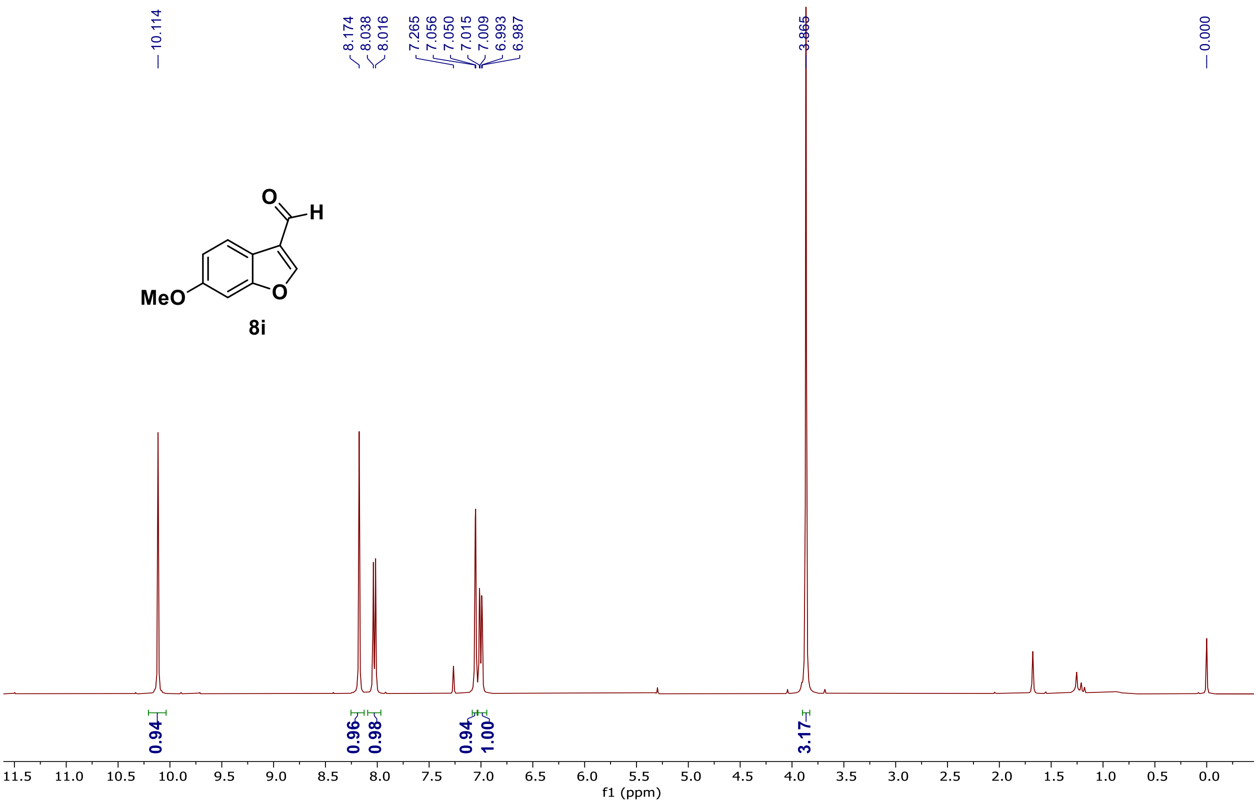

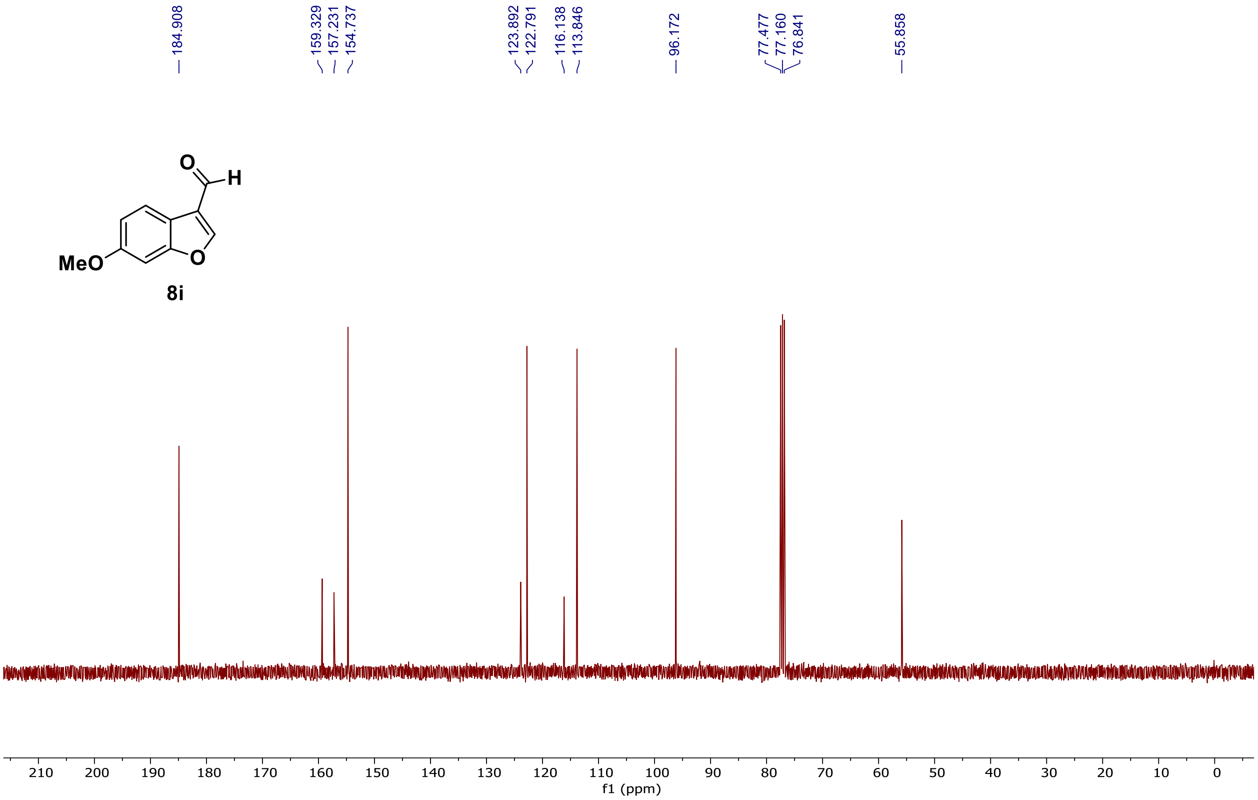

^{1}H and ^{13}C NMR Spectrum of **8i** in $CDCl_3$

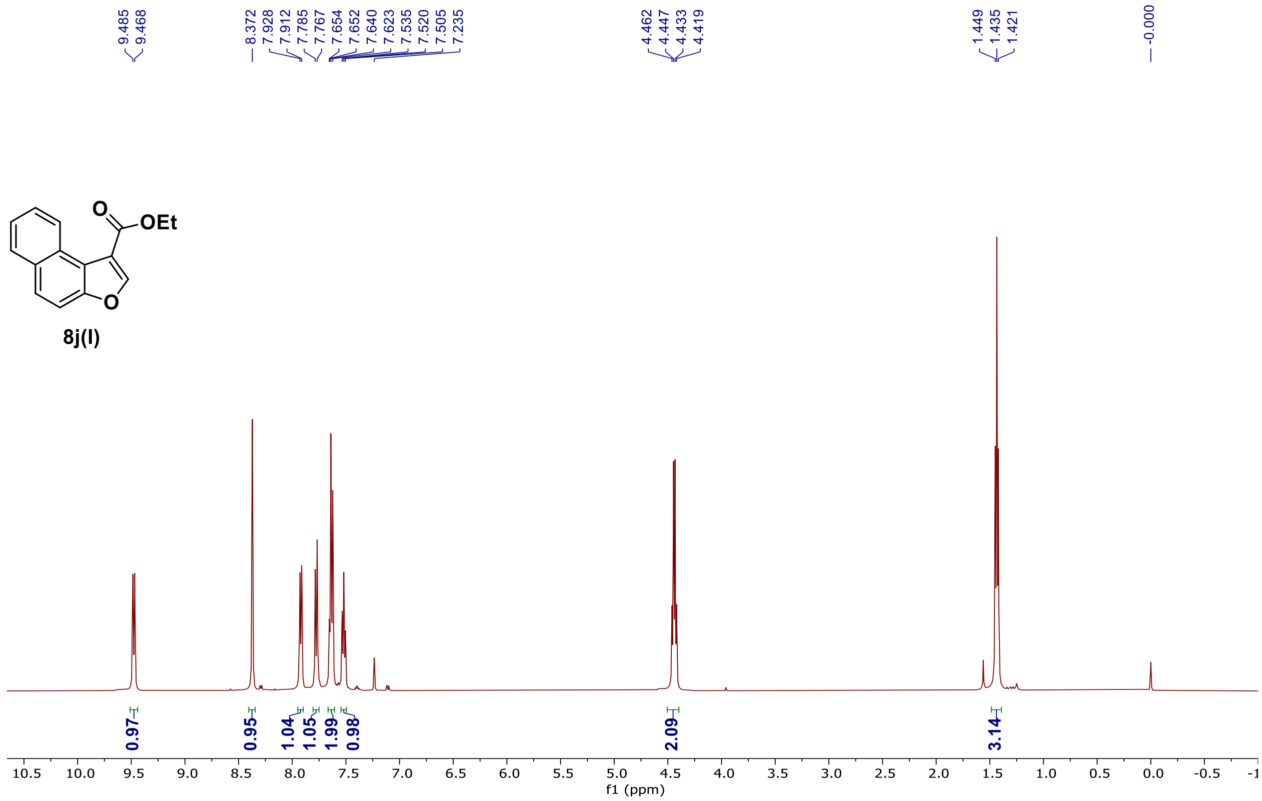

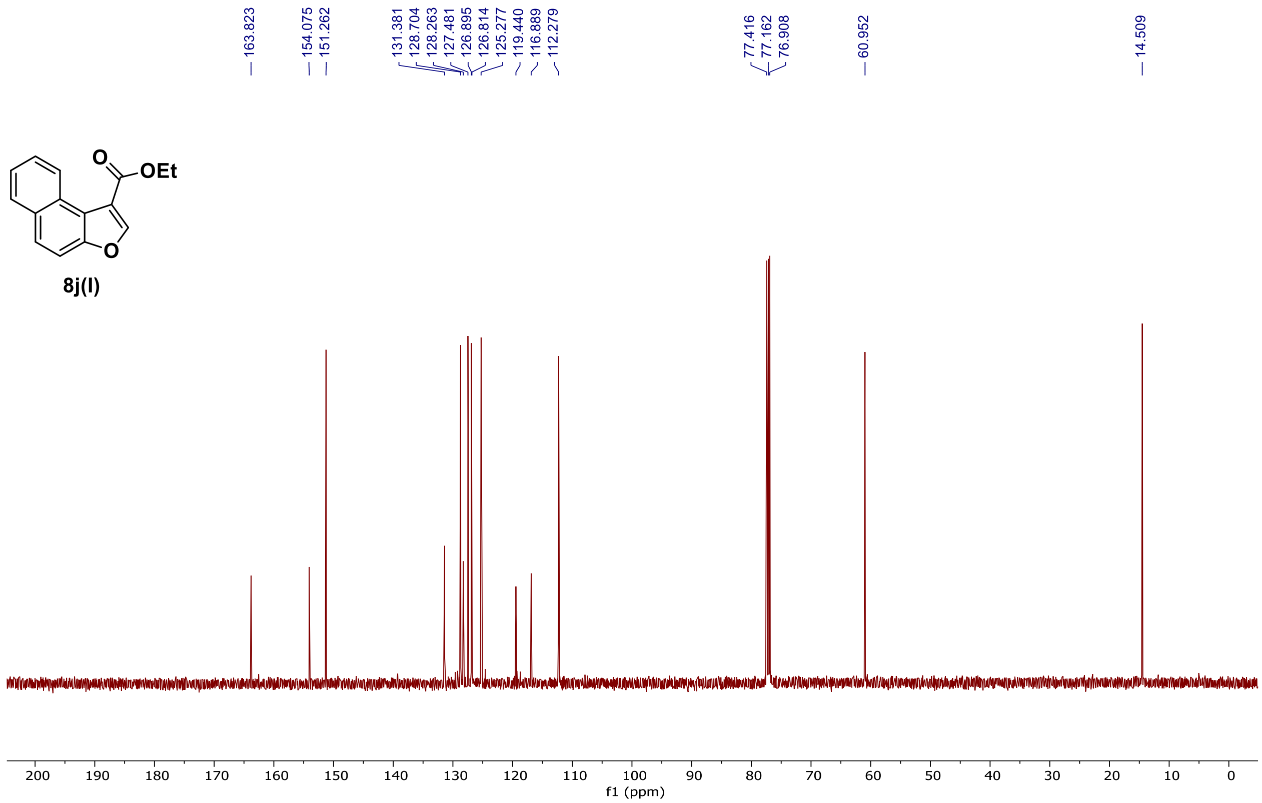

^{1}H and ^{13}C NMR Spectrum of **8j(I)** in $CDCl_3$

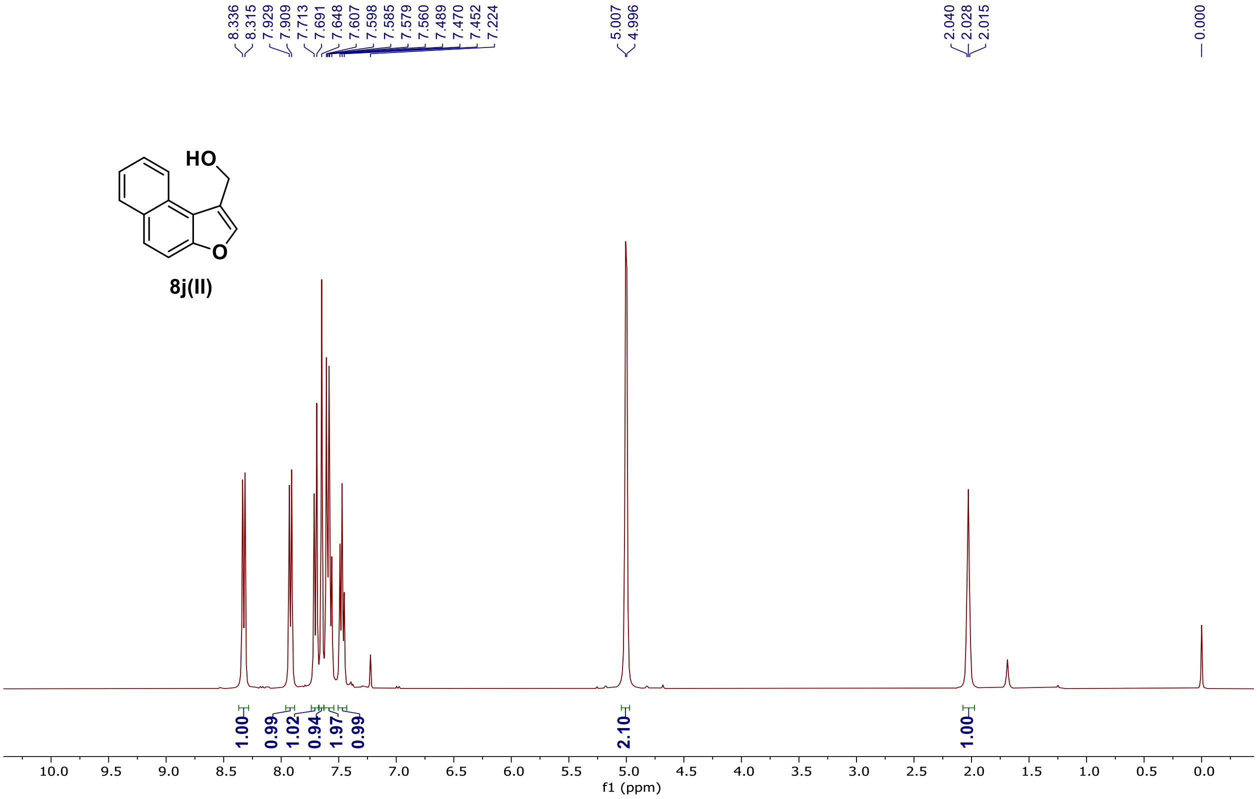

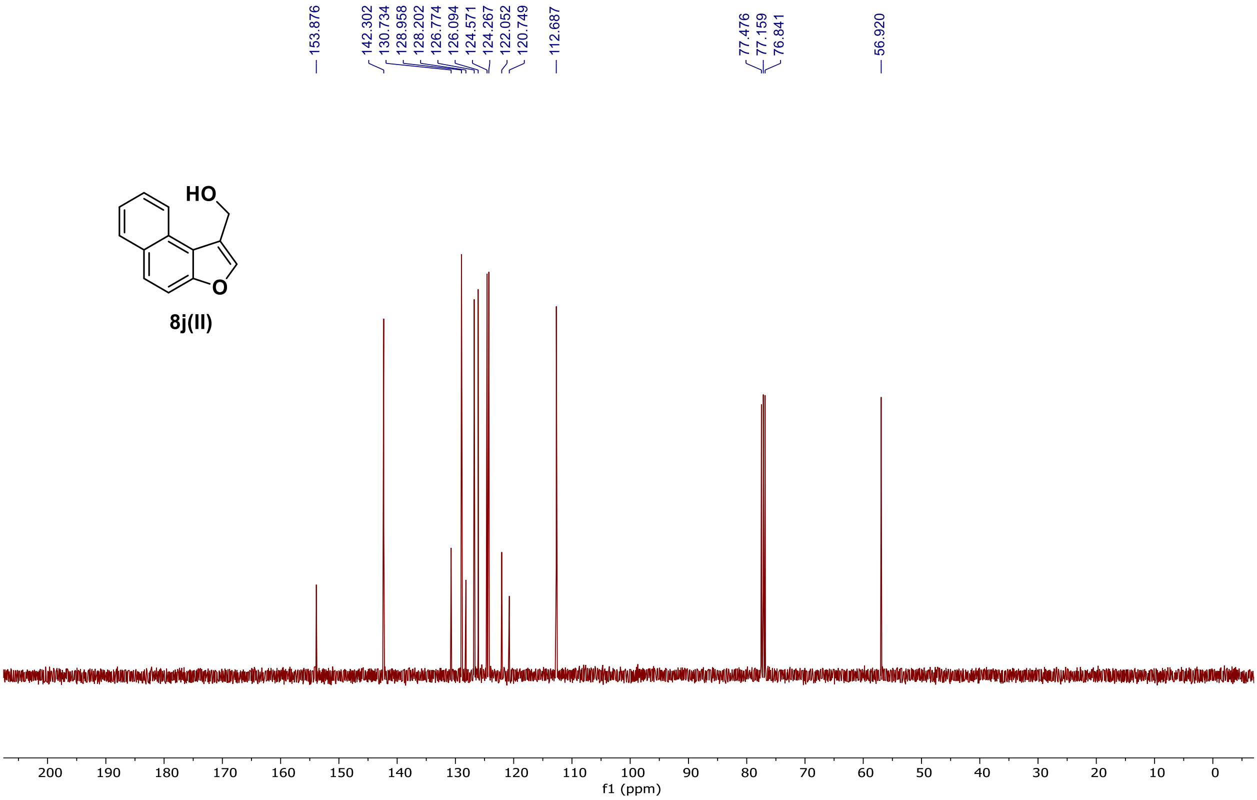

^{1}H and ^{13}C NMR Spectrum of **8j(II)** in $CDCl_3$

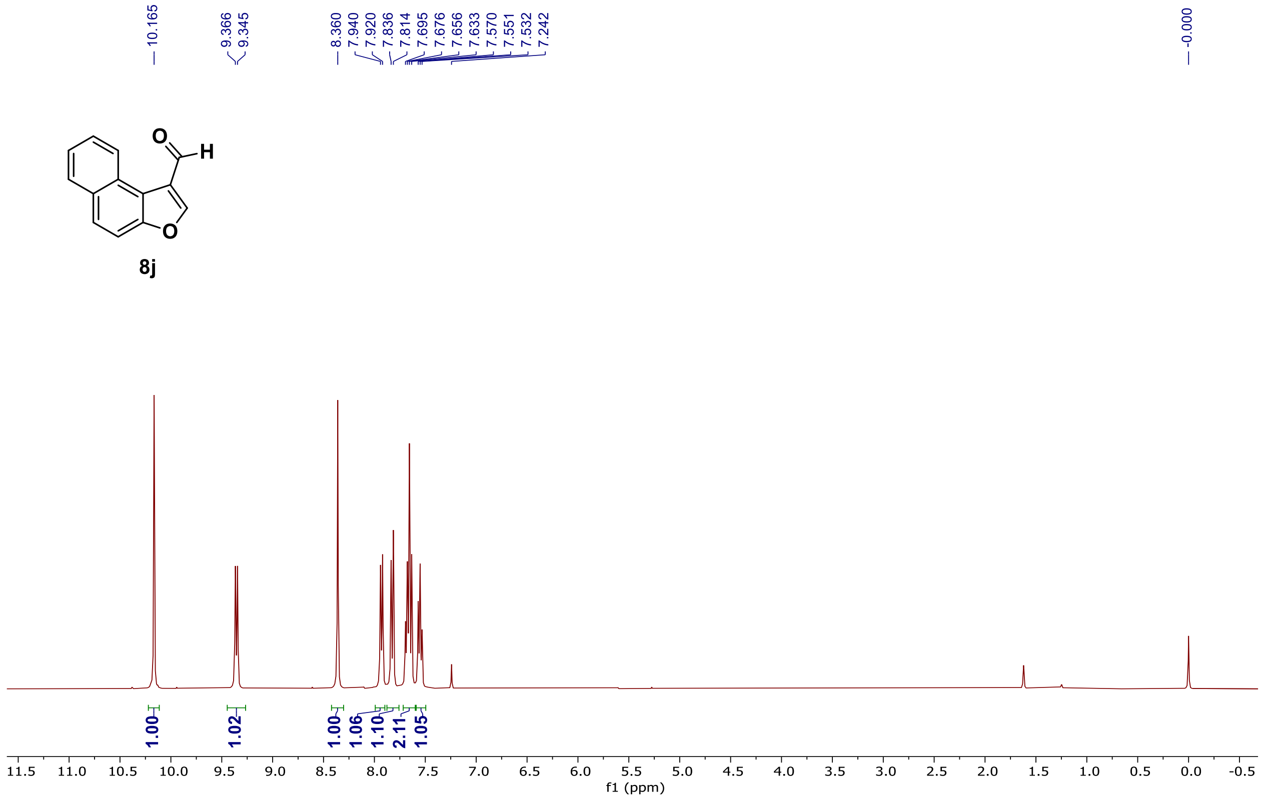

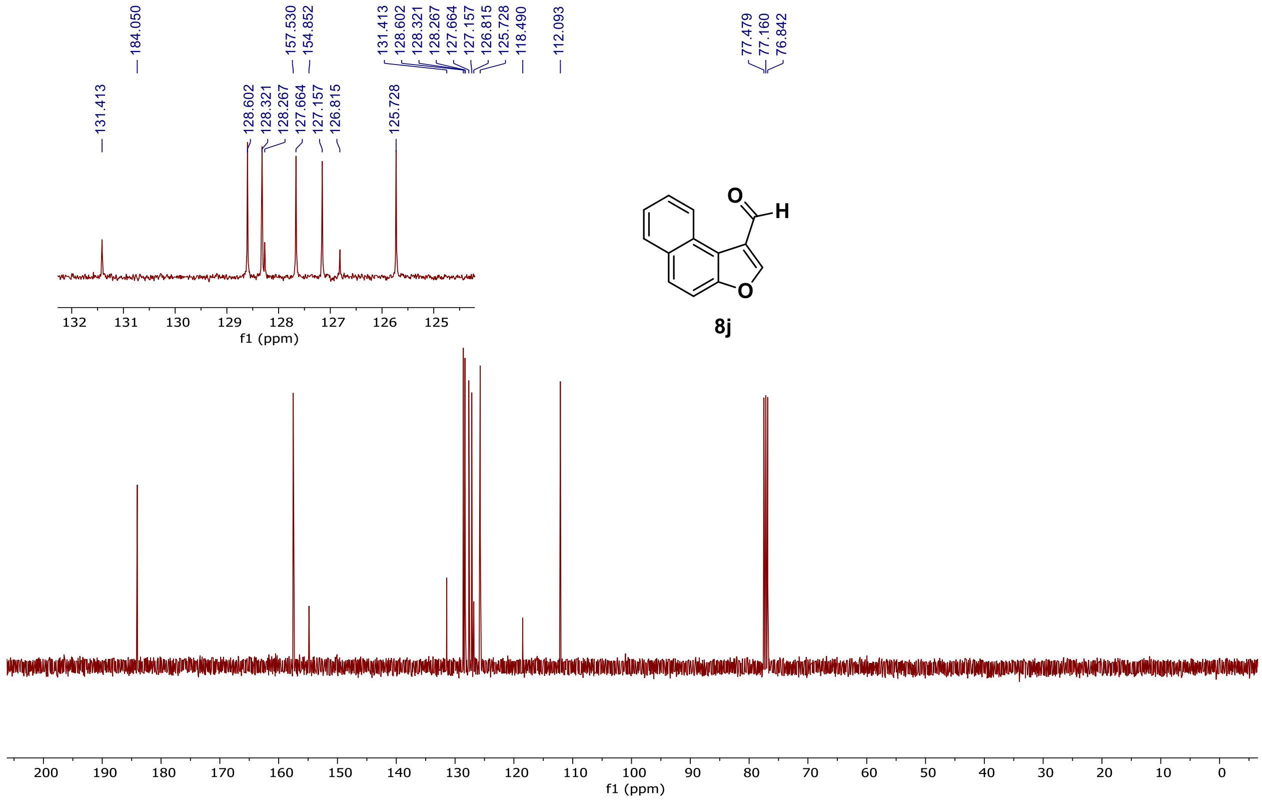

^{1}H and ^{13}C NMR Spectrum of **8j** in $CDCl_3$

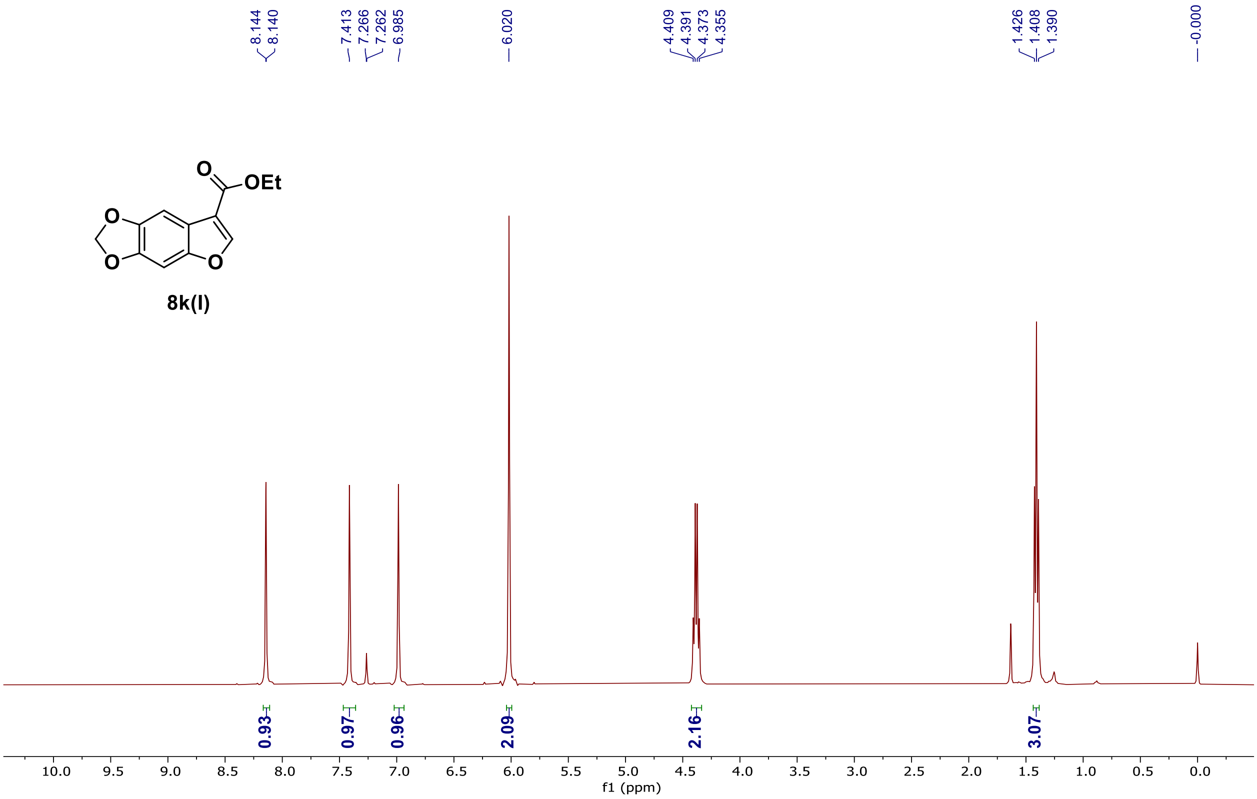

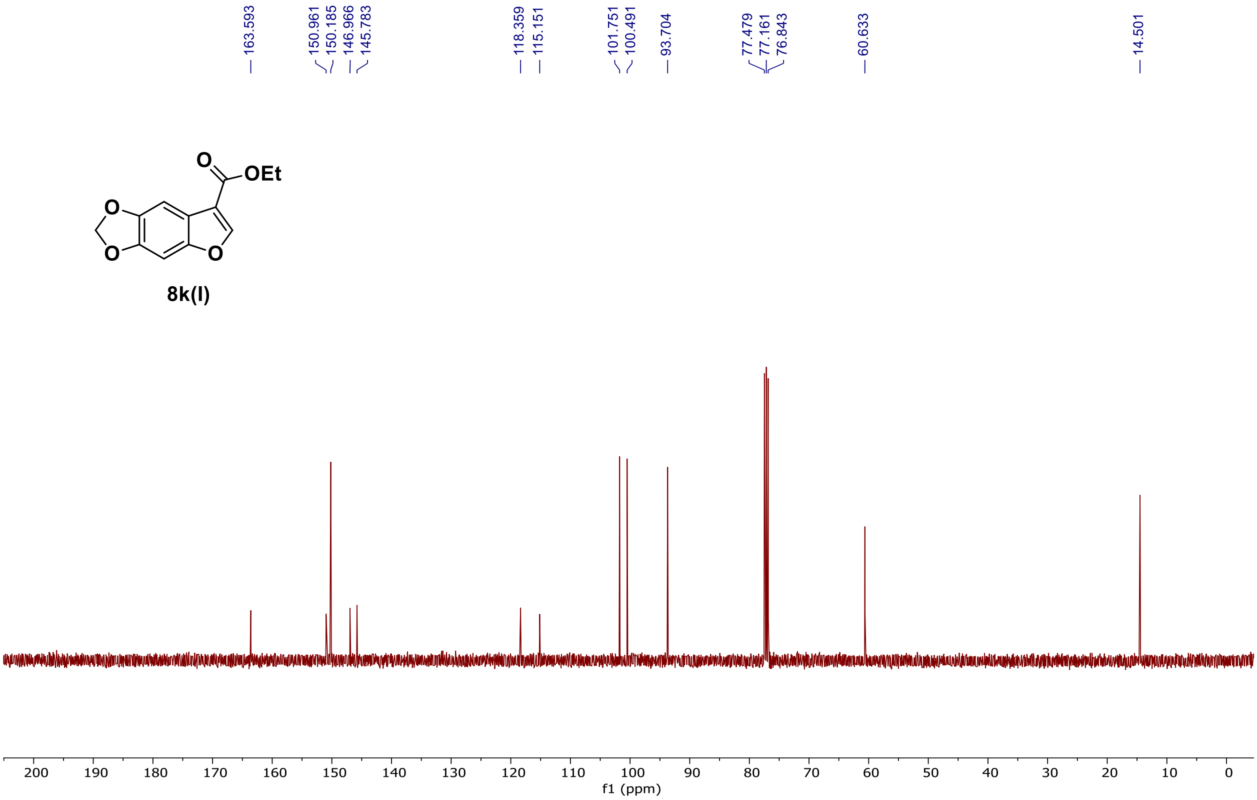

^{1}H and ^{13}C NMR Spectrum of **8k(I)** in $CDCl_3$

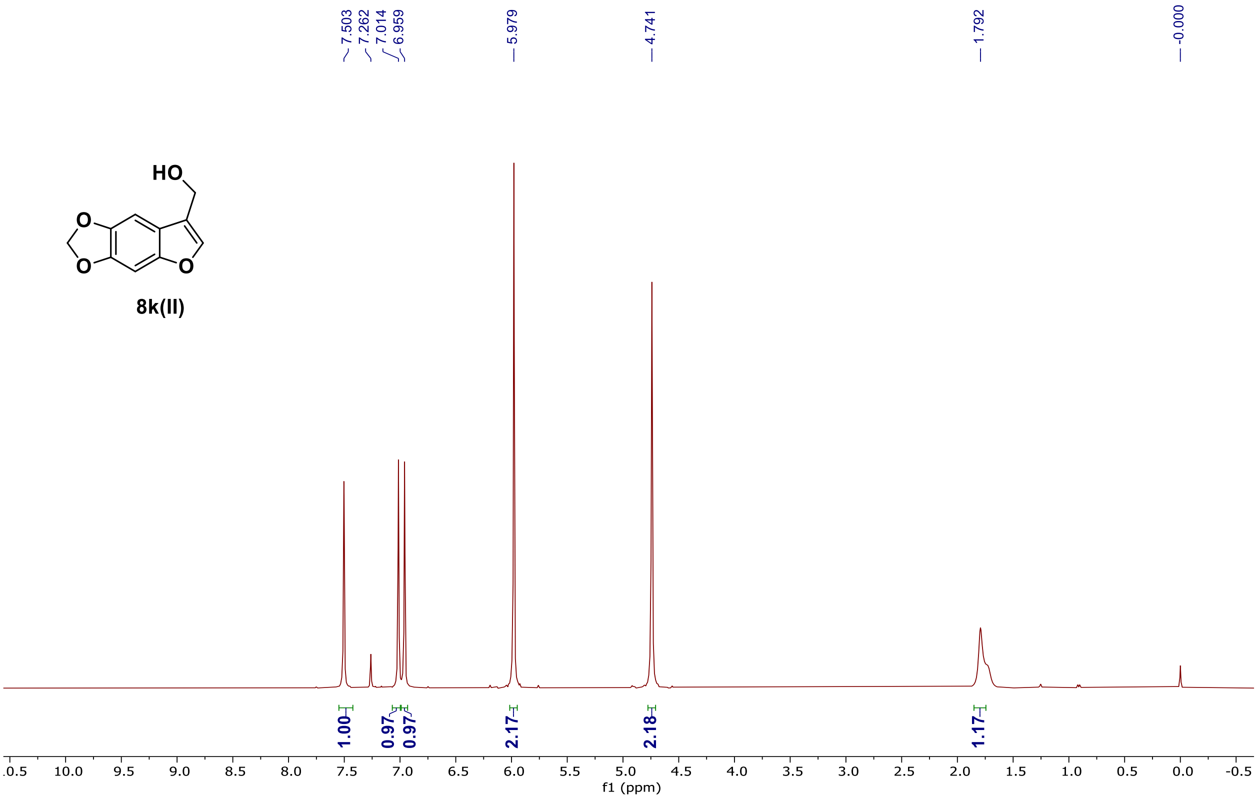

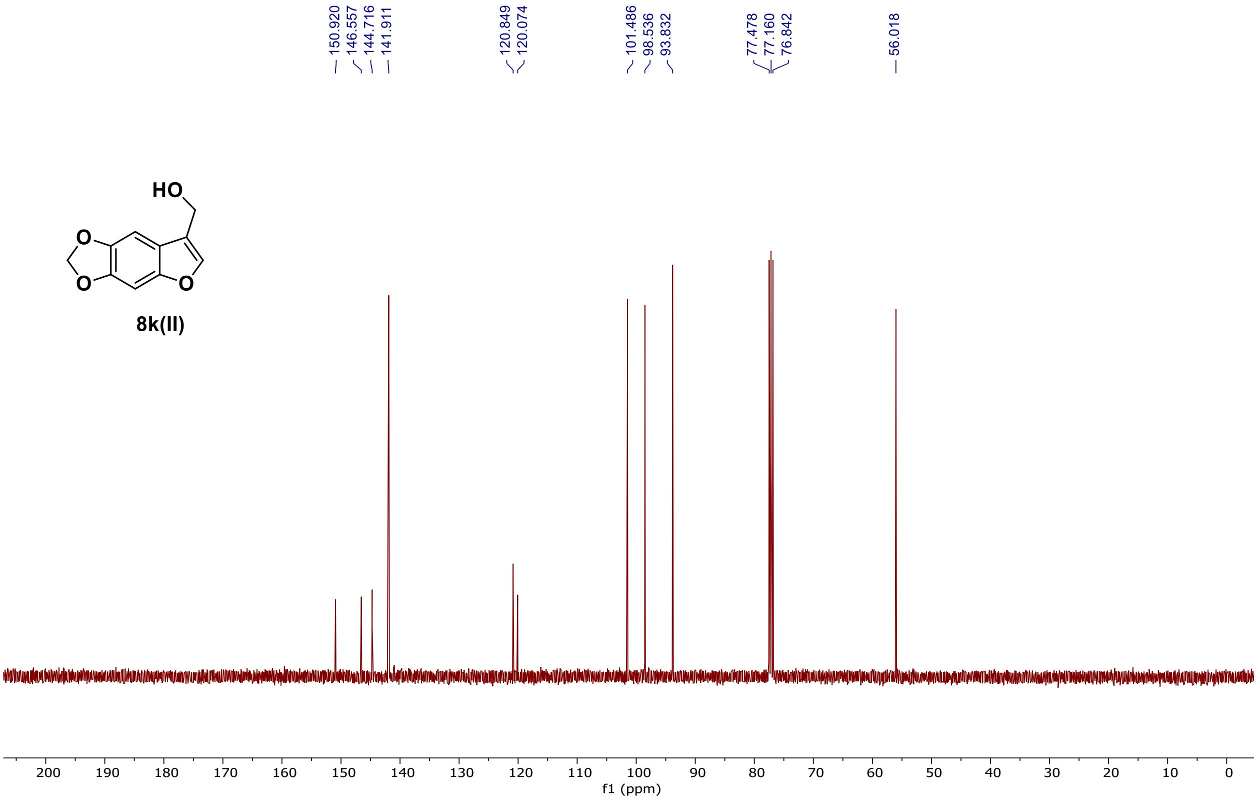

^{1}H and ^{13}C NMR Spectrum of **8k(II)** in $CDCl_3$

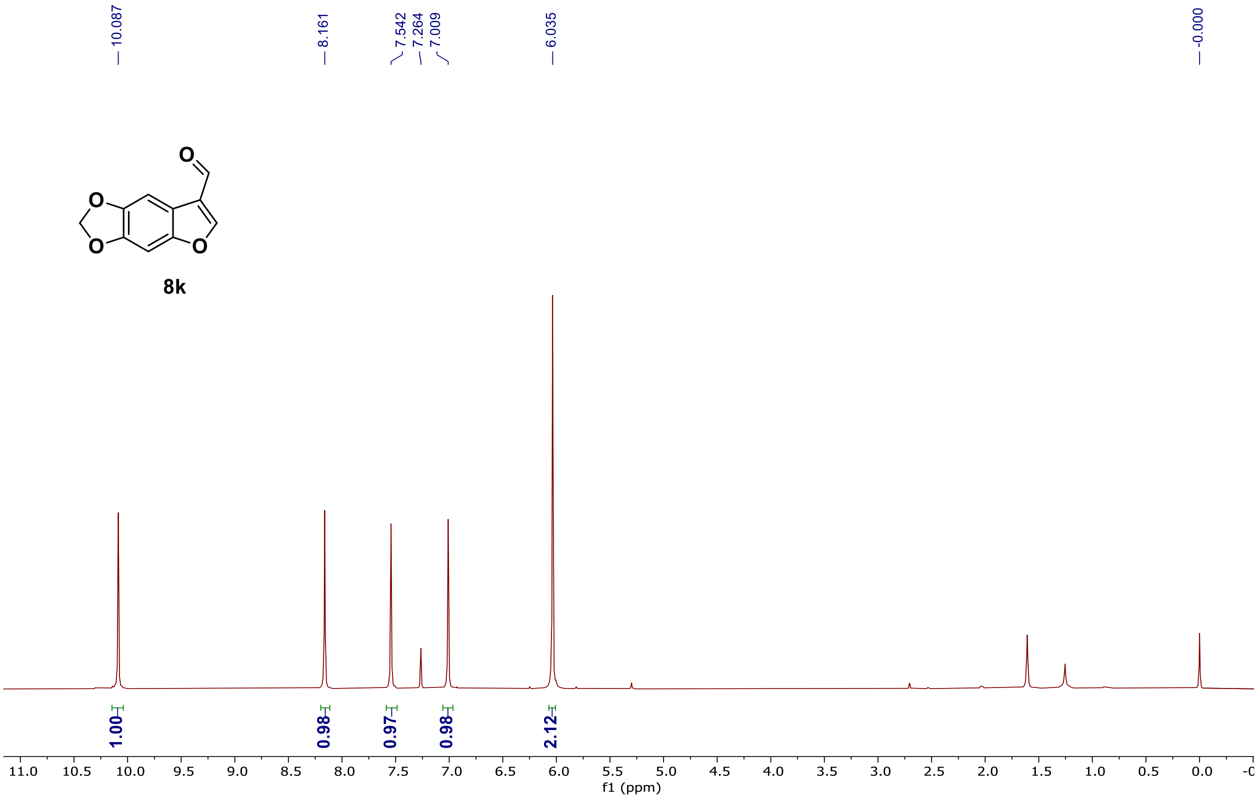

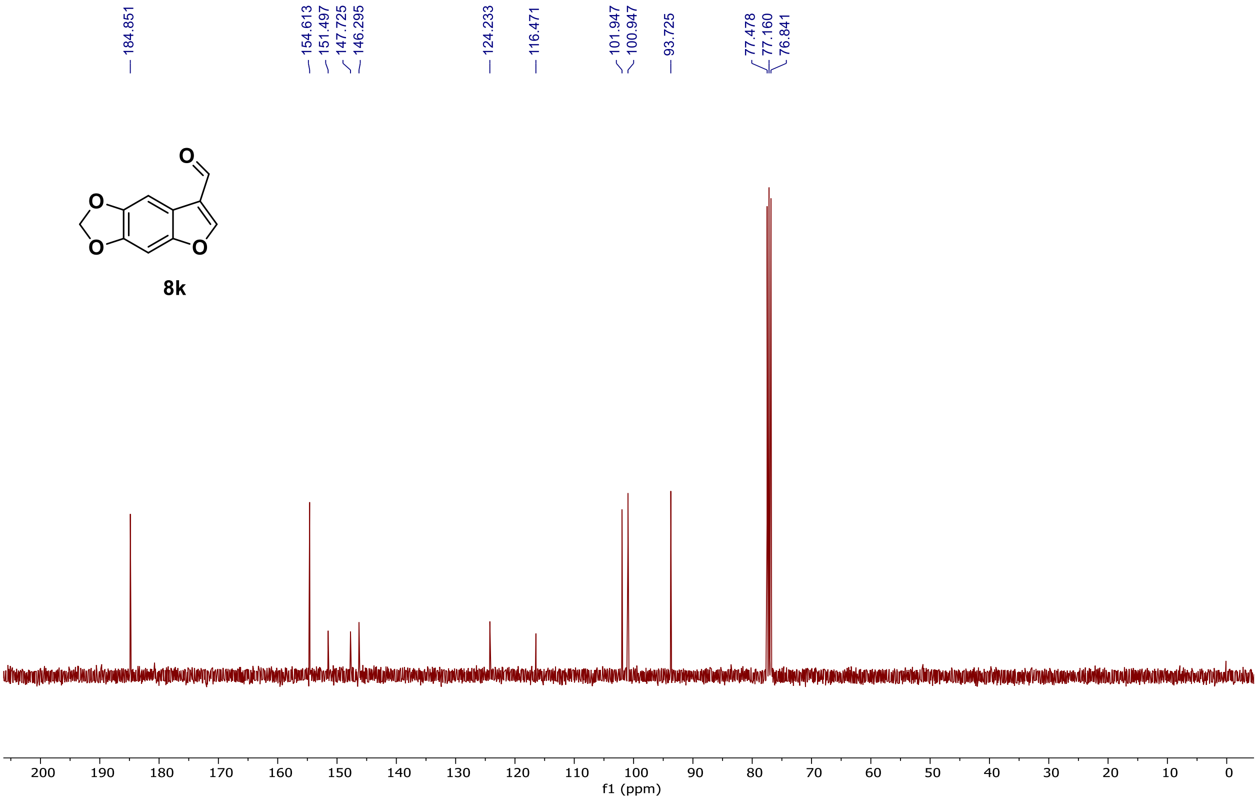

^{1}H and ^{13}C NMR Spectrum of **8k** in $CDCl_3$

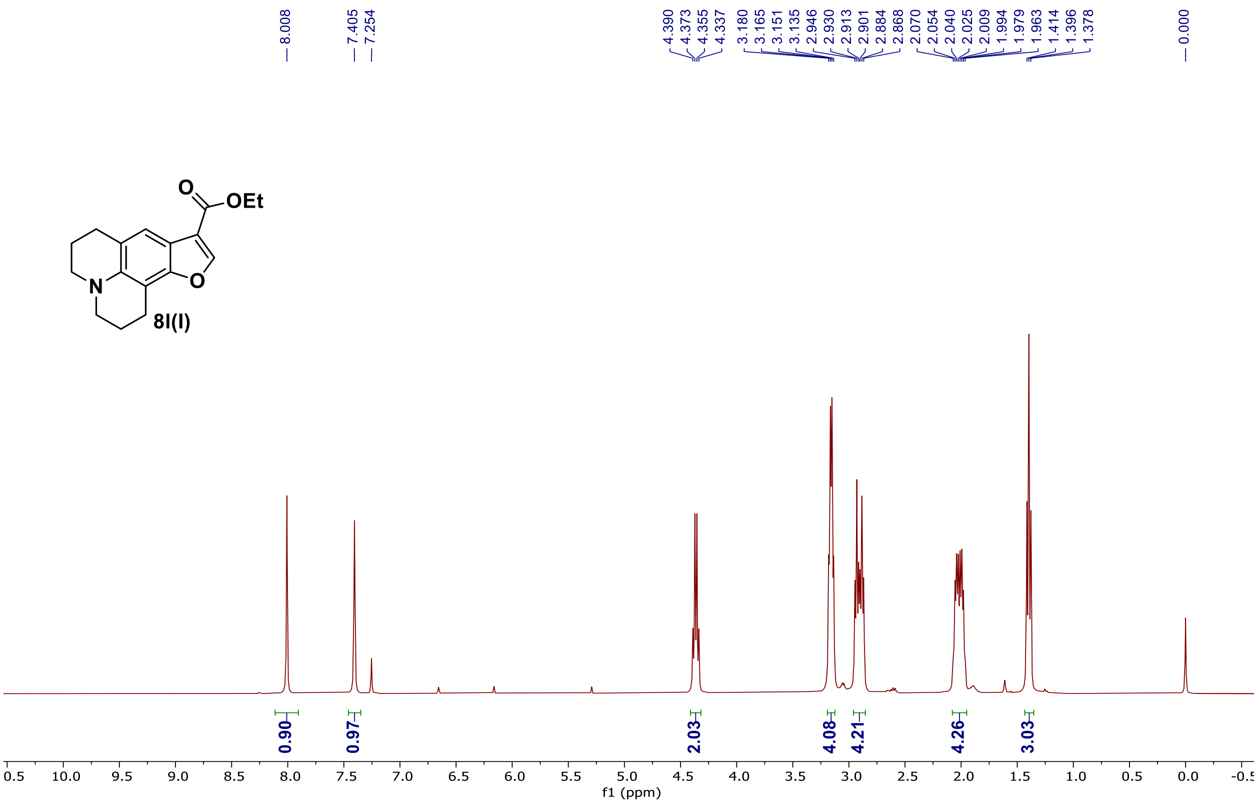

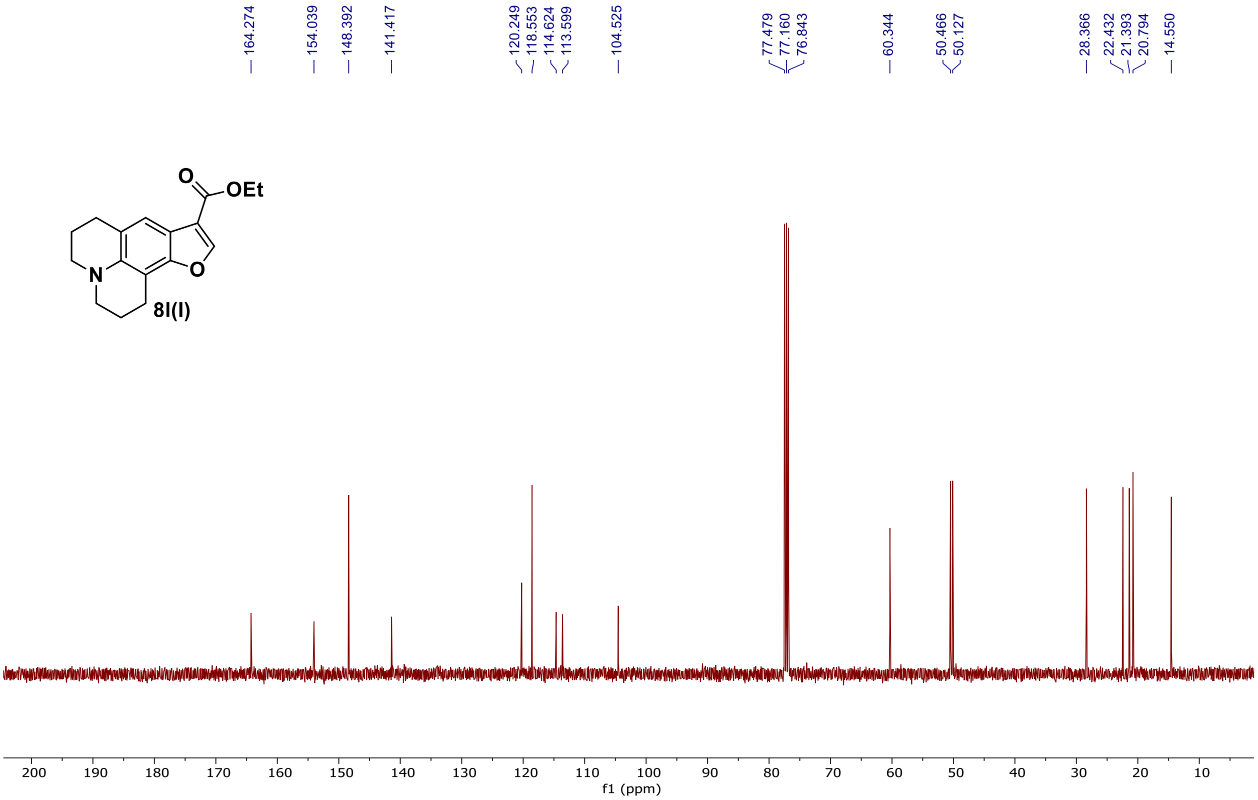

^{1}H and ^{13}C NMR Spectrum of **8l(I)** in $CDCl_3$

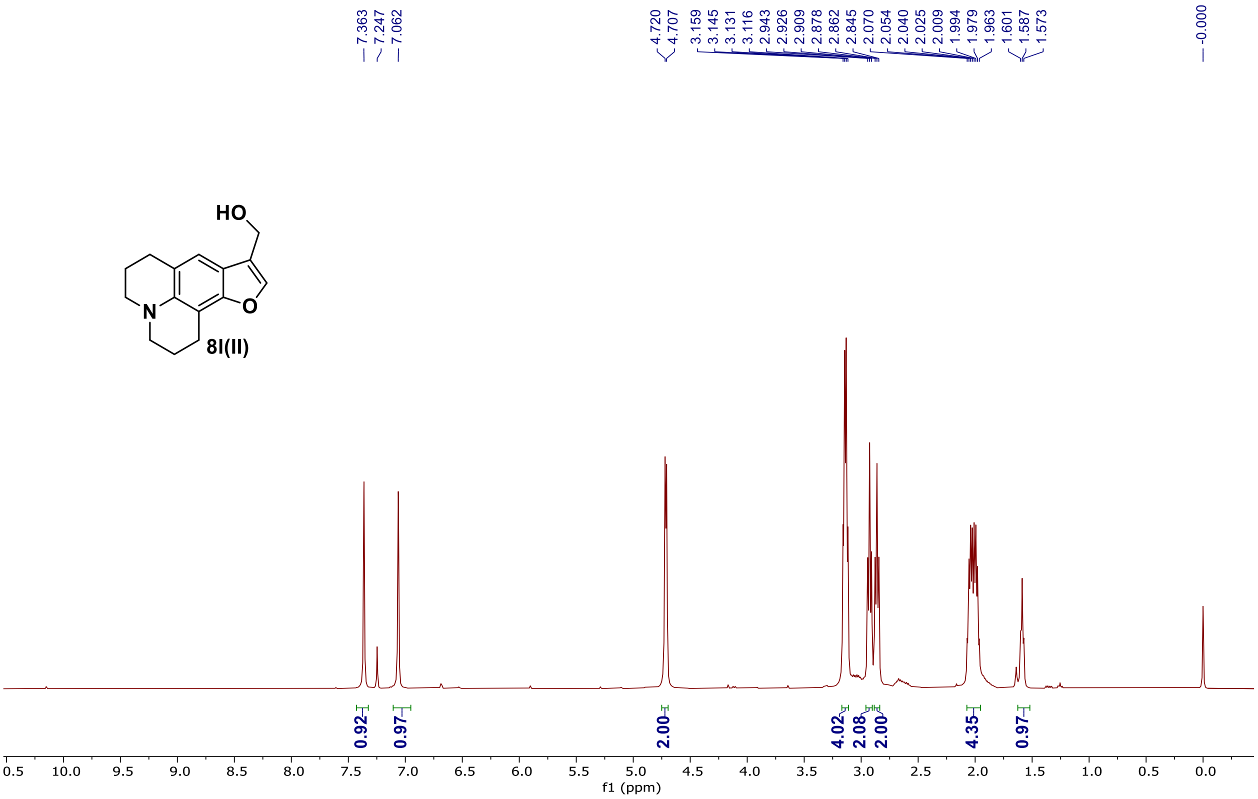

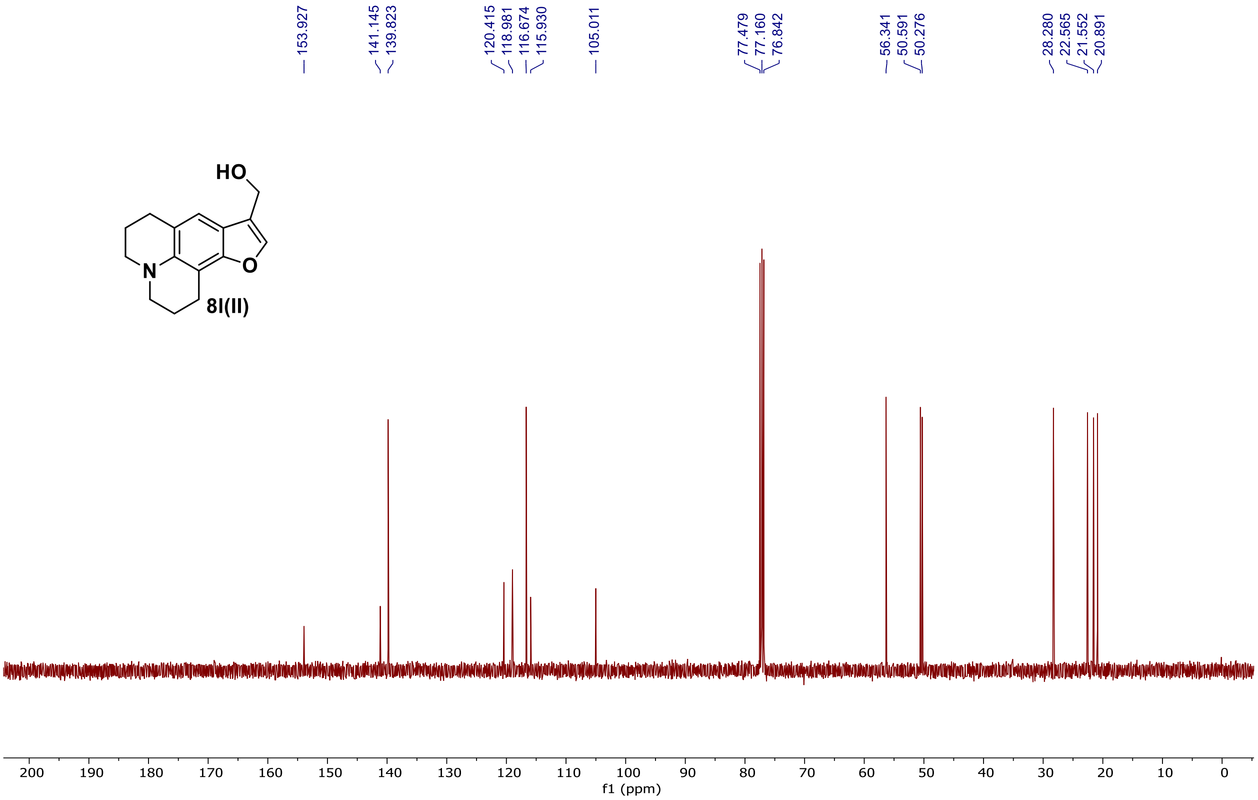

^{1}H and ^{13}C NMR Spectrum of **8l(II)** in $CDCl_3$

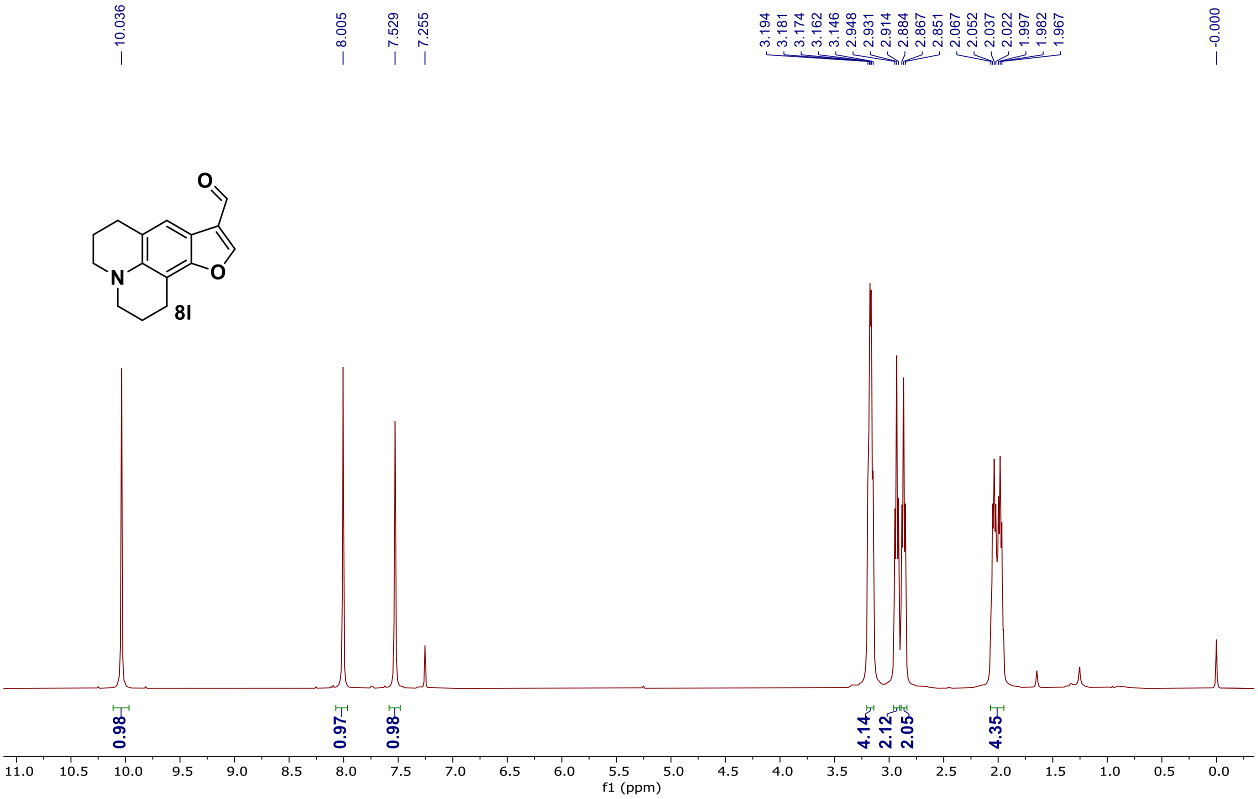

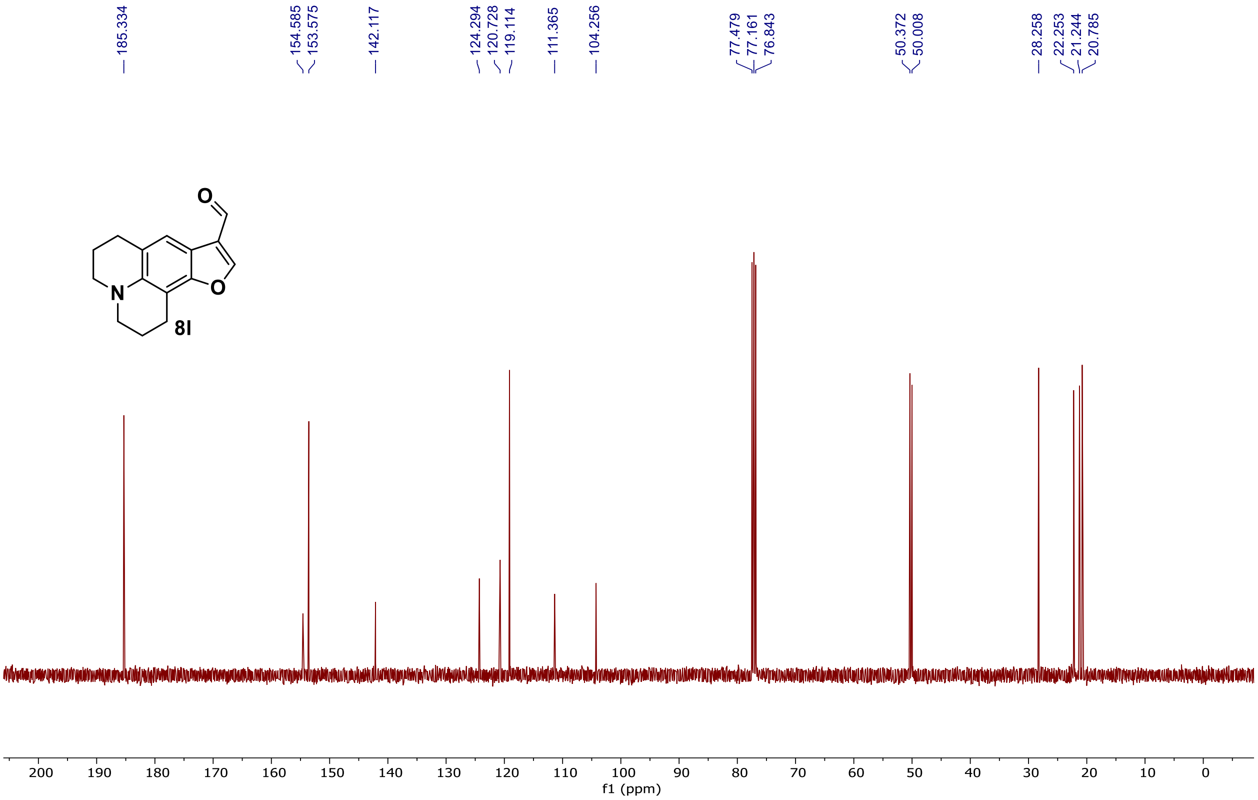

^{1}H and ^{13}C NMR Spectrum of **8l** in $CDCl_3$

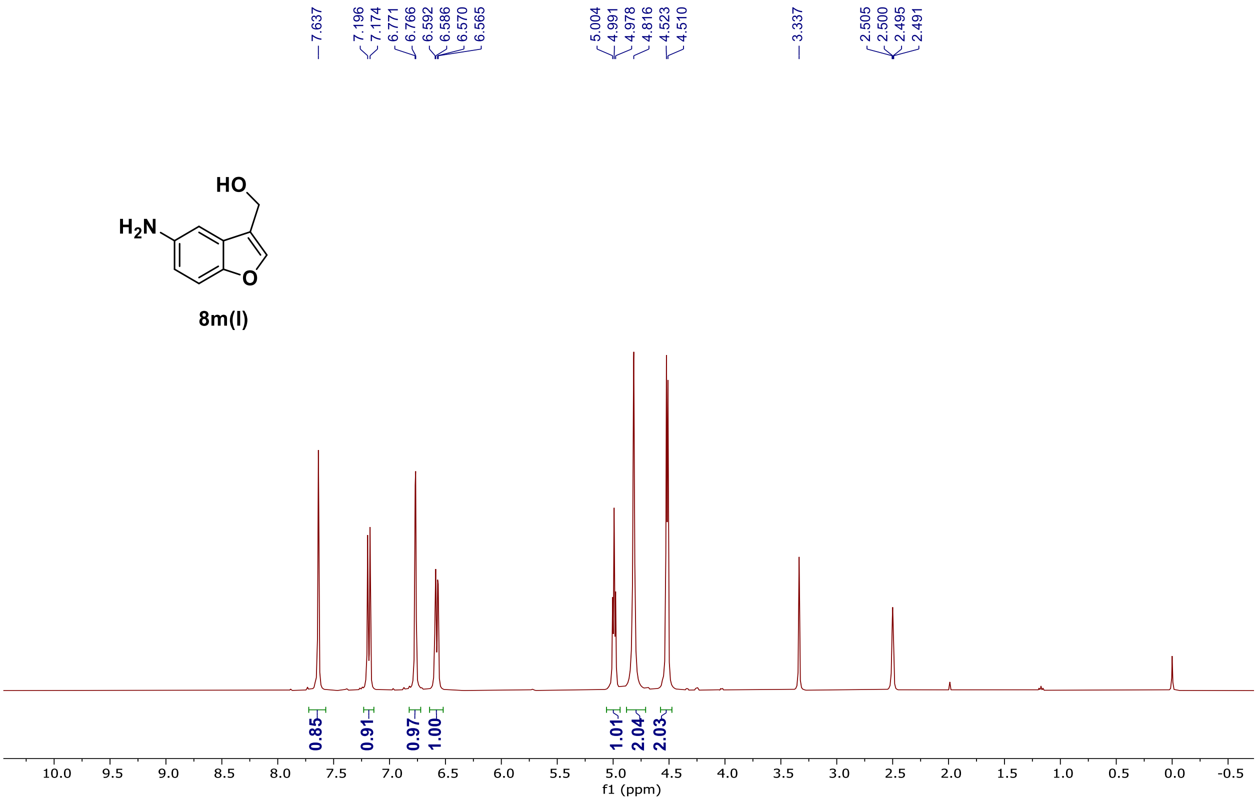

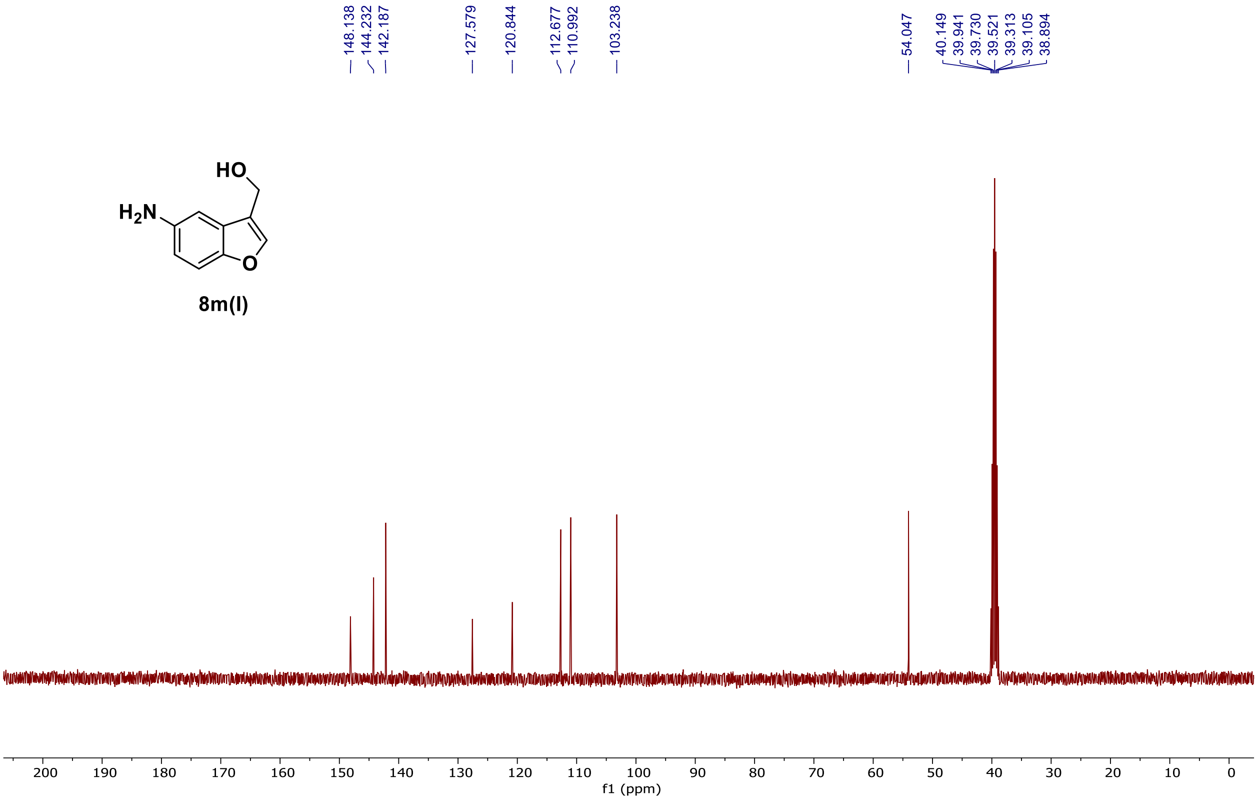

^{1}H and ^{13}C NMR Spectrum of **8m(I)** in DMSO-d_6

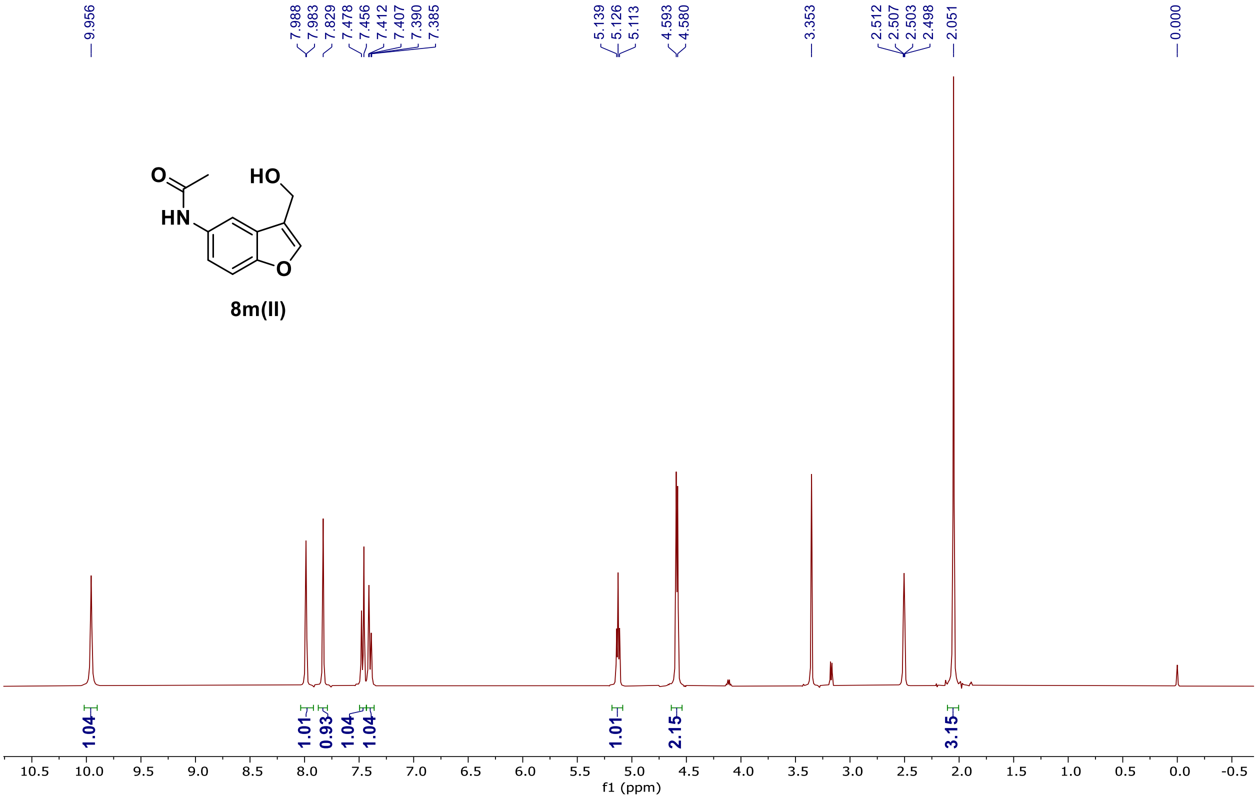

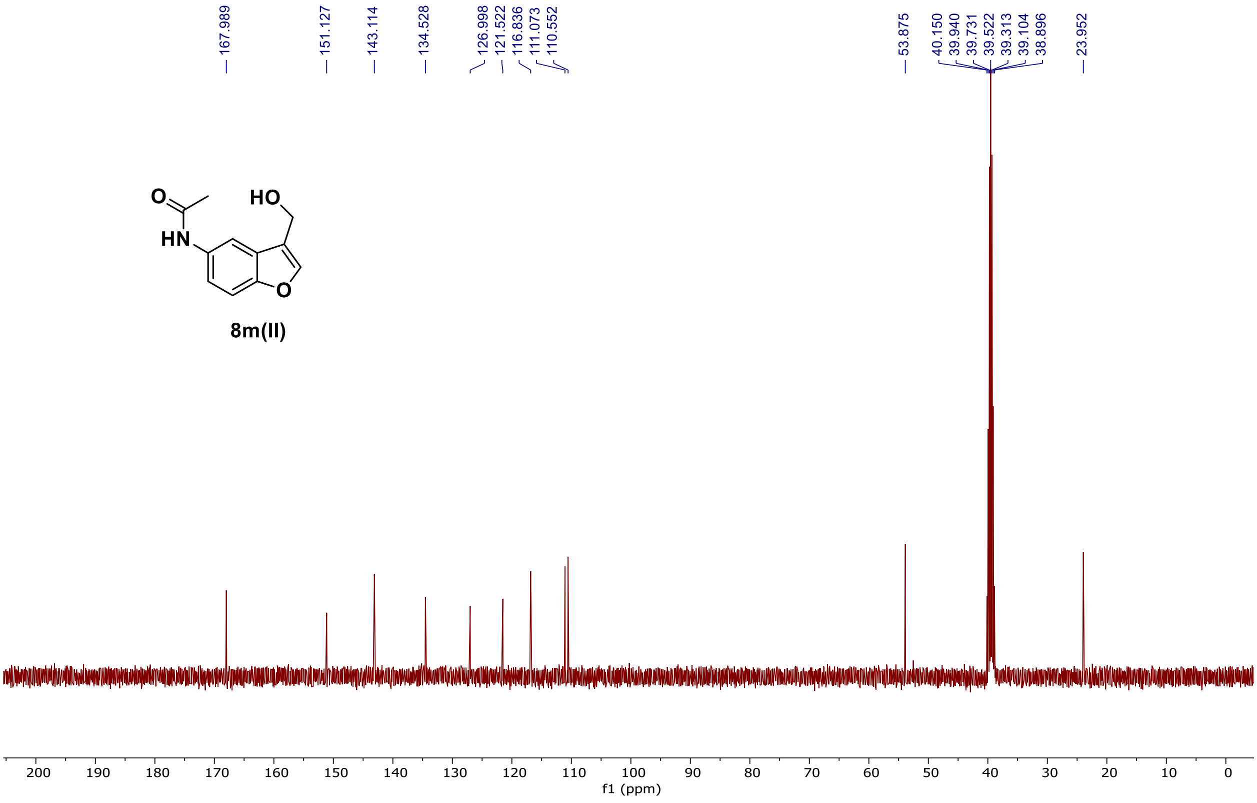

^{1}H and ^{13}C NMR Spectrum of **8m(II)** in DMSO-d_6

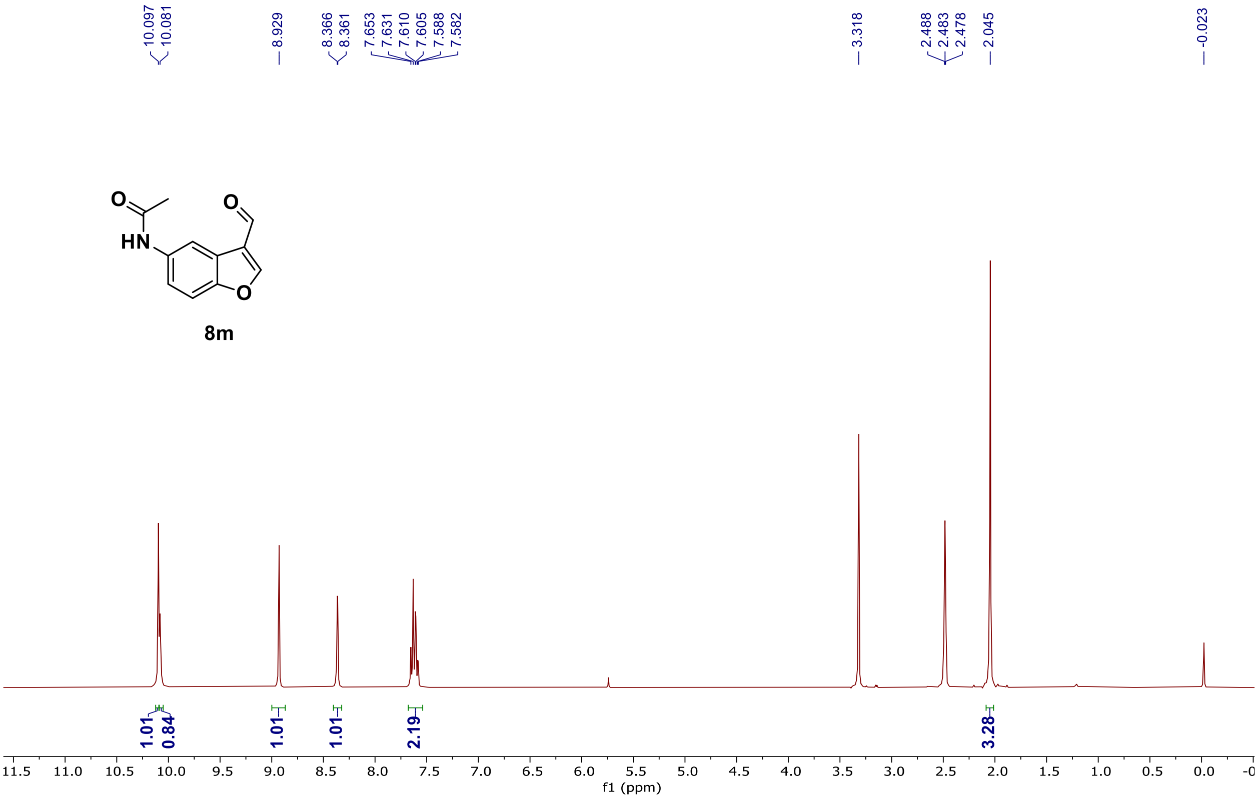

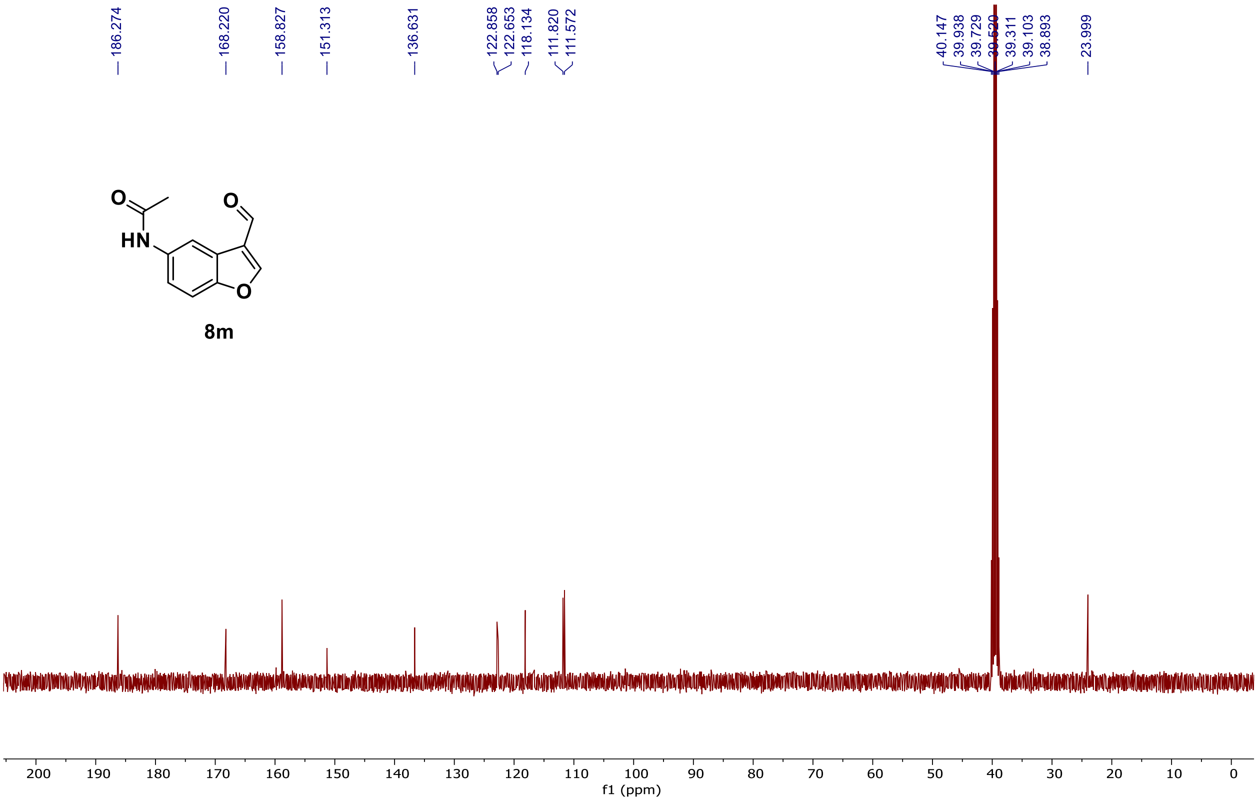

^{1}H and ^{13}C NMR Spectrum of **8k** in DMSO-d_6

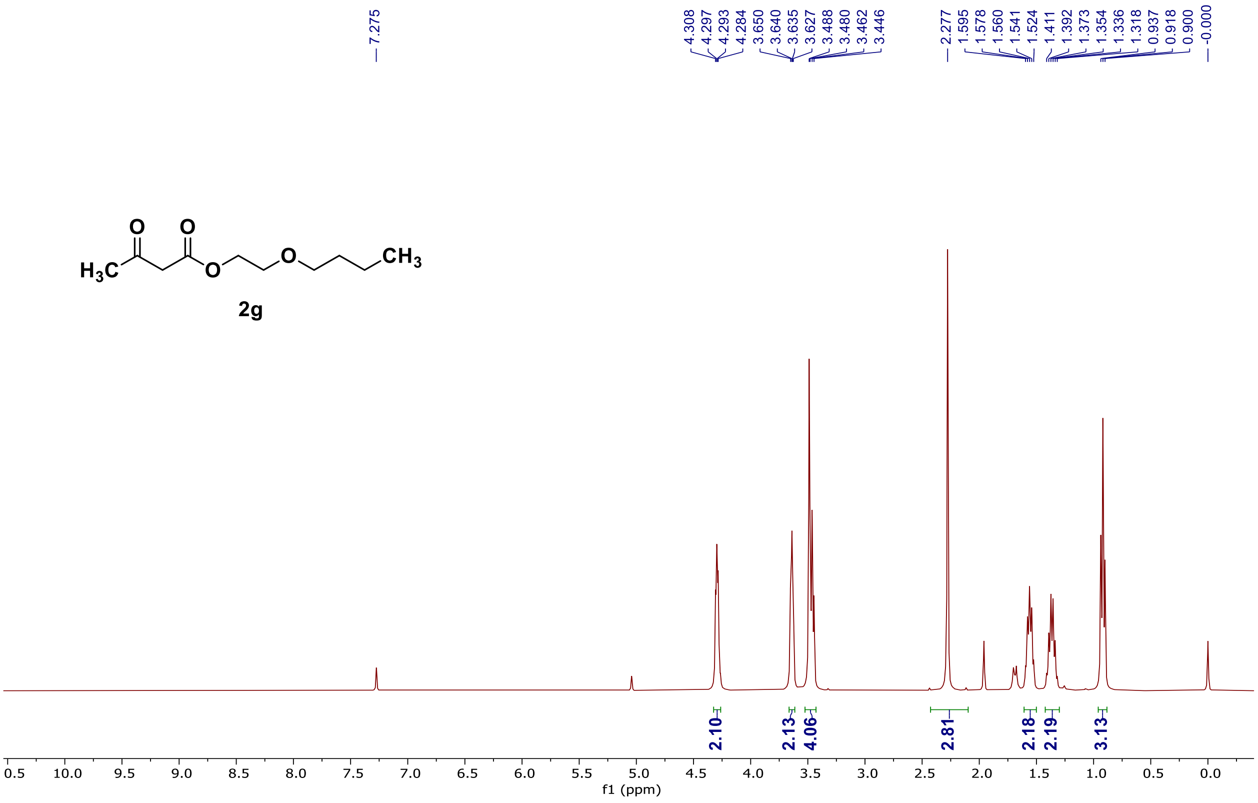

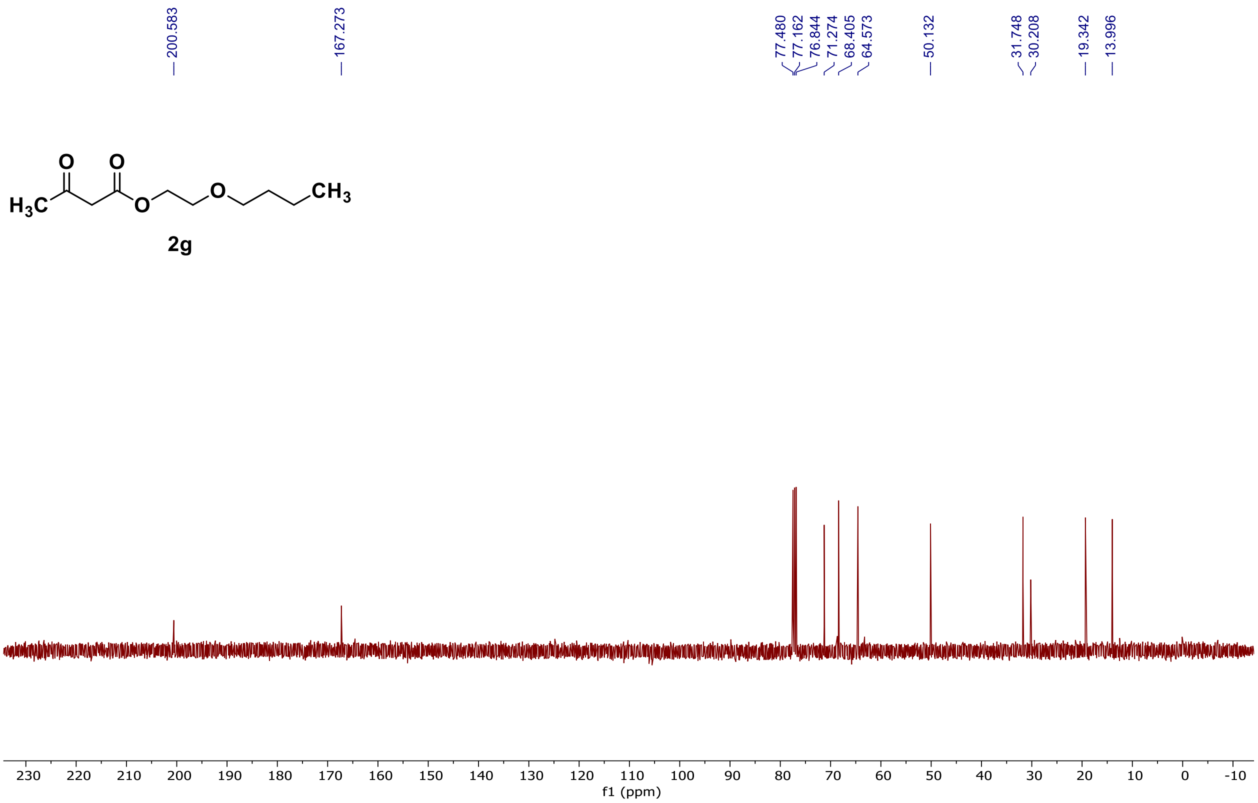

^{1}H and ^{13}C NMR Spectrum of **2g** in $CDCl_3$

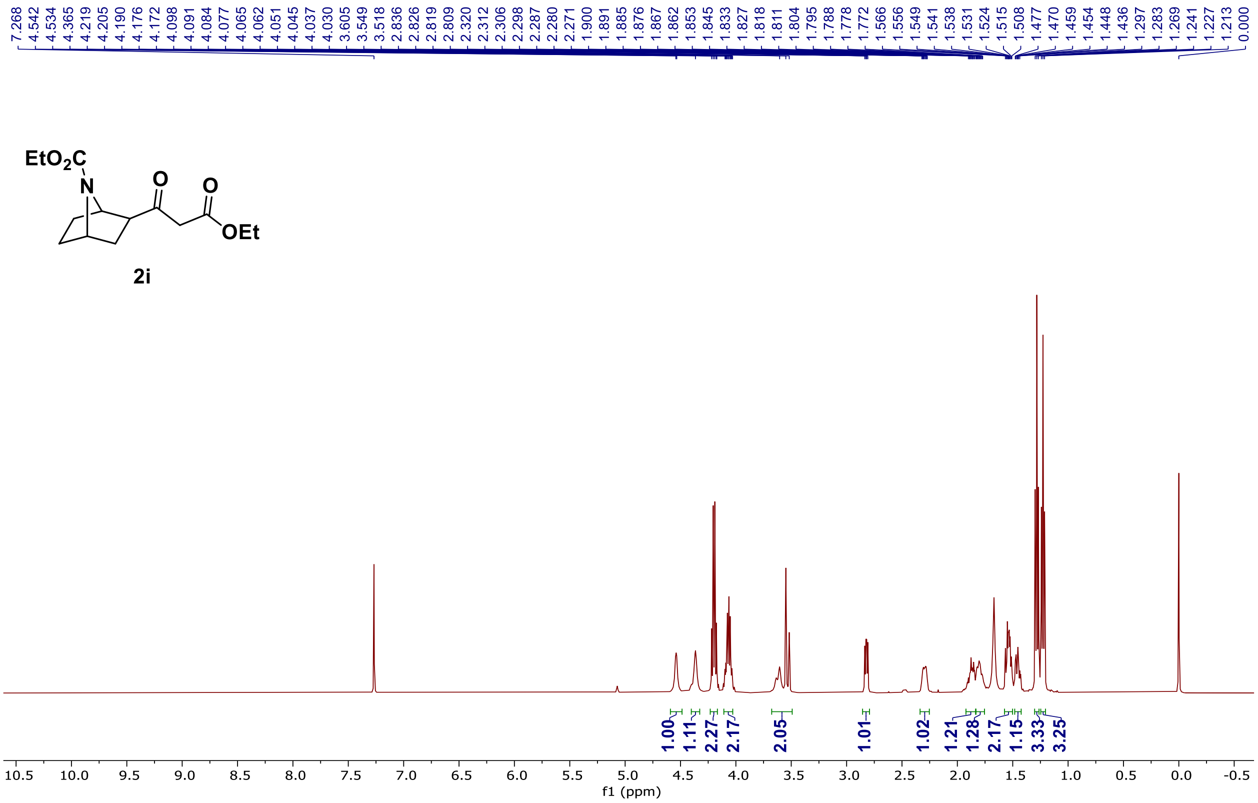

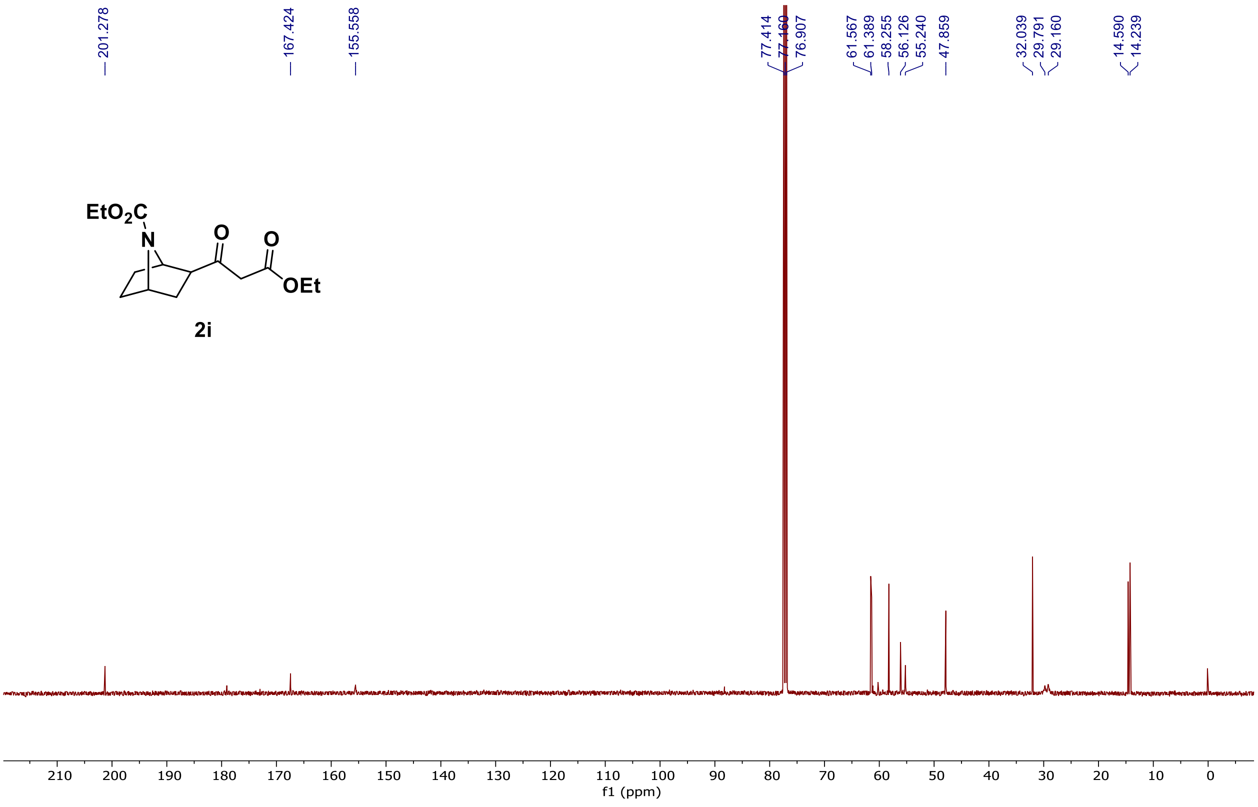

^{1}H and ^{13}C NMR Spectrum of **2i** in $CDCl_3$

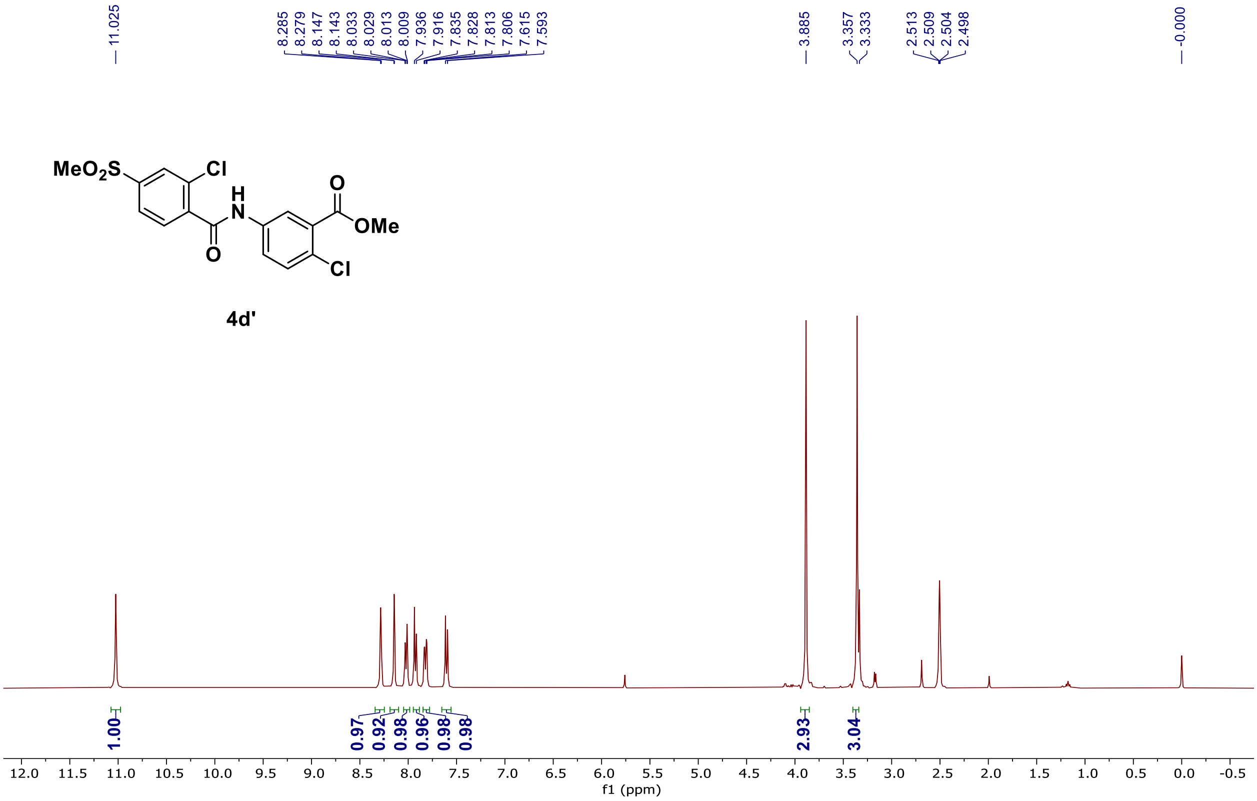

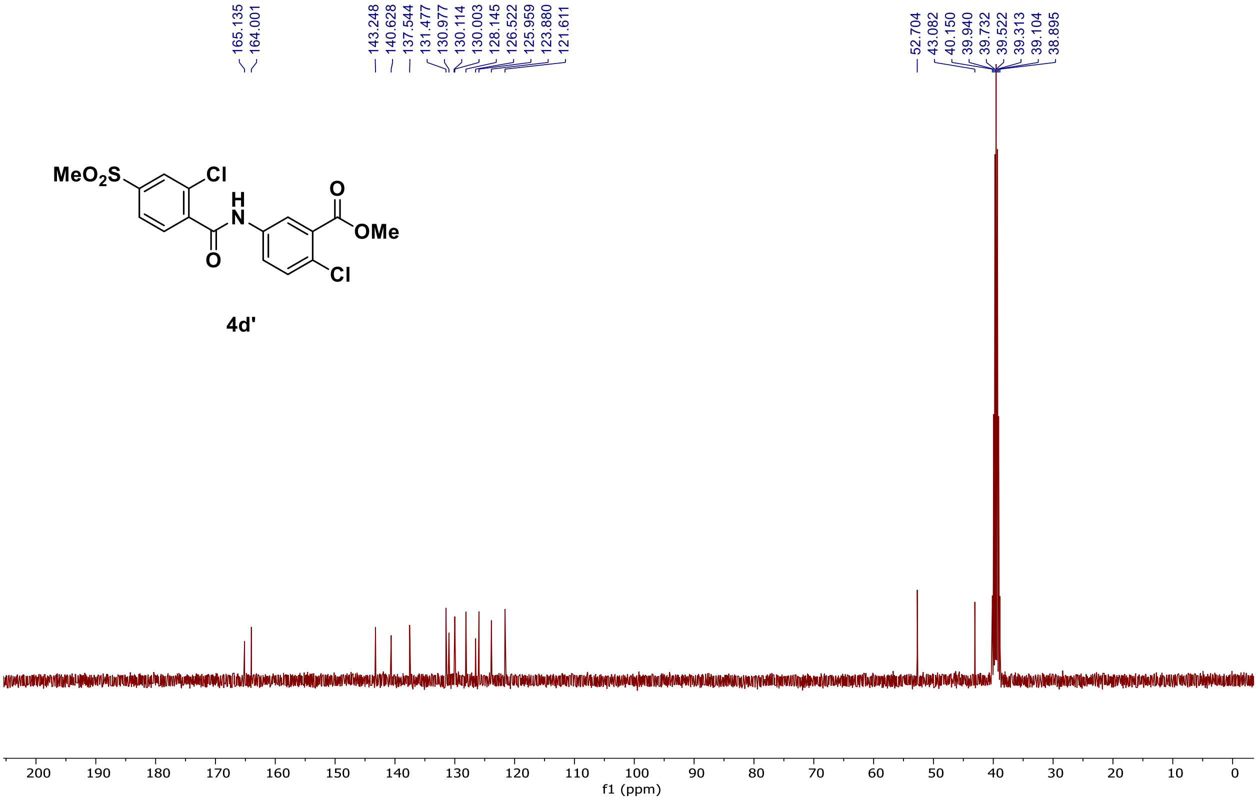

^{1}H and ^{13}C NMR Spectrum of **4d'** in DMSO-d_6

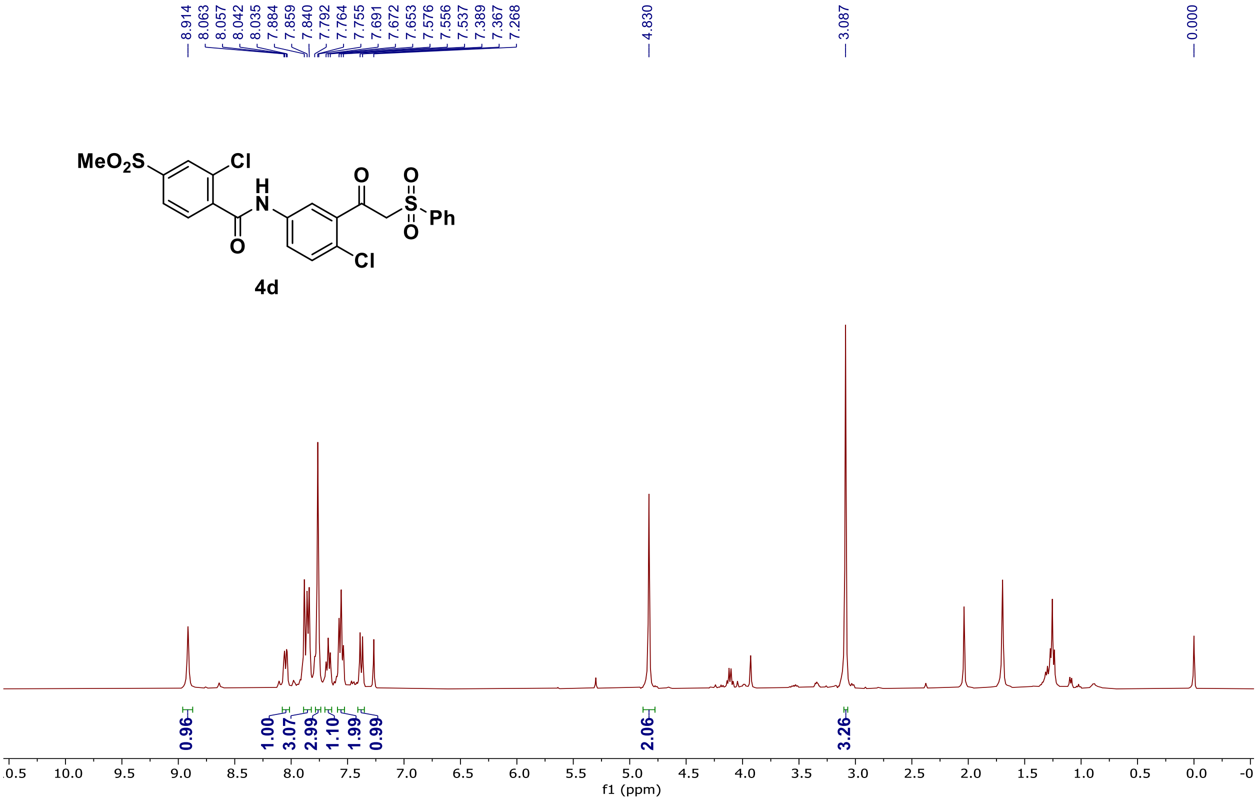

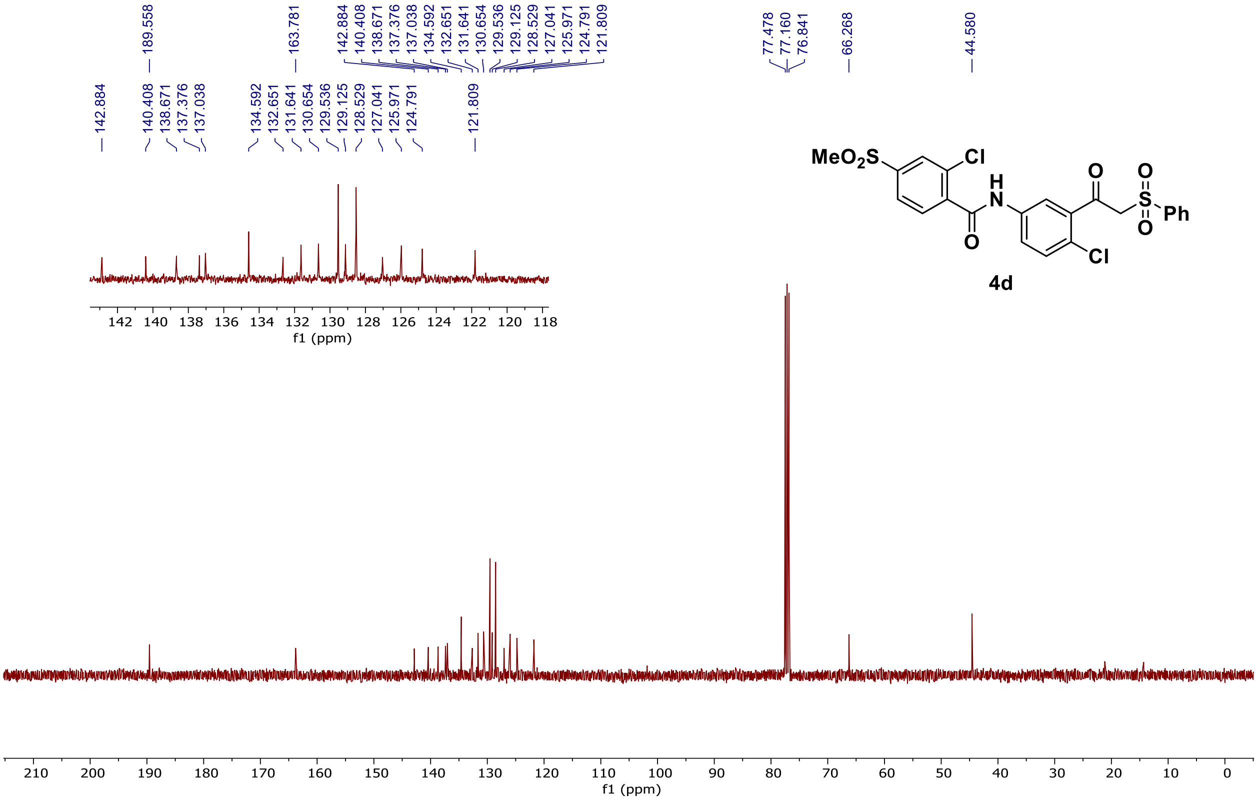

^{1}H and ^{13}C NMR Spectrum of **4d** in $CDCl_3$

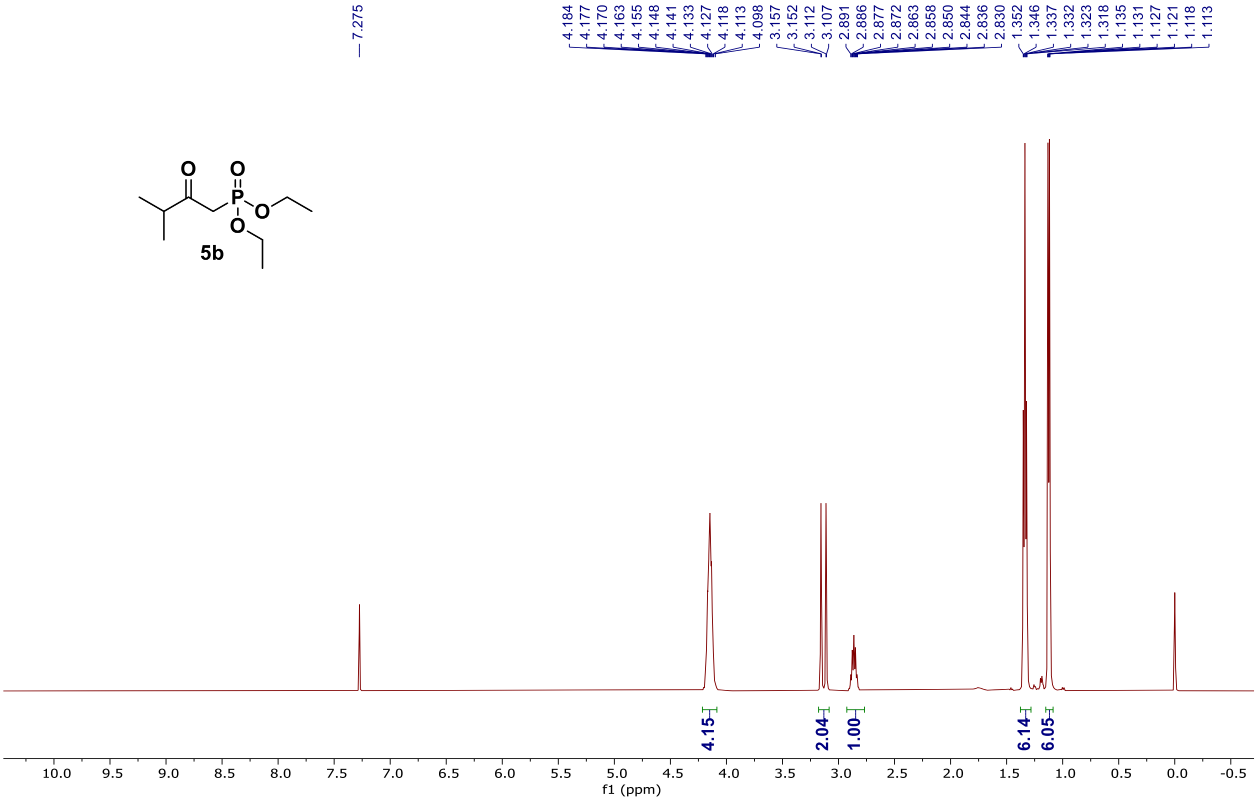

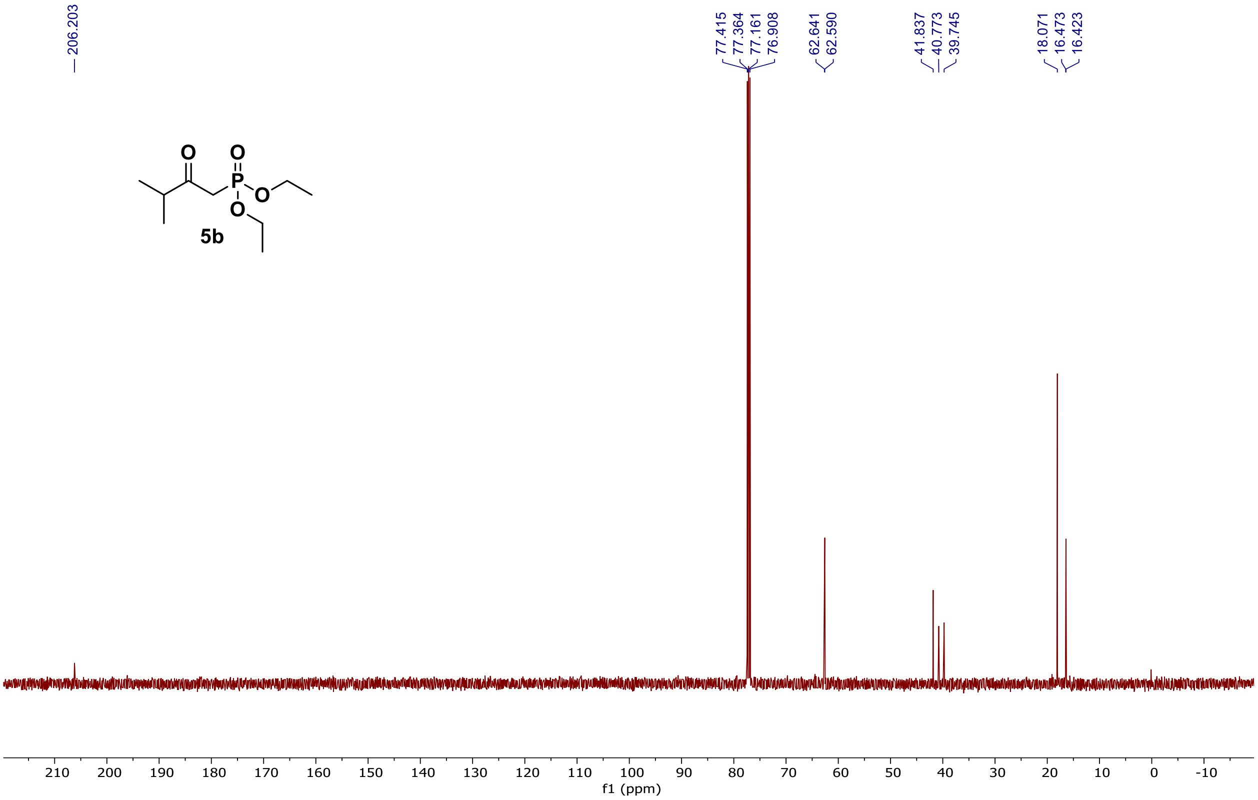

^{1}H and ^{13}C NMR Spectrum of **5b** in $CDCl_3$

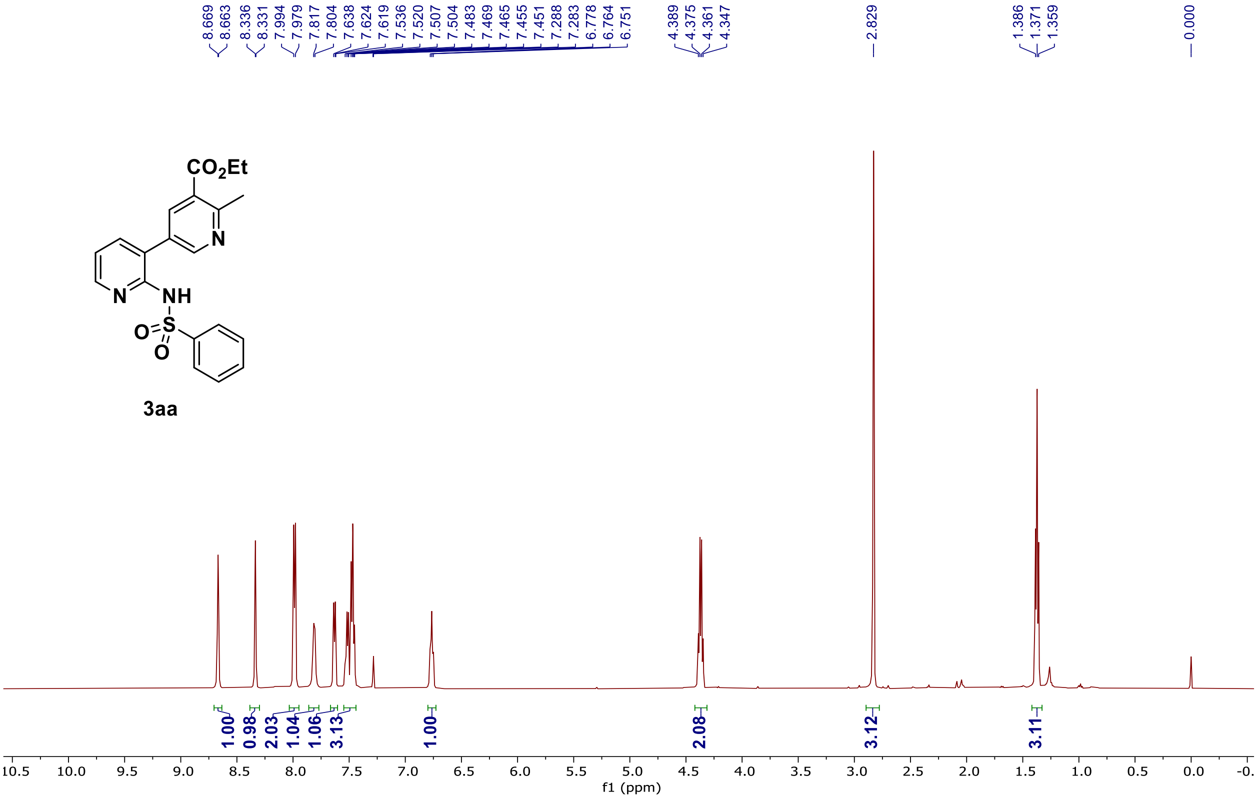

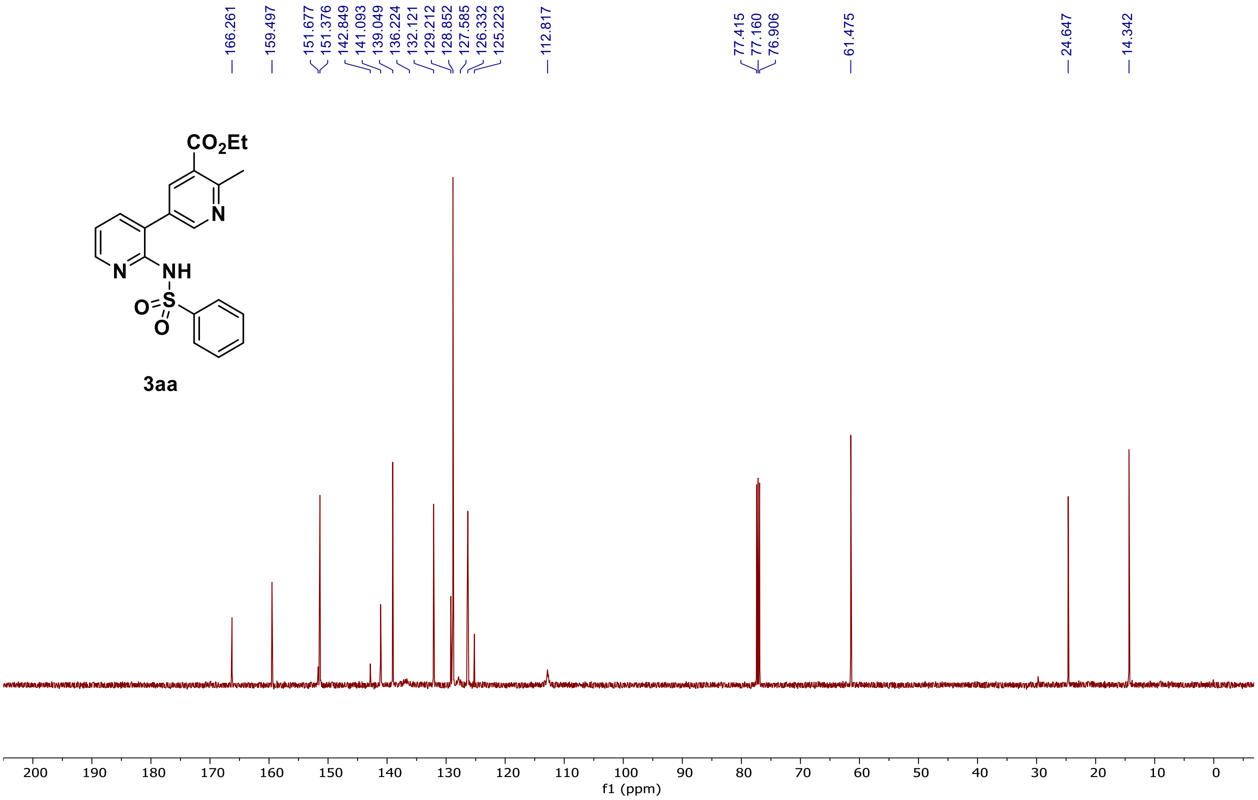

^{1}H and ^{13}C NMR Spectrum of **3aa** in $CDCl_3$

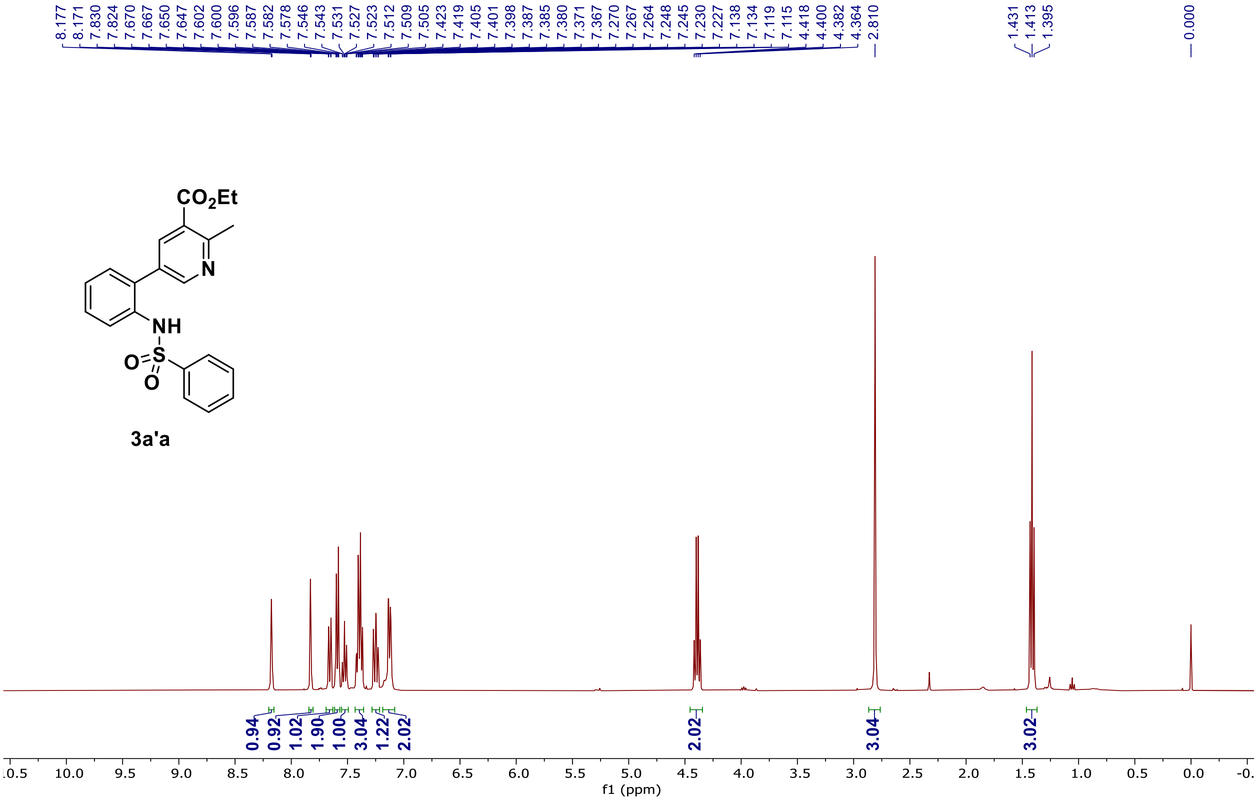

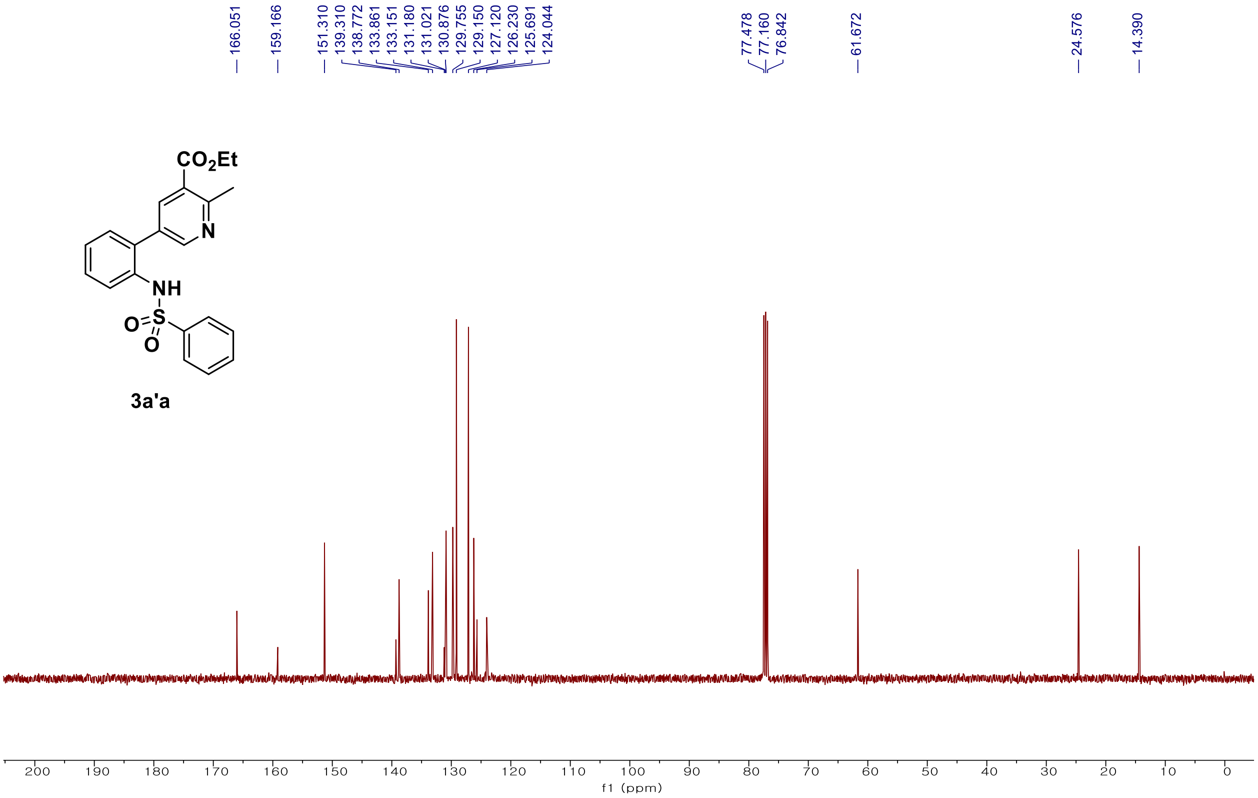

^{1}H and ^{13}C NMR Spectrum of **3a'a** in $CDCl_3$

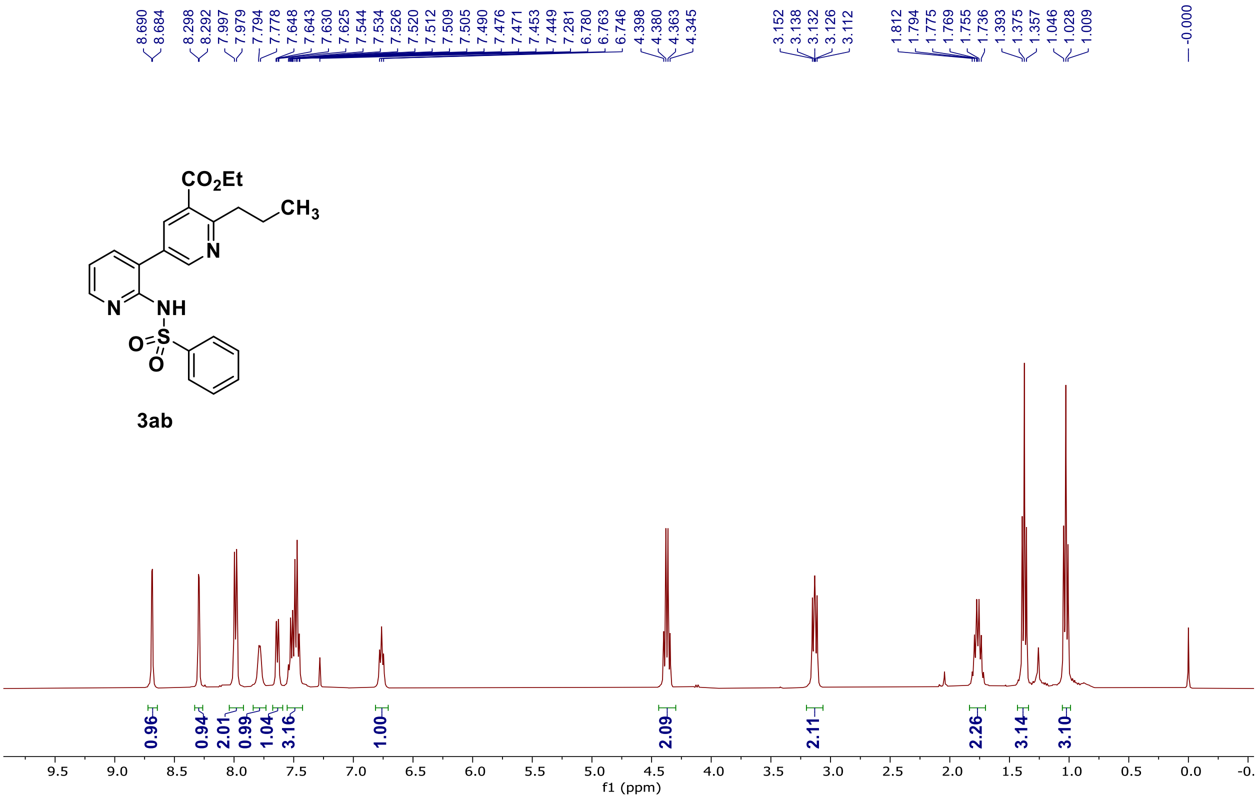

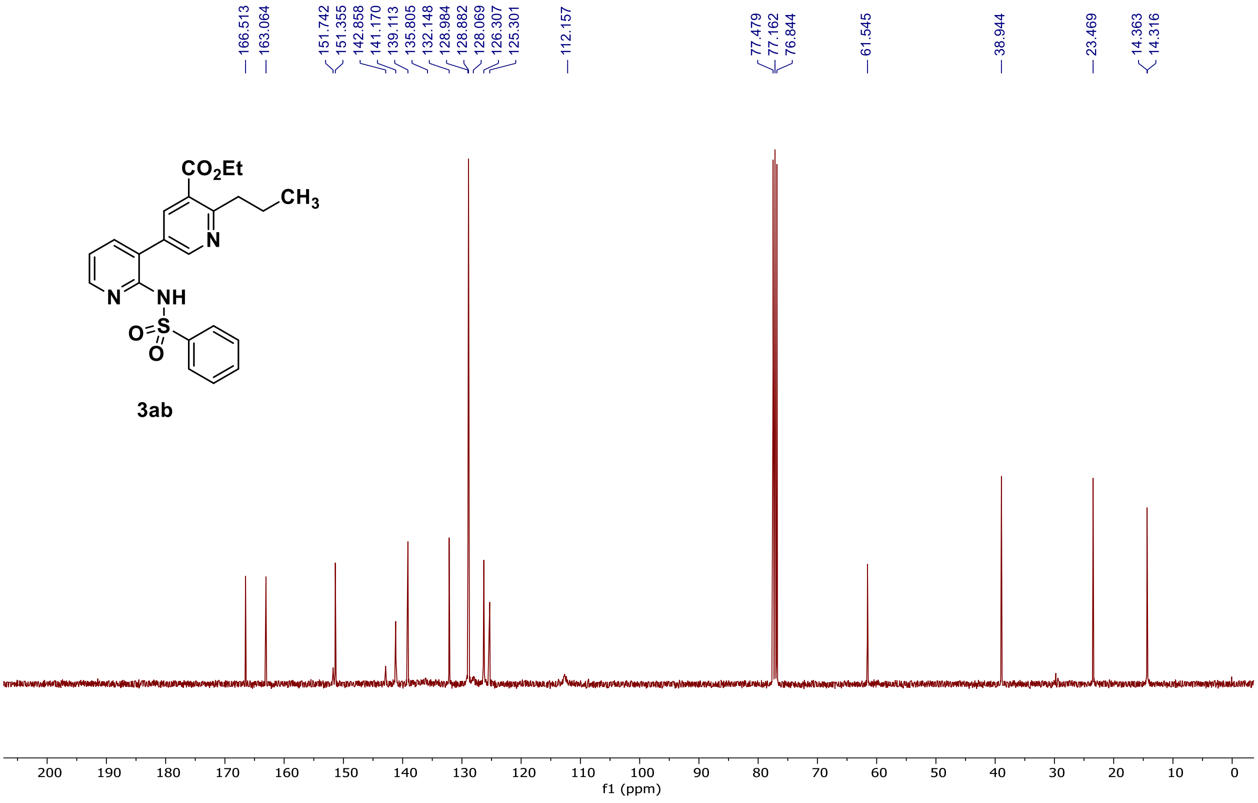

^{1}H and ^{13}C NMR Spectrum of **3ab** in $CDCl_3$

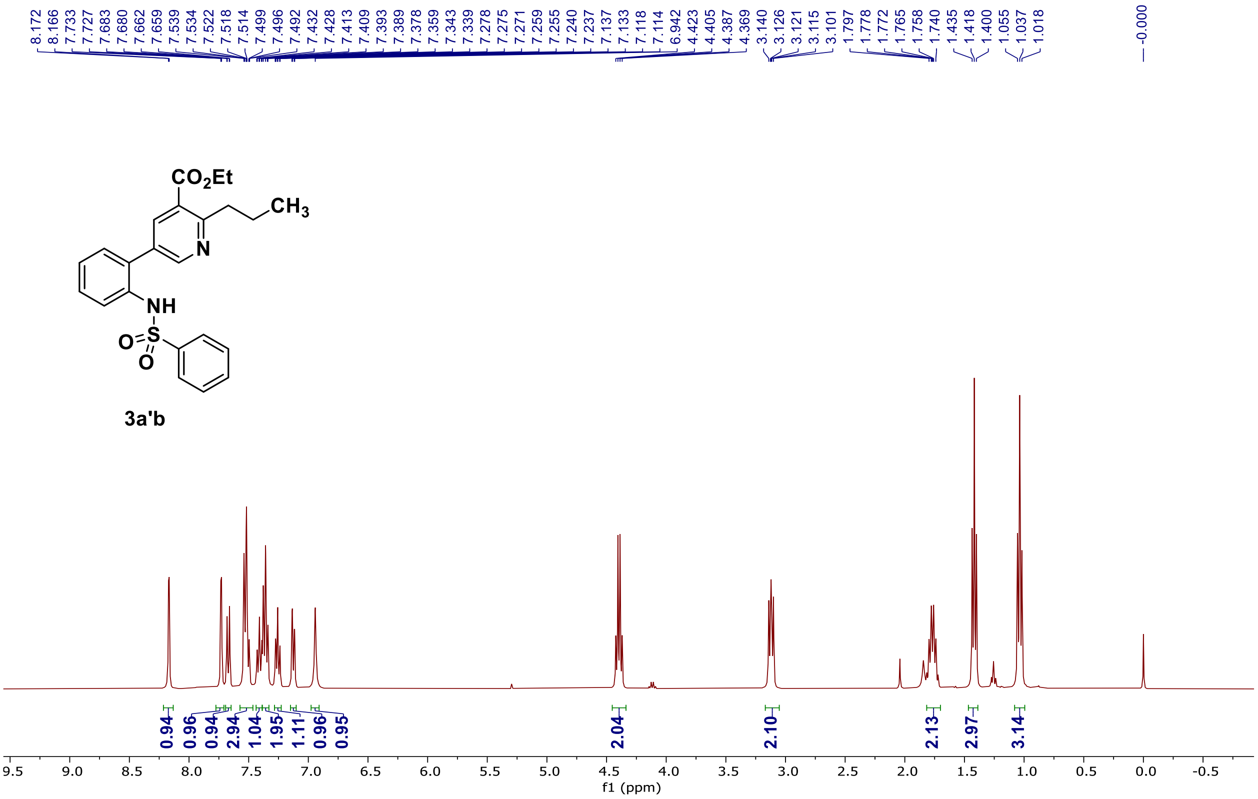

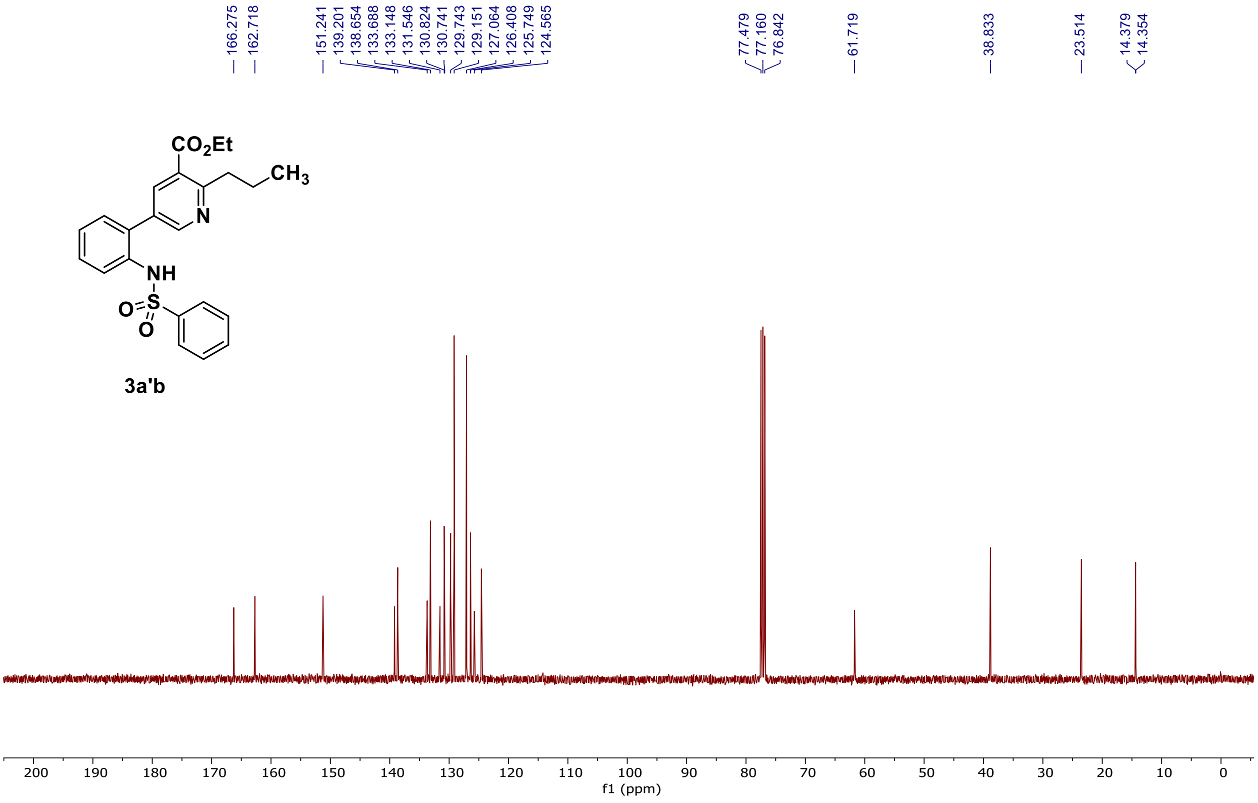

^{1}H and ^{13}C NMR Spectrum of **3a'b** in $CDCl_3$

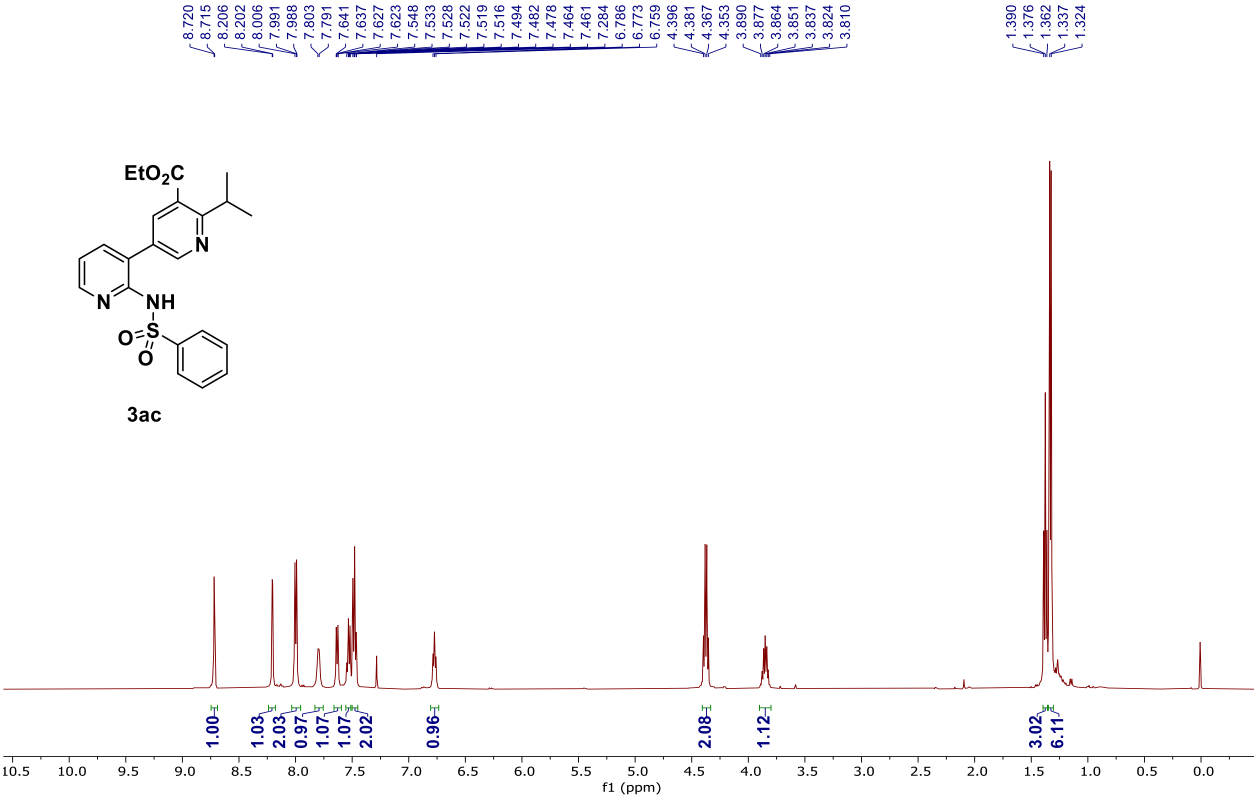

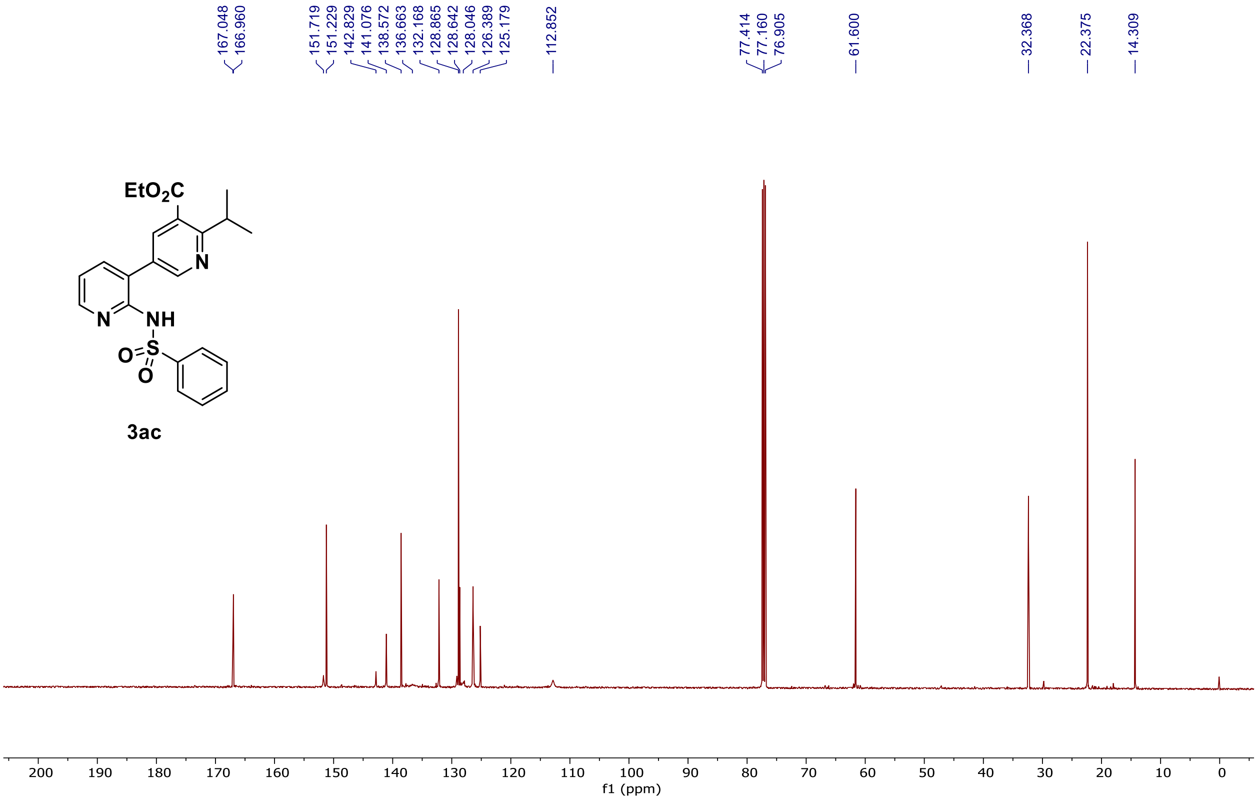

^{1}H and ^{13}C NMR Spectrum of **3ac** in $CDCl_3$

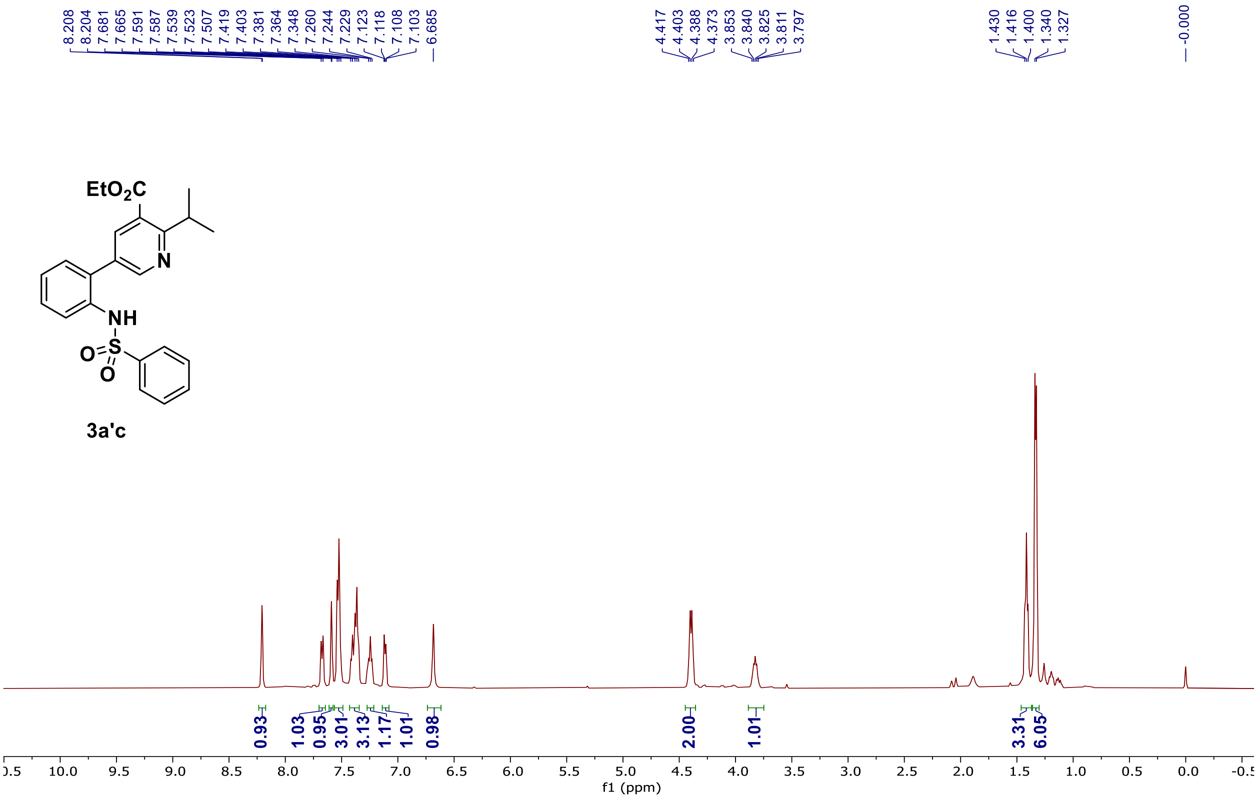

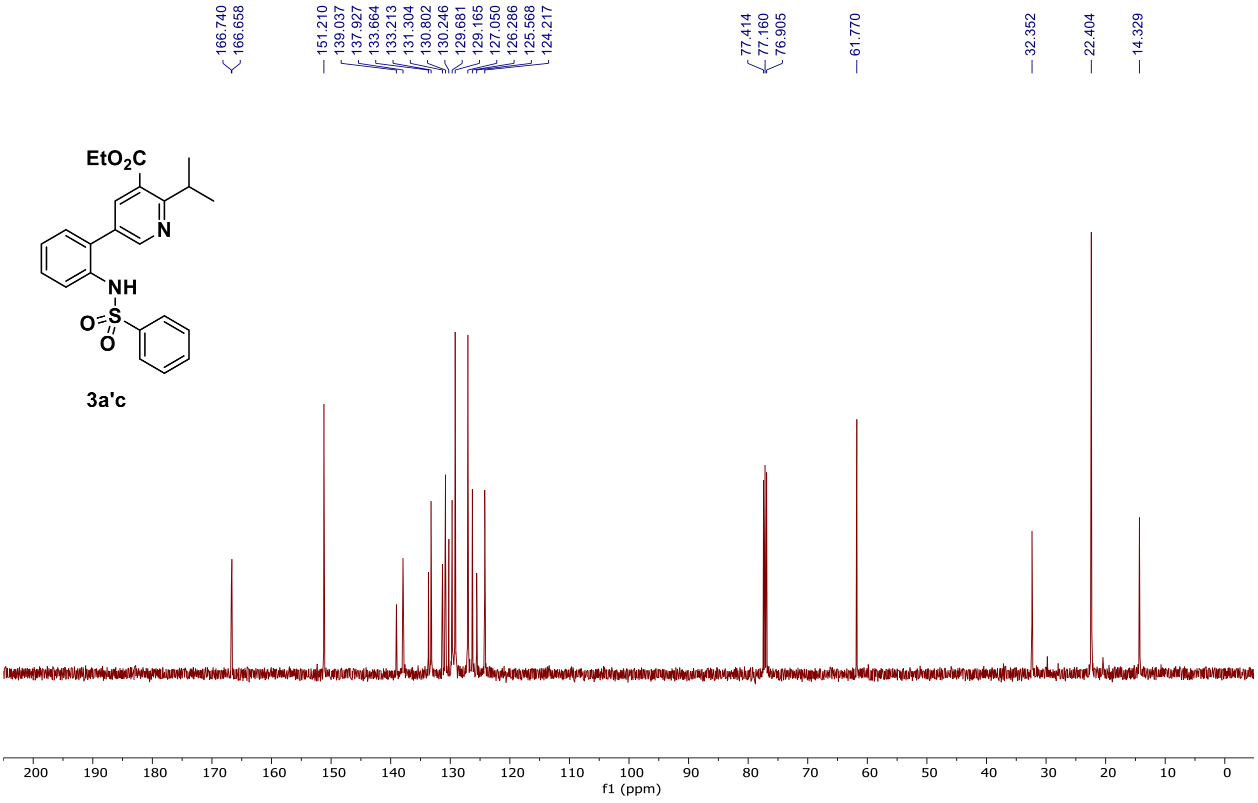

^{1}H and ^{13}C NMR Spectrum of **3a'c** in $CDCl_3$

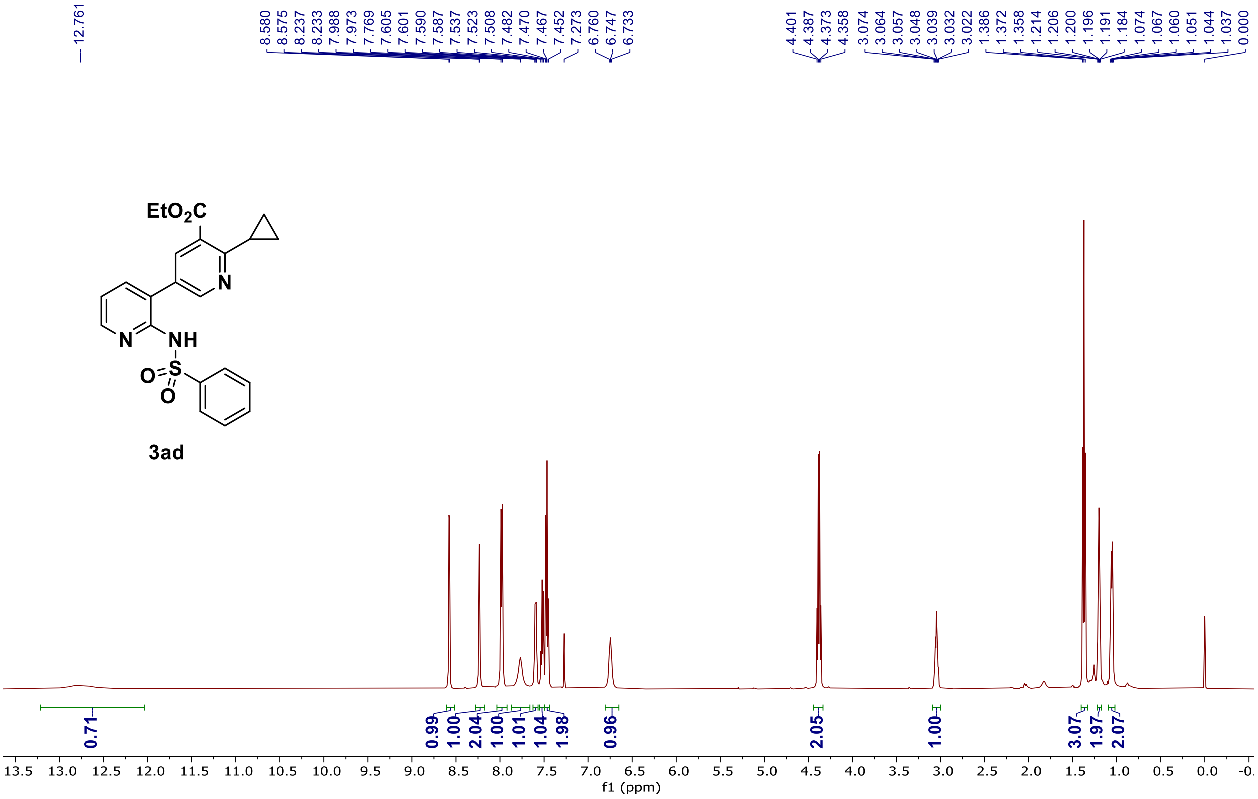

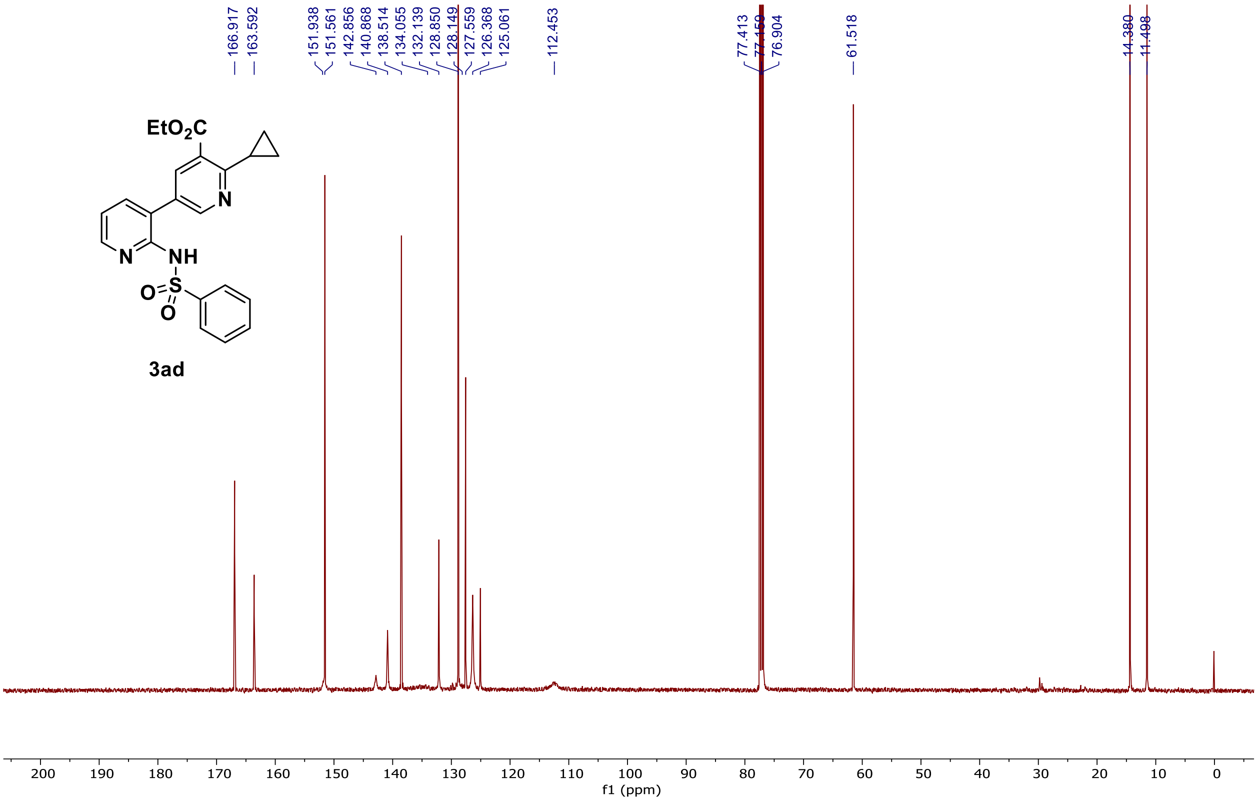

^{1}H and ^{13}C NMR Spectrum of **3ad** in $CDCl_3$

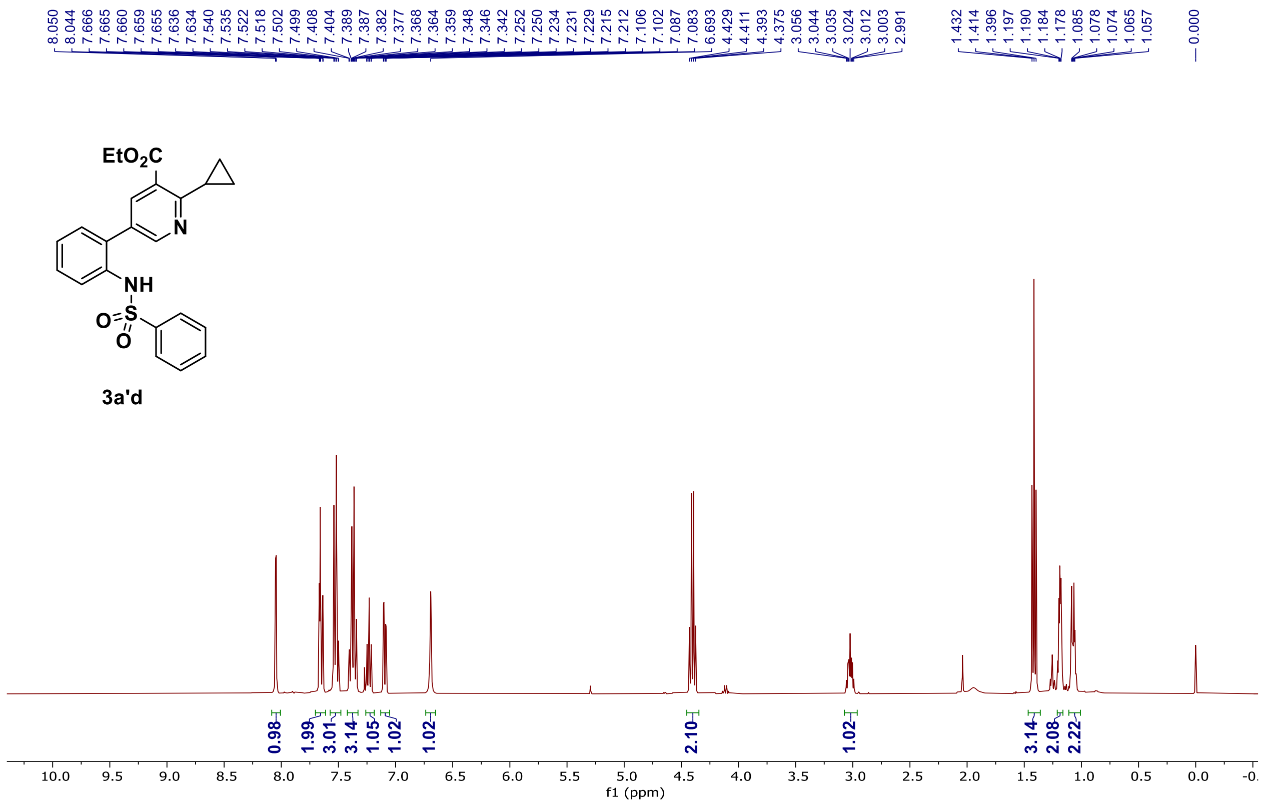

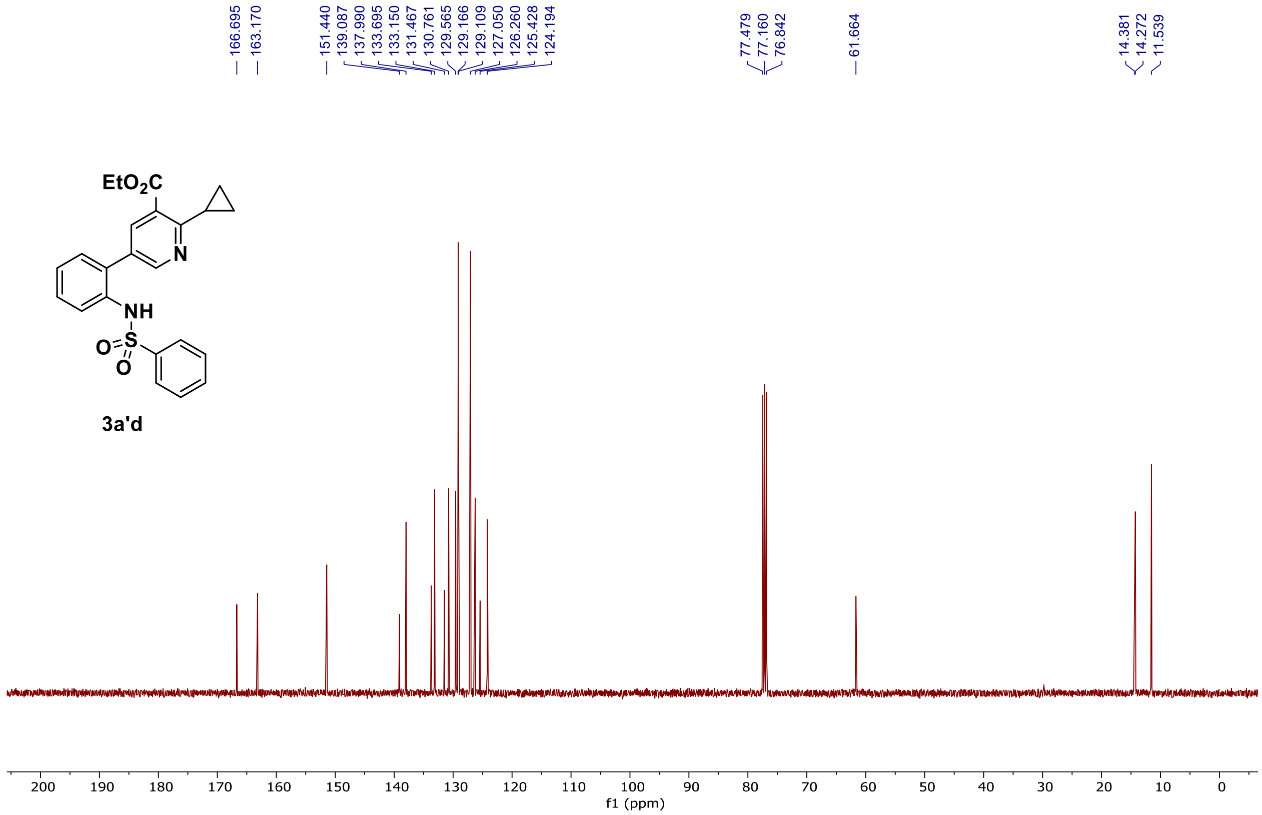

^{1}H and ^{13}C NMR Spectrum of **3a'd** in $CDCl_3$

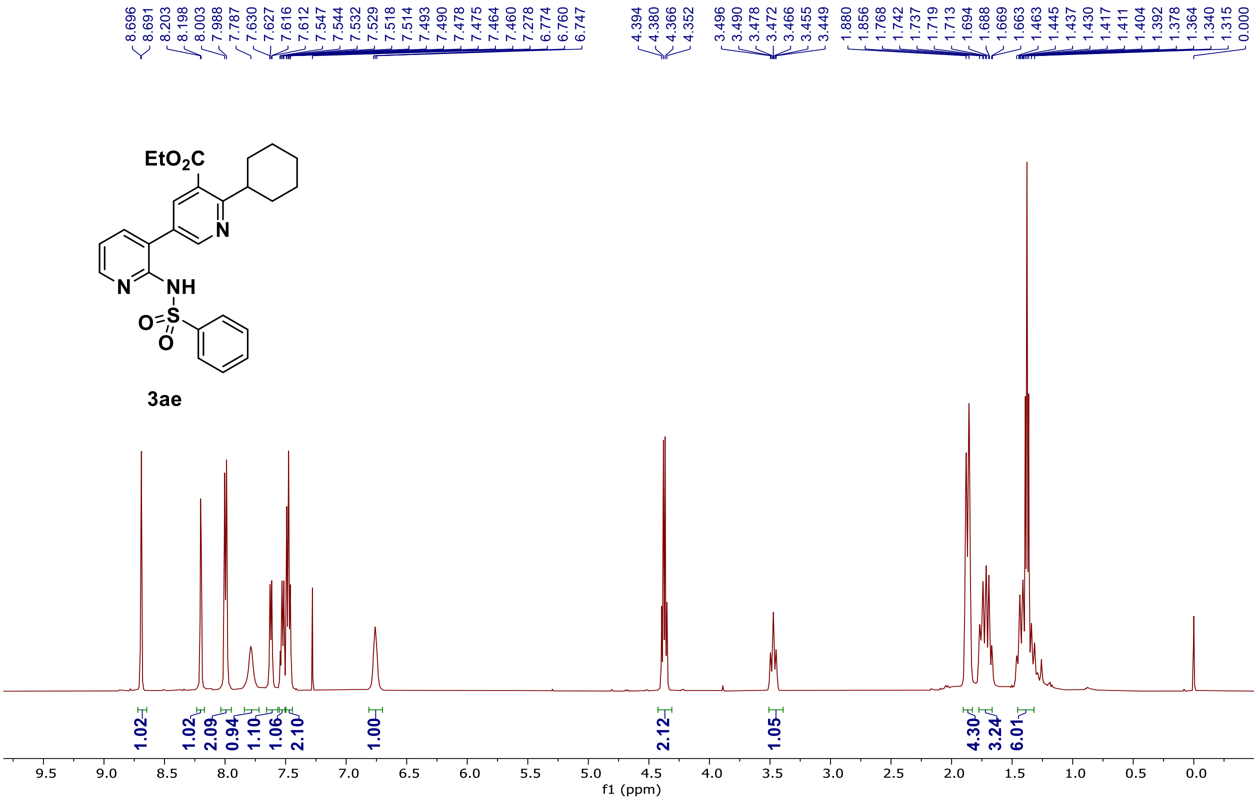

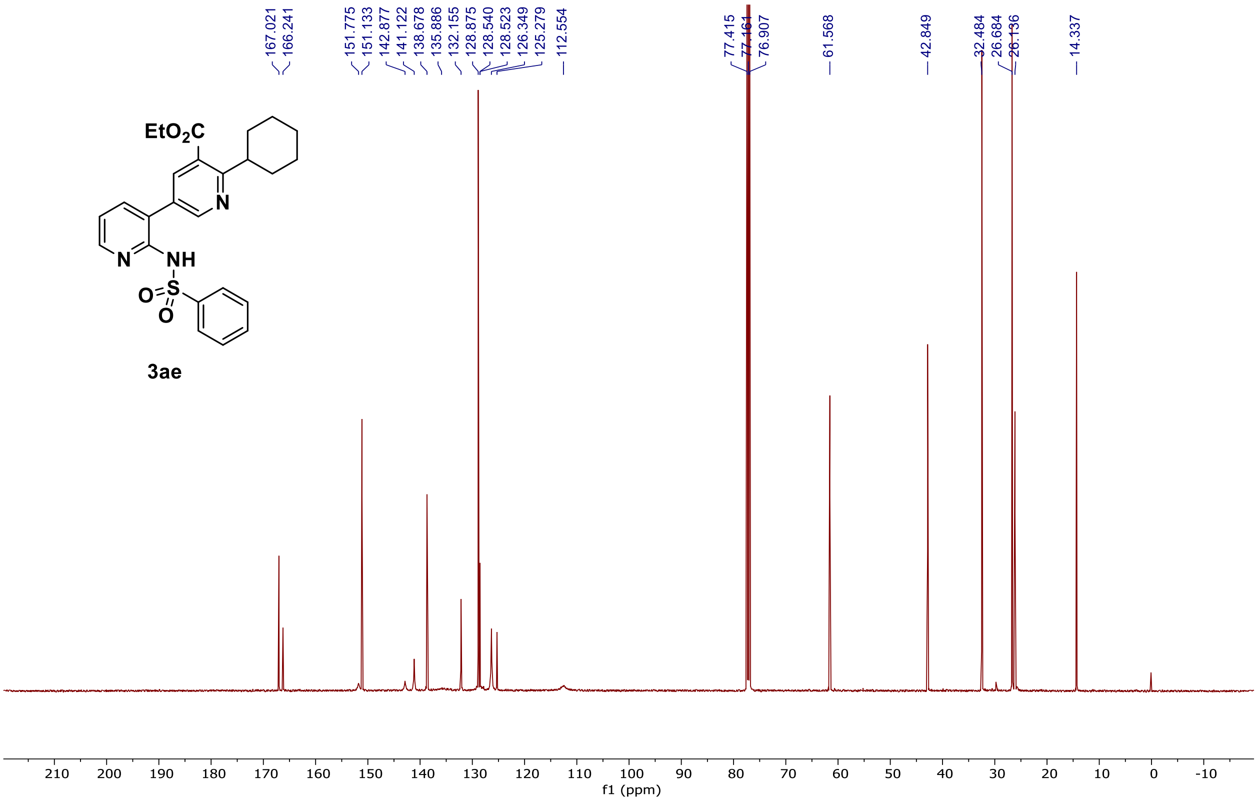

^{1}H and ^{13}C NMR Spectrum of **3ae** in $CDCl_3$

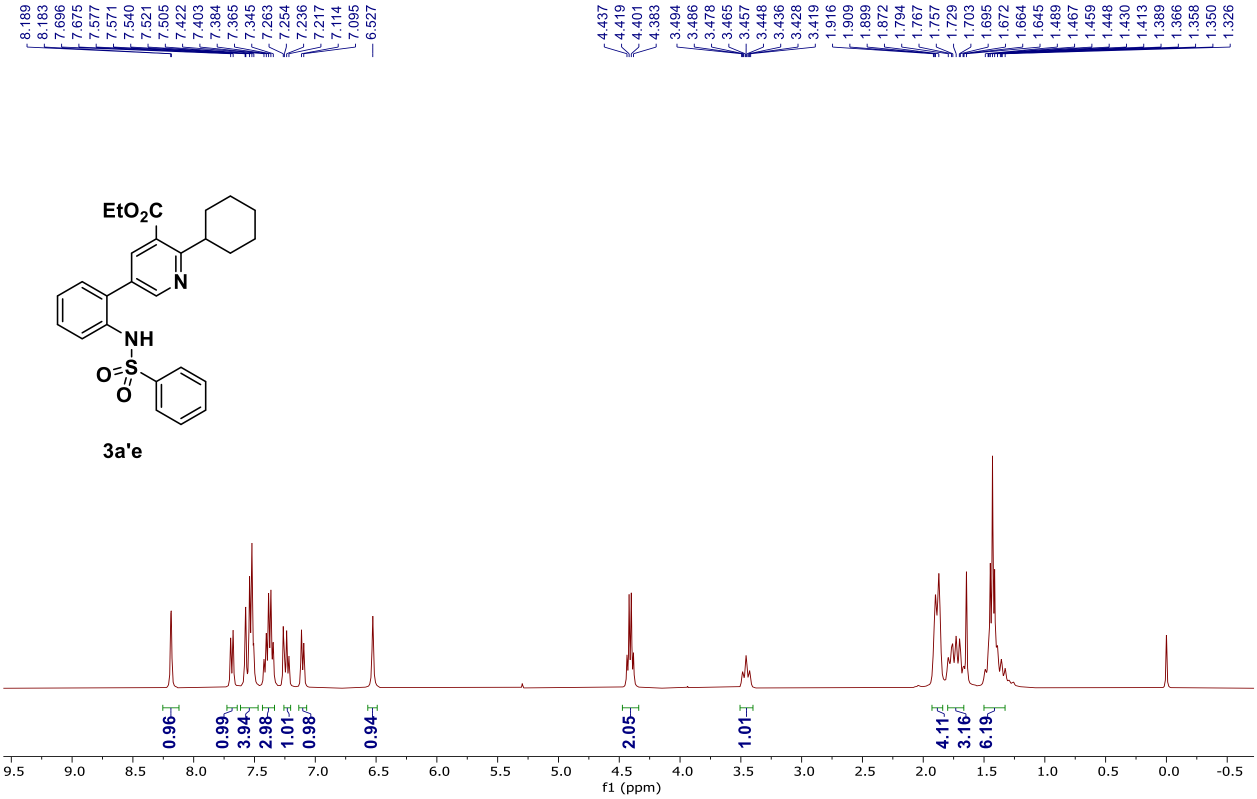

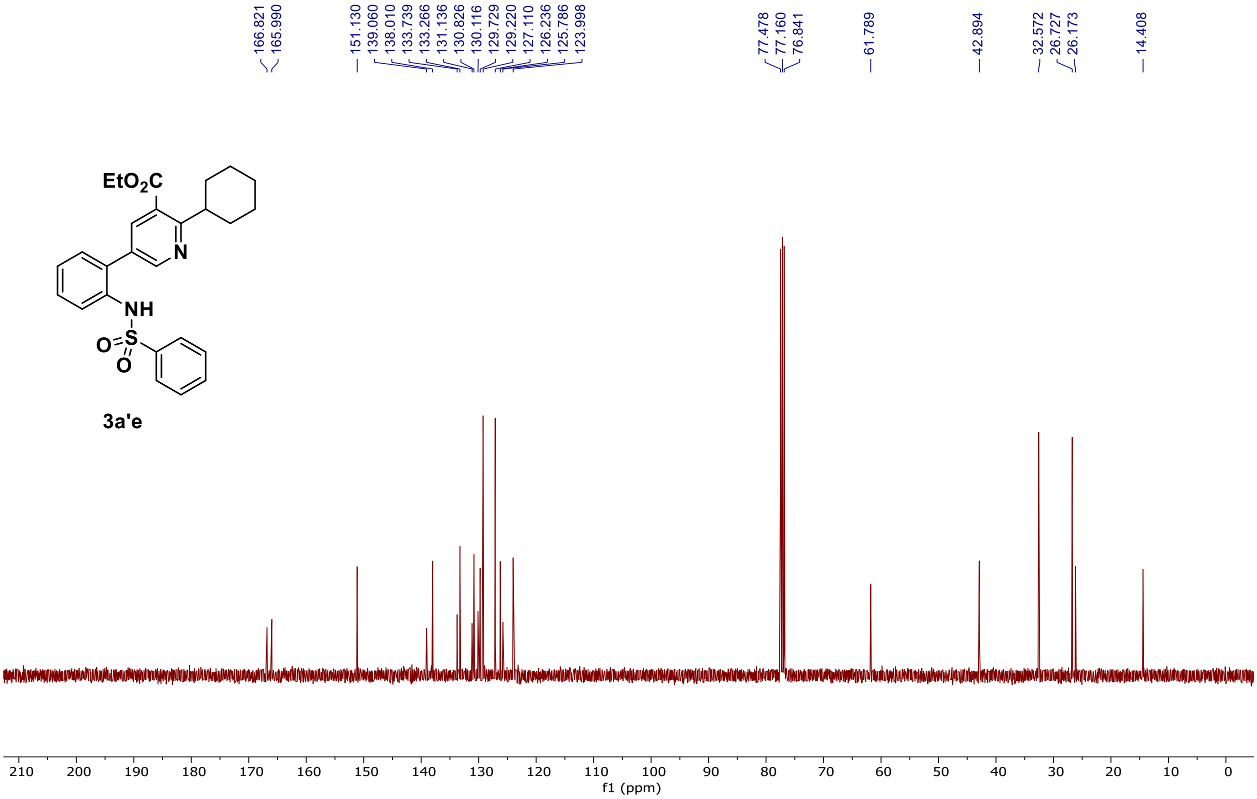

^{1}H and ^{13}C NMR Spectrum of **3a'e** in $CDCl_3$

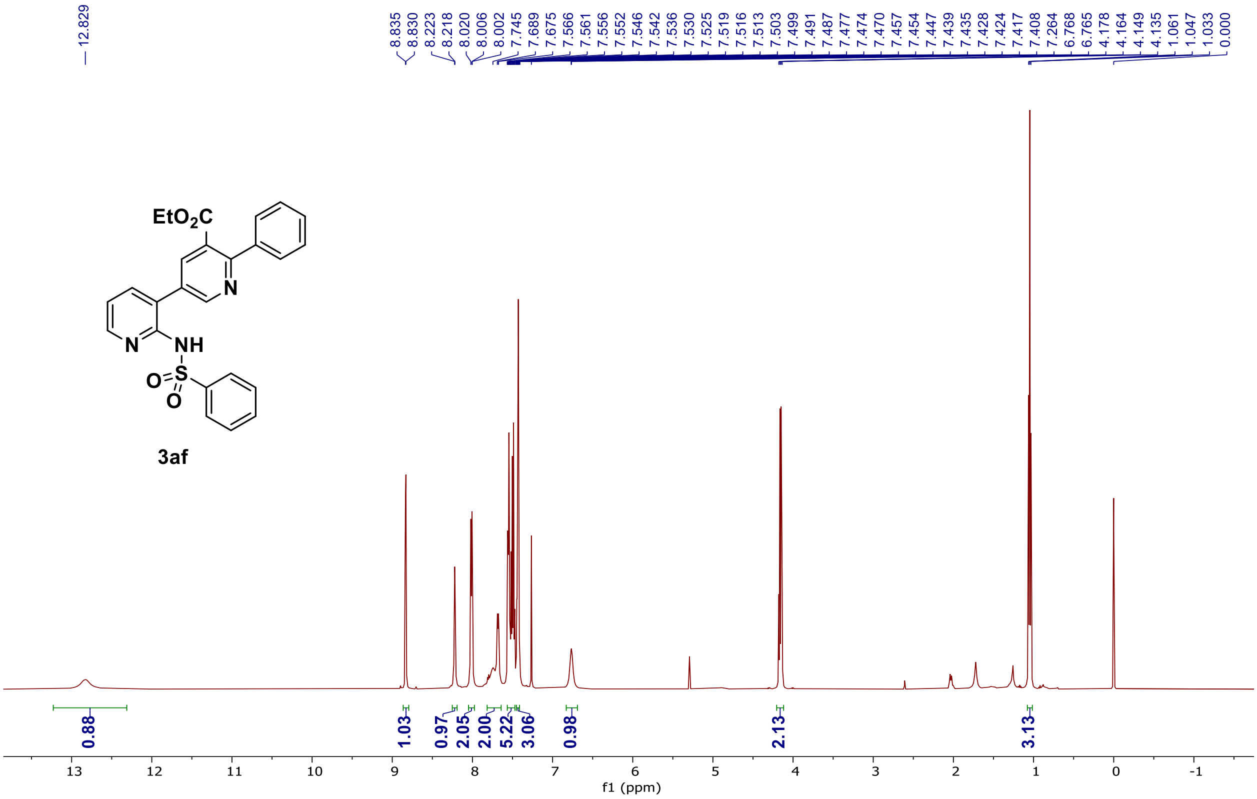

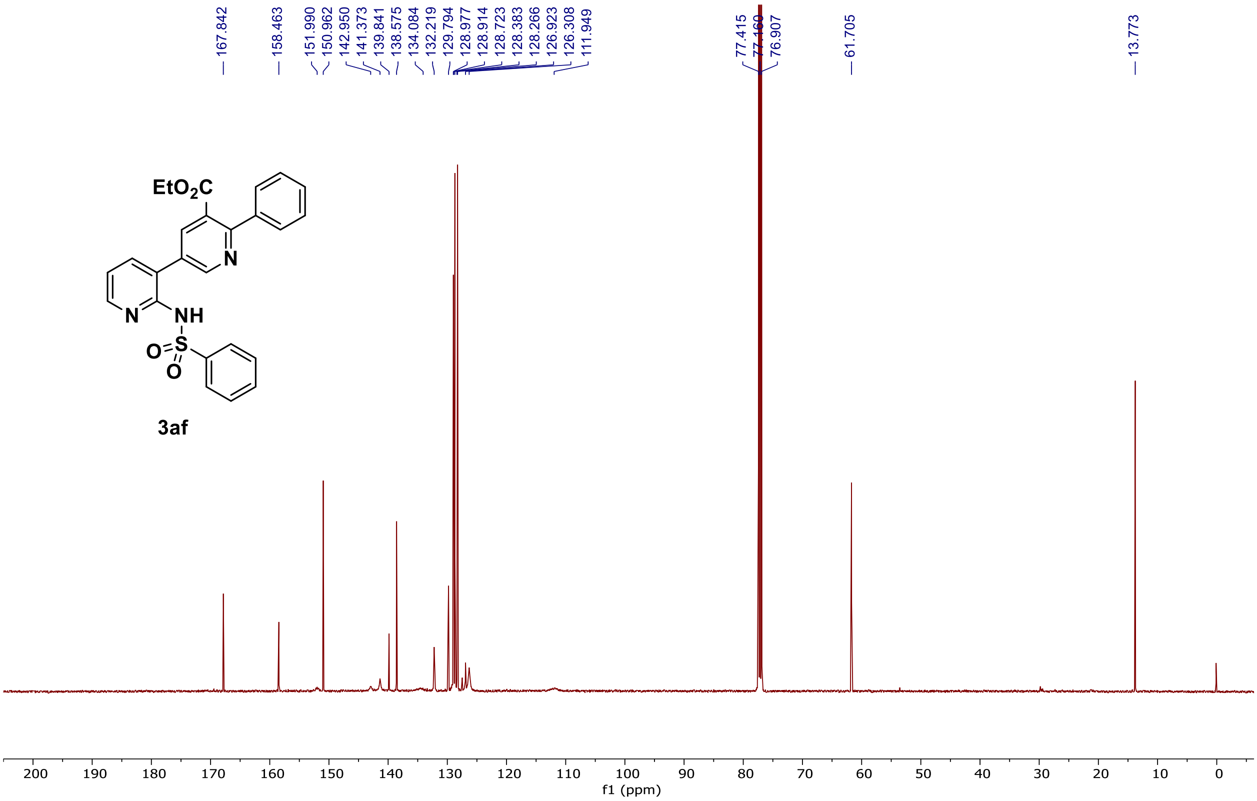

^{1}H and ^{13}C NMR Spectrum of **3af** in $CDCl_3$

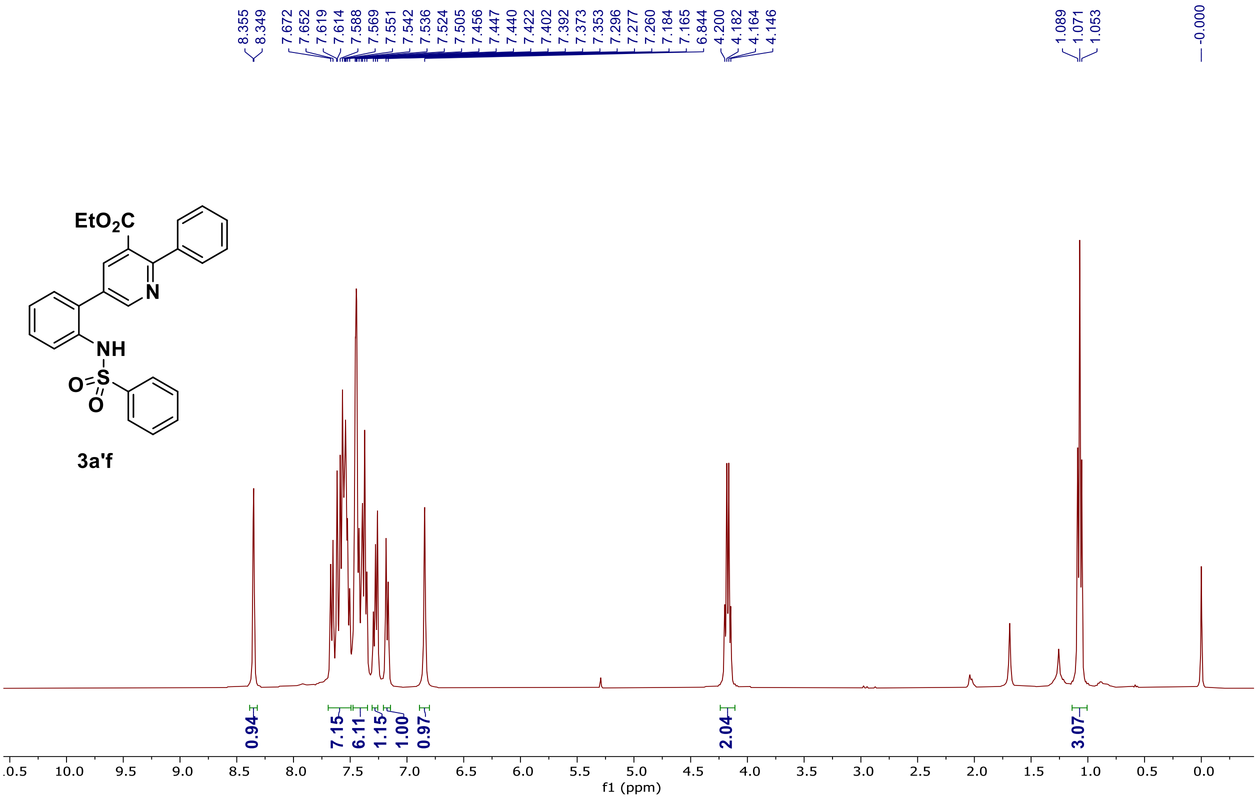

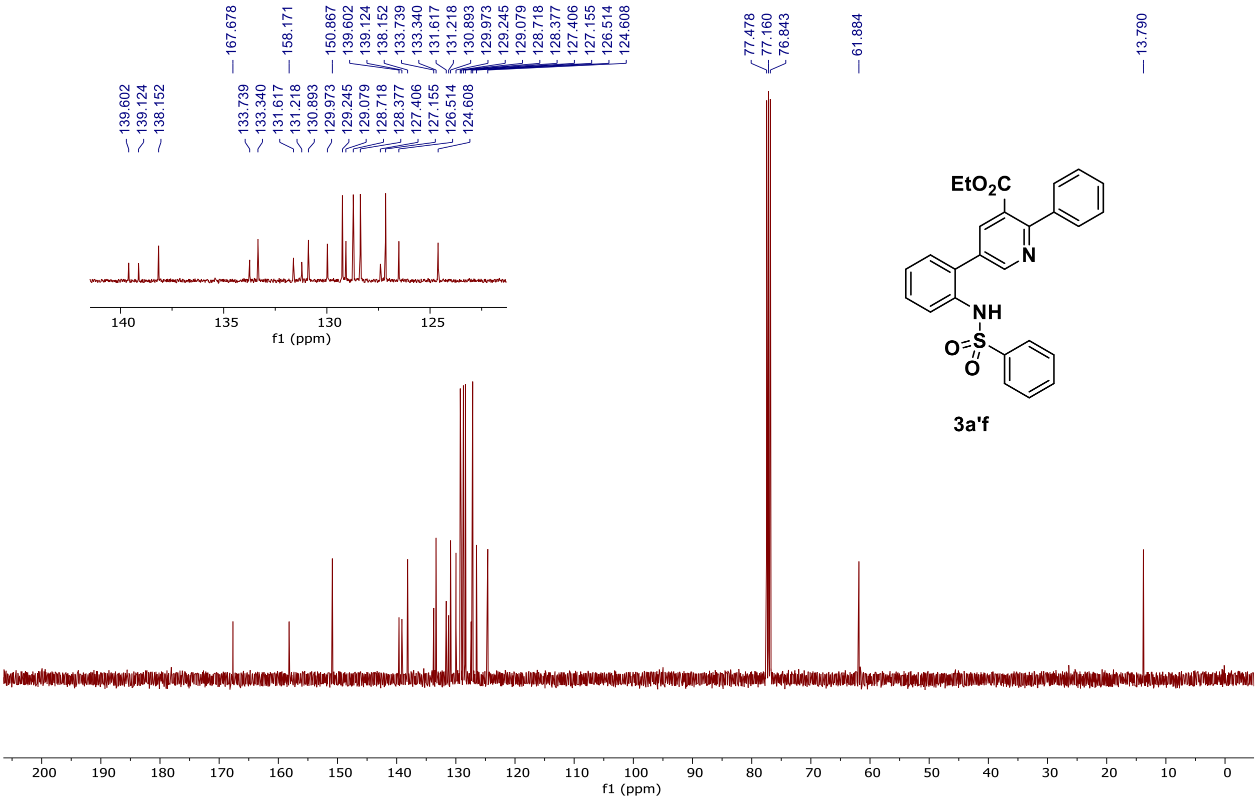

^{1}H and ^{13}C NMR Spectrum of **3a'f** in $CDCl_3$

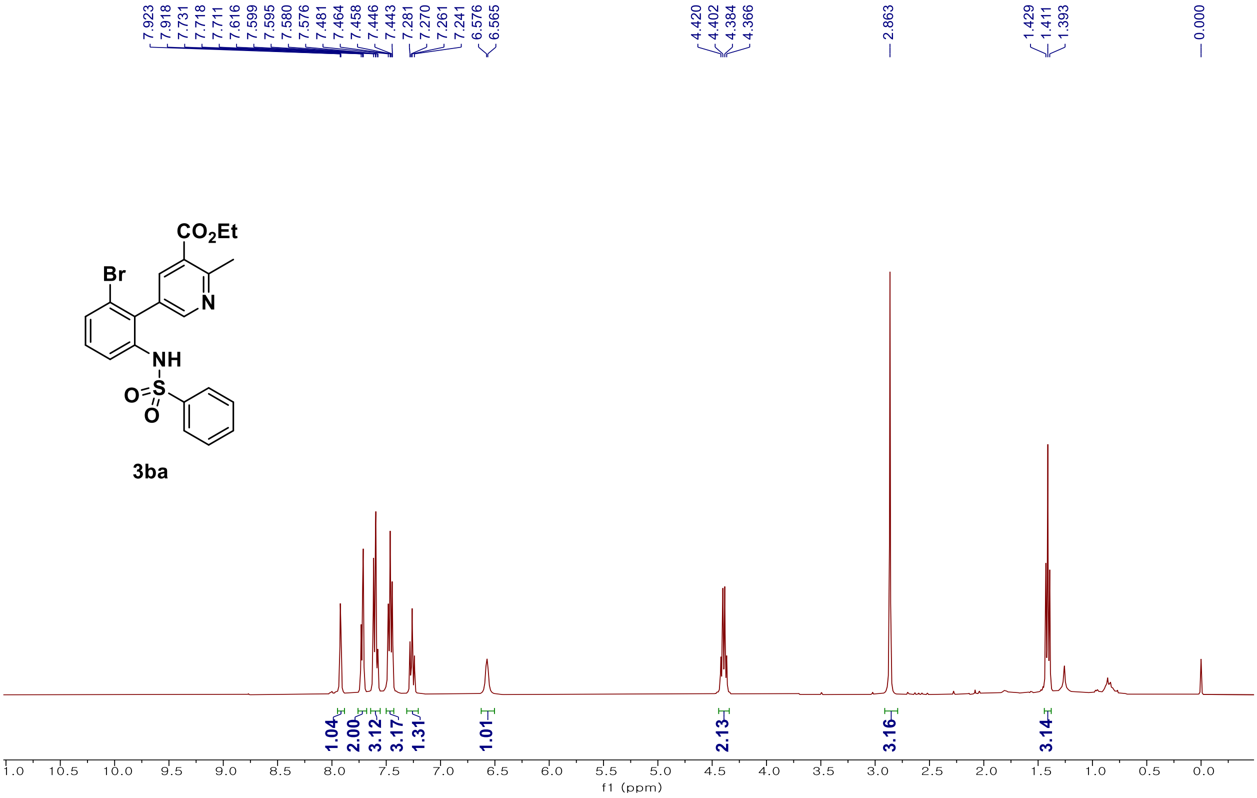

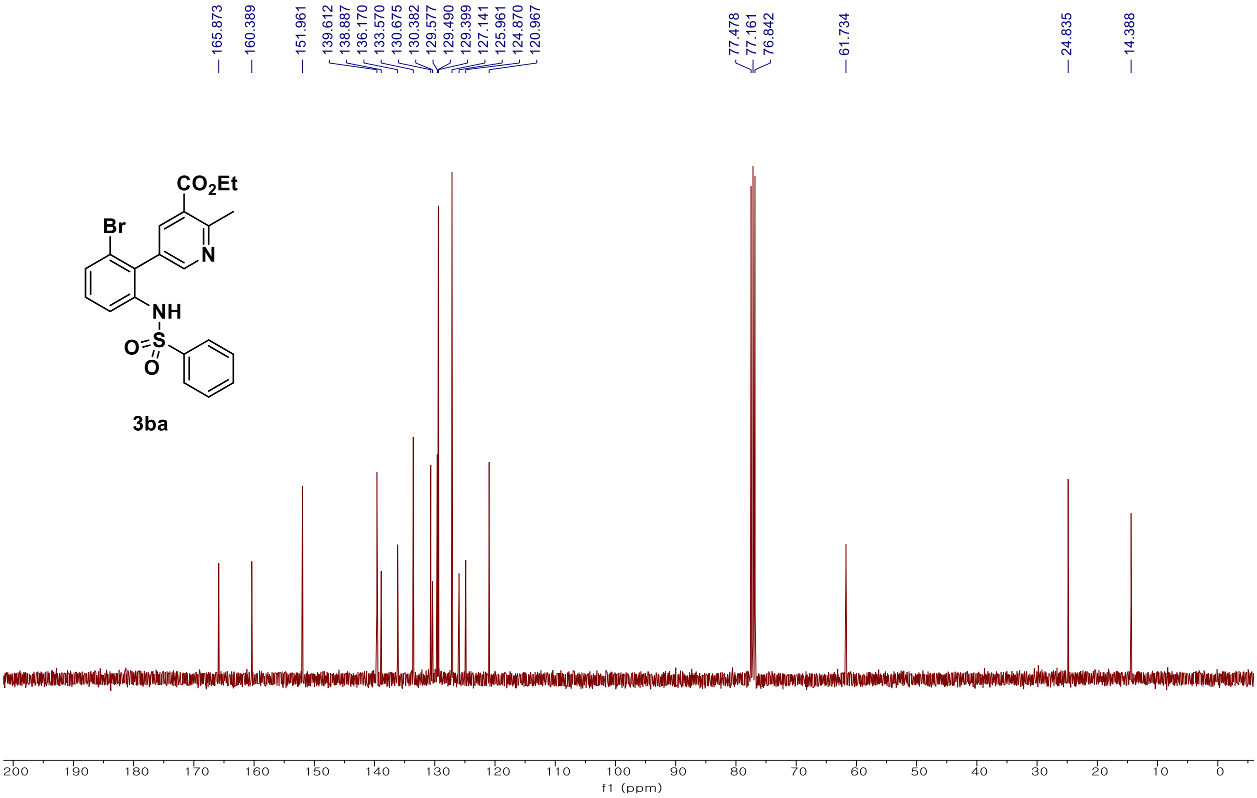

^{1}H and ^{13}C NMR Spectrum of **3ba** in $CDCl_3$

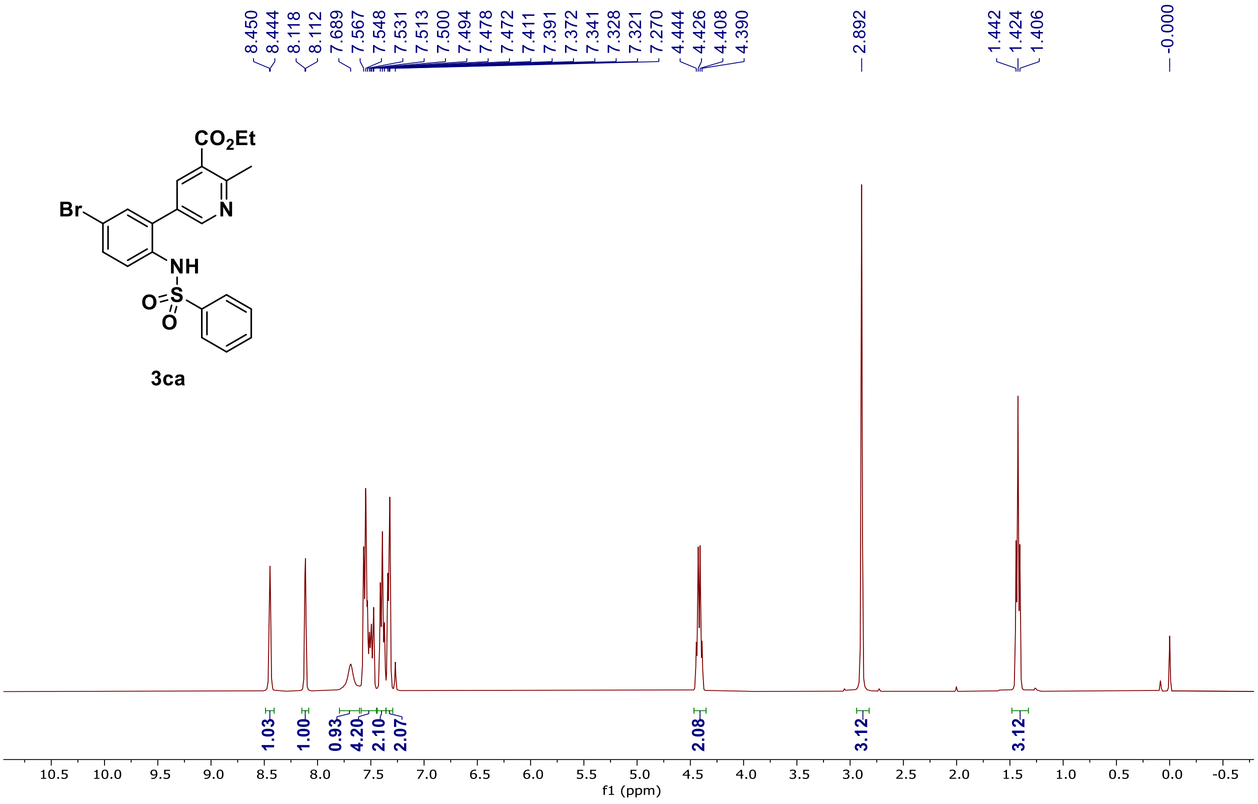

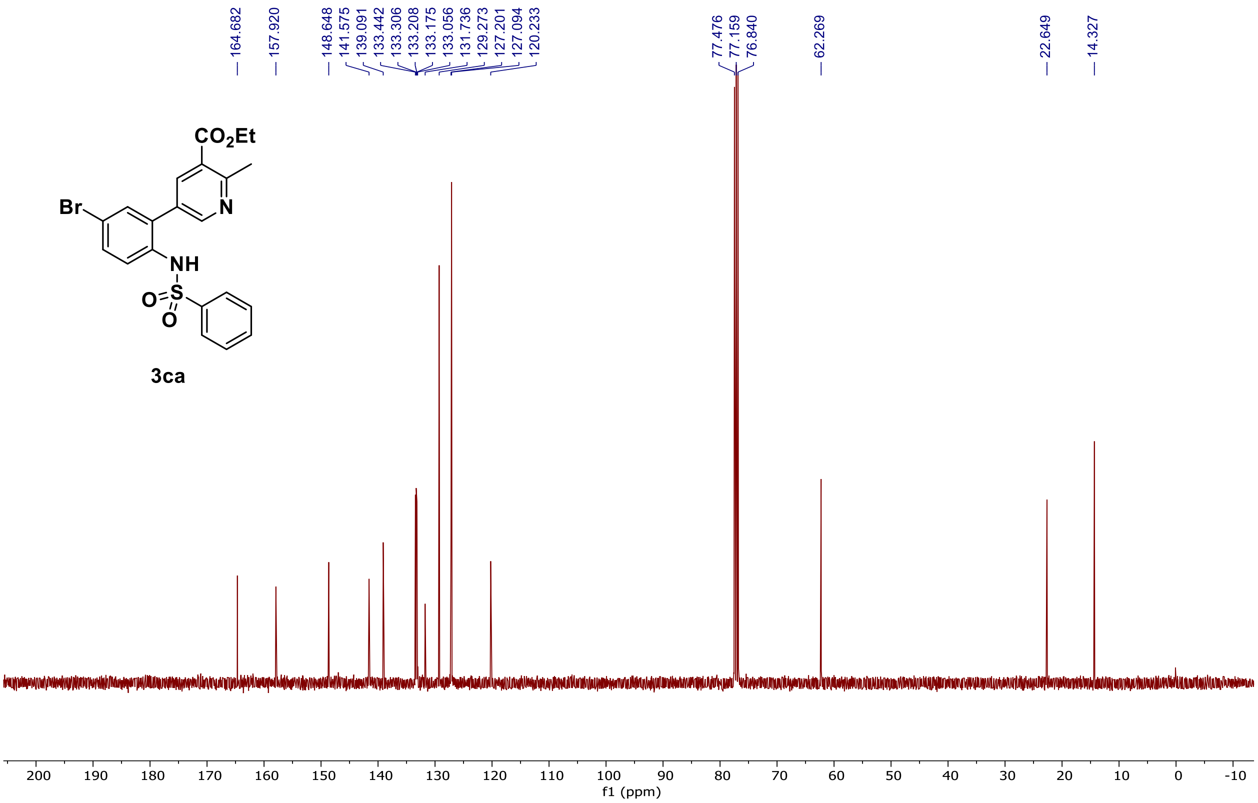

^{1}H and ^{13}C NMR Spectrum of **3ca** in $CDCl_3$

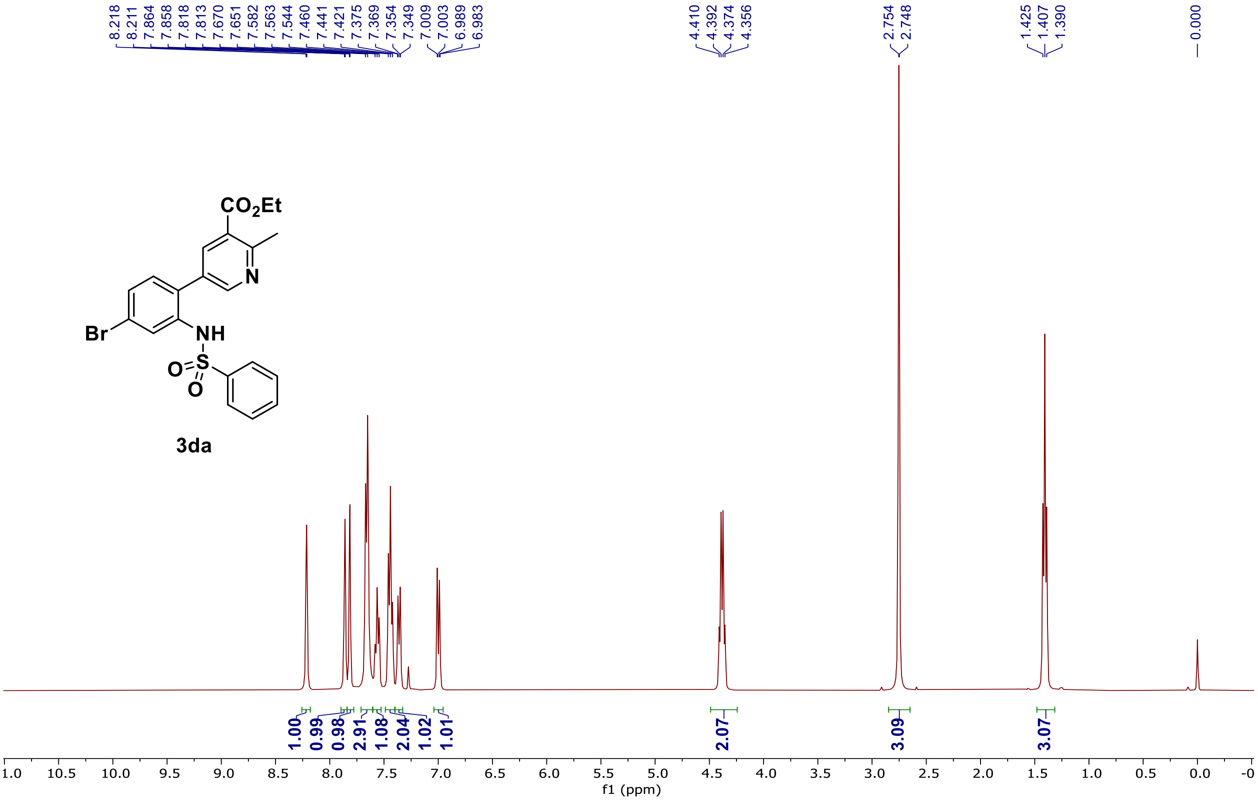

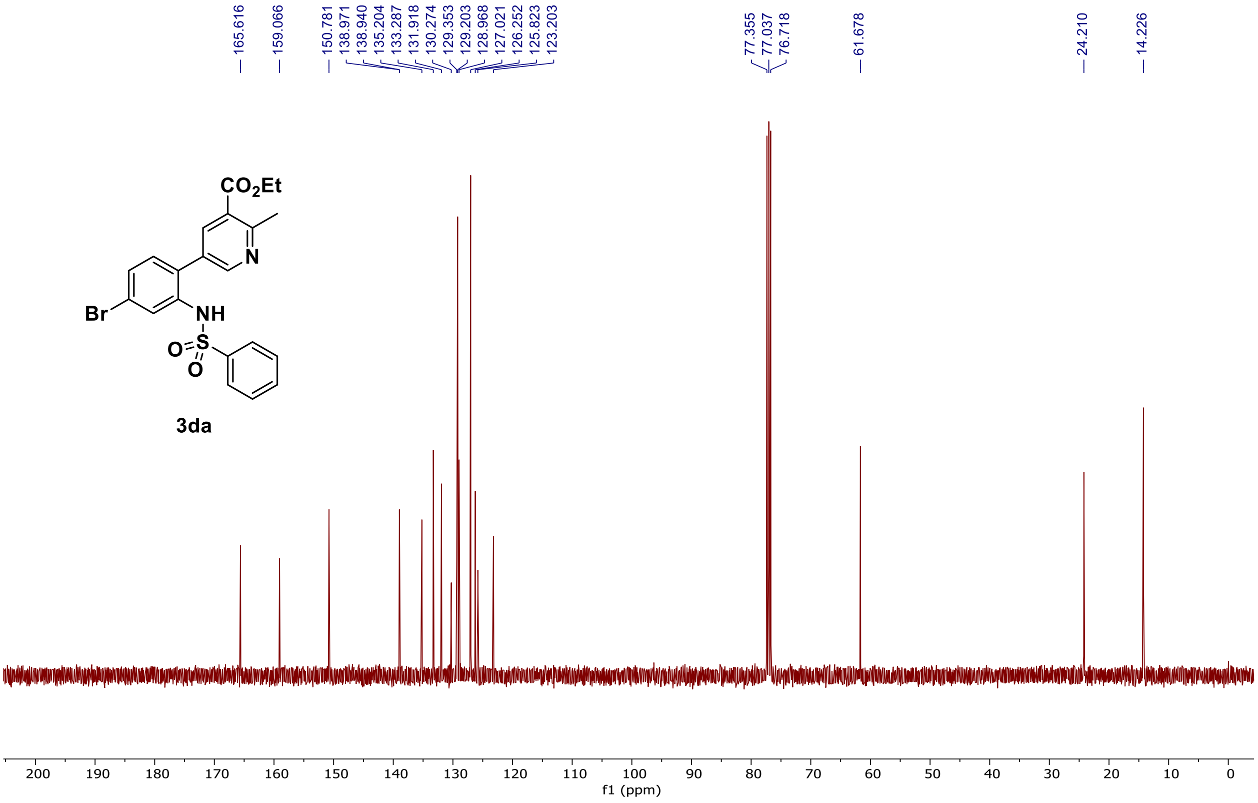

^{1}H and ^{13}C NMR Spectrum of **3da** in $CDCl_3$

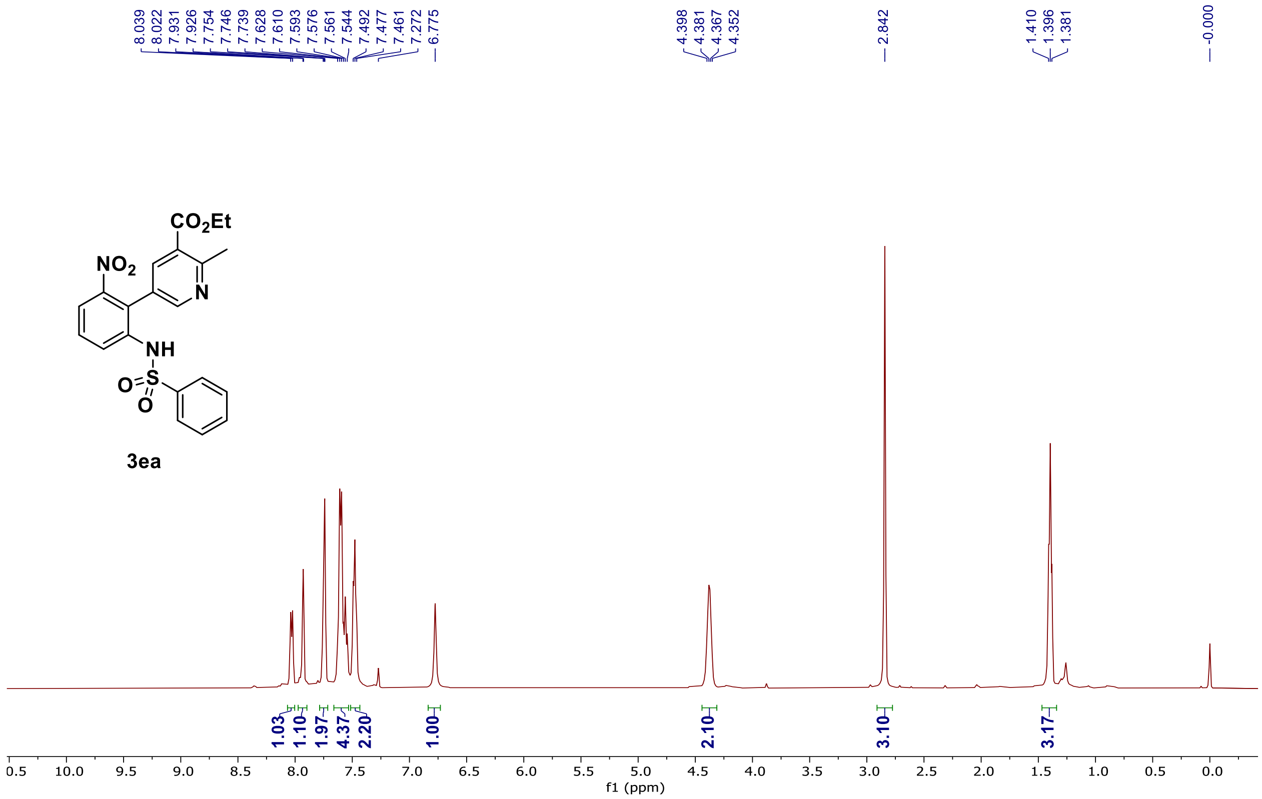

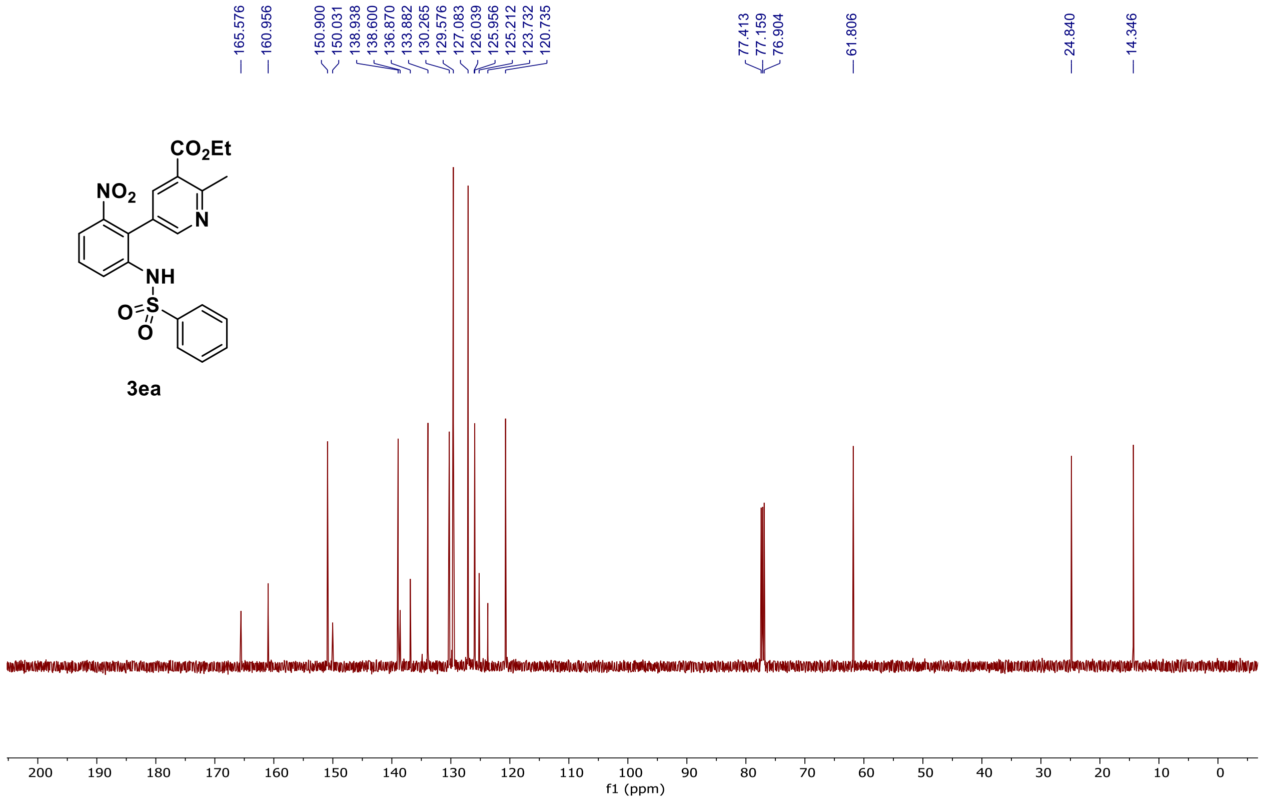

^{1}H and ^{13}C NMR Spectrum of **3ea** in $CDCl_3$

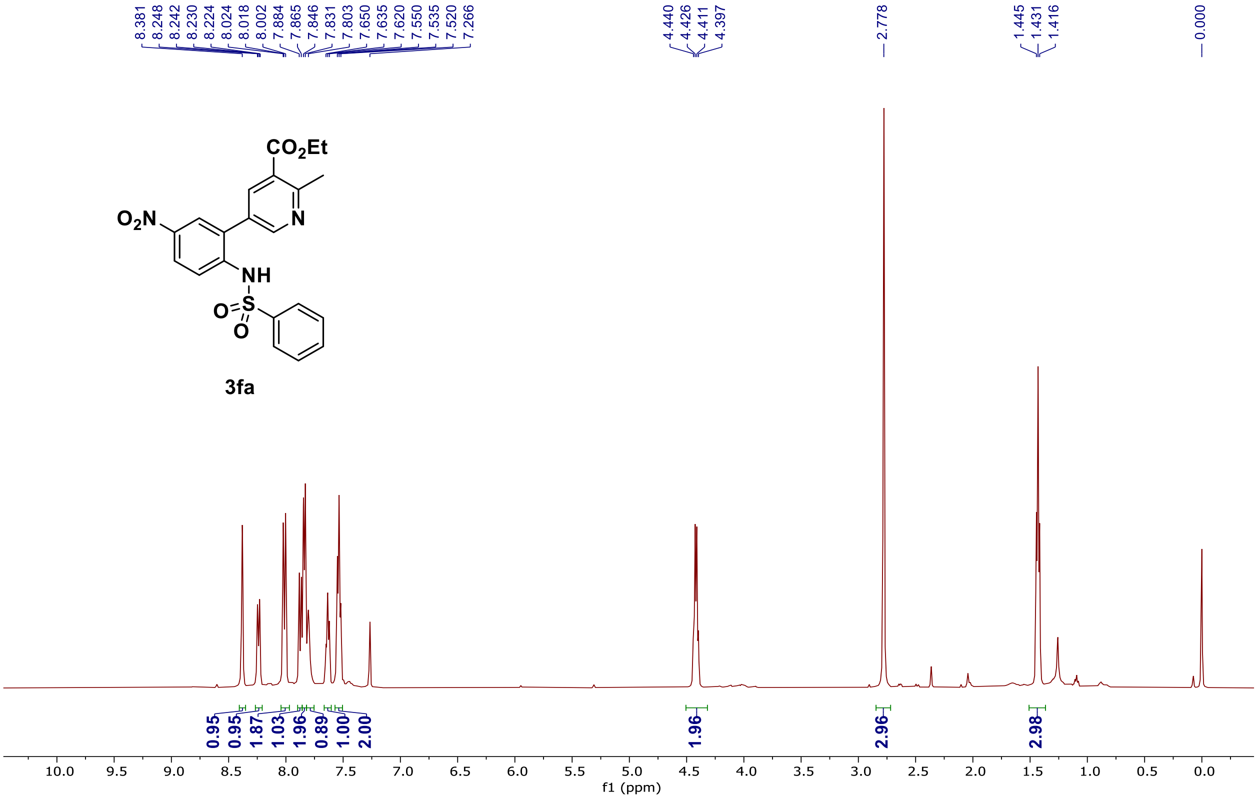

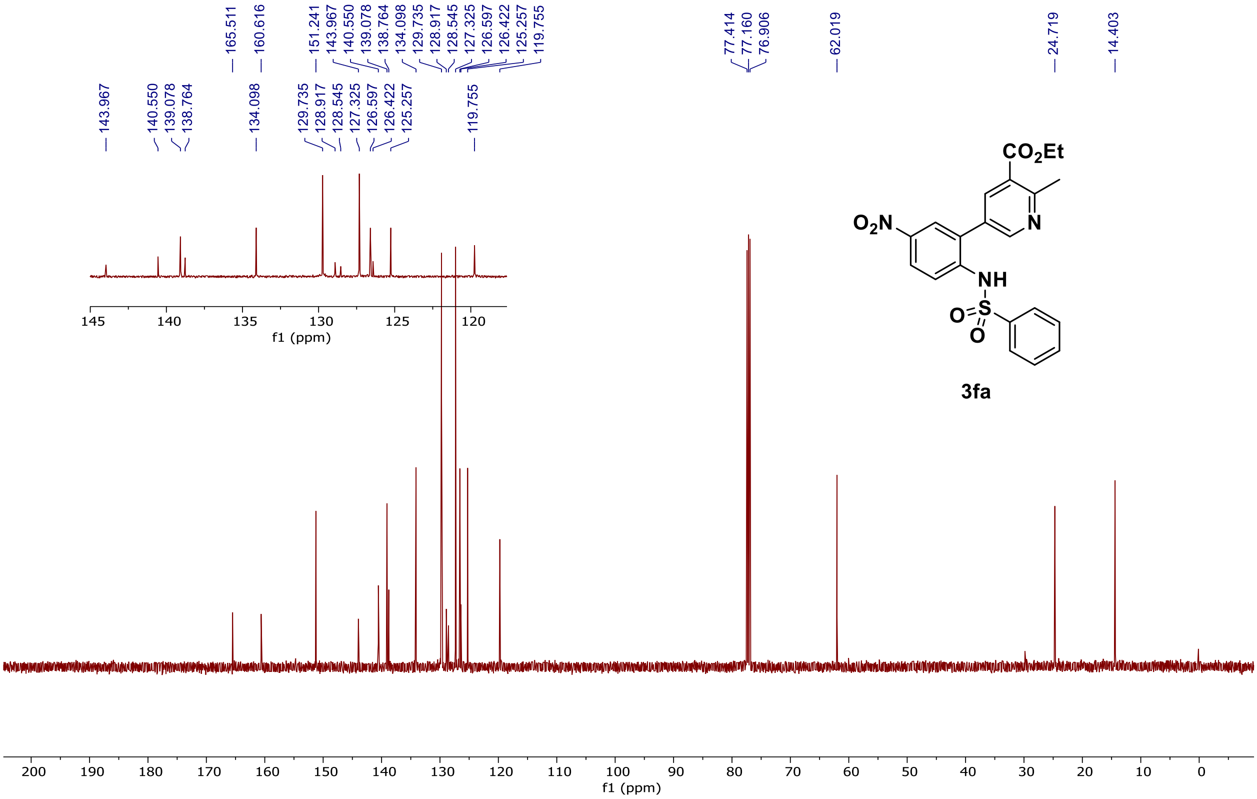

^{1}H and ^{13}C NMR Spectrum of **3fa** in $CDCl_3$

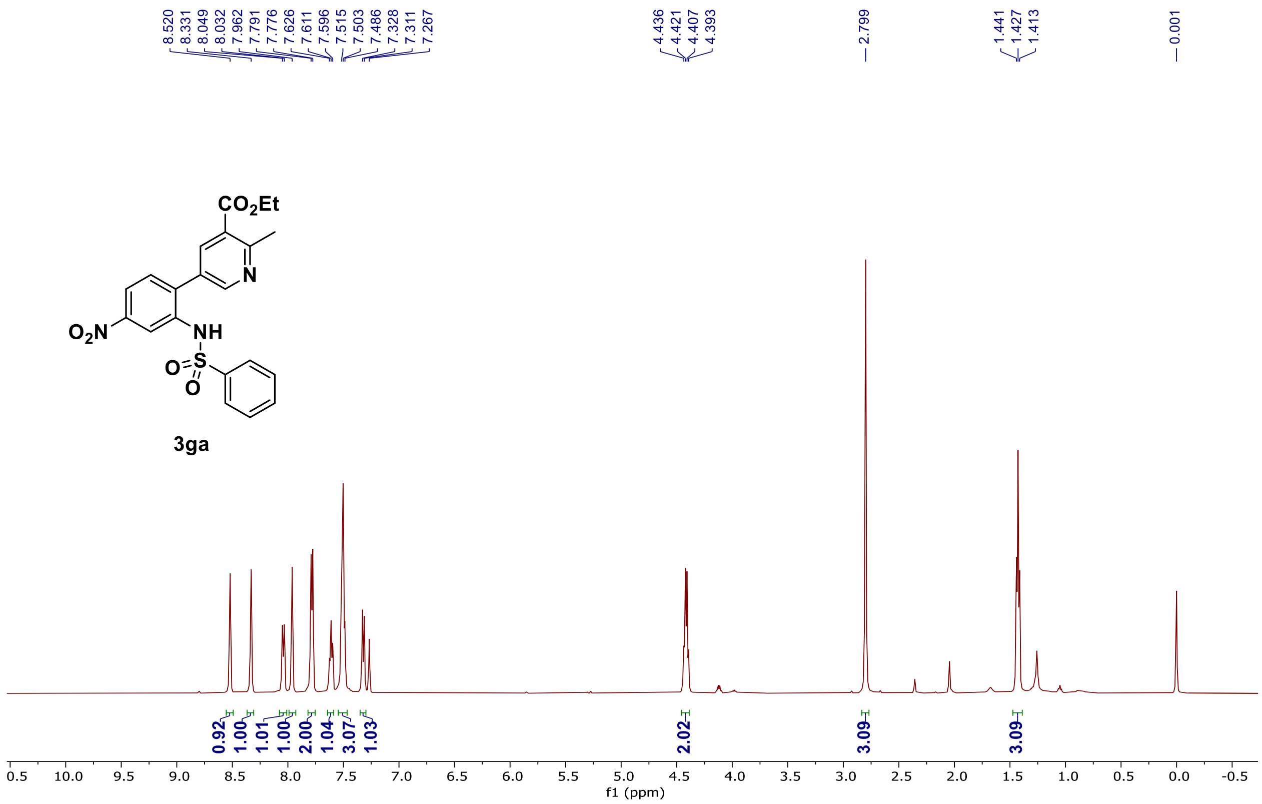

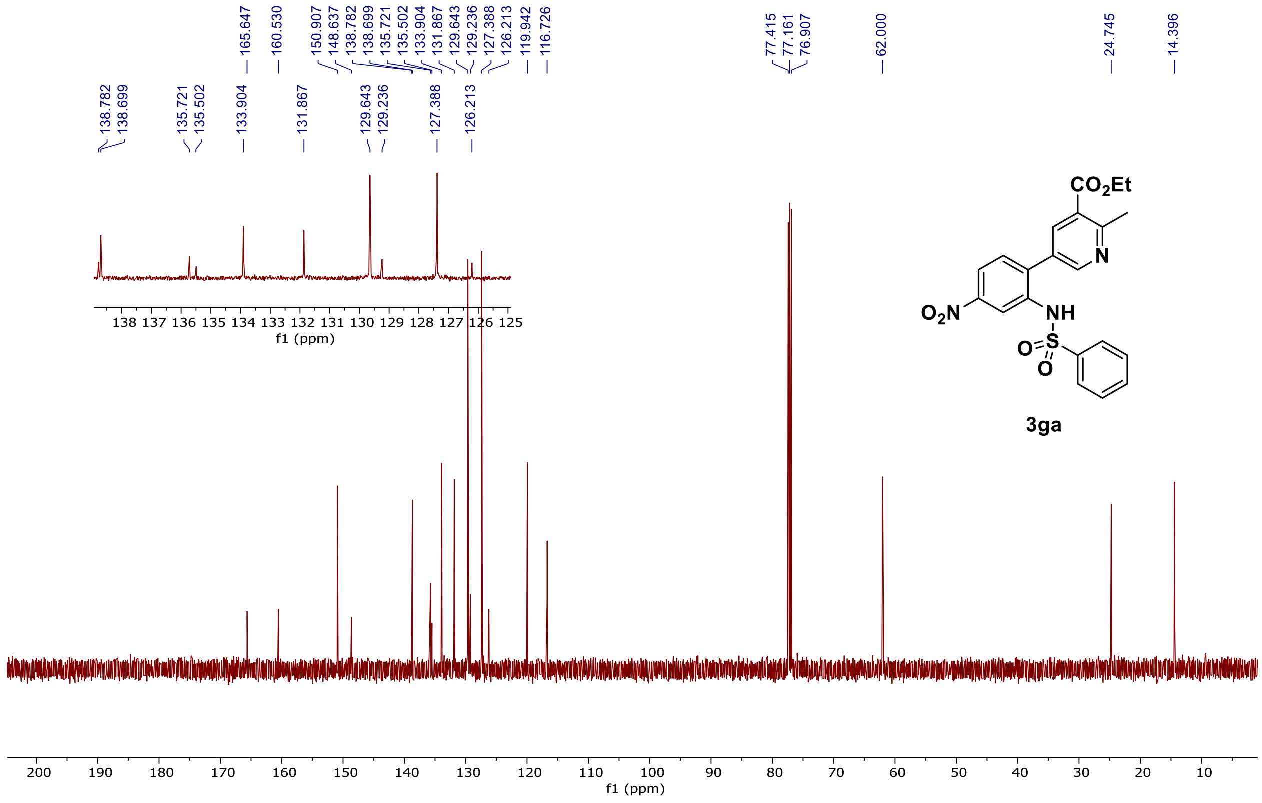

^{1}H and ^{13}C NMR Spectrum of **3ga** in $CDCl_3$

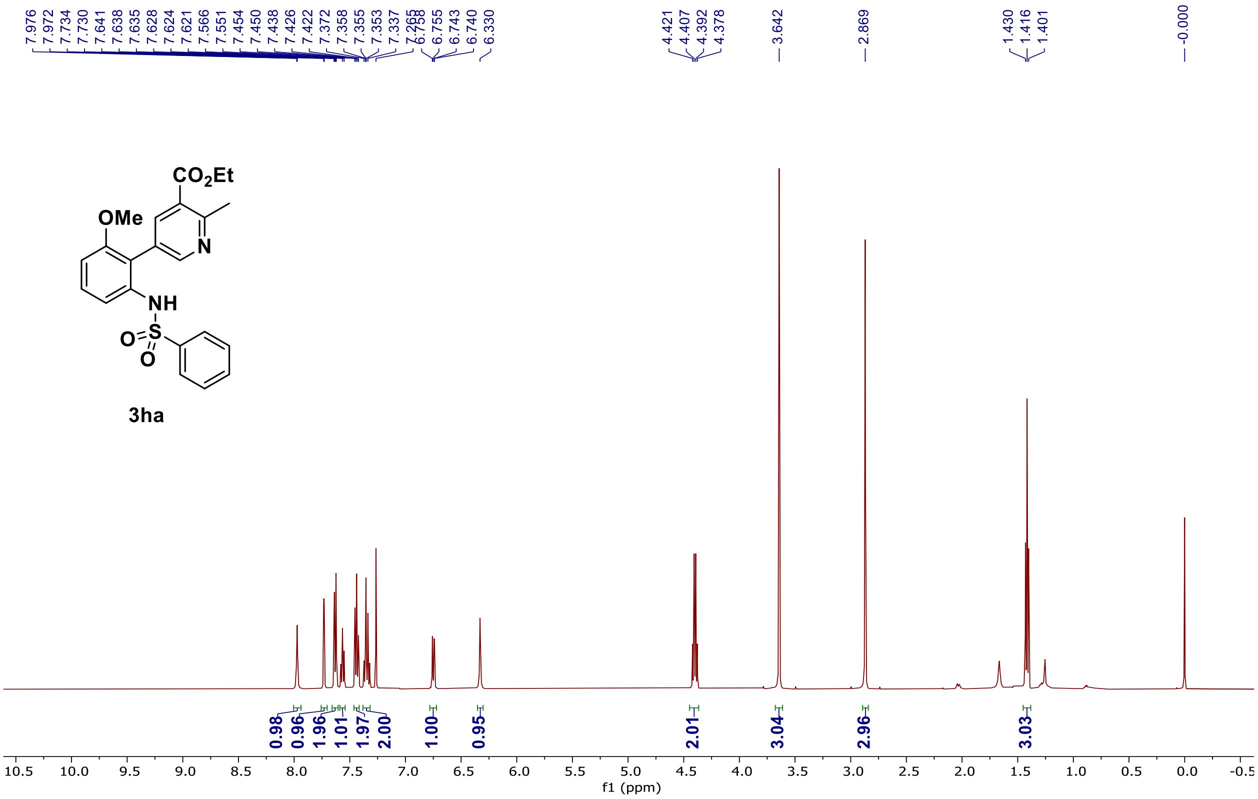

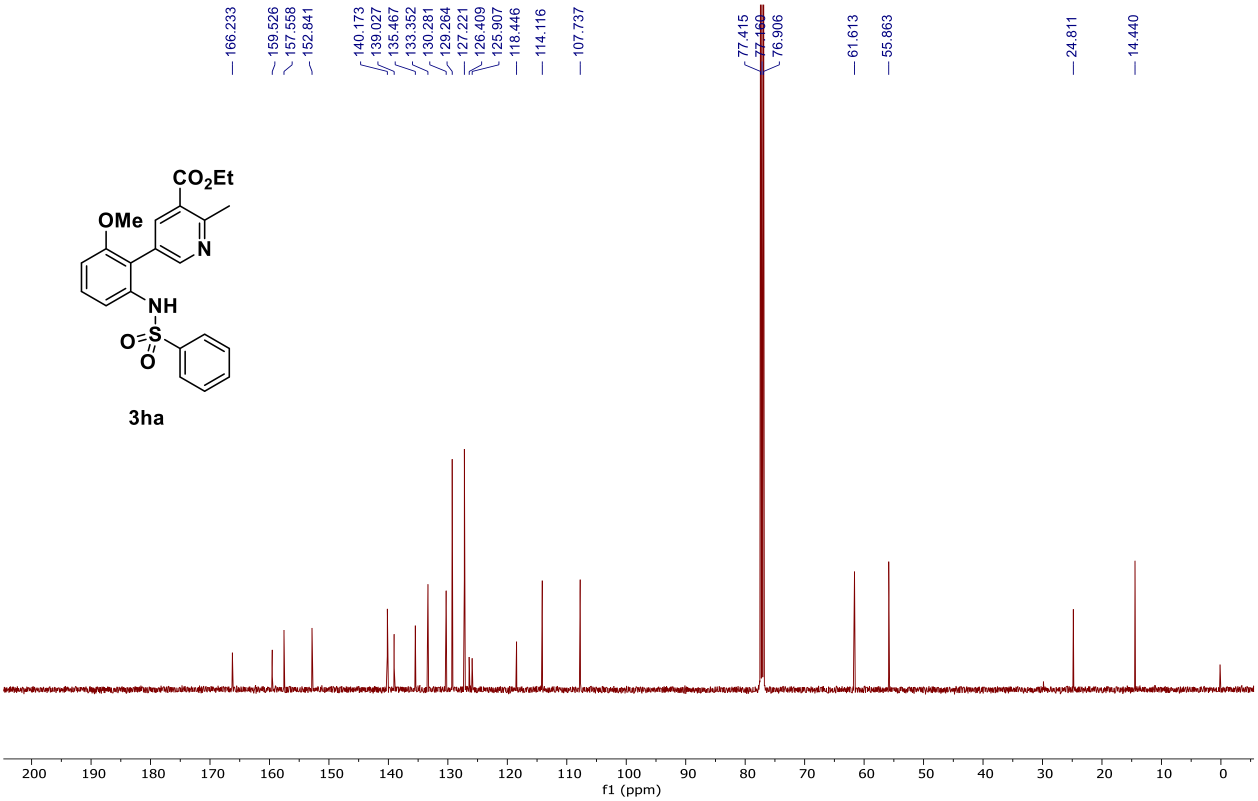

^{1}H and ^{13}C NMR Spectrum of **3ha** in $CDCl_3$

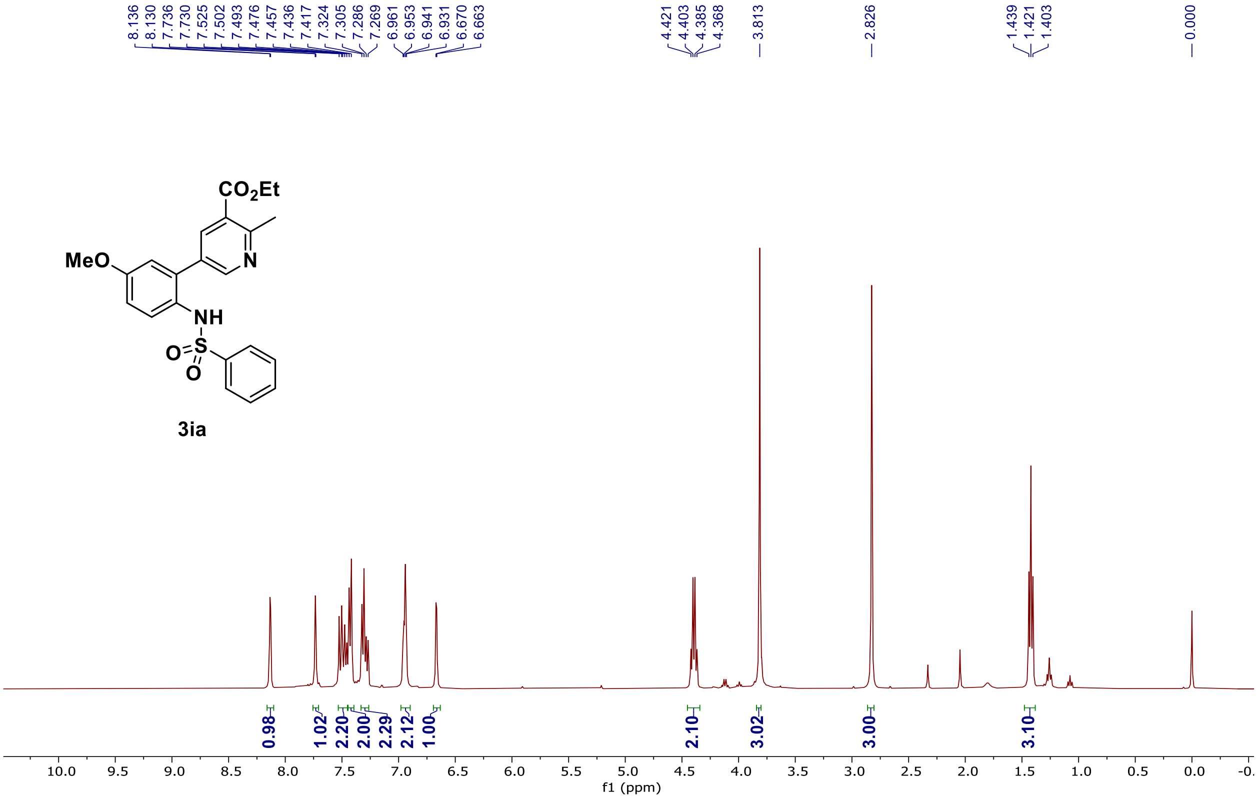

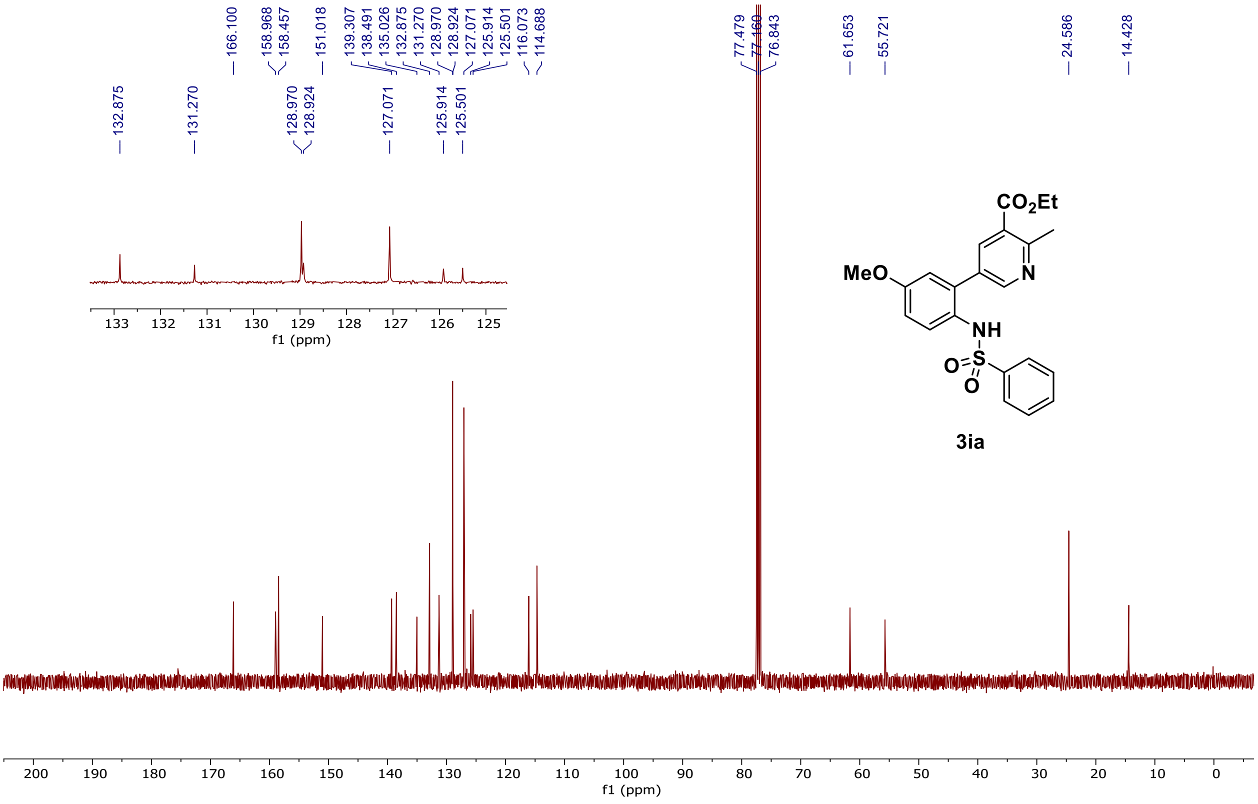

^{1}H and ^{13}C NMR Spectrum of **3ia** in $CDCl_3$

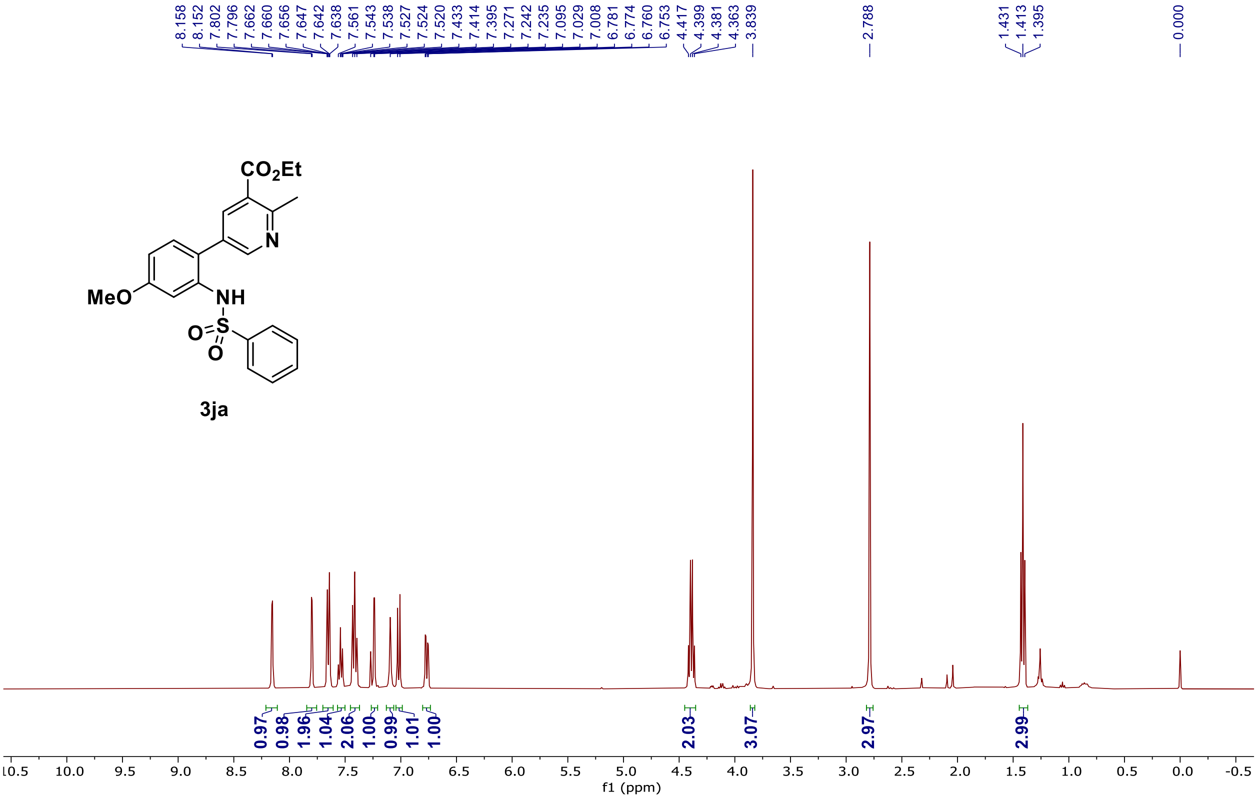

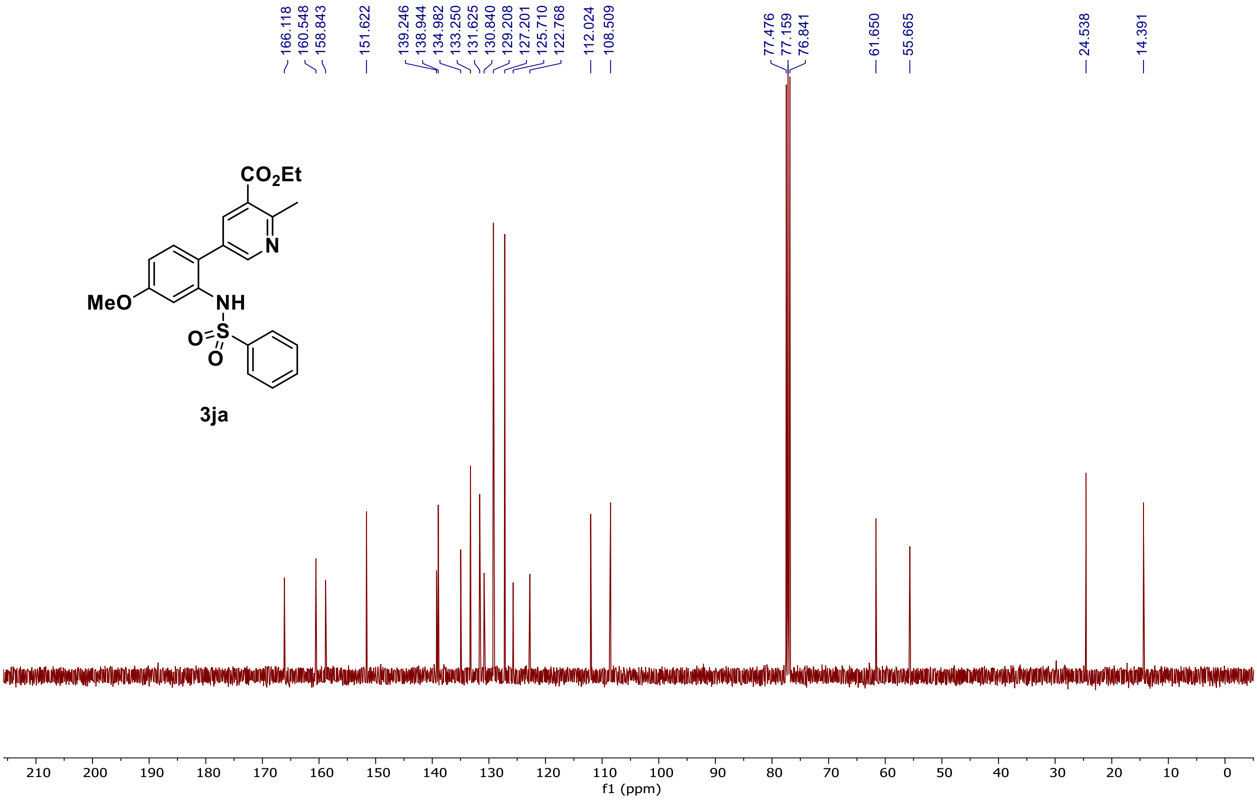

^{1}H and ^{13}C NMR Spectrum of **3ja** in $CDCl_3$

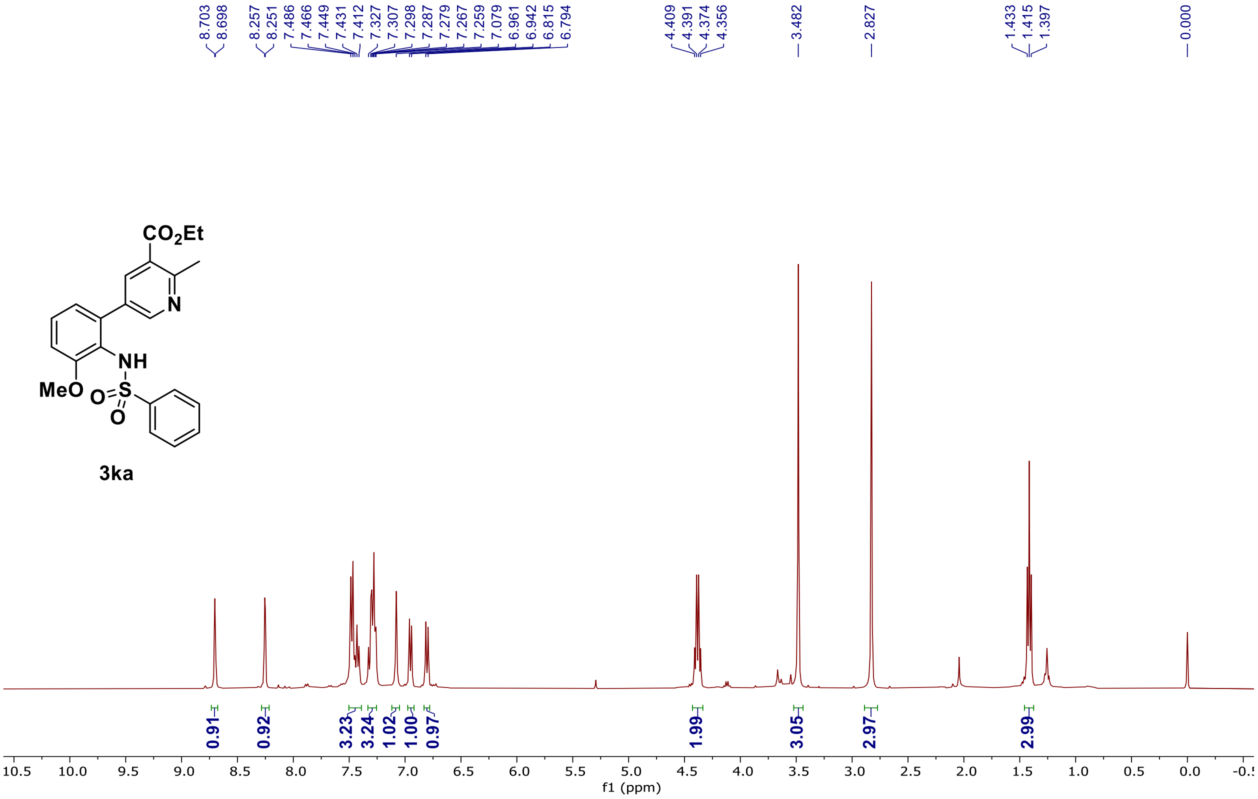

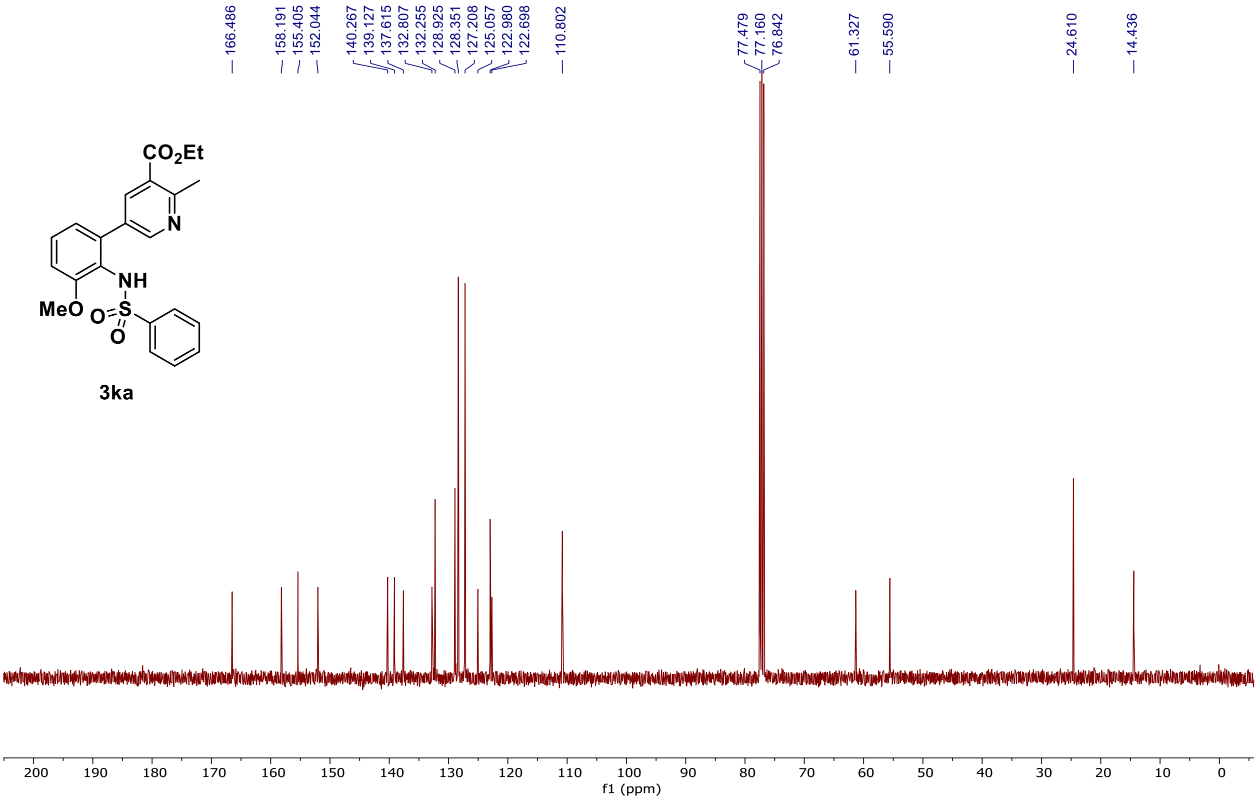

^{1}H and ^{13}C NMR Spectrum of **3ka** in $CDCl_3$

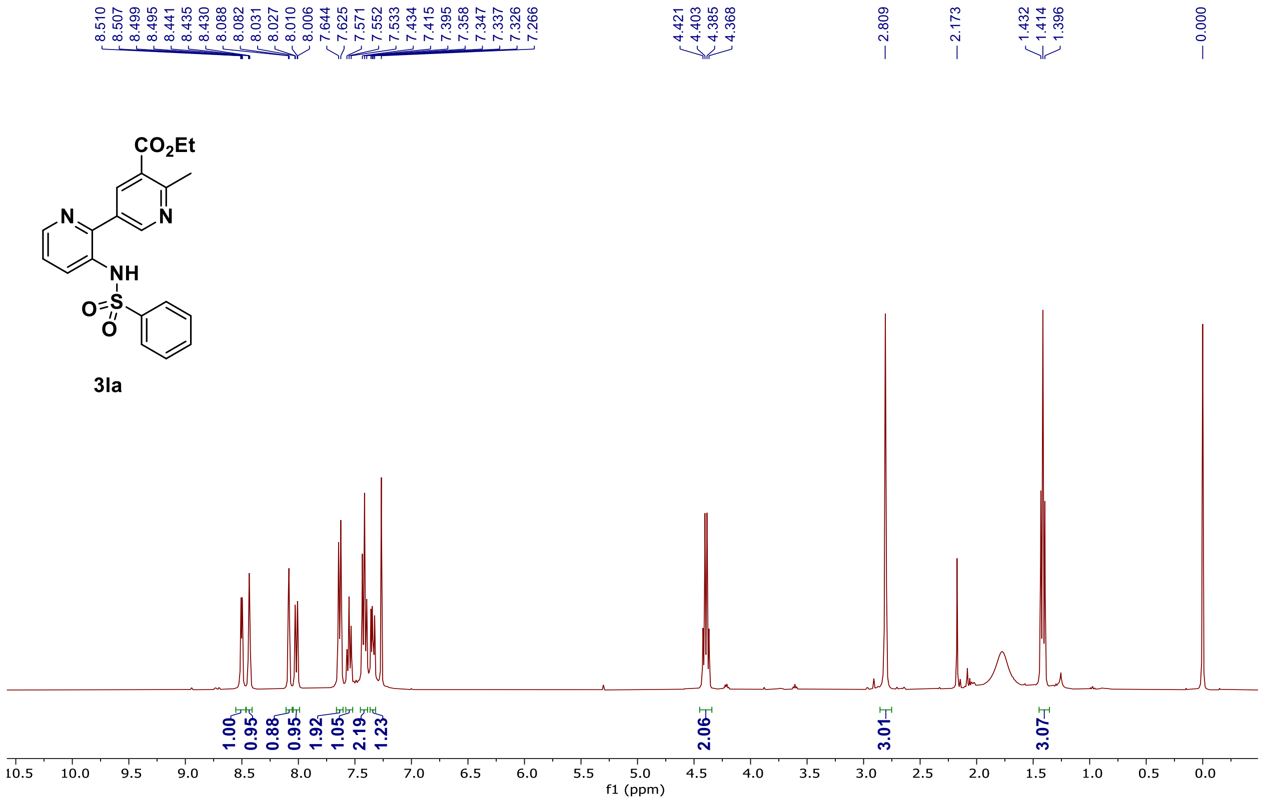

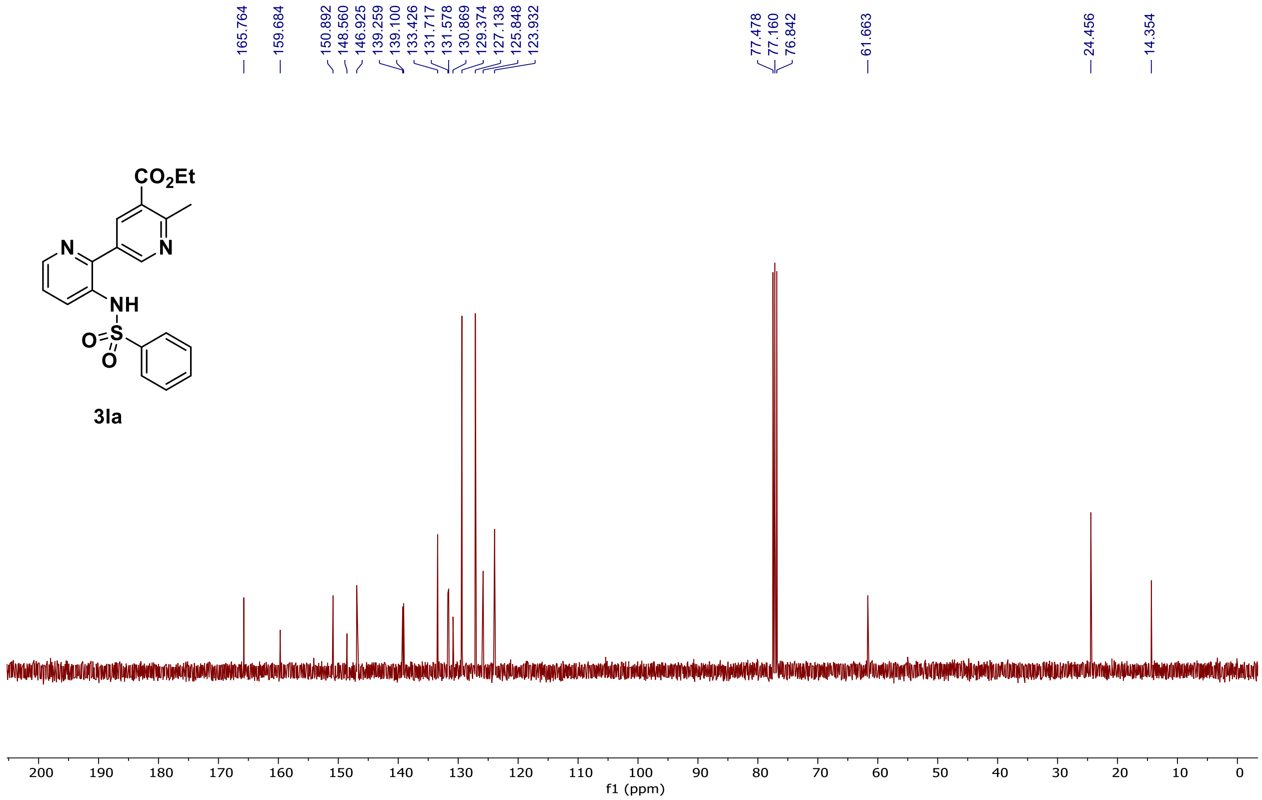

^{1}H and ^{13}C NMR Spectrum of **3la** in $CDCl_3$

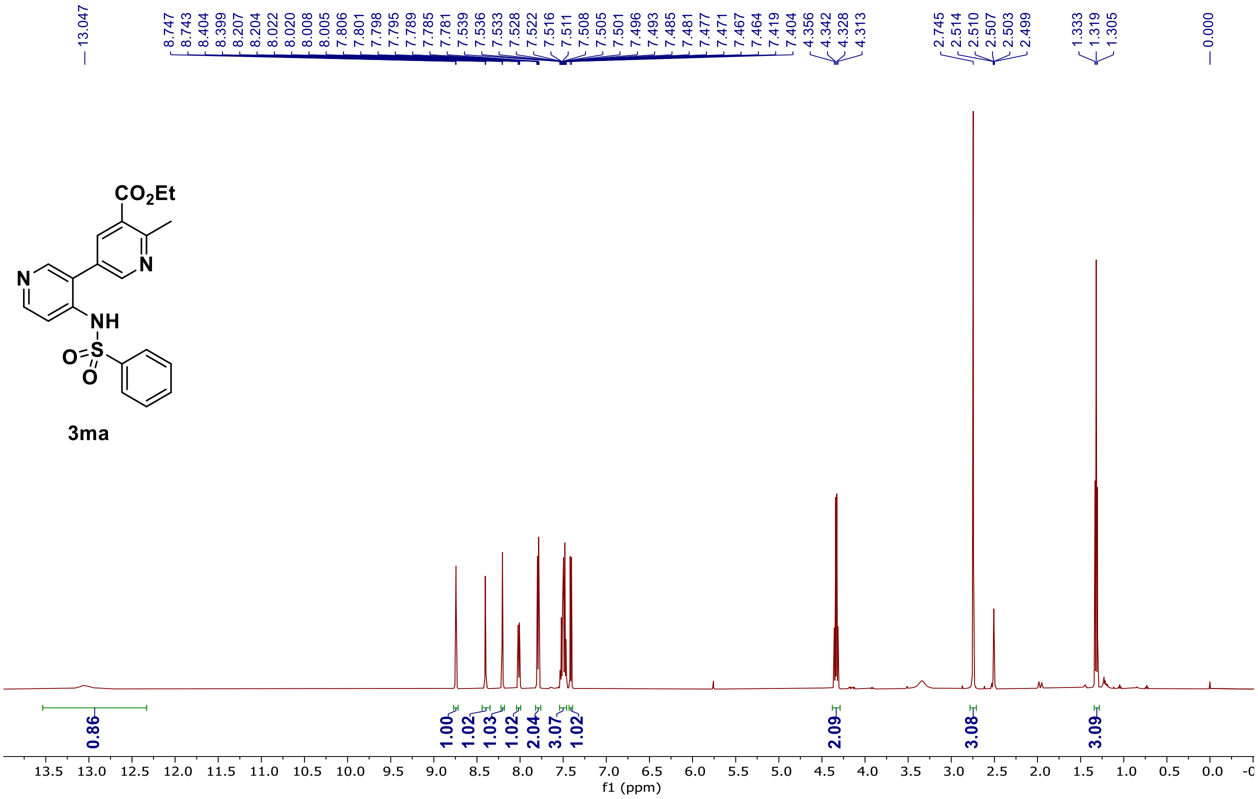

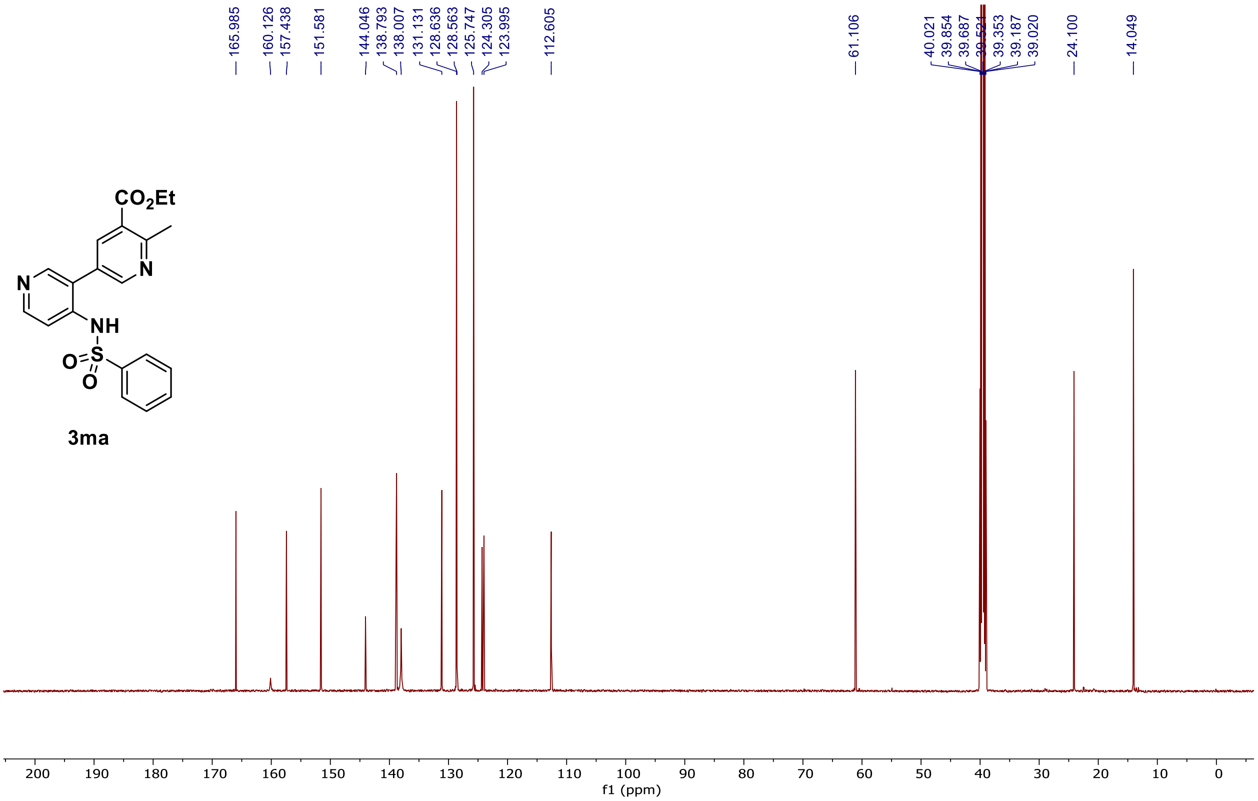

^{1}H and ^{13}C NMR Spectrum of **3ma** in DMSO-d_6

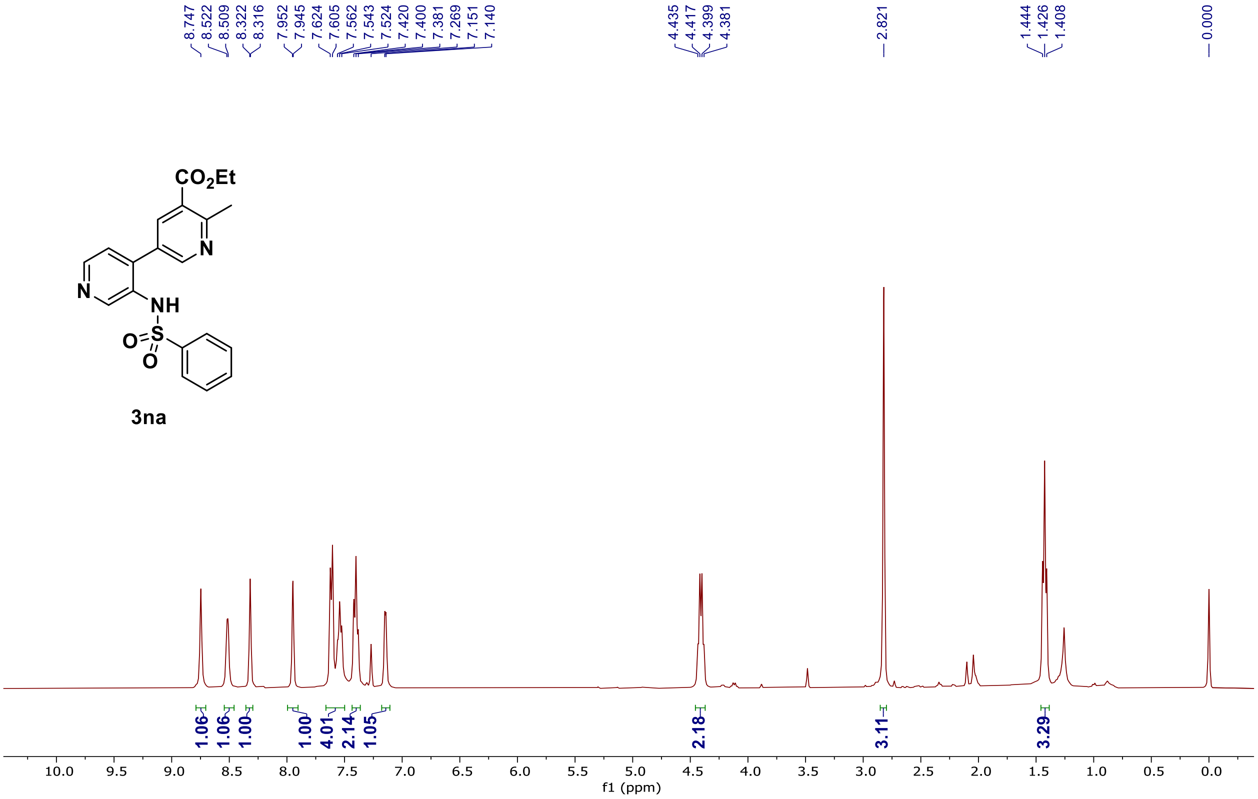

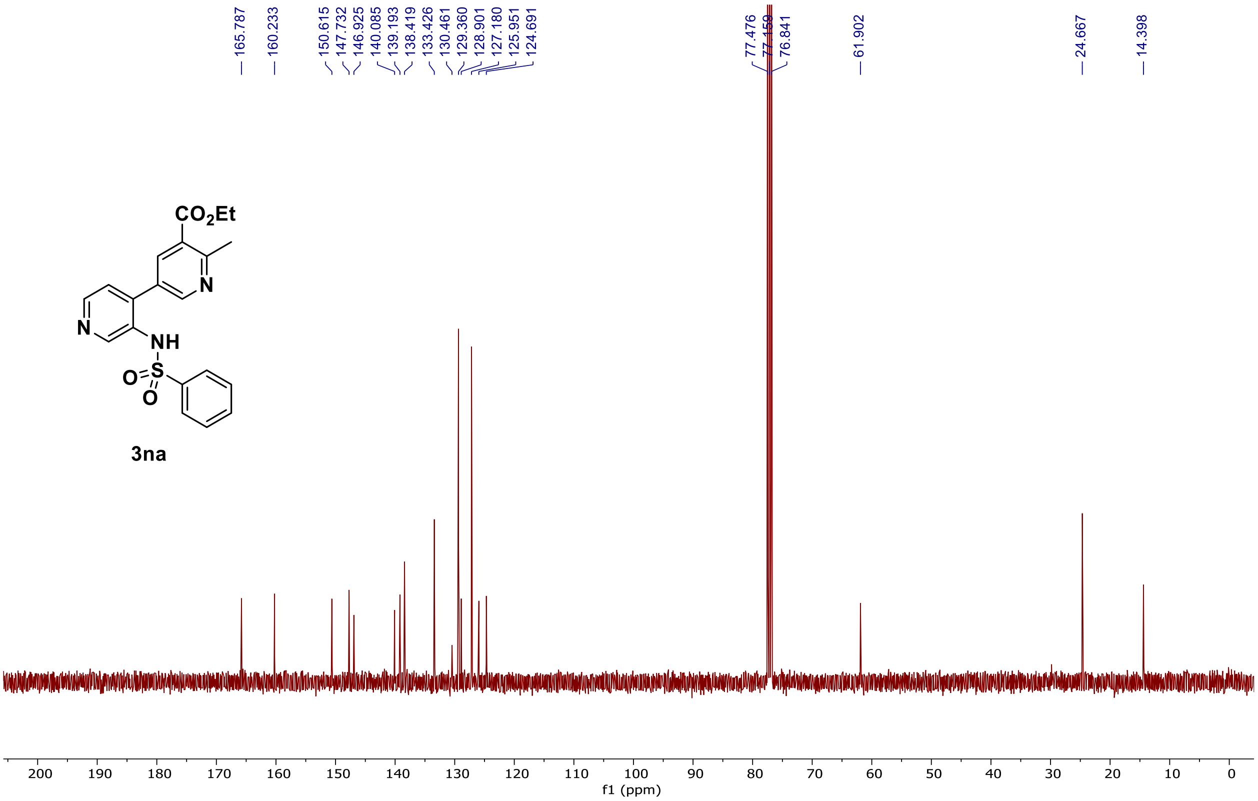

^{1}H and ^{13}C NMR Spectrum of **3na** in $CDCl_3$

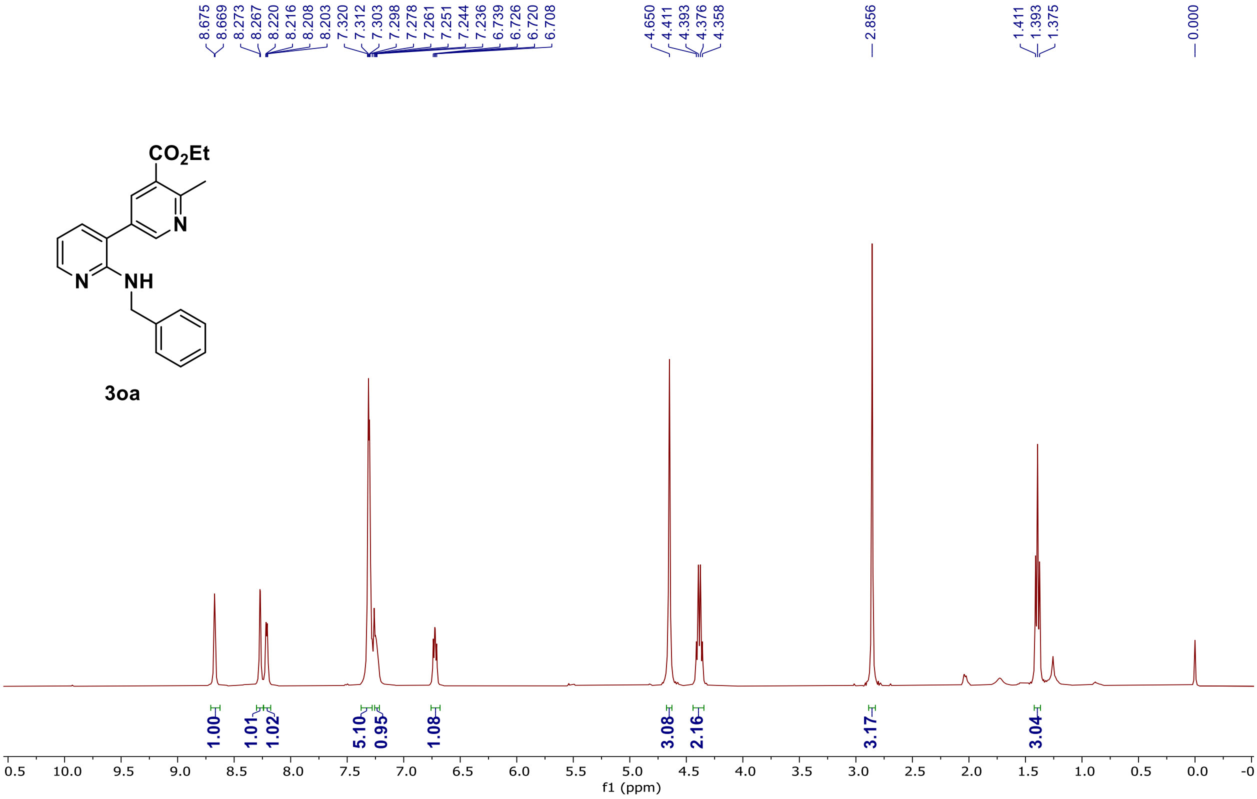

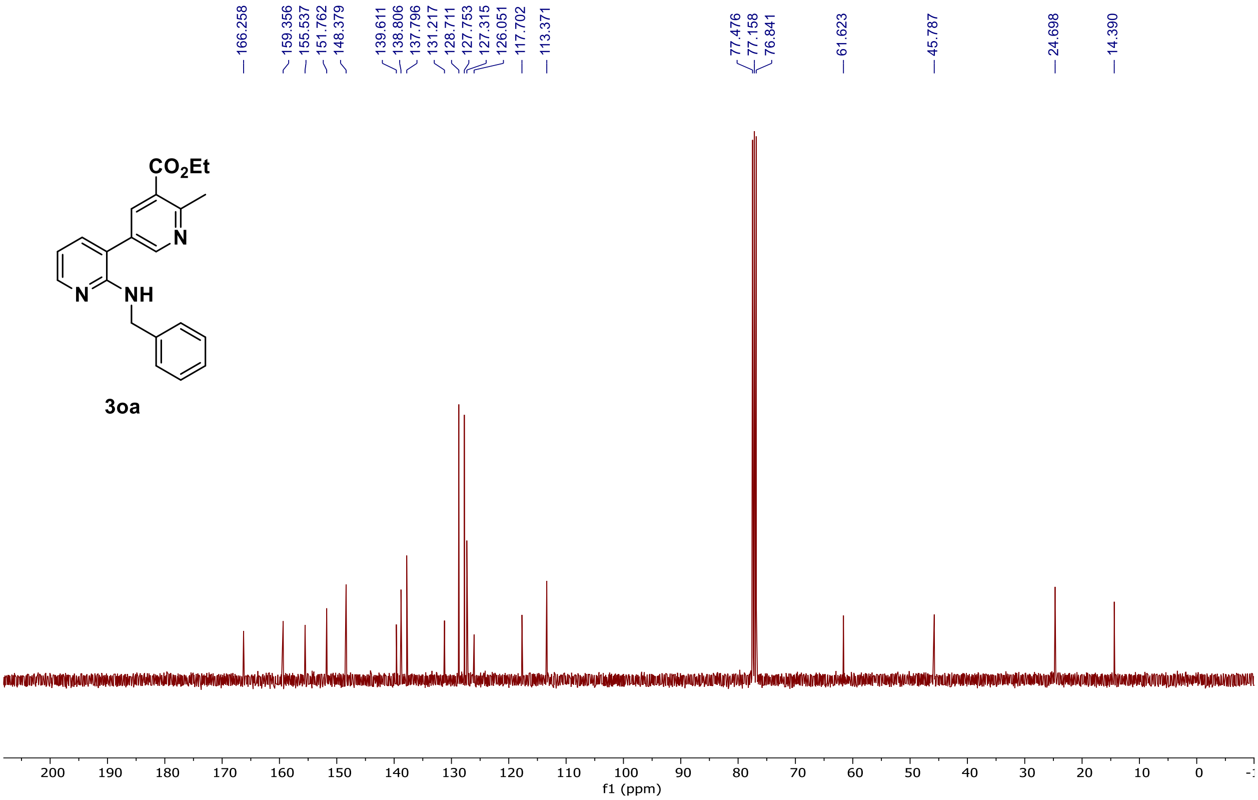

^1H and ^{13}C NMR Spectrum of **3oa** in $CDCl_3$

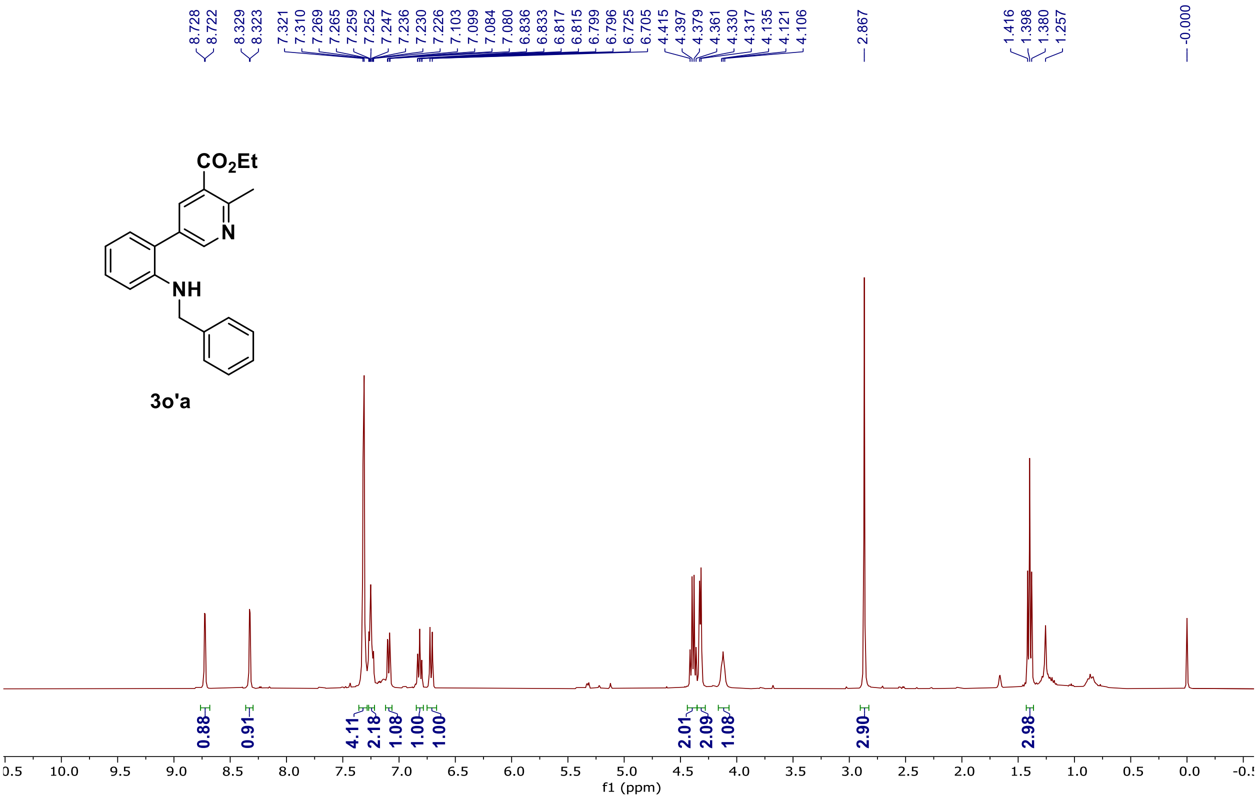

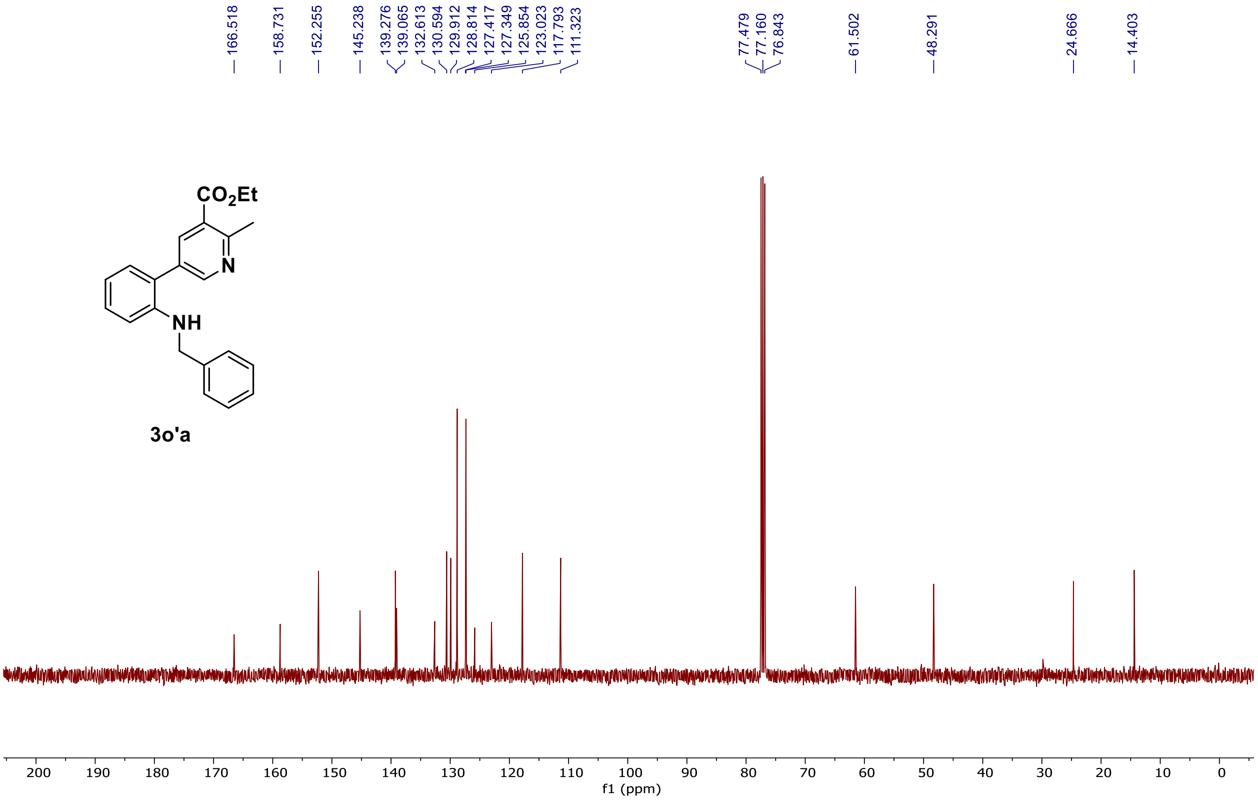

^{1}H and ^{13}C NMR Spectrum of **3o'a** in $CDCl_3$

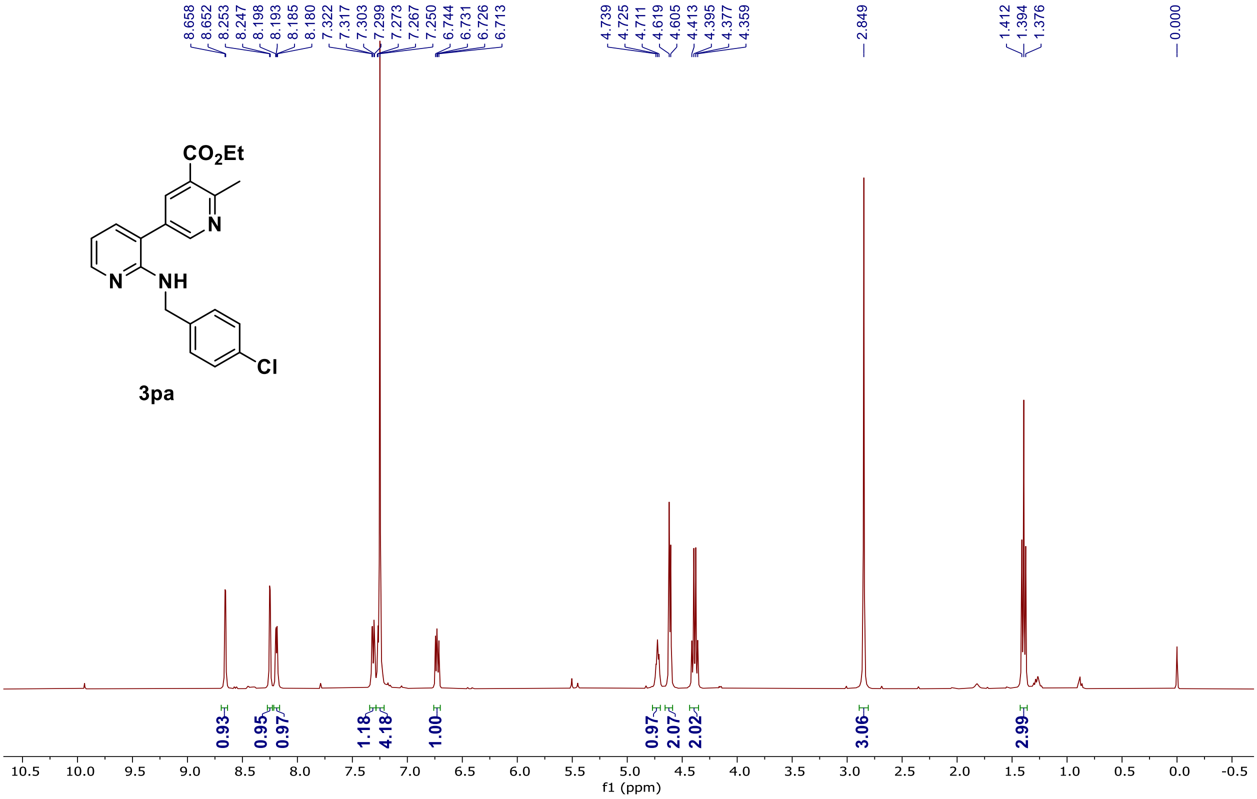

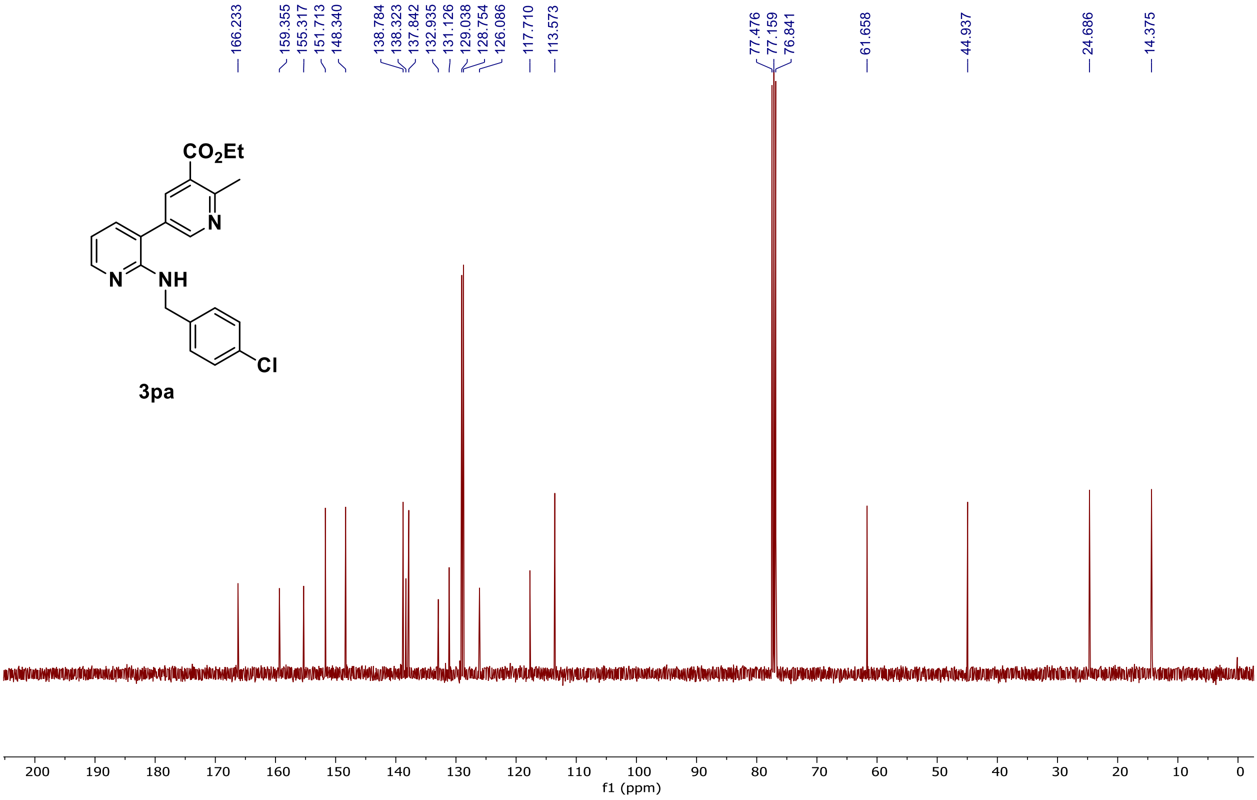

^{1}H and ^{13}C NMR Spectrum of **3pa** in $CDCl_3$

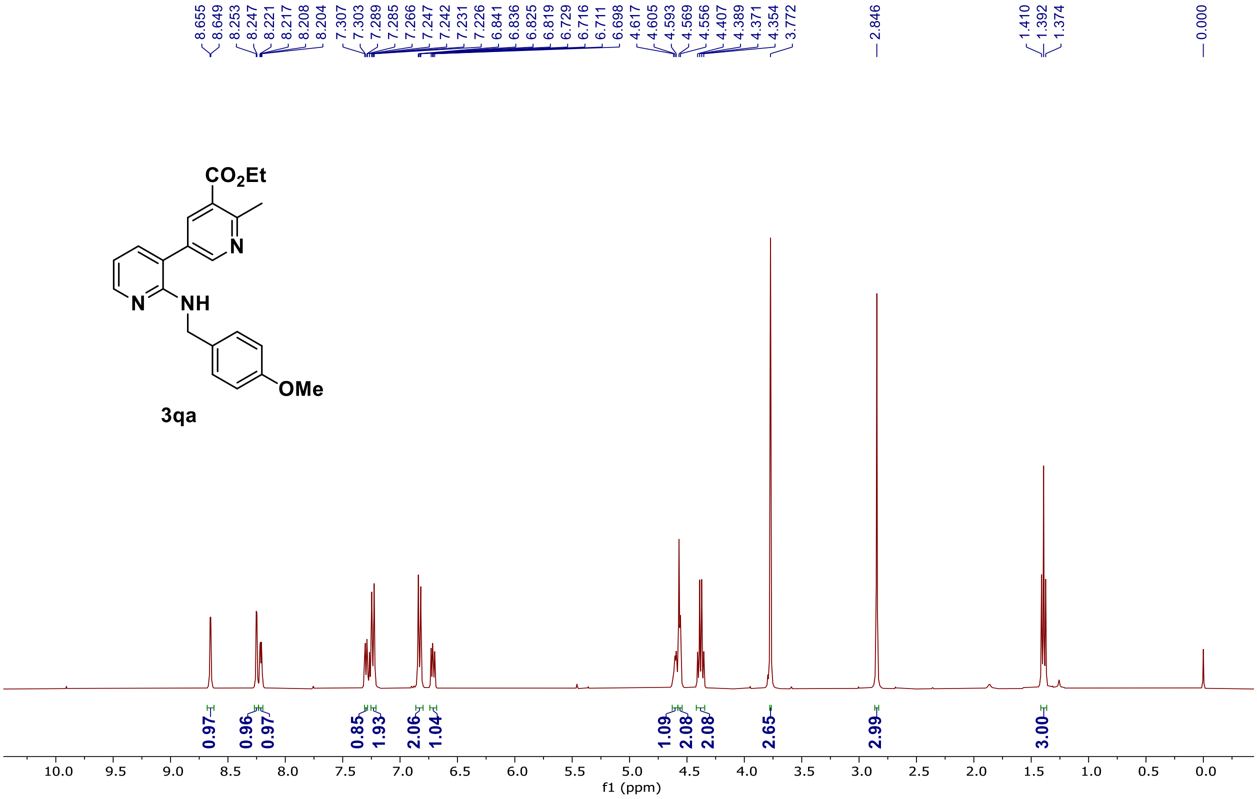

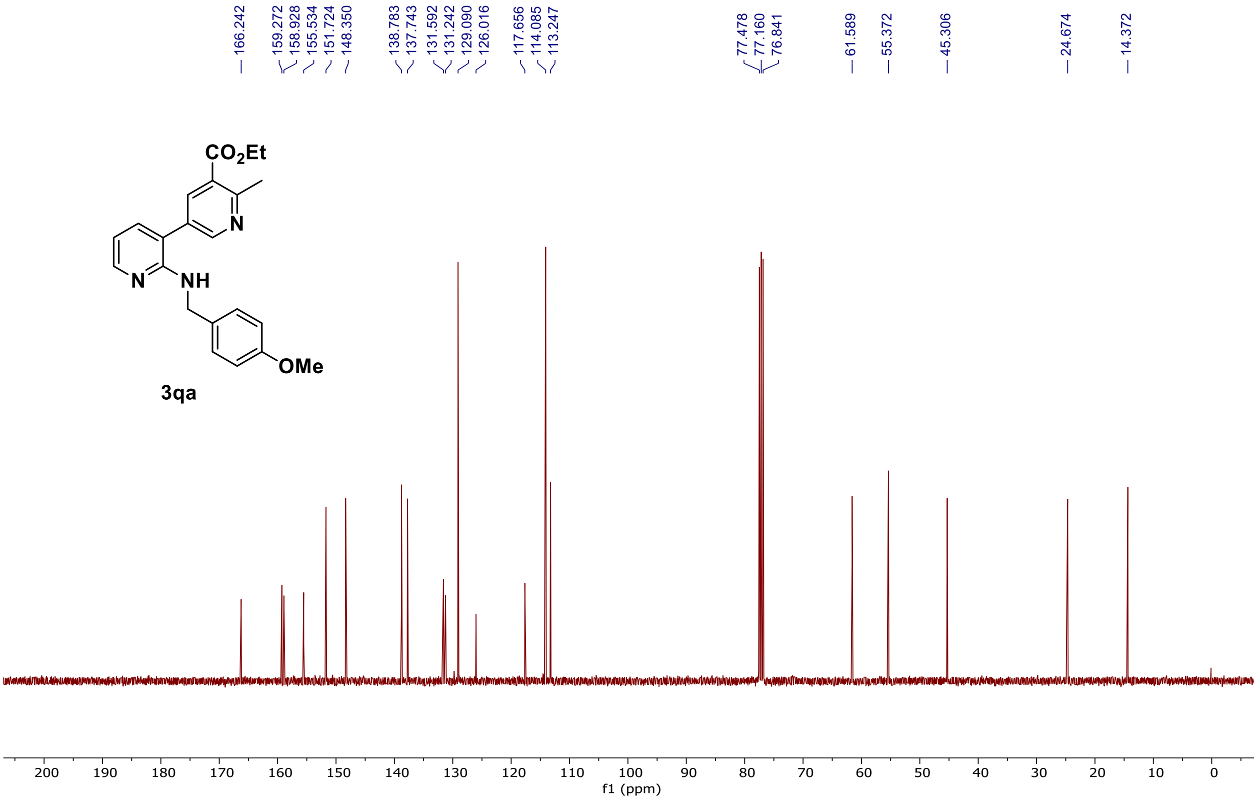

^{1}H and ^{13}C NMR Spectrum of **3qa** in $CDCl_3$

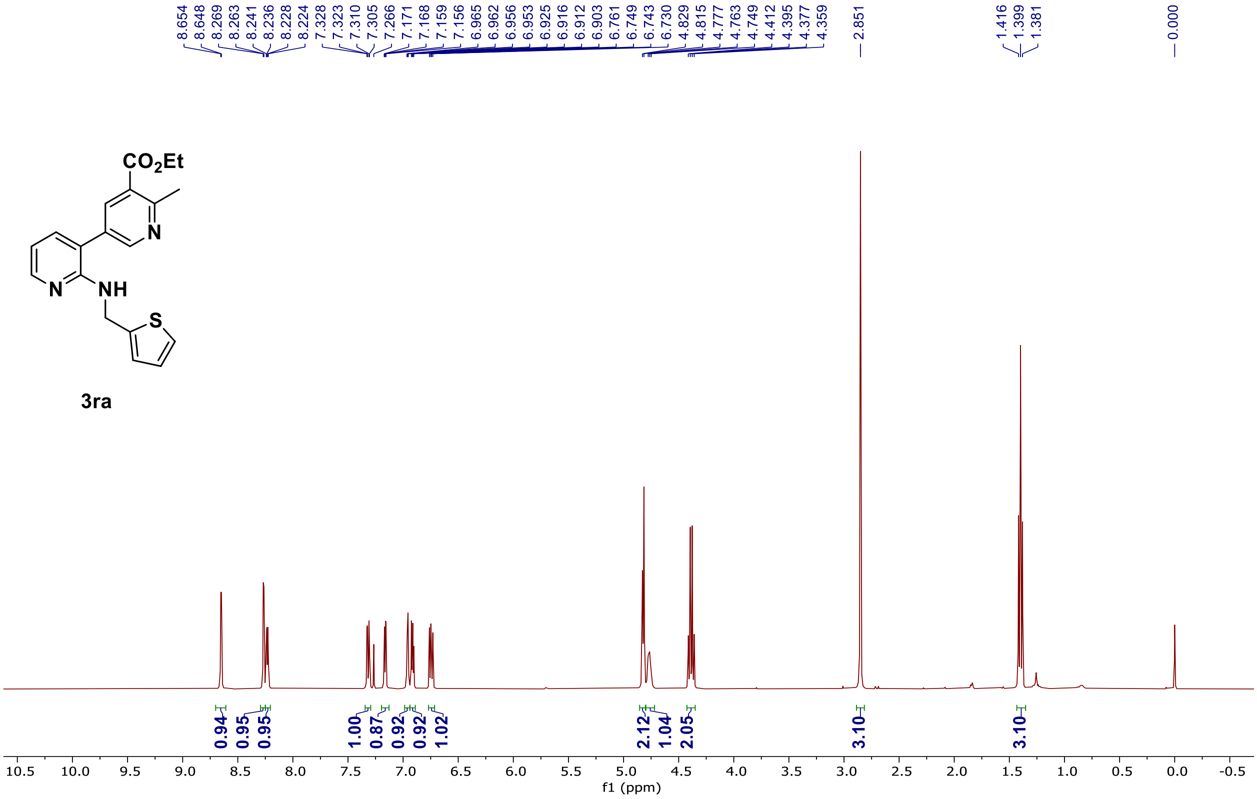

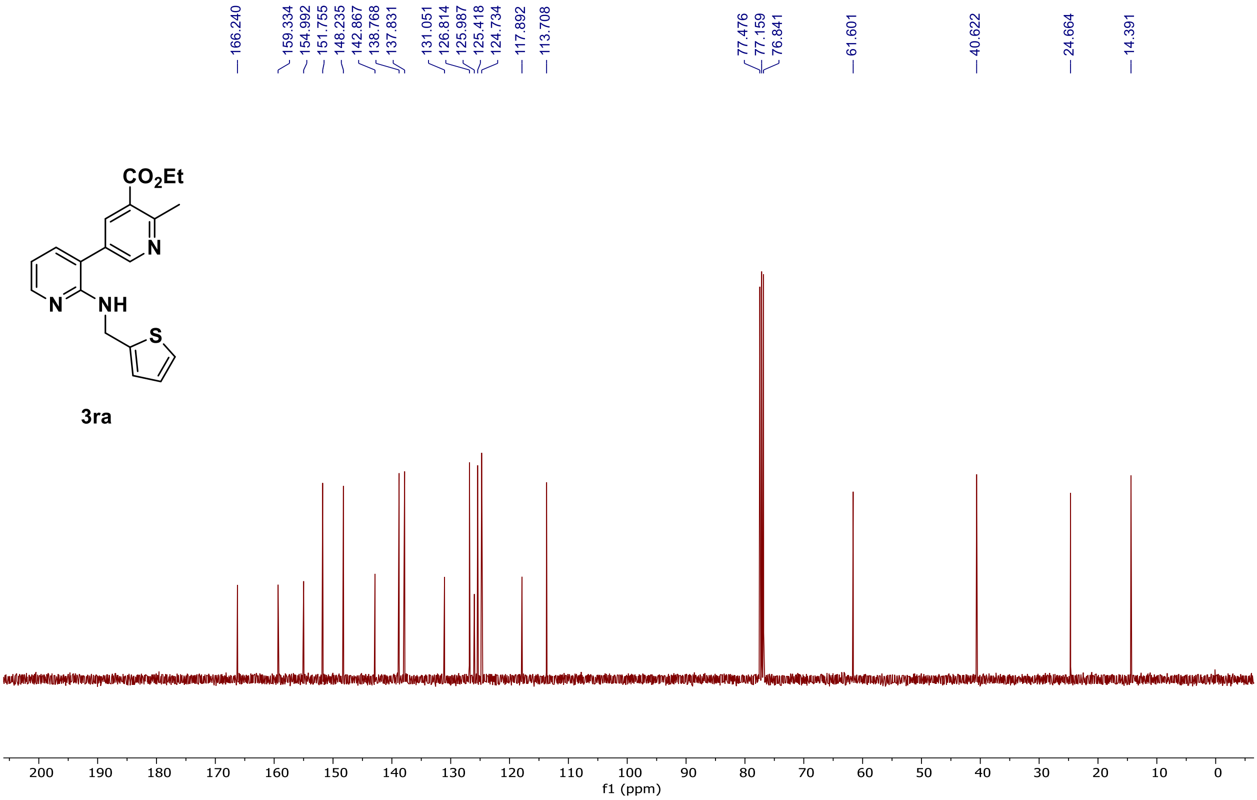

^{1}H and ^{13}C NMR Spectrum of **3ra** in $CDCl_3$

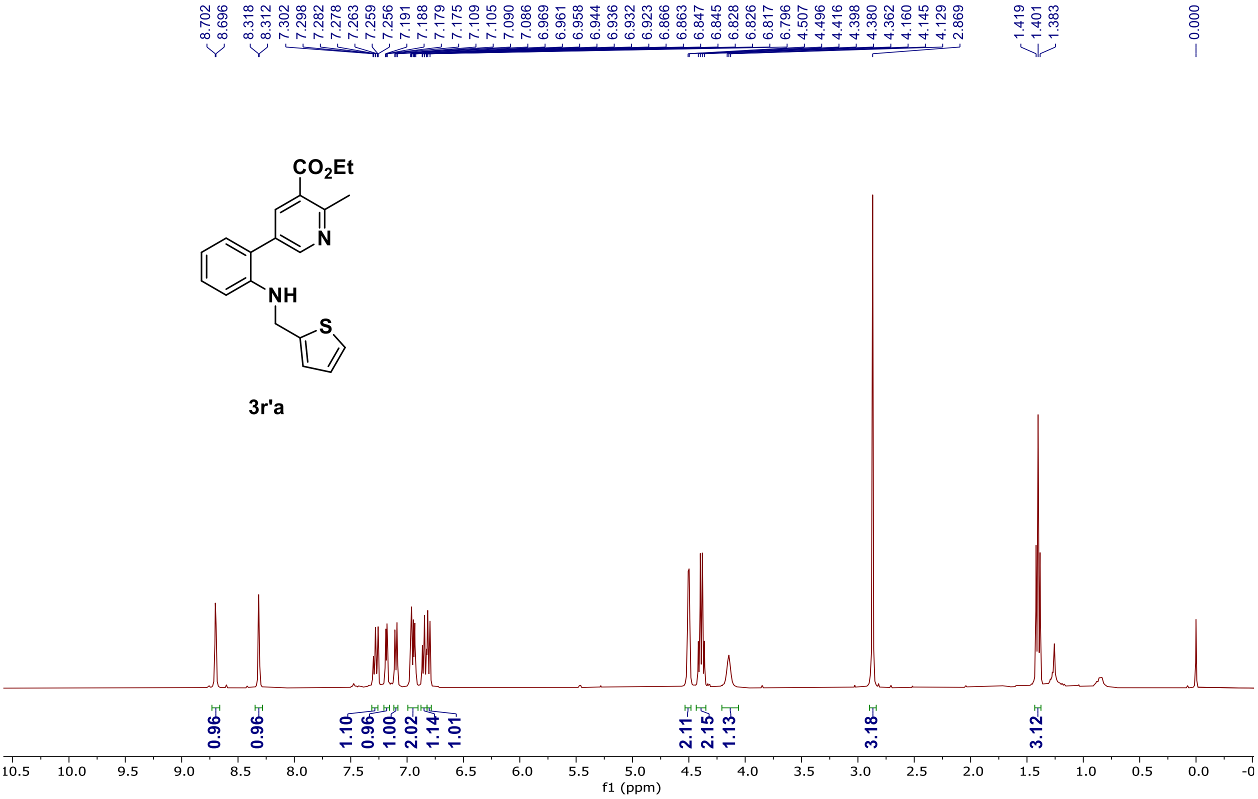

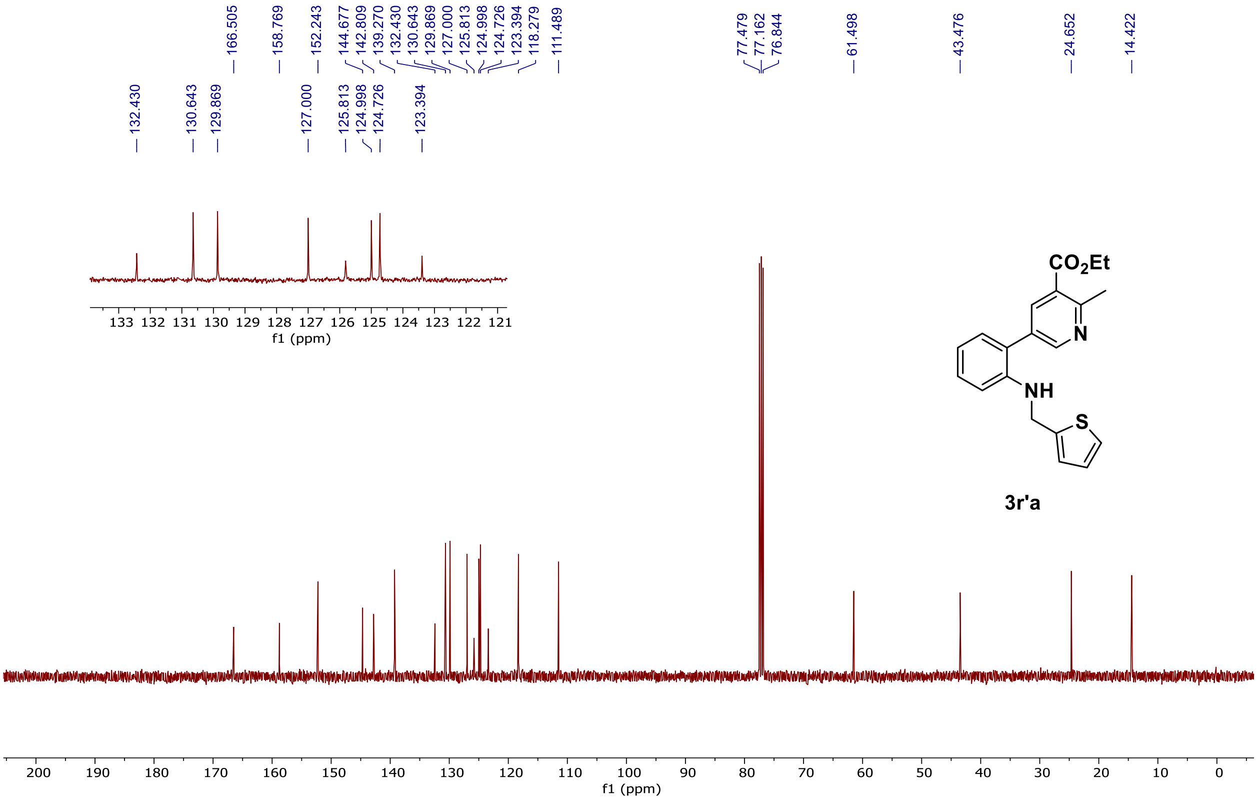

^{1}H and ^{13}C NMR Spectrum of **3r'a** in $CDCl_3$

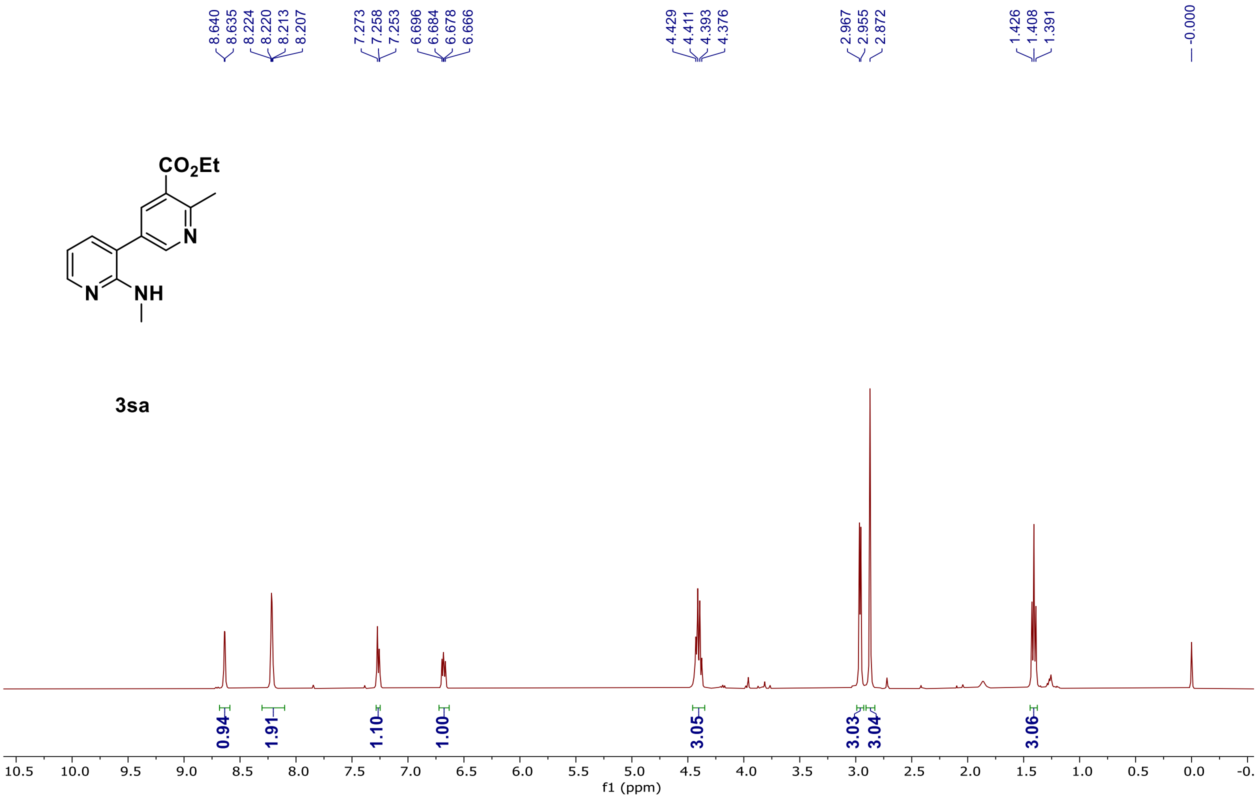

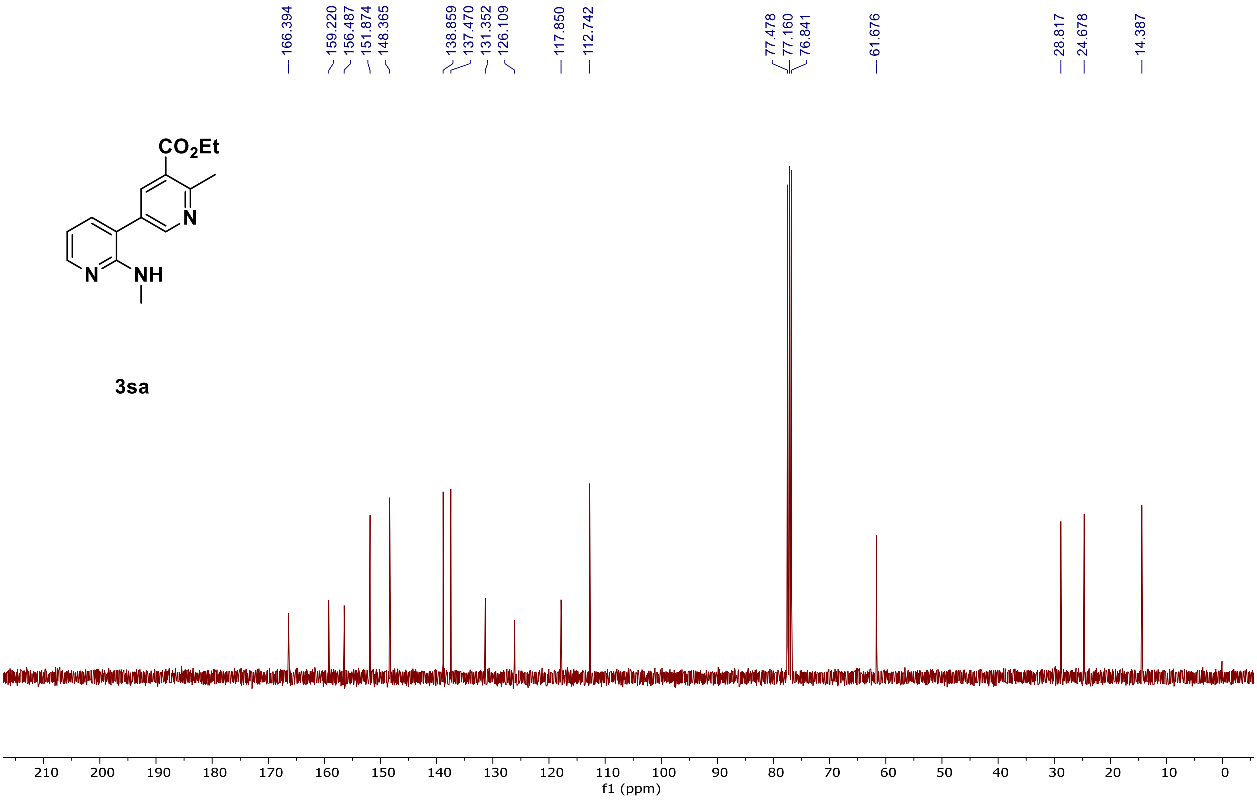

^{1}H and ^{13}C NMR Spectrum of **3sa** in $CDCl_3$

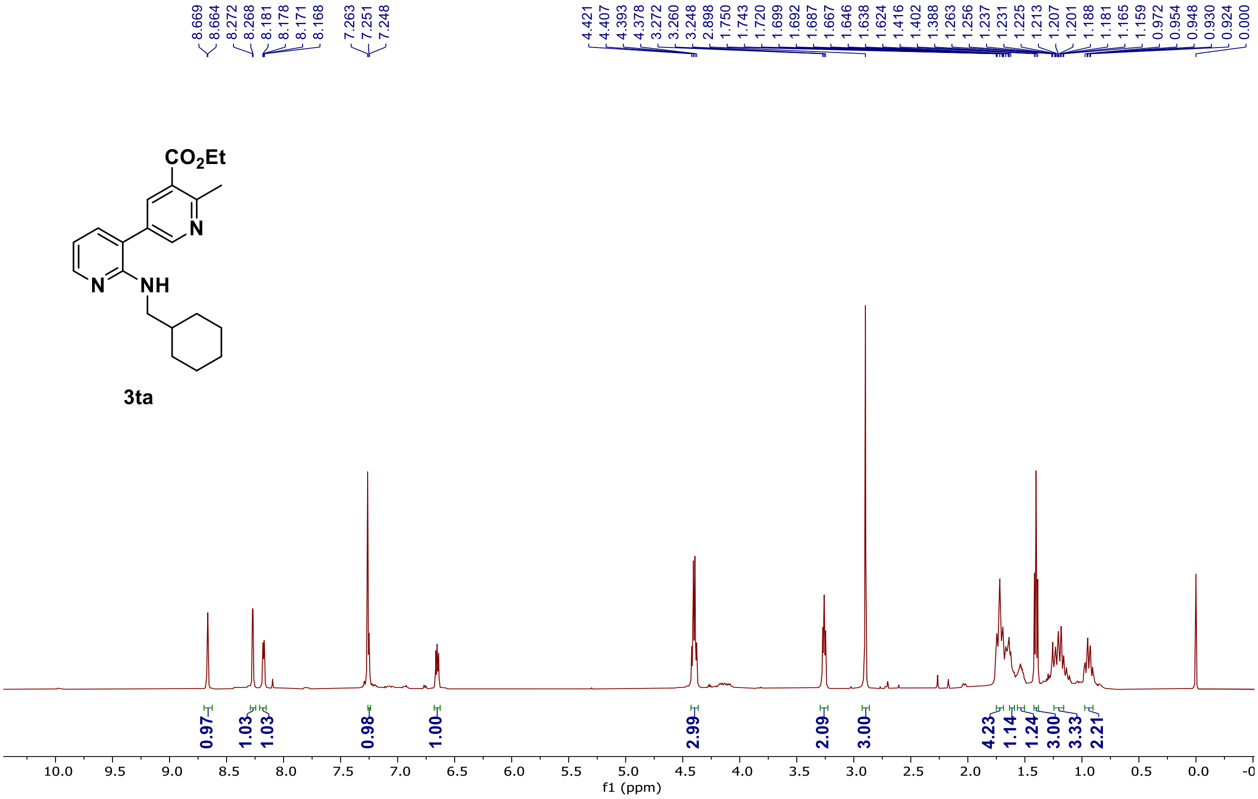

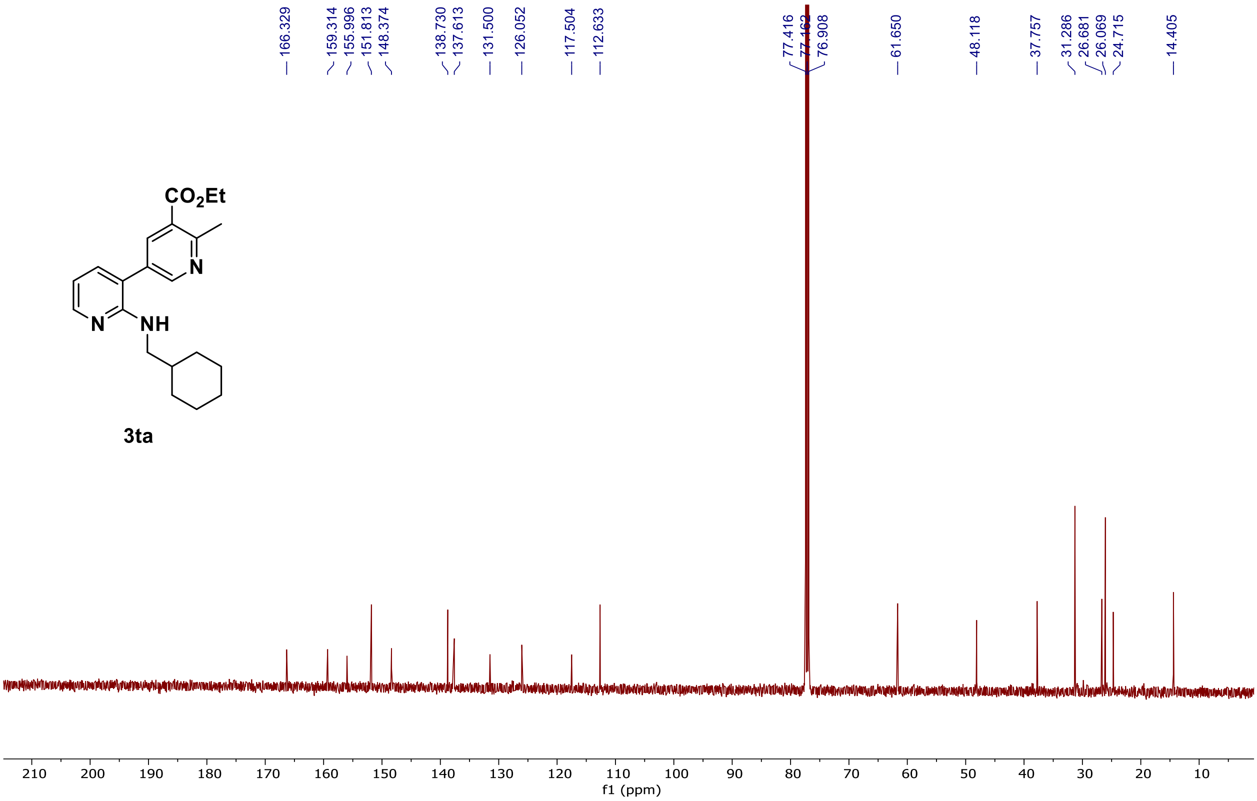

^1H and ^{13}C NMR Spectrum of **3ta** in $CDCl_3$

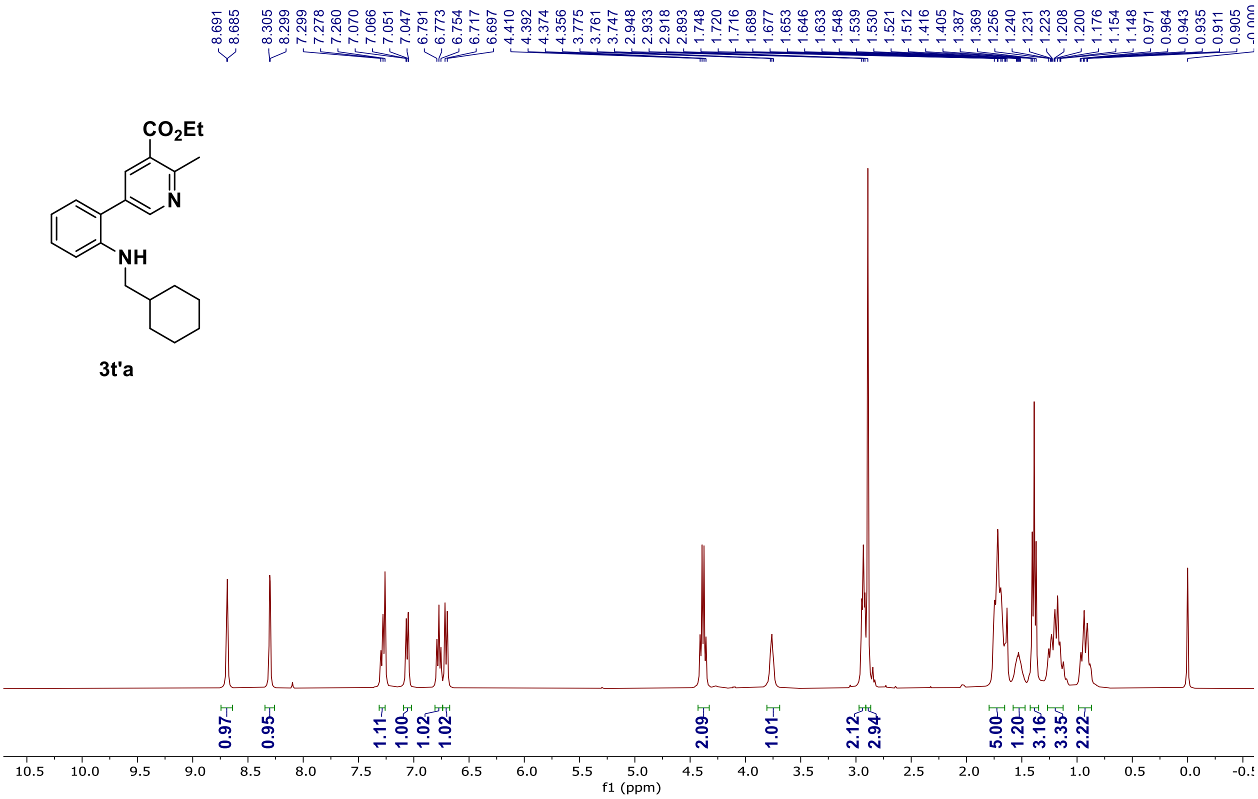

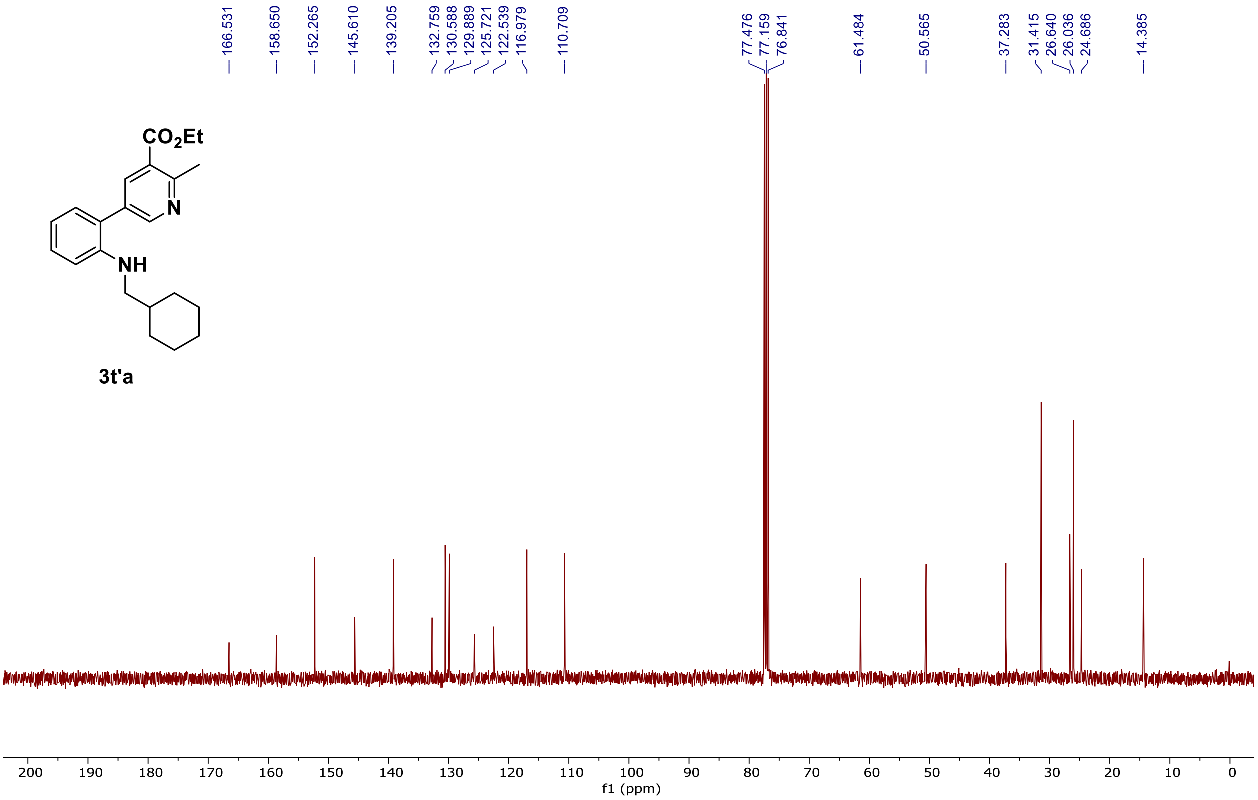

^{1}H and ^{13}C NMR Spectrum of **3t'a** in $CDCl_3$

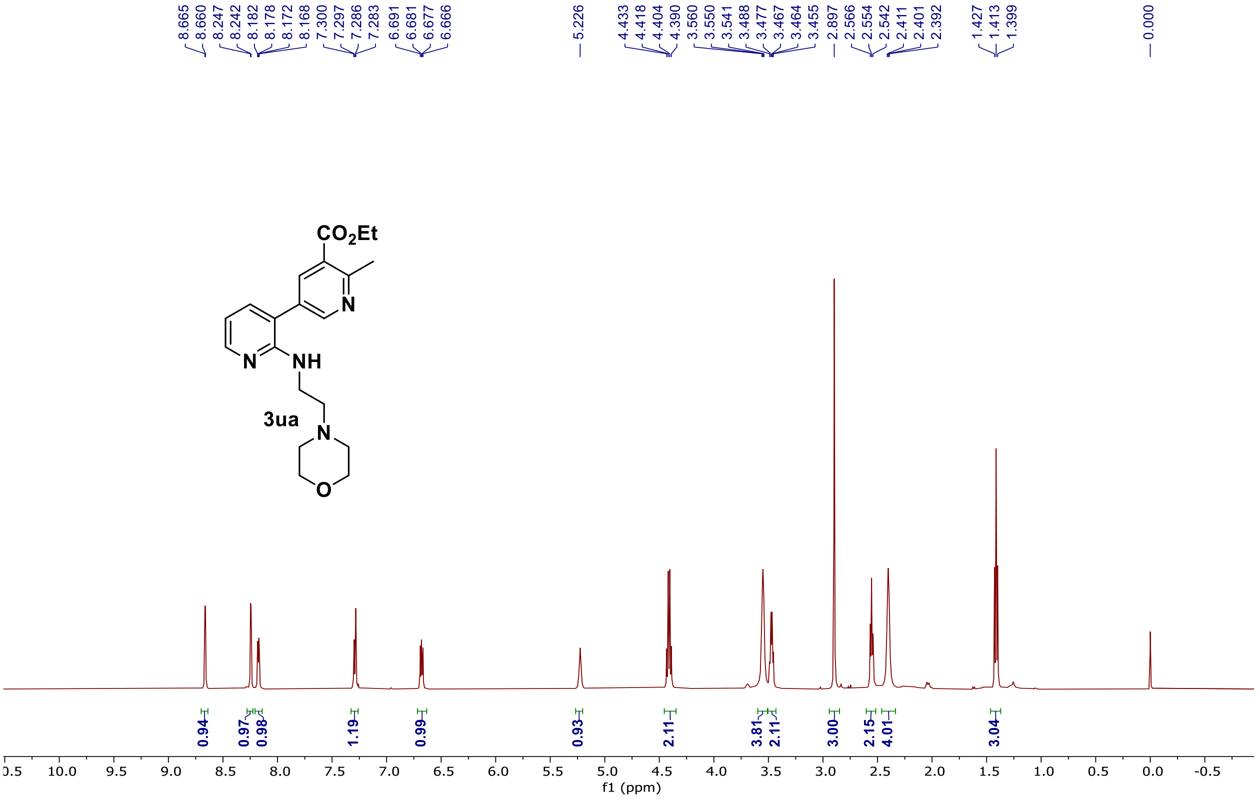

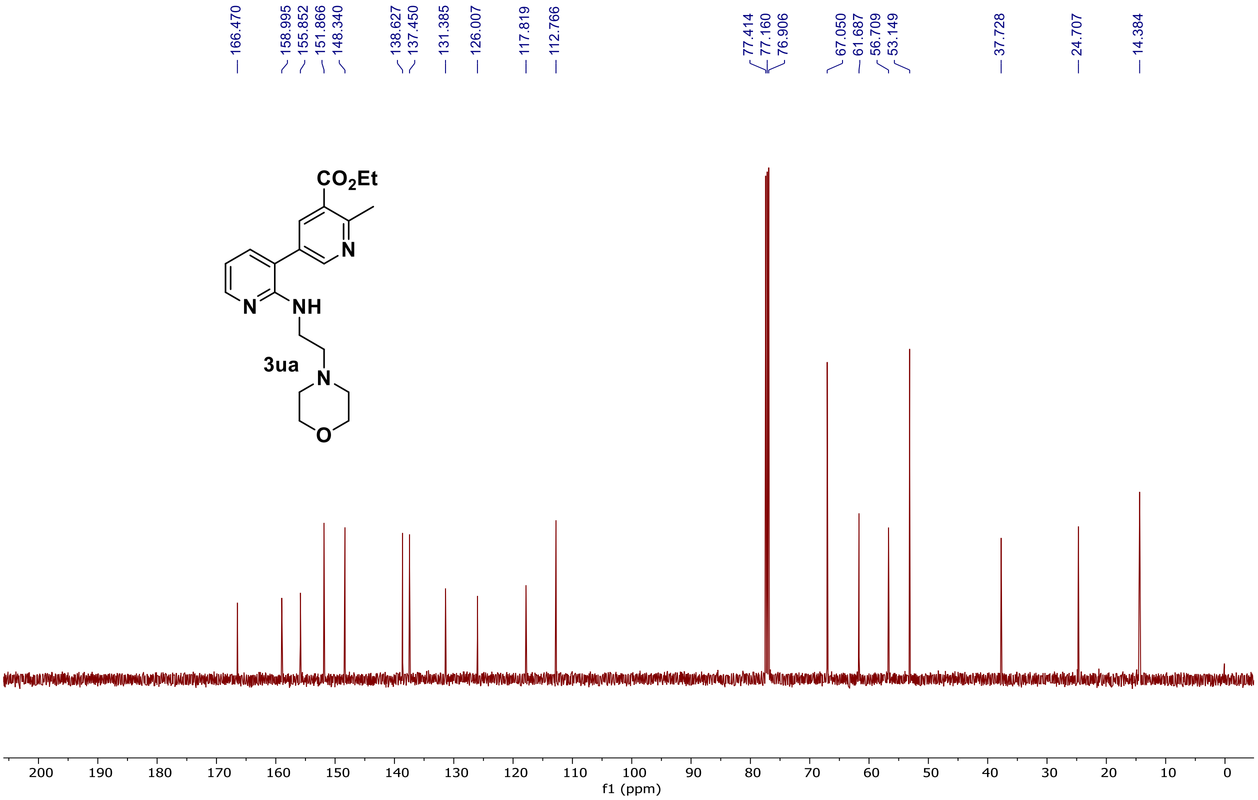

^{1}H and ^{13}C NMR Spectrum of **3ua** in $CDCl_3$

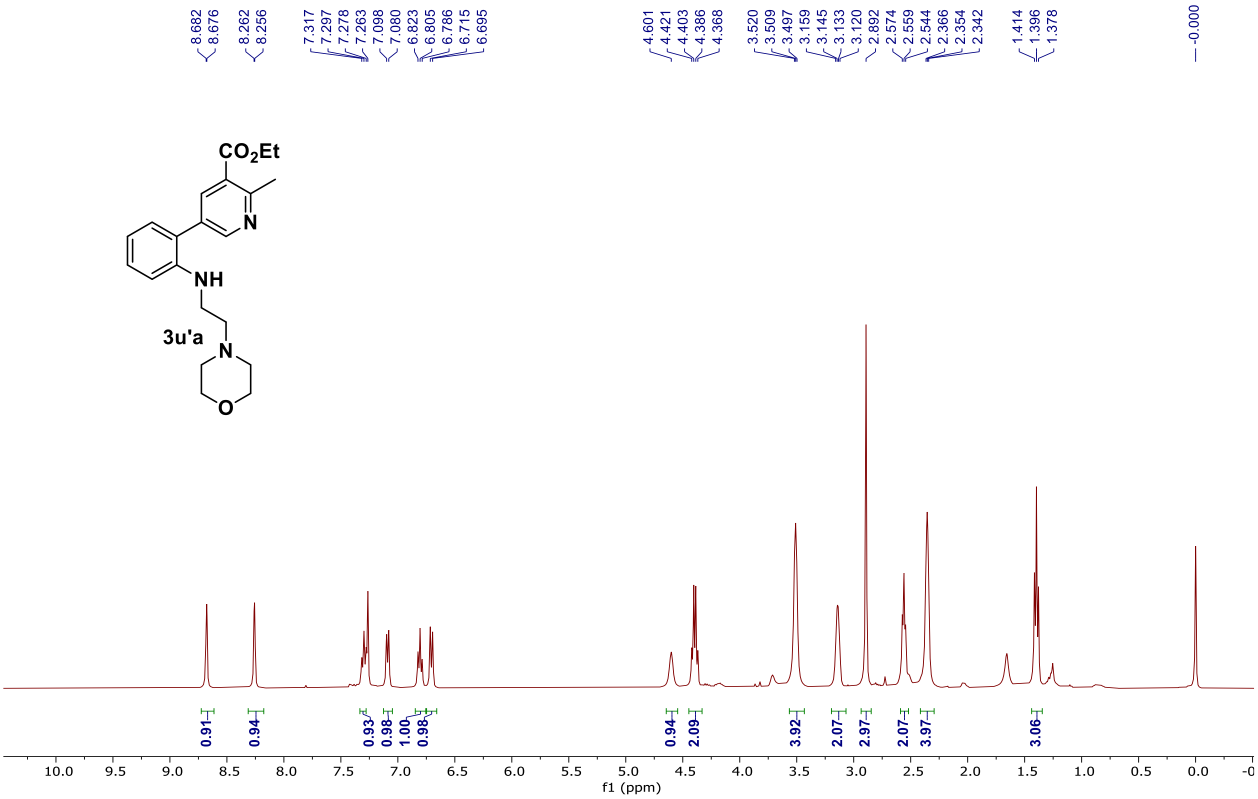

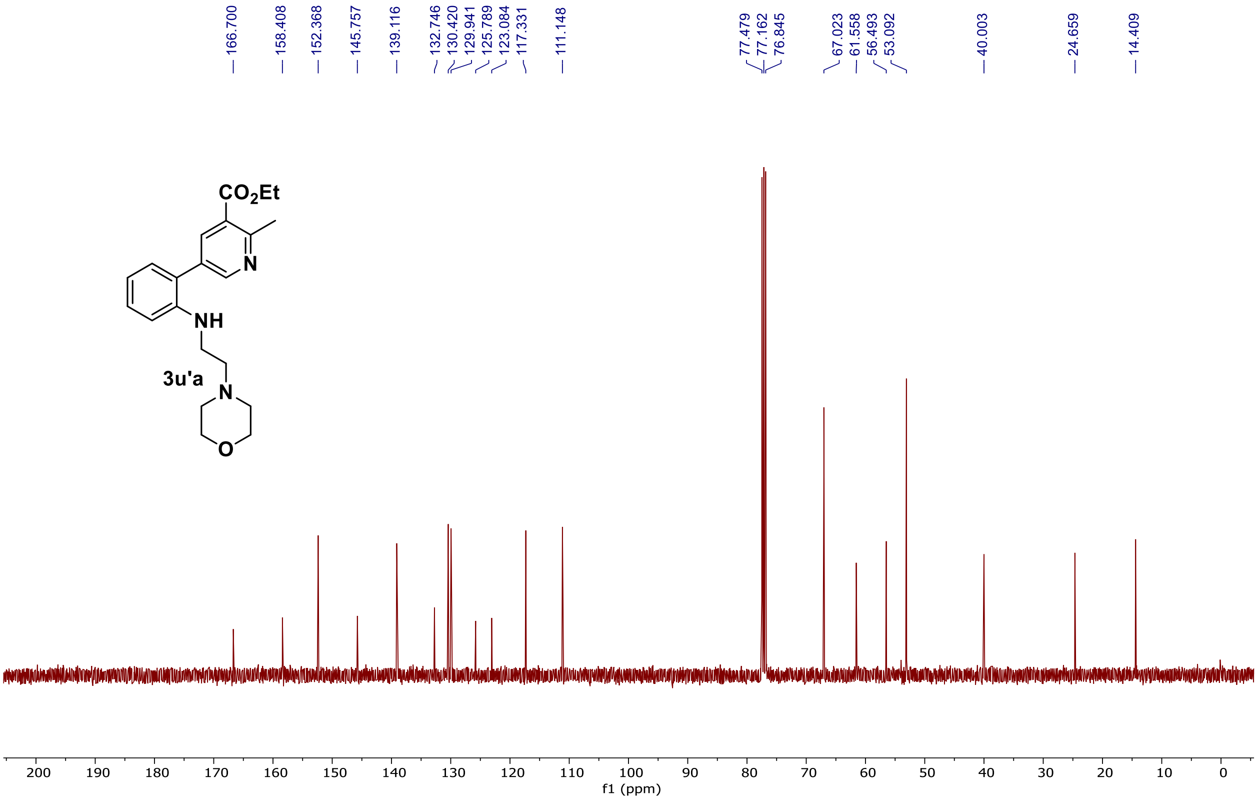

^{1}H and ^{13}C NMR Spectrum of **3u'a** in $CDCl_3$

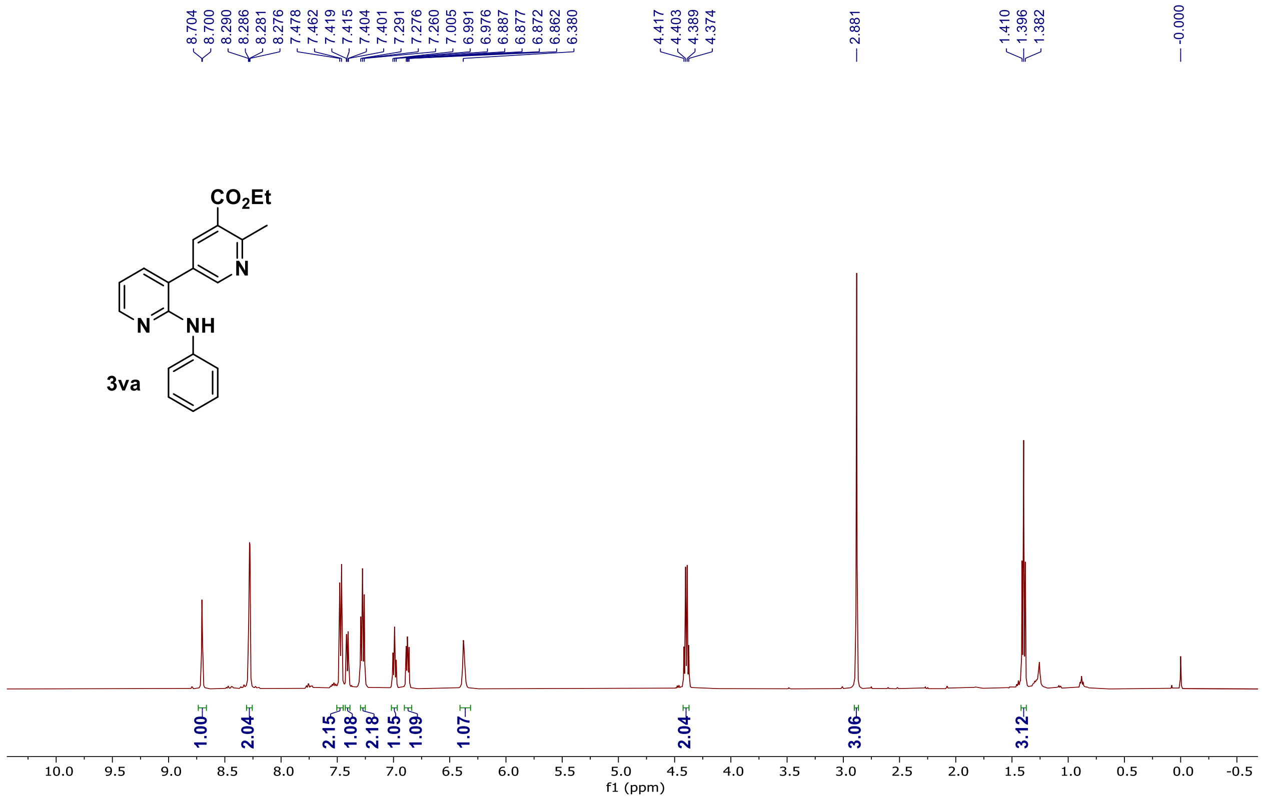

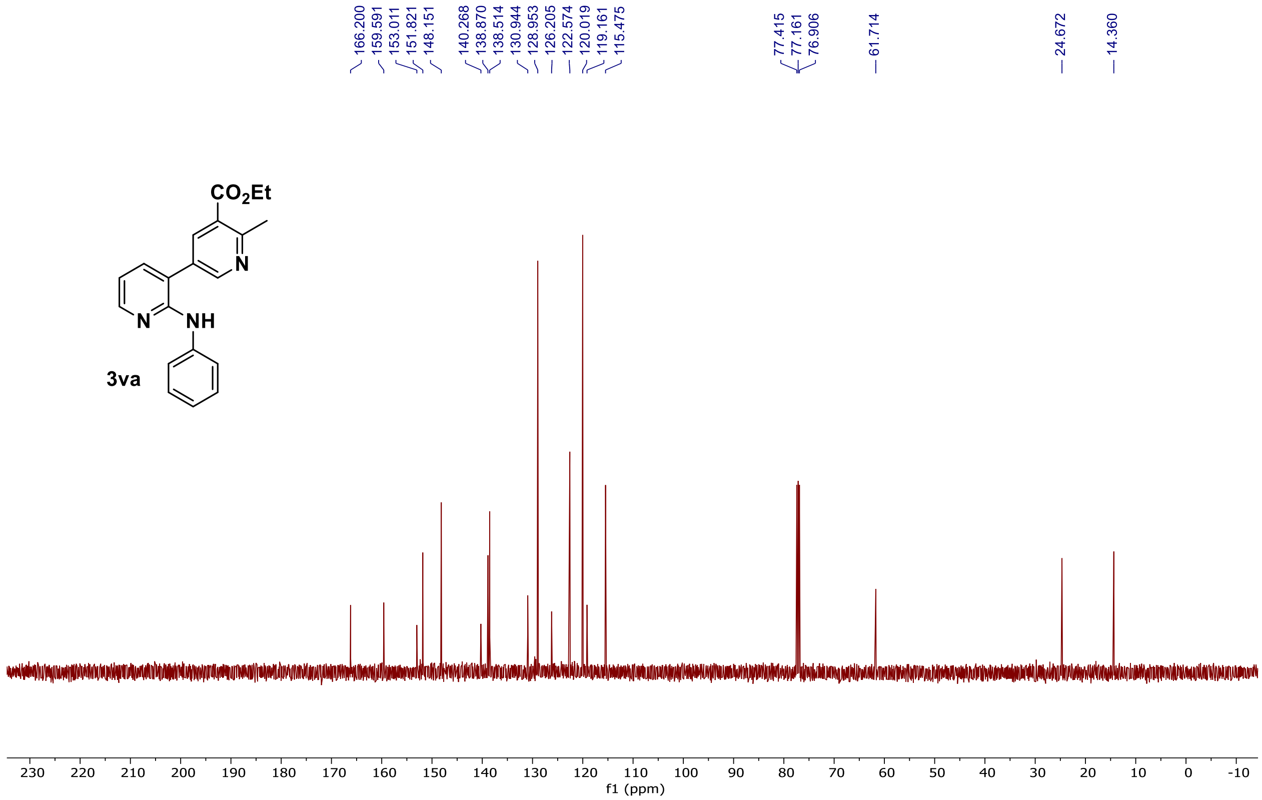

^{1}H and ^{13}C NMR Spectrum of **3va** in CDCl$_3$

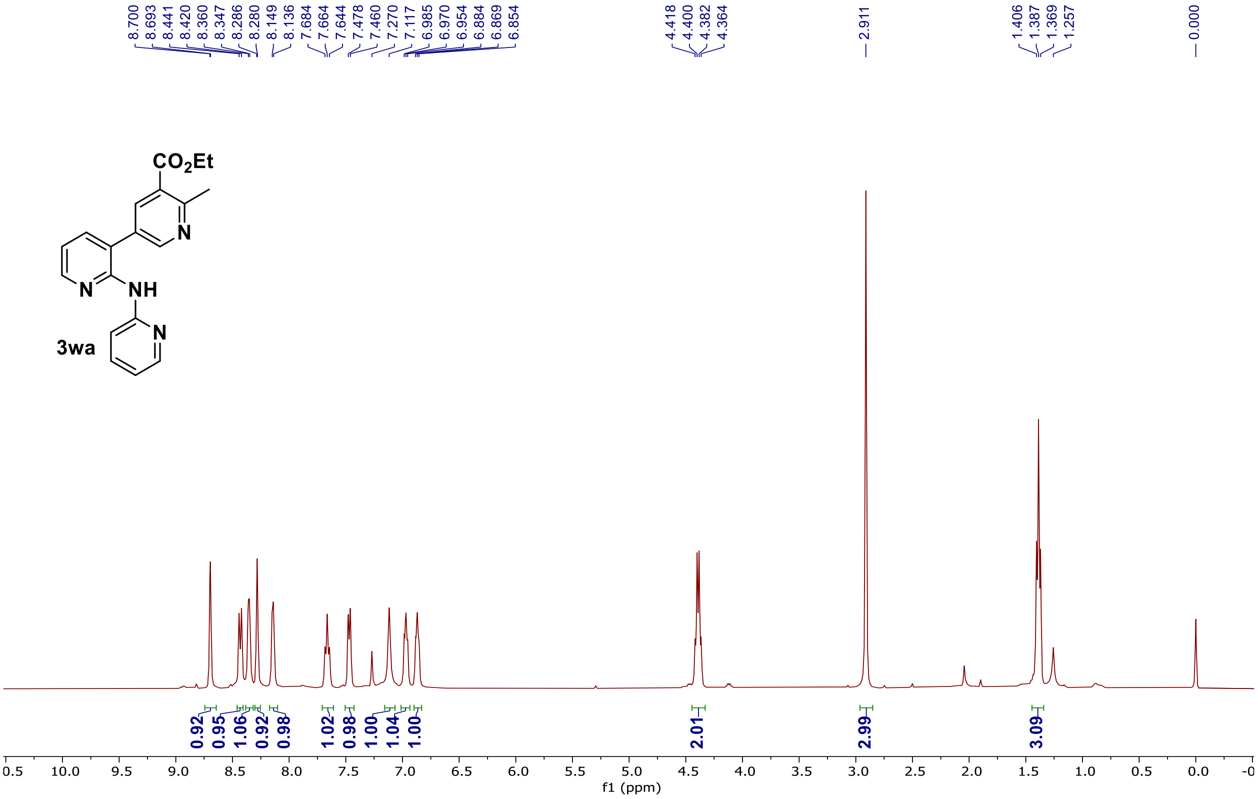

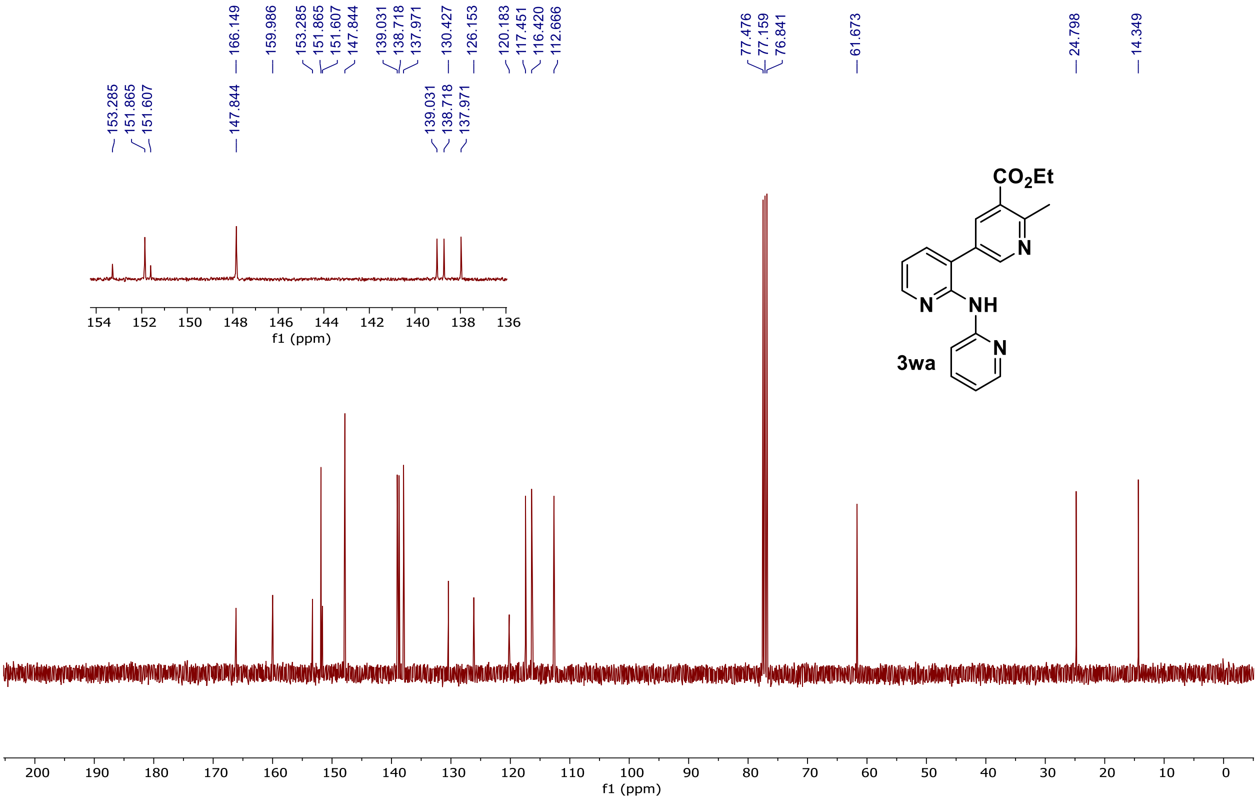

^{1}H and ^{13}C NMR Spectrum of **3wa** in $CDCl_3$

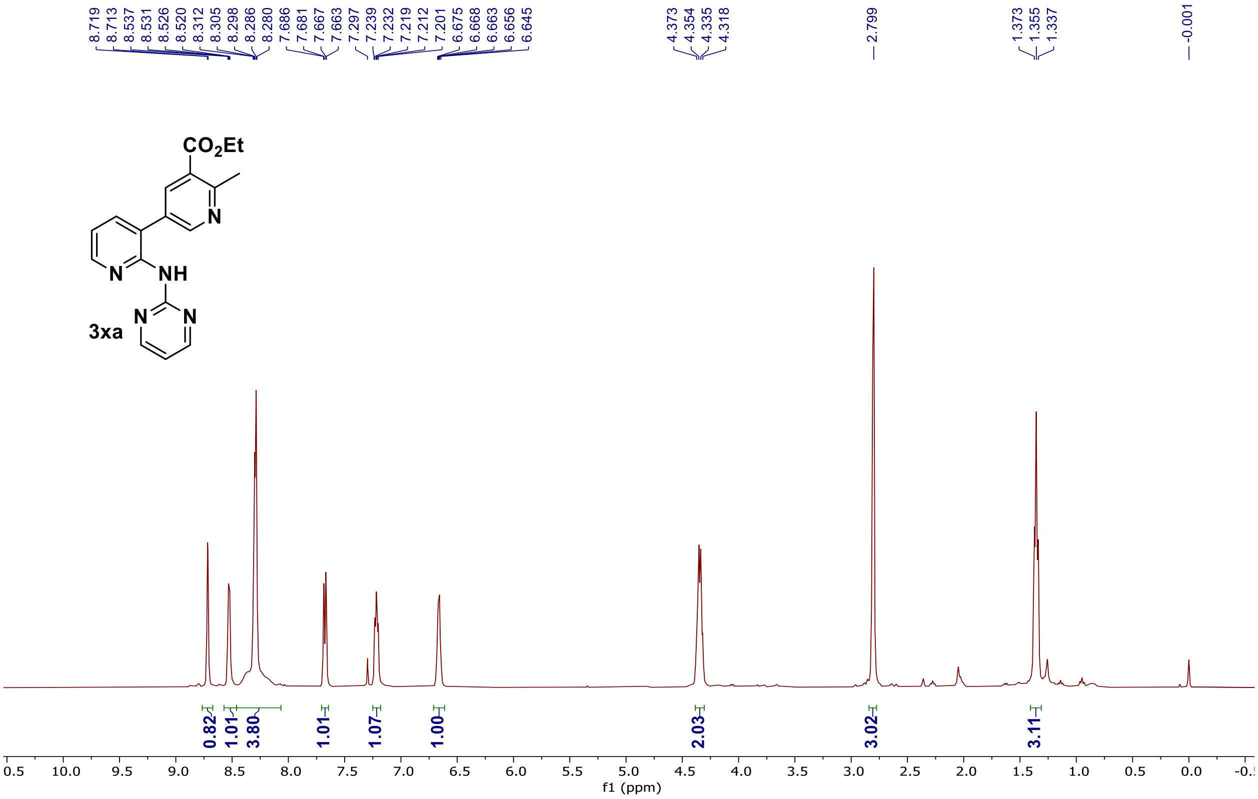

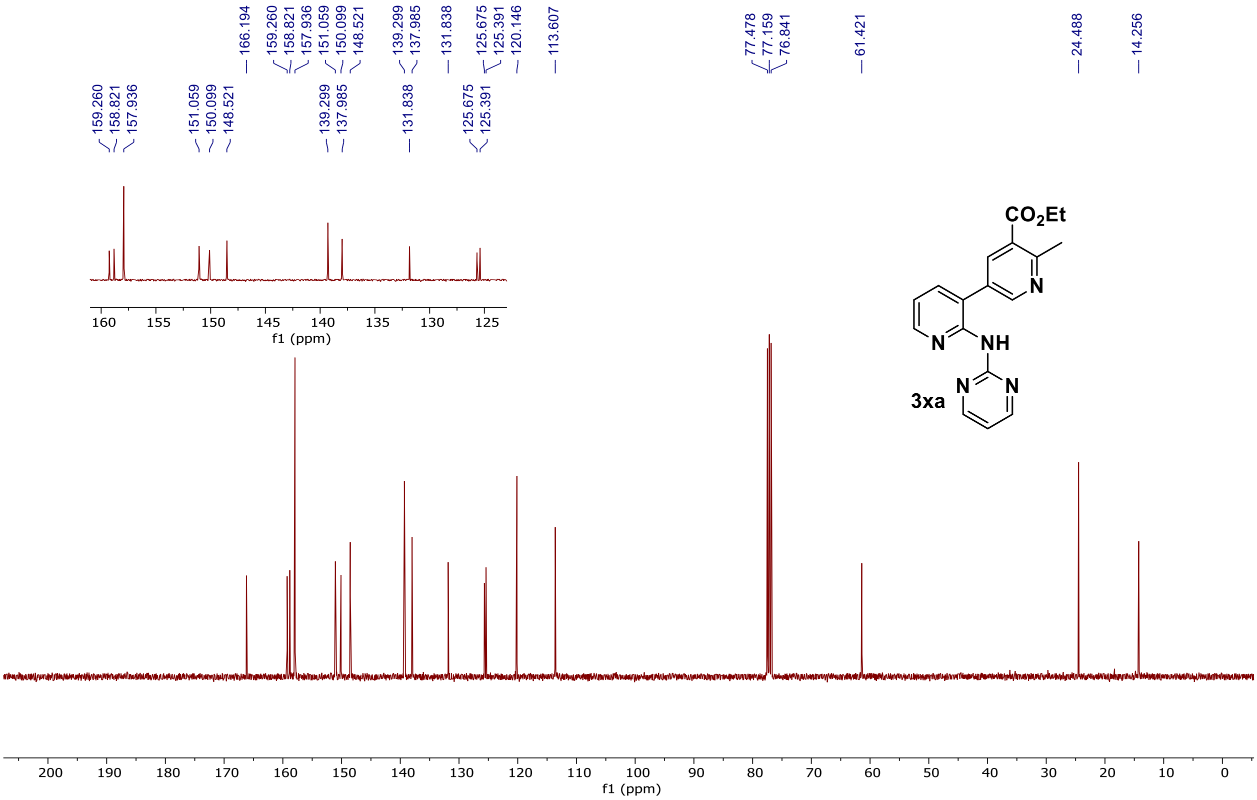

^{1}H and ^{13}C NMR Spectrum of **3xa** in $CDCl_3$

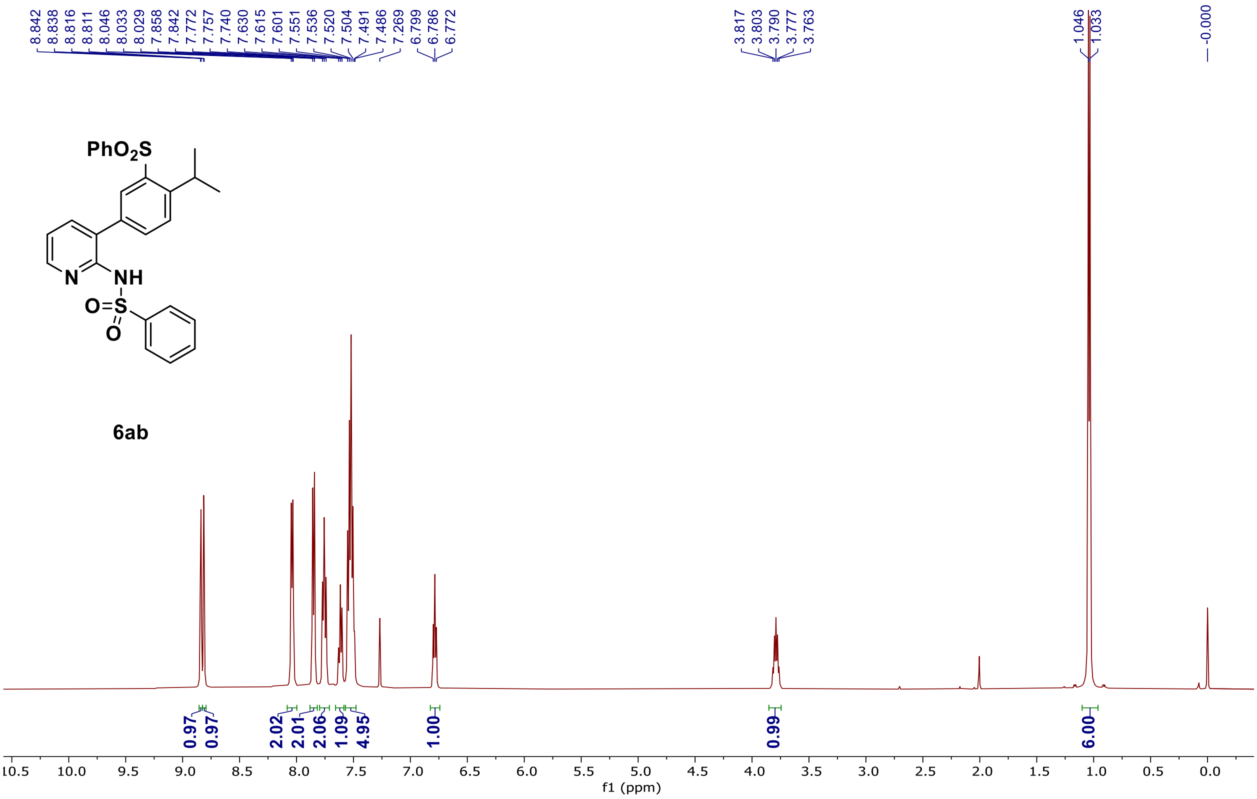

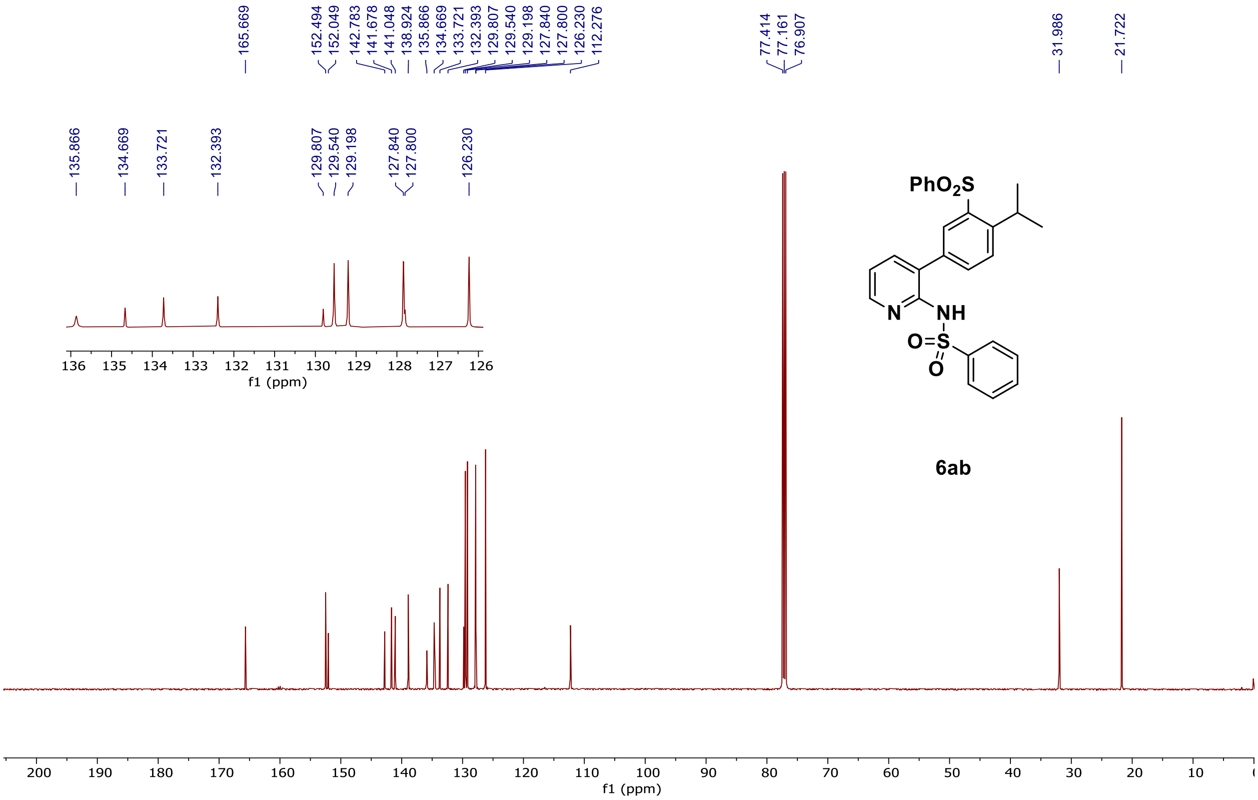

^{1}H and ^{13}C NMR Spectrum of **6ab** in $CDCl_3$

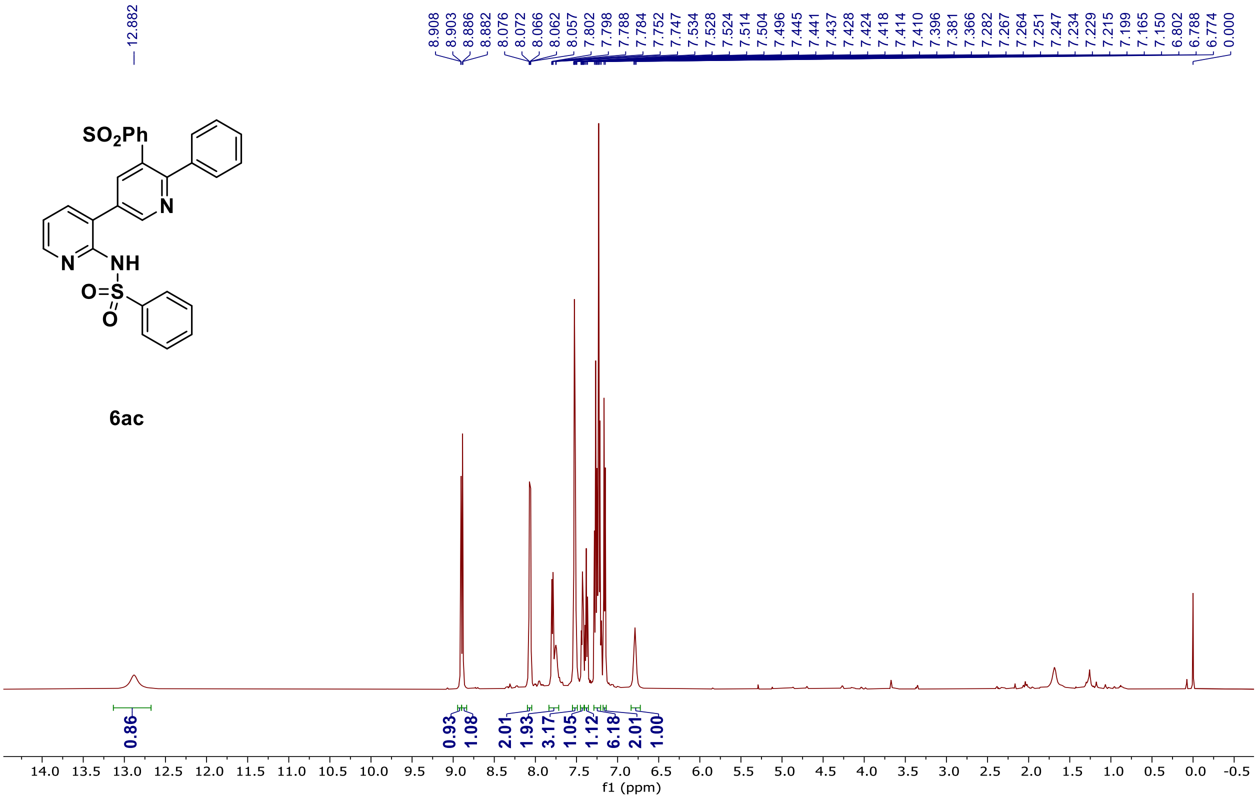

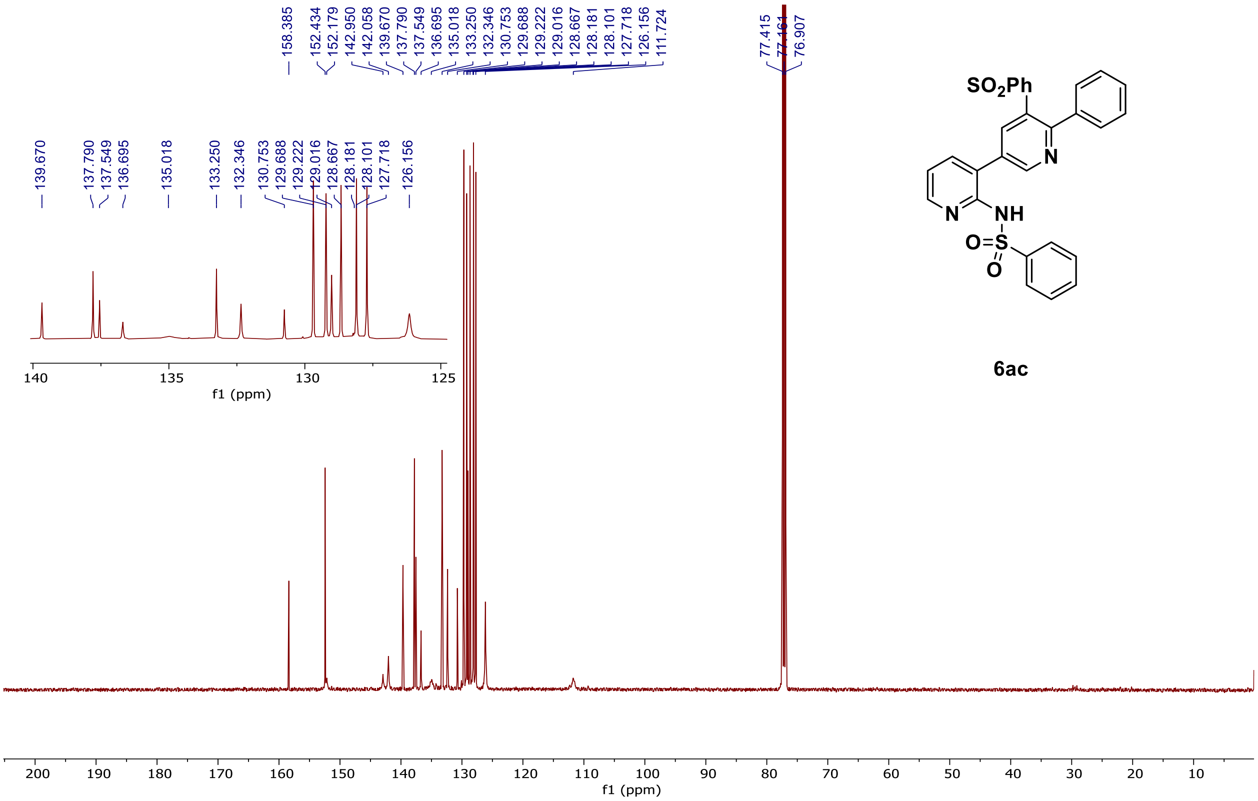

^{1}H and ^{13}C NMR Spectrum of **6ac** in $CDCl_3$

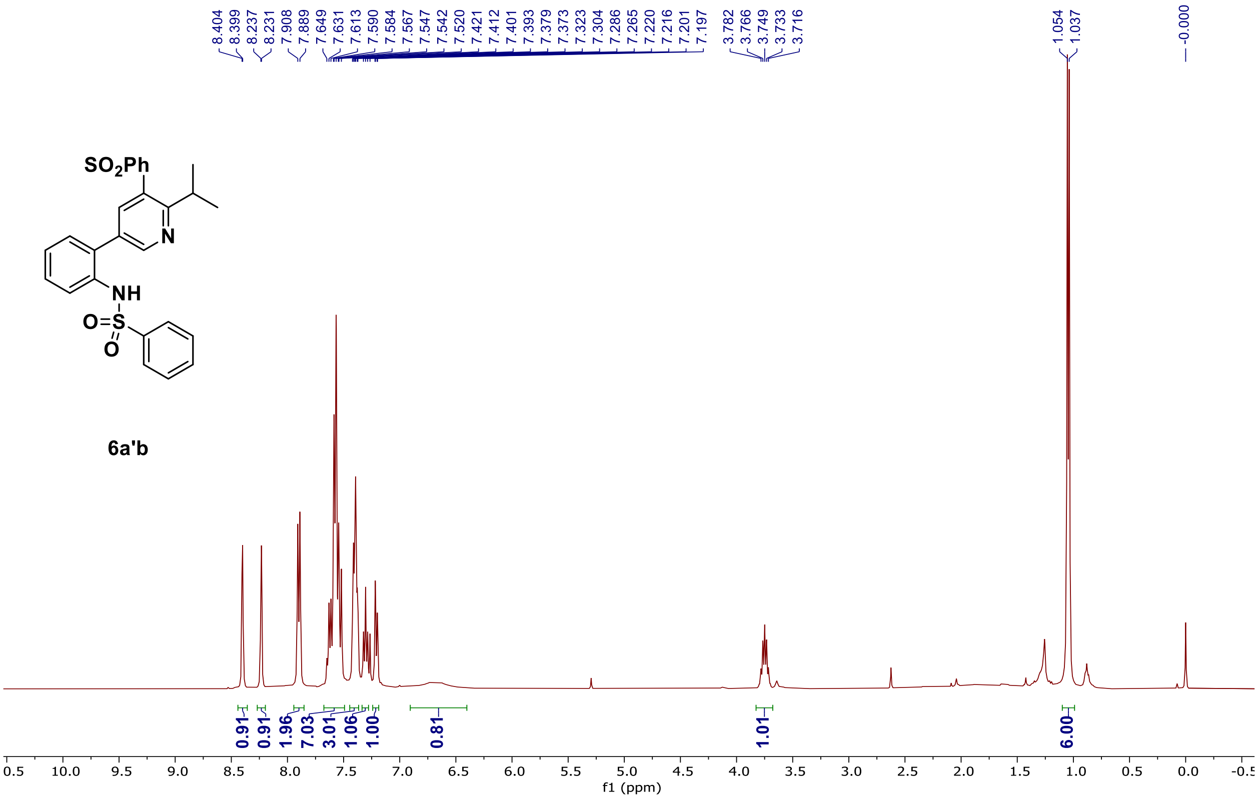

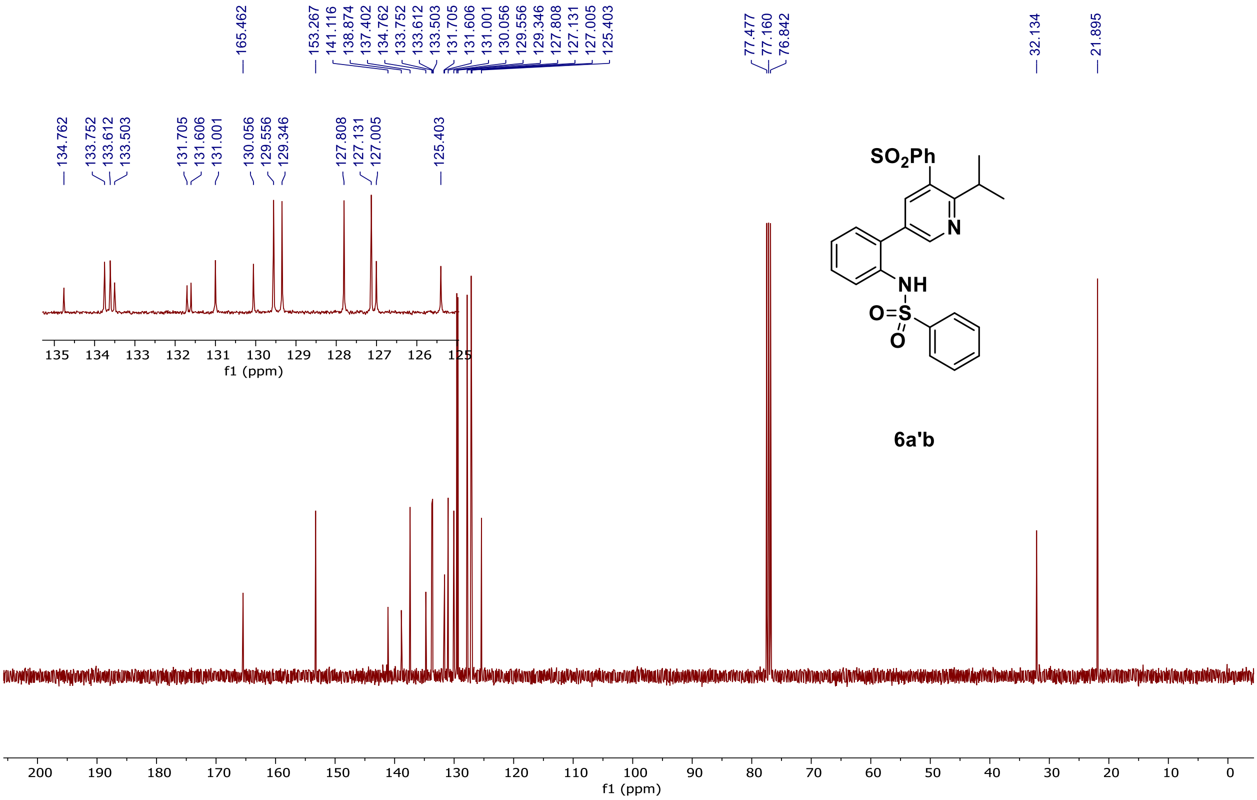

^{1}H and ^{13}C NMR Spectrum of **6a'b** in $CDCl_3$

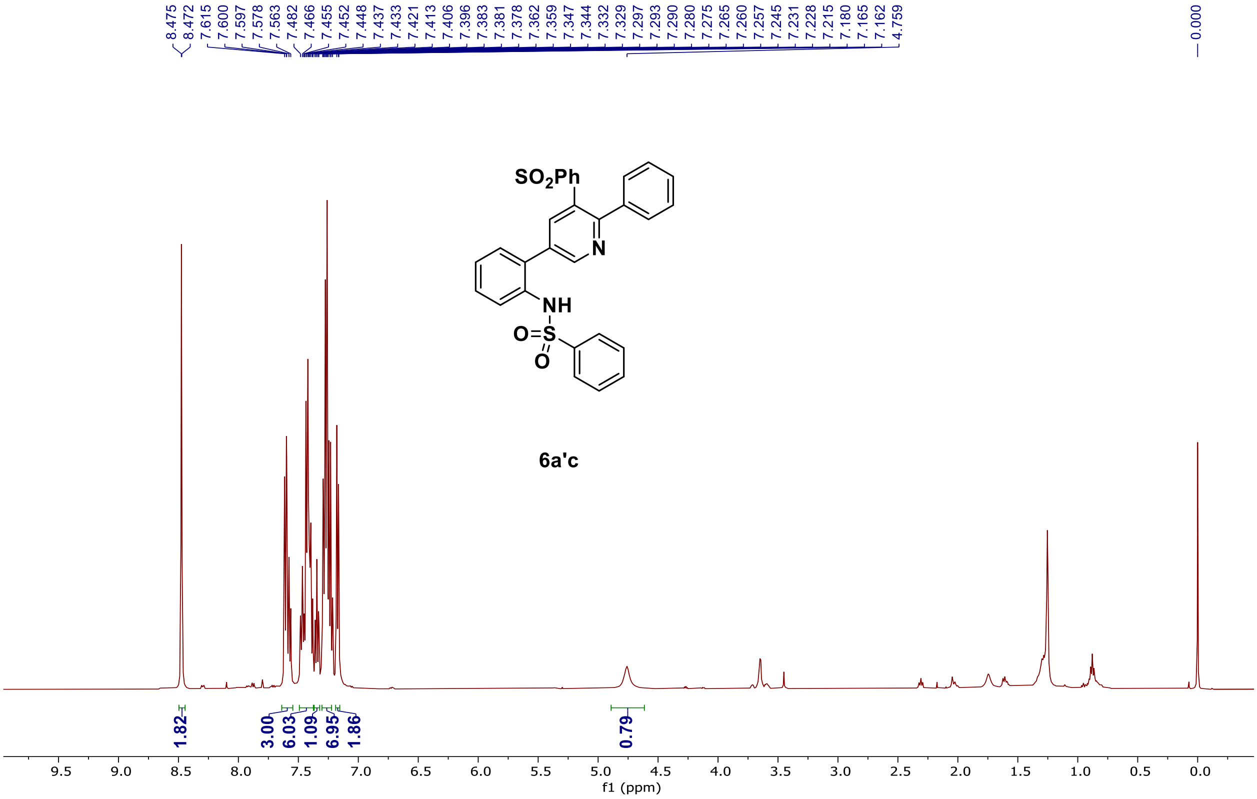

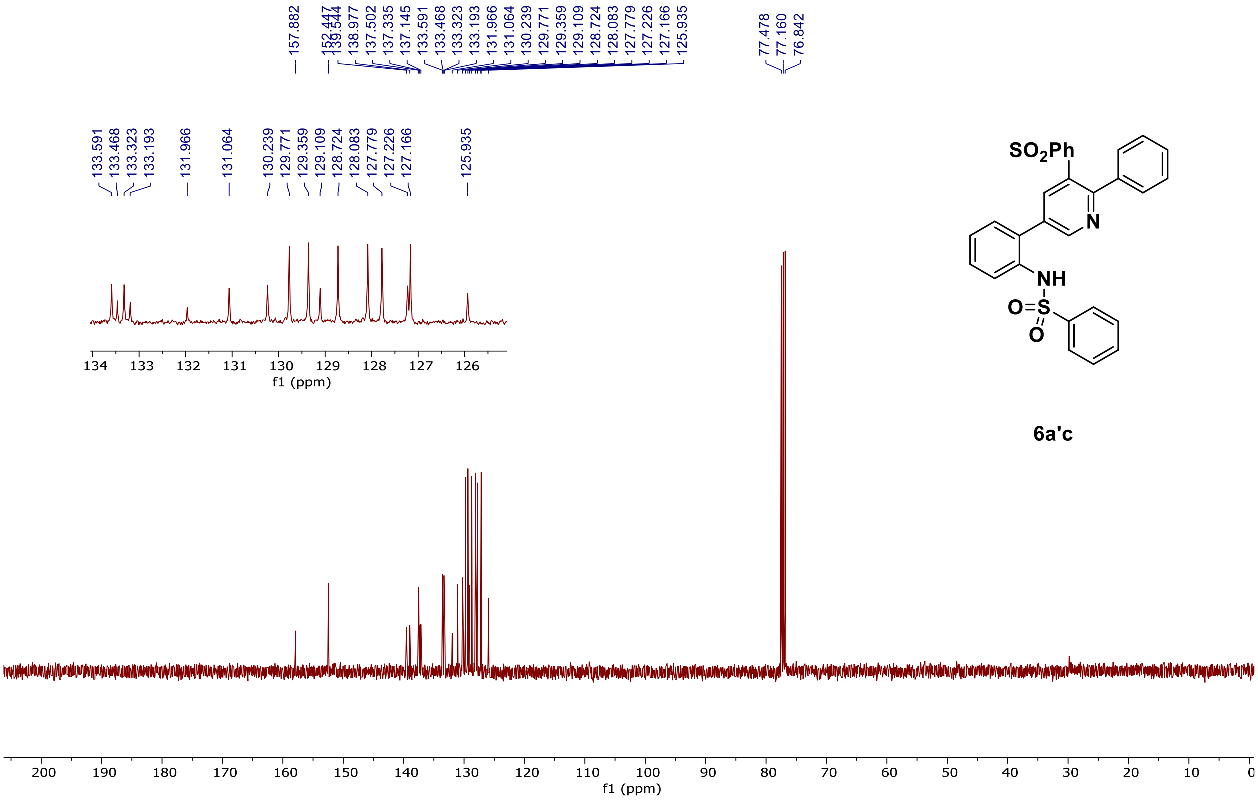

^{1}H and ^{13}C NMR Spectrum of **6a'c** in $CDCl_3$

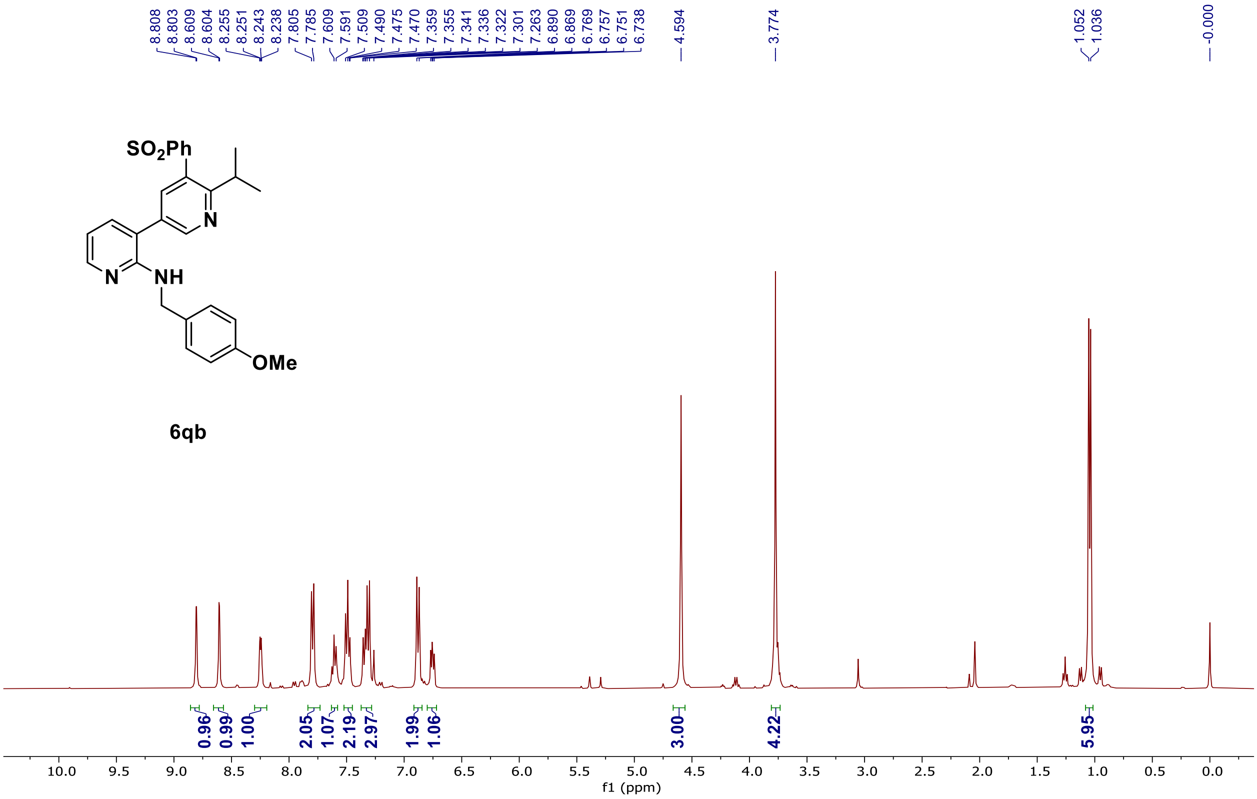

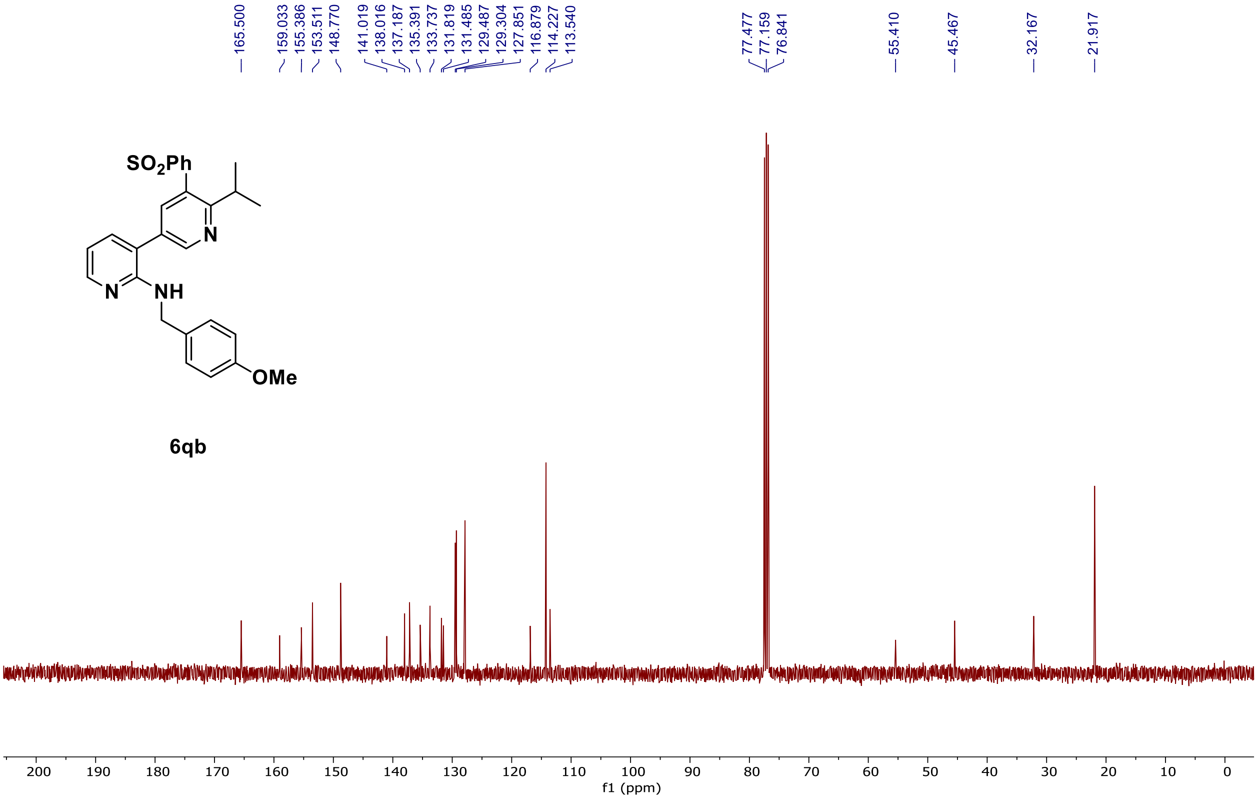

^{1}H and ^{13}C NMR Spectrum of **6qb** in $CDCl_3$

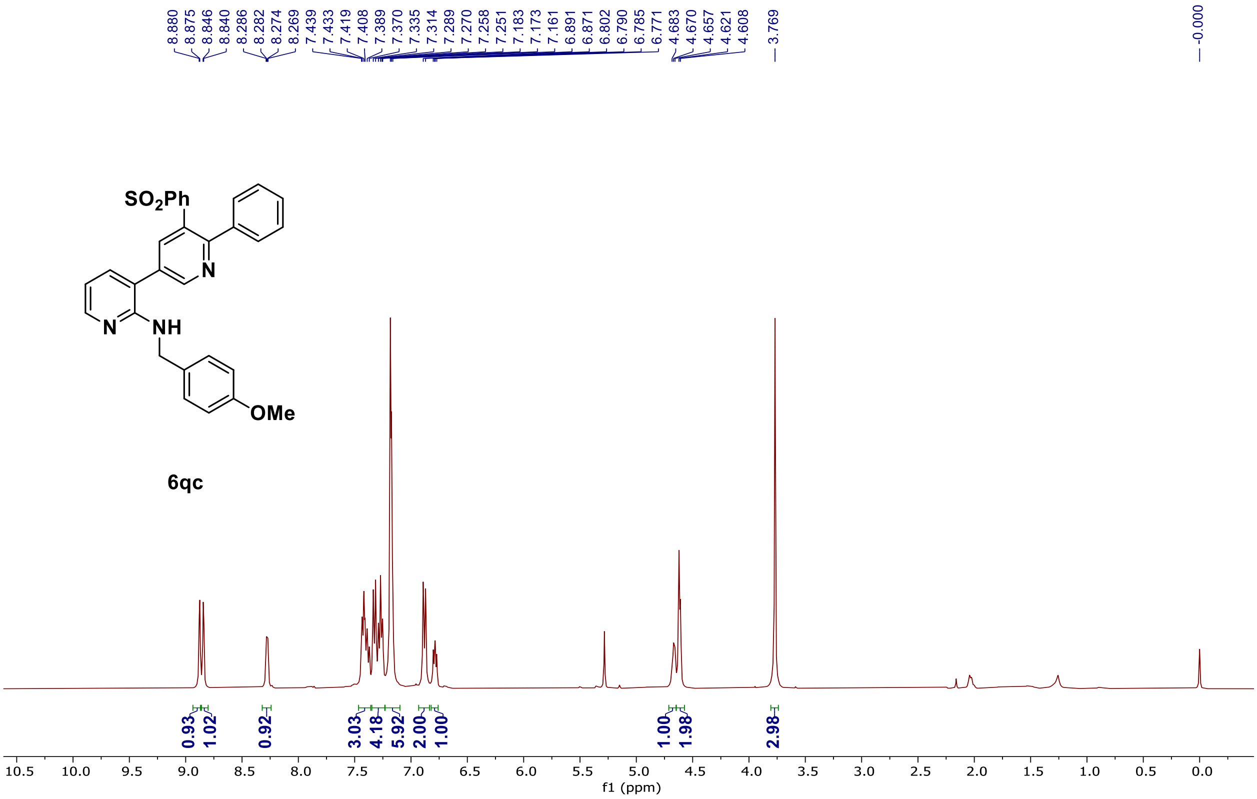

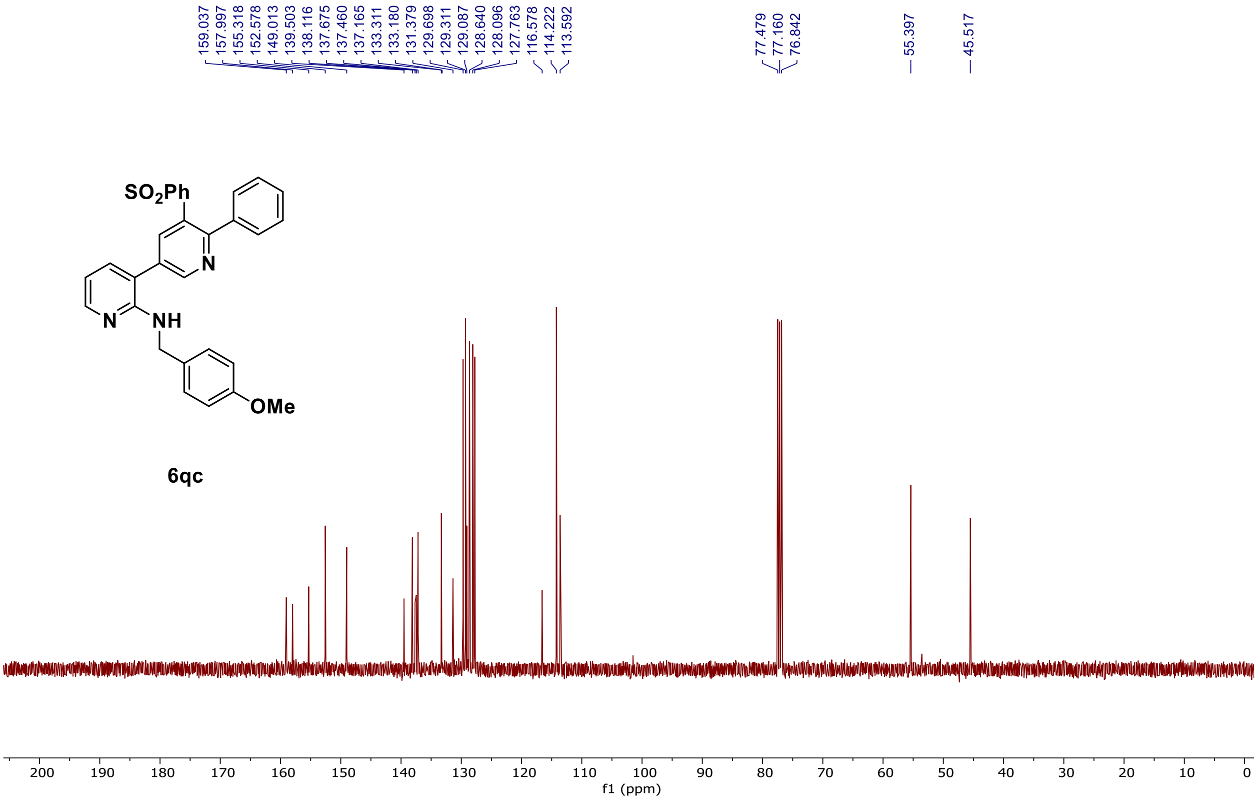

^{1}H and ^{13}C NMR Spectrum of **6qc** in $CDCl_3$

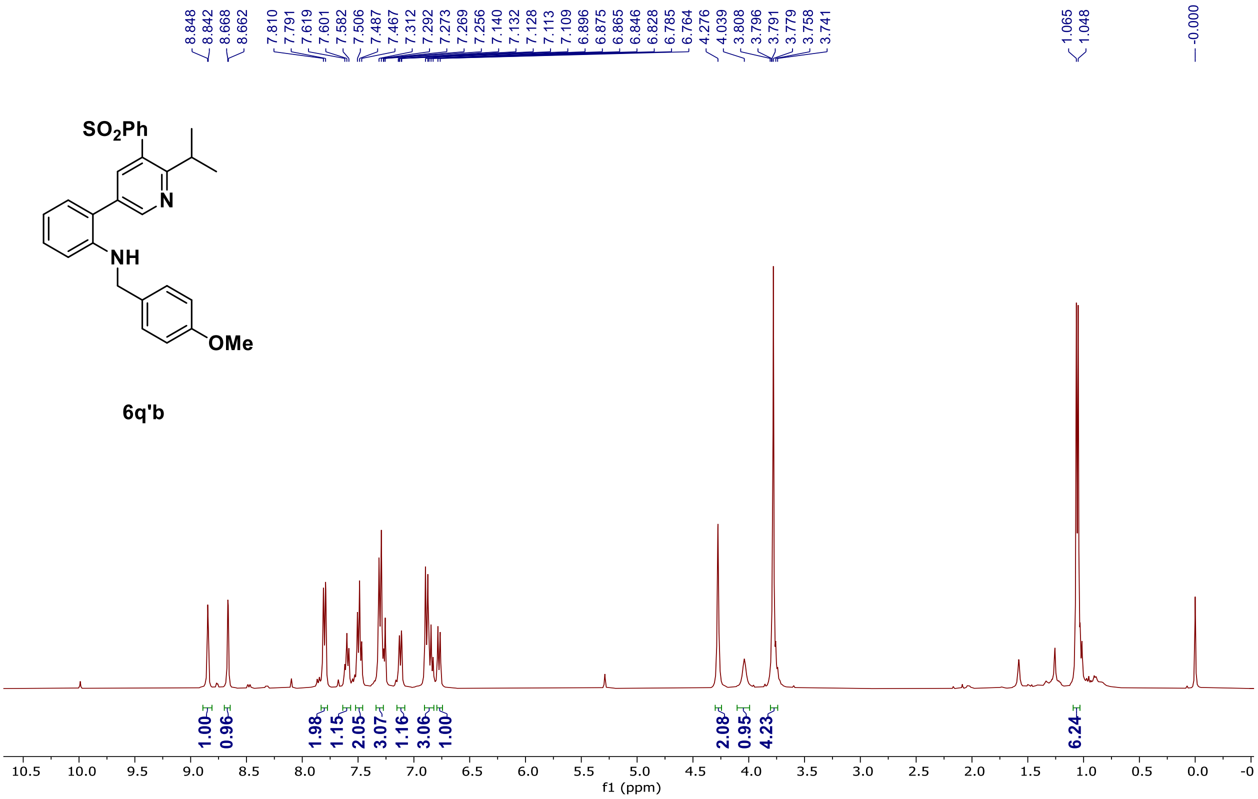

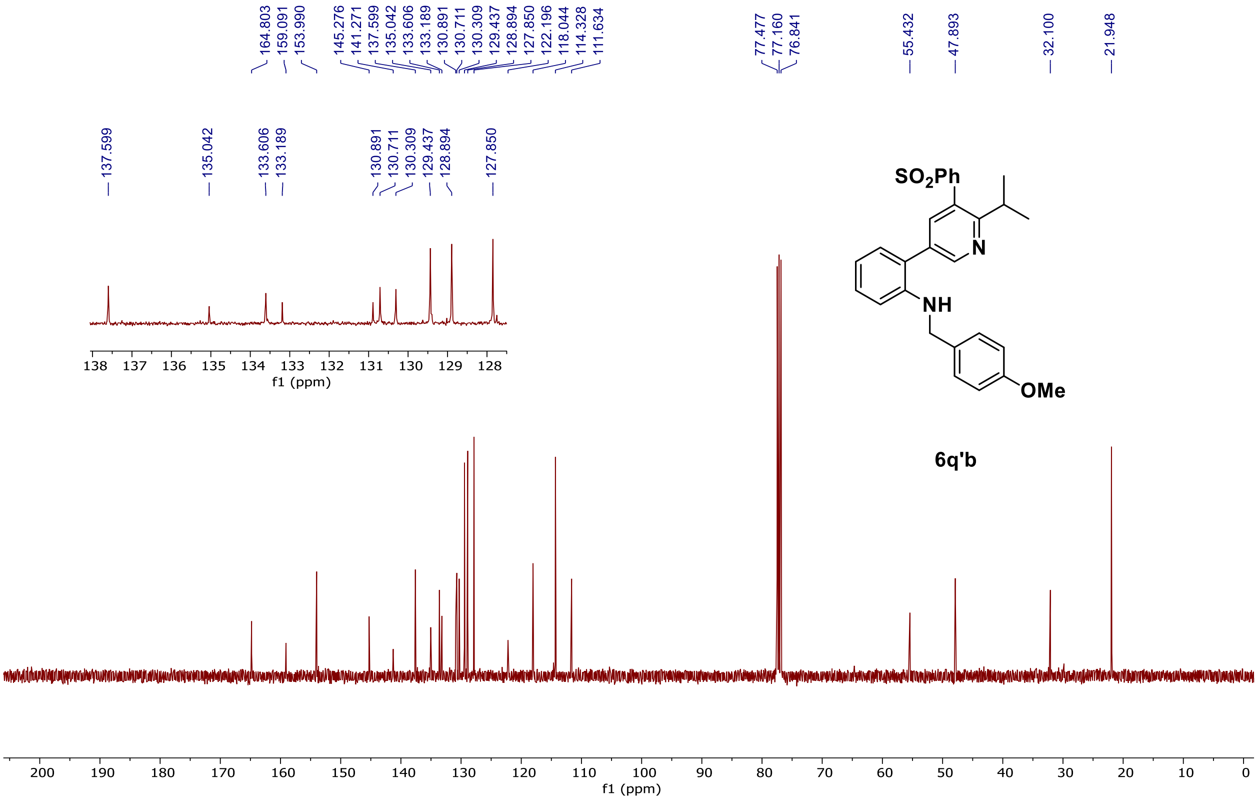

^{1}H and ^{13}C NMR Spectrum of **6q'b** in $CDCl_3$

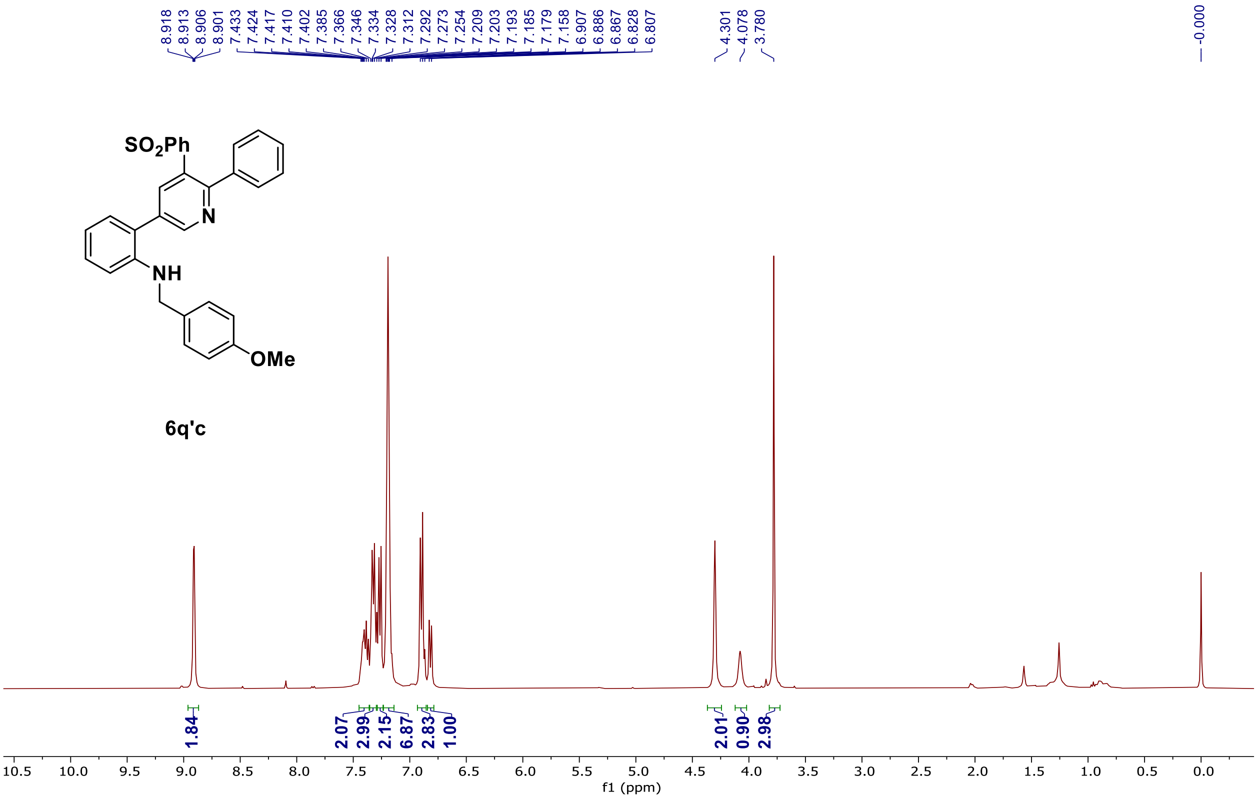

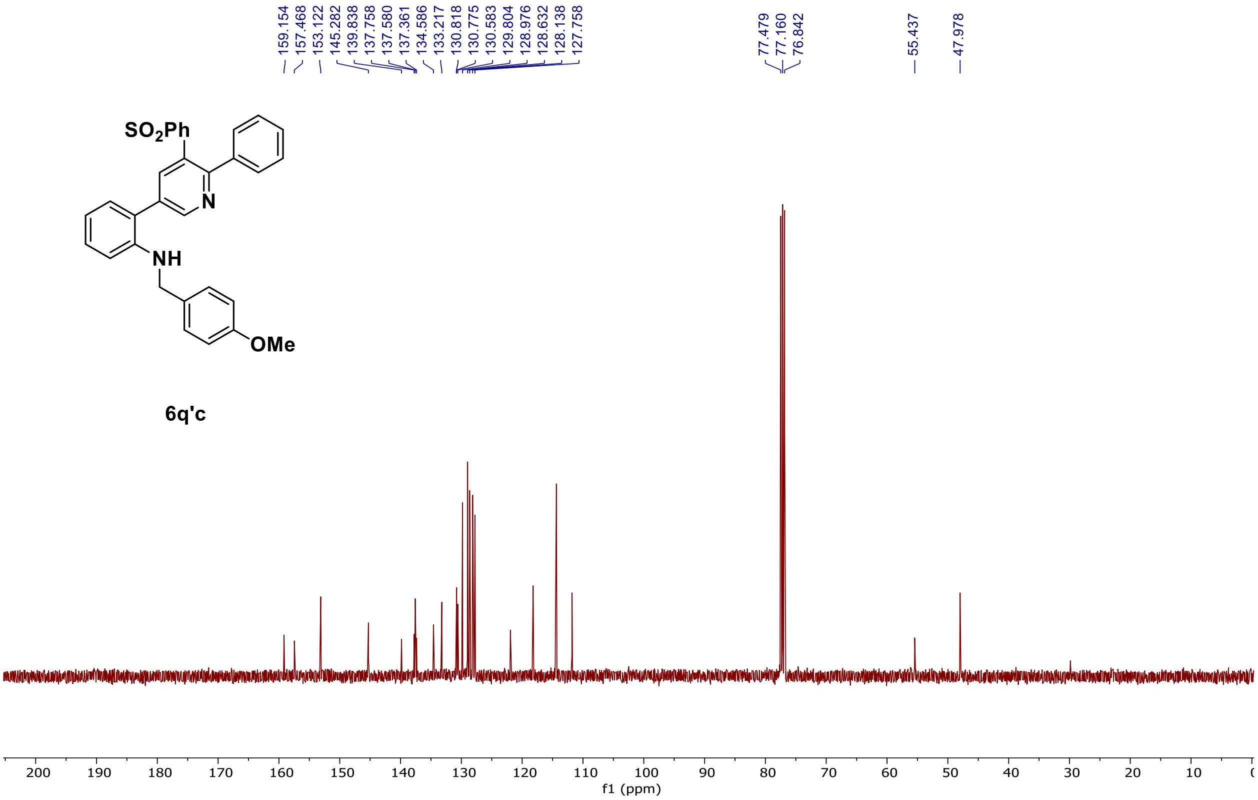

^{1}H and ^{13}C NMR Spectrum of **6q'c** in $CDCl_3$

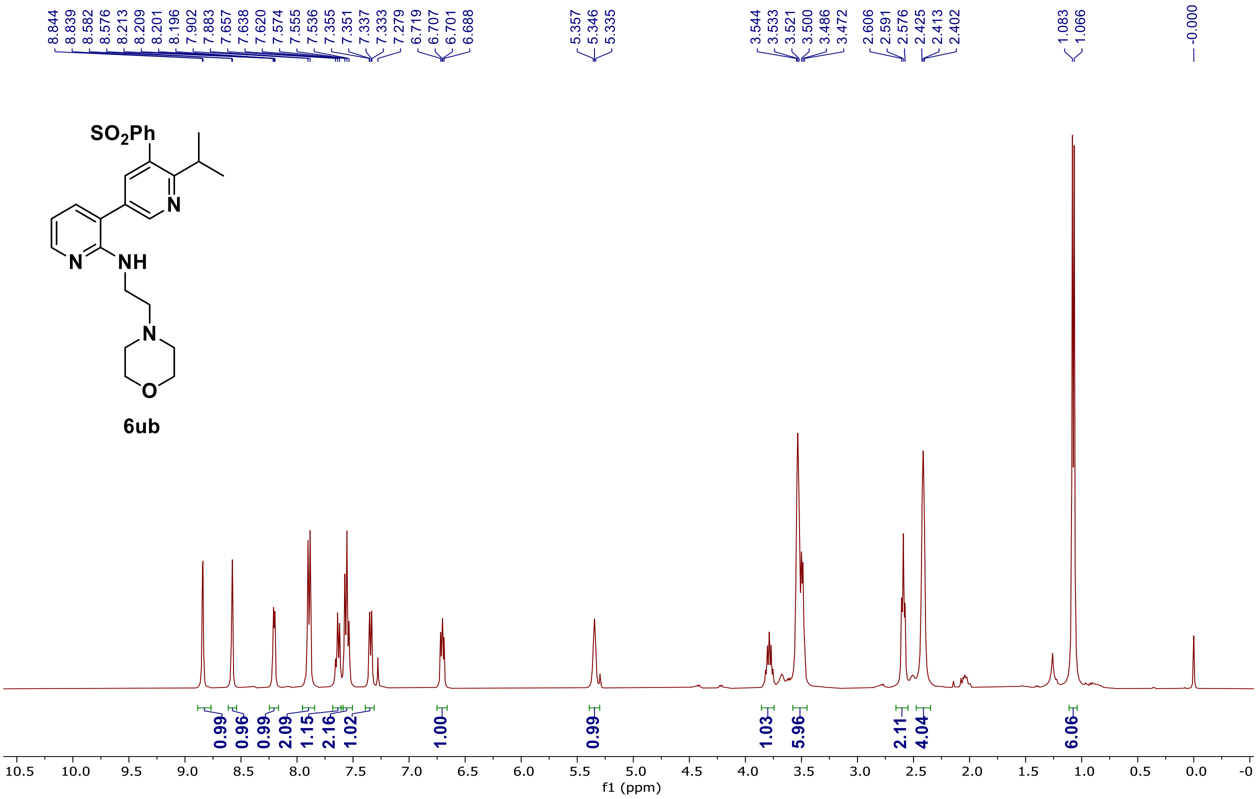

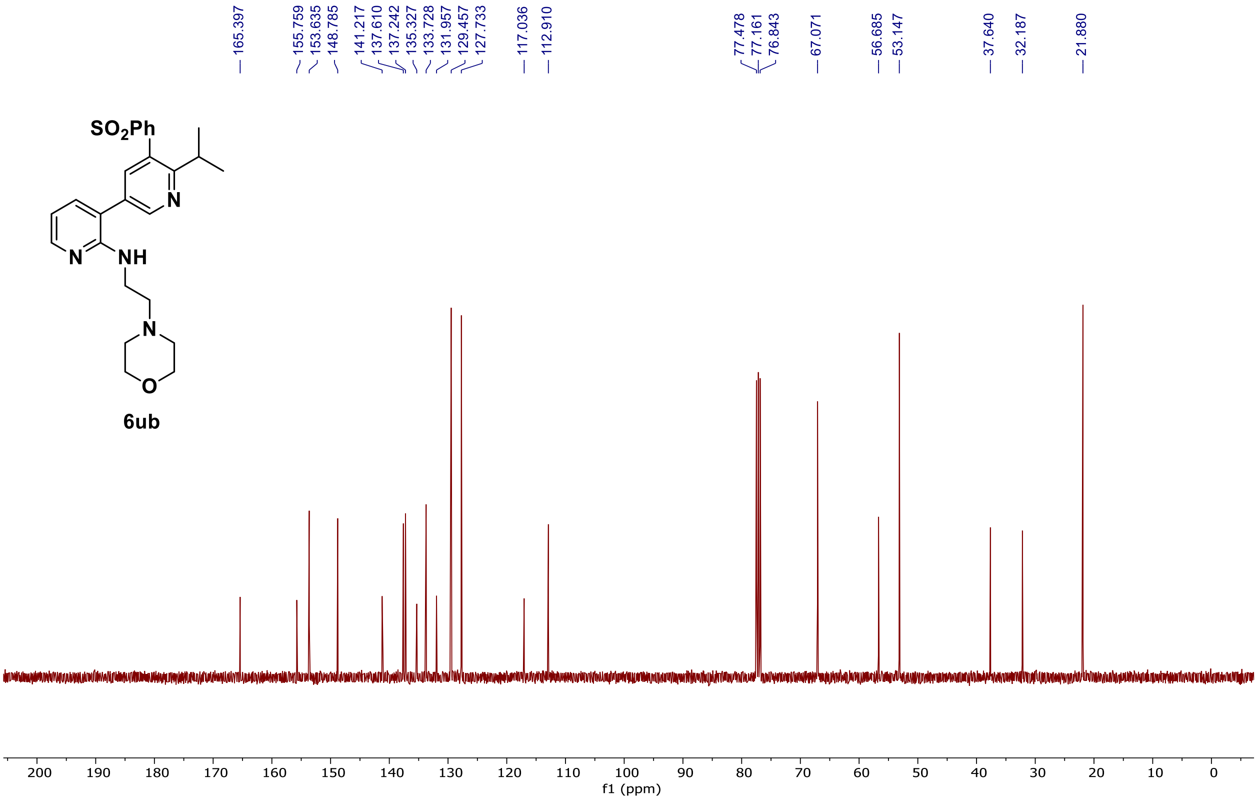

^{1}H and ^{13}C NMR Spectrum of **6ub** in $CDCl_3$

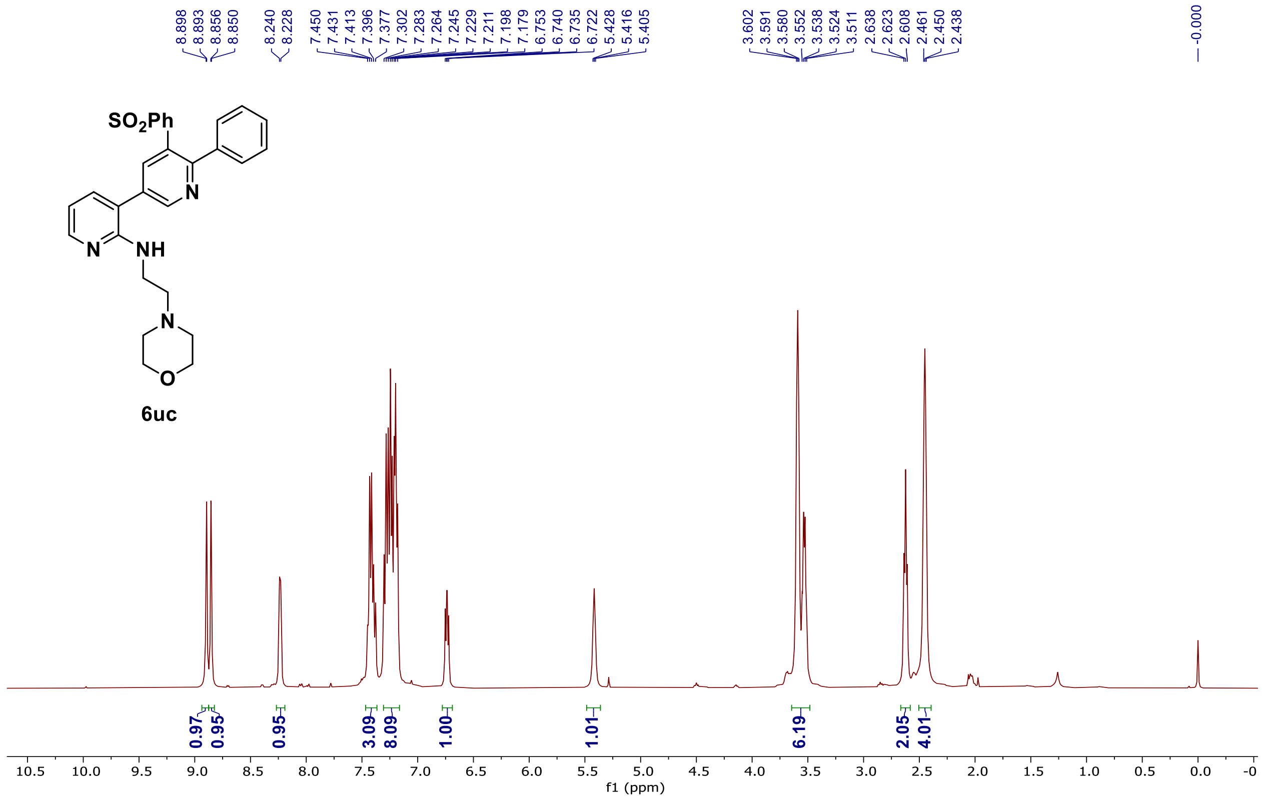

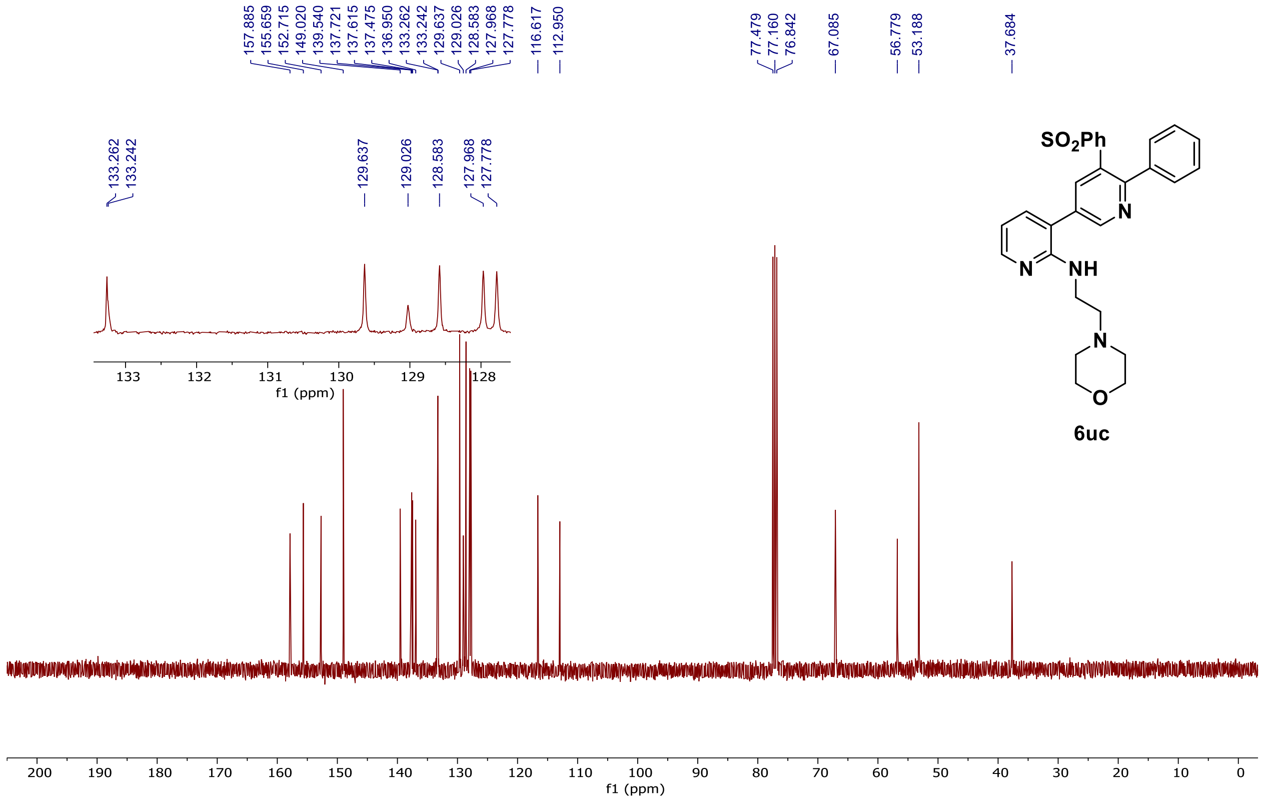

^{1}H and ^{13}C NMR Spectrum of **6uc** in $CDCl_3$

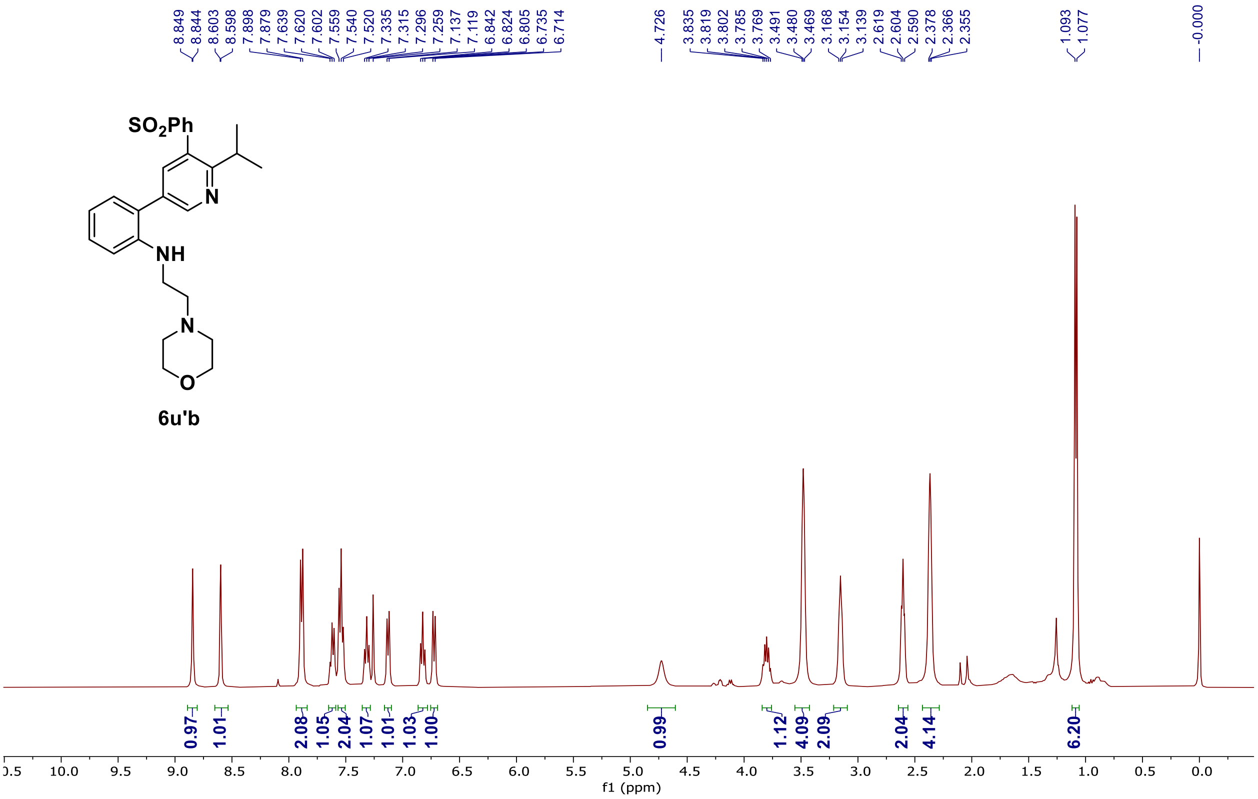

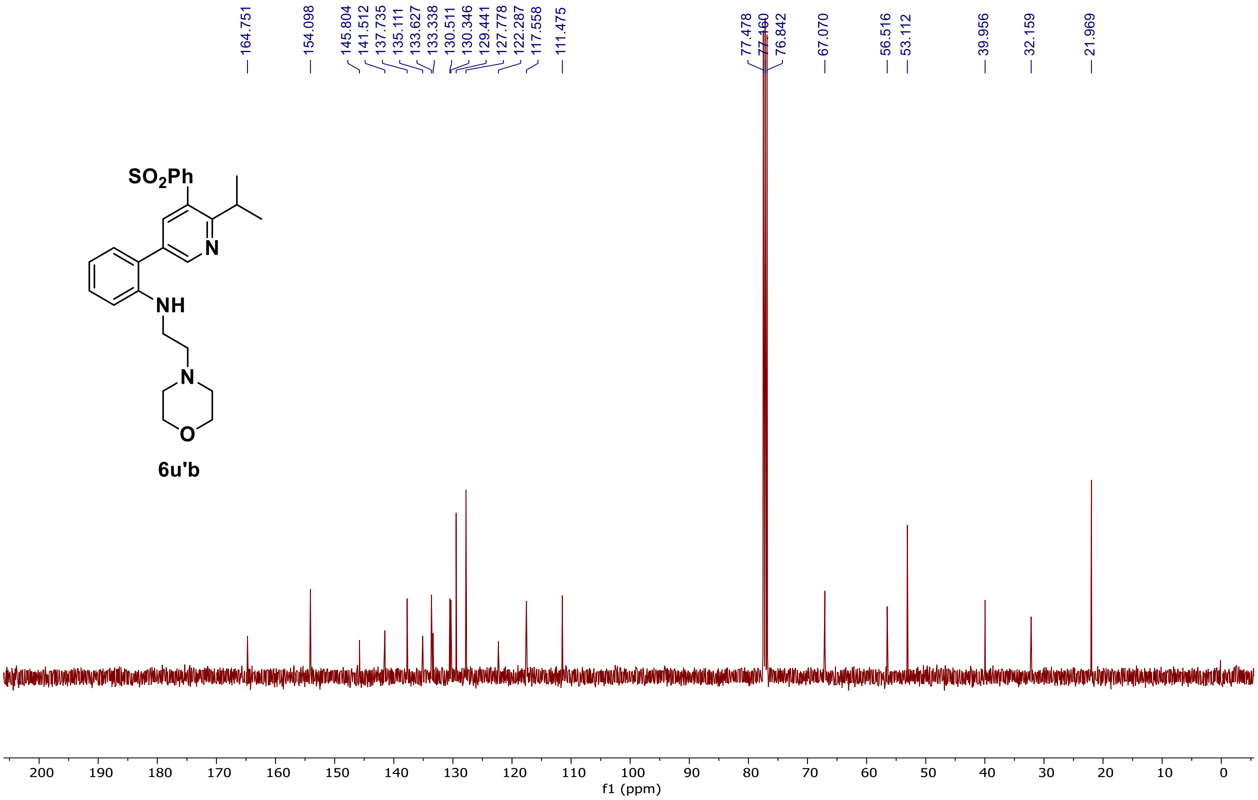

^{1}H and ^{13}C NMR Spectrum of **6u'b** in $CDCl_3$

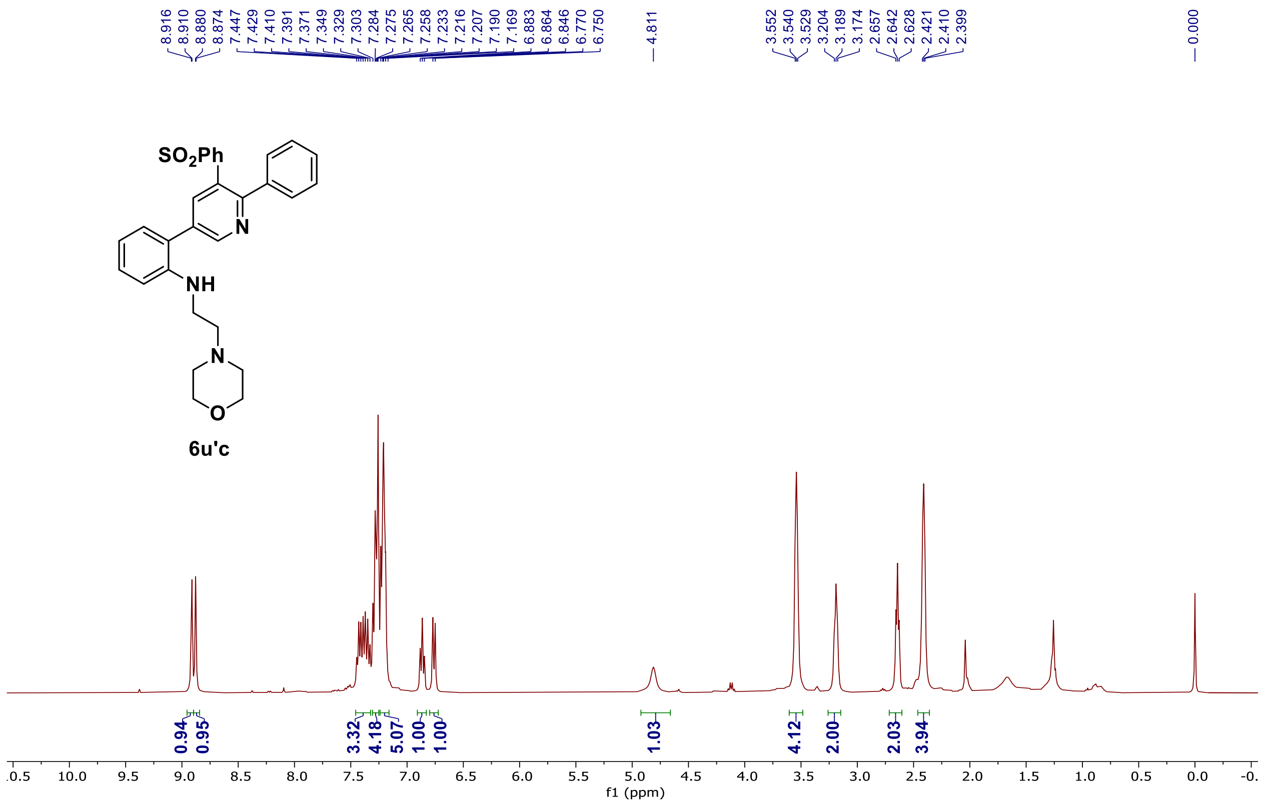

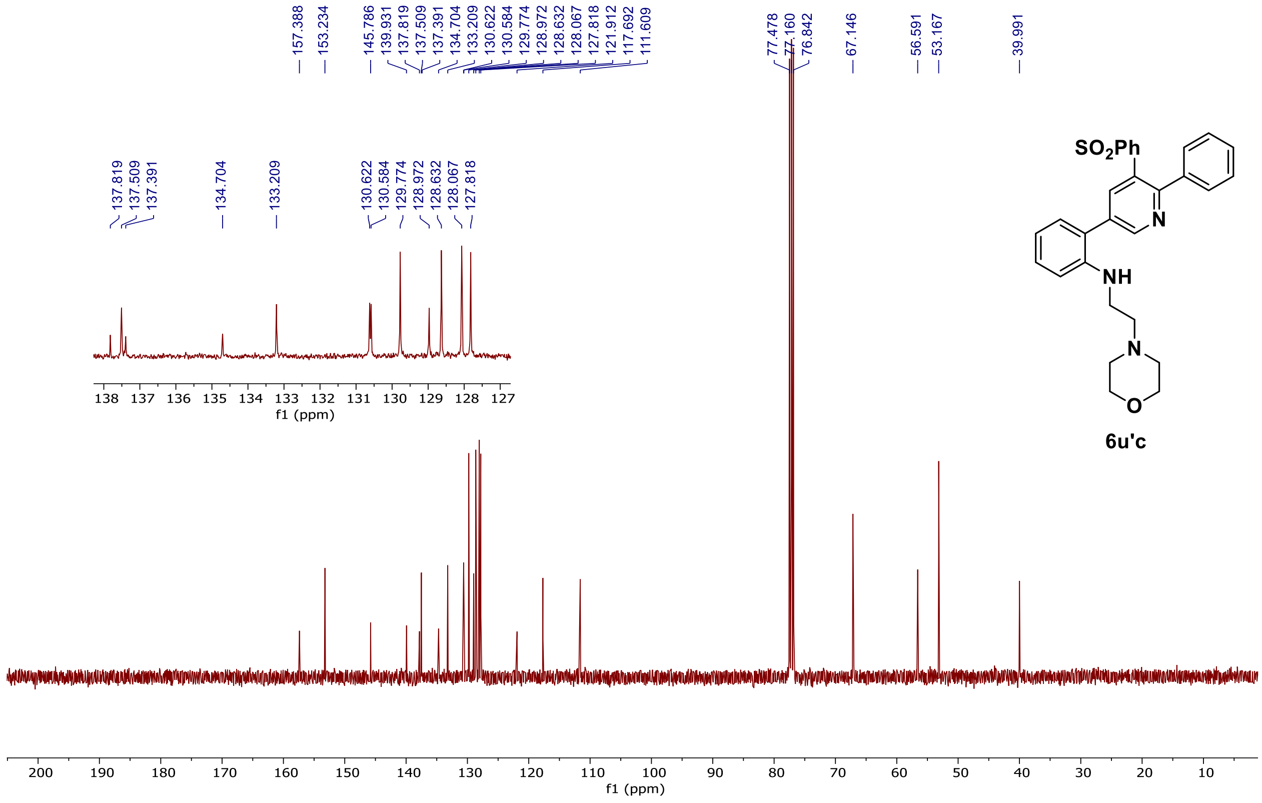

^{1}H and ^{13}C NMR Spectrum of **6u'c** in $CDCl_3$

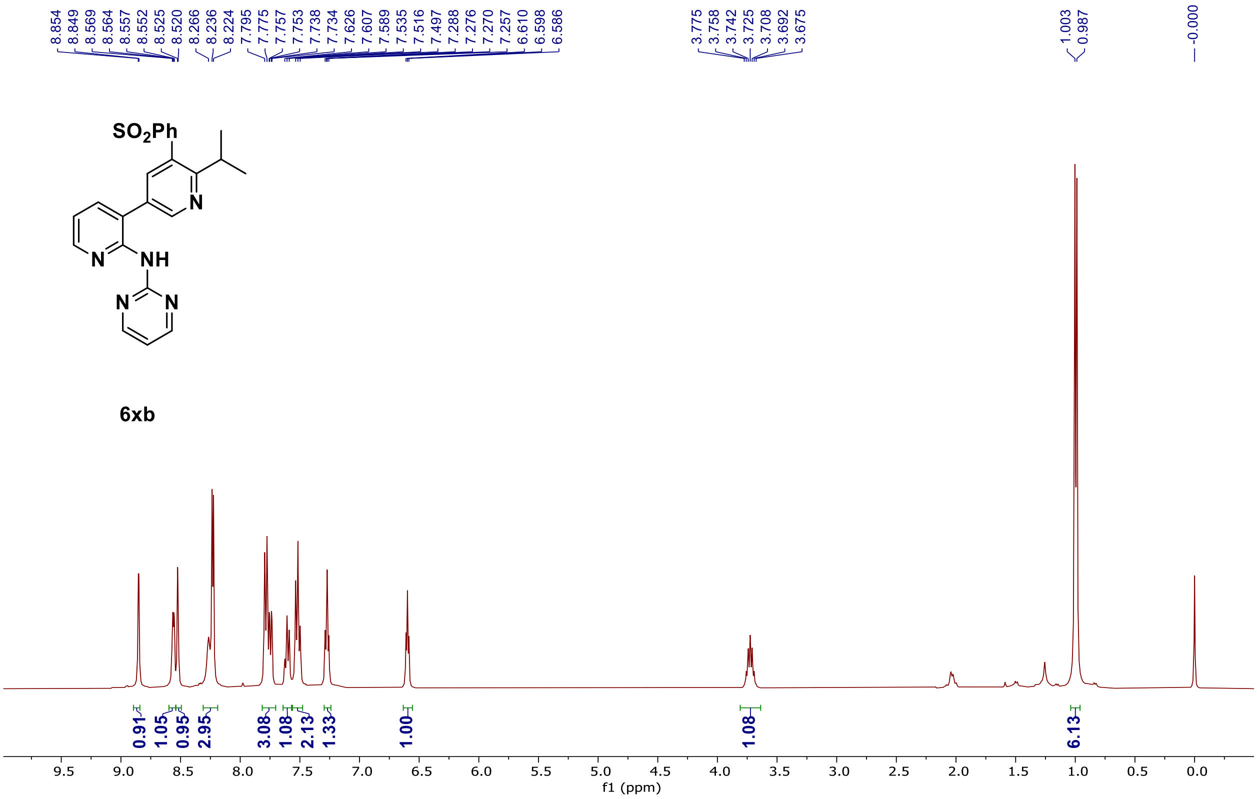

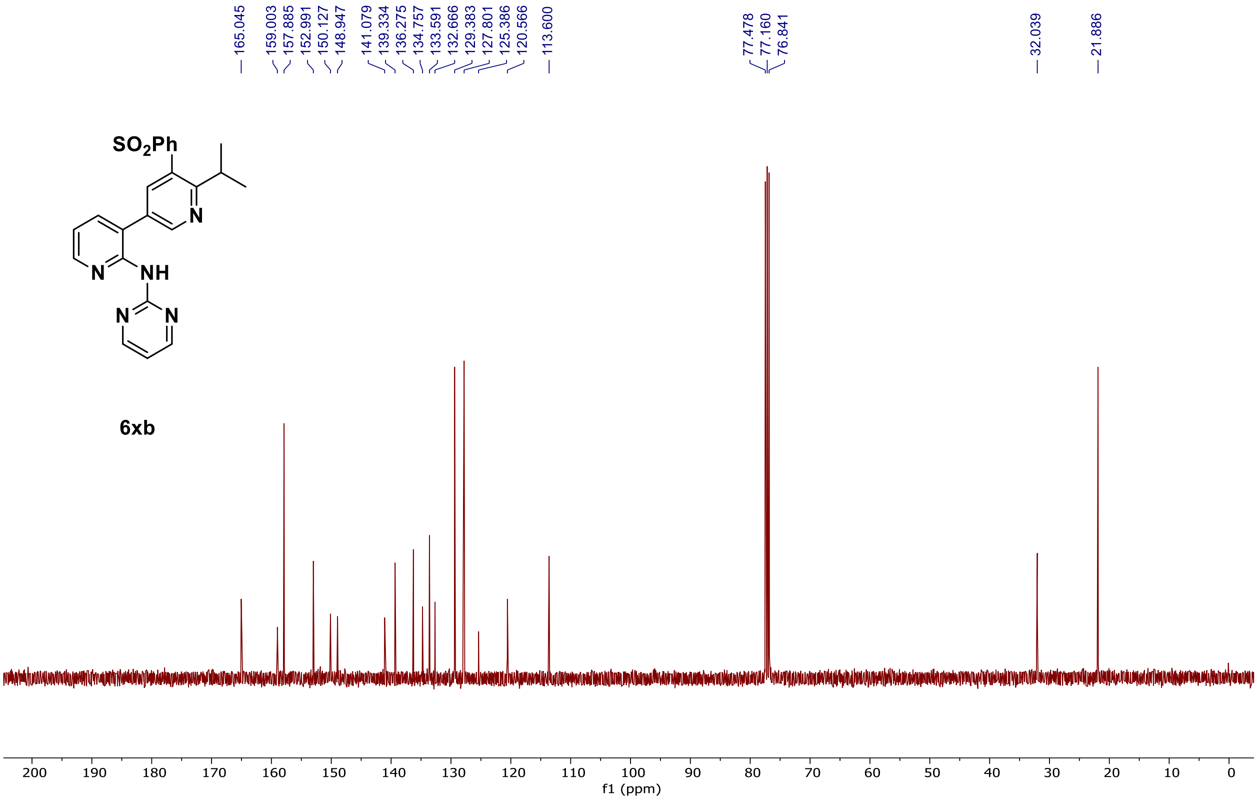

^{1}H and ^{13}C NMR Spectrum of **6xb** in $CDCl_3$

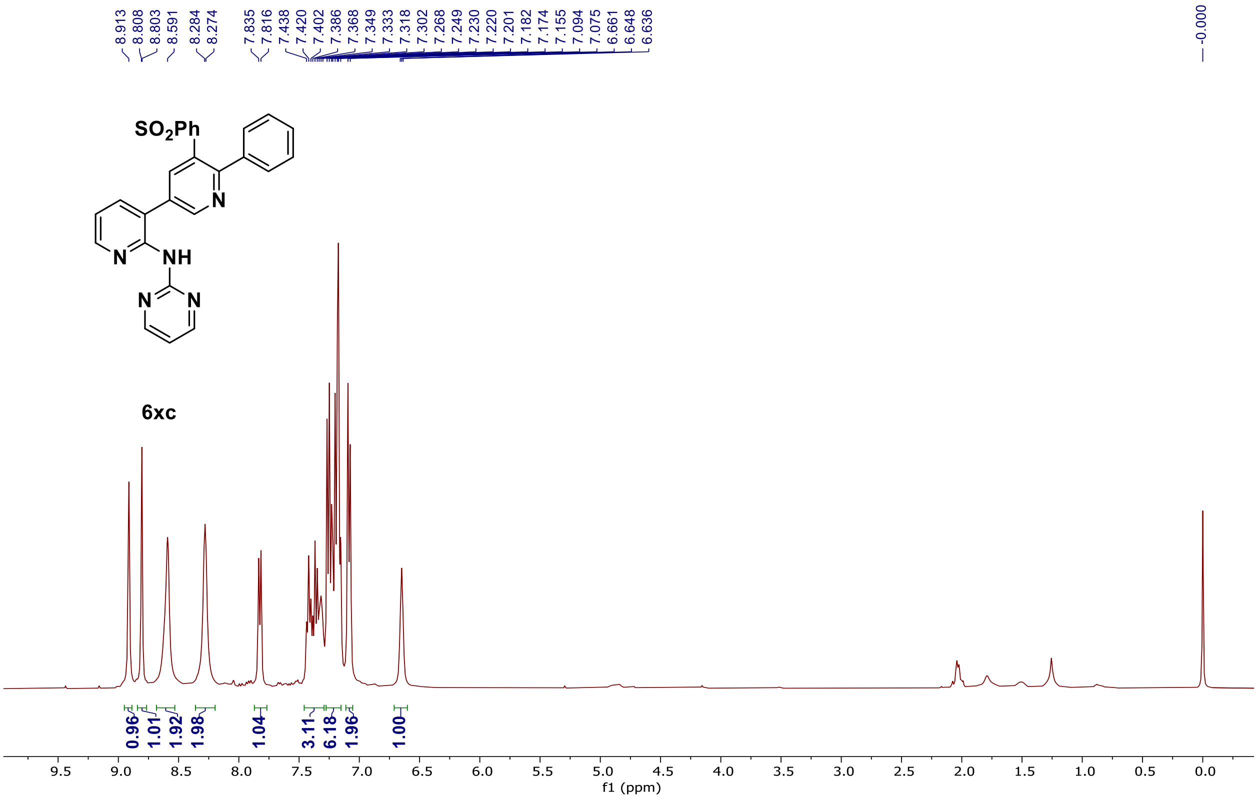

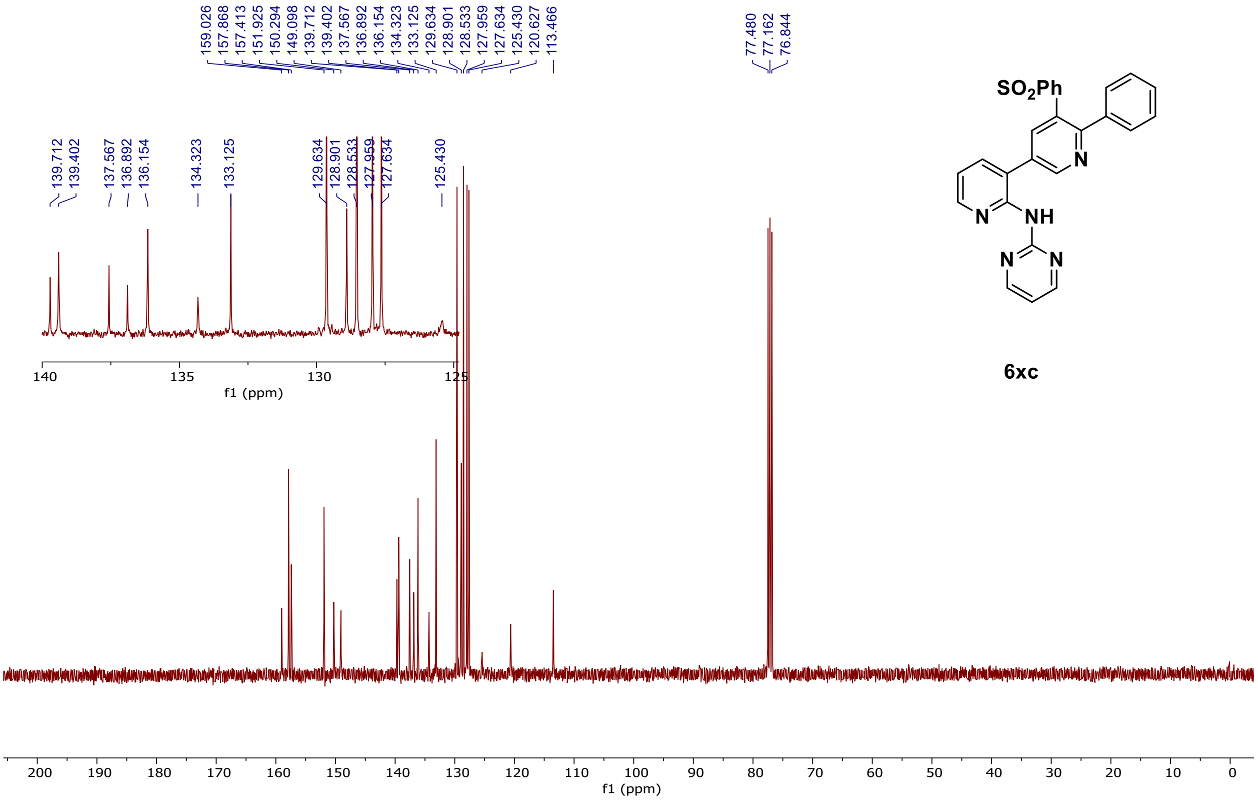

^{1}H and ^{13}C NMR Spectrum of **6xc** in $CDCl_3$

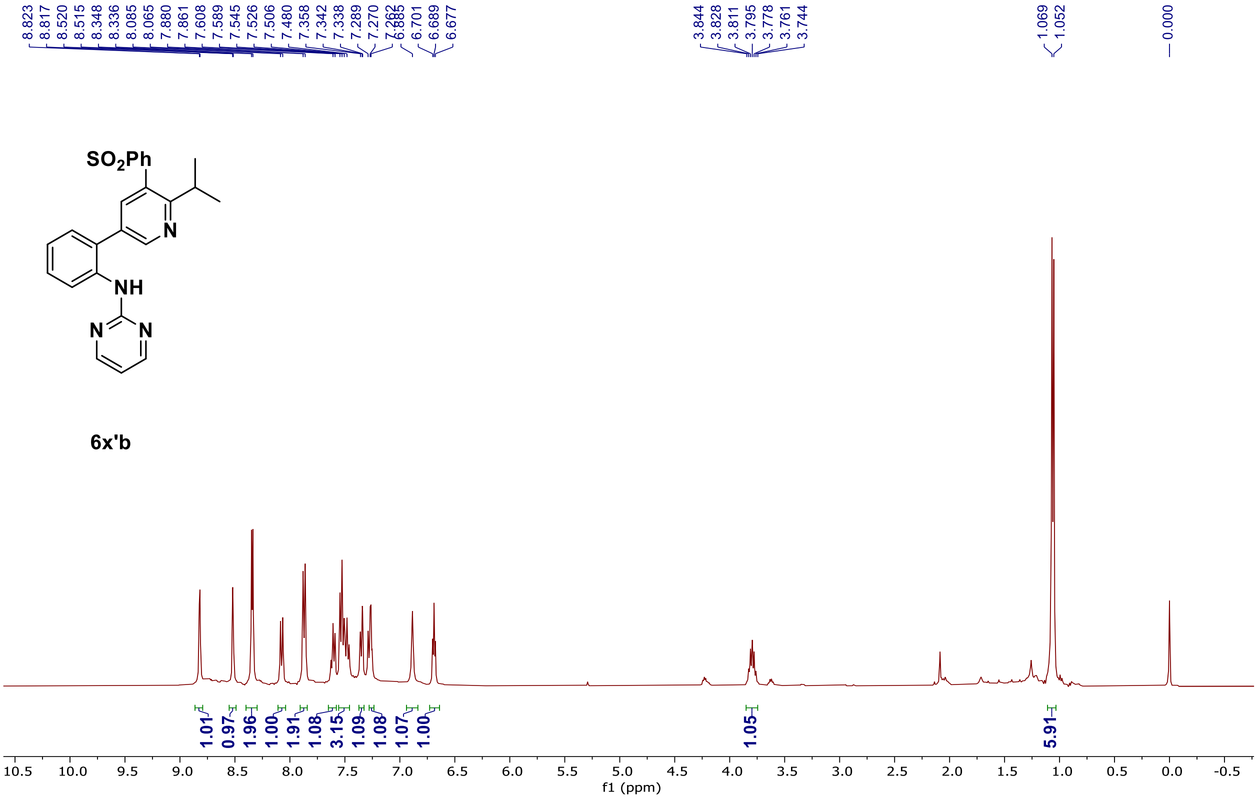

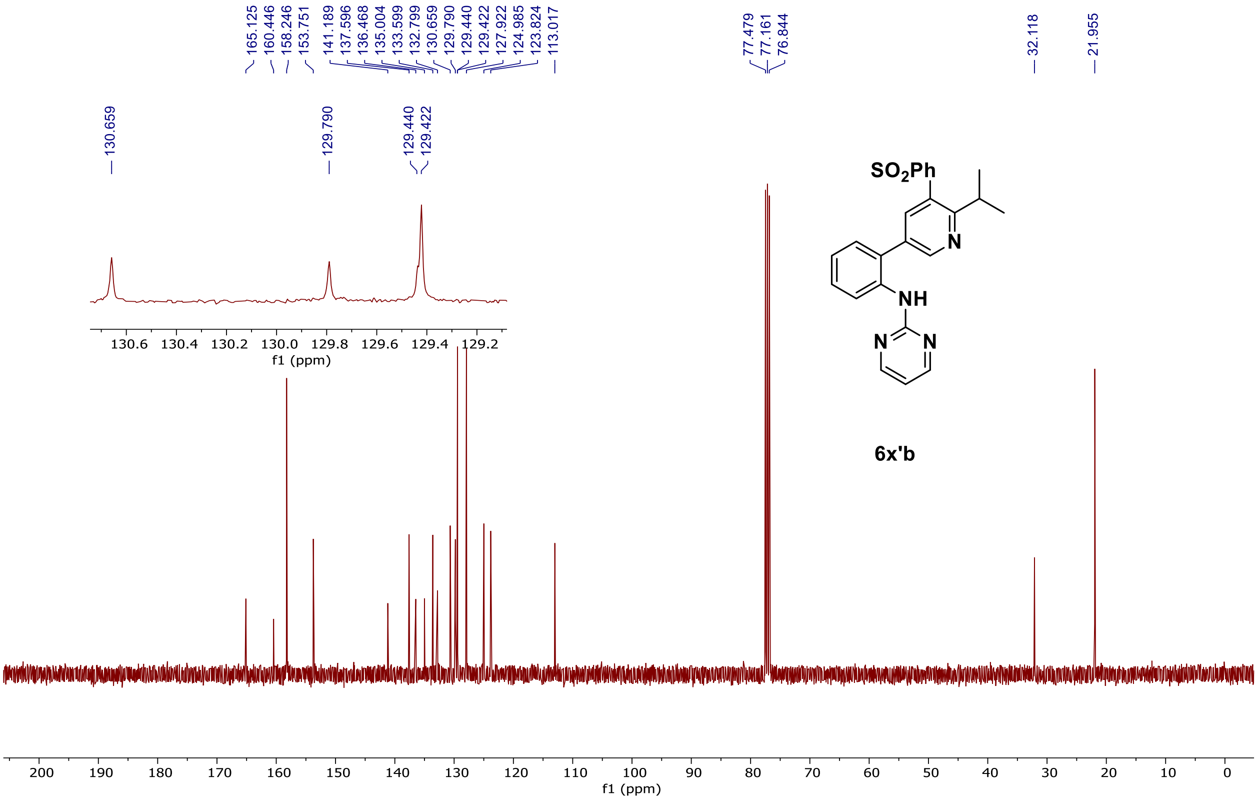

^{1}H and ^{13}C NMR Spectrum of **6x'b** in $CDCl_3$

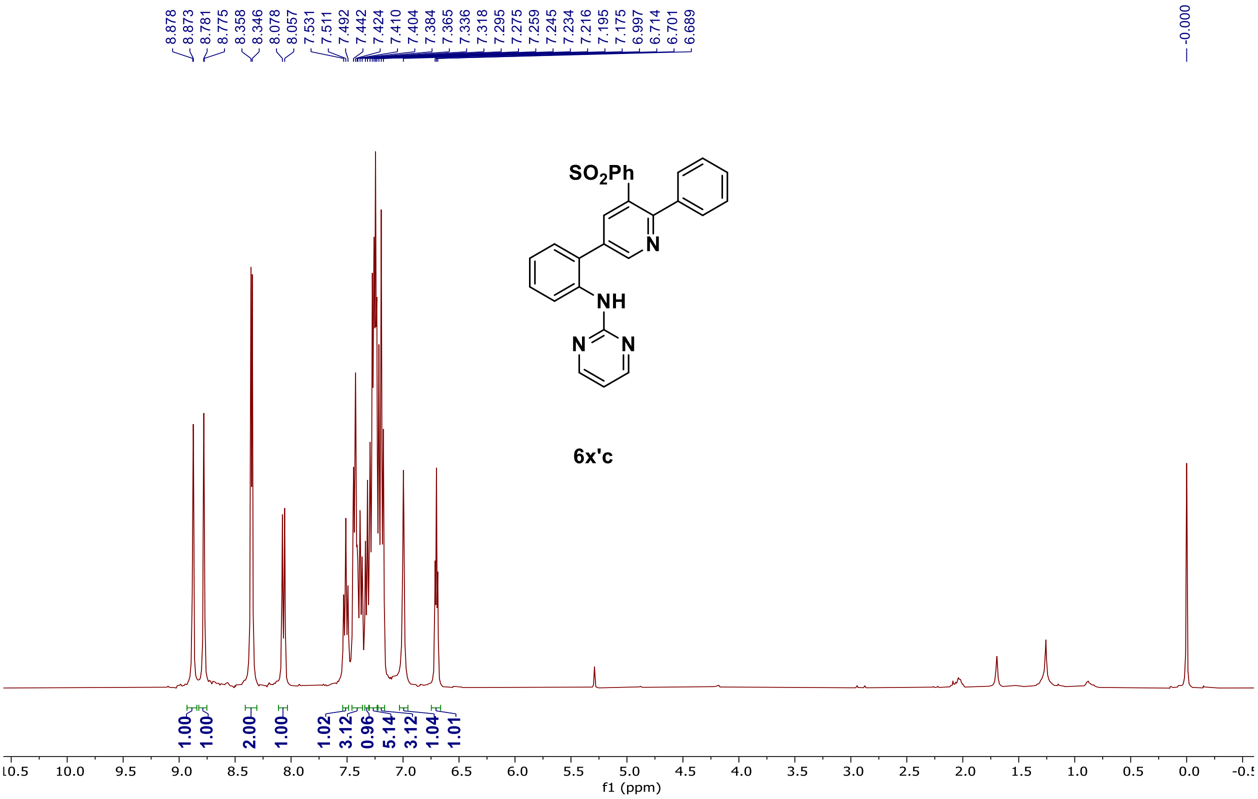

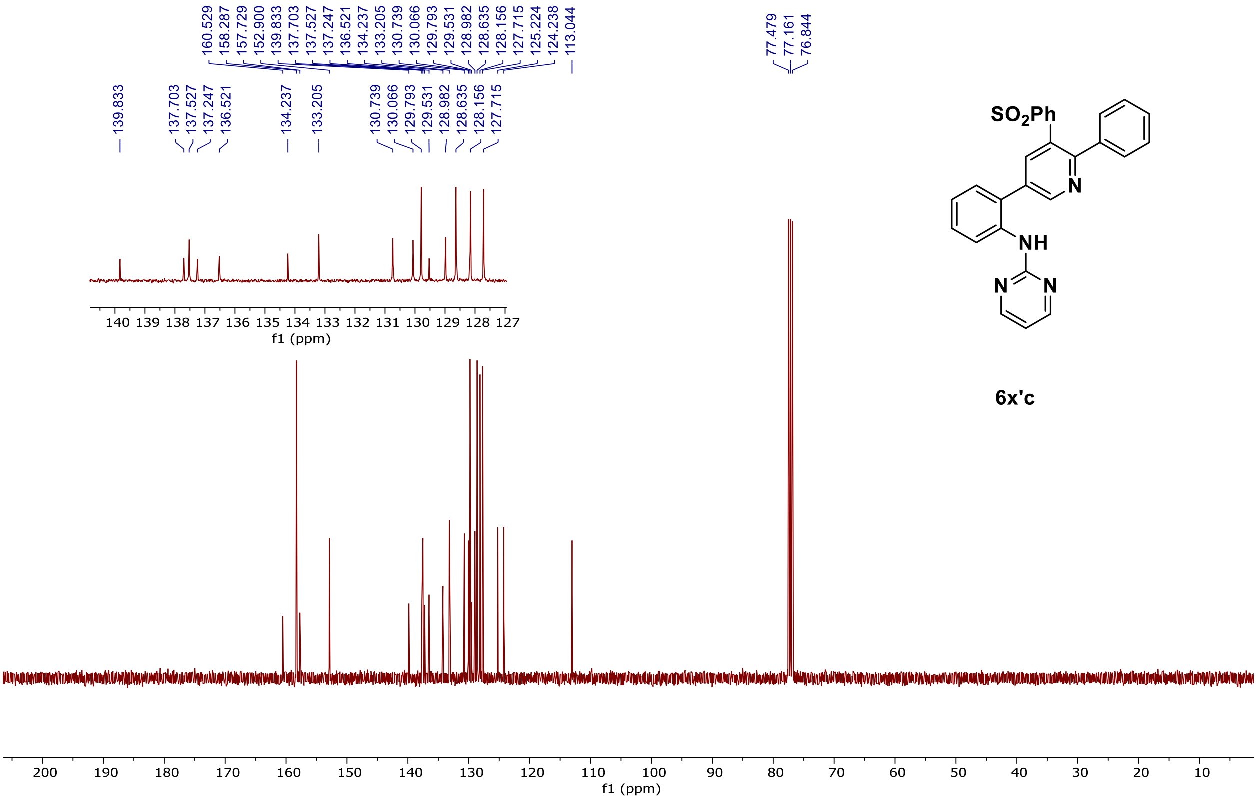

^{1}H and ^{13}C NMR Spectrum of **6x'c** in $CDCl_3$

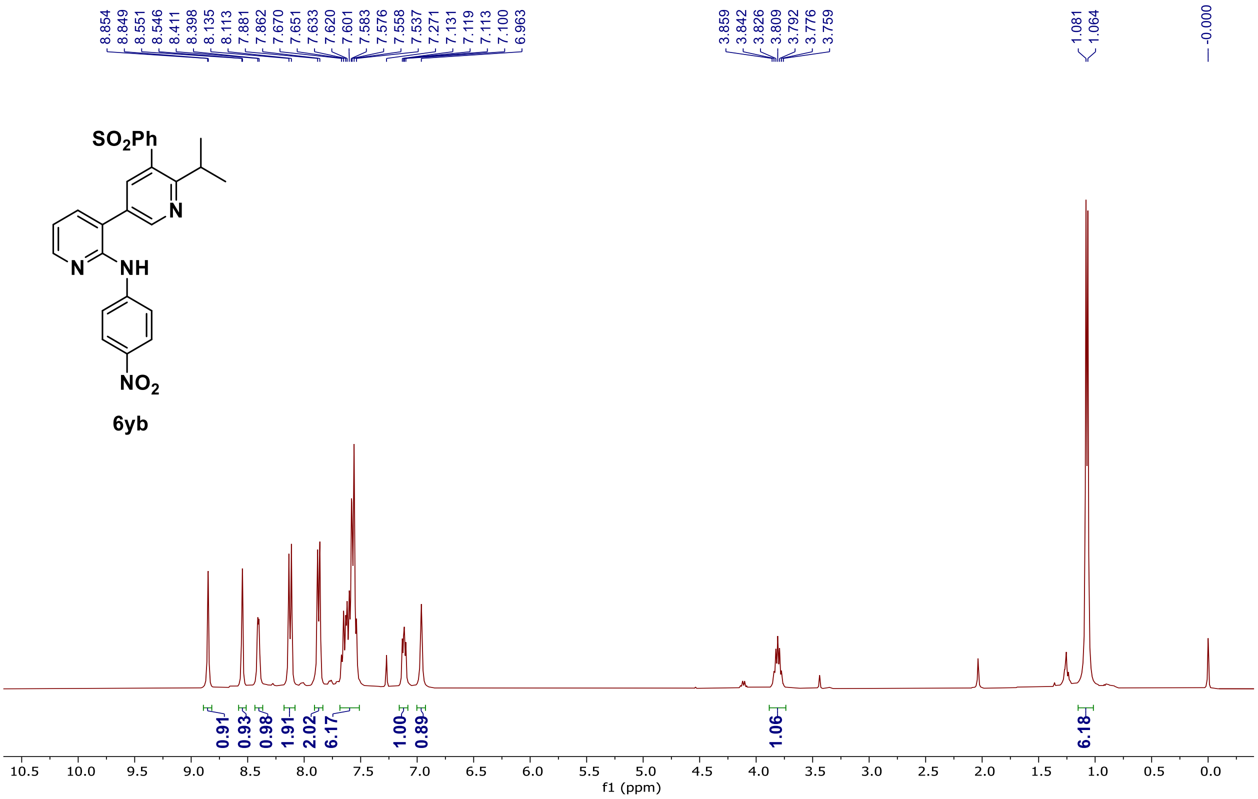

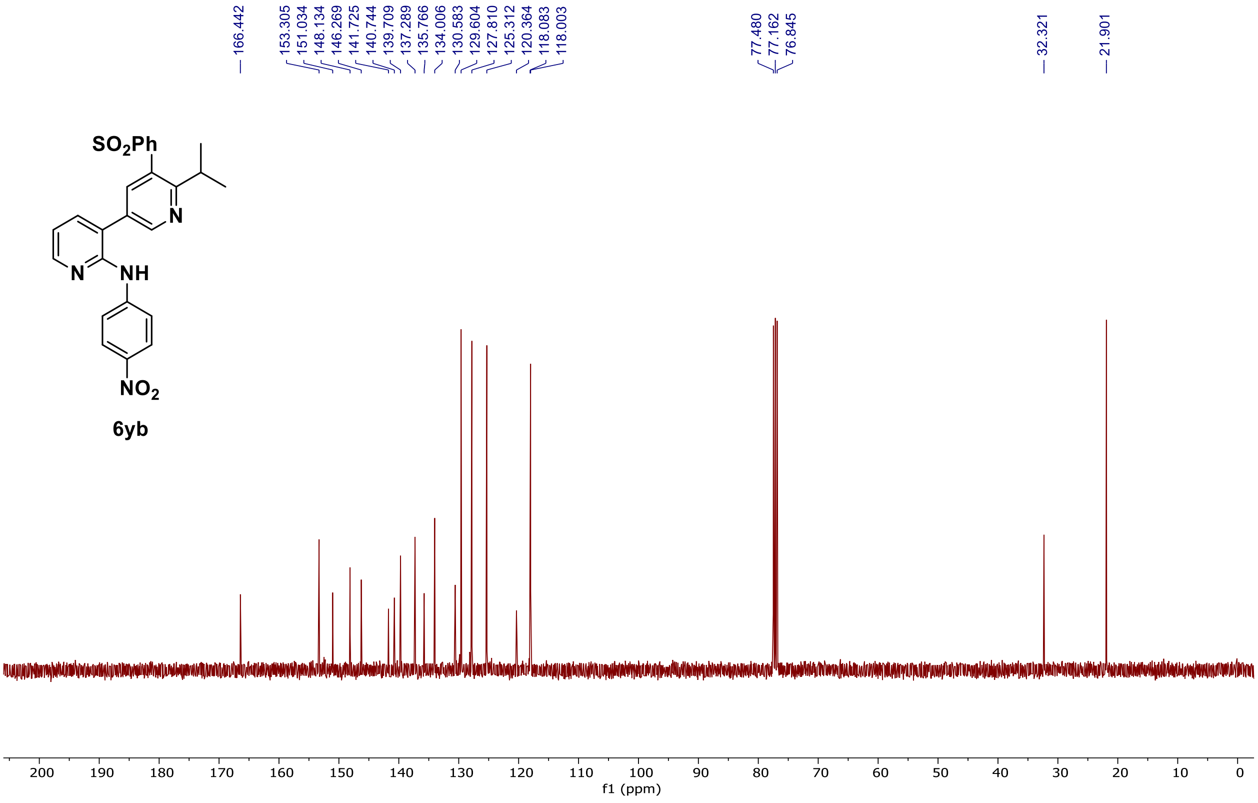

^{1}H and ^{13}C NMR Spectrum of **6yb** in $CDCl_3$

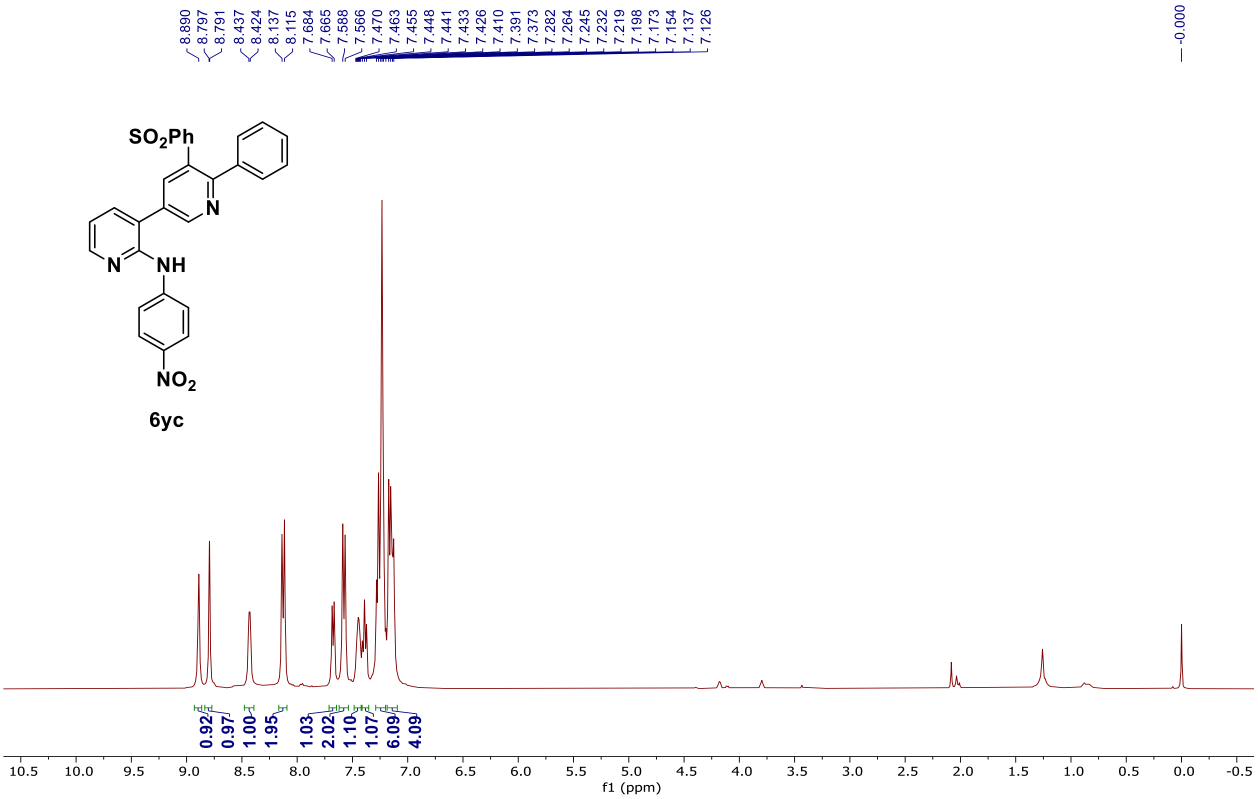

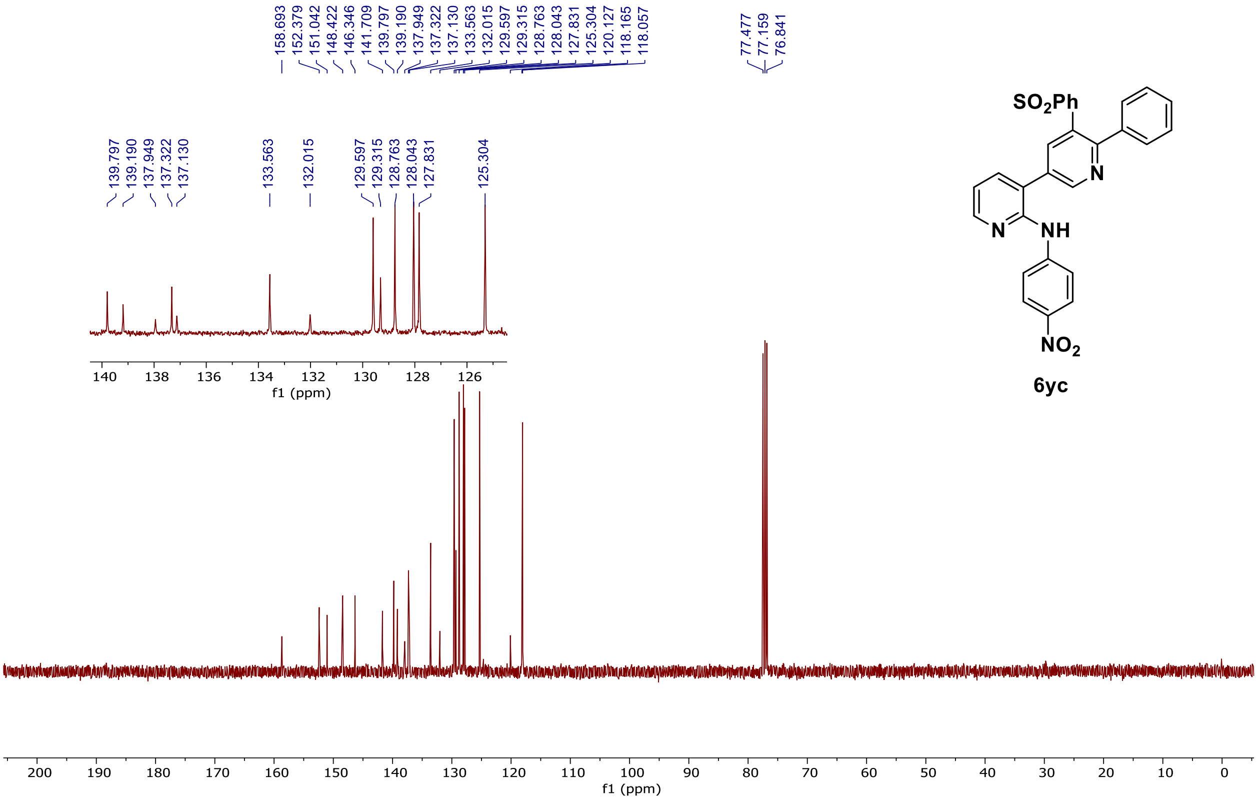

^{1}H and ^{13}C NMR Spectrum of **6yc** in $CDCl_3$

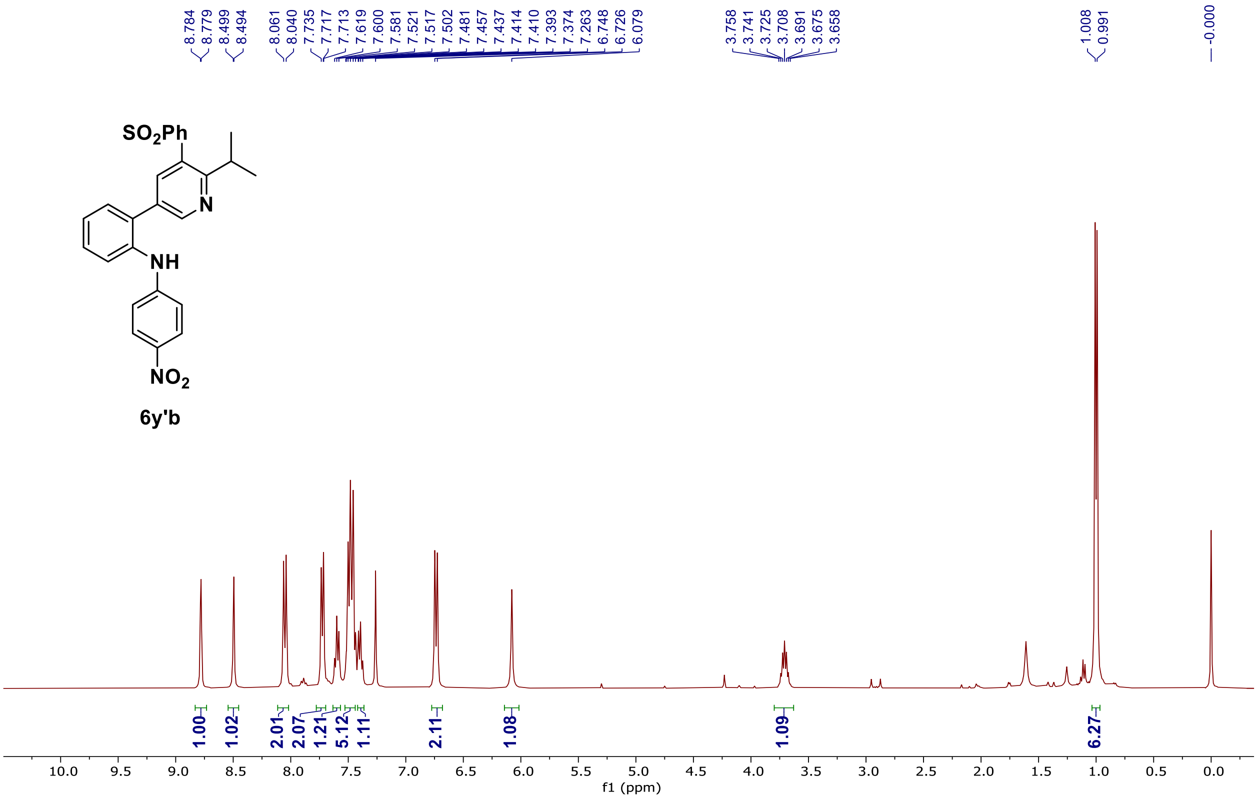

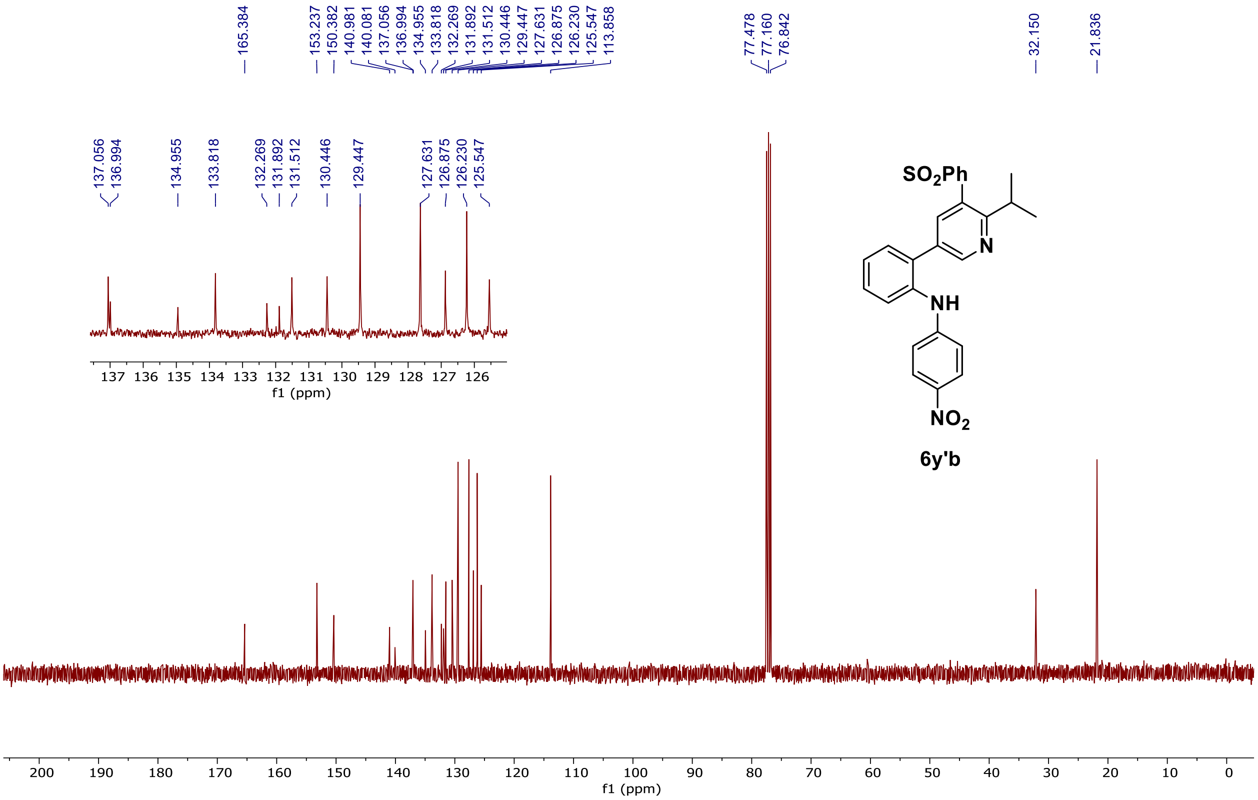

^{1}H and ^{13}C NMR Spectrum of **6y'b** in $CDCl_3$

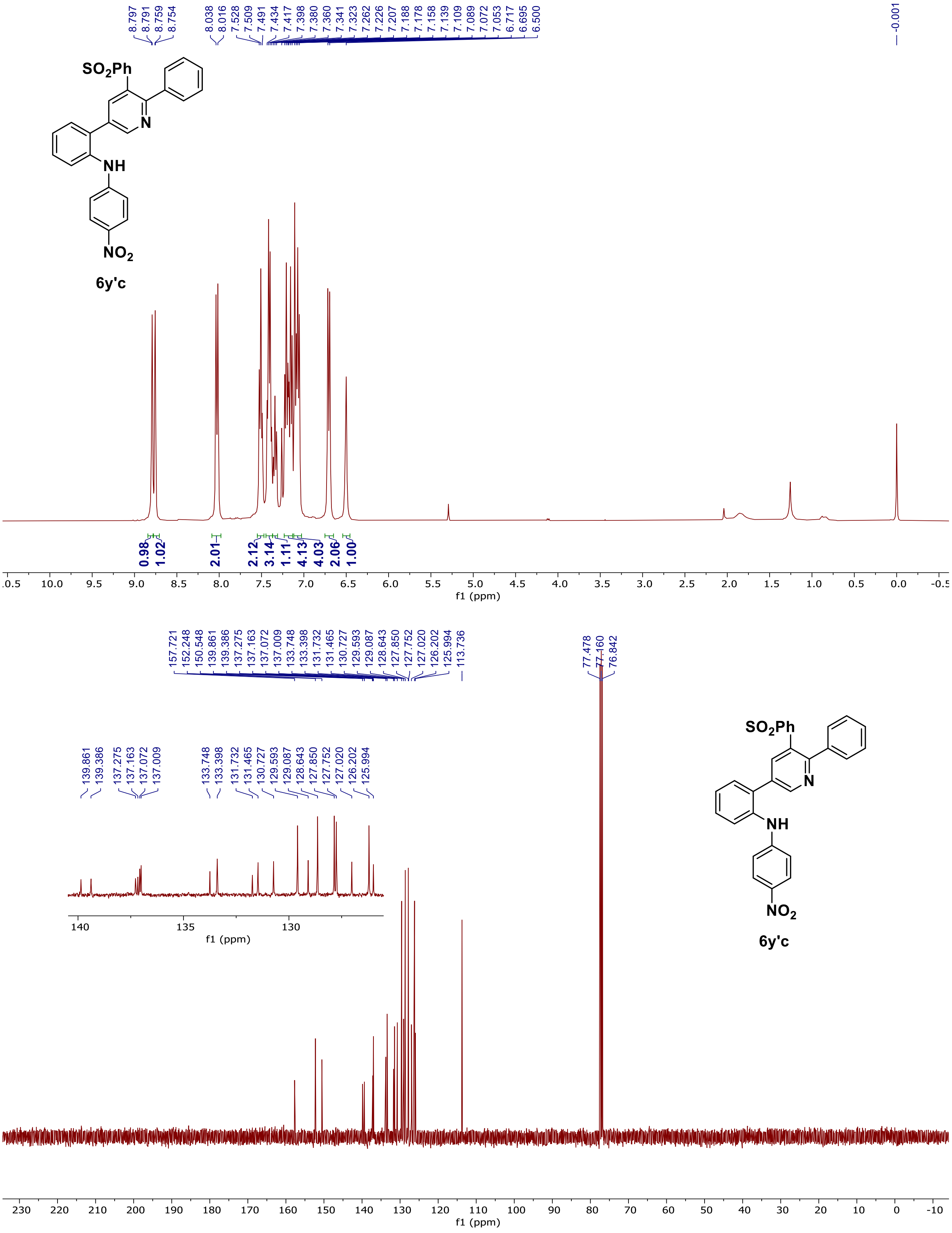

^{1}H and ^{13}C NMR Spectrum of **6y'c** in $CDCl_3$

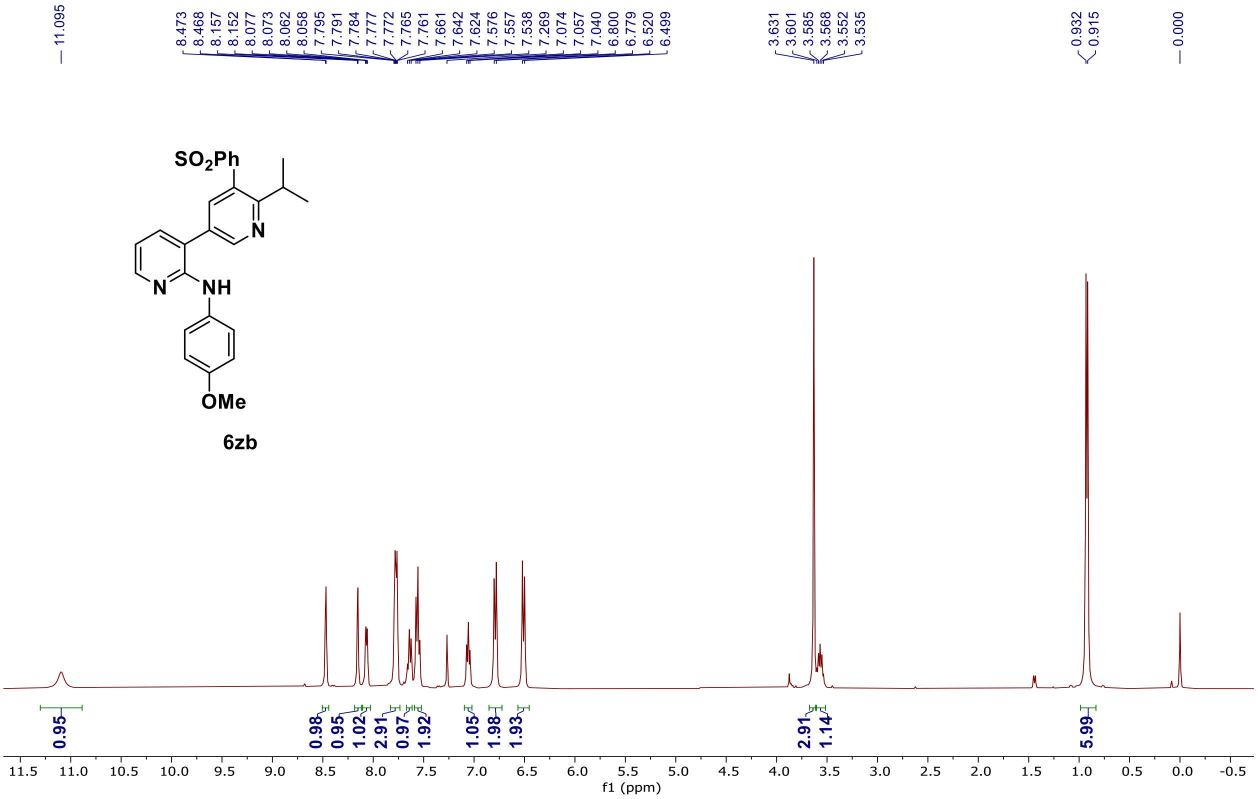

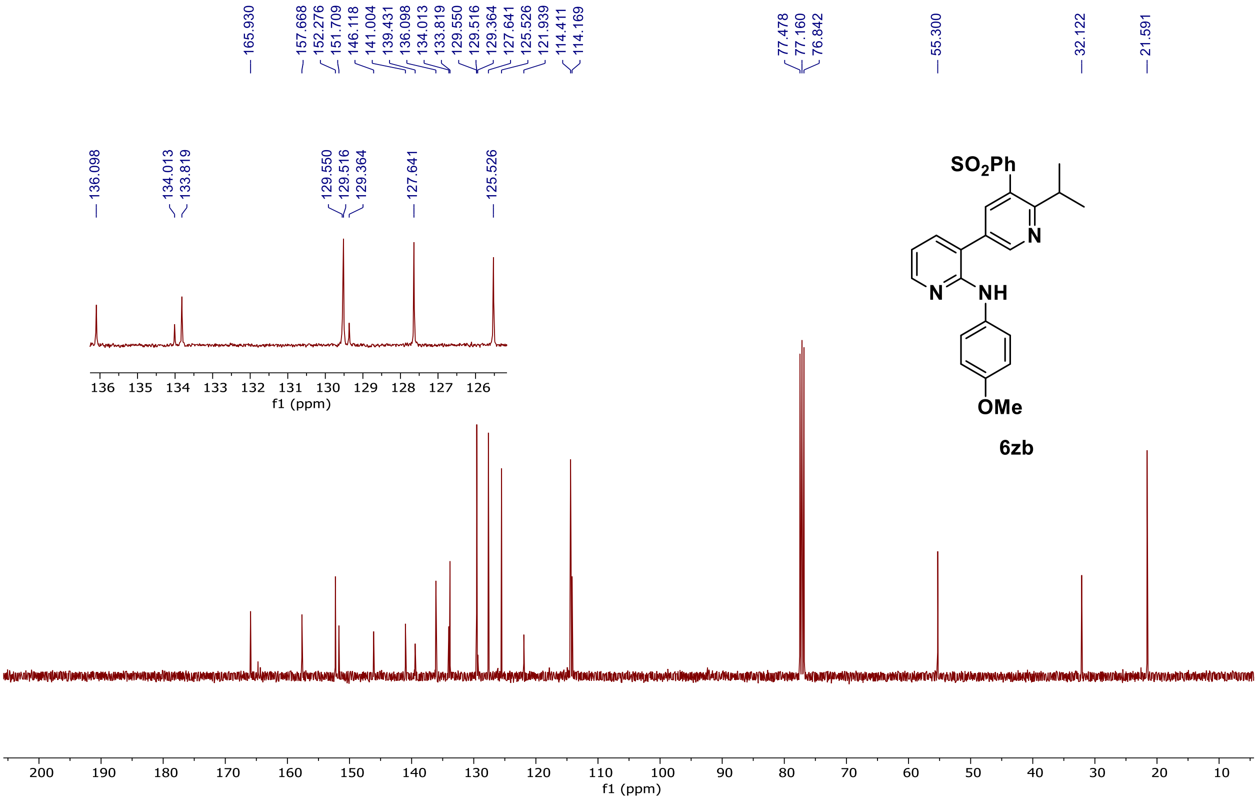

^{1}H and ^{13}C NMR Spectrum of **6zb** in $CDCl_3$

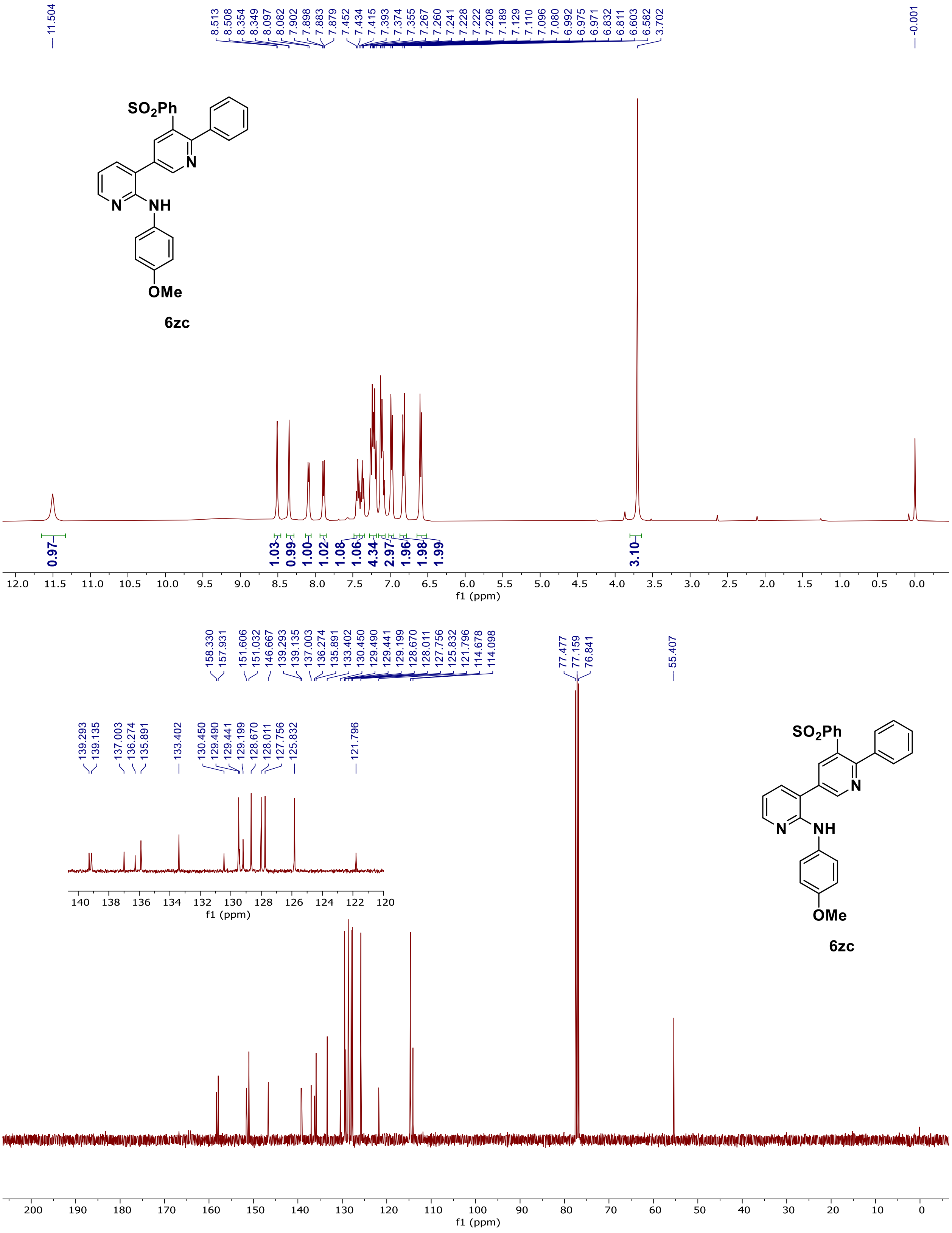

^{1}H and ^{13}C NMR Spectrum of **6zc** in $CDCl_3$

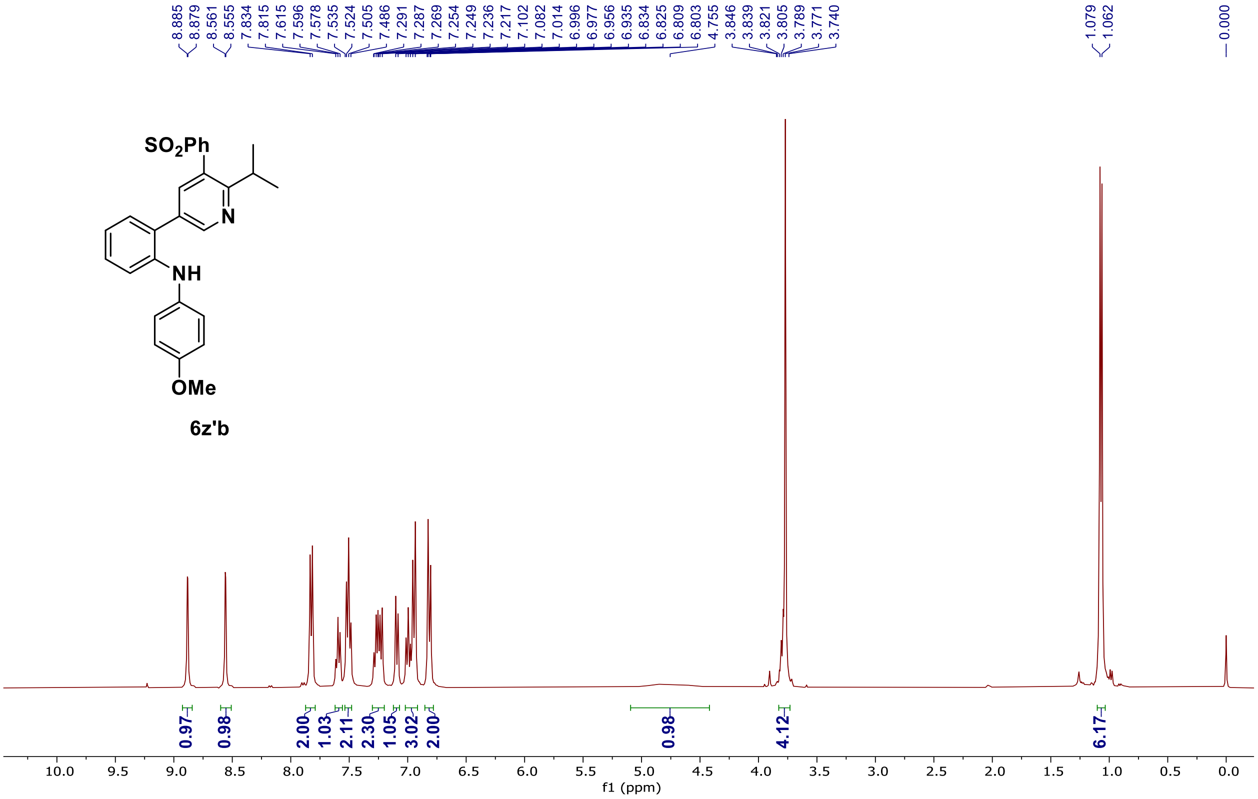

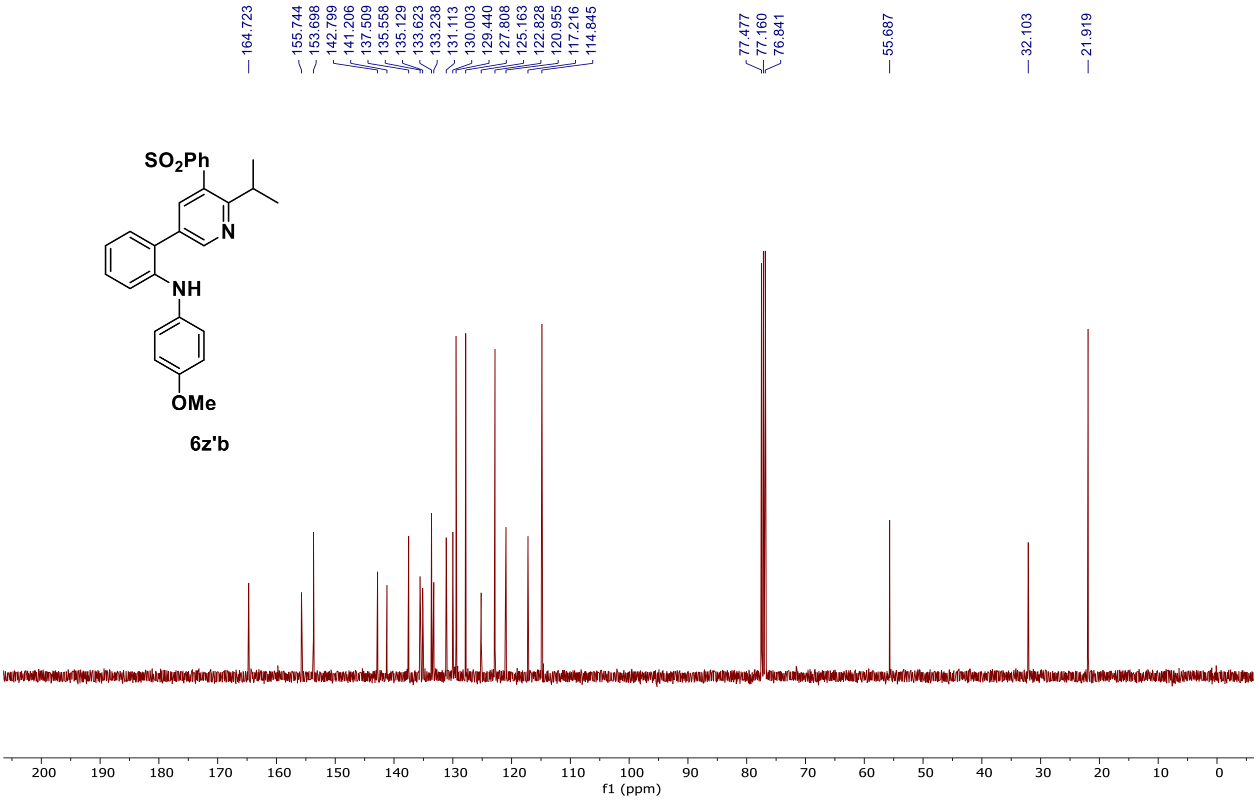

^{1}H and ^{13}C NMR Spectrum of **6z'b** in $CDCl_3$

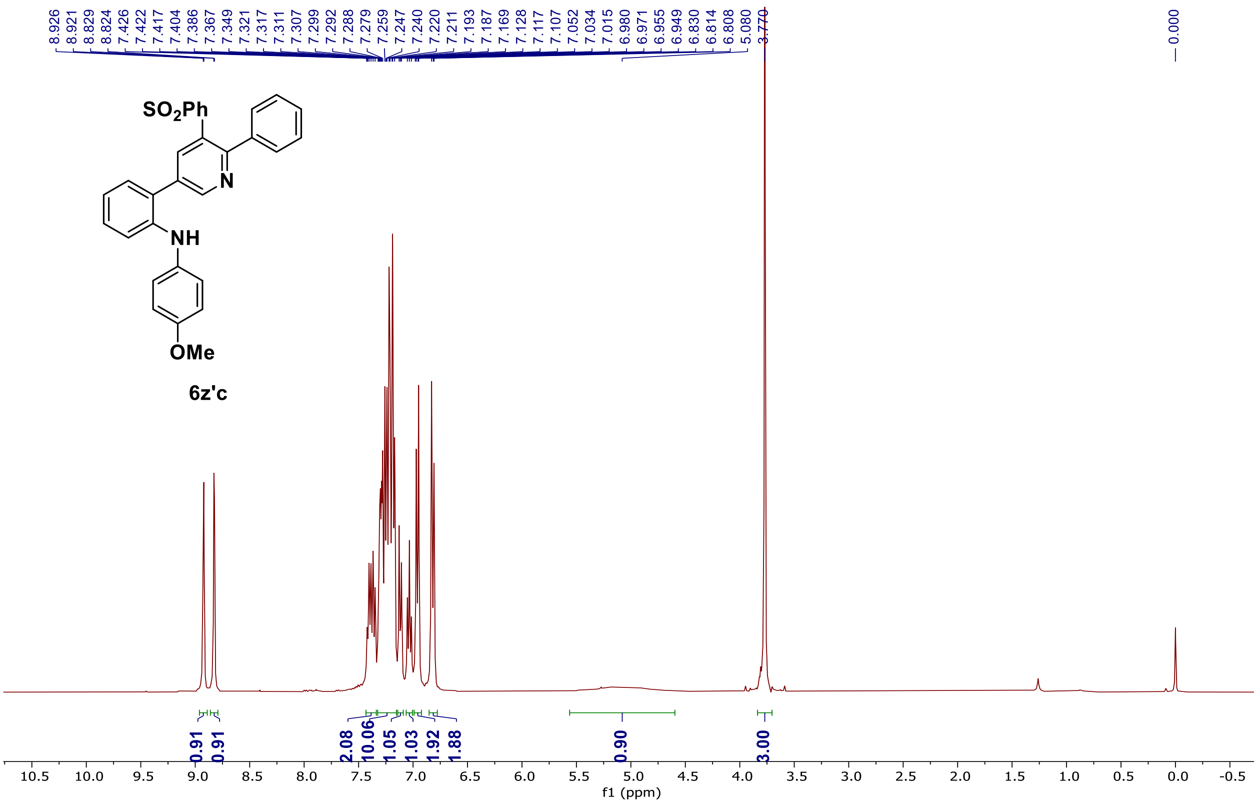

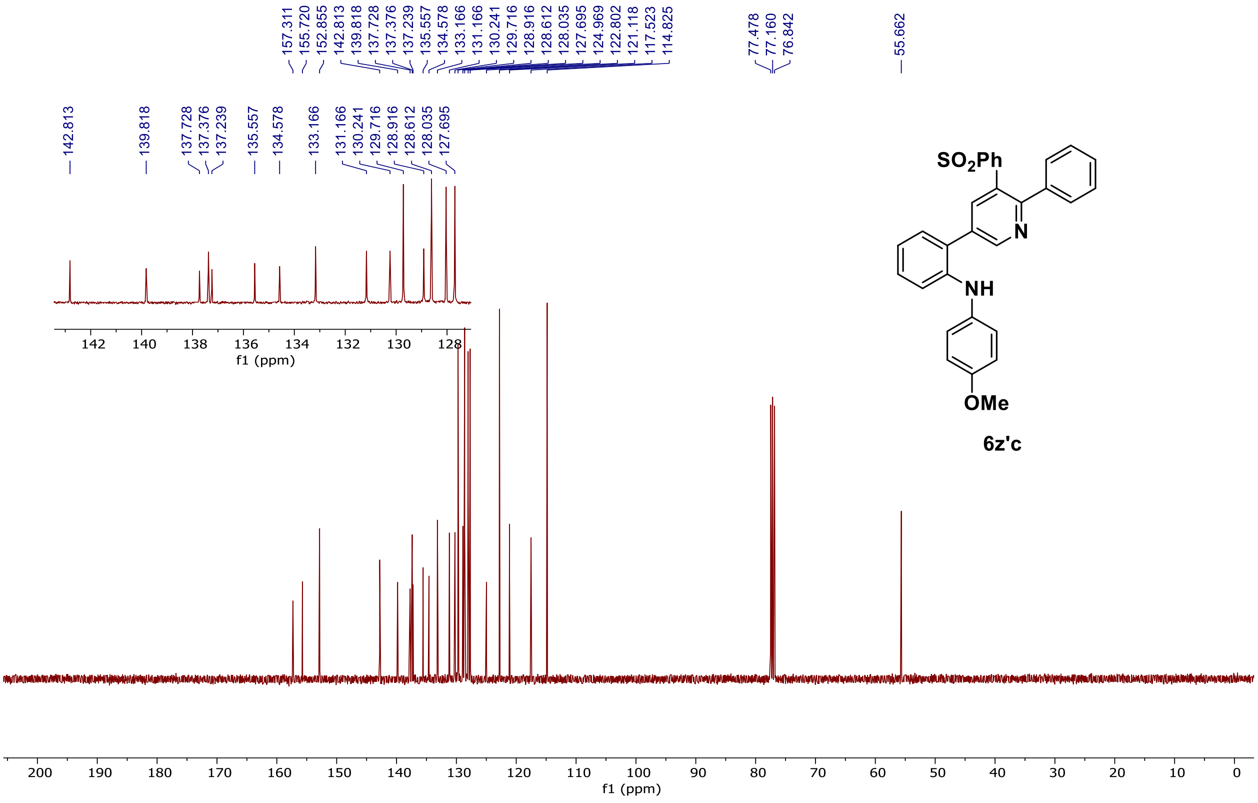

^{1}H and ^{13}C NMR Spectrum of **6z'c** in $CDCl_3$

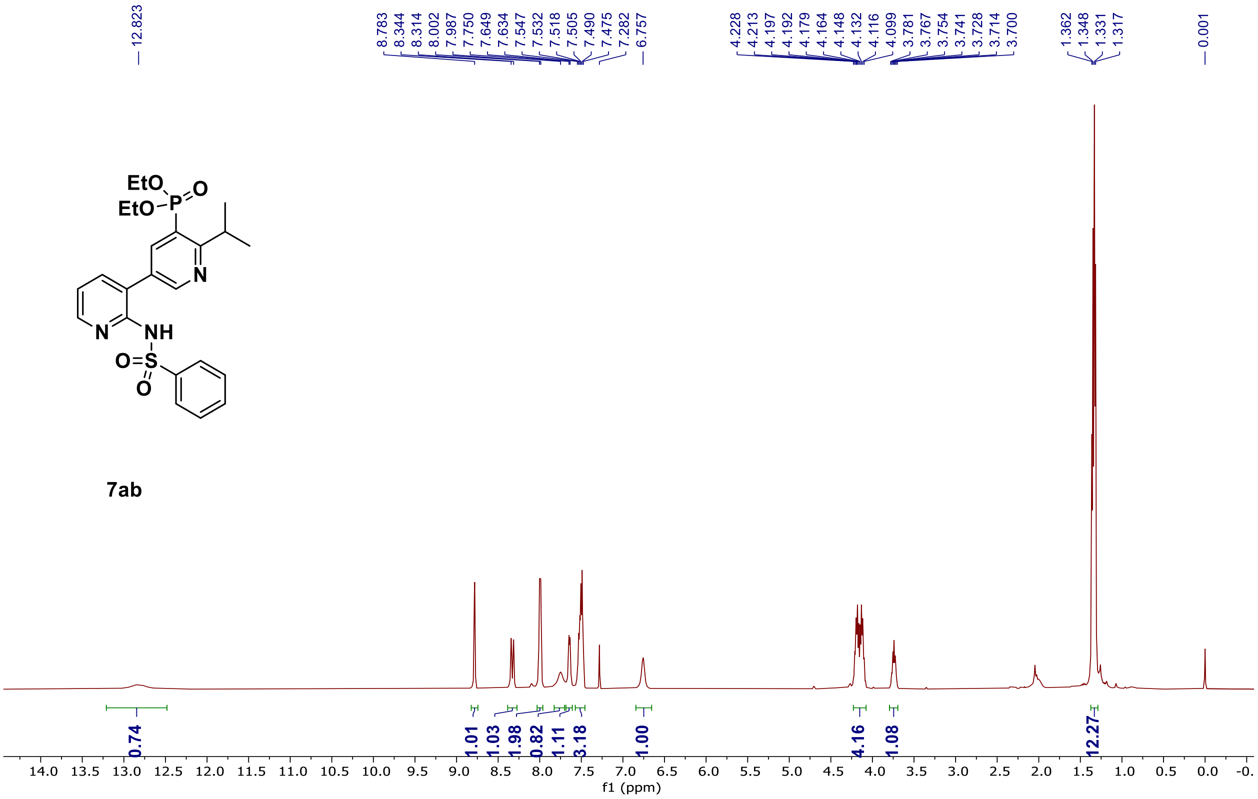

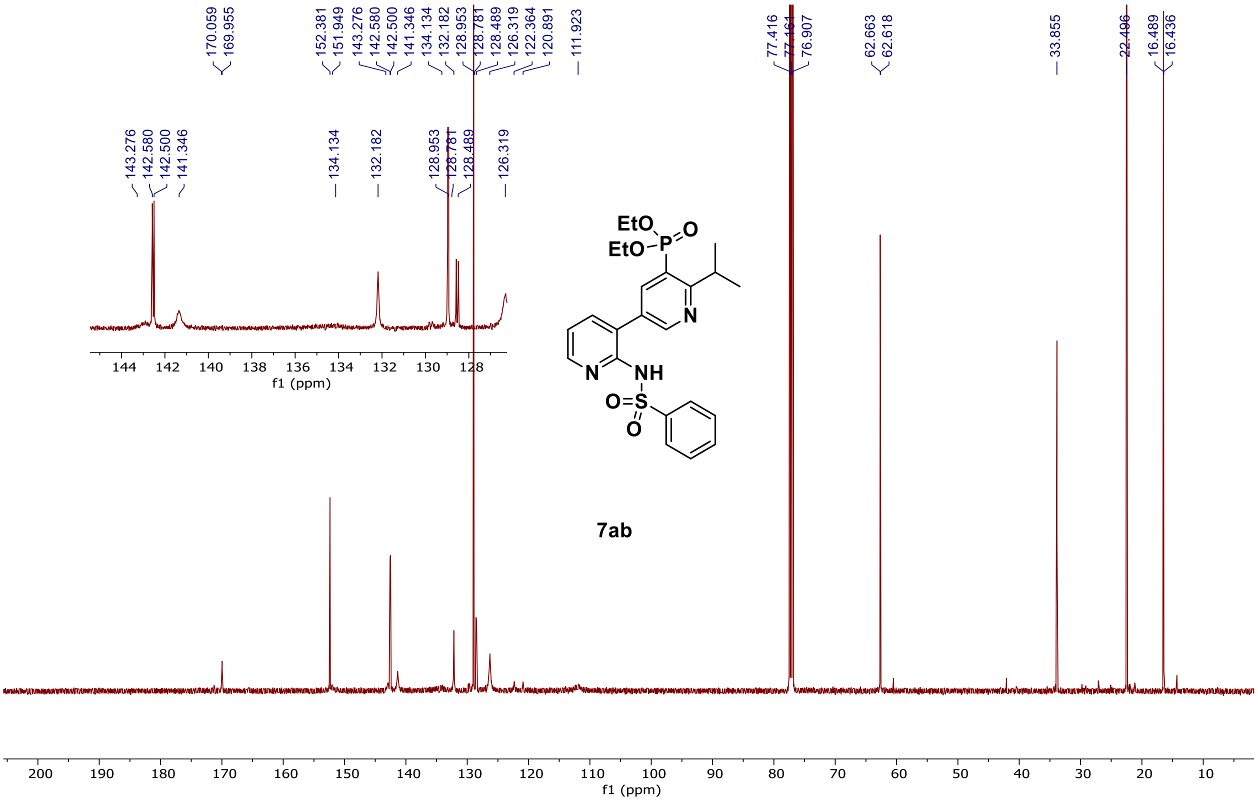

^{1}H and ^{13}C NMR Spectrum of **7ab** in $CDCl_3$

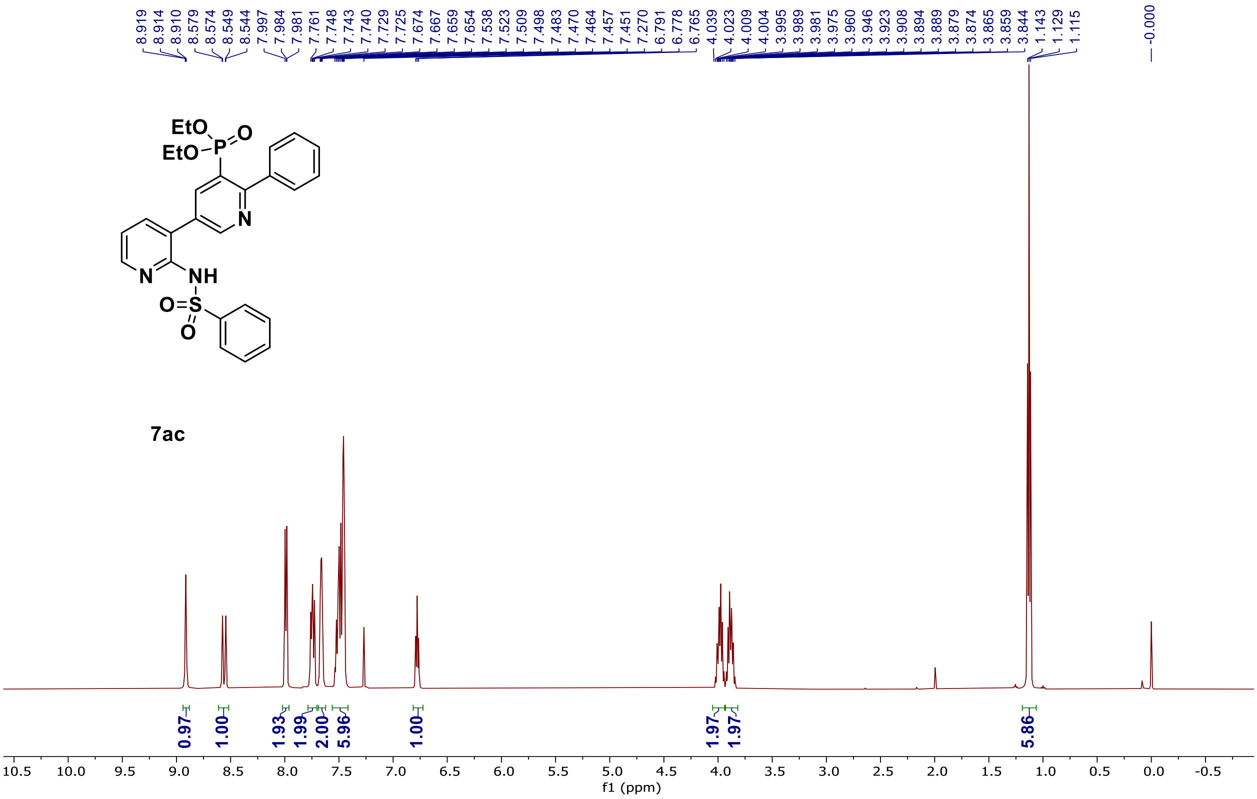

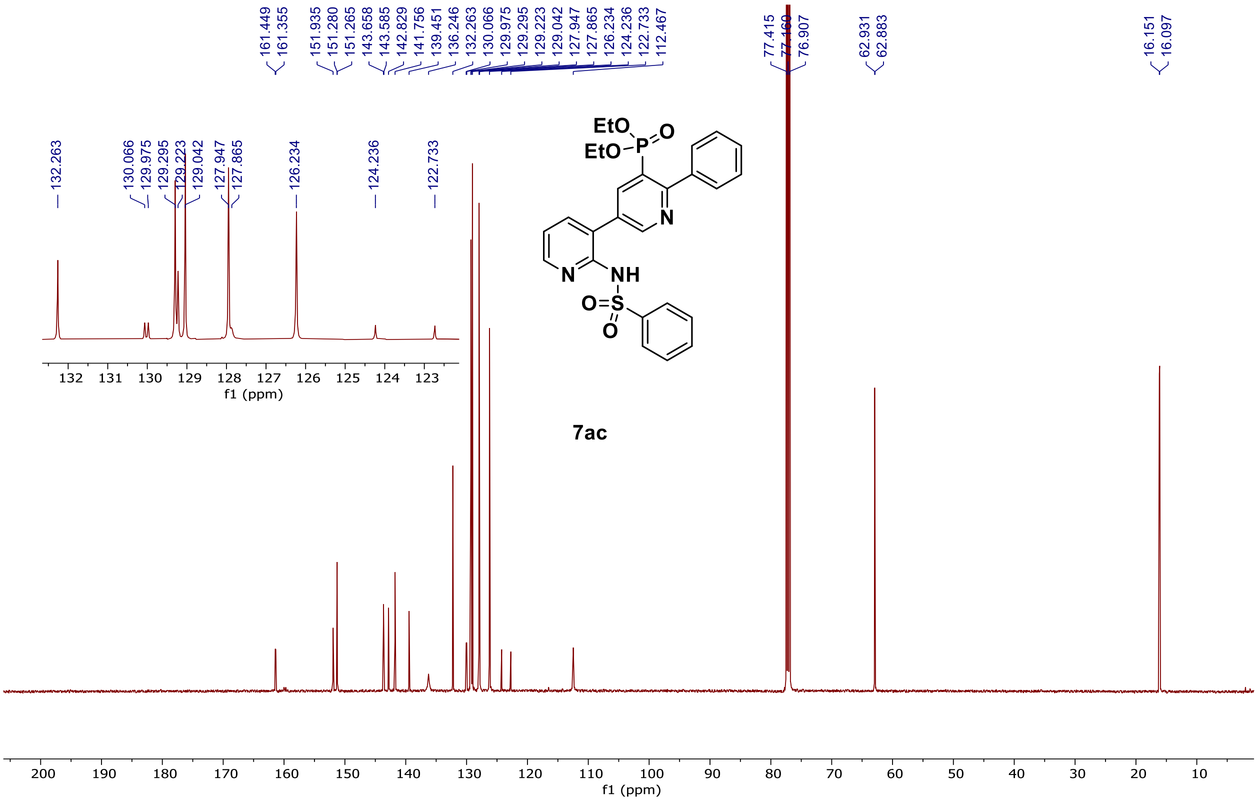

^{1}H and ^{13}C NMR Spectrum of **7ac** in $CDCl_3$

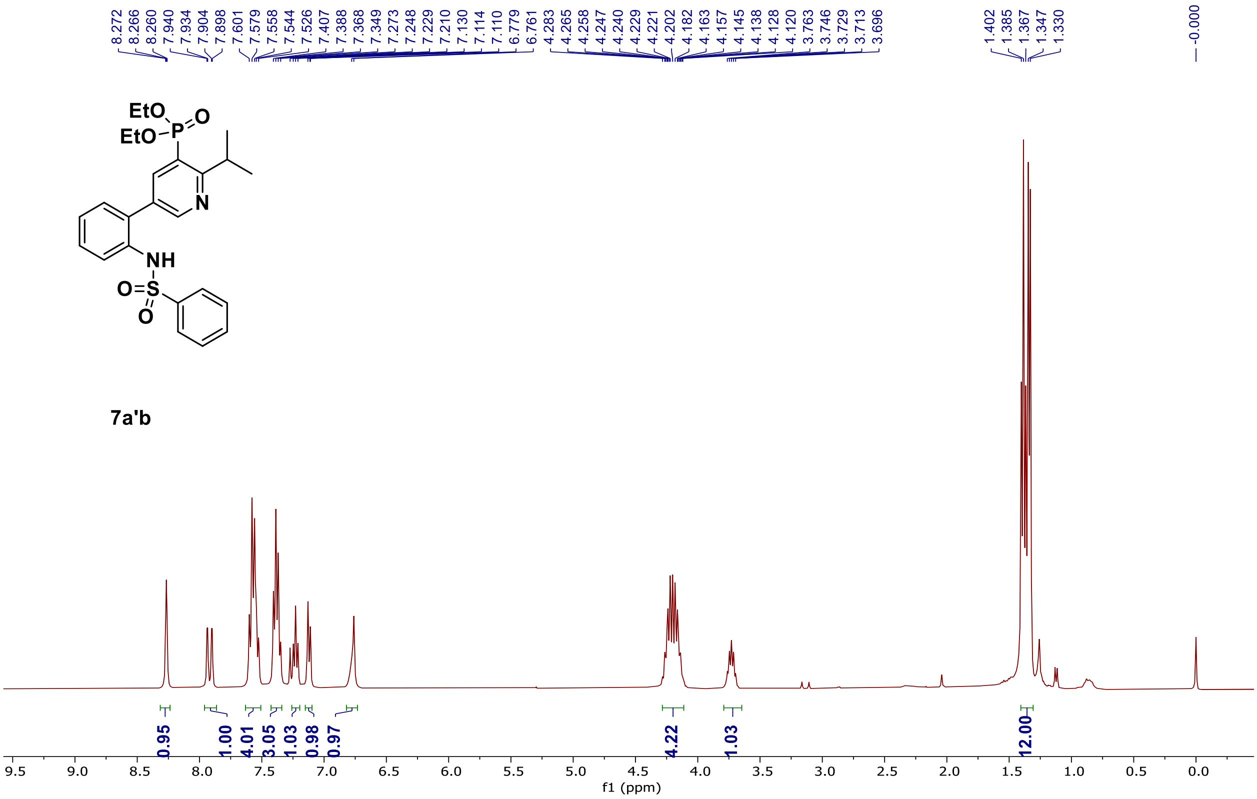

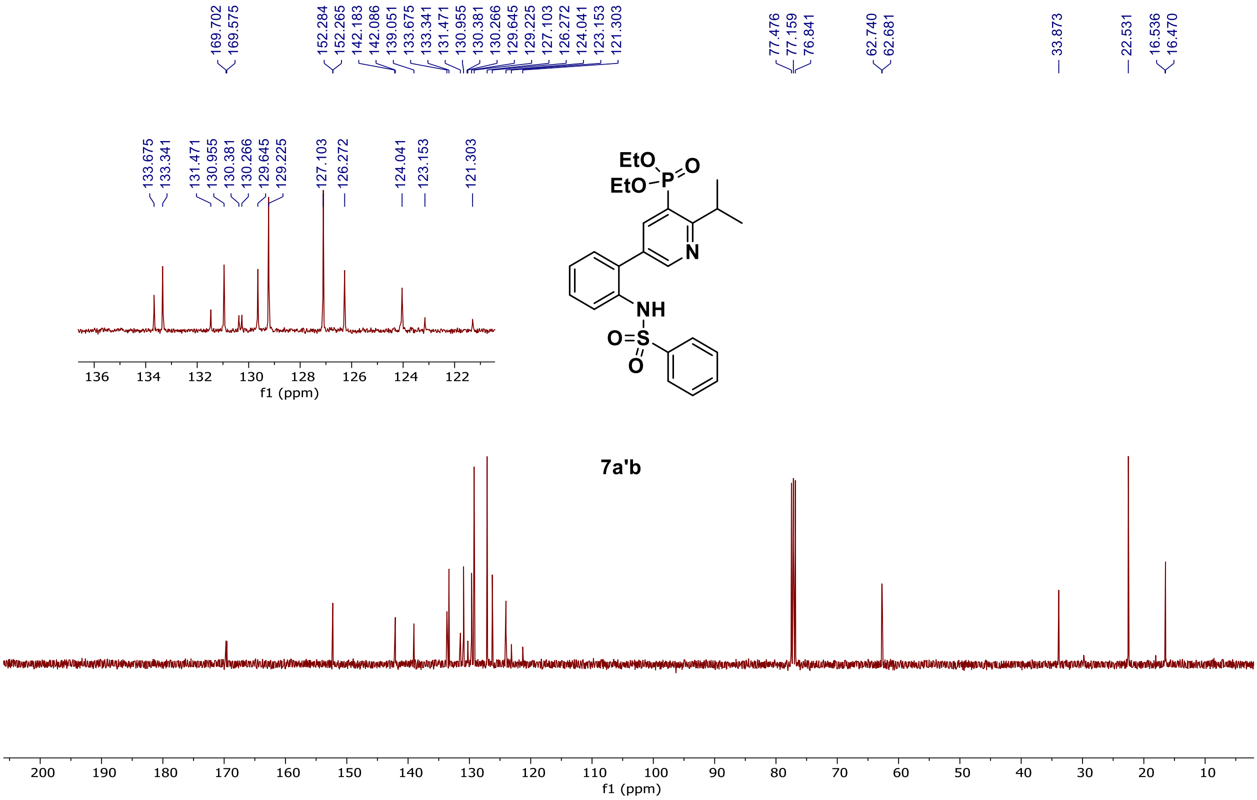

^{1}H and ^{13}C NMR Spectrum of **7a'b**in $CDCl_3$

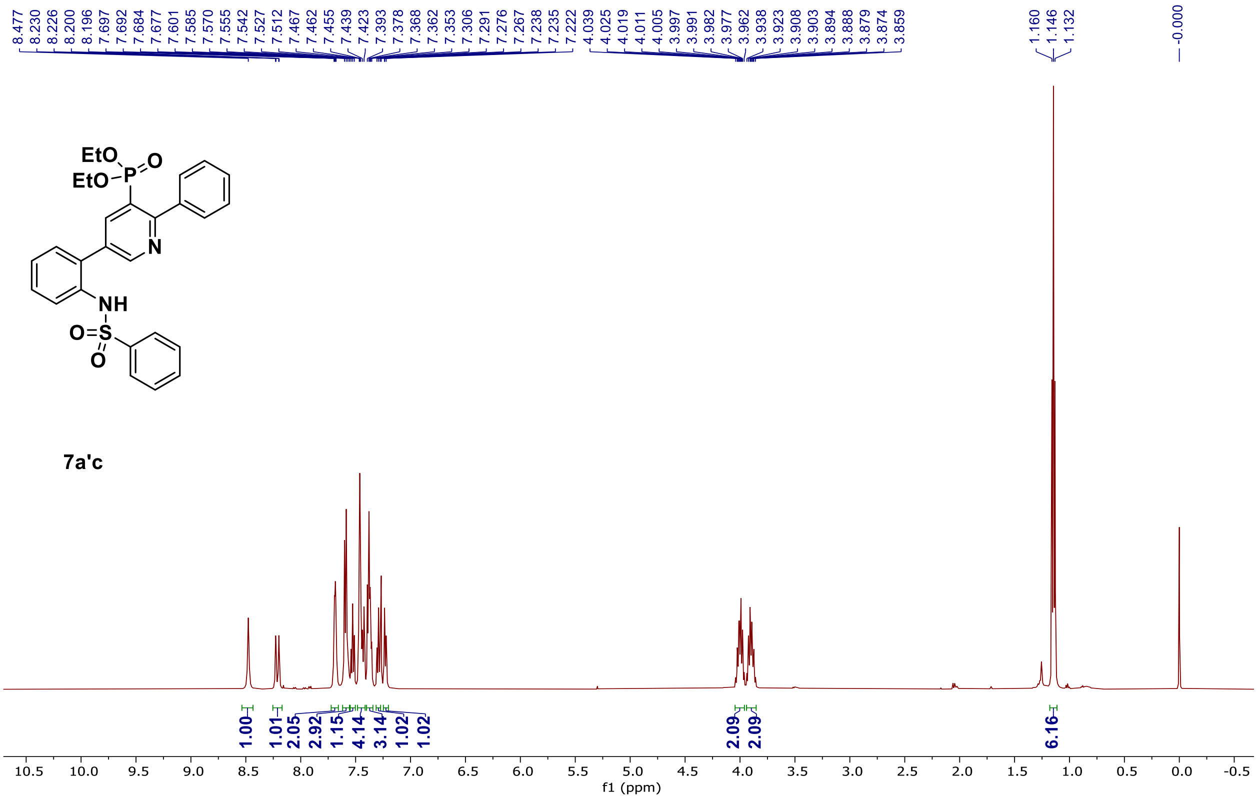

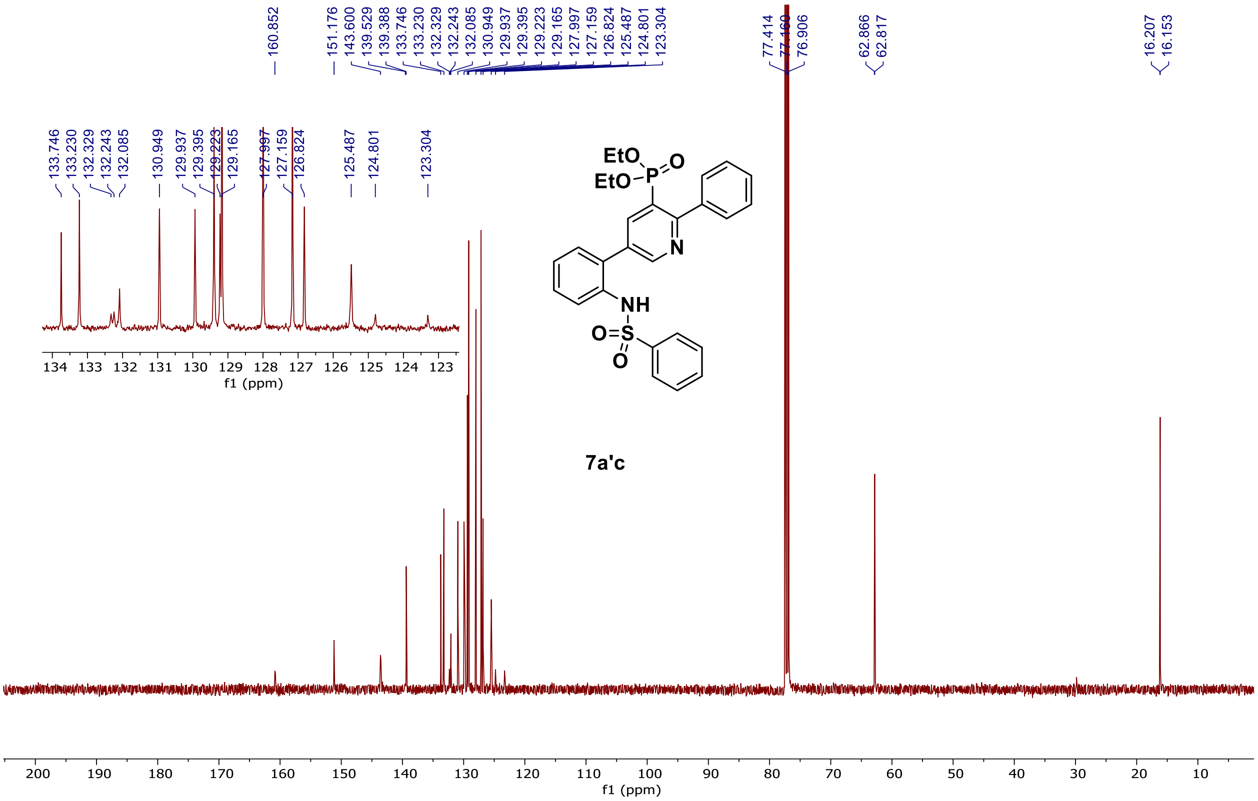

^{1}H and ^{13}C NMR Spectrum of **7a'c** in $CDCl_3$

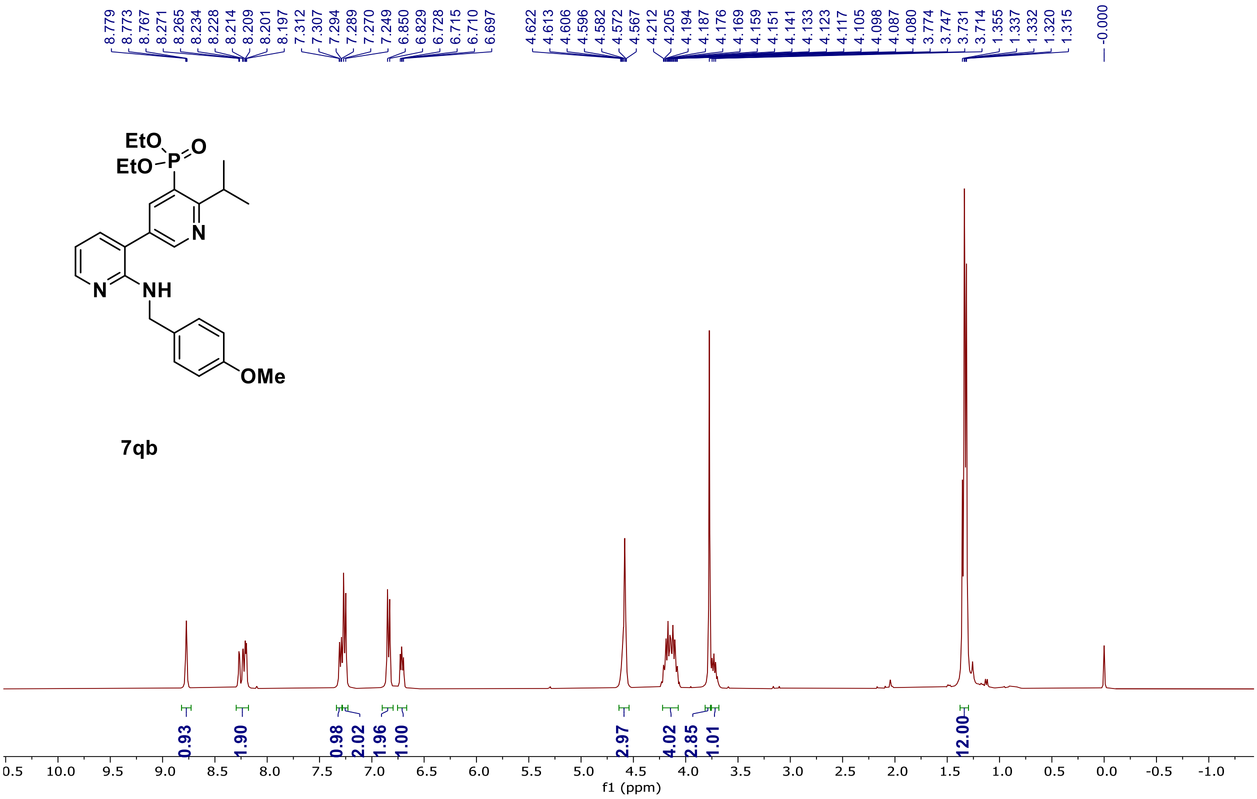

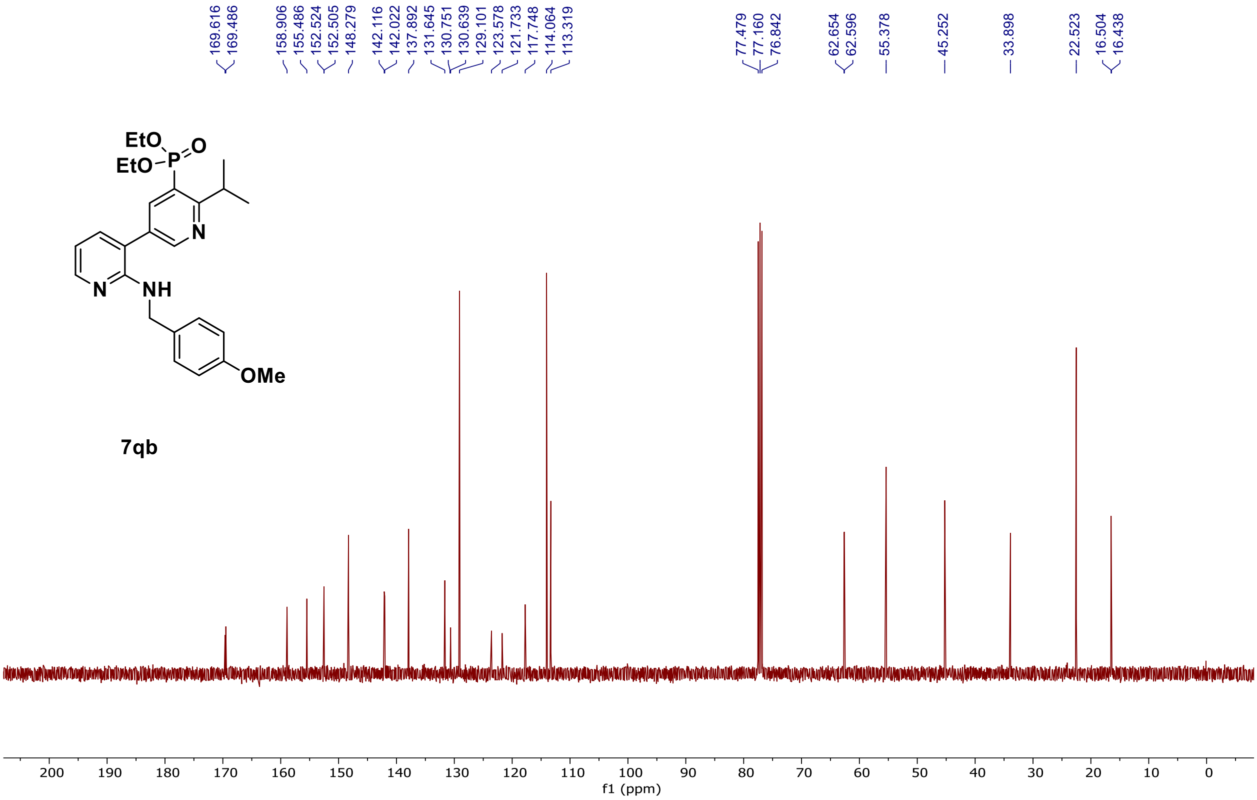

^{1}H and ^{13}C NMR Spectrum of **7qb** in $CDCl_3$

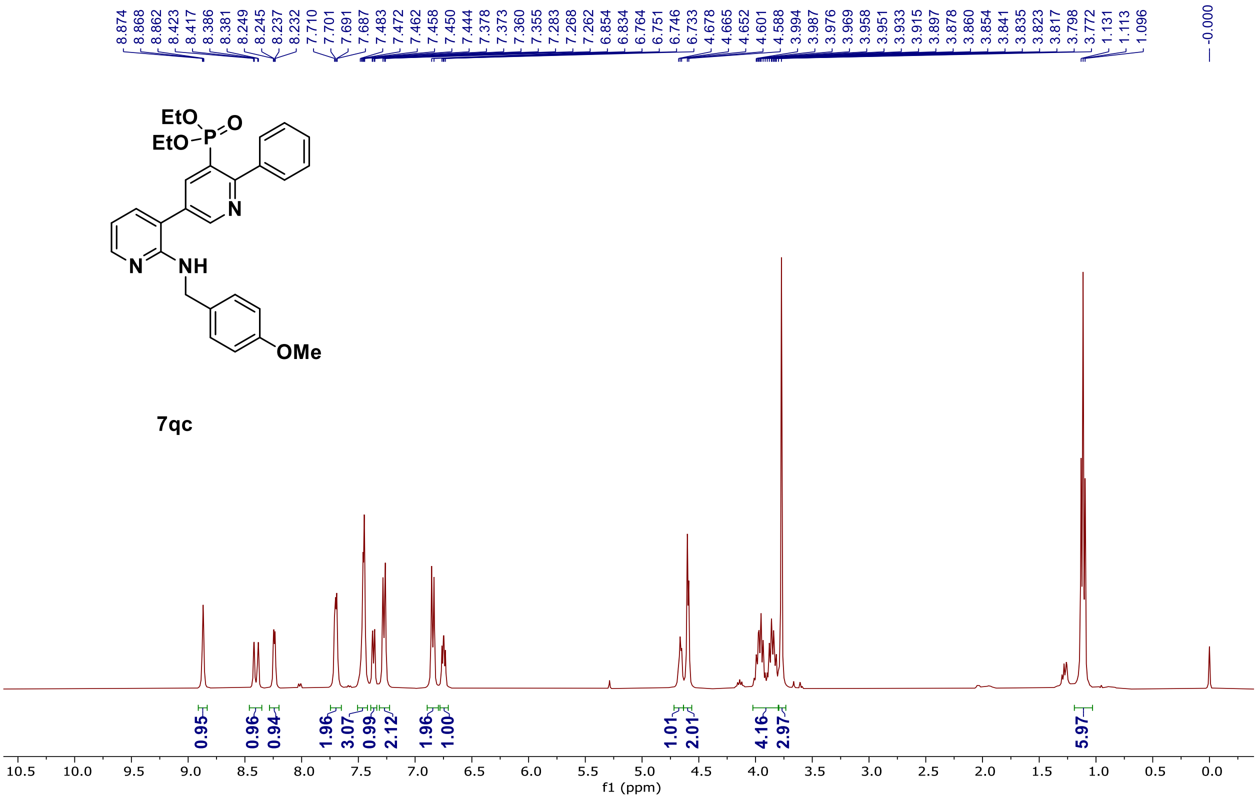

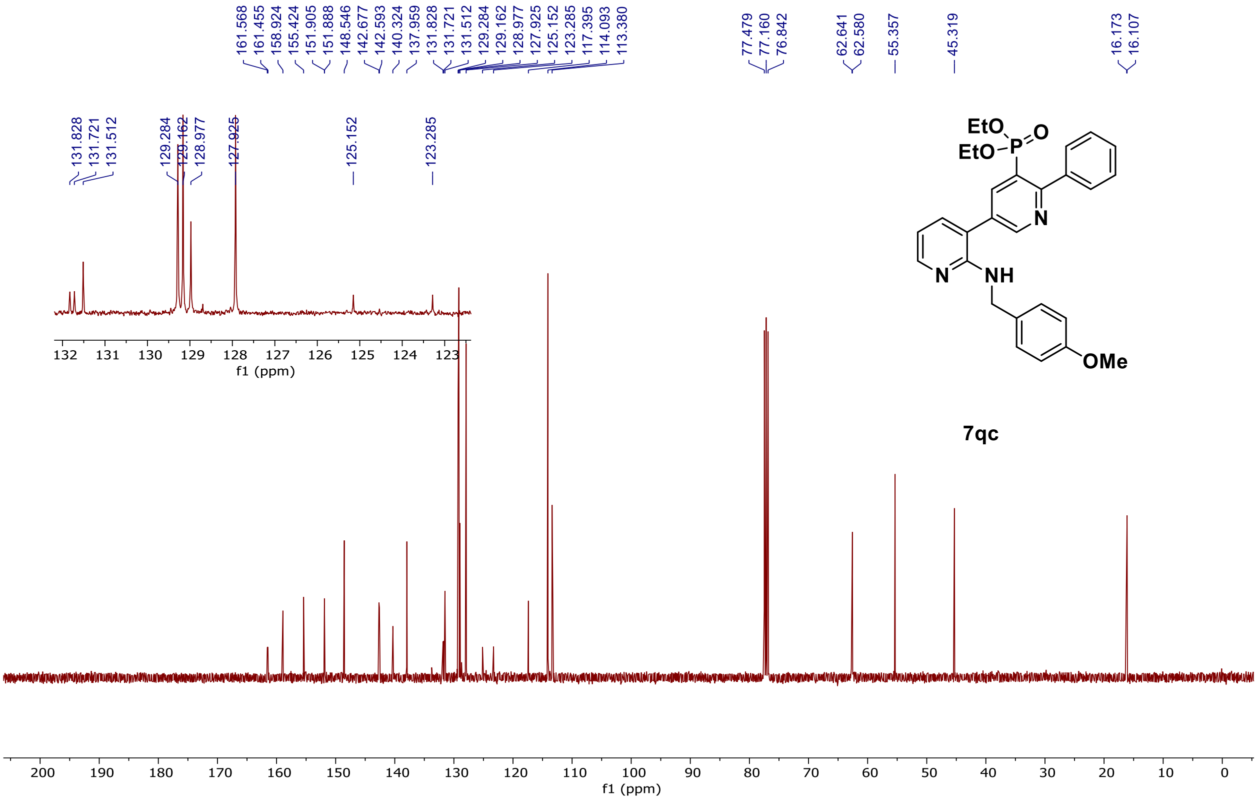

^{1}H and ^{13}C NMR Spectrum of **7qc** in $CDCl_3$

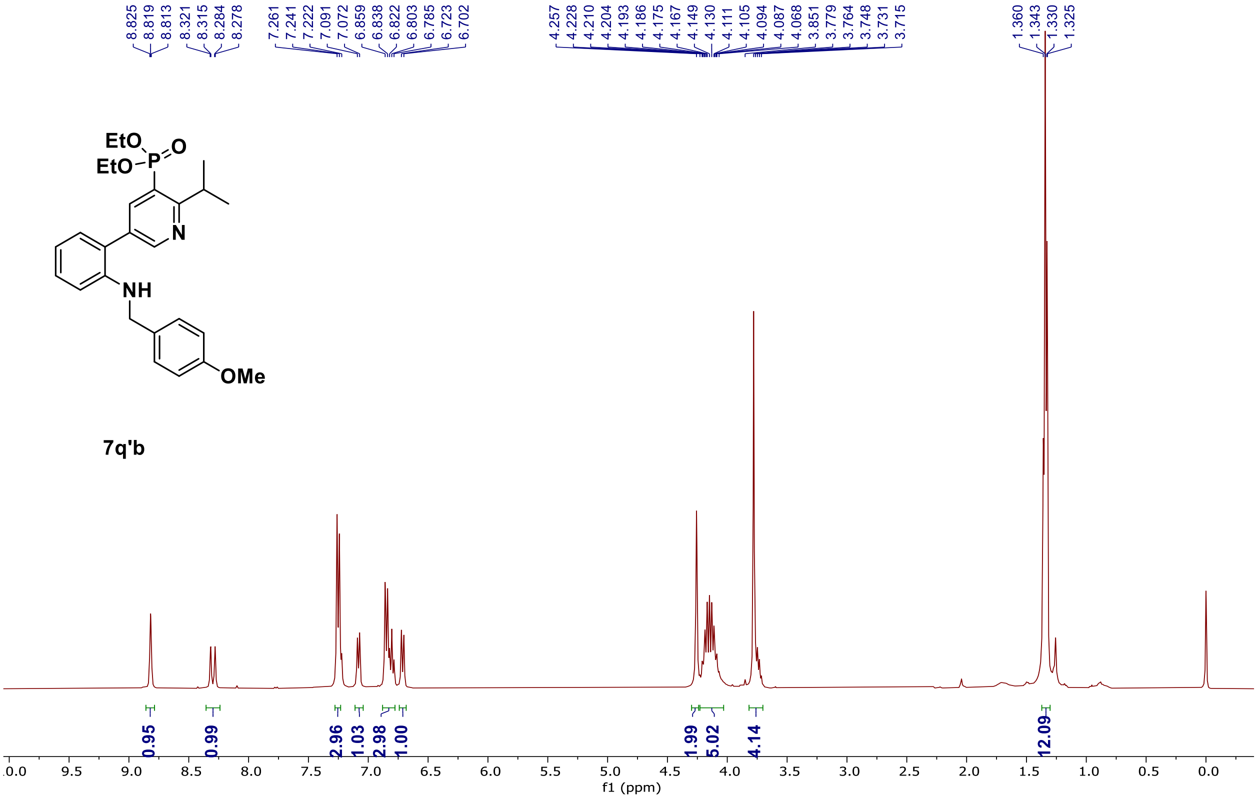

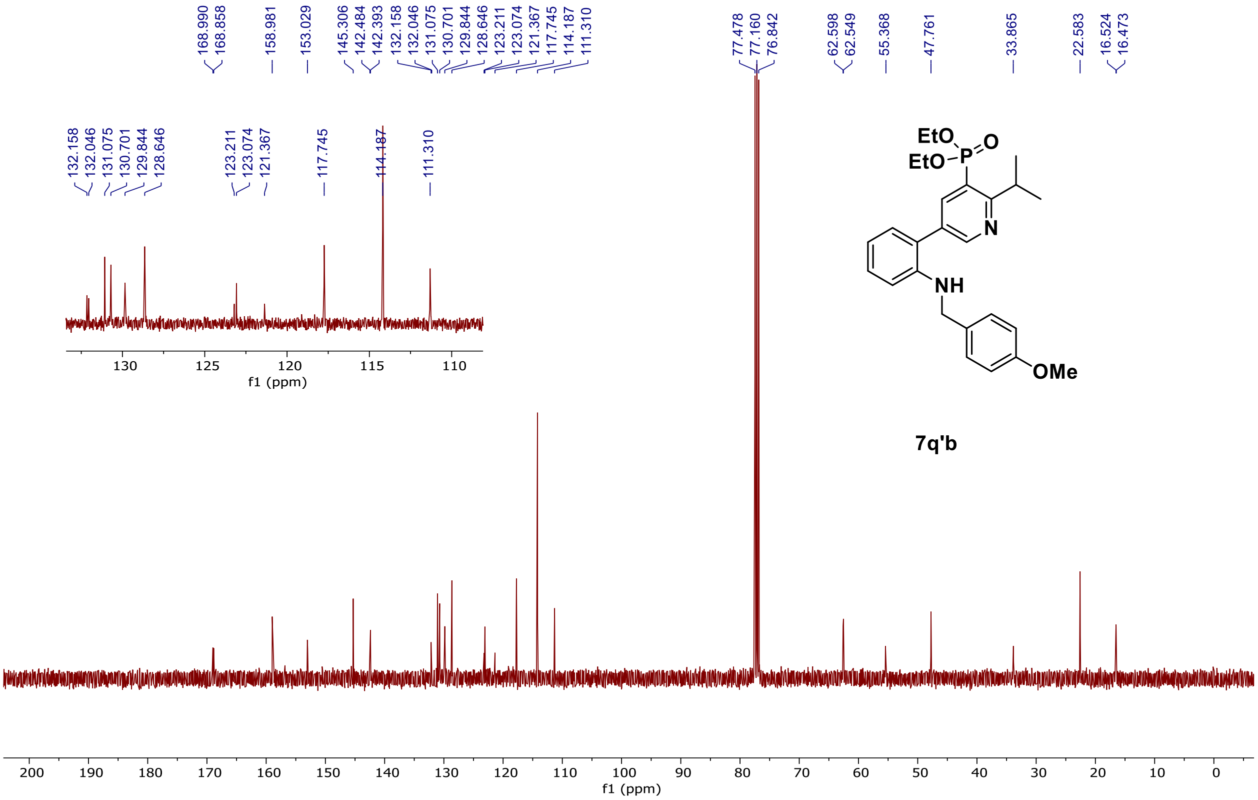

^{1}H and ^{13}C NMR Spectrum of **7q'b** in $CDCl_3$

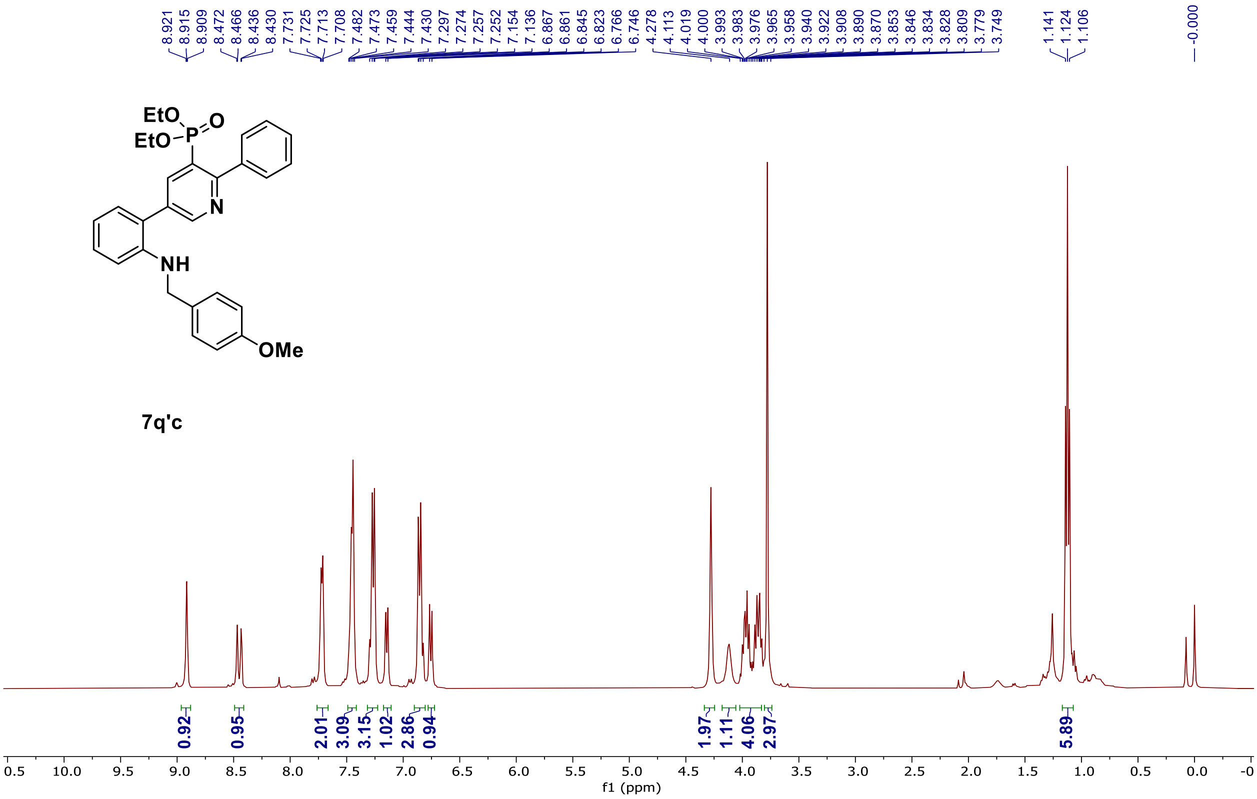

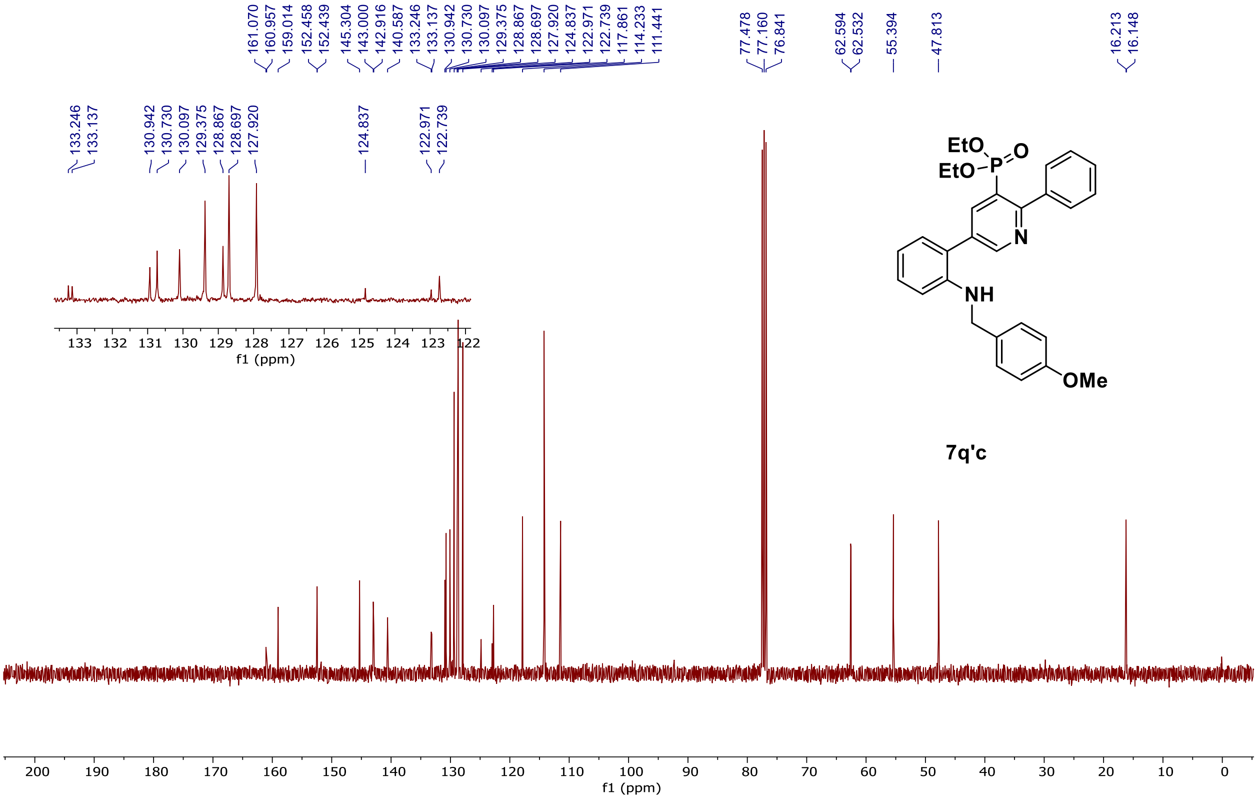

^{1}H and ^{13}C NMR Spectrum of **7q'c** in $CDCl_3$

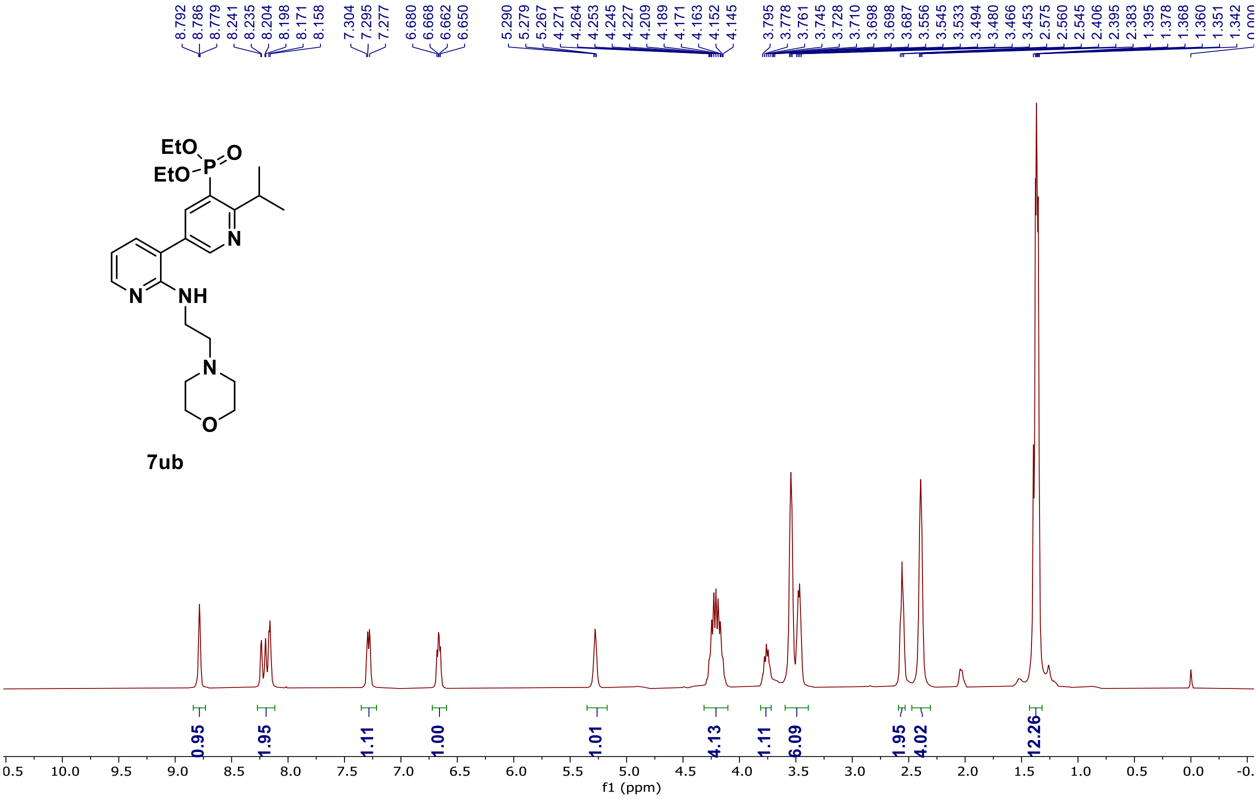

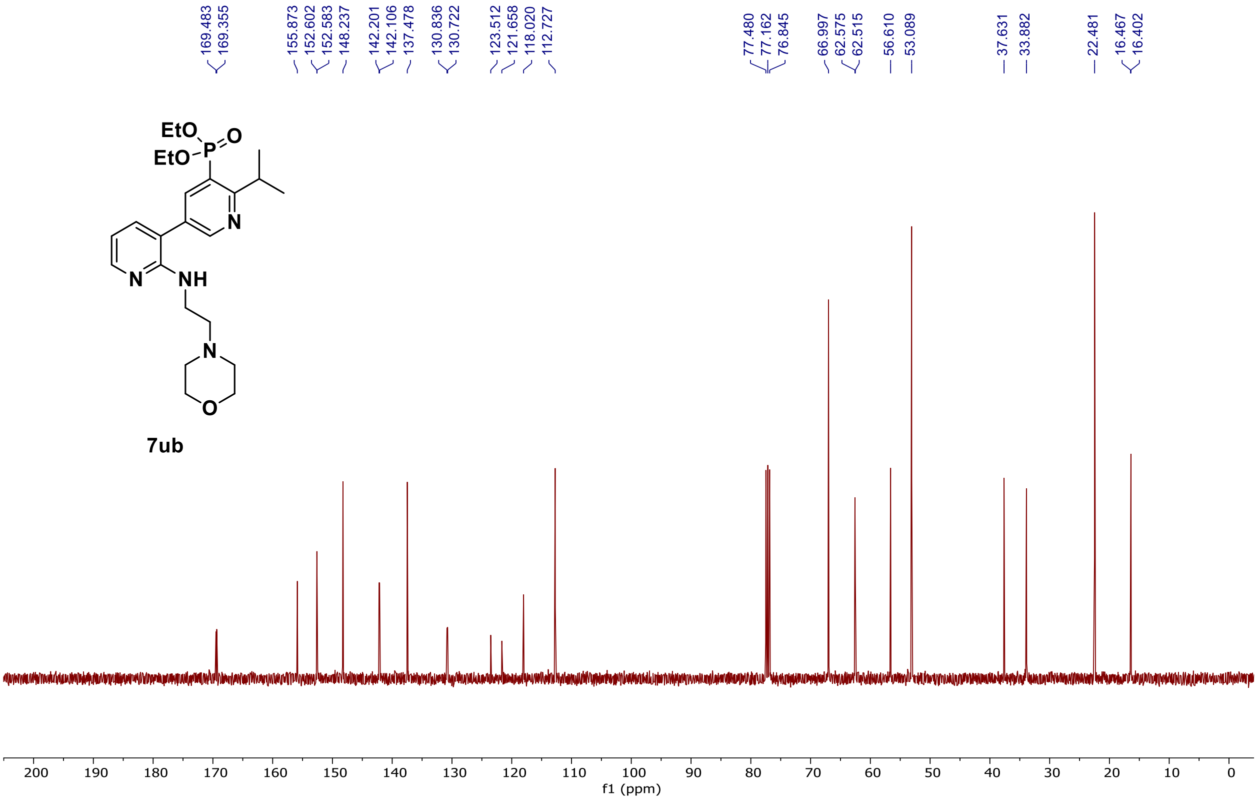

^{1}H and ^{13}C NMR Spectrum of **7ub** in $CDCl_3$

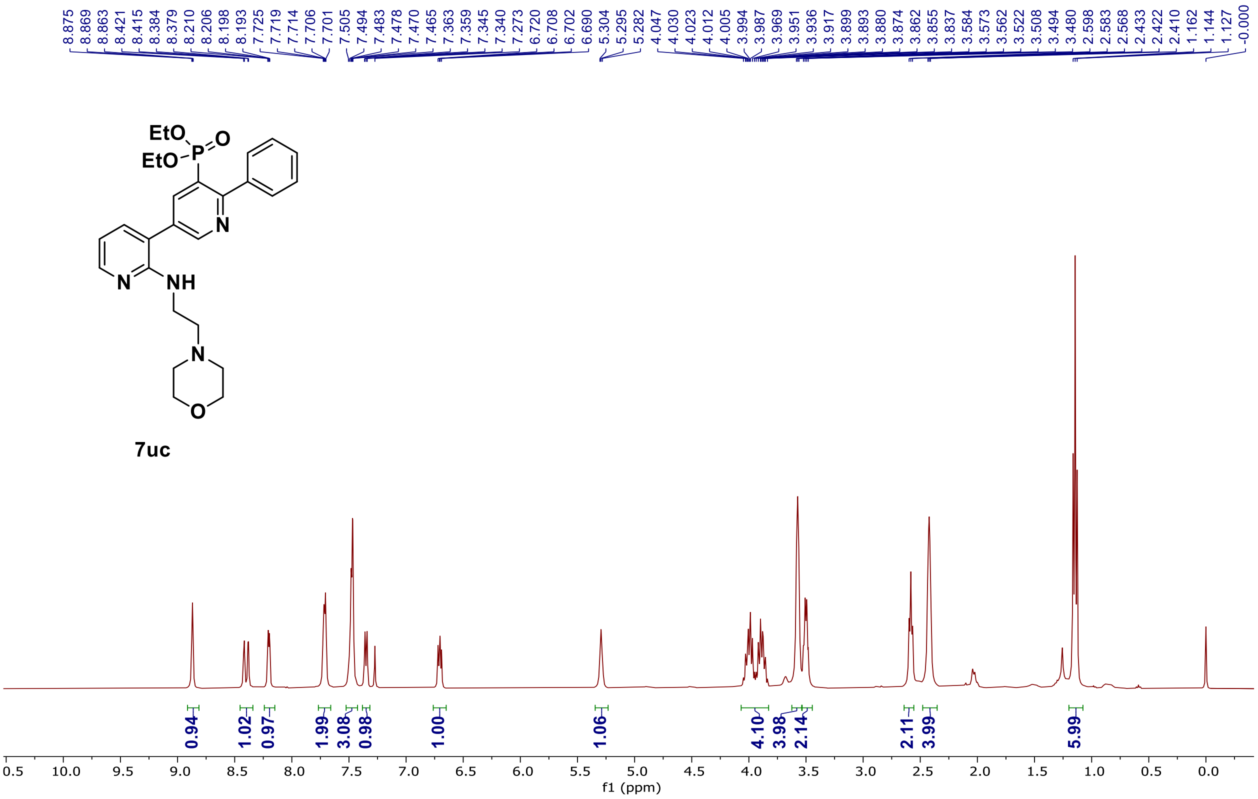

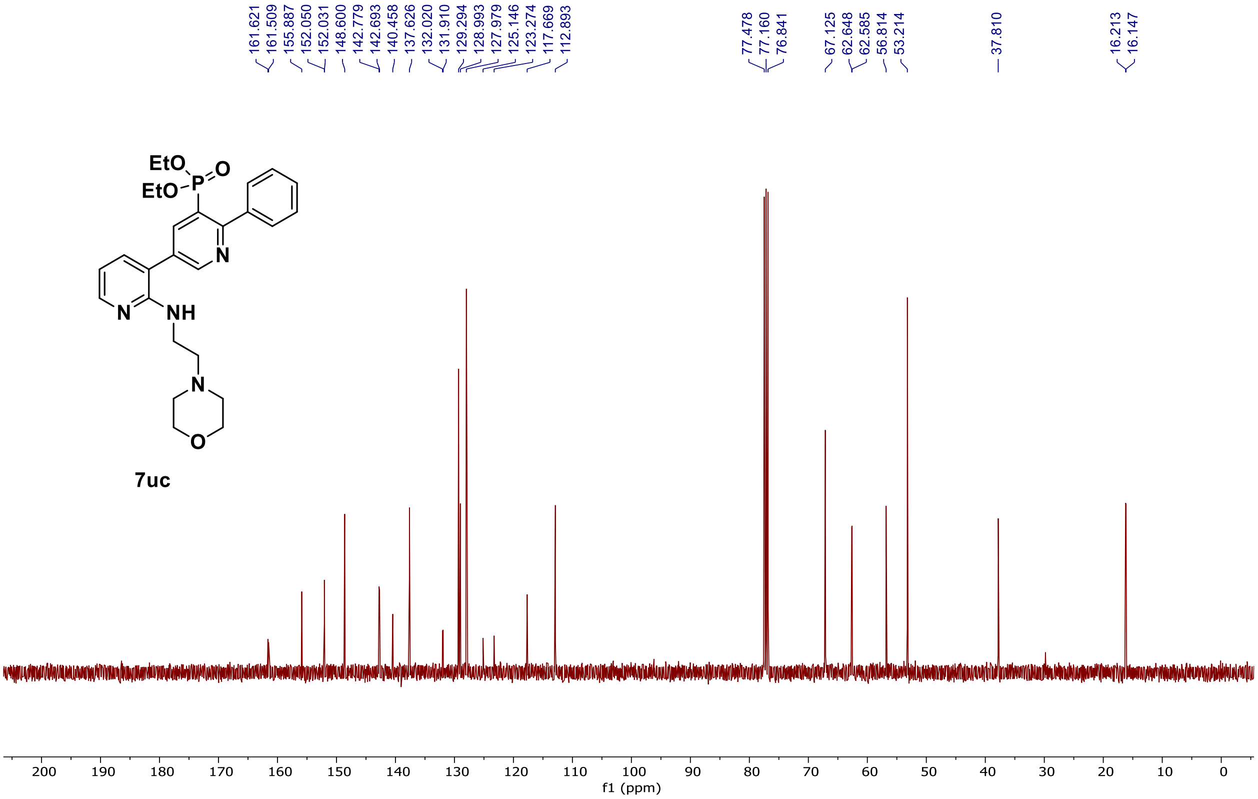

^{1}H and ^{13}C NMR Spectrum of **7uc i**n $CDCl_3$

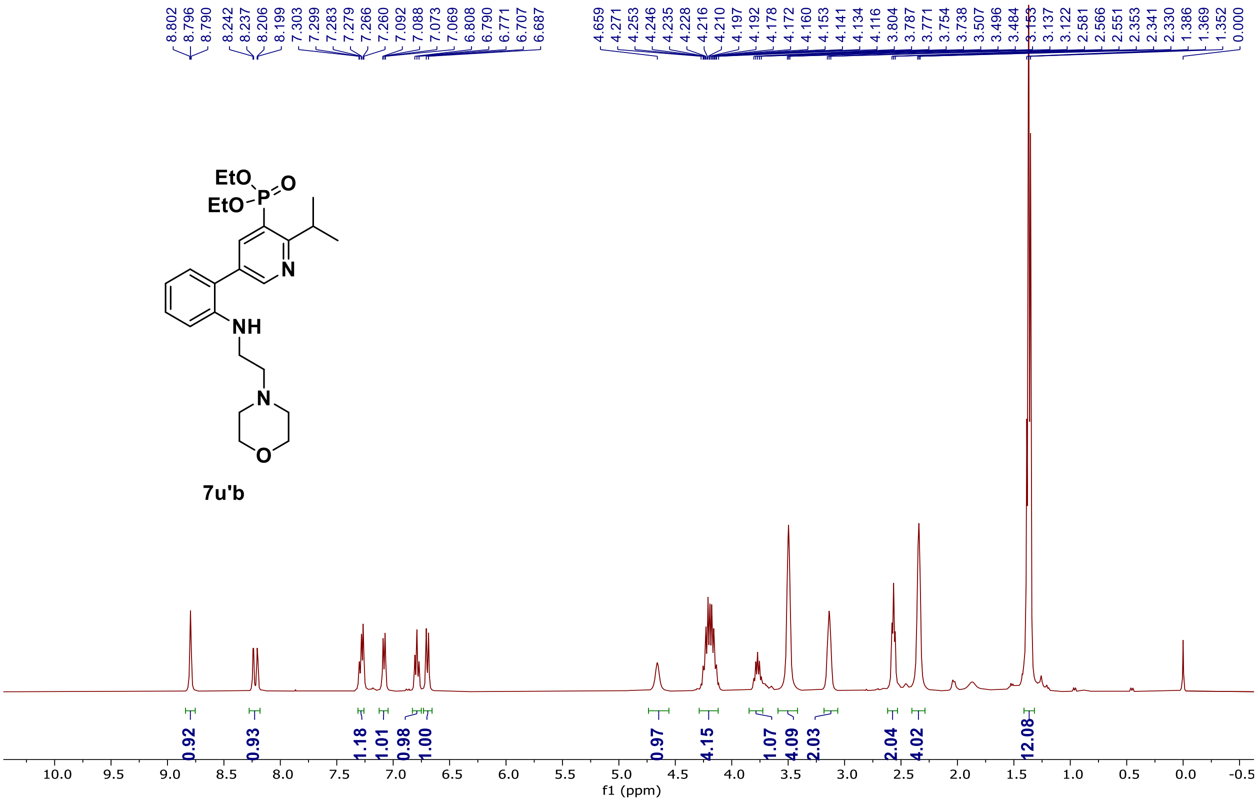

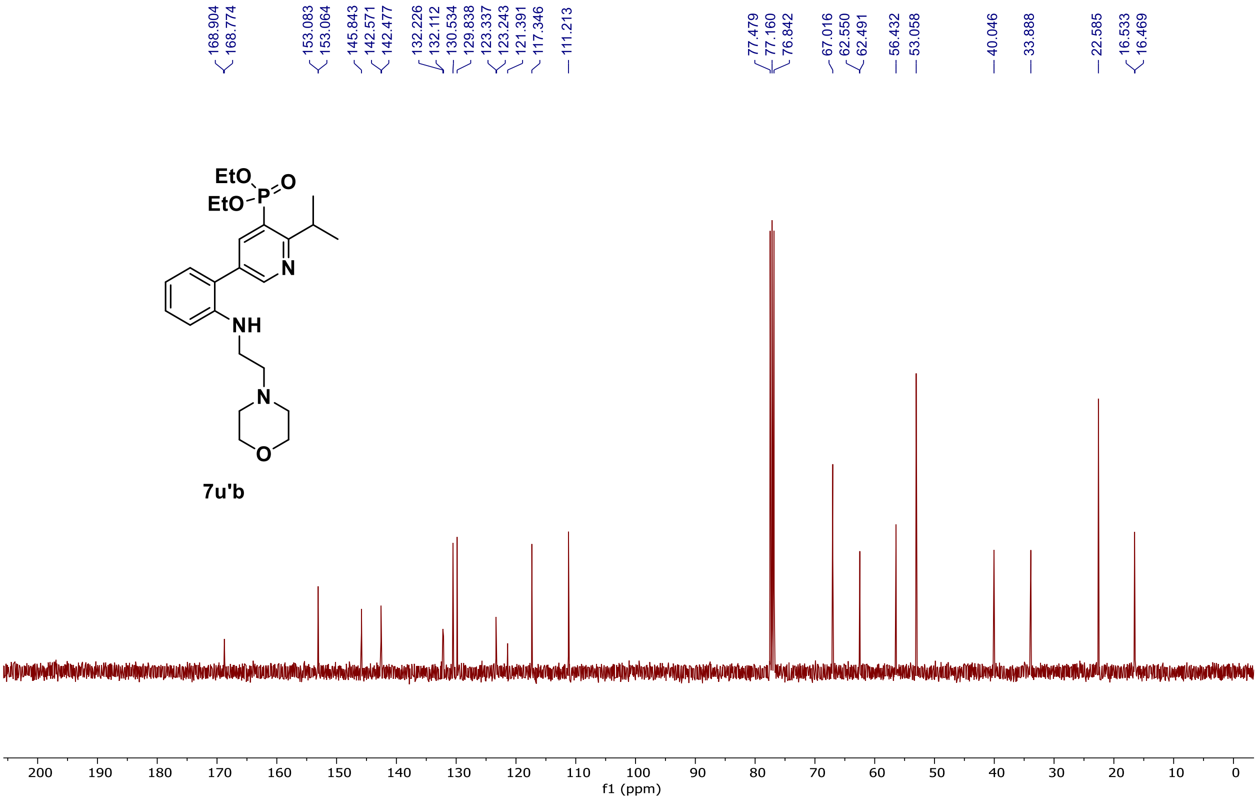

^{1}H and ^{13}C NMR Spectrum of **7u'b** in $CDCl_3$

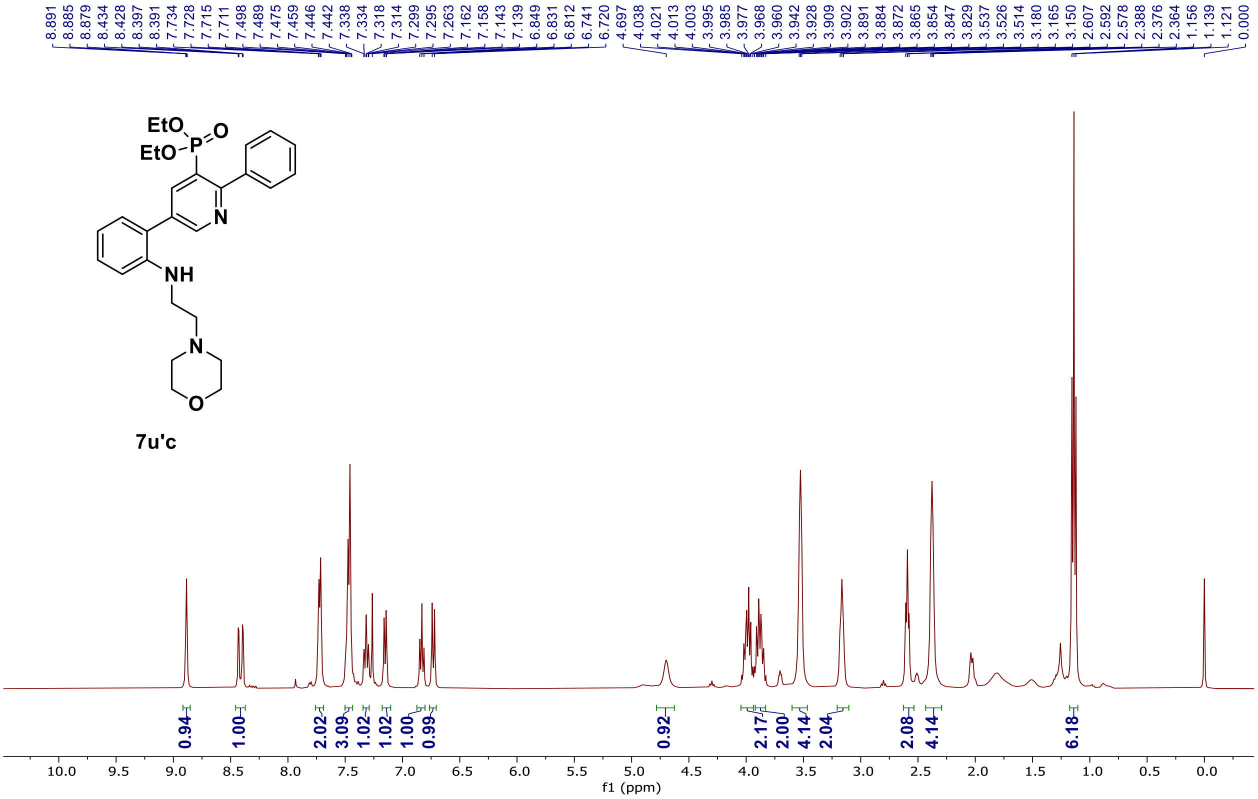

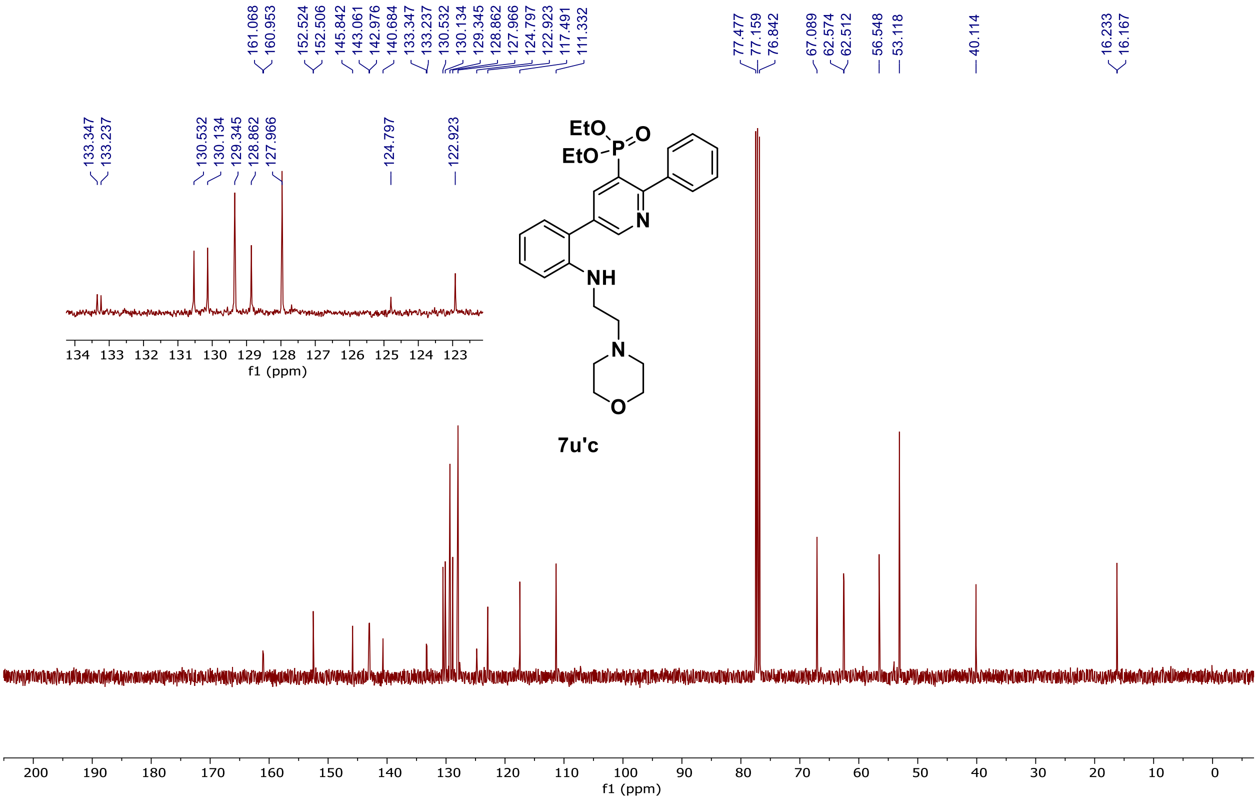

^{1}H and ^{13}C NMR Spectrum of **7u'c** in $CDCl_3$

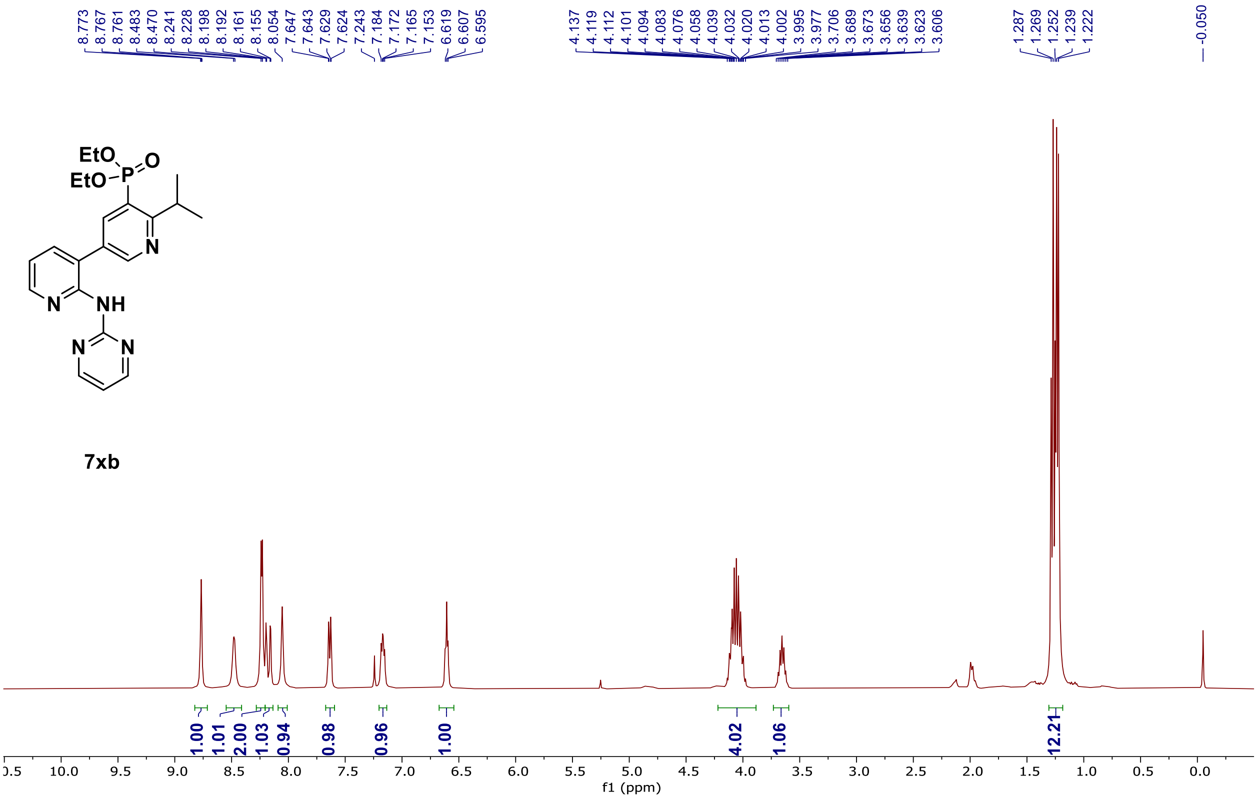

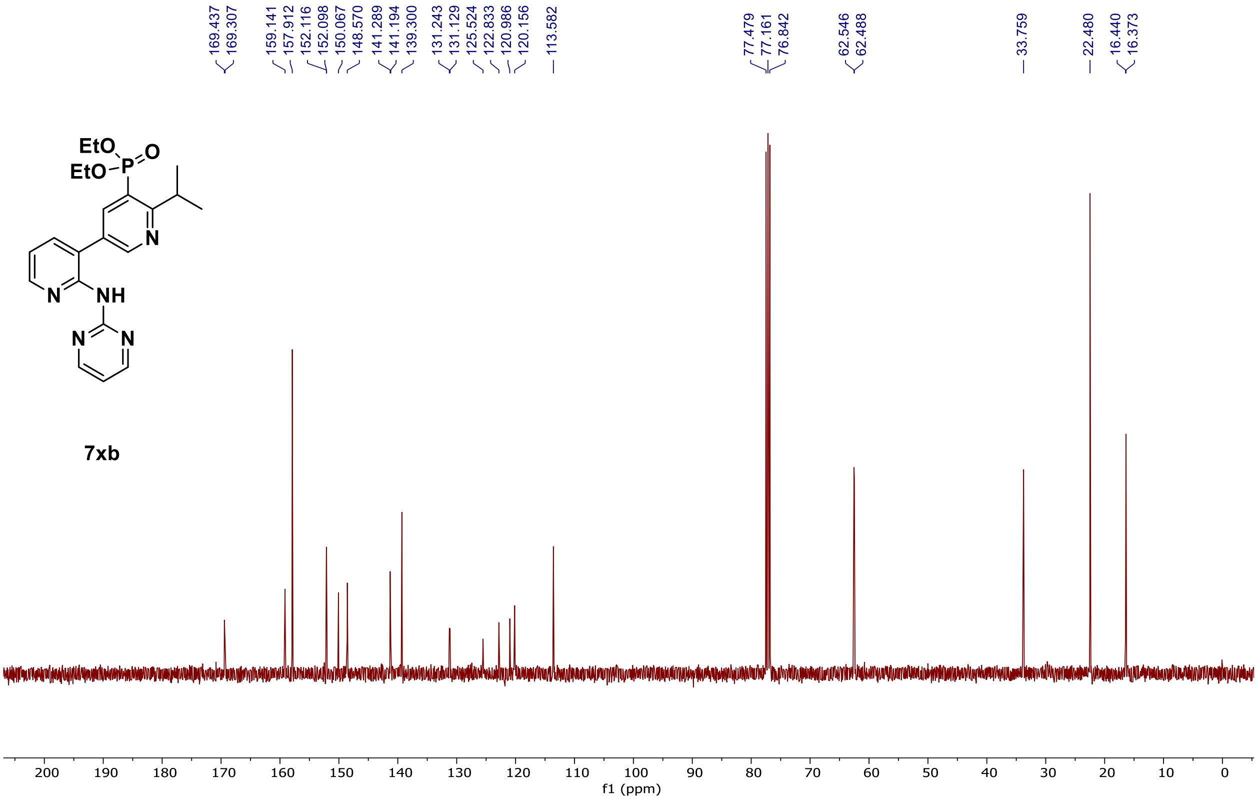

^{1}H and ^{13}C NMR Spectrum of **7xb** in $CDCl_3$

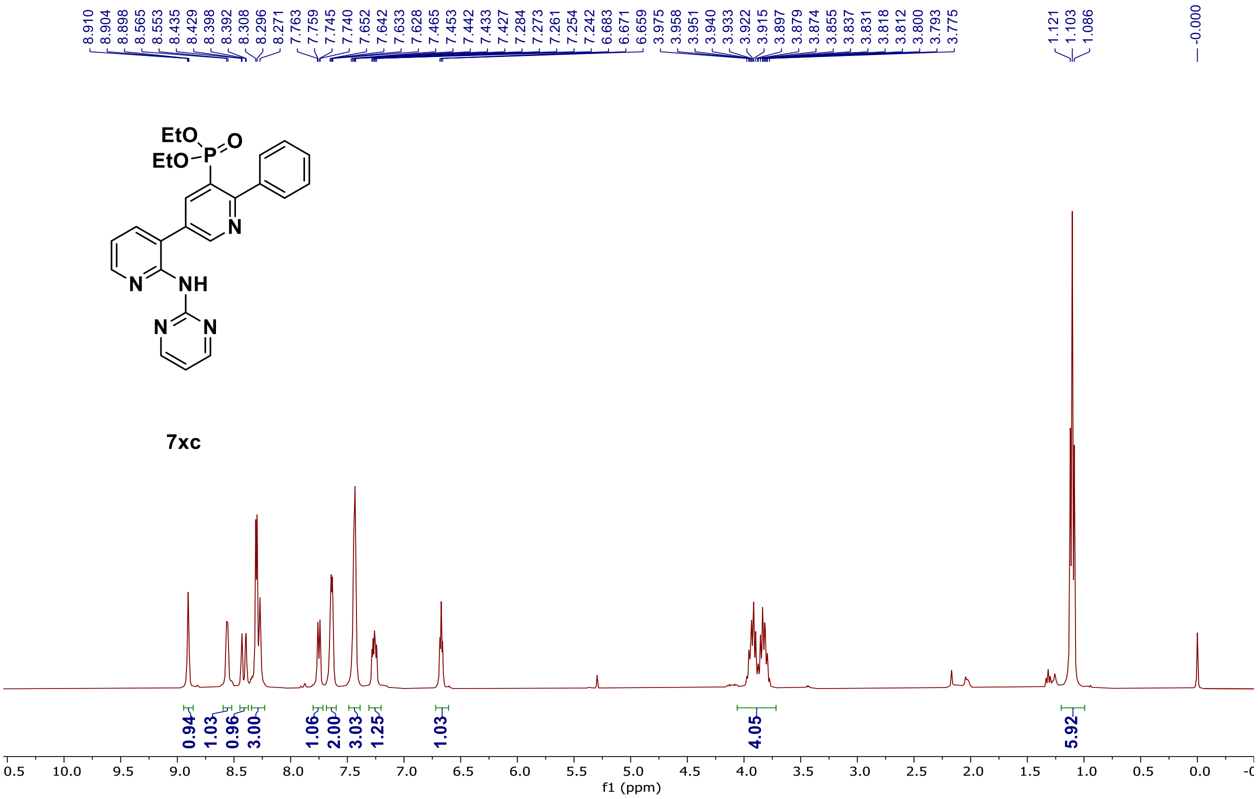

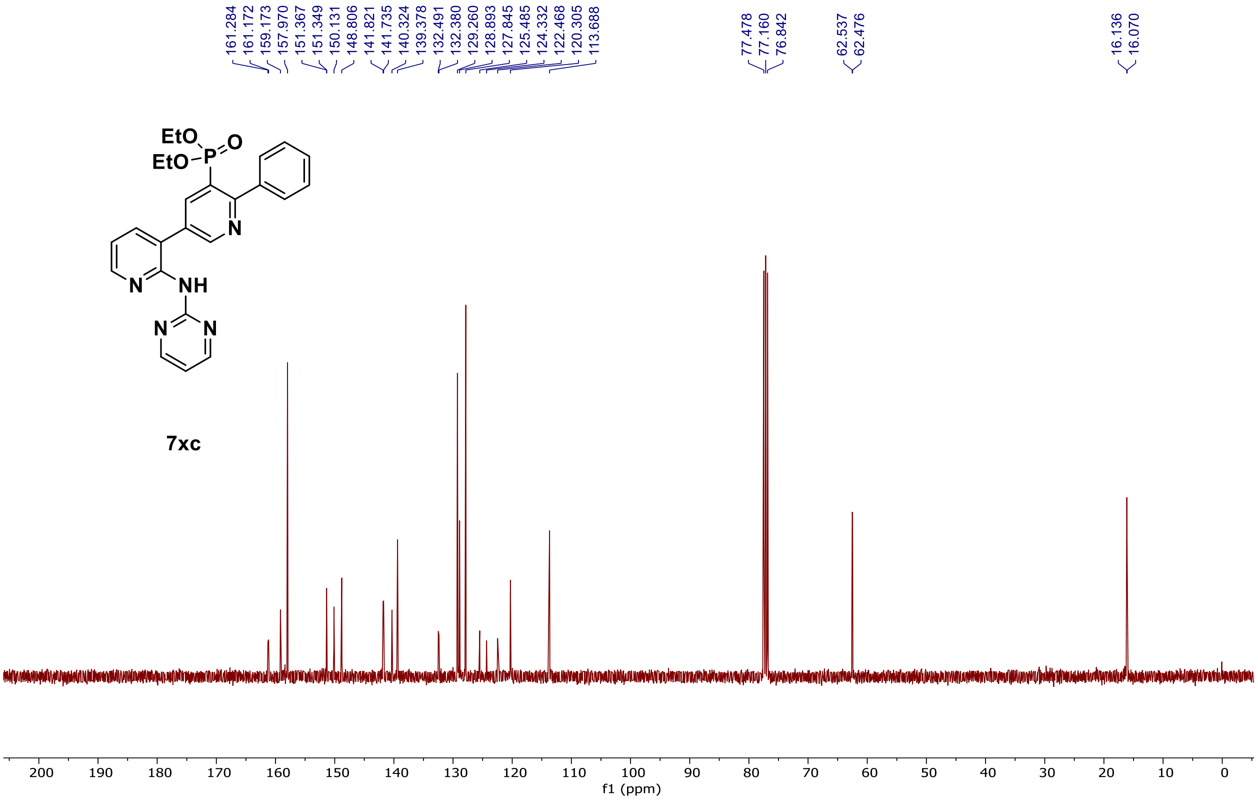

^{1}H and ^{13}C NMR Spectrum of **7xc** in $CDCl_3$

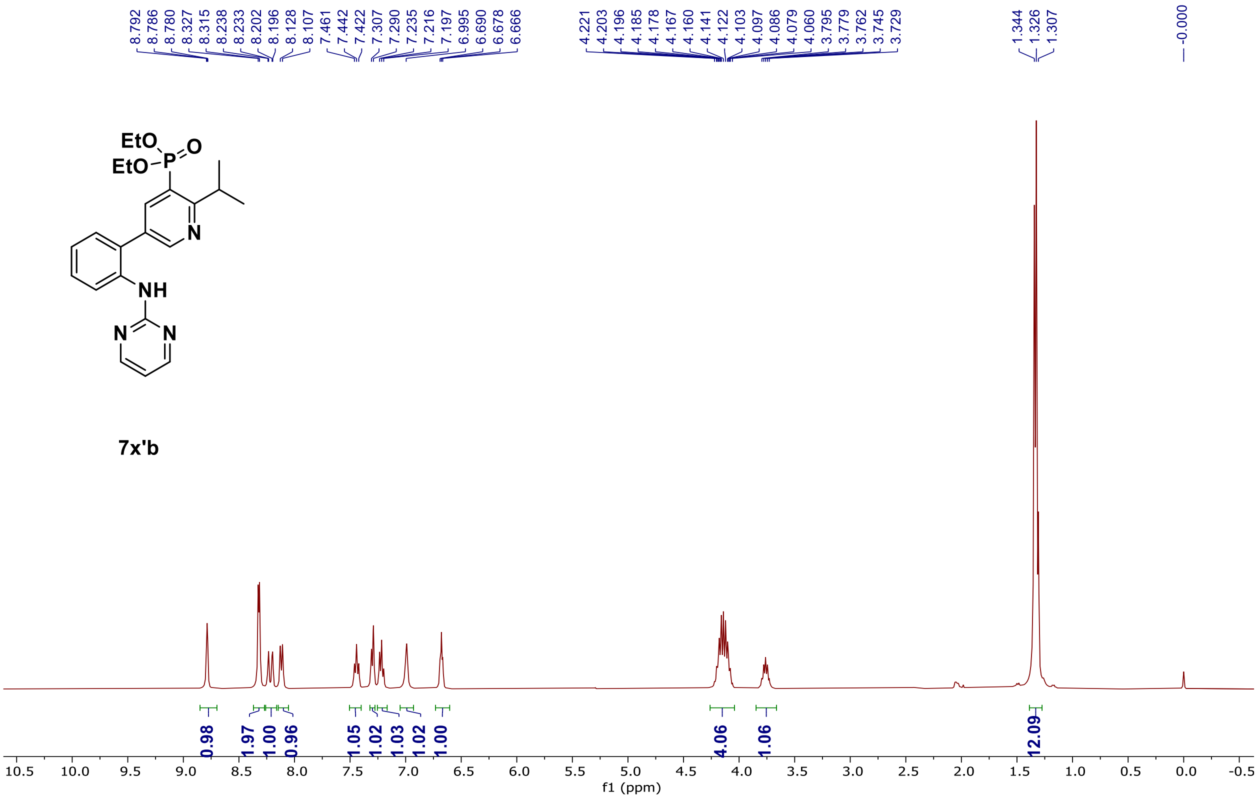

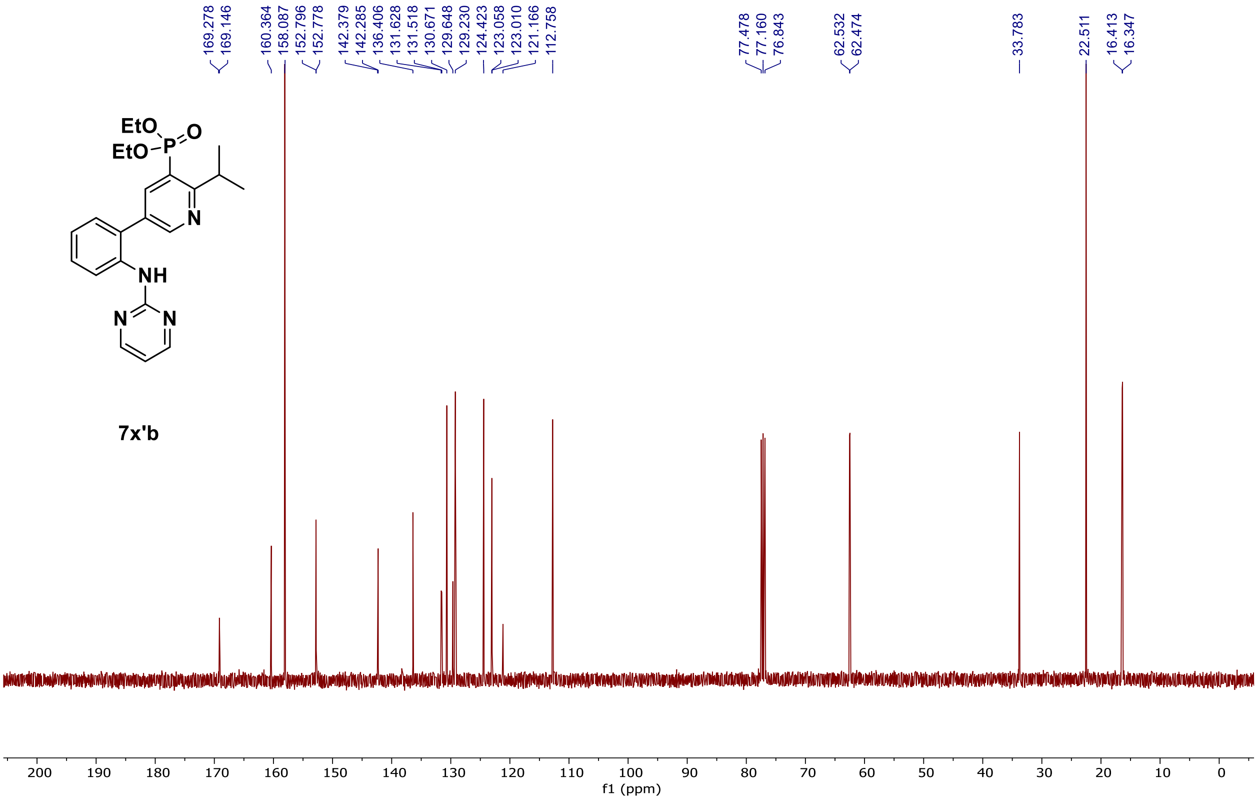

^{1}H and ^{13}C NMR Spectrum of **7x'b** in $CDCl_3$

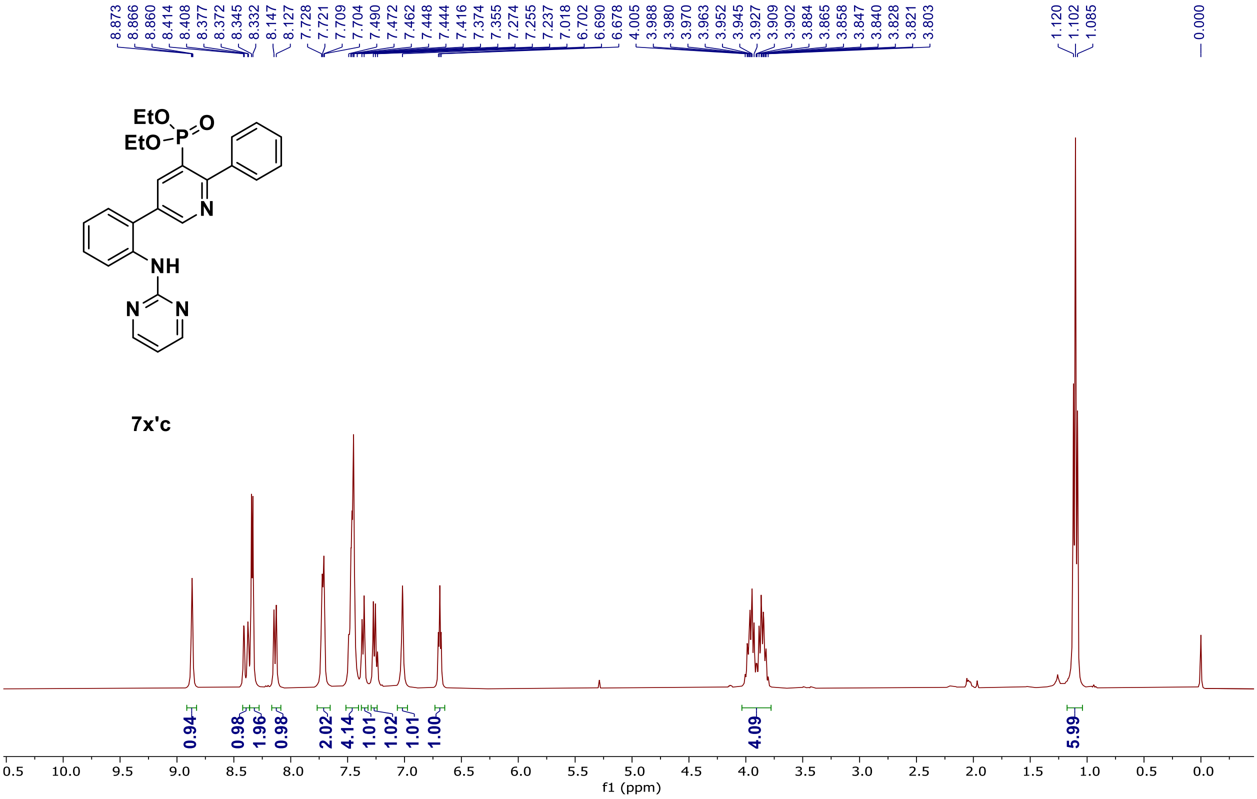

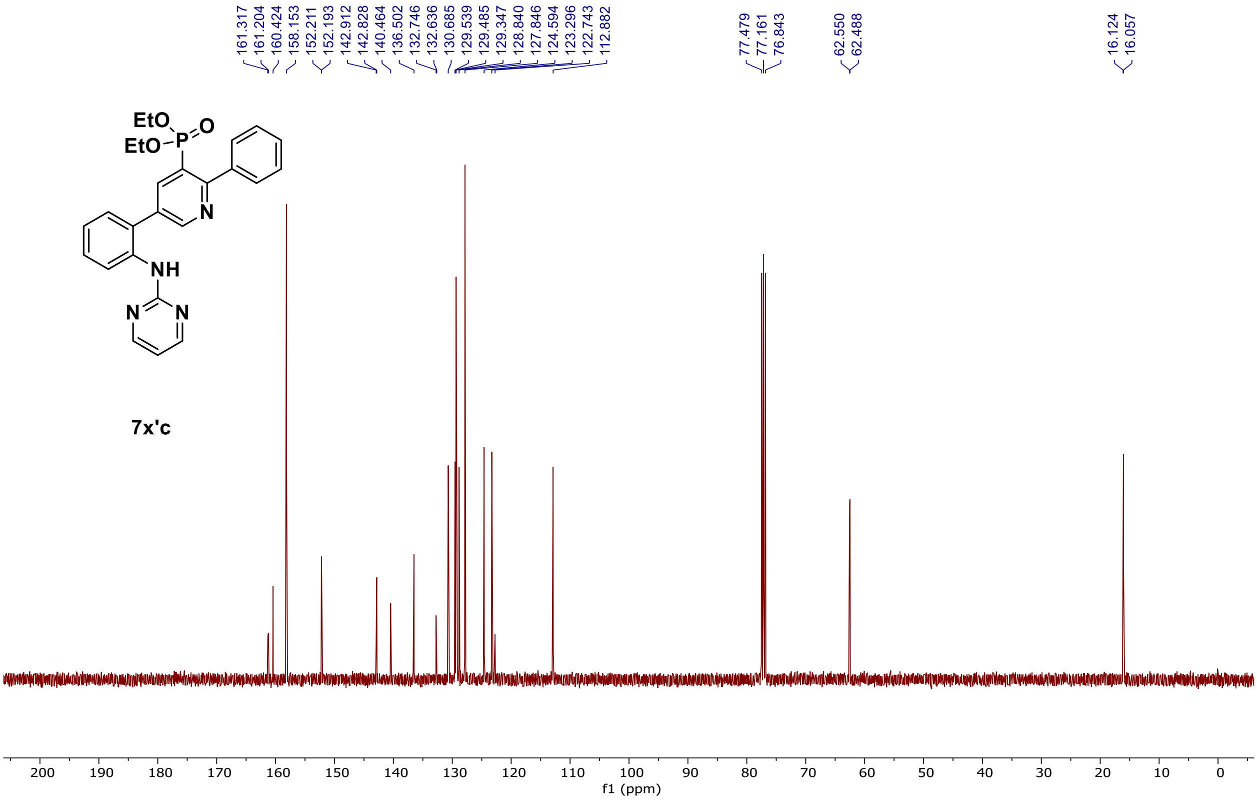

^{1}H and ^{13}C NMR Spectrum of **7x'c** in $CDCl_3$

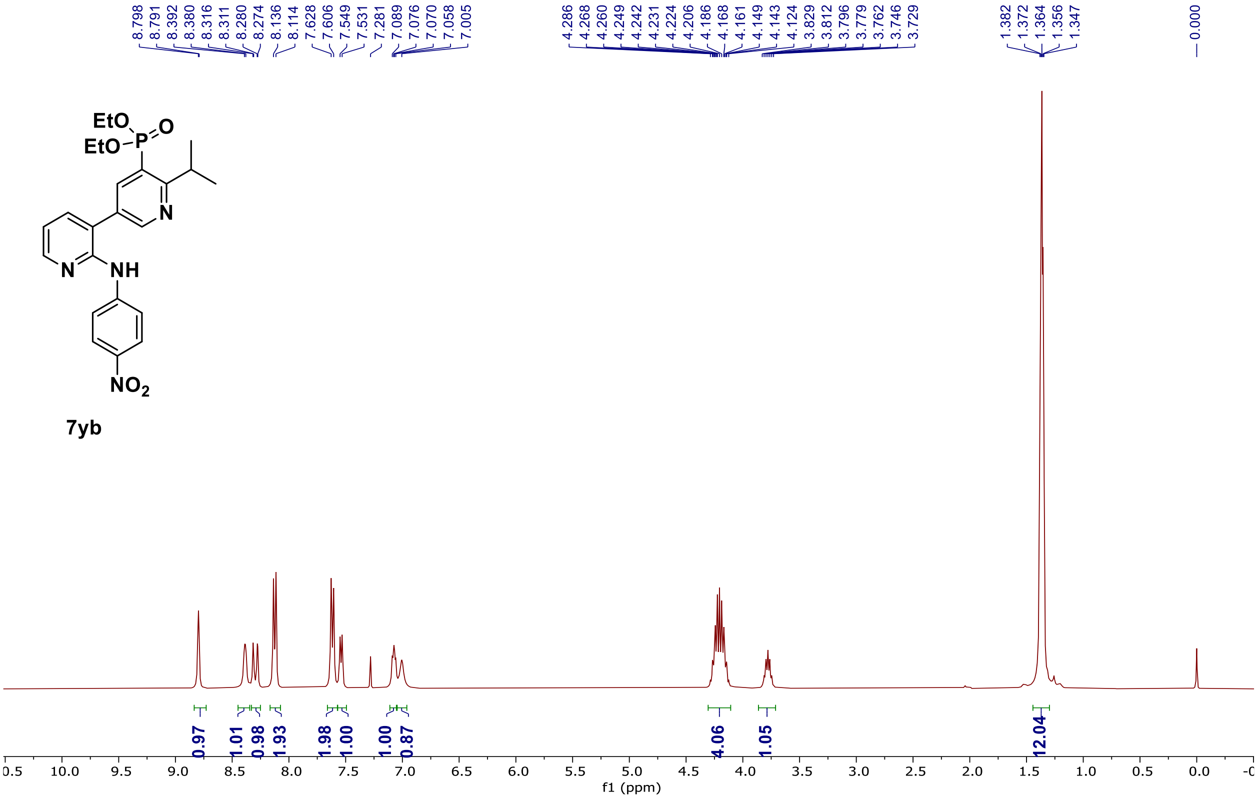

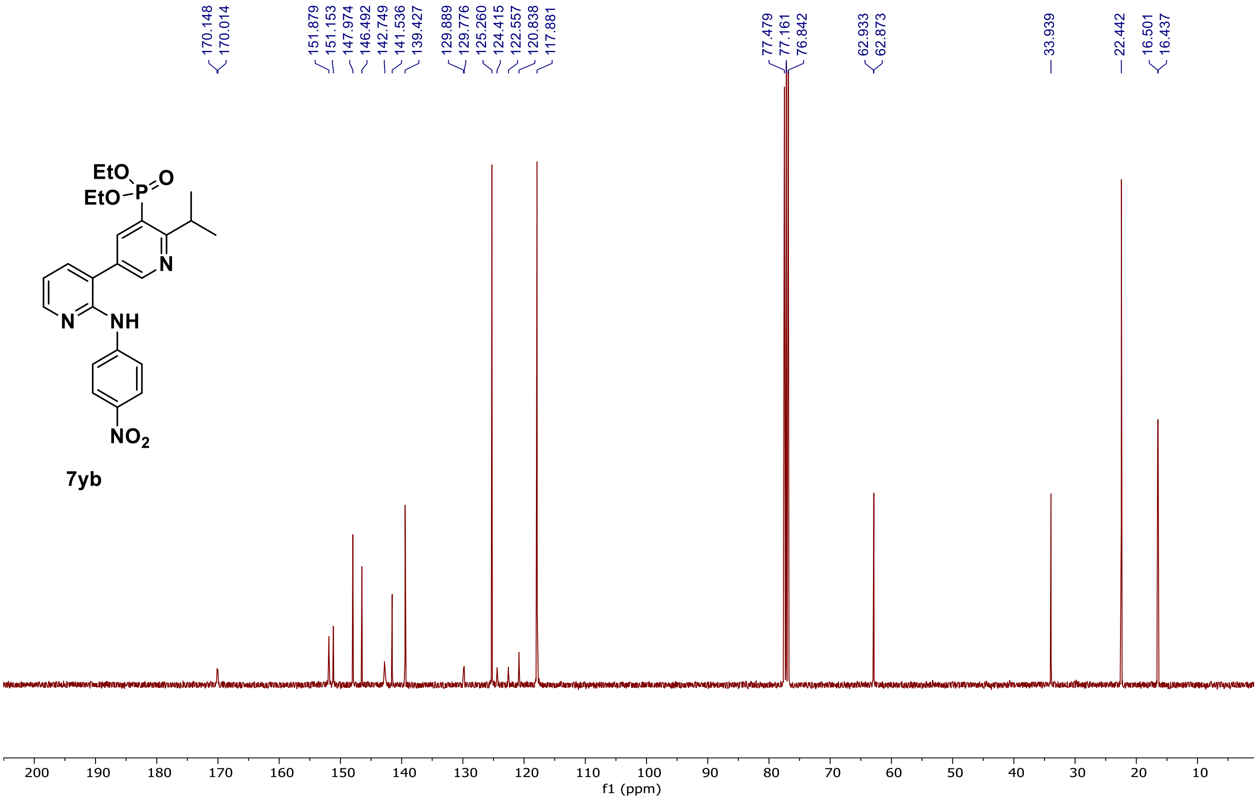

^{1}H and ^{13}C NMR Spectrum of **7yb** in $CDCl_3$

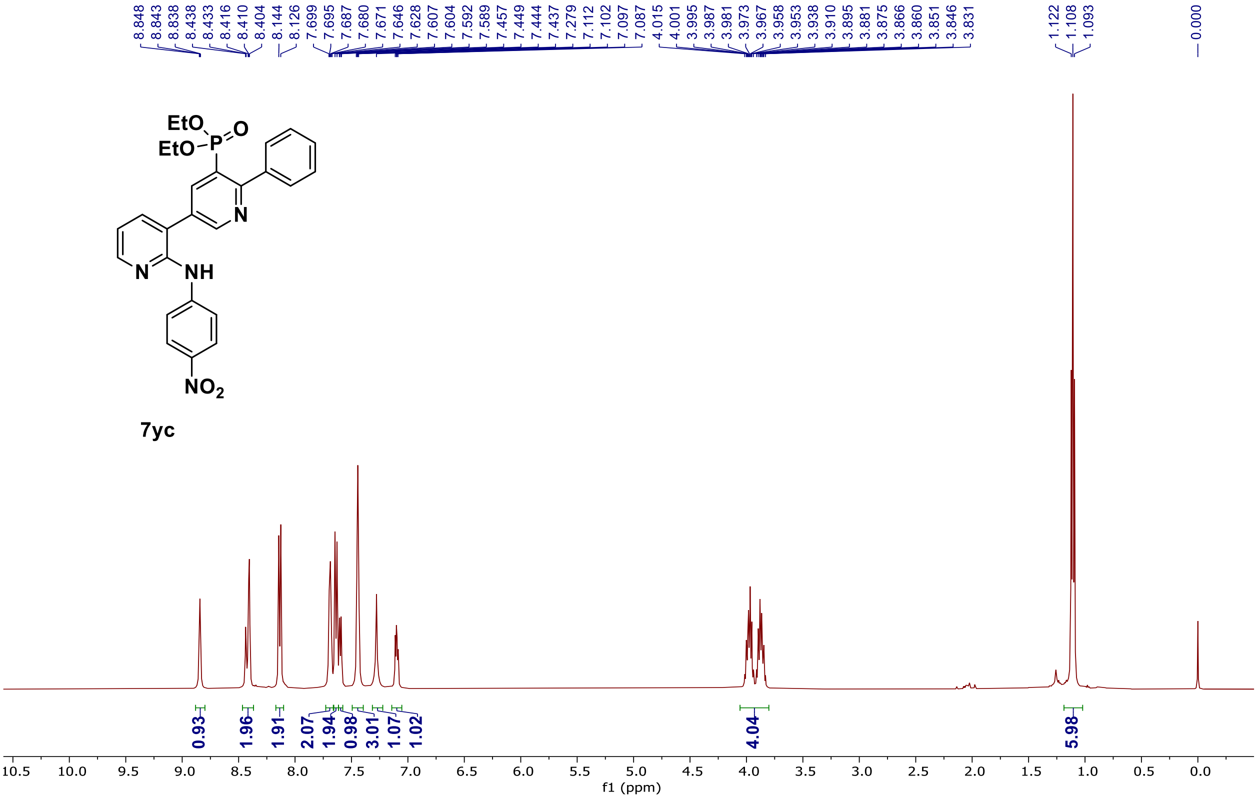

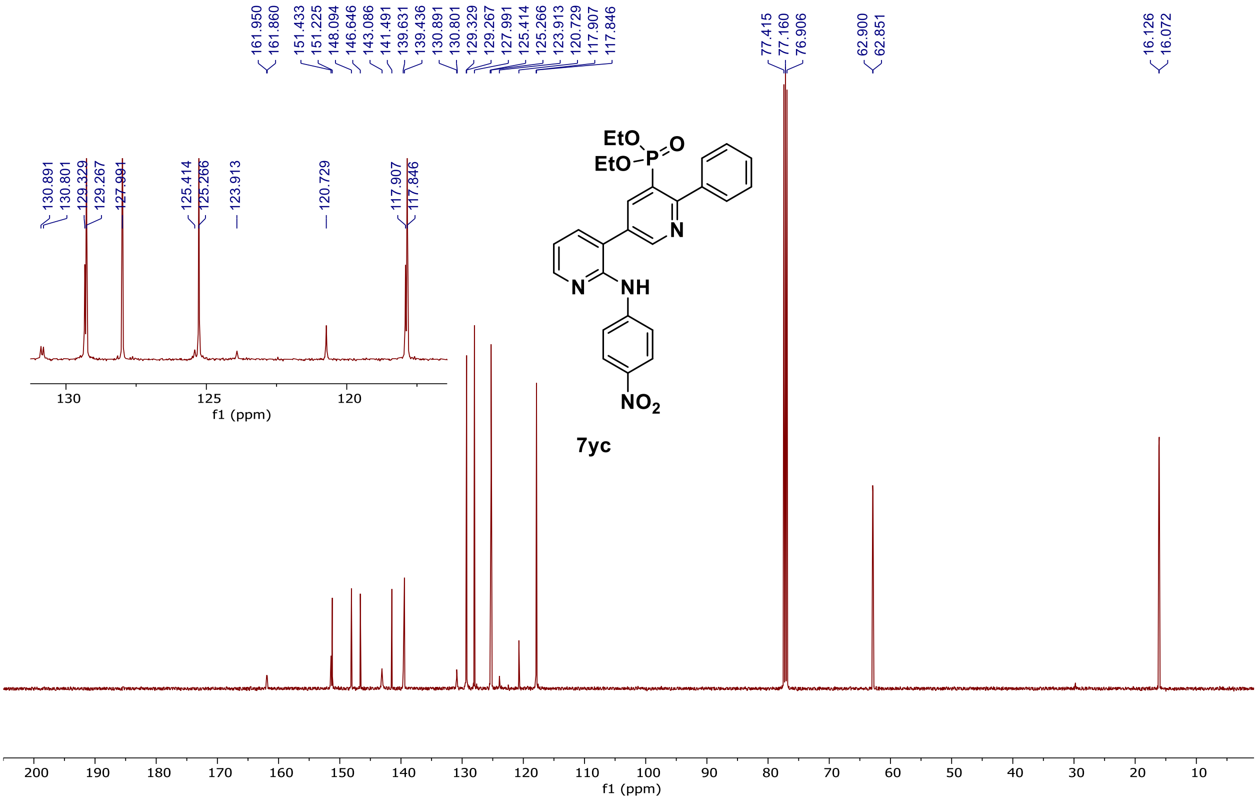

^{1}H and ^{13}C NMR Spectrum of **7yc** in $CDCl_3$

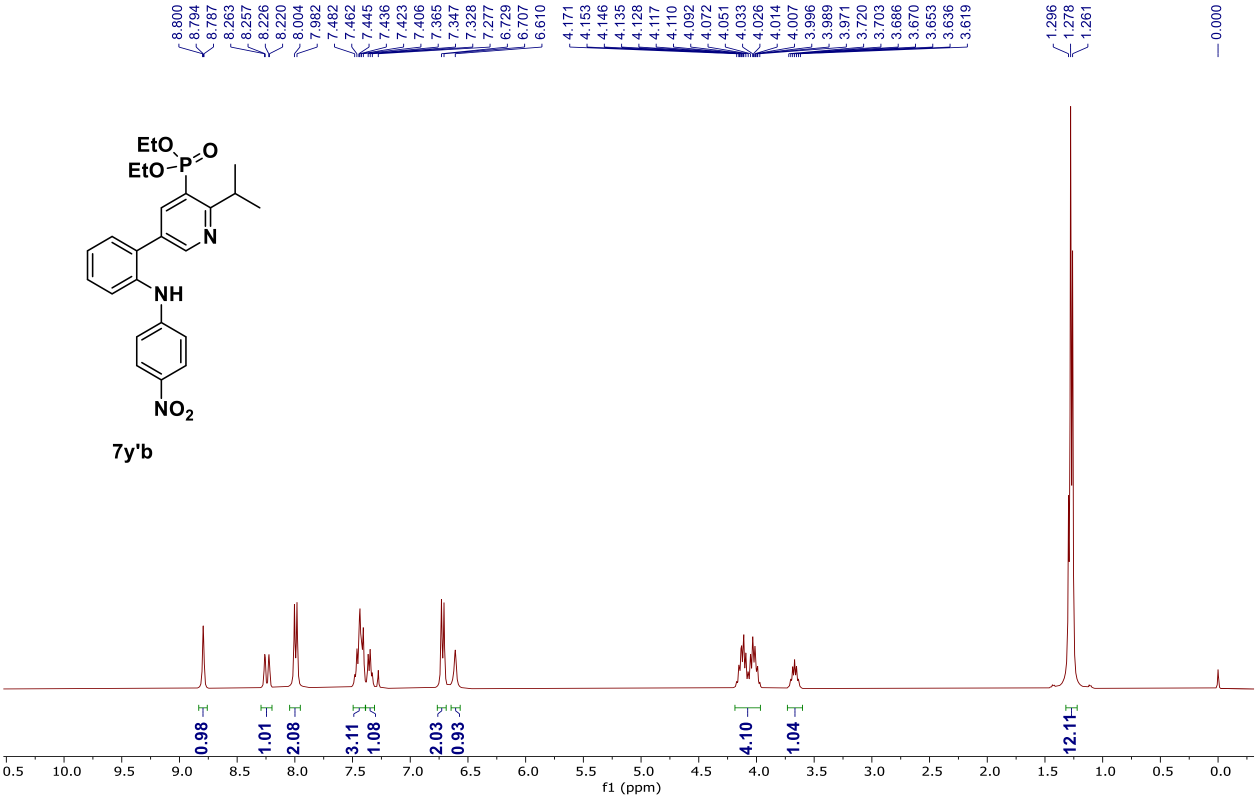

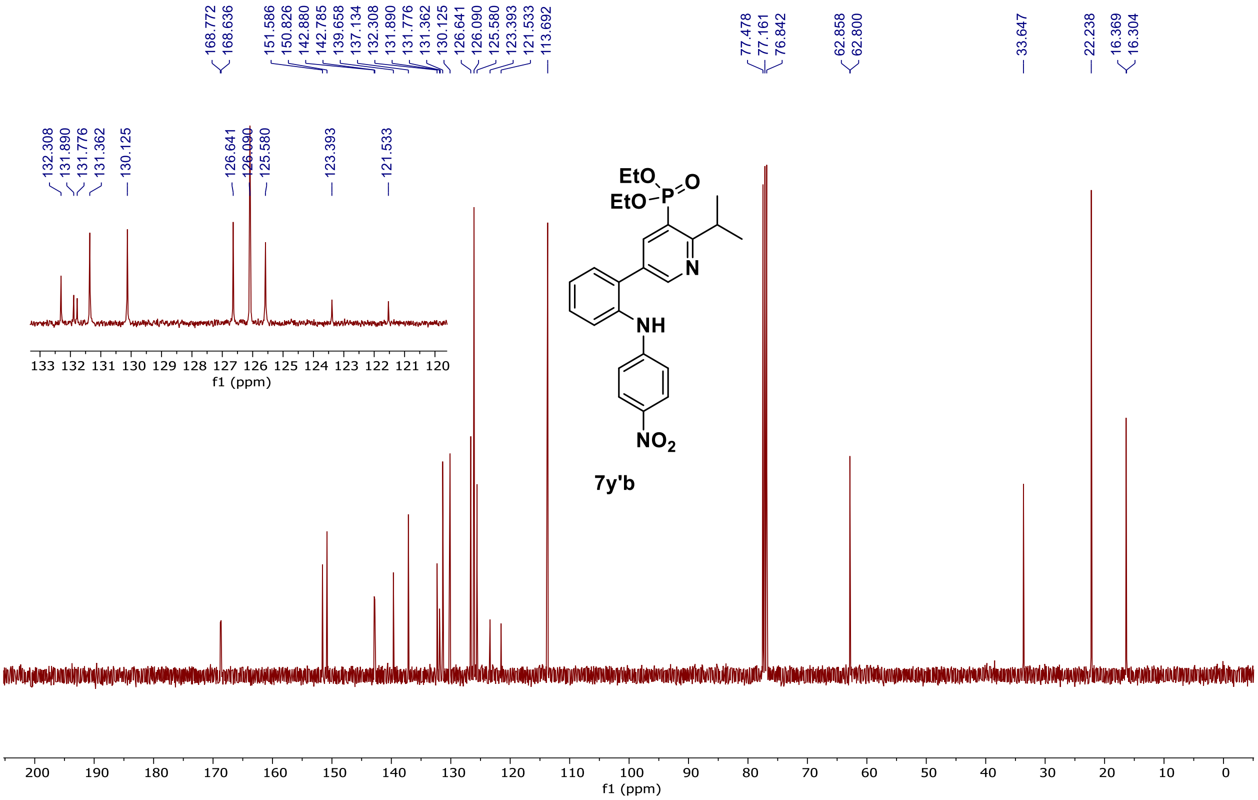

^{1}H and ^{13}C NMR Spectrum of **7y'b** in $CDCl_3$

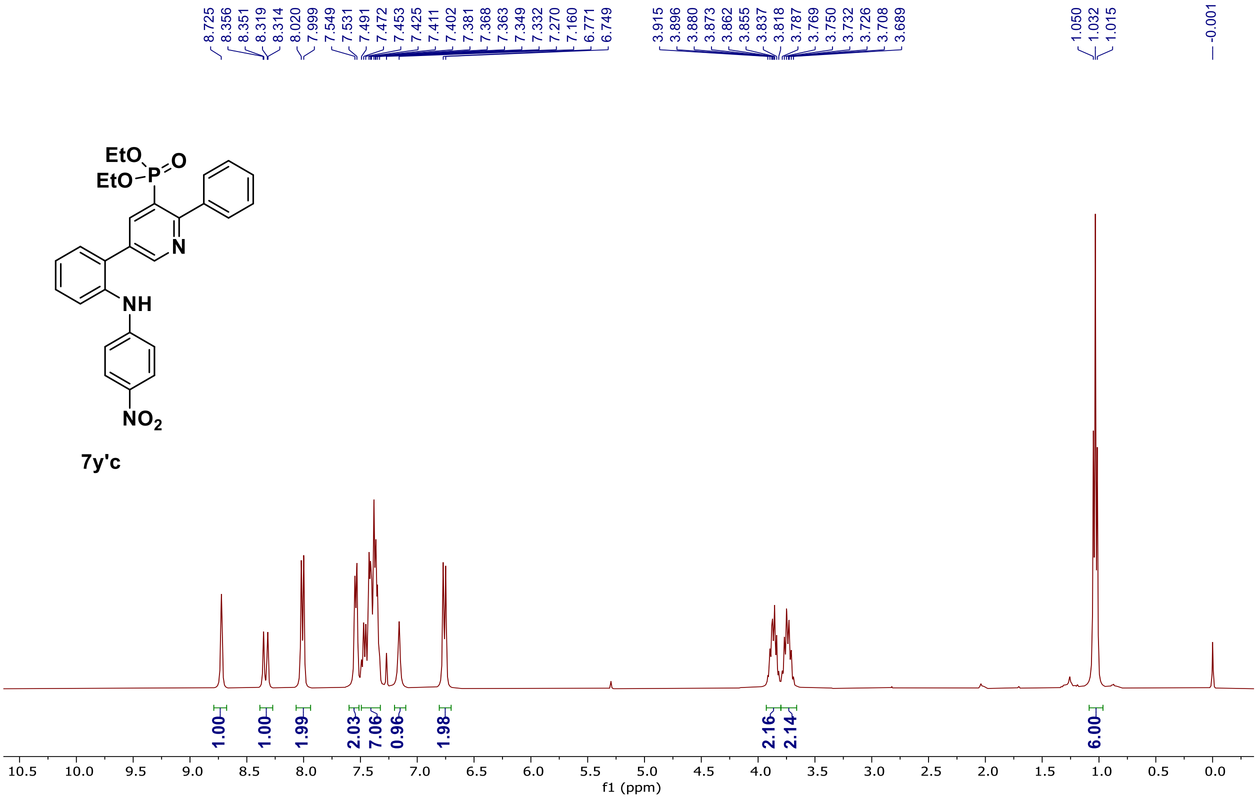

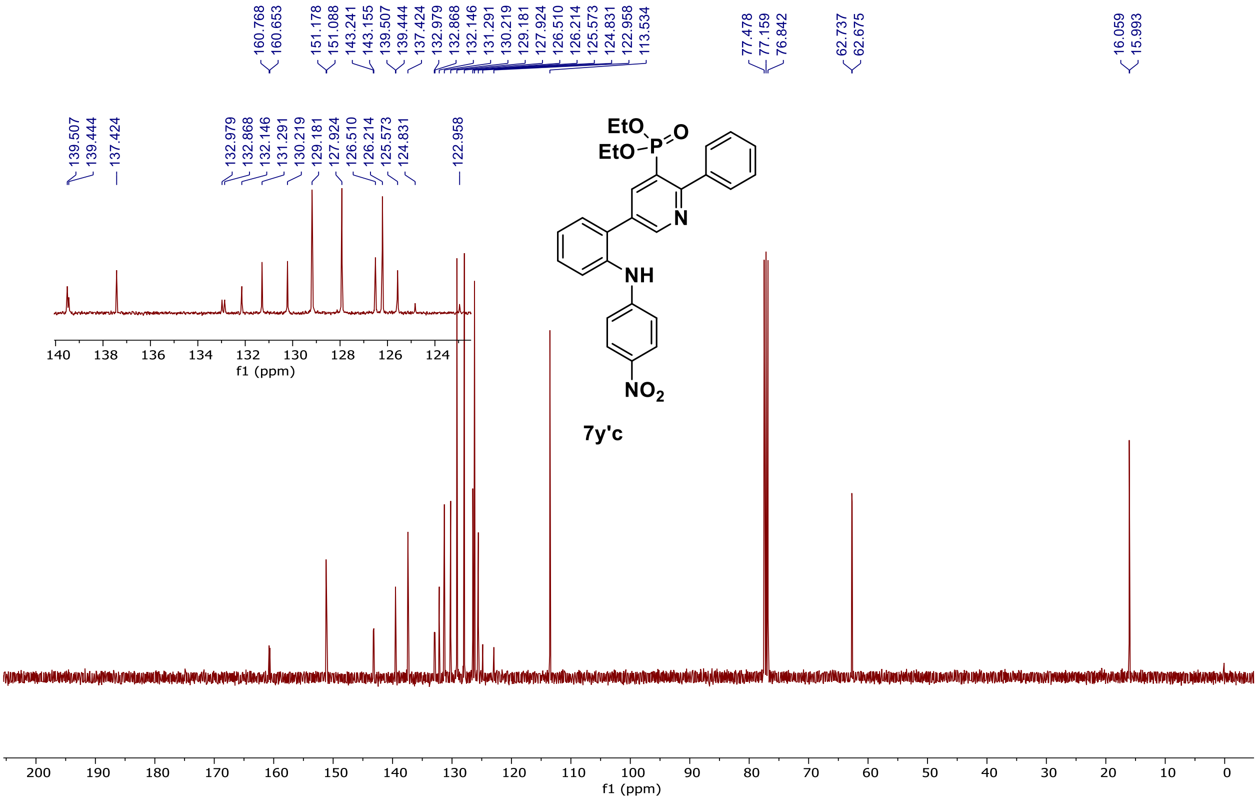

^{1}H and ^{13}C NMR Spectrum of **7y'c** in $CDCl_3$

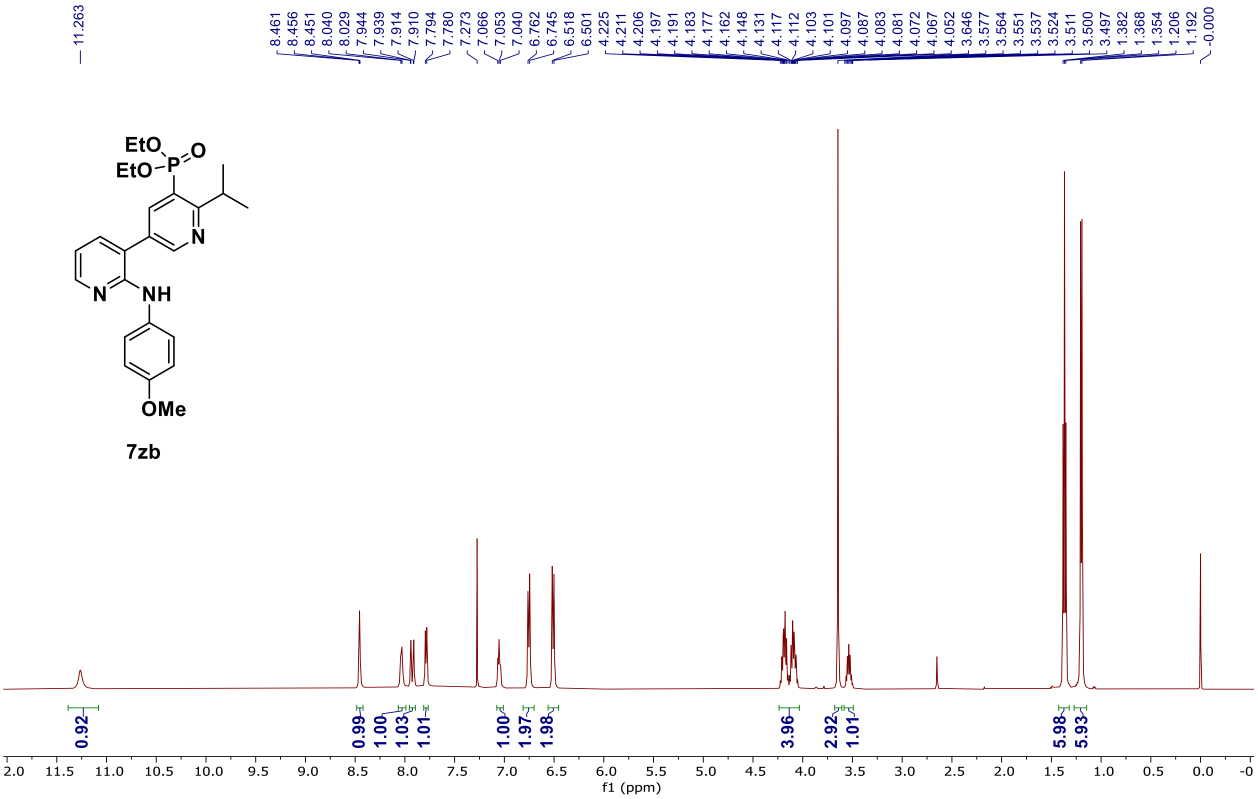

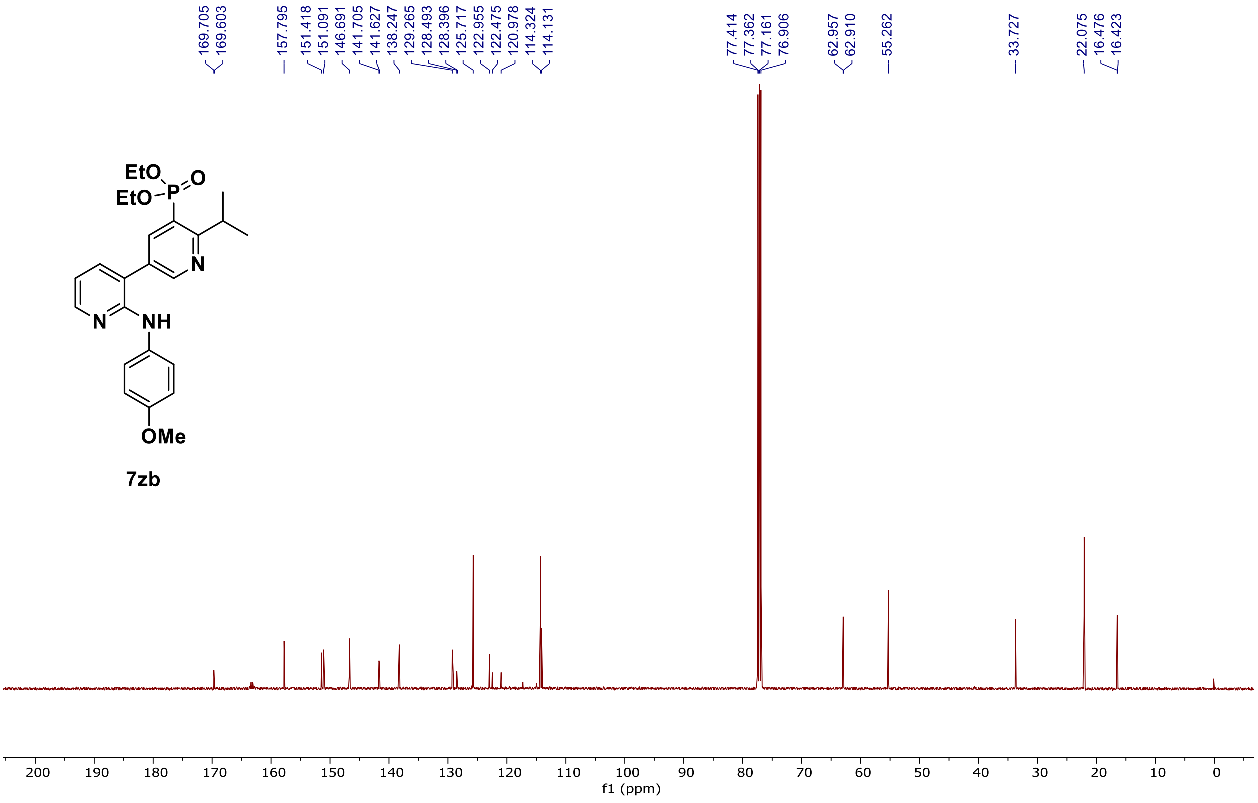

^{1}H and ^{13}C NMR Spectrum of **7zb** in $CDCl_3$

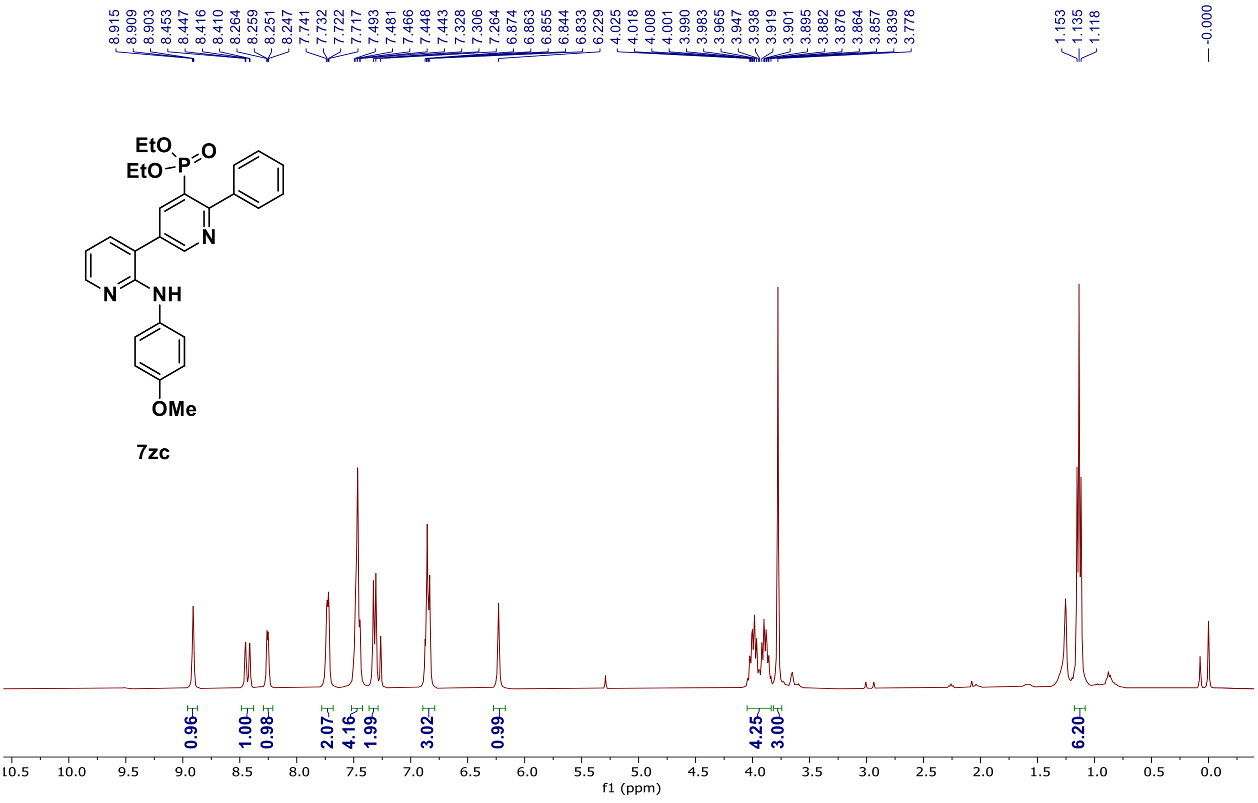

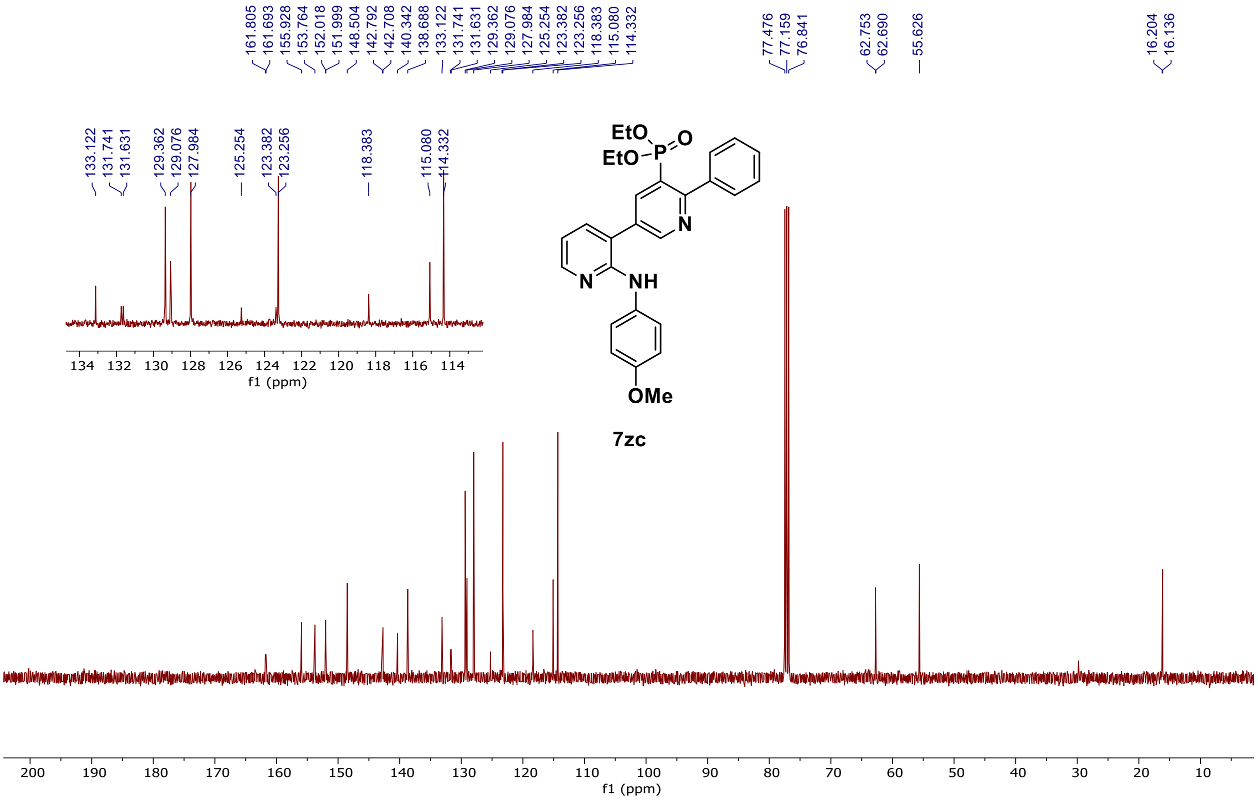

^{1}H and ^{13}C NMR Spectrum of **7zc** in $CDCl_3$

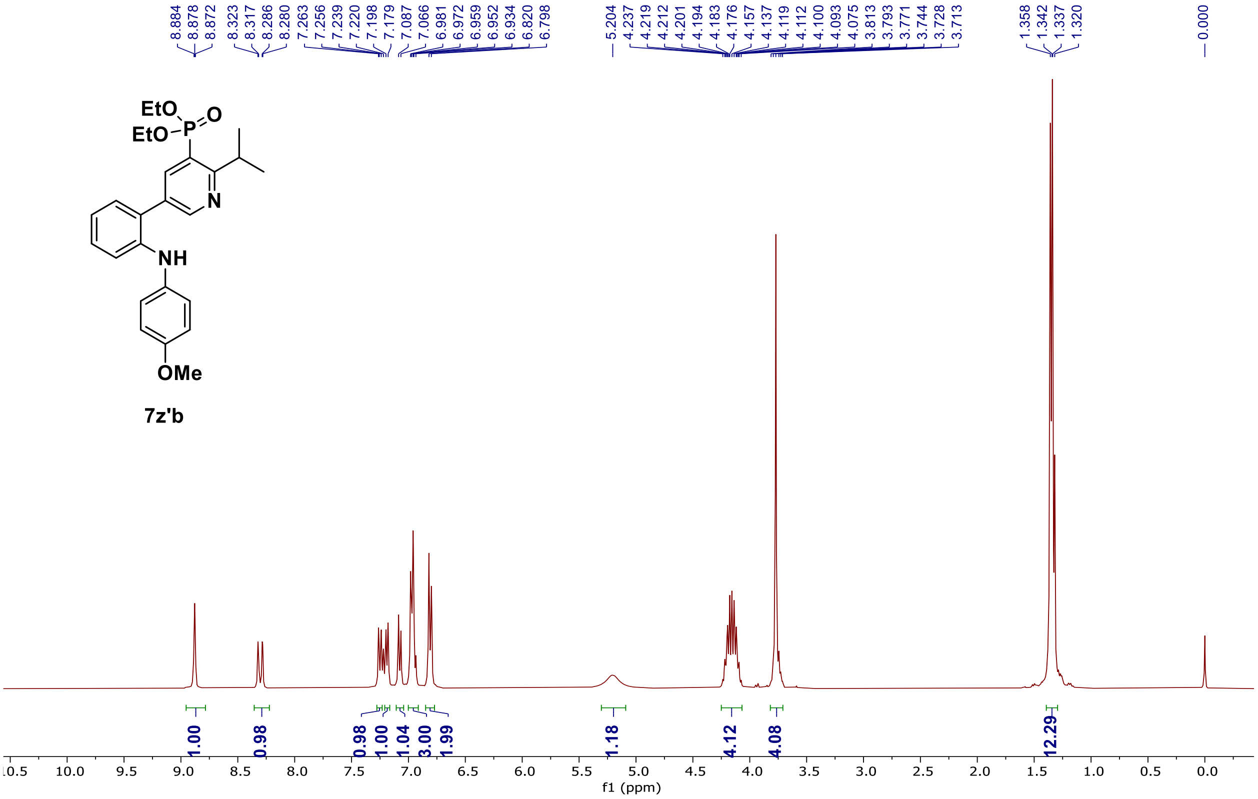

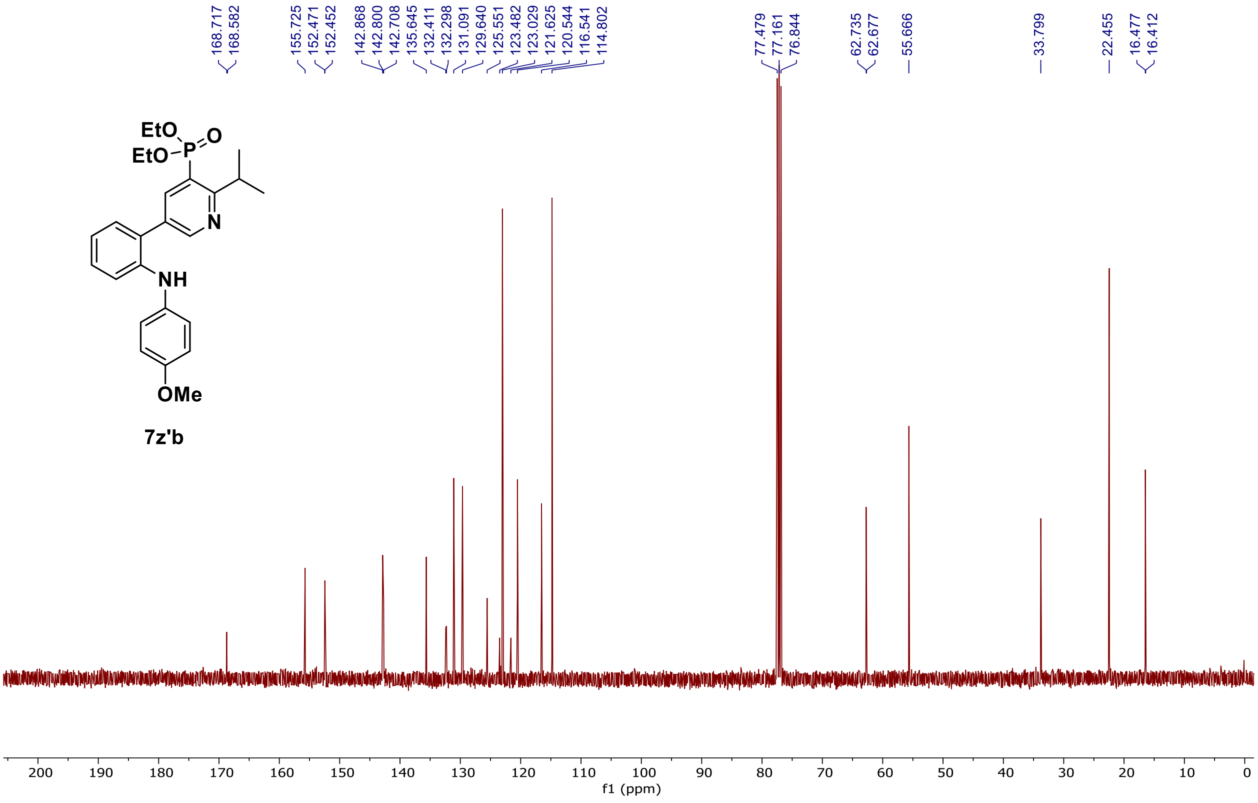

^{1}H and ^{13}C NMR Spectrum of **7z'b** in $CDCl_3$

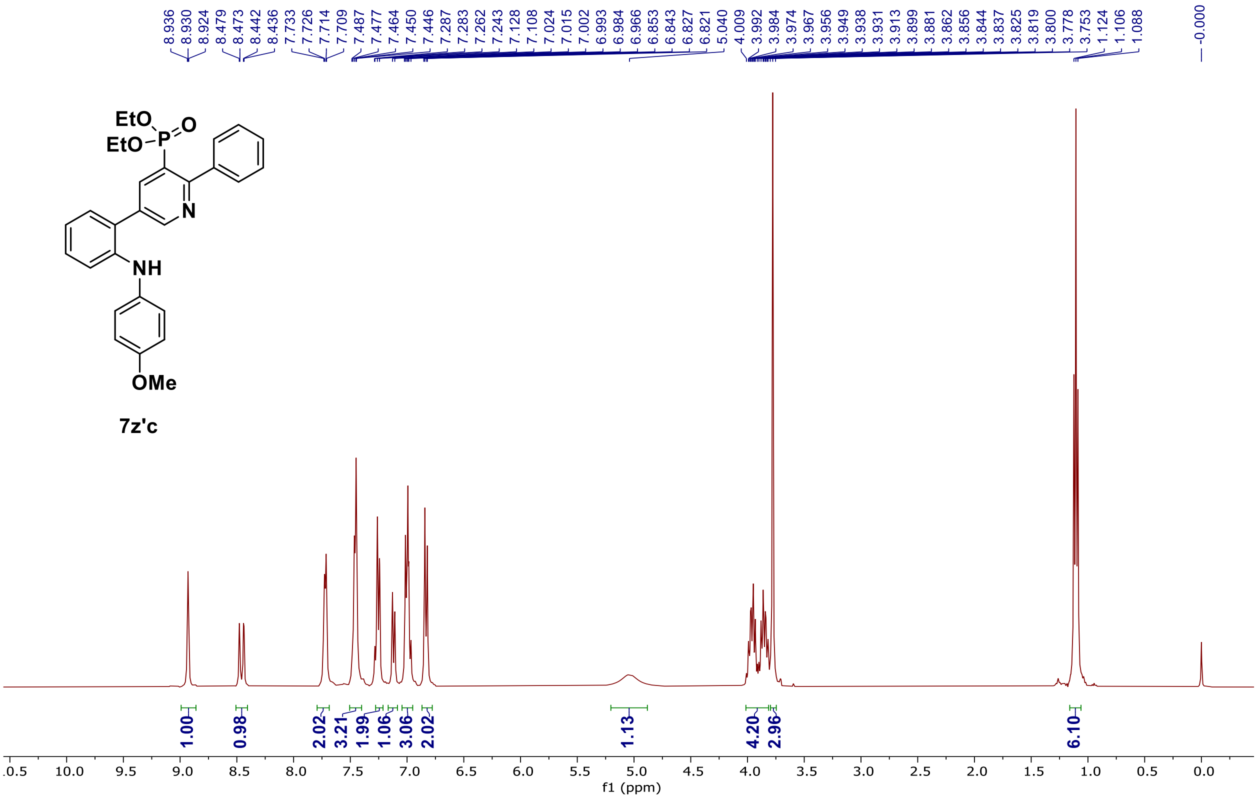

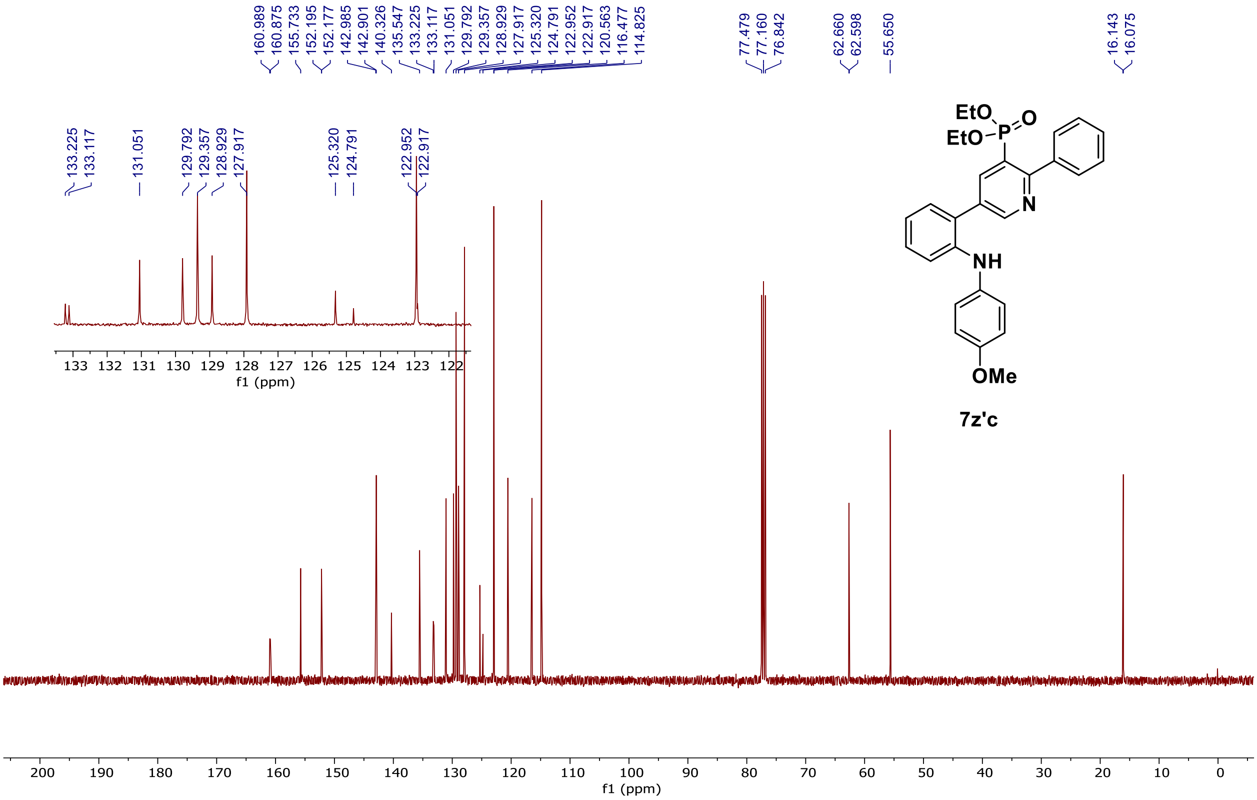

^{1}H and ^{13}C NMR Spectrum of **7z'c** in $CDCl_3$

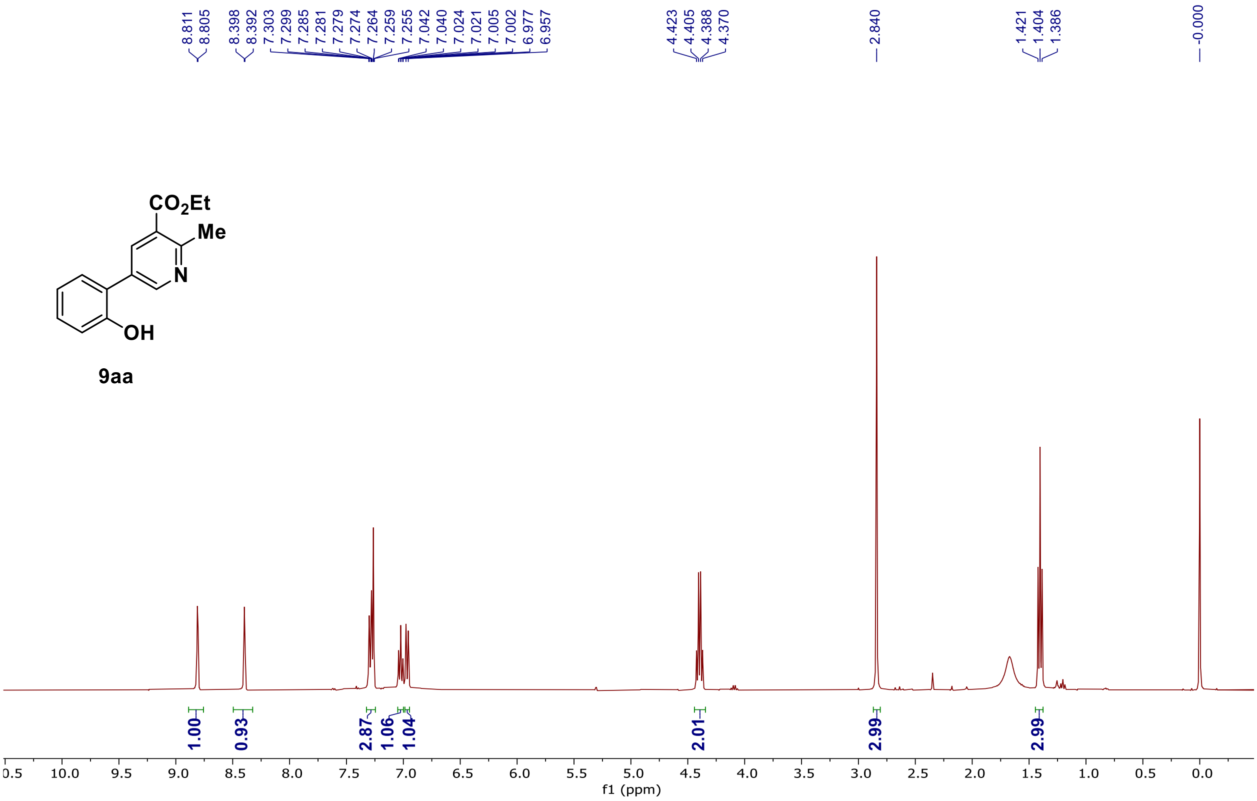

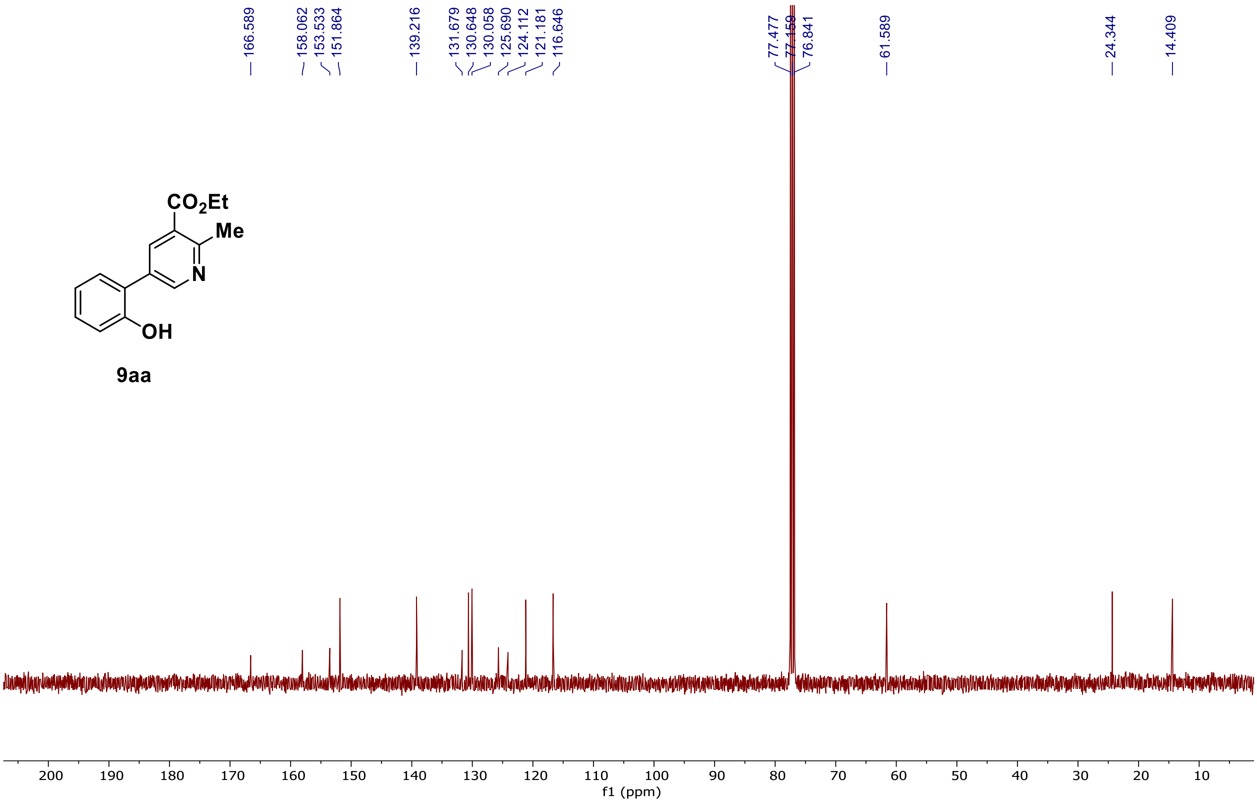

^{1}H and ^{13}C NMR Spectrum of **9aa** in $CDCl_3$

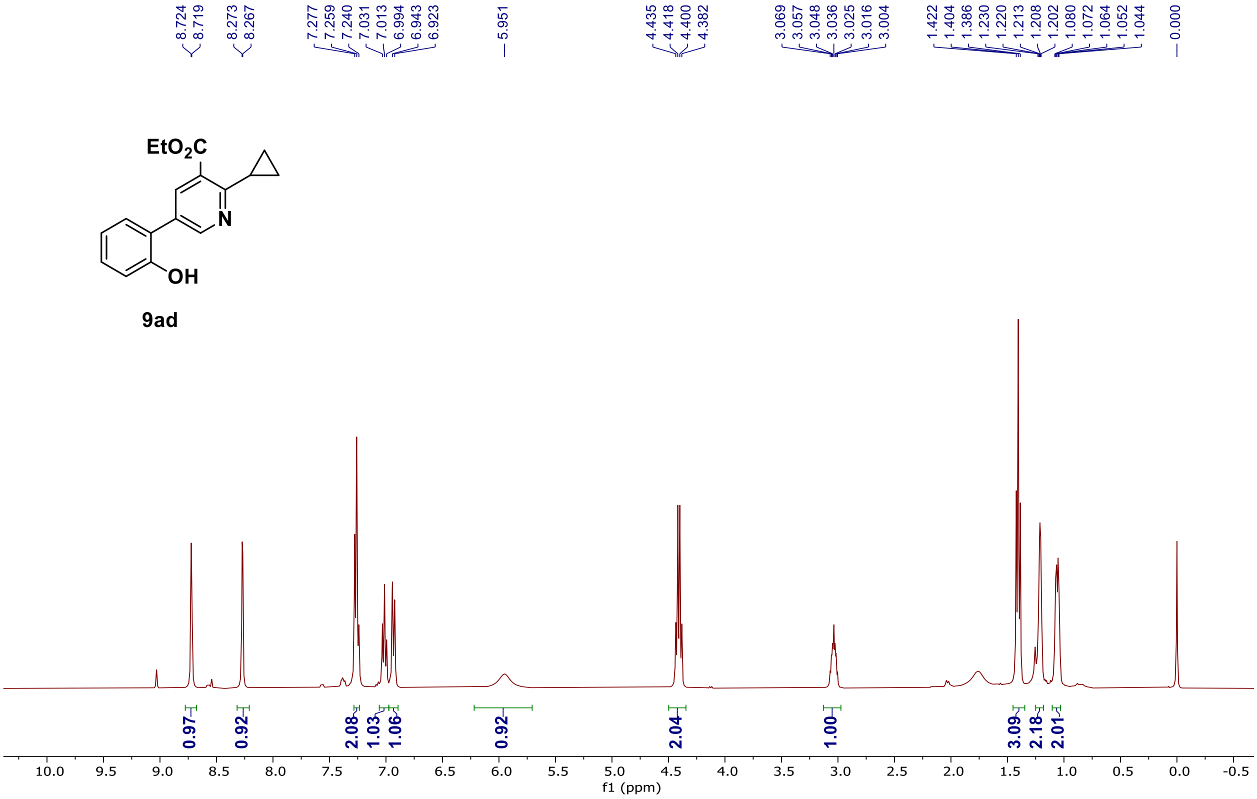

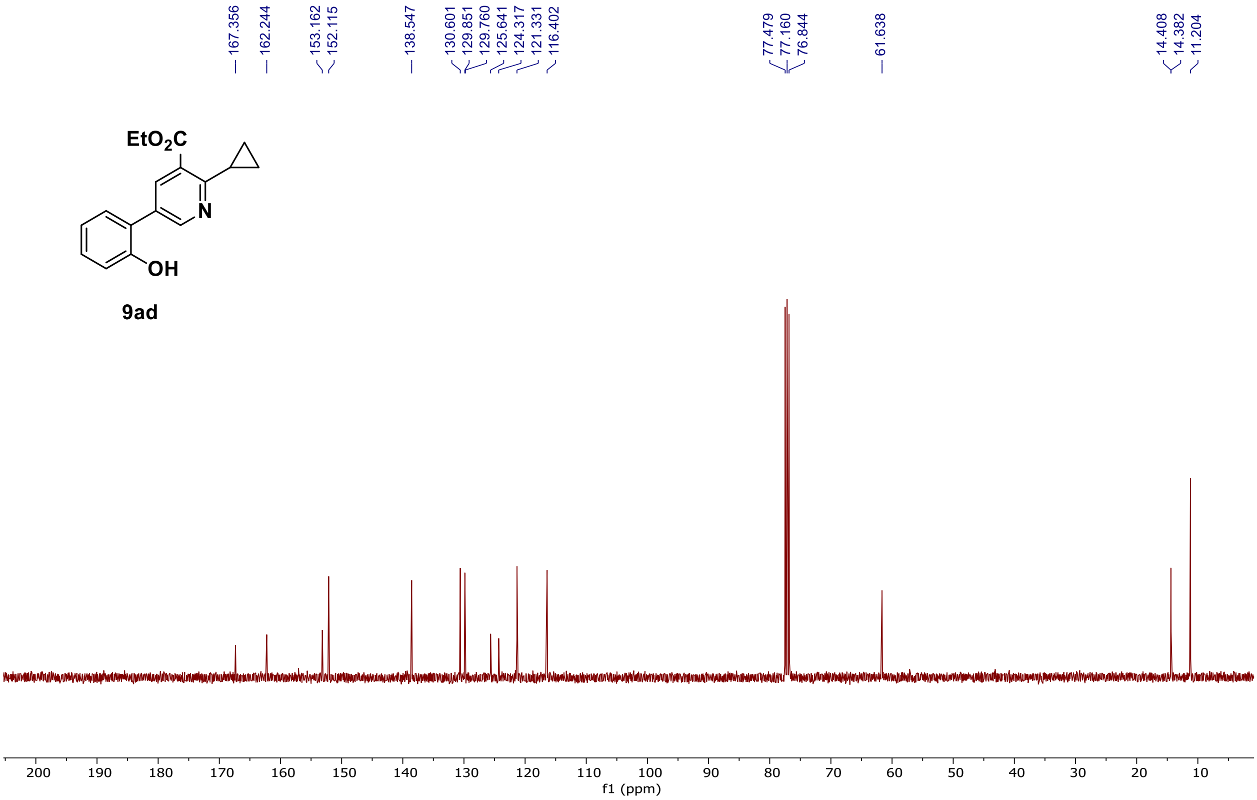

^{1}H and ^{13}C NMR Spectrum of **9ad** in $CDCl_3$

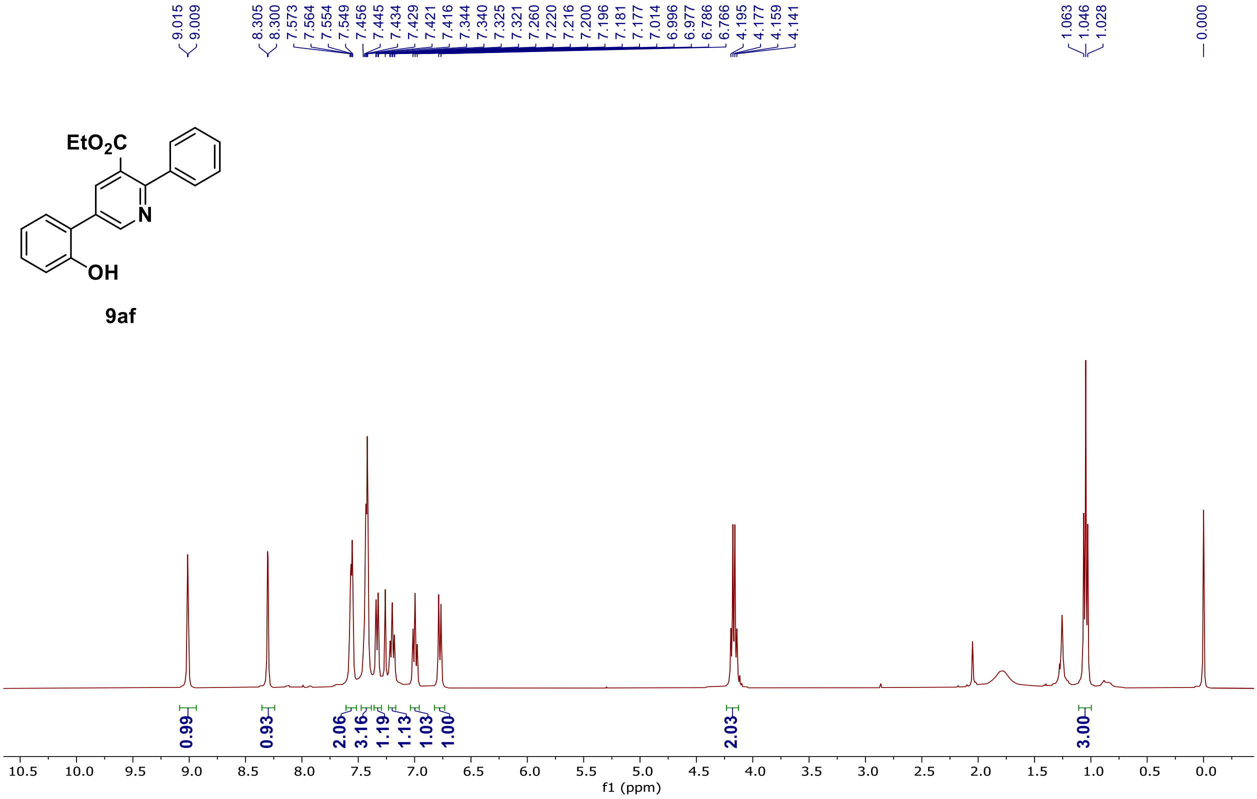

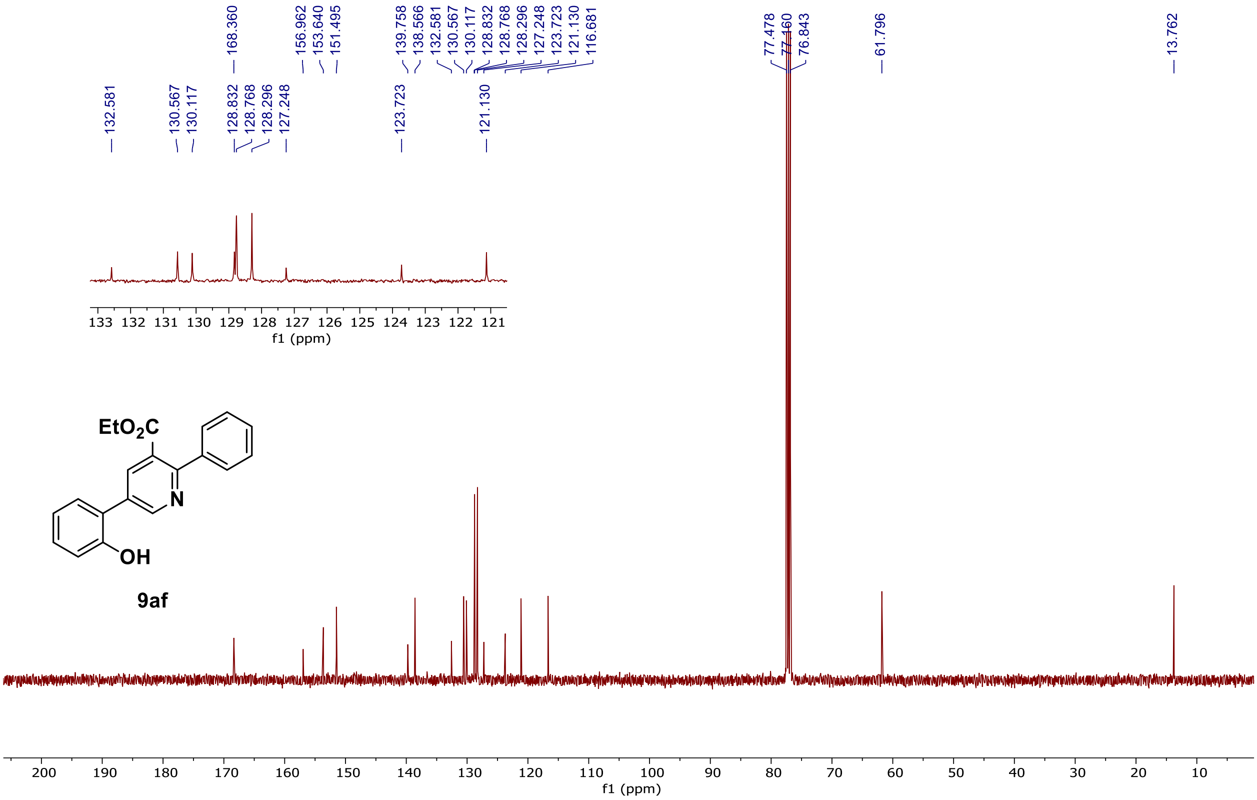

^{1}H and ^{13}C NMR Spectrum of **9af** in $CDCl_3$

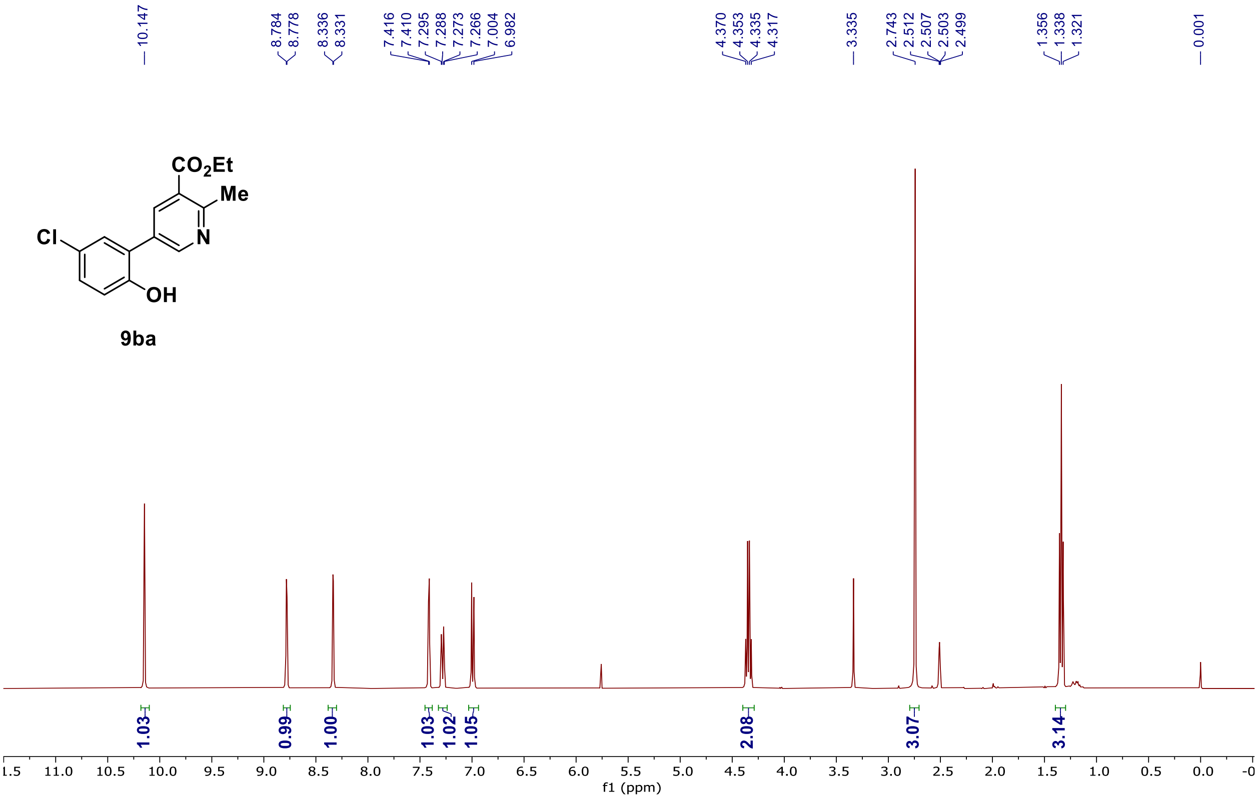

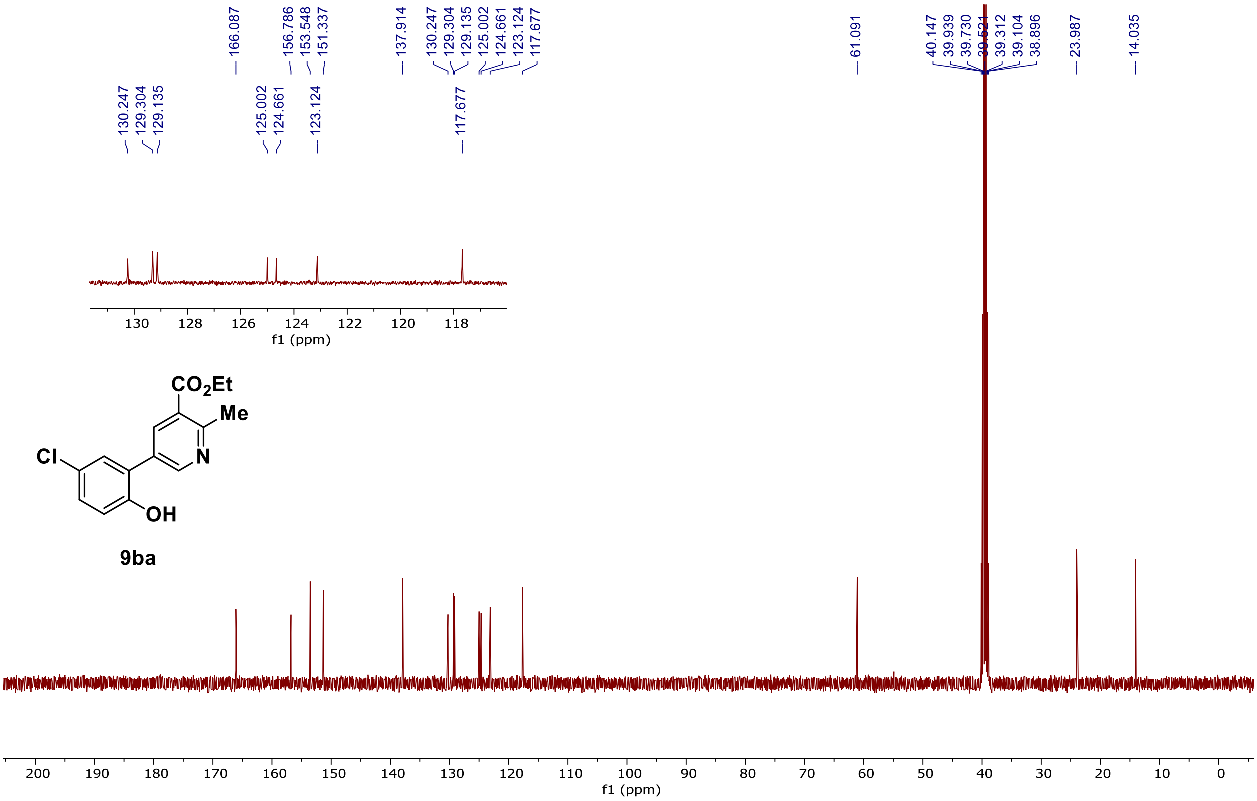

^{1}H and ^{13}C NMR Spectrum of **9ba** in DMSO-*d*$_6$

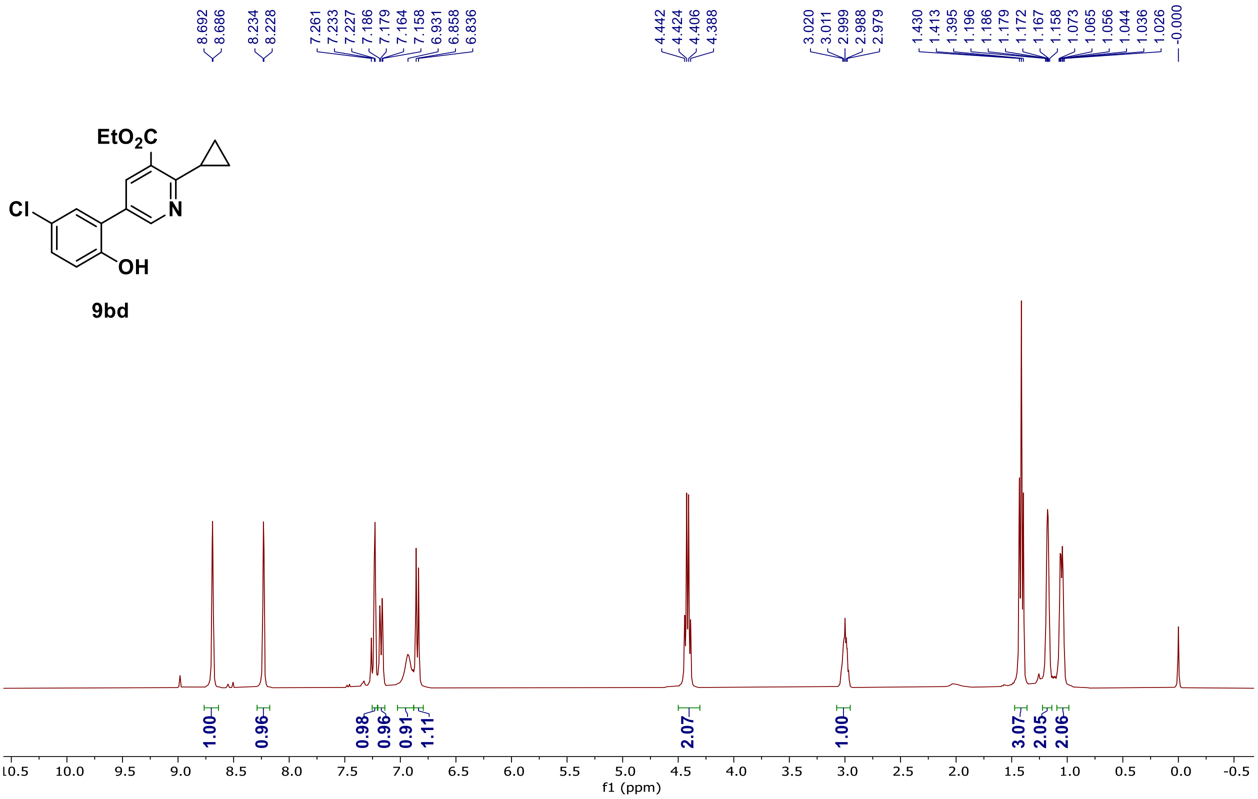

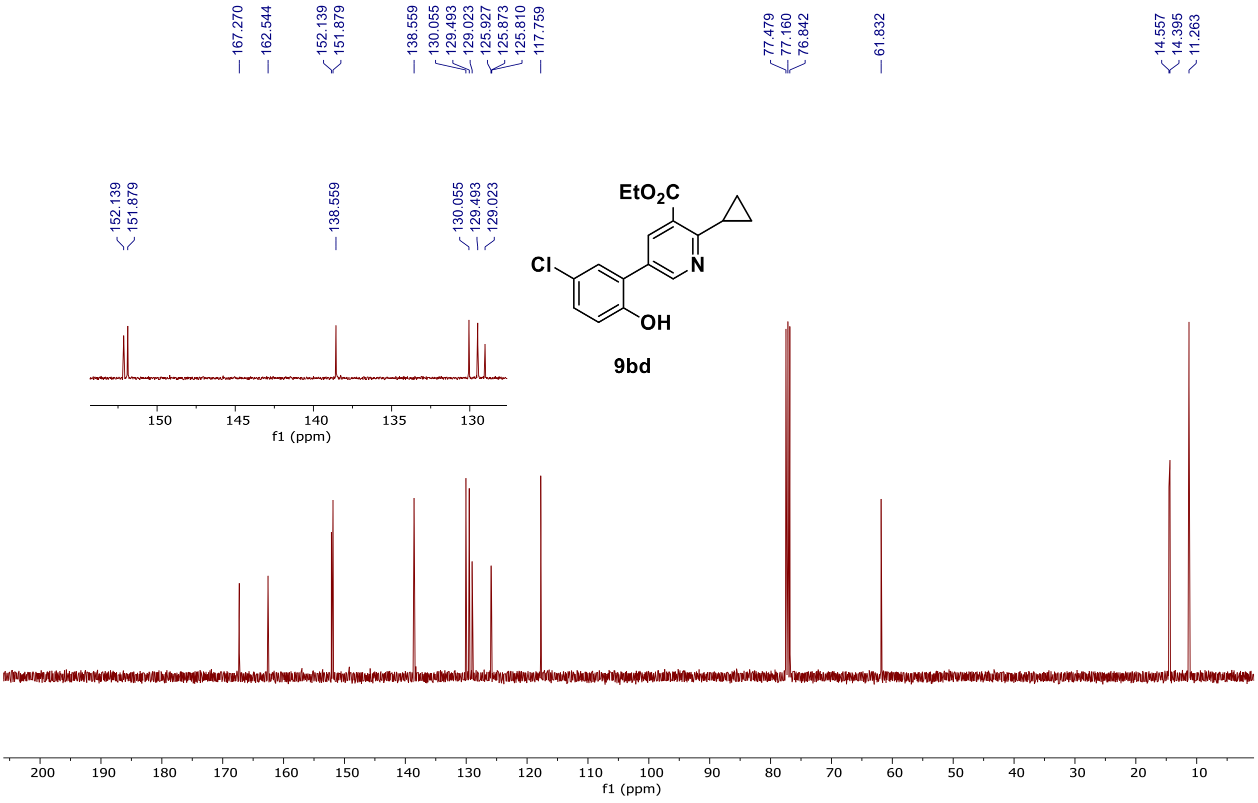

^{1}H and ^{13}C NMR Spectrum of **9bd** in $CDCl_3$

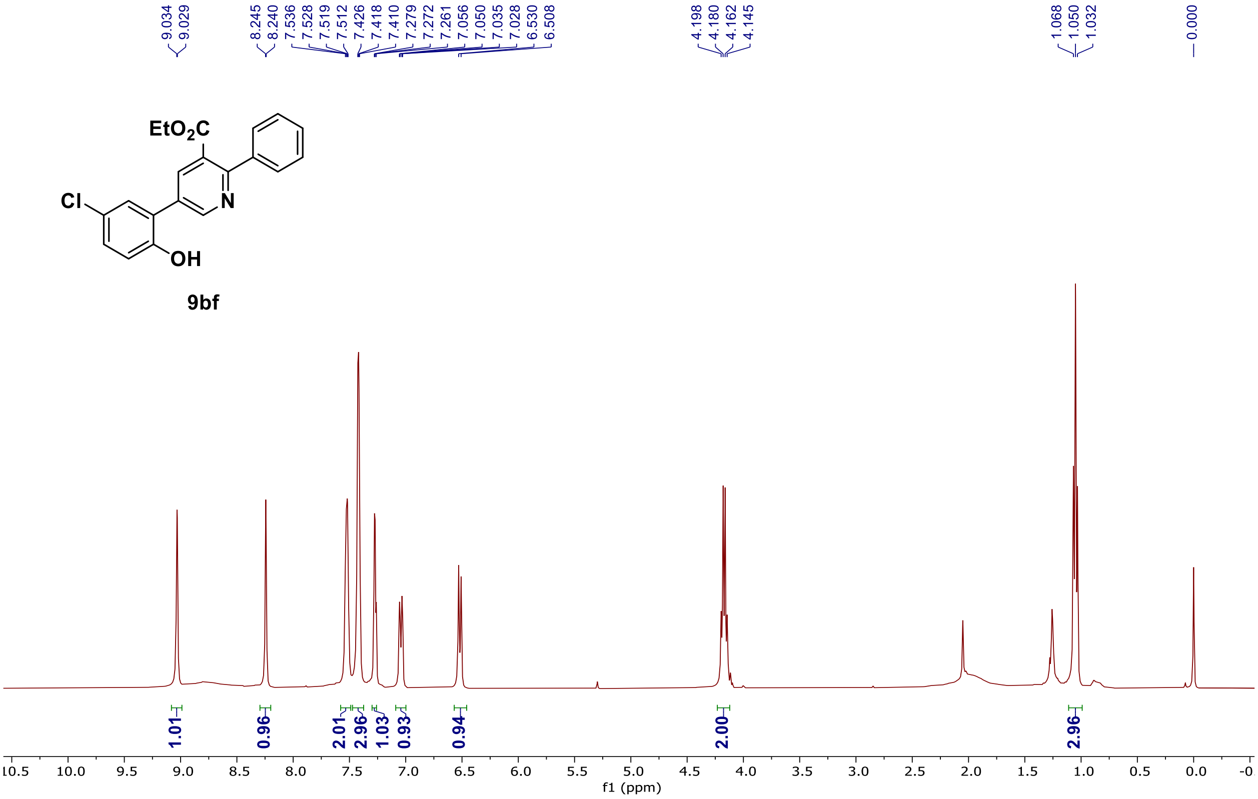

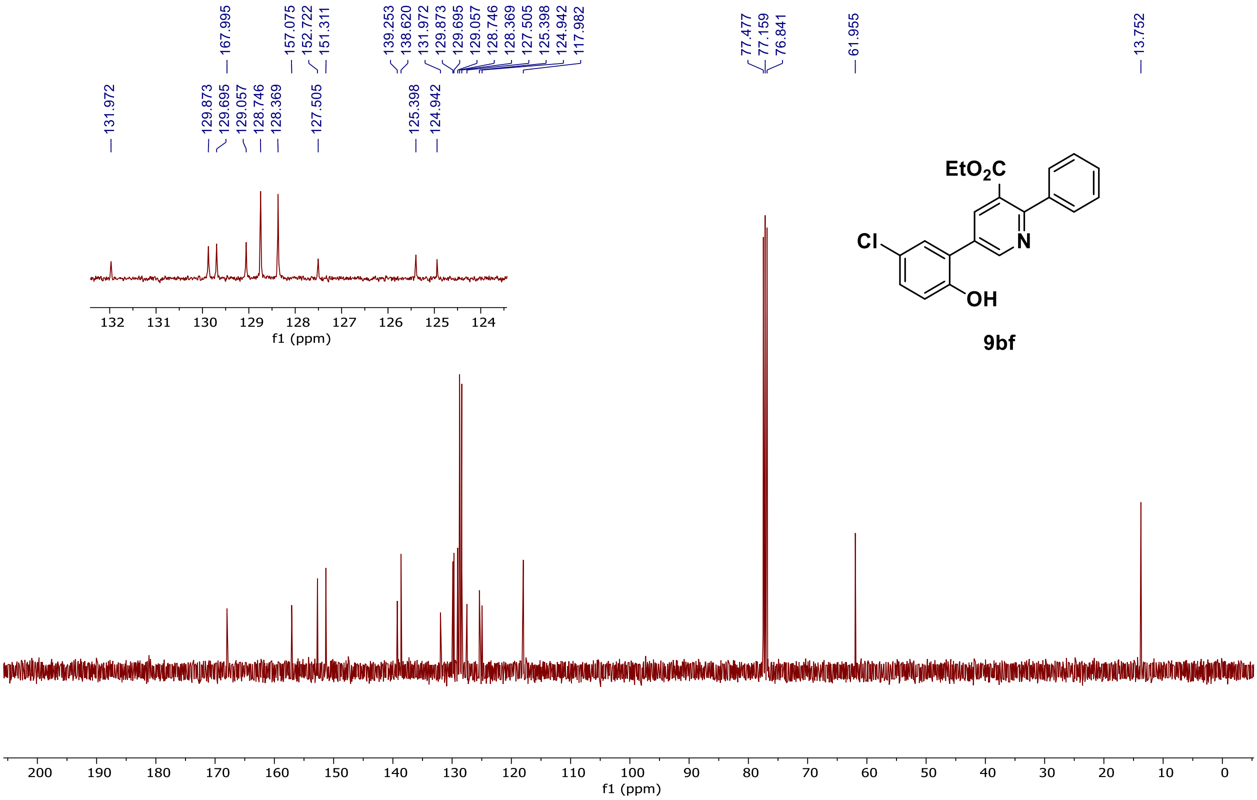

^{1}H and ^{13}C NMR Spectrum of **9bf** in $CDCl_3$

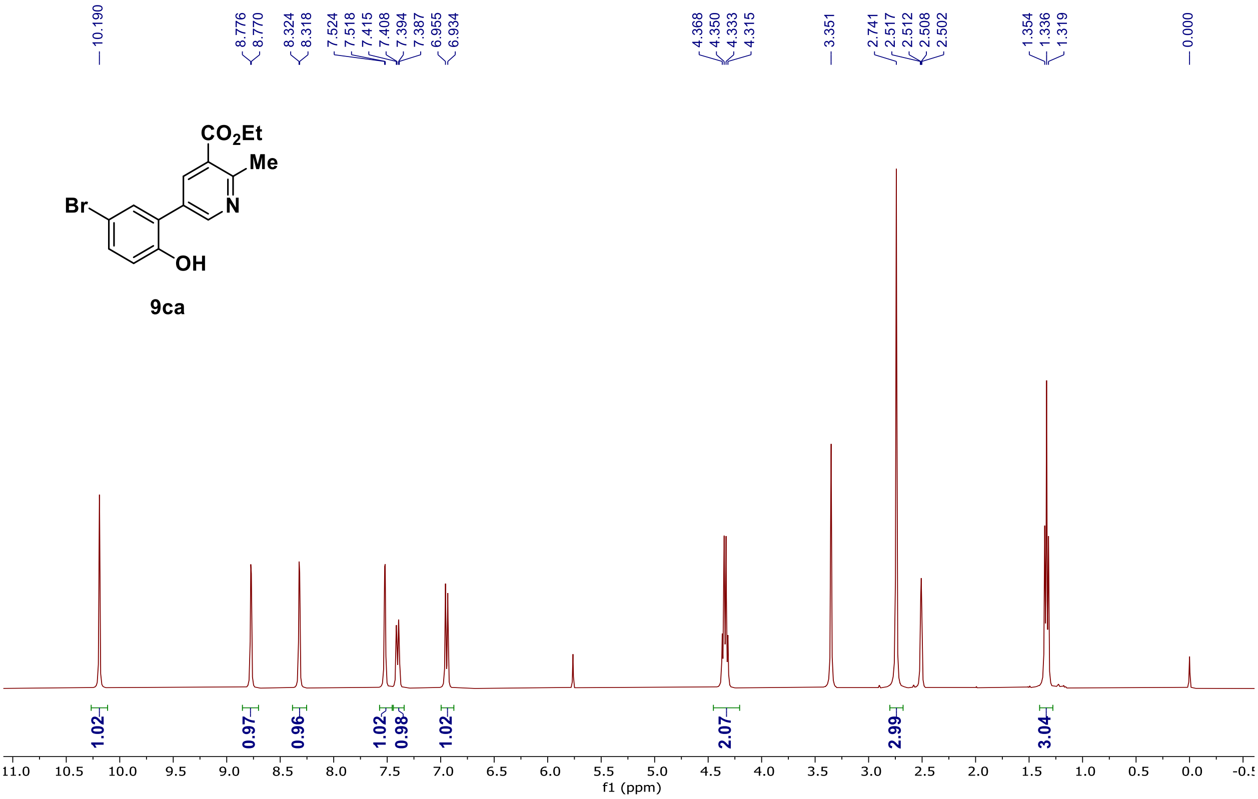

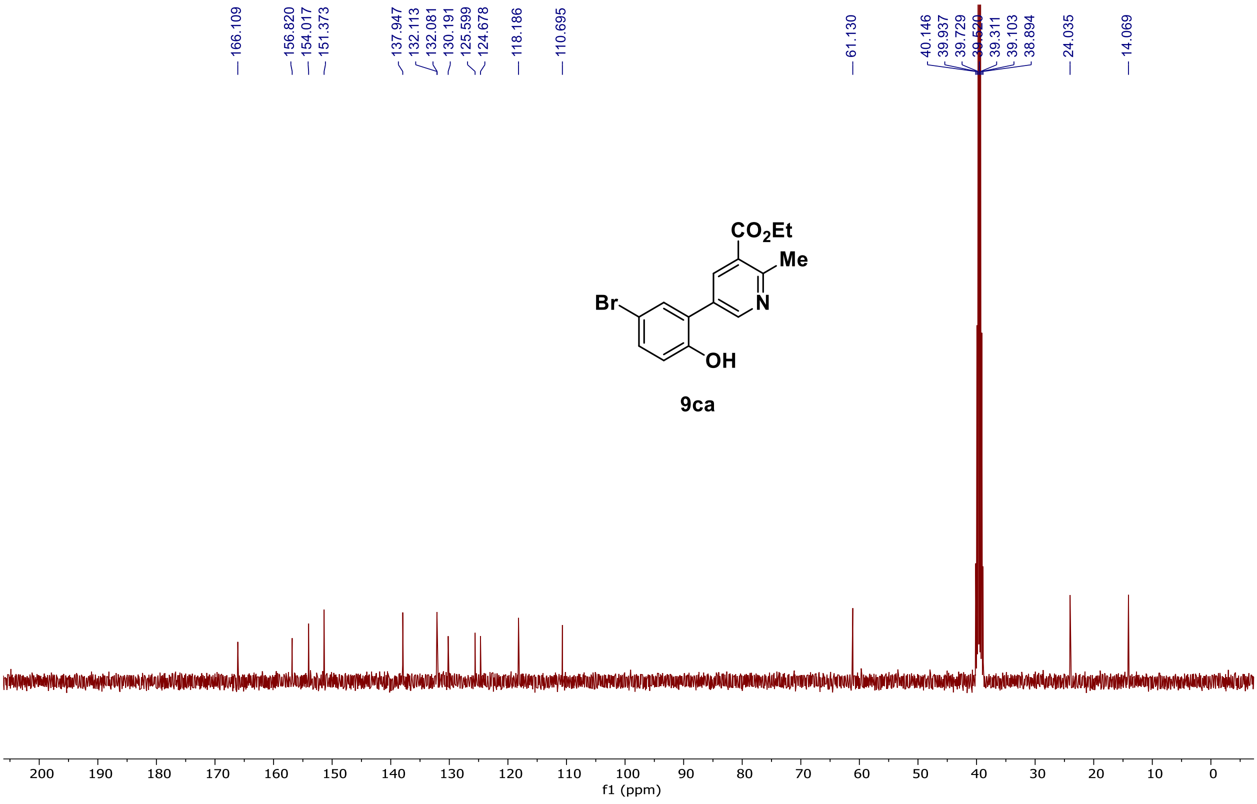

^{1}H and ^{13}C NMR Spectrum of **9ca** in DMSO-d_6

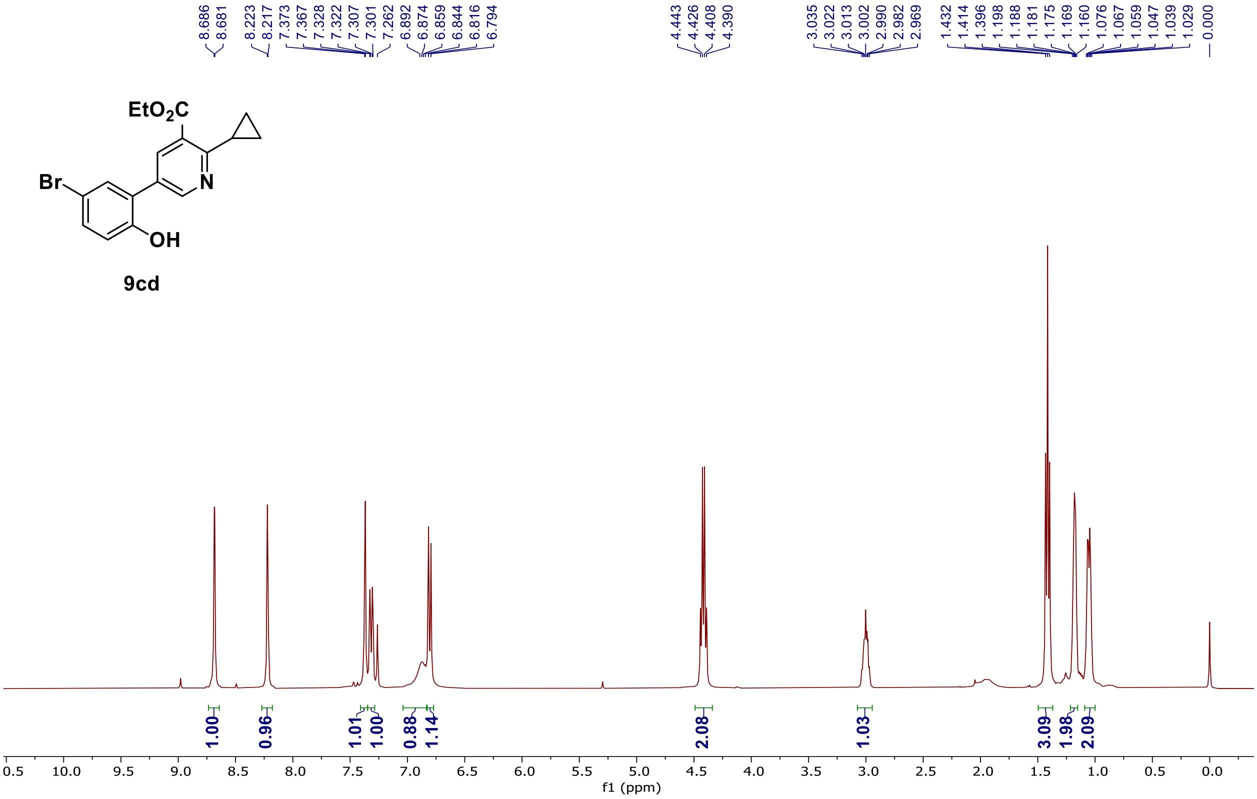

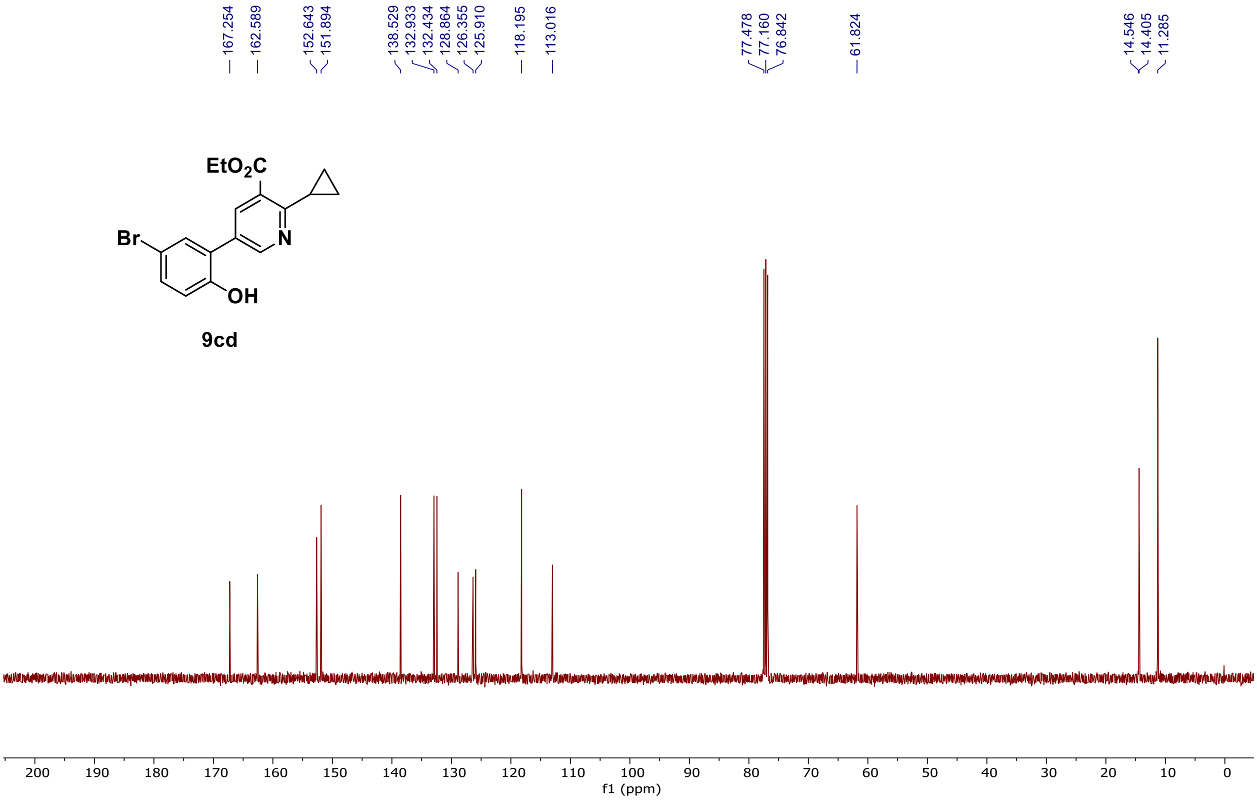

^{1}H and ^{13}C NMR Spectrum of **9cd** in $CDCl_3$

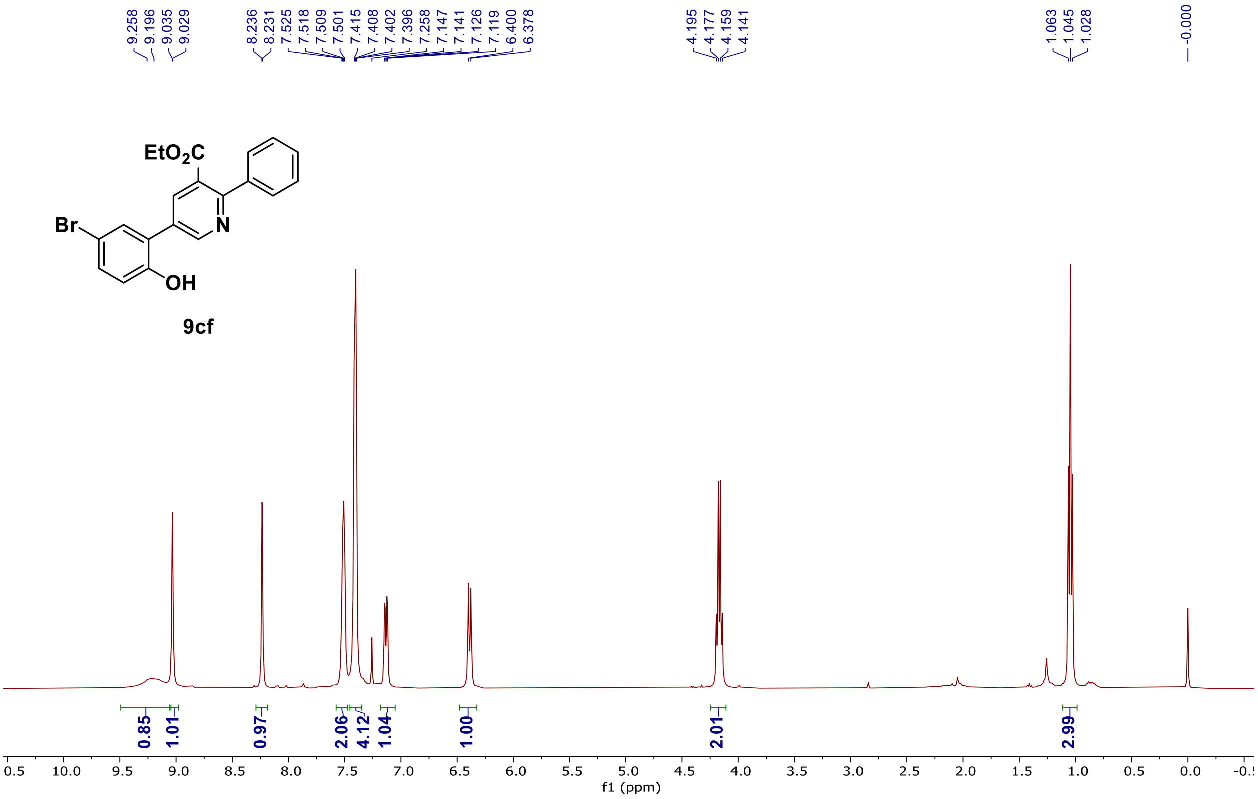

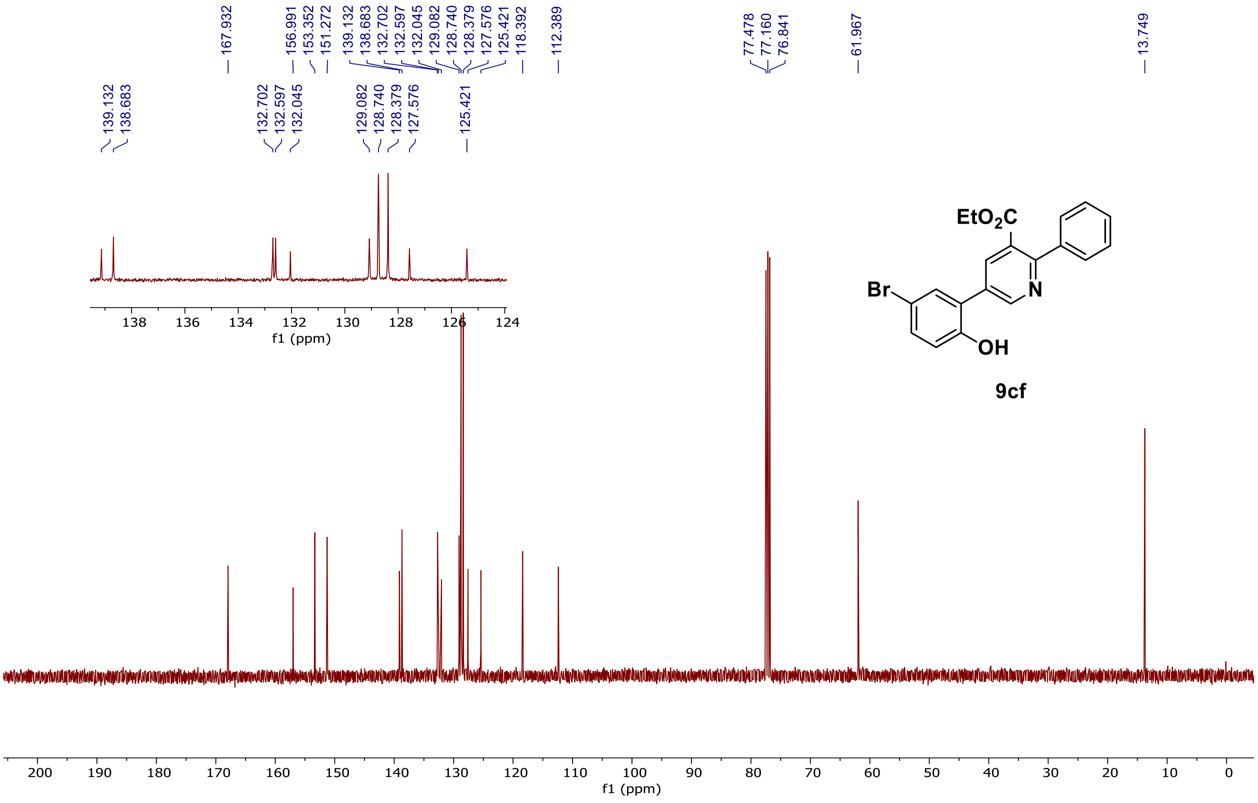

^{1}H and ^{13}C NMR Spectrum of **9cf** in $CDCl_3$

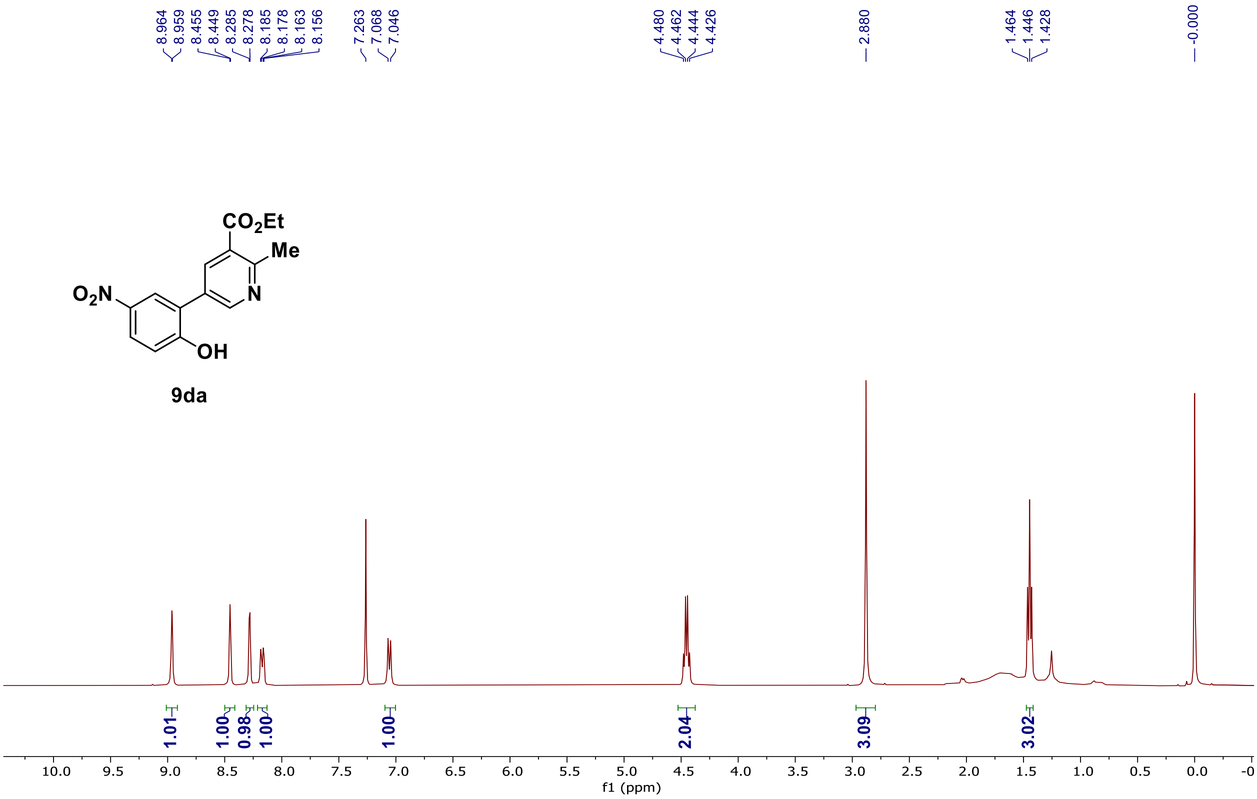

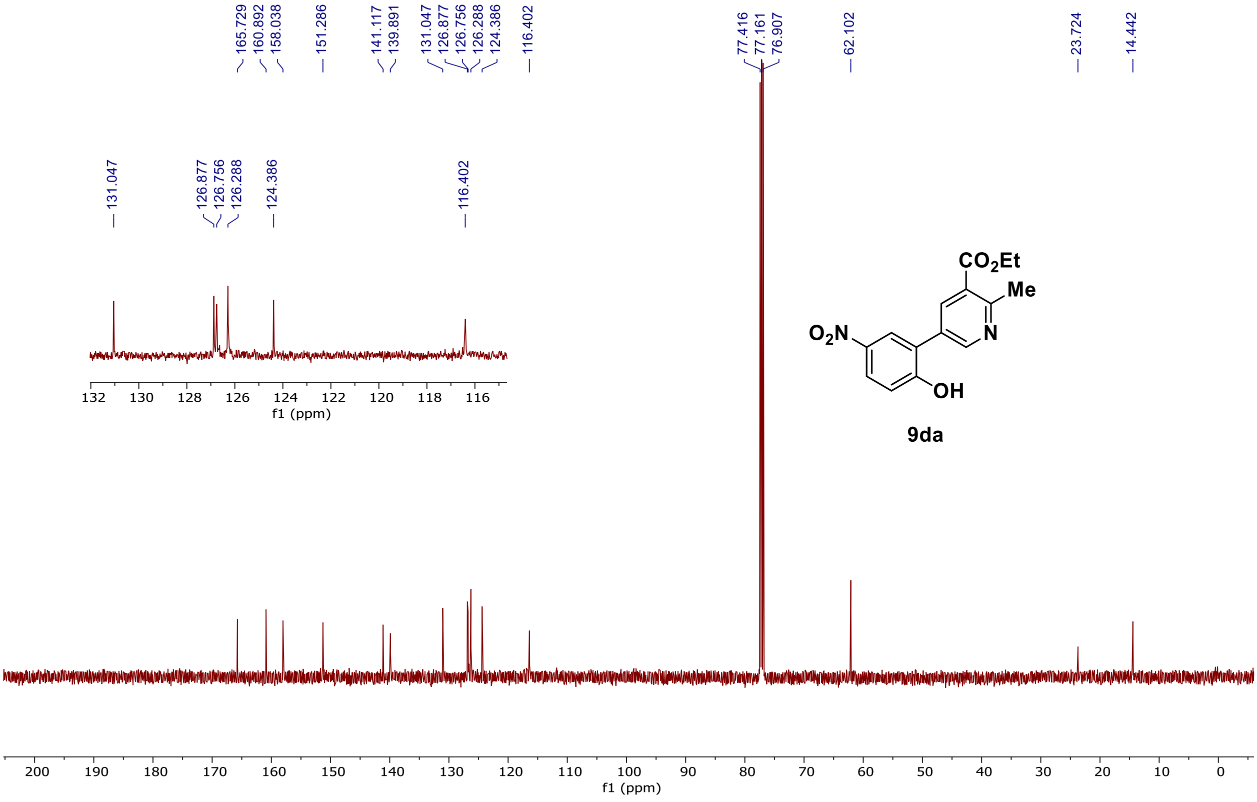

^{1}H and ^{13}C NMR Spectrum of **9da** in $CDCl_3$

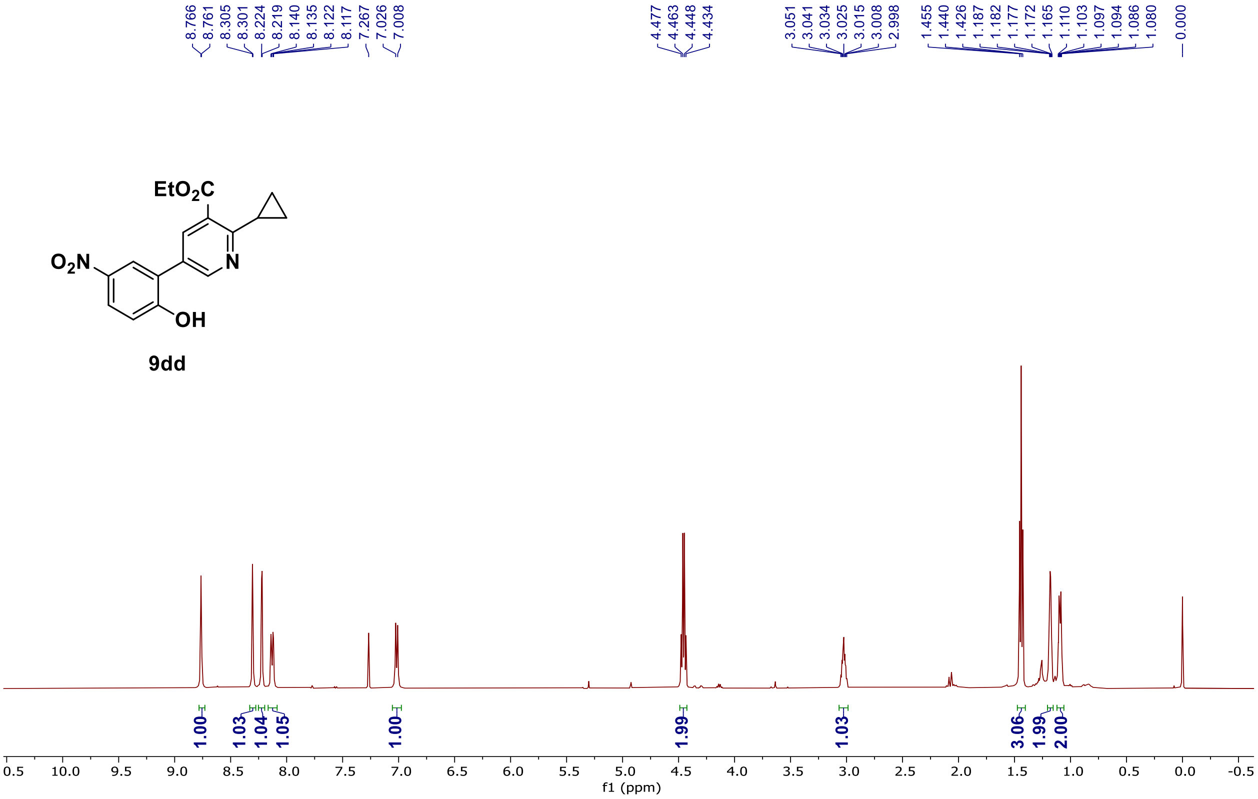

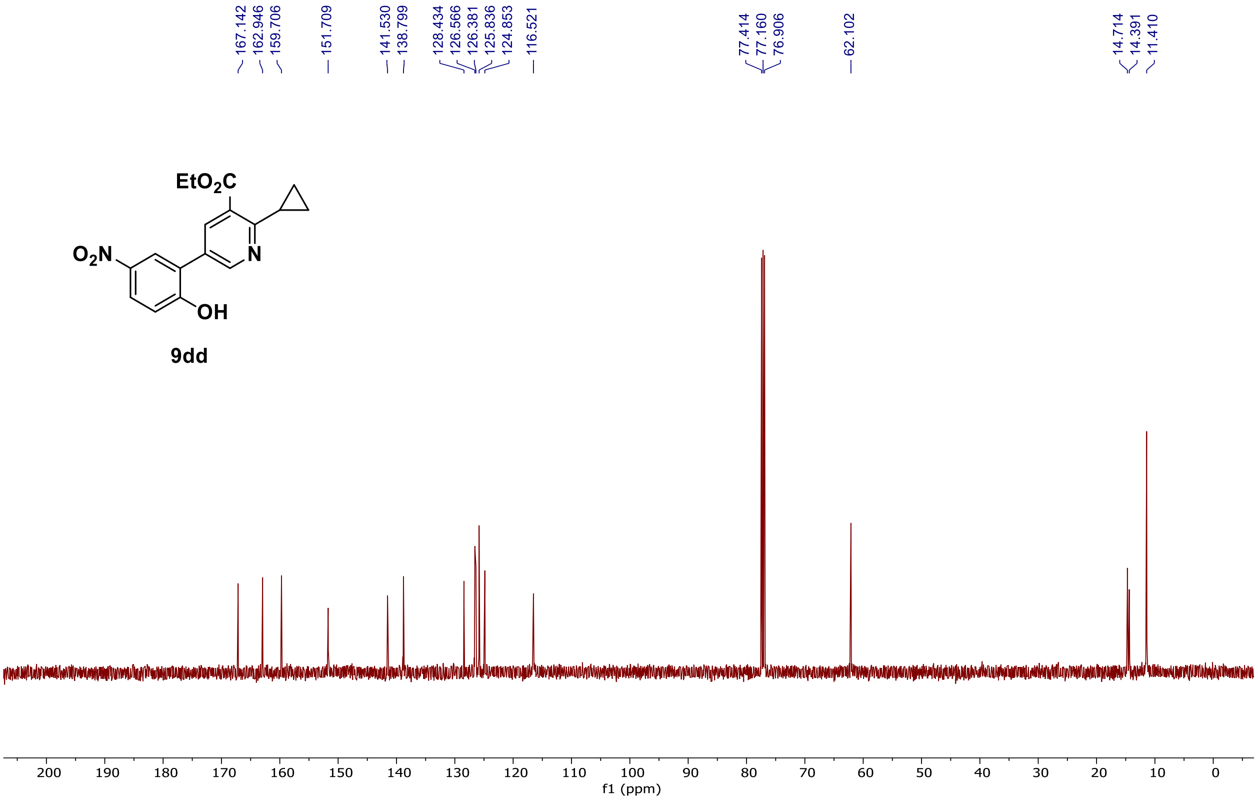

^{1}H and ^{13}C NMR Spectrum of **9dd** in $CDCl_3$

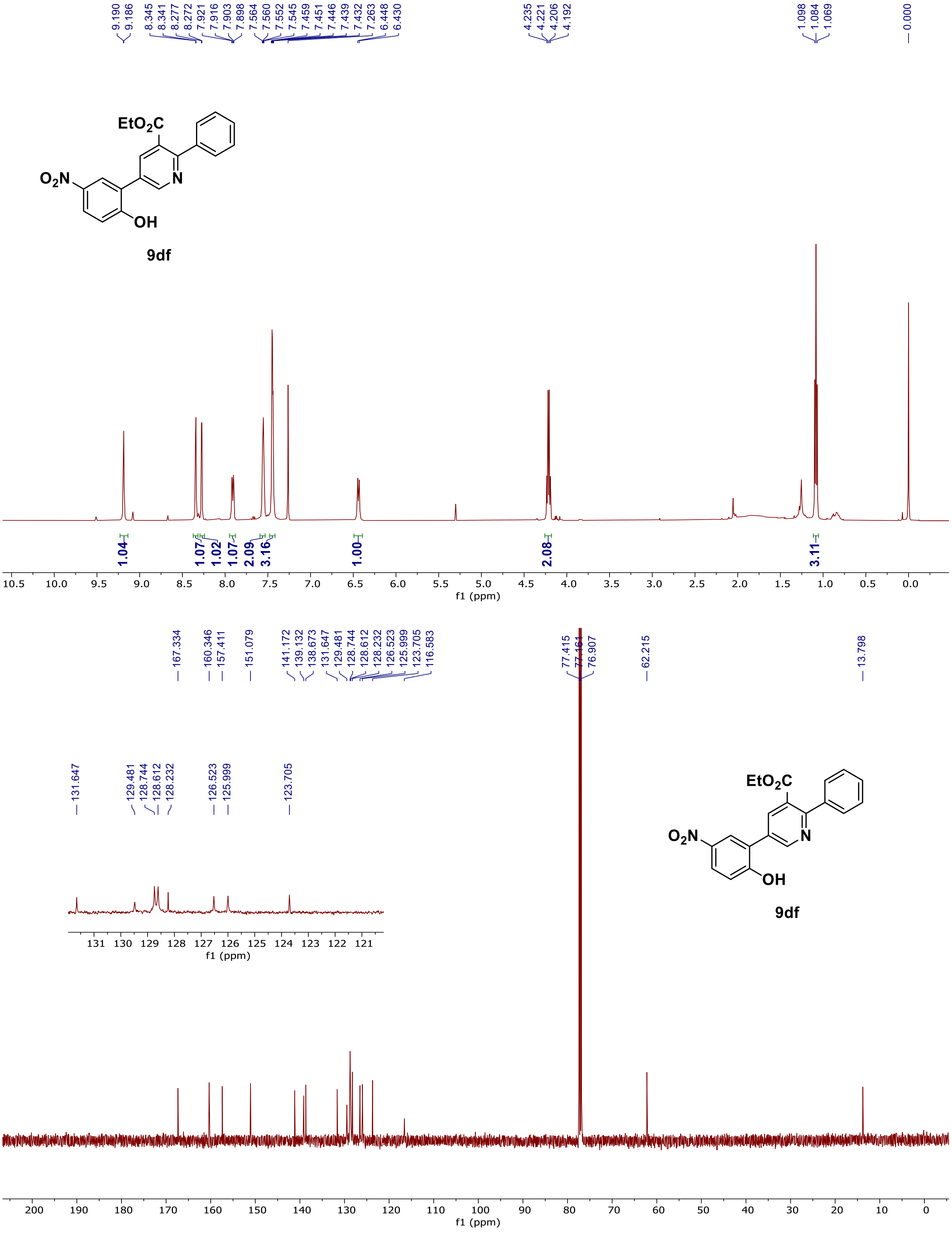

^{1}H and ^{13}C NMR Spectrum of **9df** in $CDCl_3$

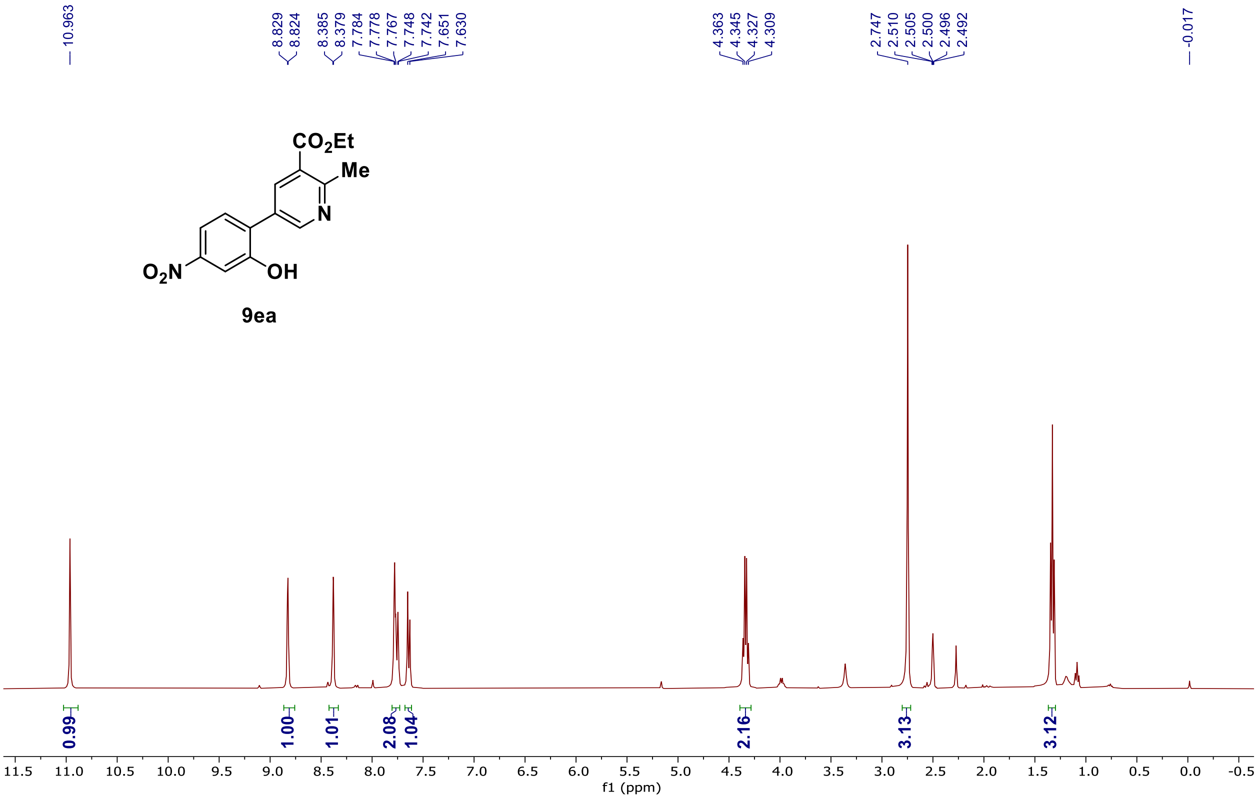

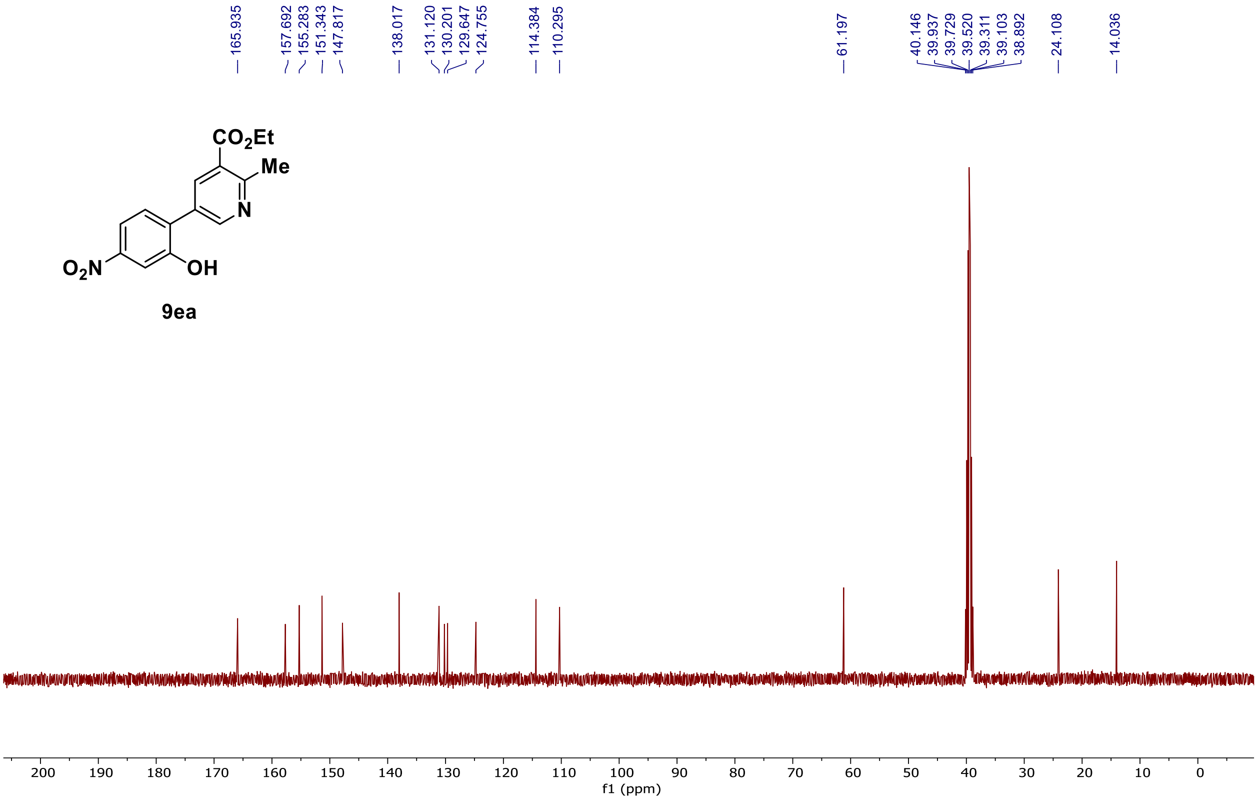

^{1}H and ^{13}C NMR Spectrum of **9ea** in DMSO-d_6

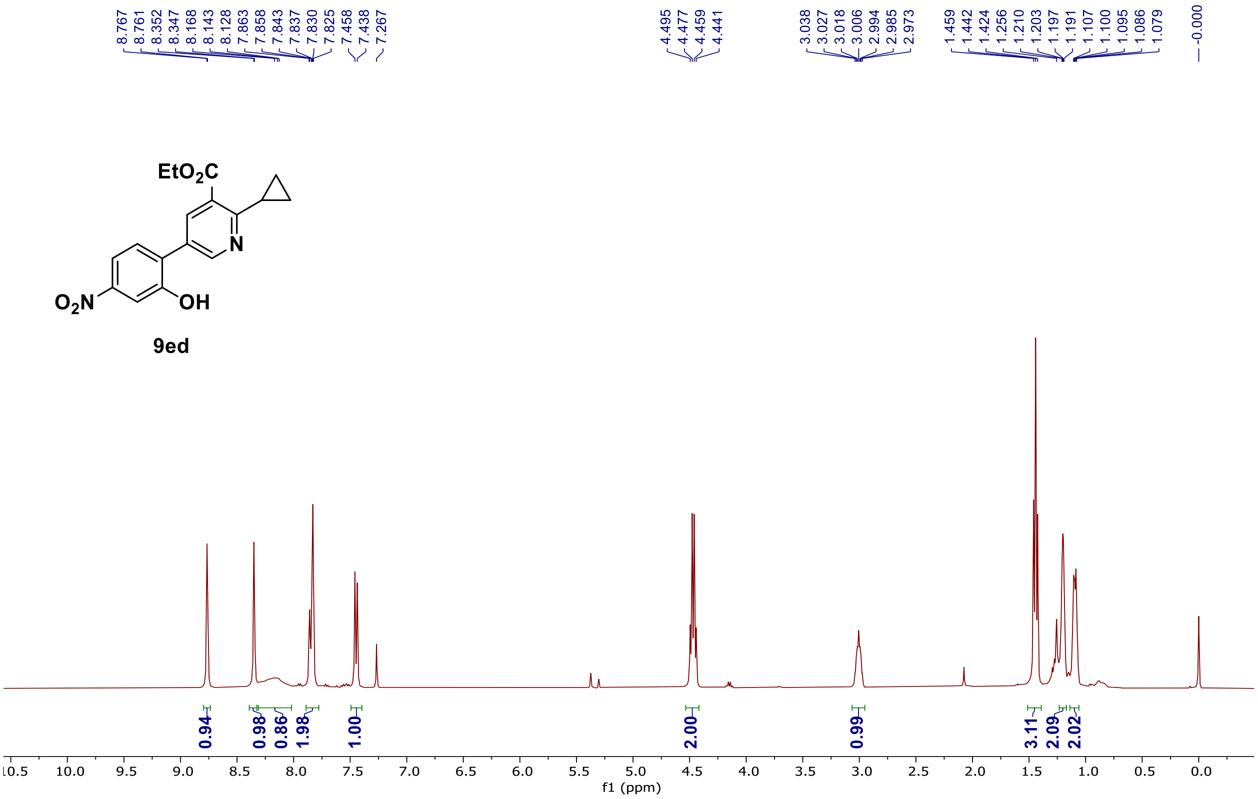

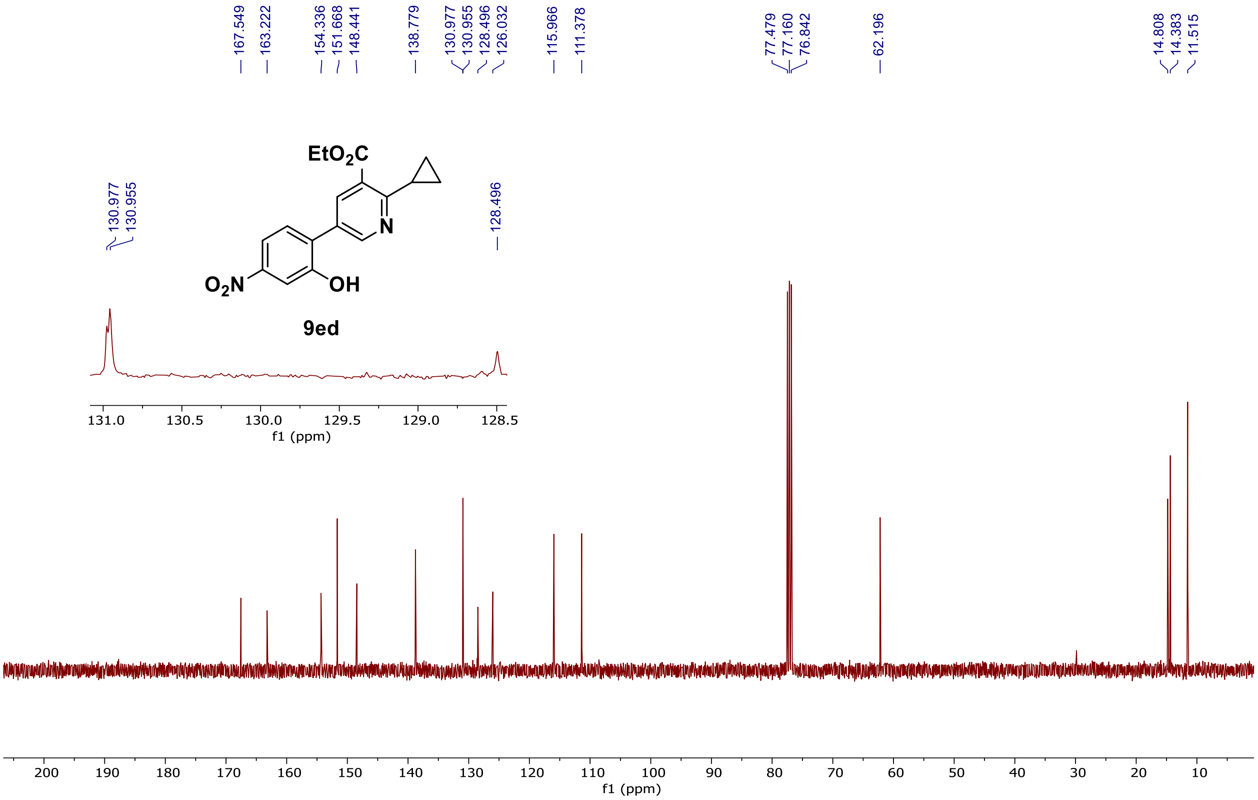

^{1}H and ^{13}C NMR Spectrum of **9ed** in $CDCl_3$

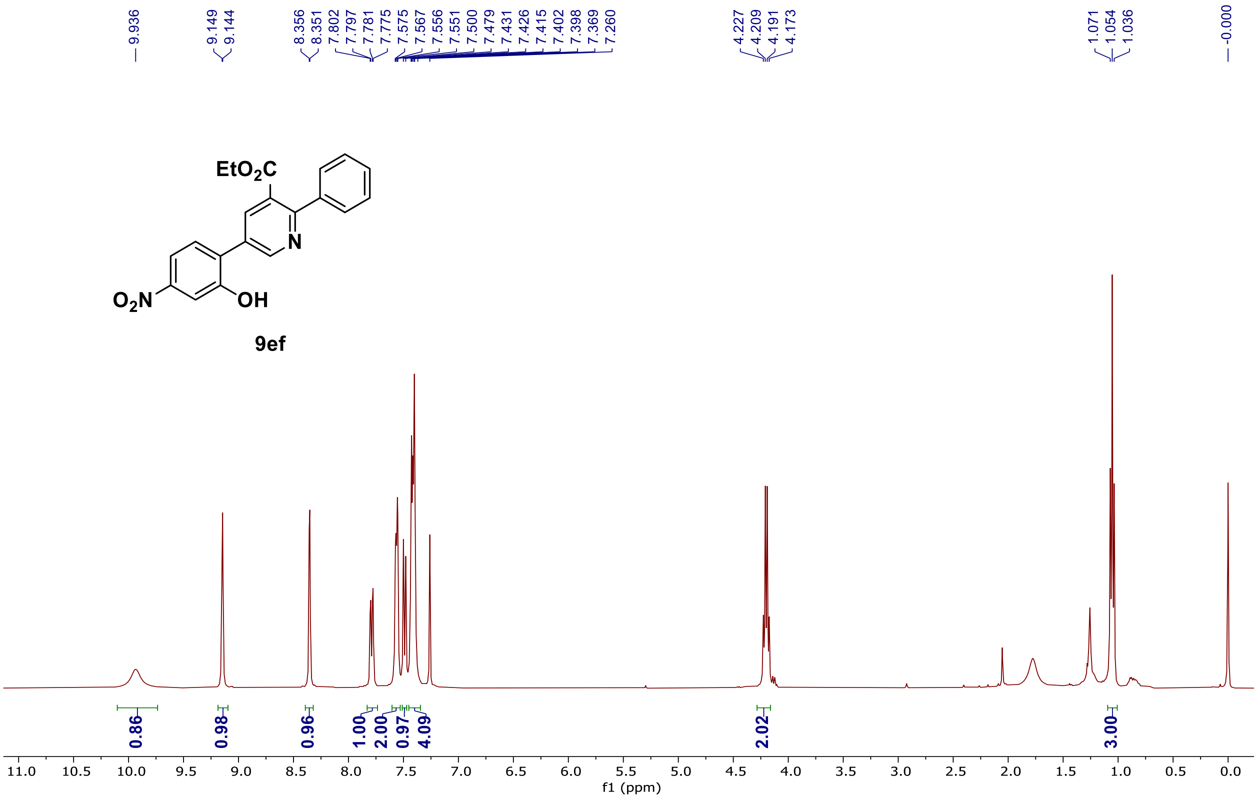

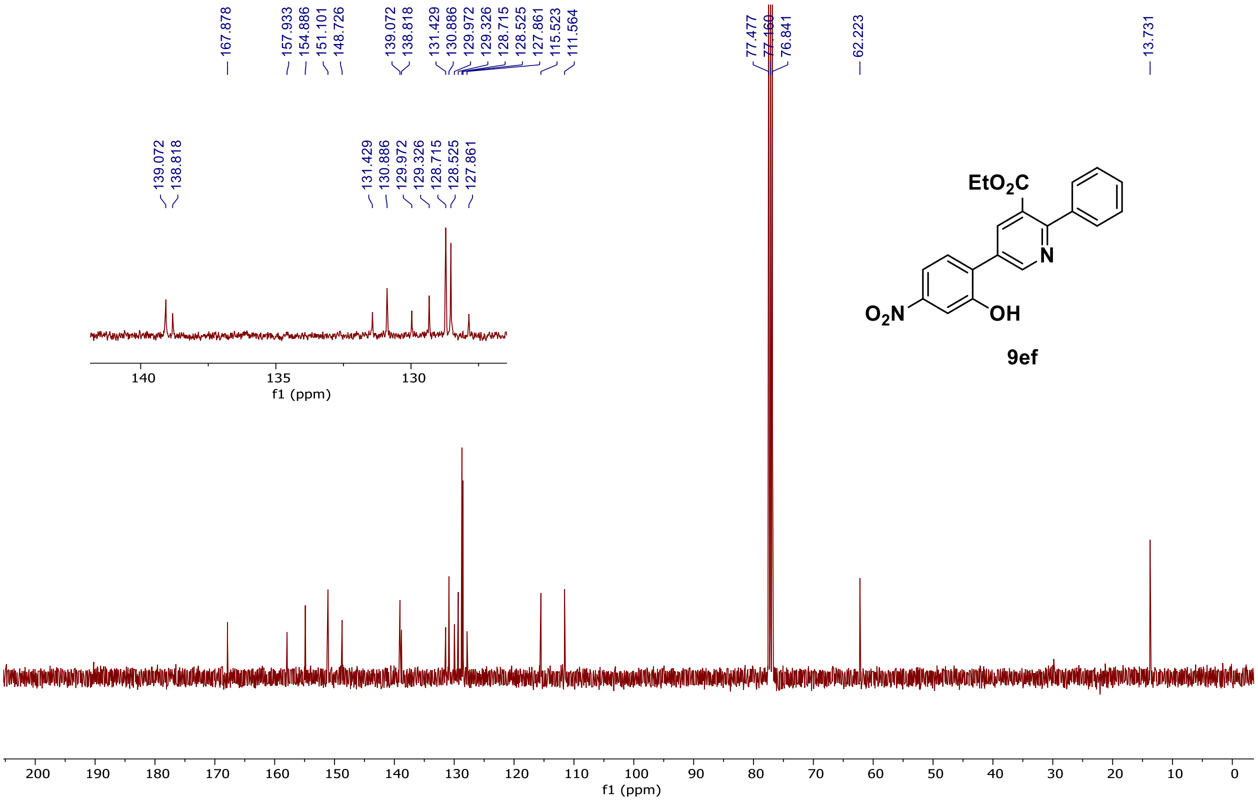

^{1}H and ^{13}C NMR Spectrum of **9ef** in $CDCl_3$

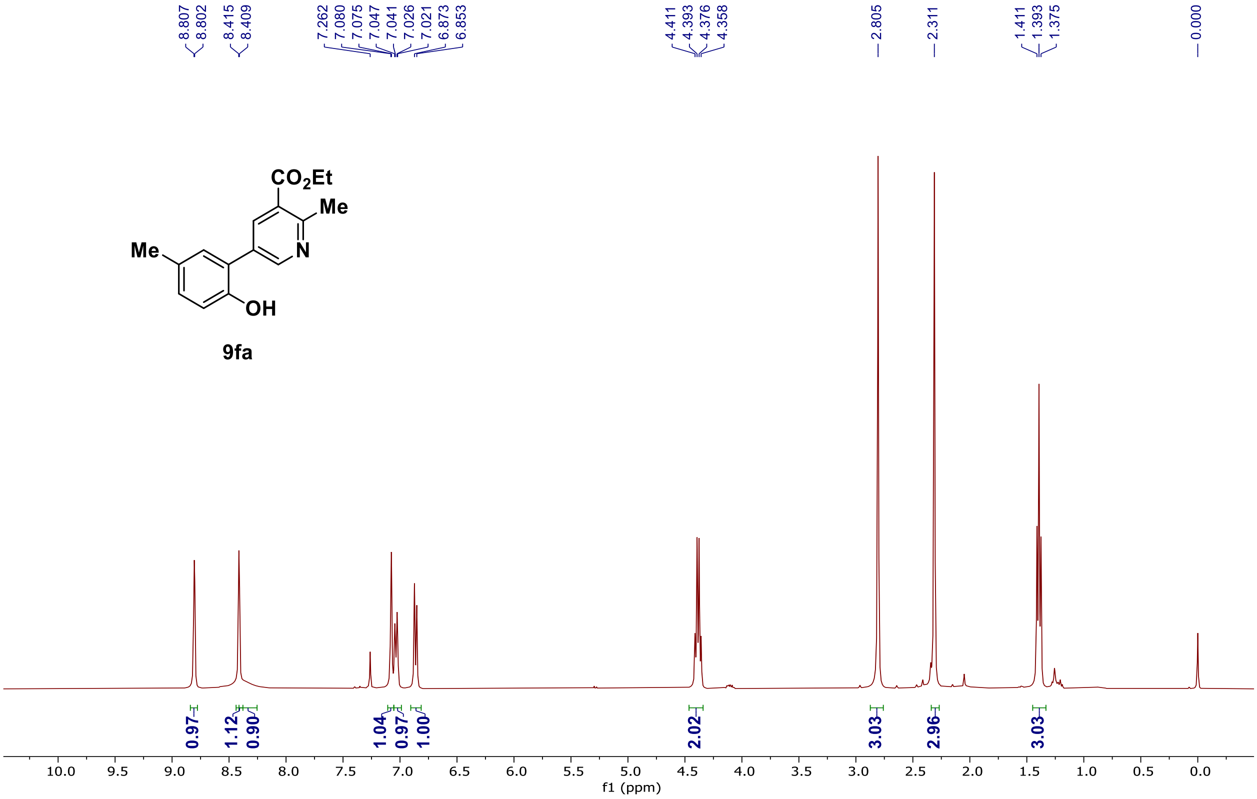

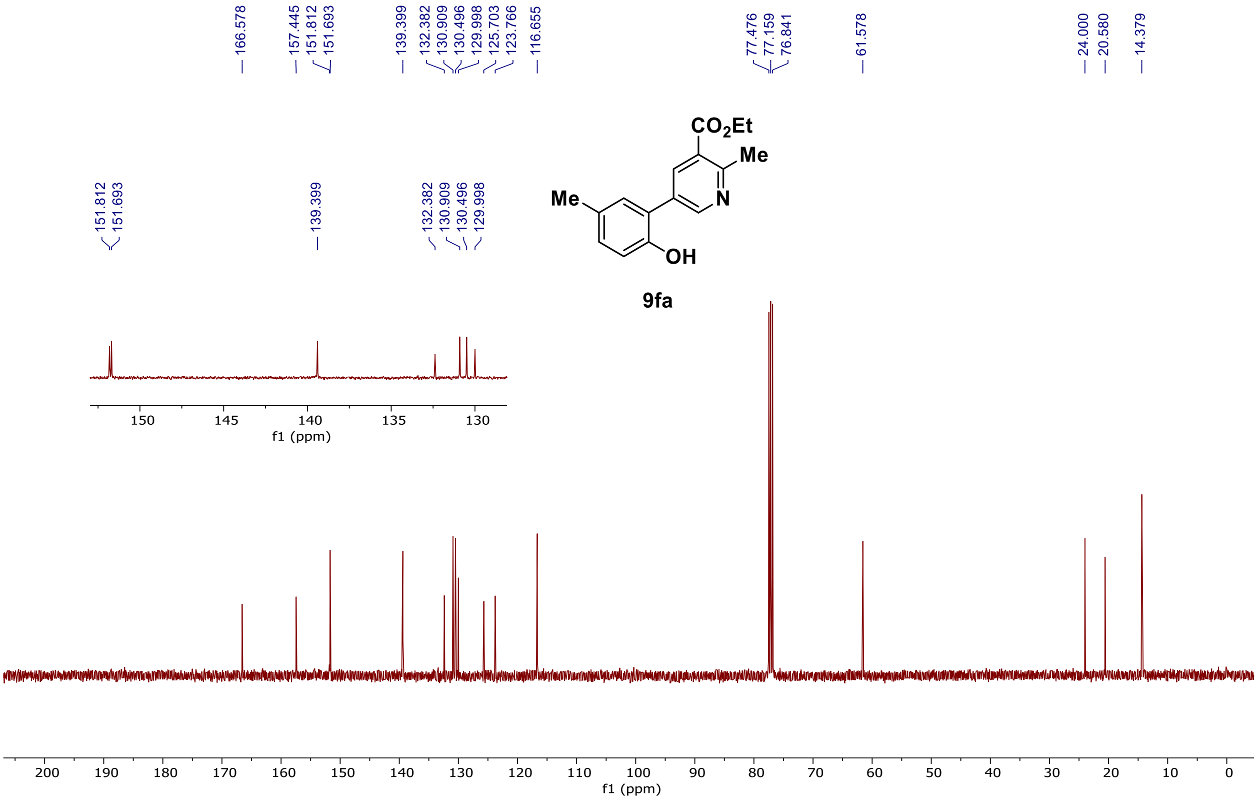

^{1}H and ^{13}C NMR Spectrum of **9fa** in $CDCl_3$

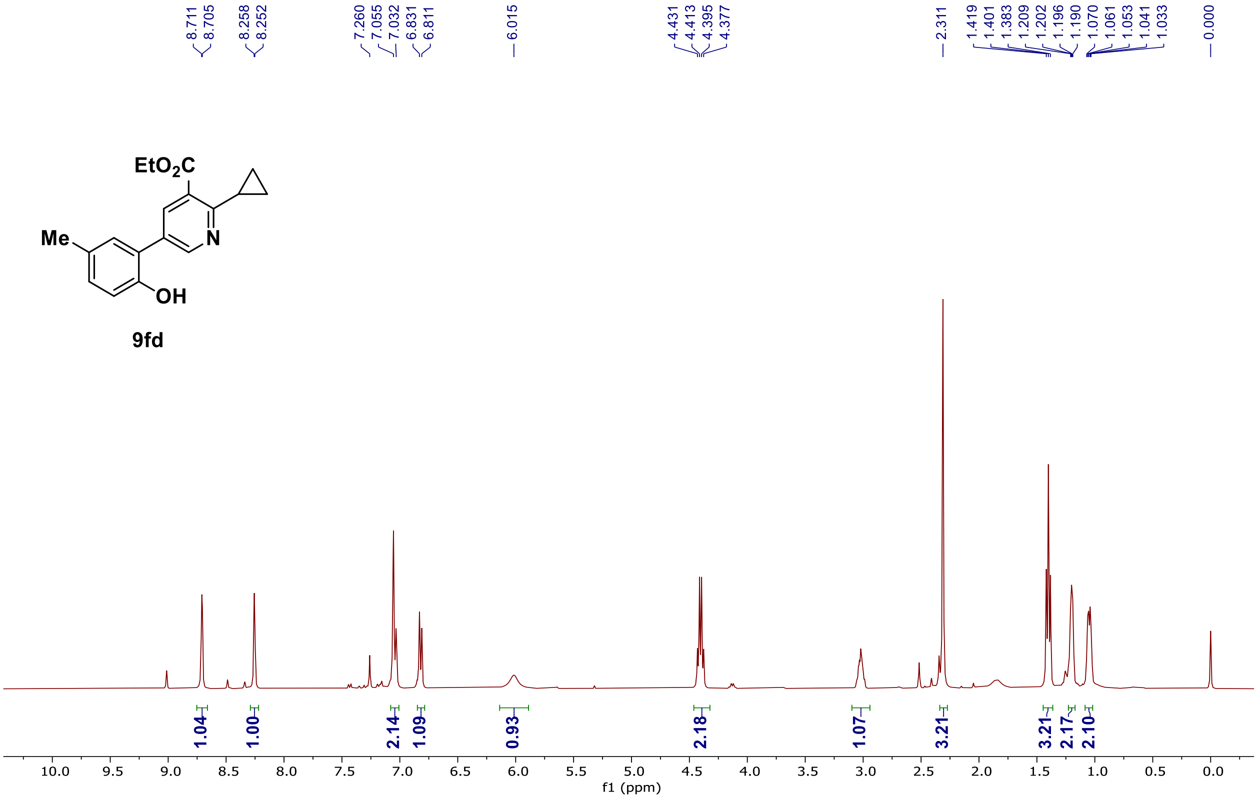

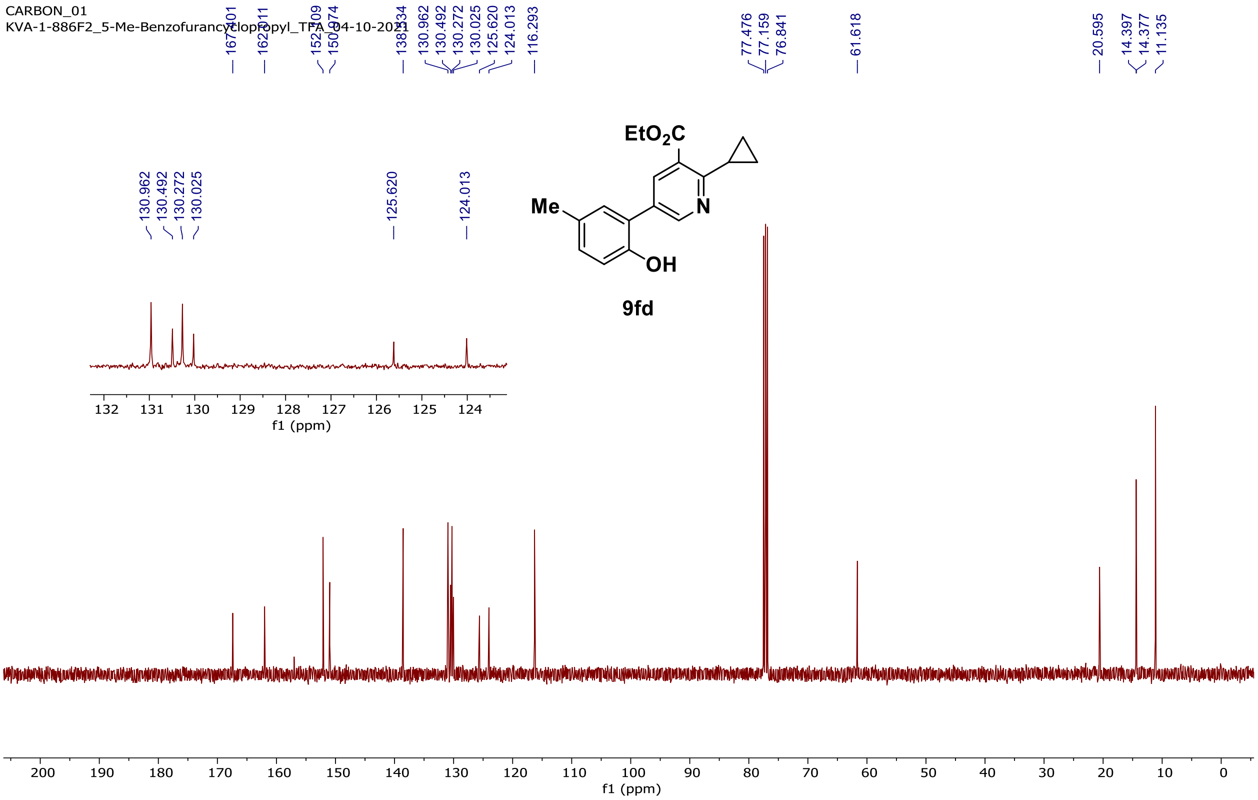

^{1}H and ^{13}C NMR Spectrum of **9fd** in $CDCl_3$

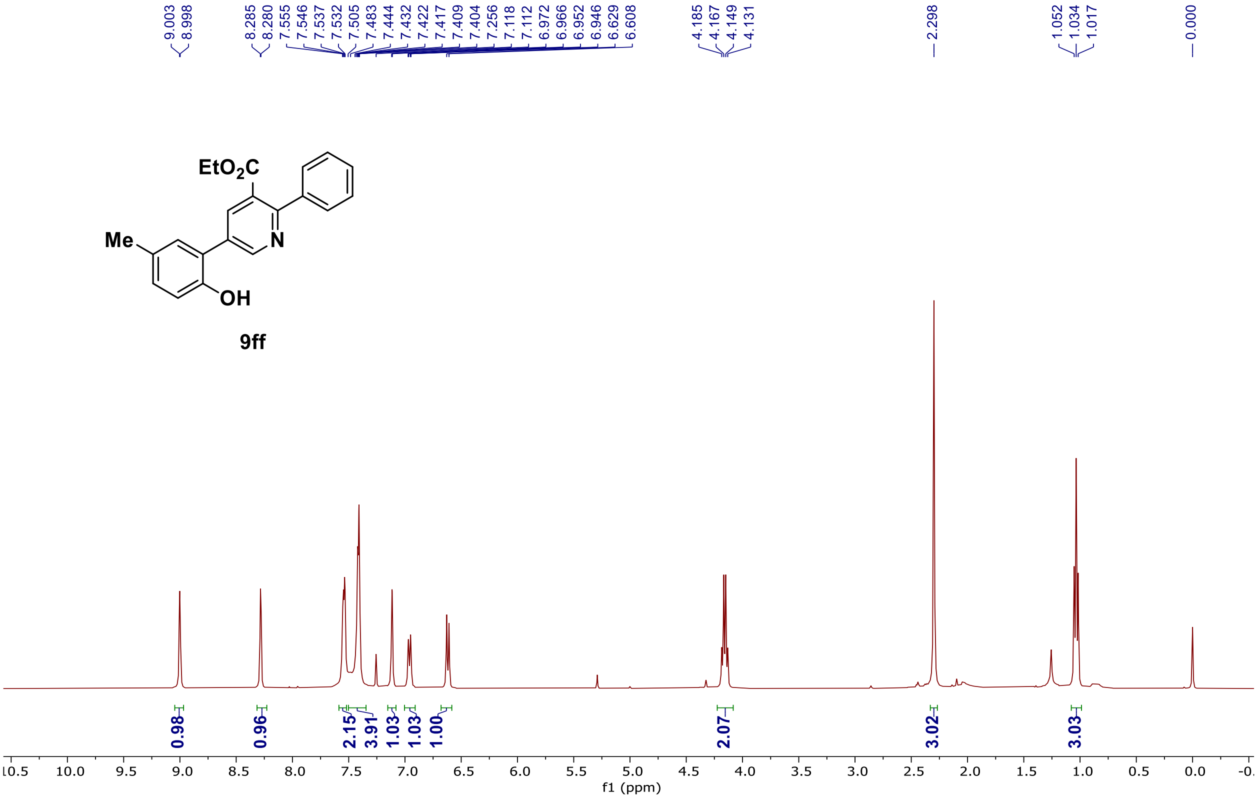

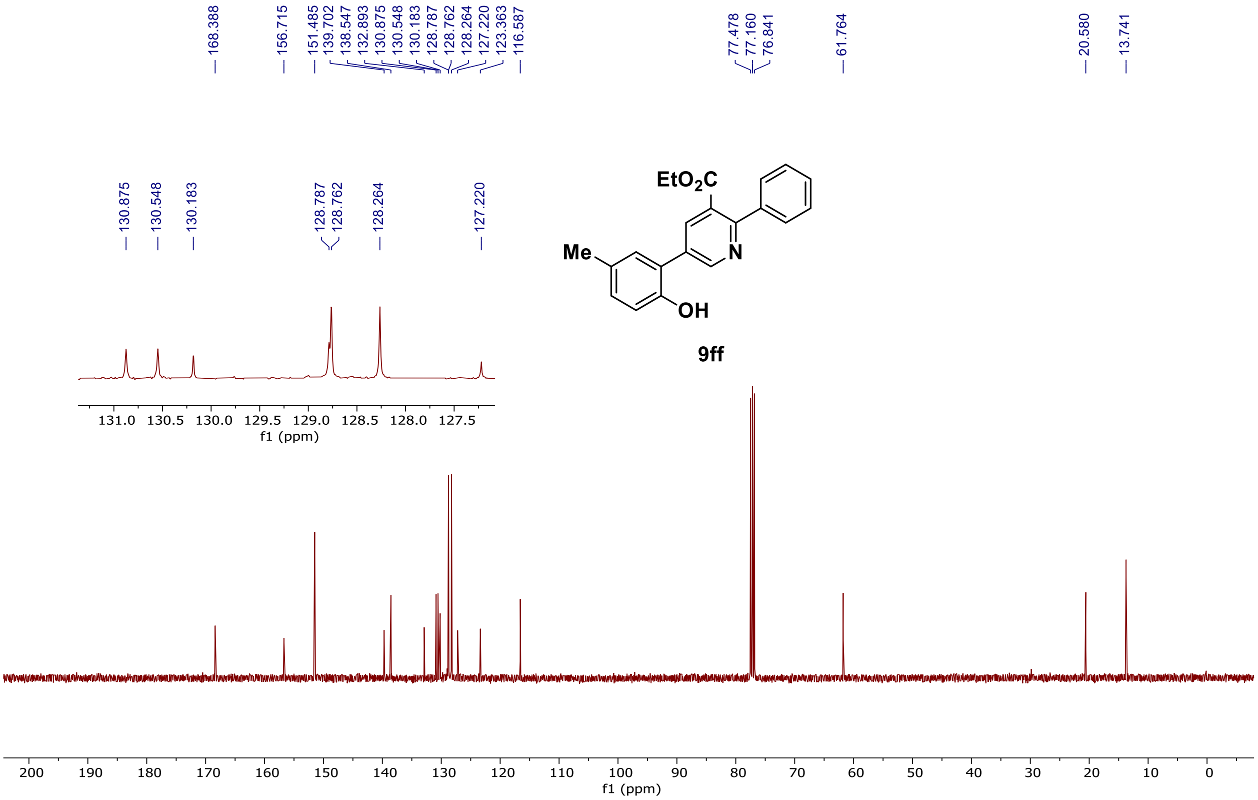

^{1}H and ^{13}C NMR Spectrum of **9ff** in $CDCl_3$

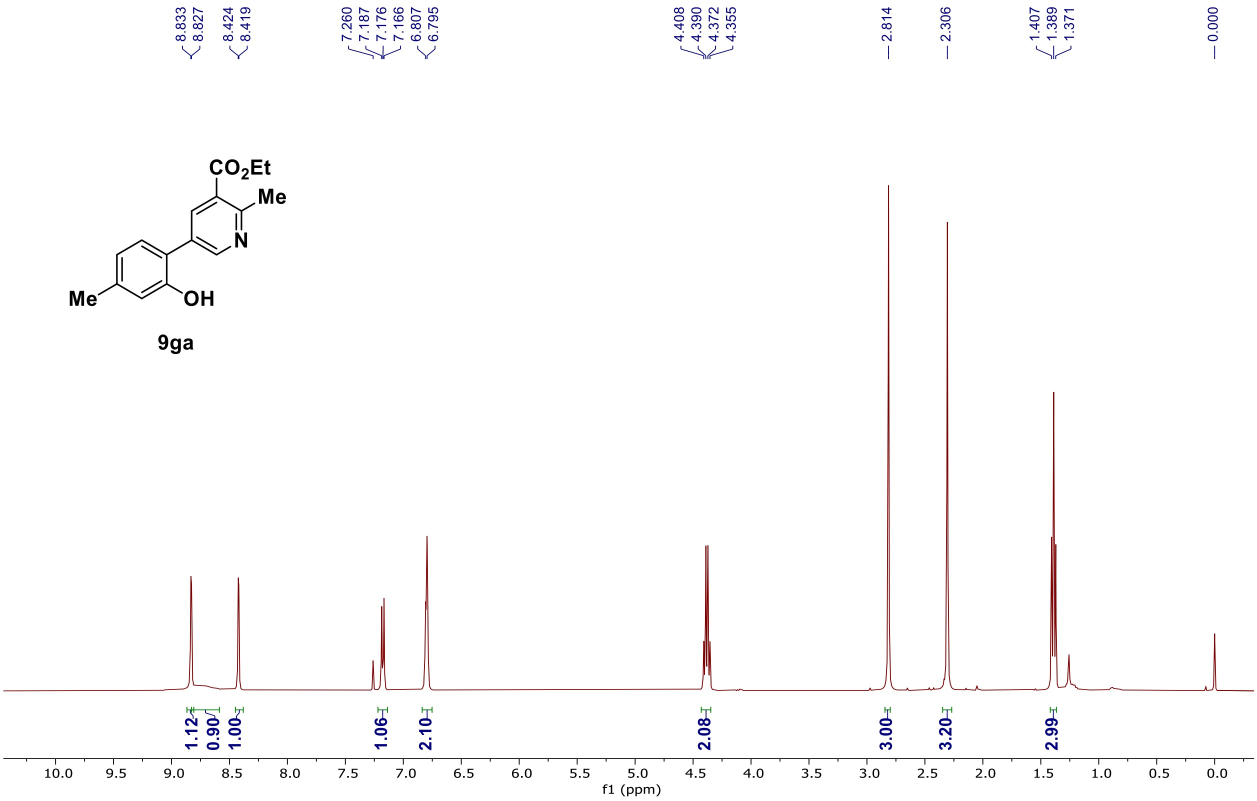

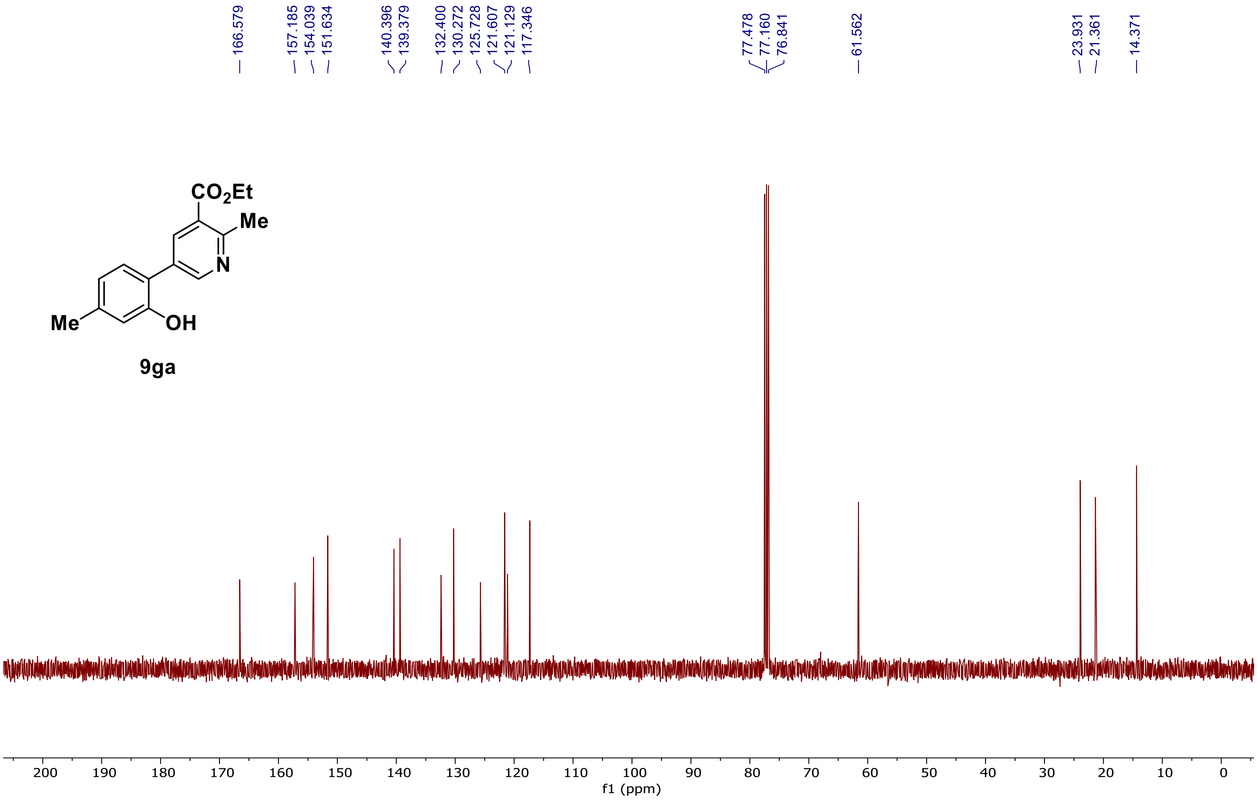

^{1}H and ^{13}C NMR Spectrum of **9ga** in $CDCl_3$

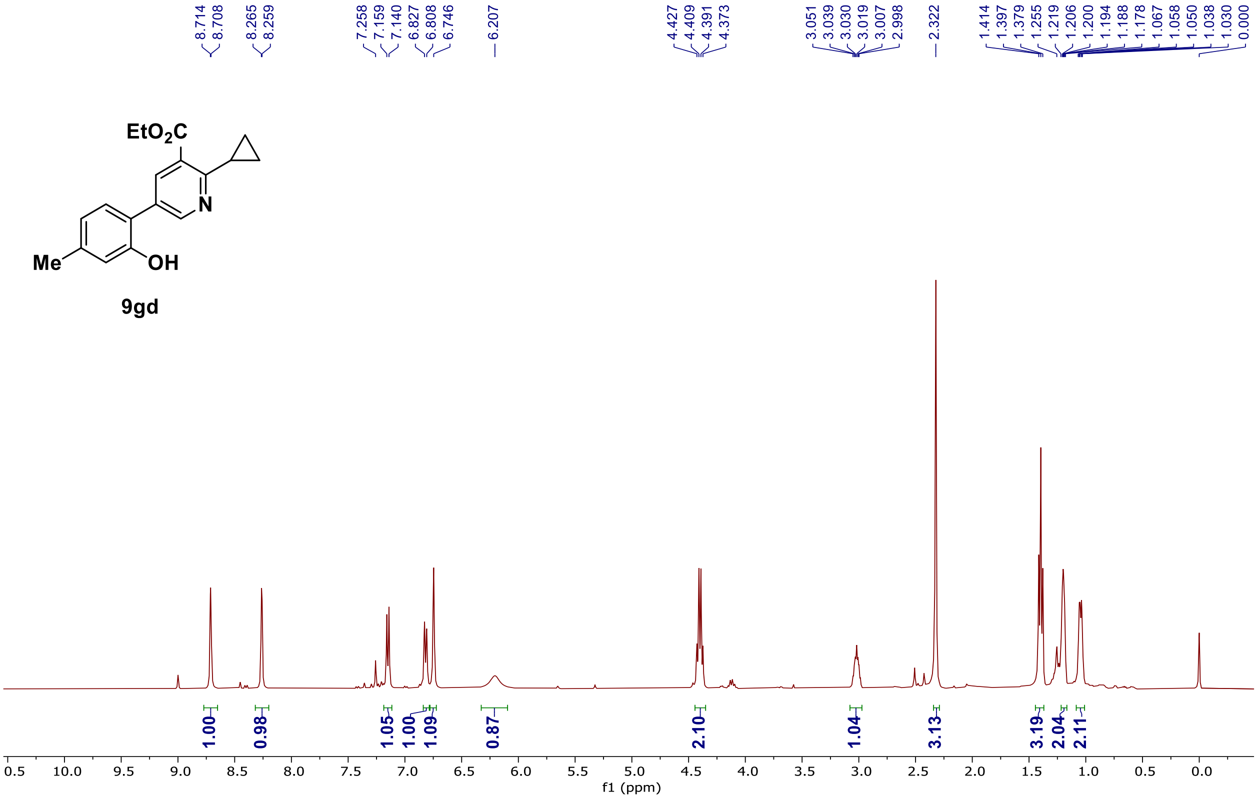

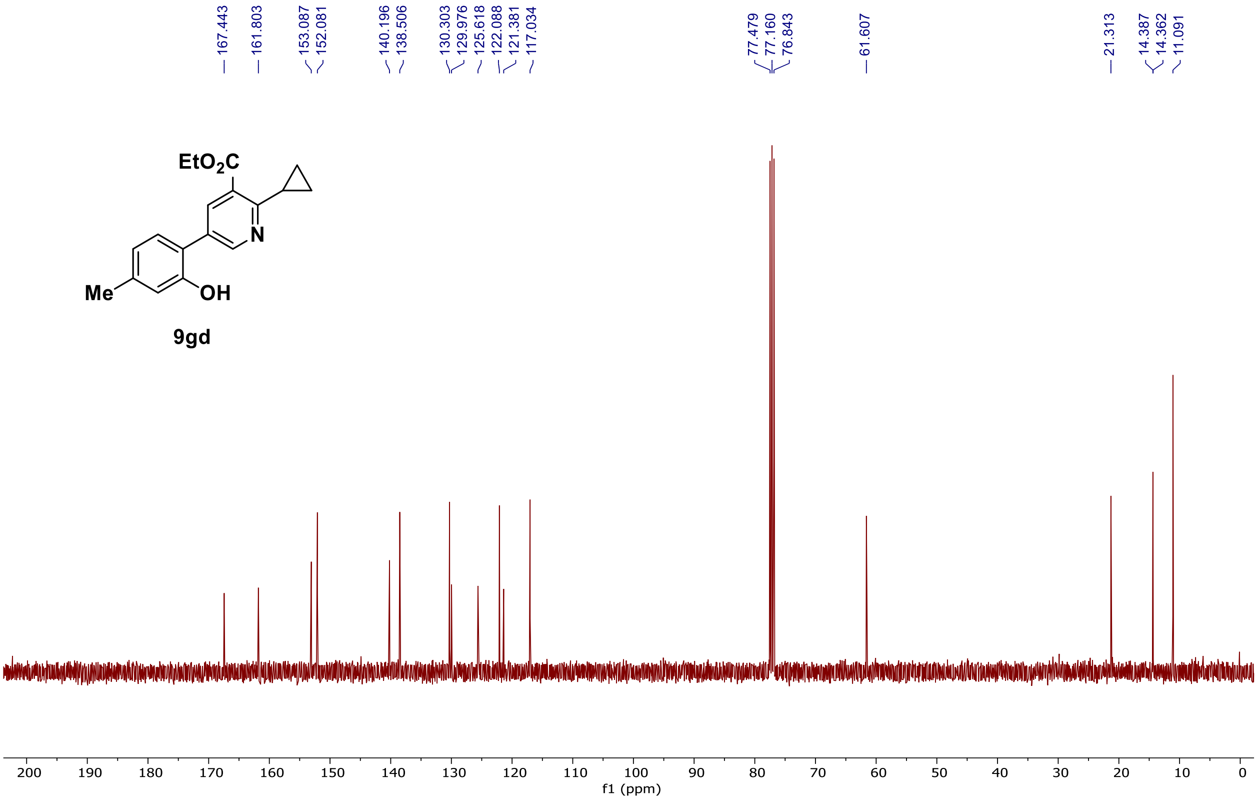

^{1}H and ^{13}C NMR Spectrum of **9gd** in $CDCl_3$

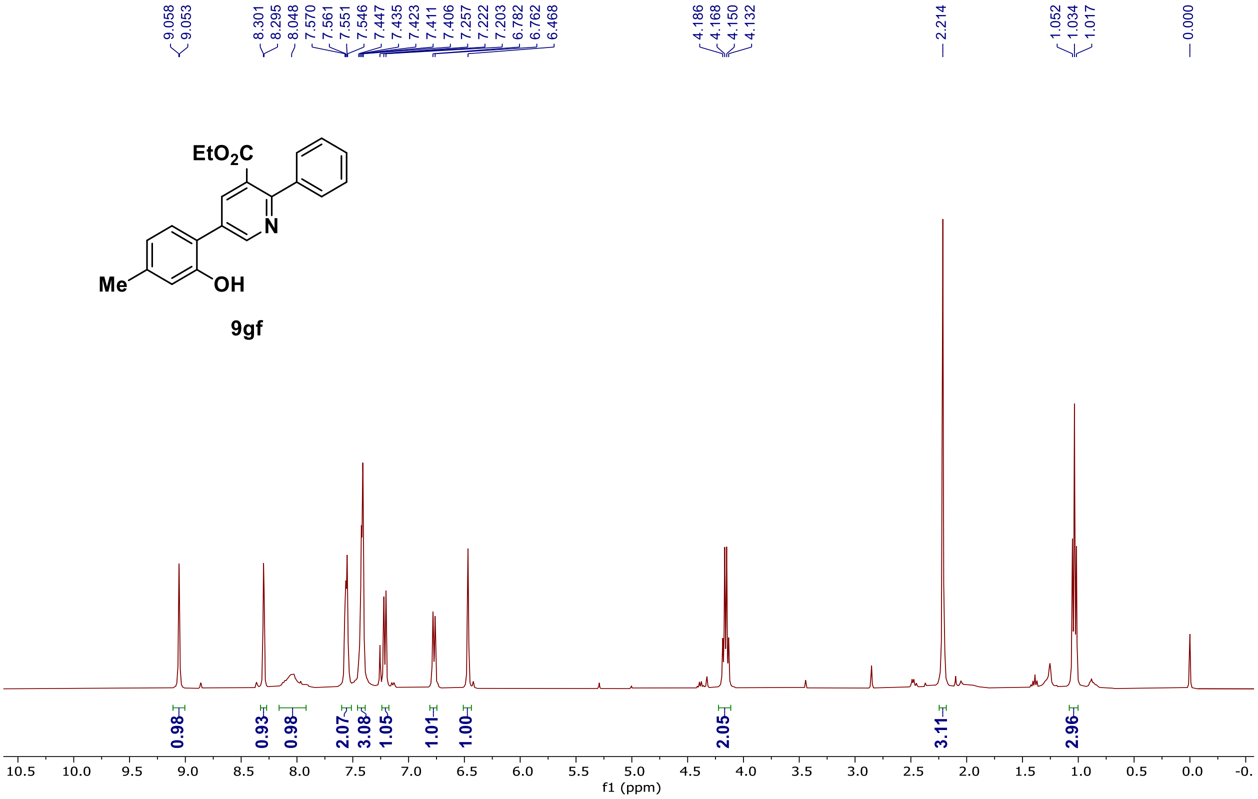

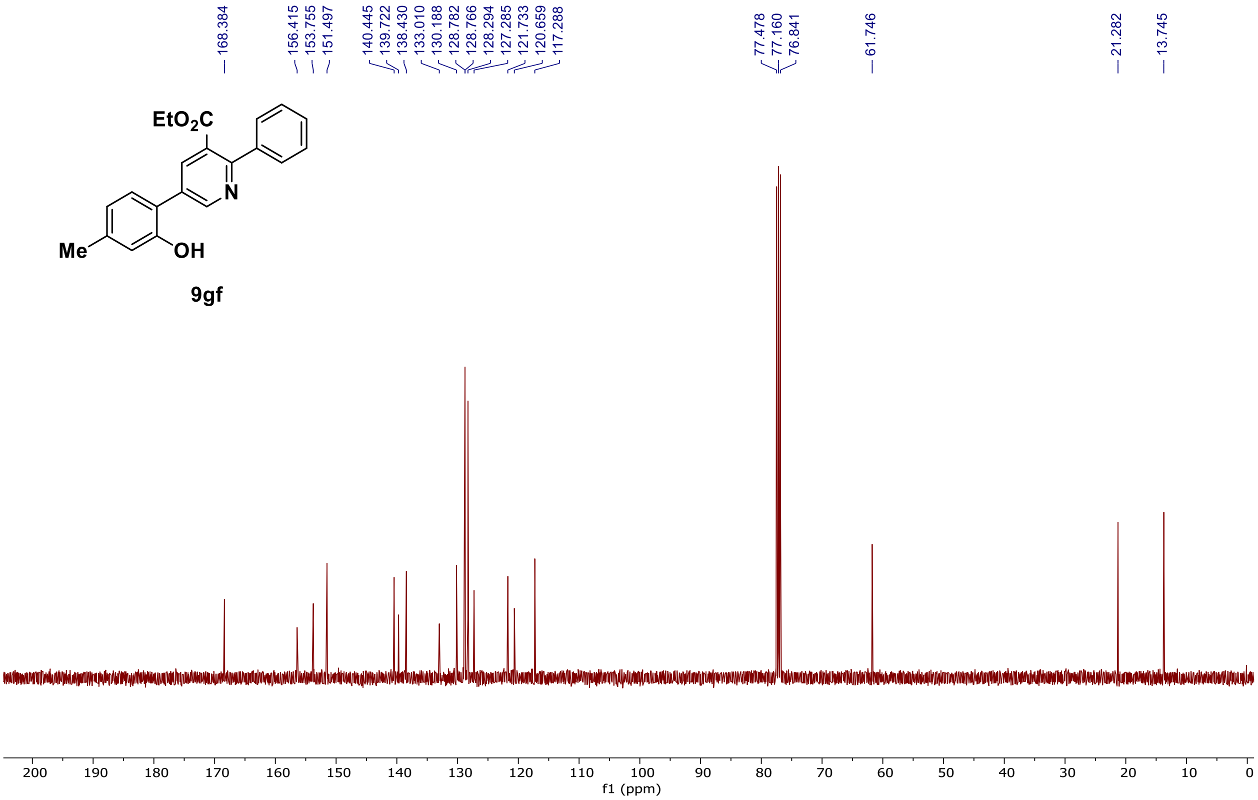

^{1}H and ^{13}C NMR Spectrum of **9gf** in $CDCl_3$

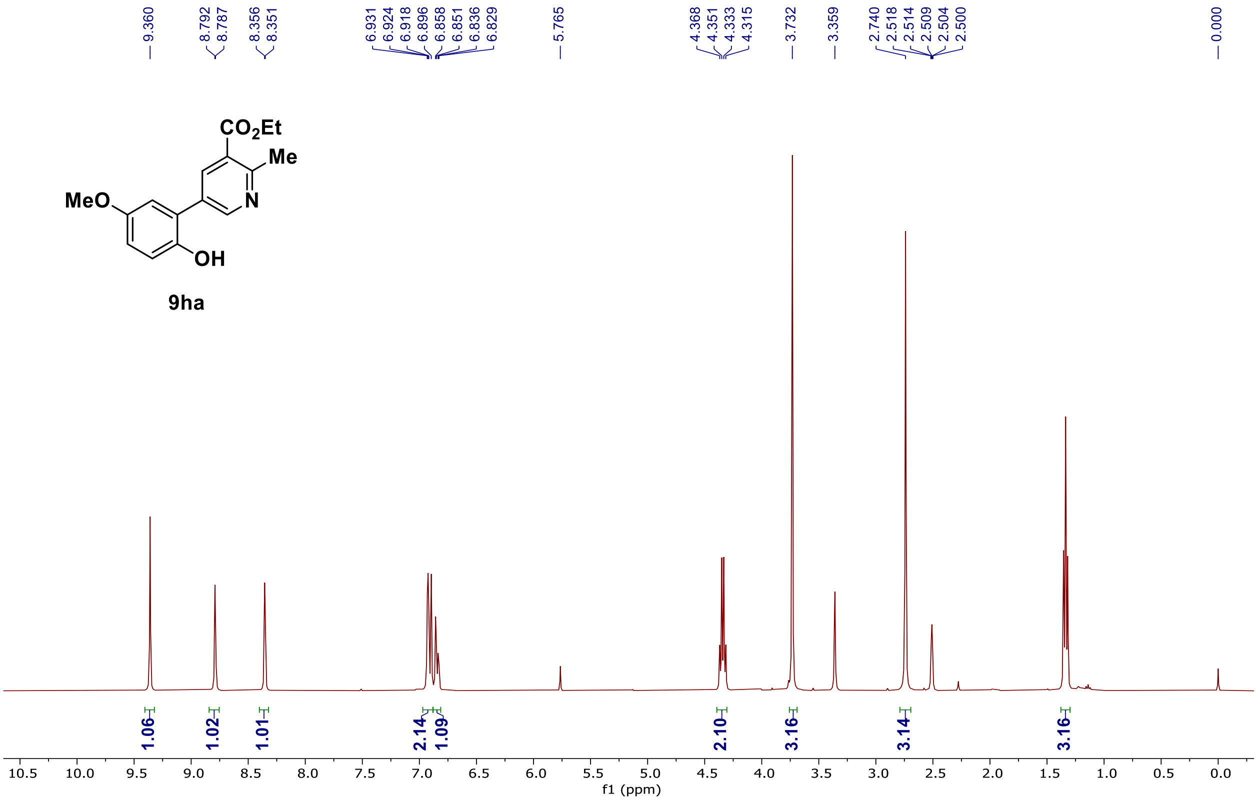

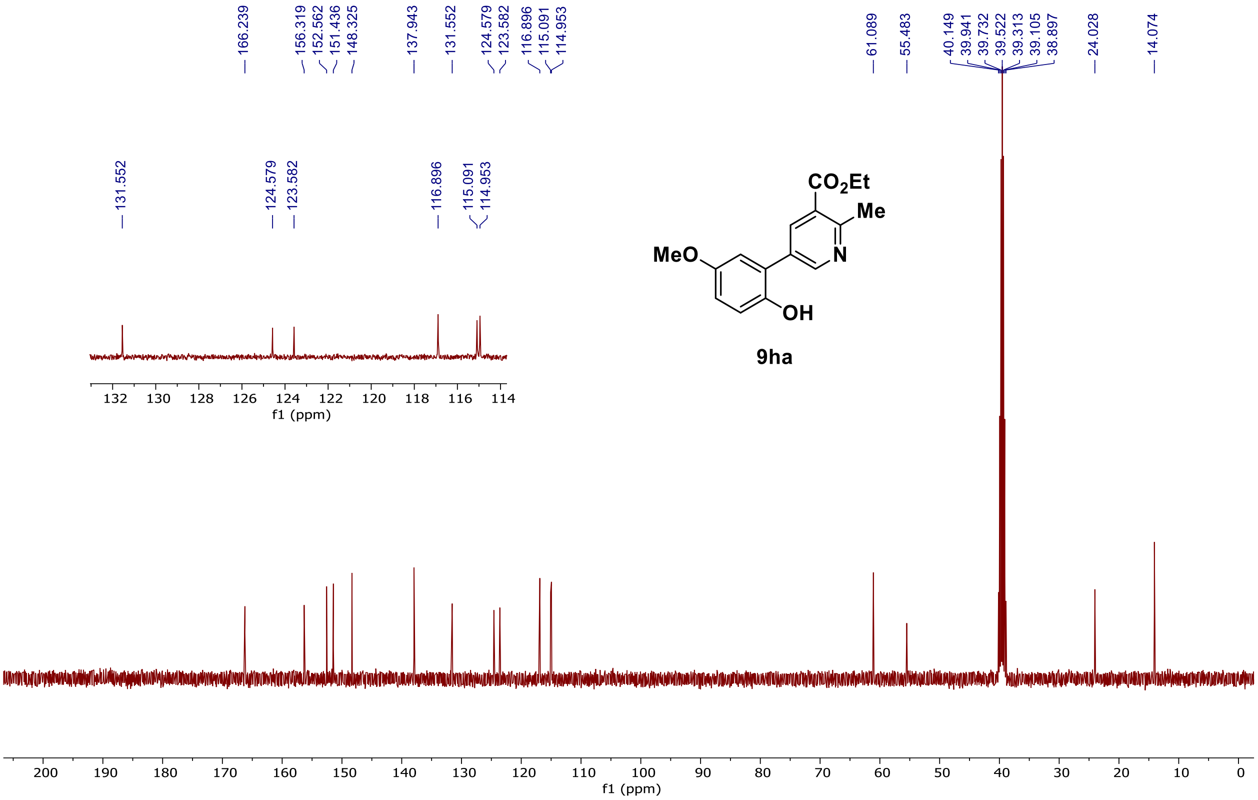

^{1}H and ^{13}C NMR Spectrum of **9ha** in DMSO-d_6

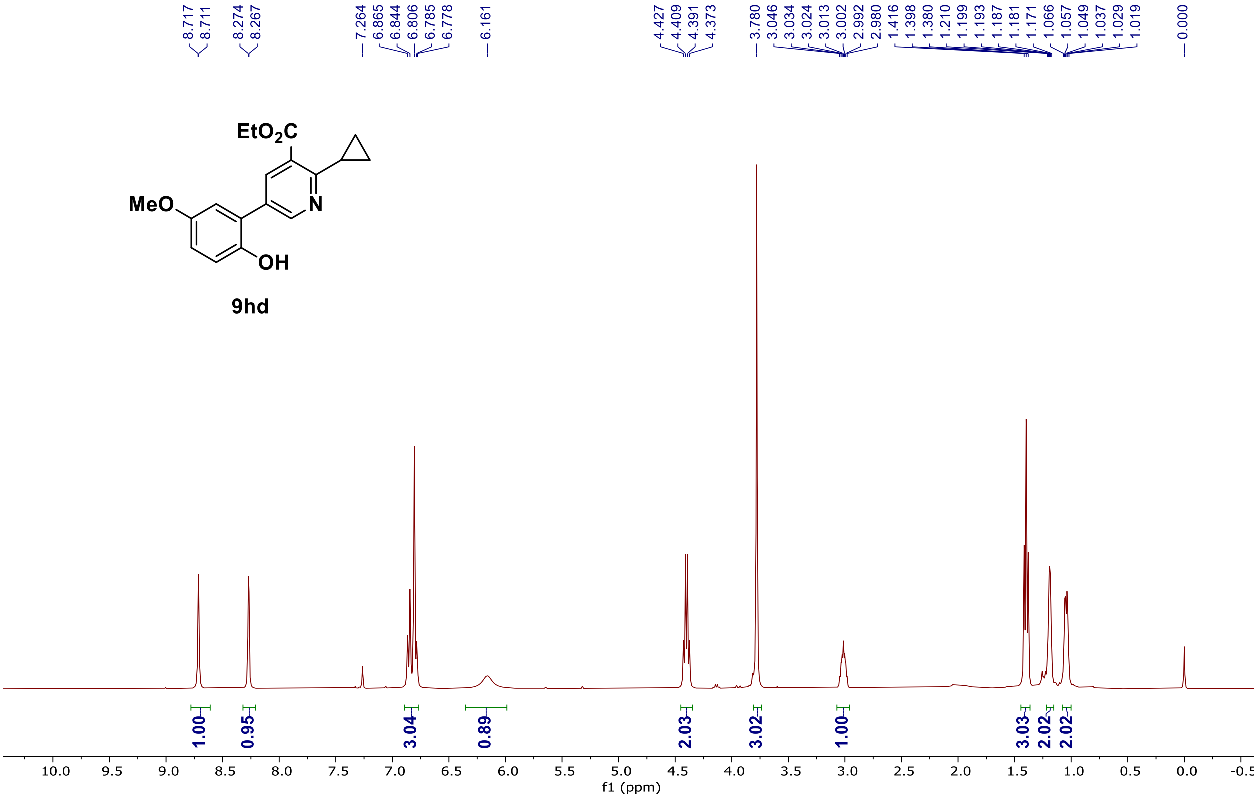

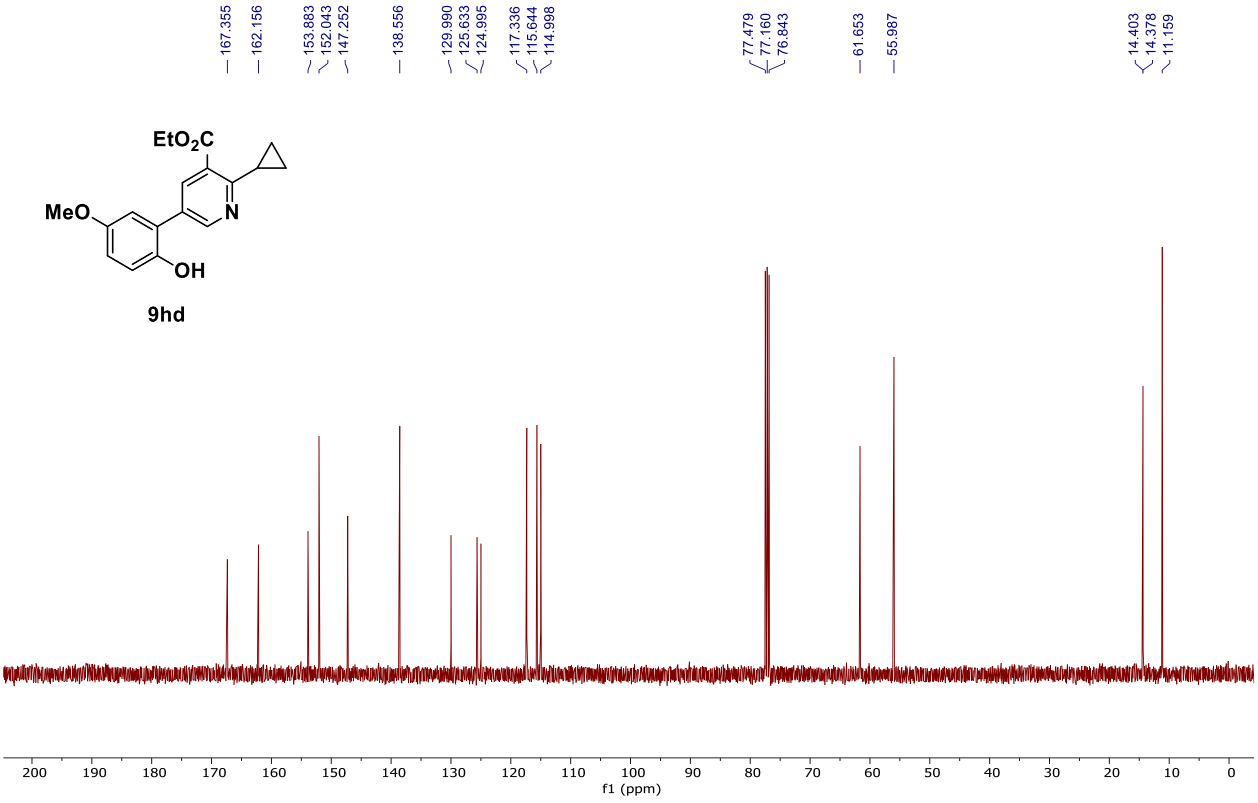

^{1}H and ^{13}C NMR Spectrum of **9hd** in $CDCl_3$

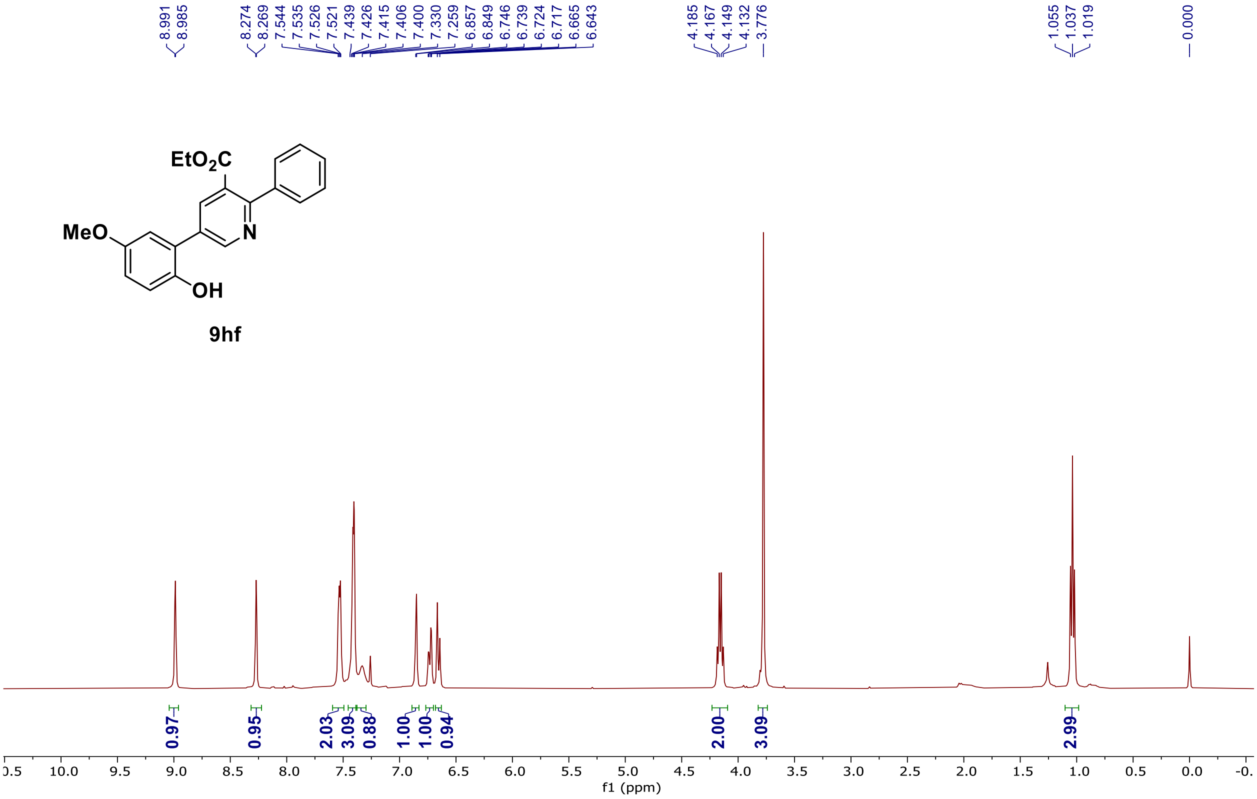

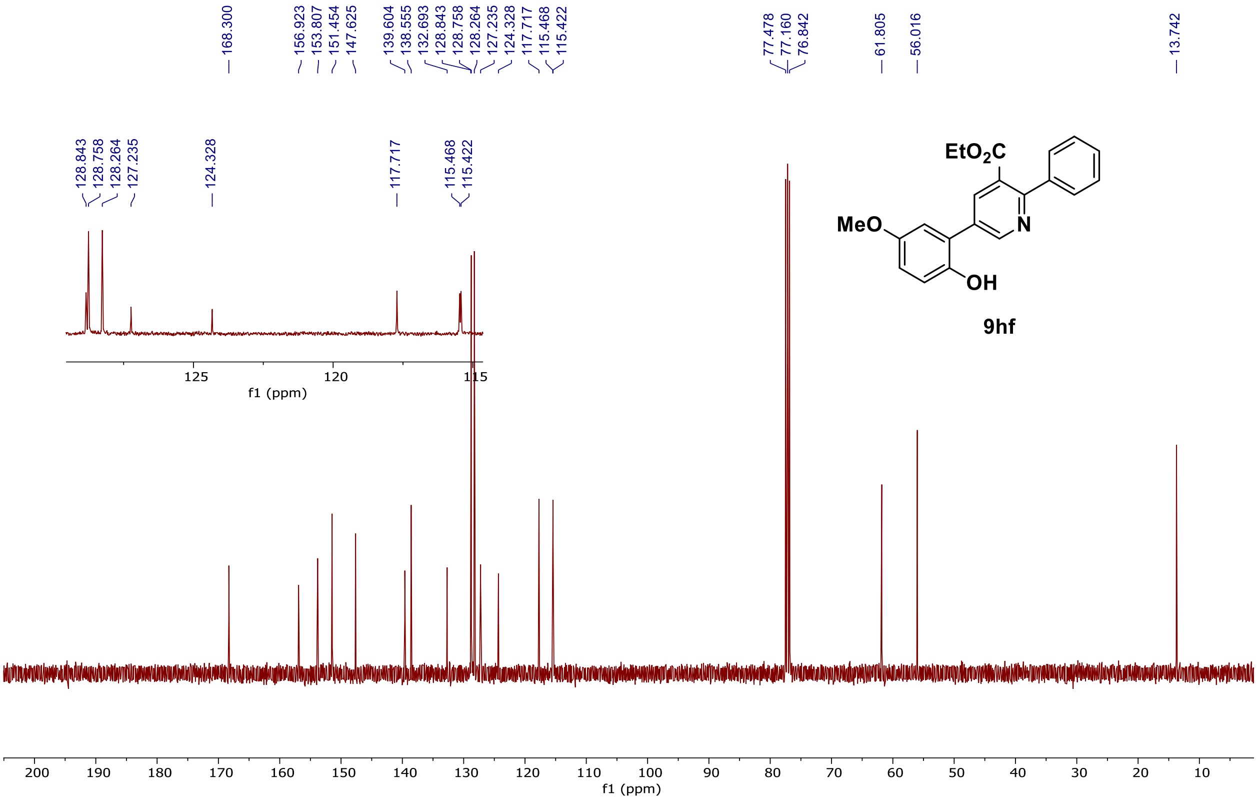

^{1}H and ^{13}C NMR Spectrum of **9hf** in $CDCl_3$

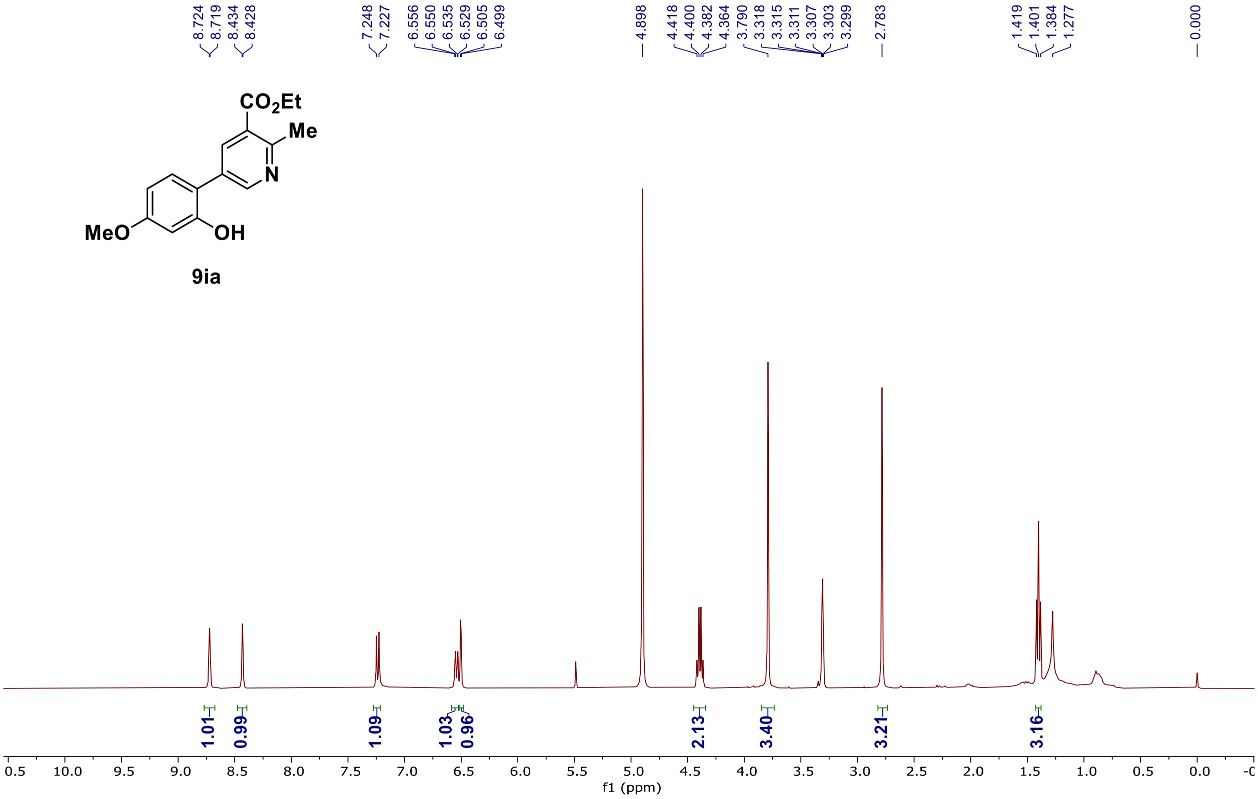

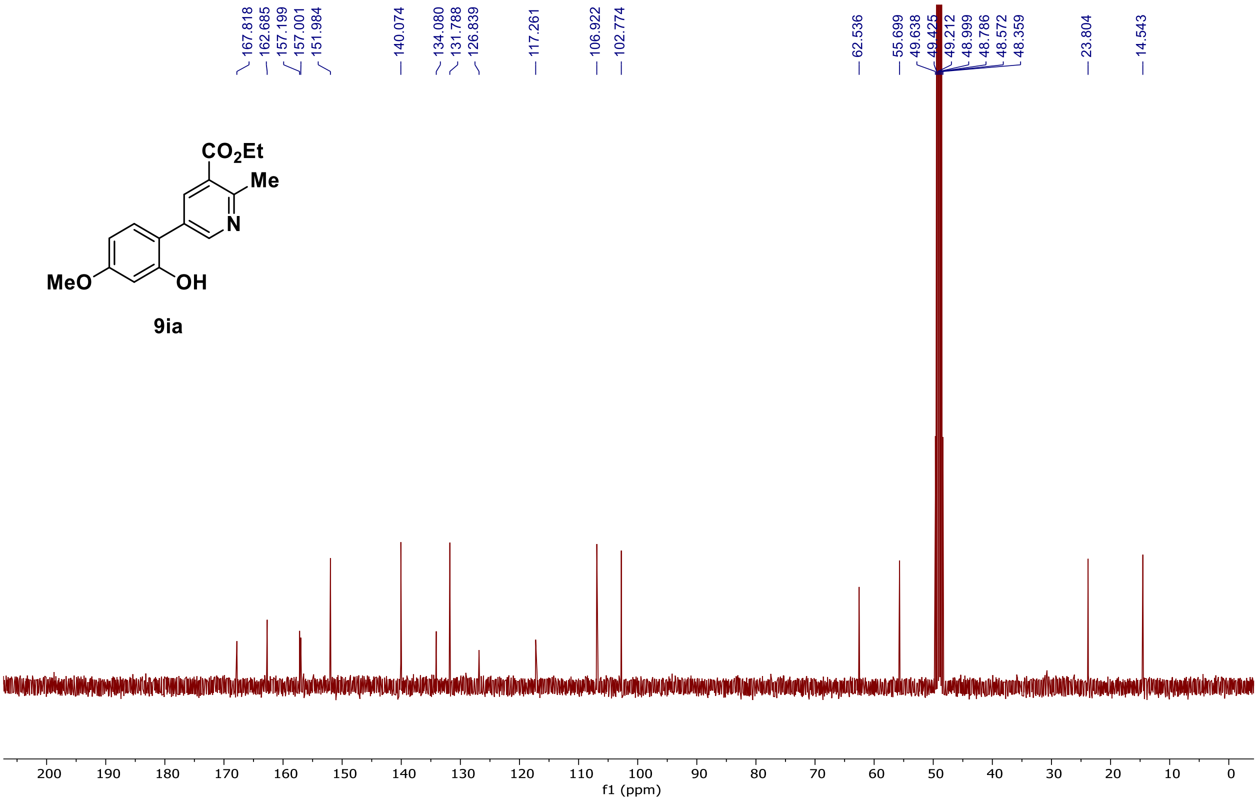

^{1}H and ^{13}C NMR Spectrum of **9ia** in CD_3OD

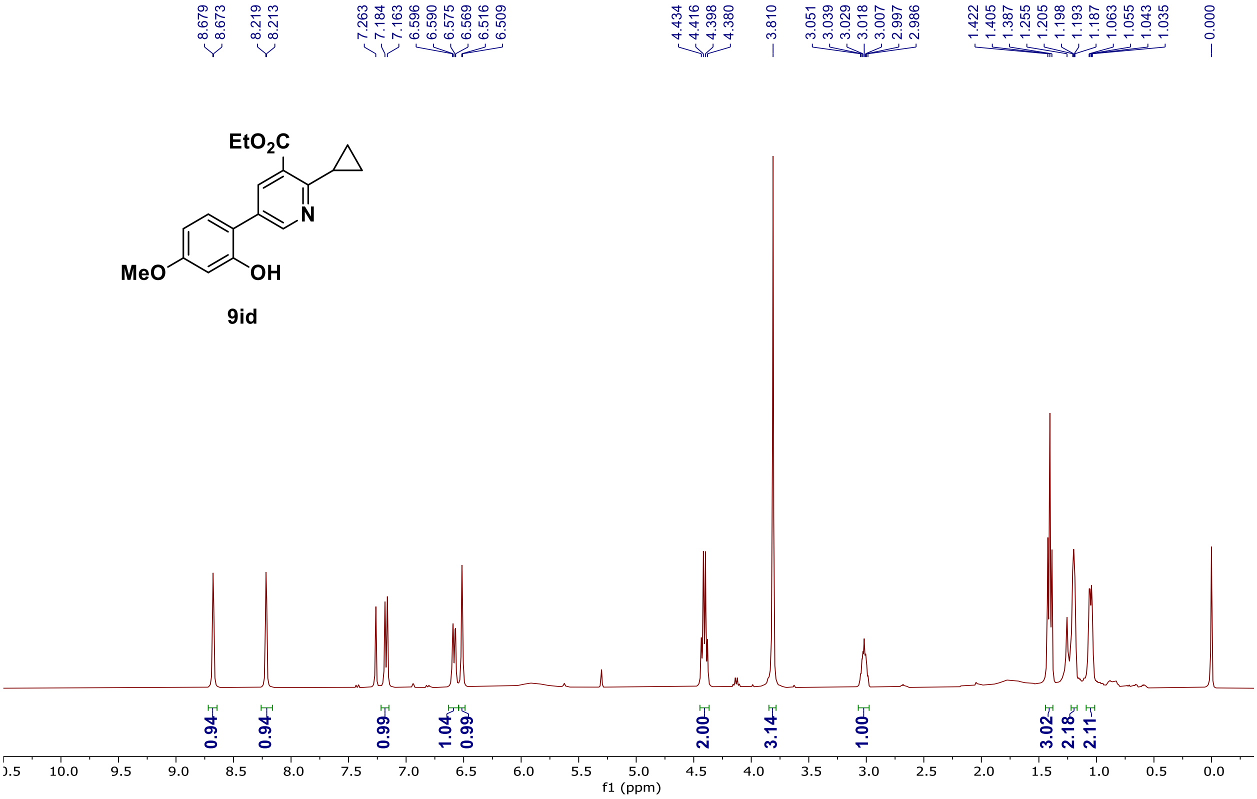

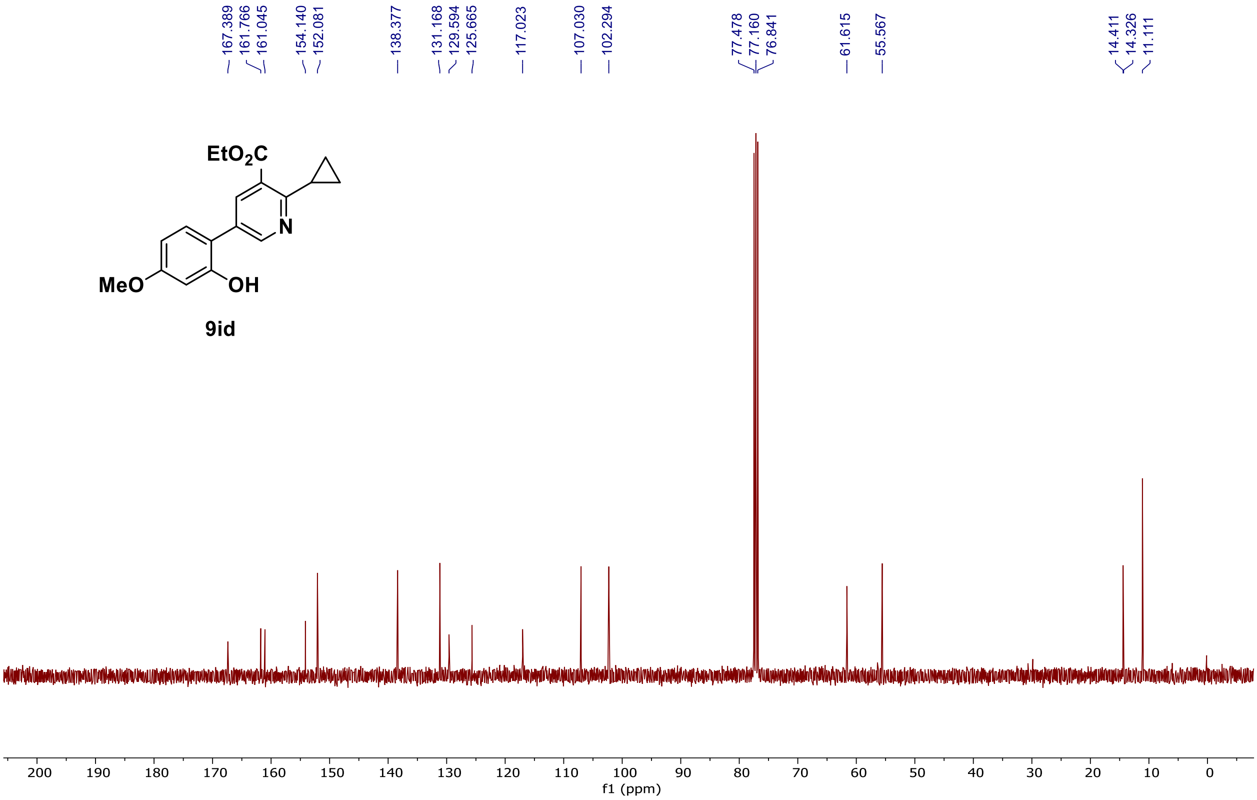

^{1}H and ^{13}C NMR Spectrum of **9id** in $CDCl_3$

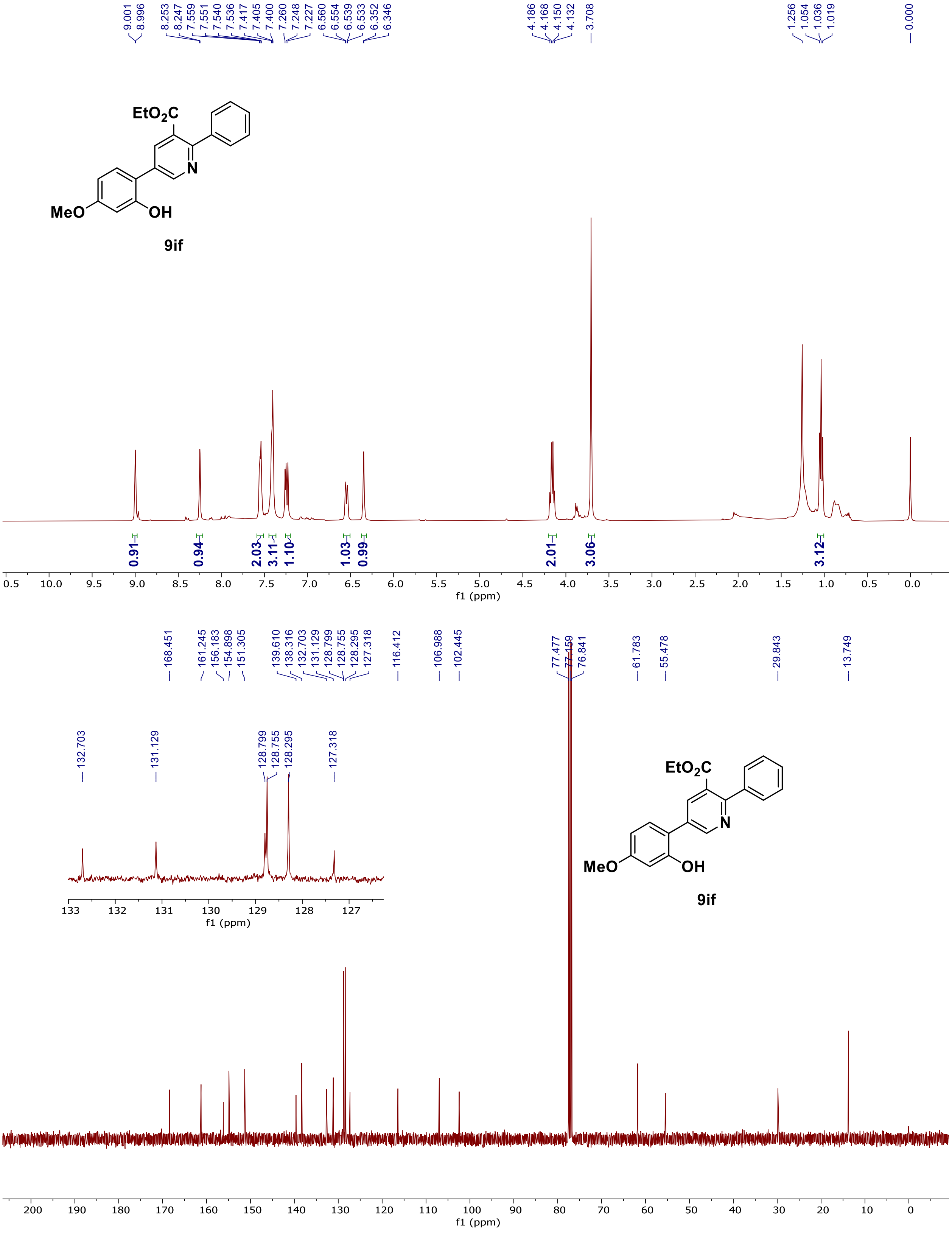

^{1}H and ^{13}C NMR Spectrum of **9if** in $CDCl_3$

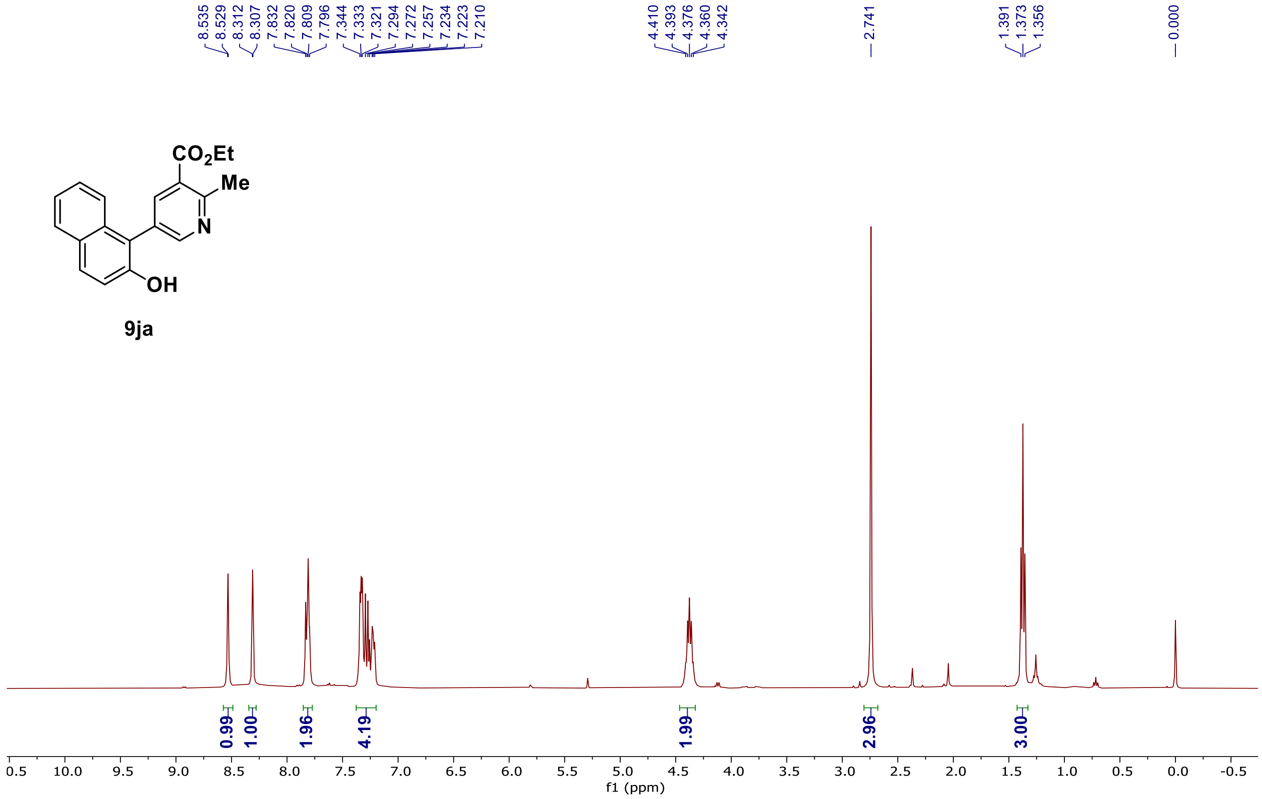

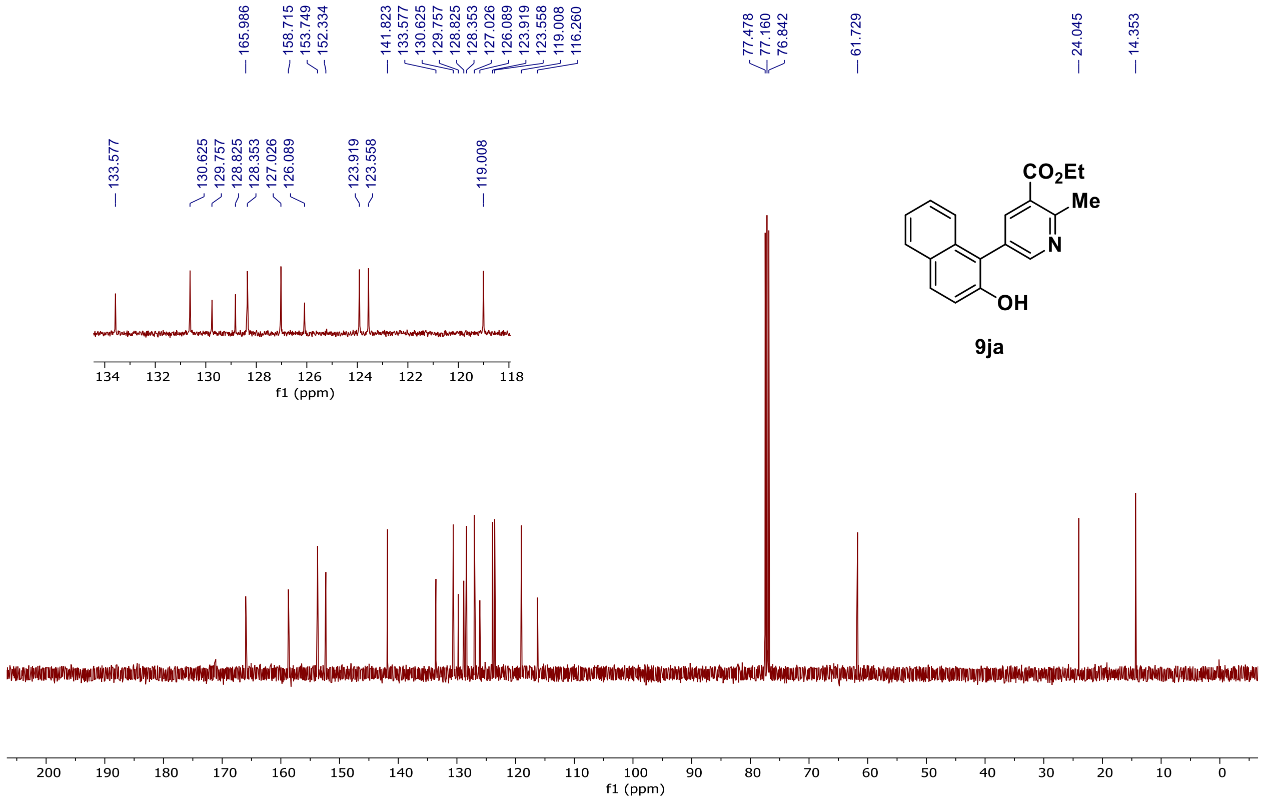

^{1}H and ^{13}C NMR Spectrum of **9ja** in $CDCl_3$

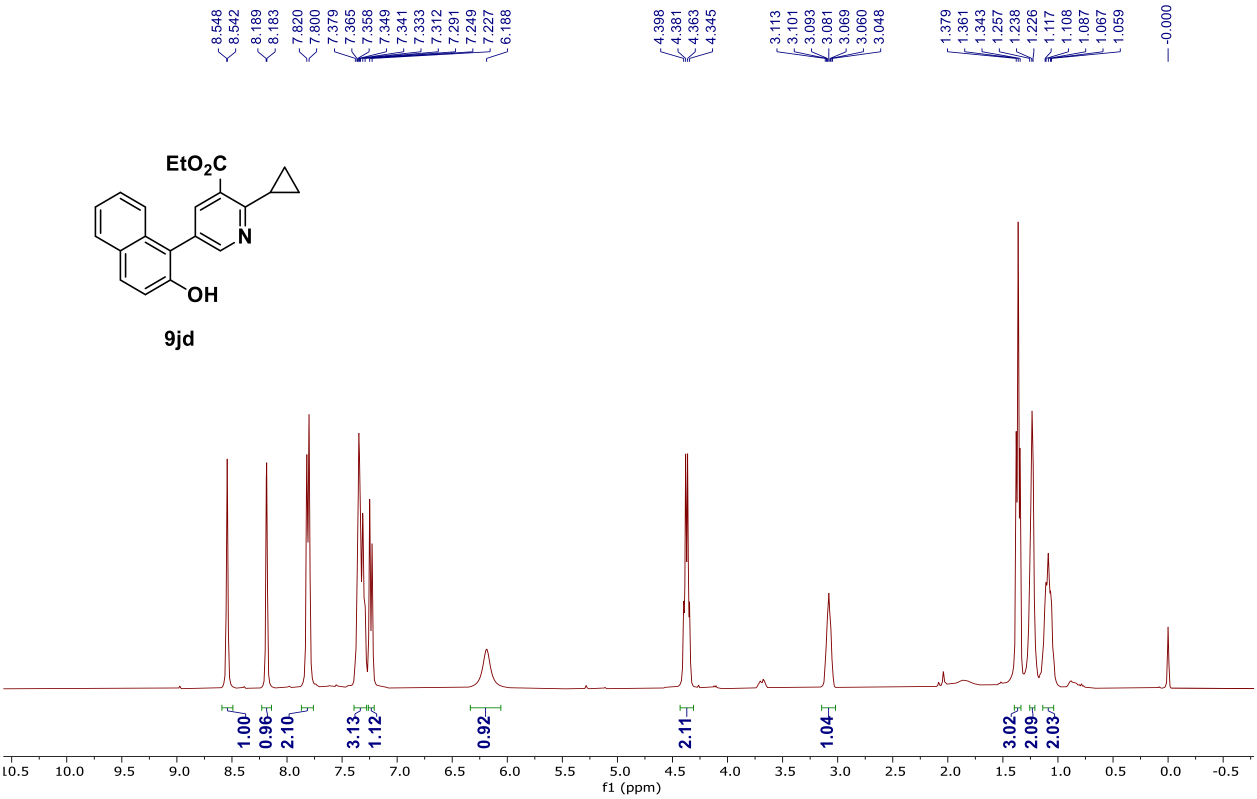

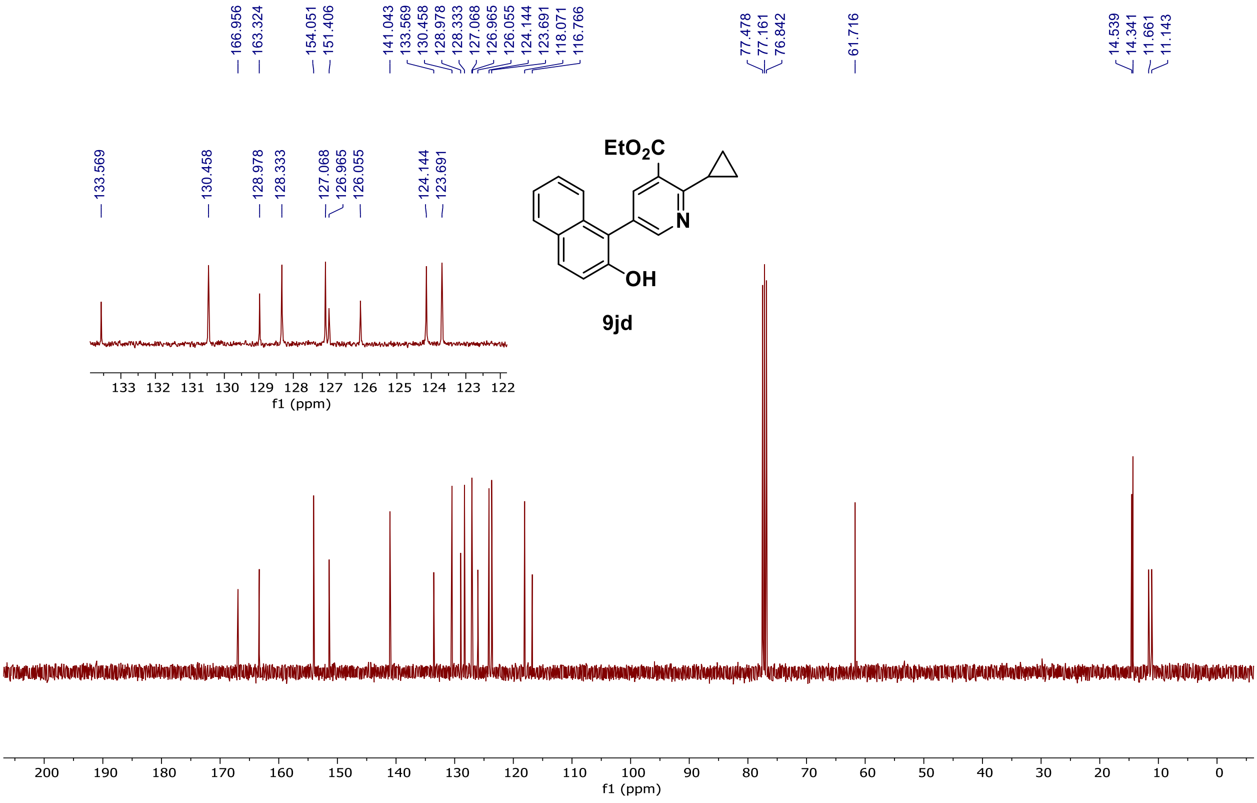

^{1}H and ^{13}C NMR Spectrum of **9jd** in $CDCl_3$

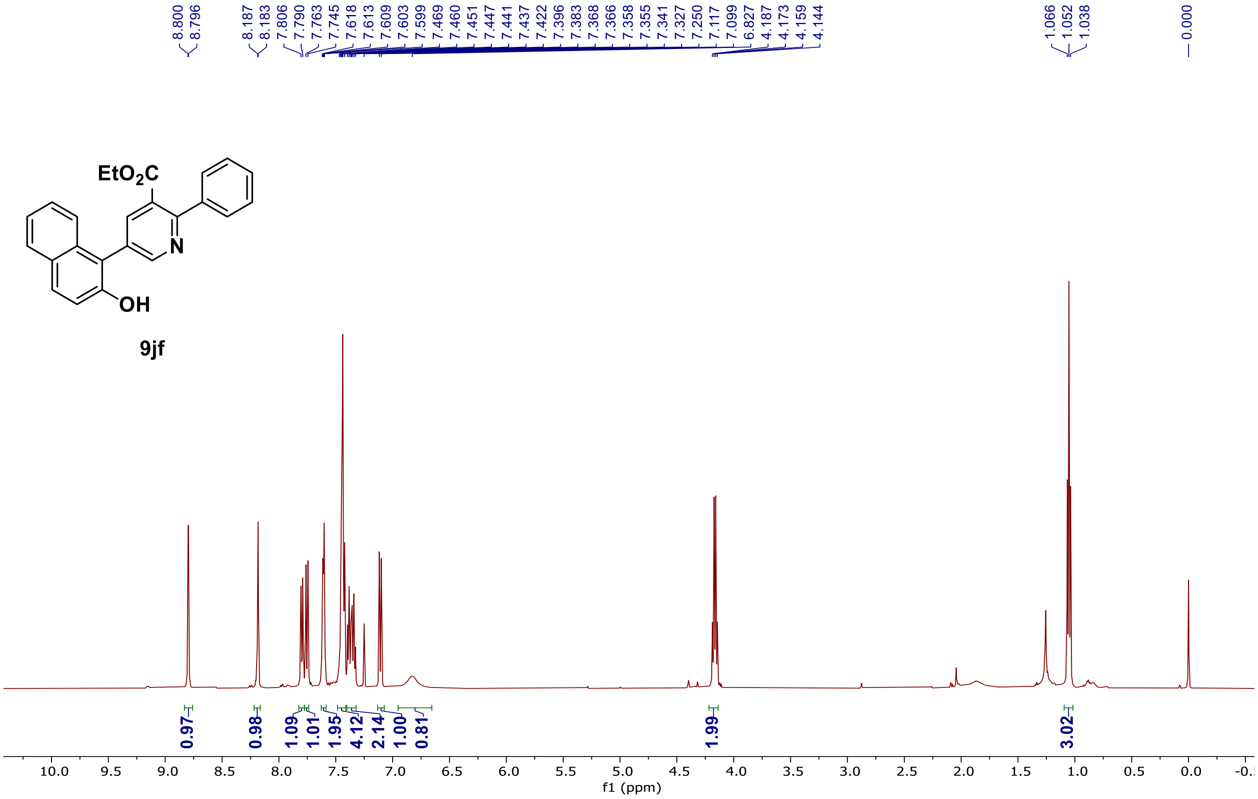

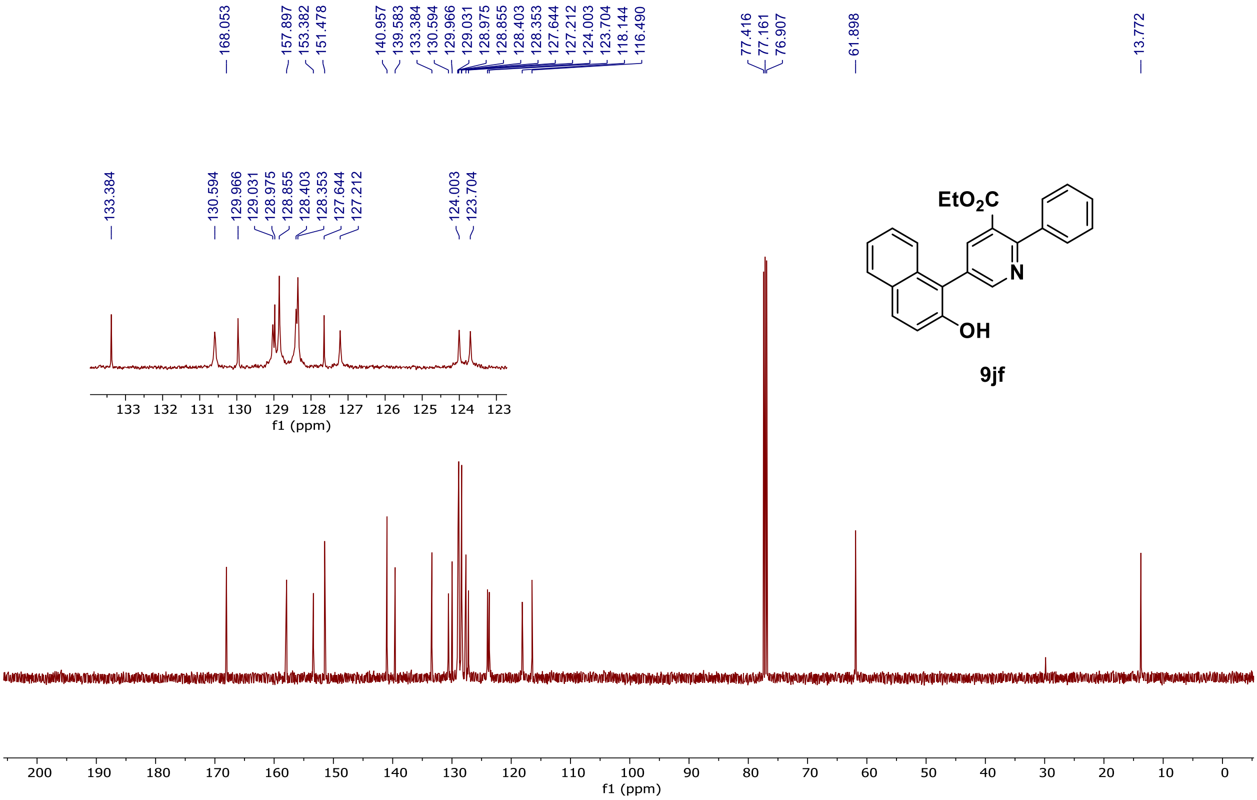

^{1}H and ^{13}C NMR Spectrum of **9jf** in $CDCl_3$

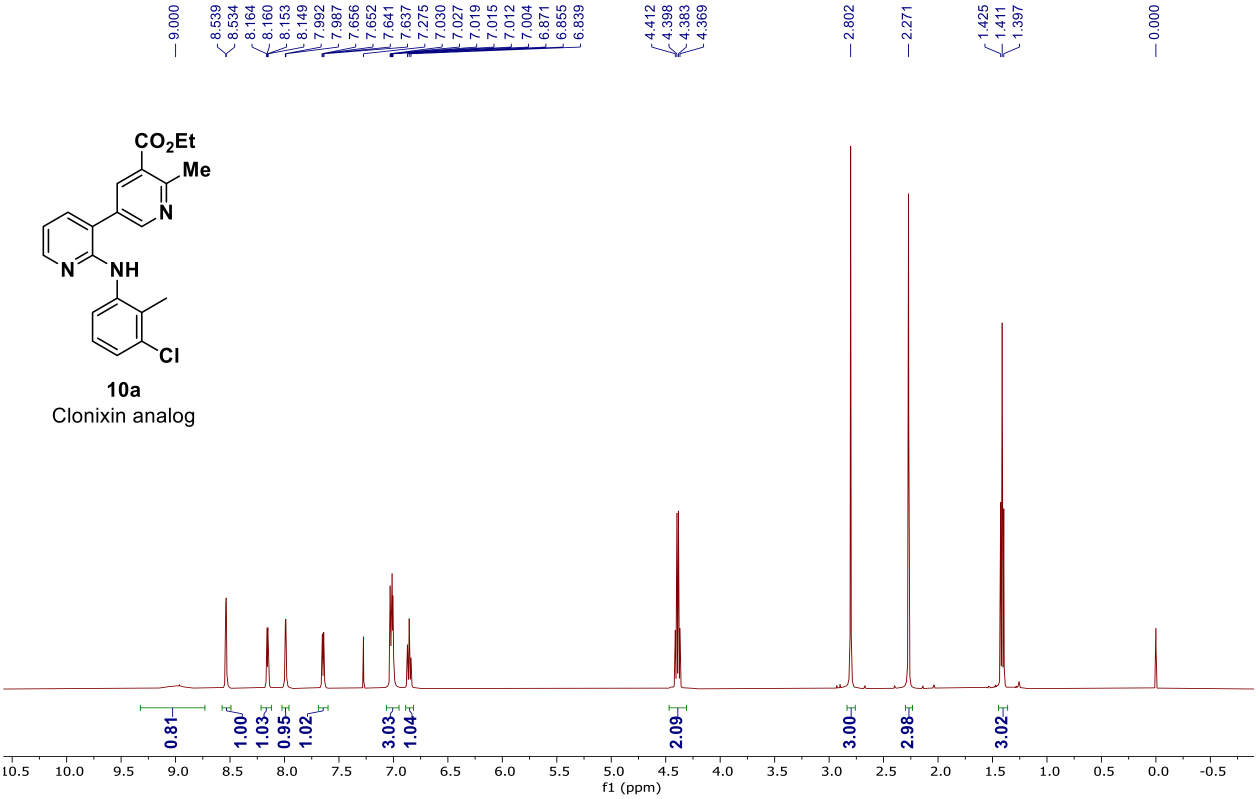

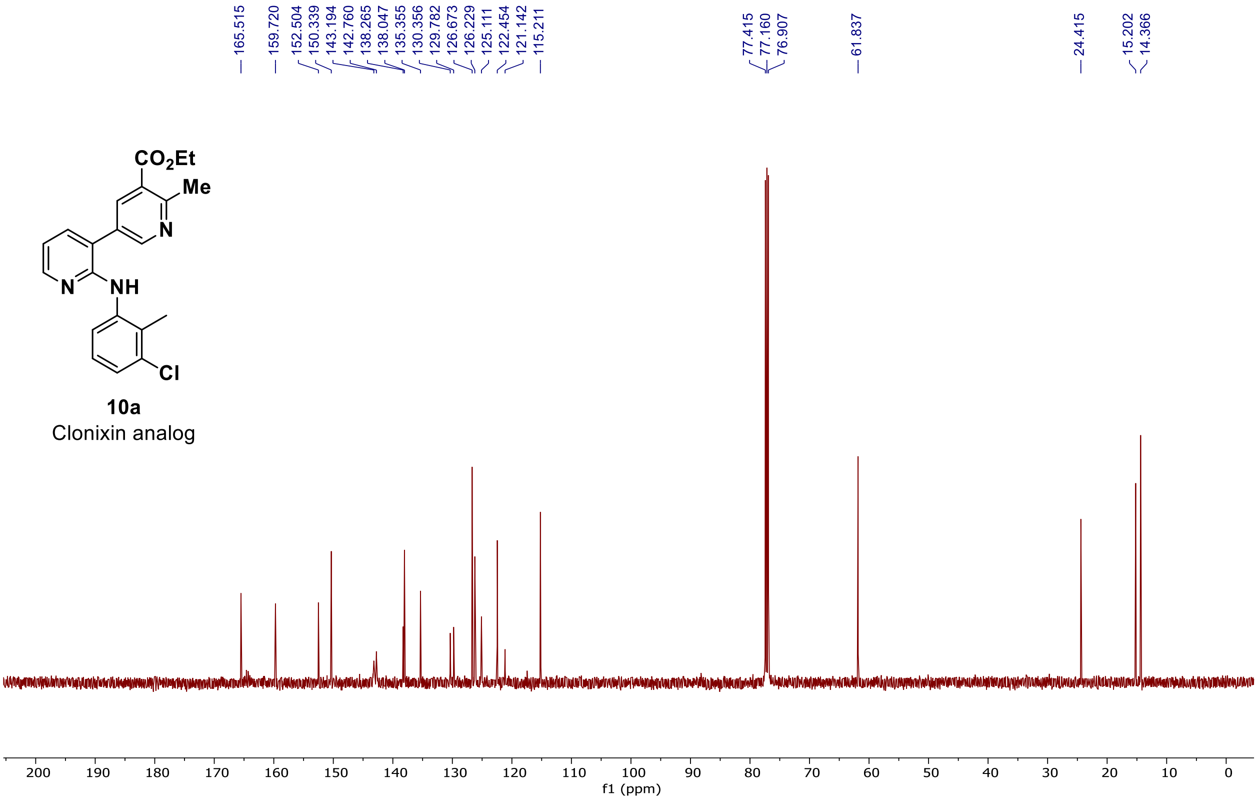

^{1}H and ^{13}C NMR Spectrum of **10a** in $CDCl_3$

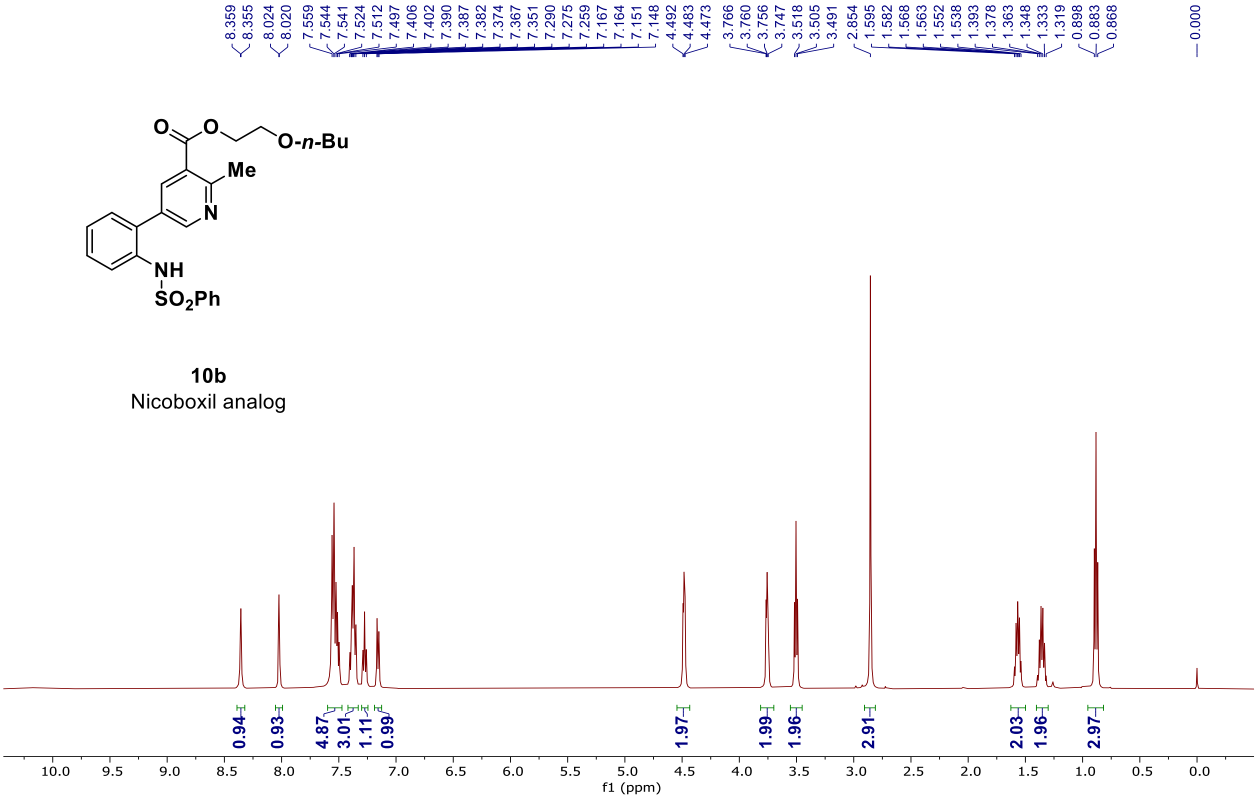

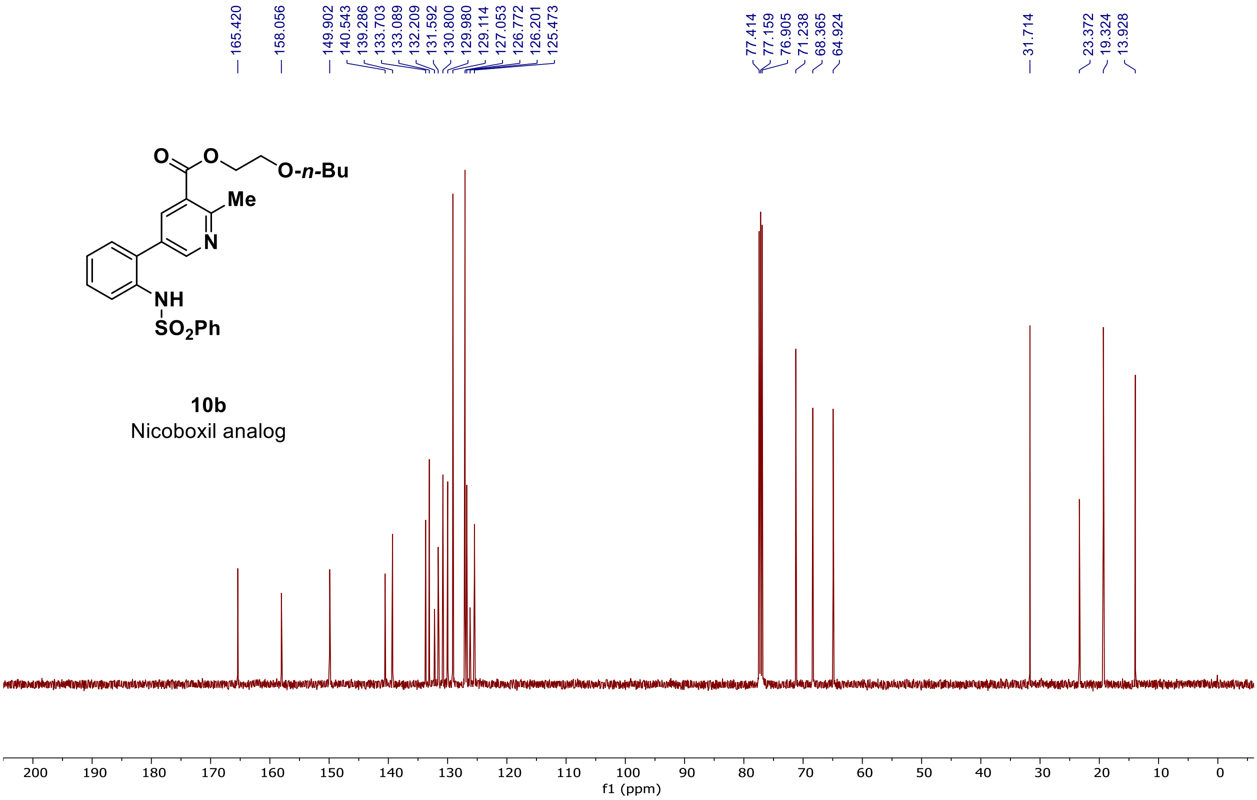

^{1}H and ^{13}C NMR Spectrum of **10b** in $CDCl_3$

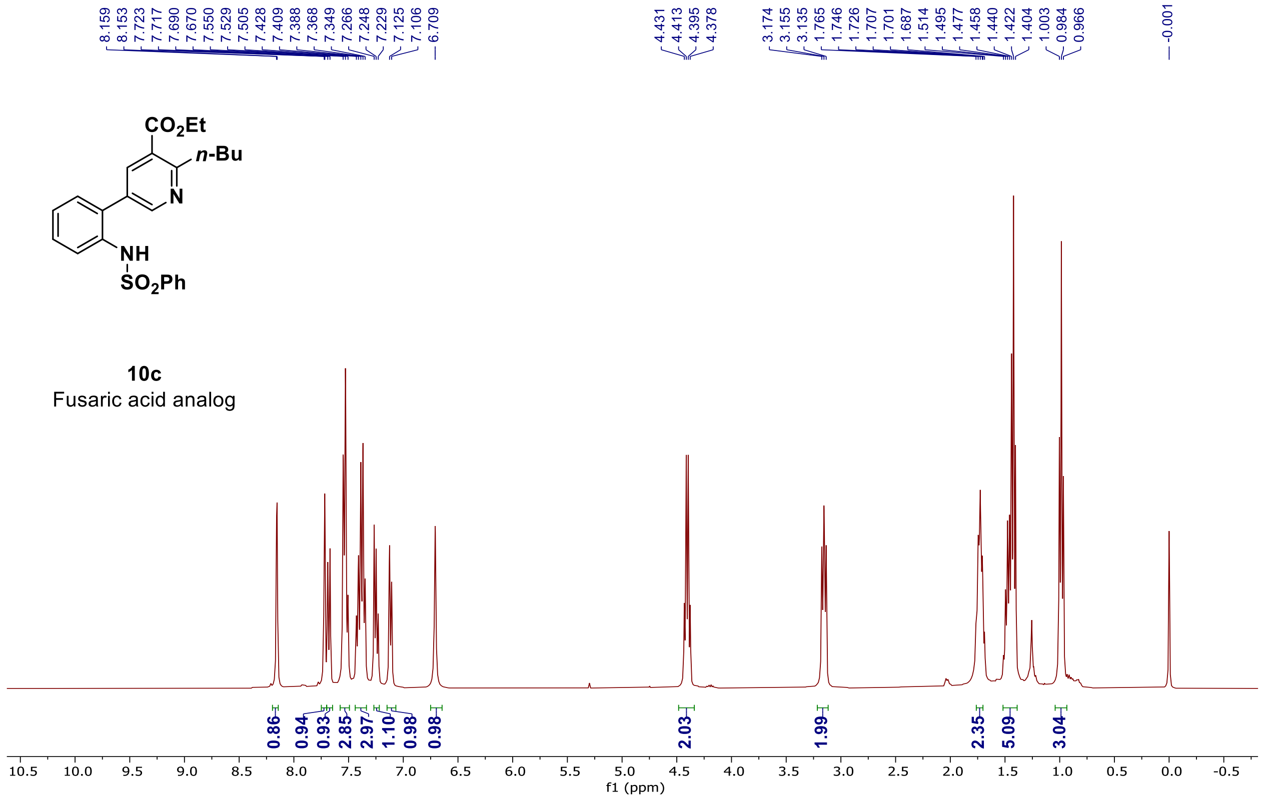

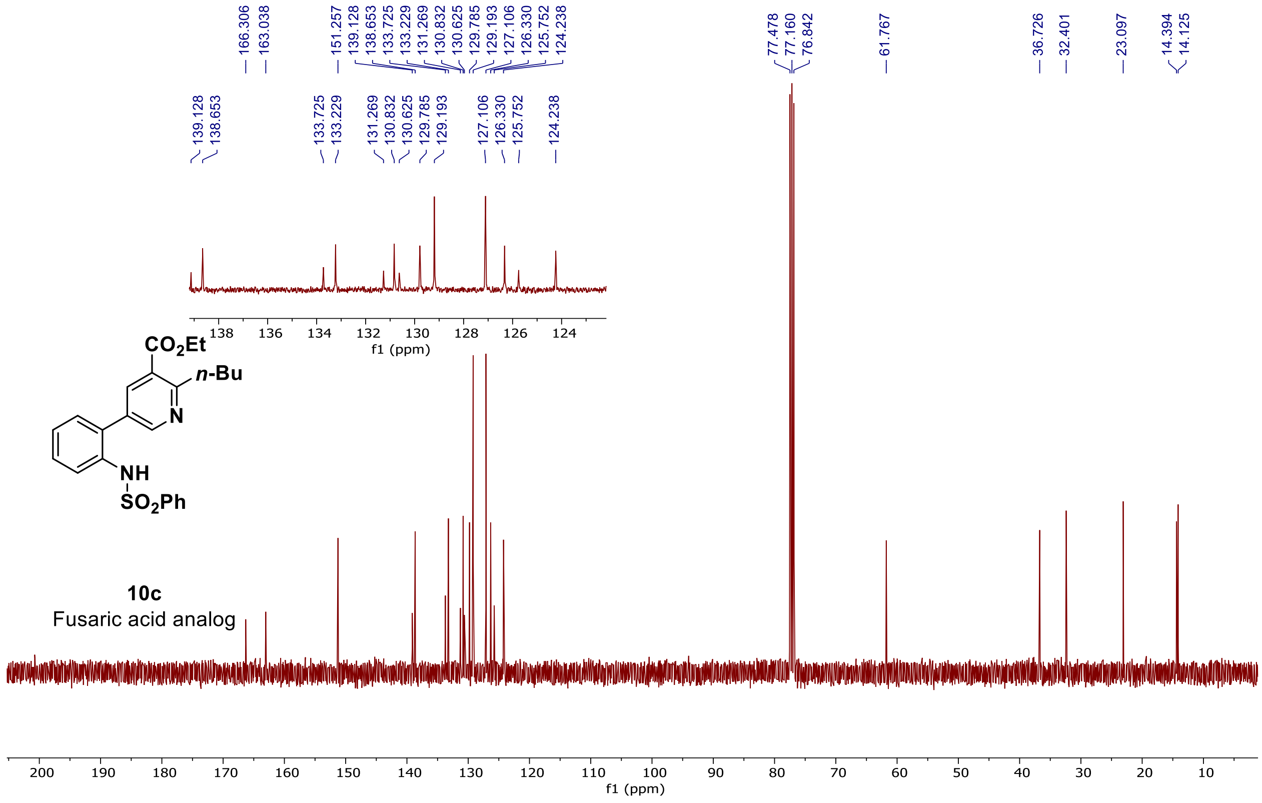

^{1}H and ^{13}C NMR Spectrum of **10c** in $CDCl_3$

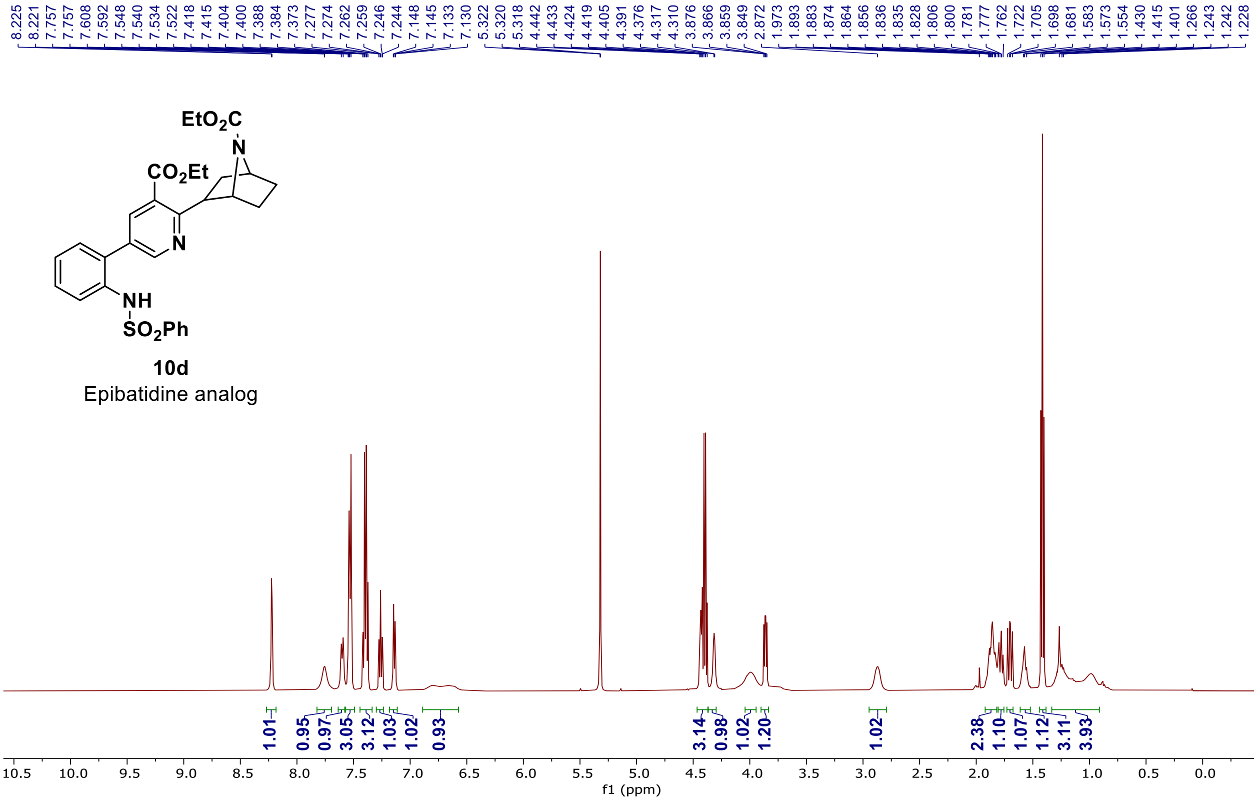

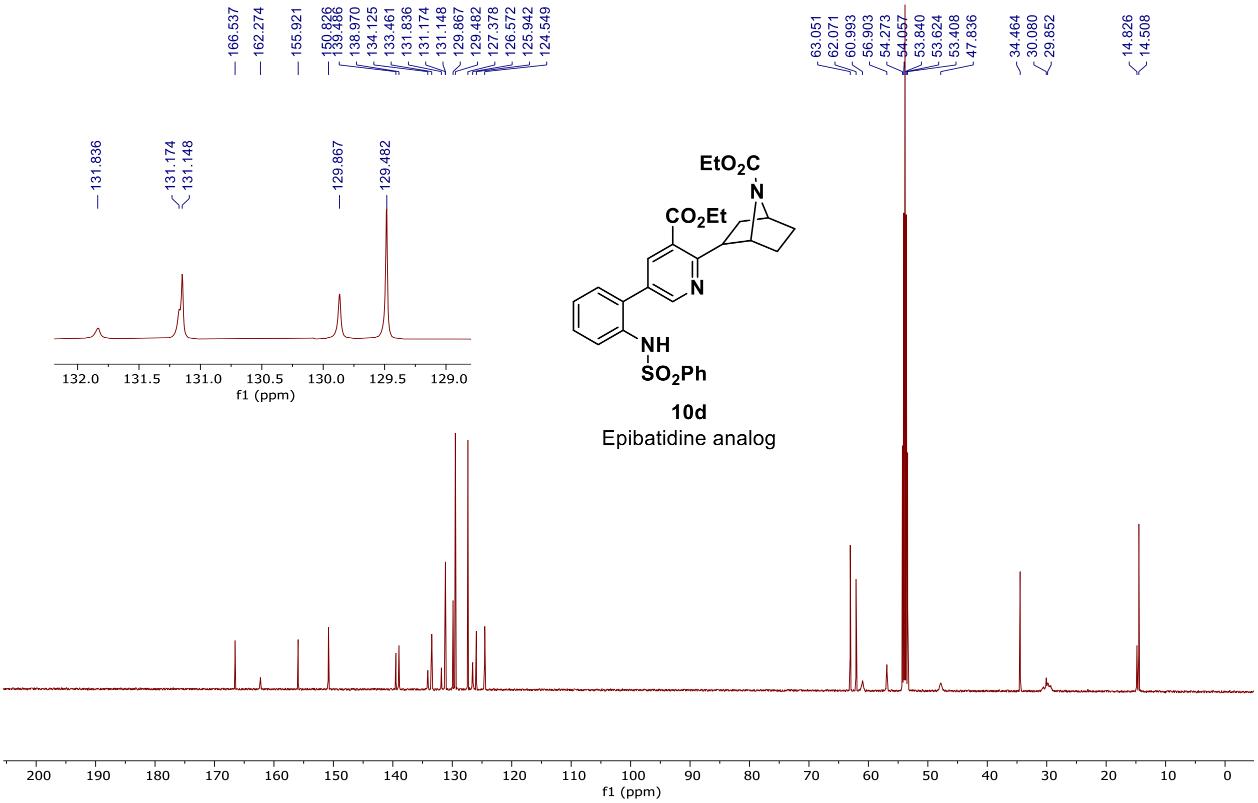

^{1}H and ^{13}C NMR Spectrum of **10d** in CD_2Cl_2

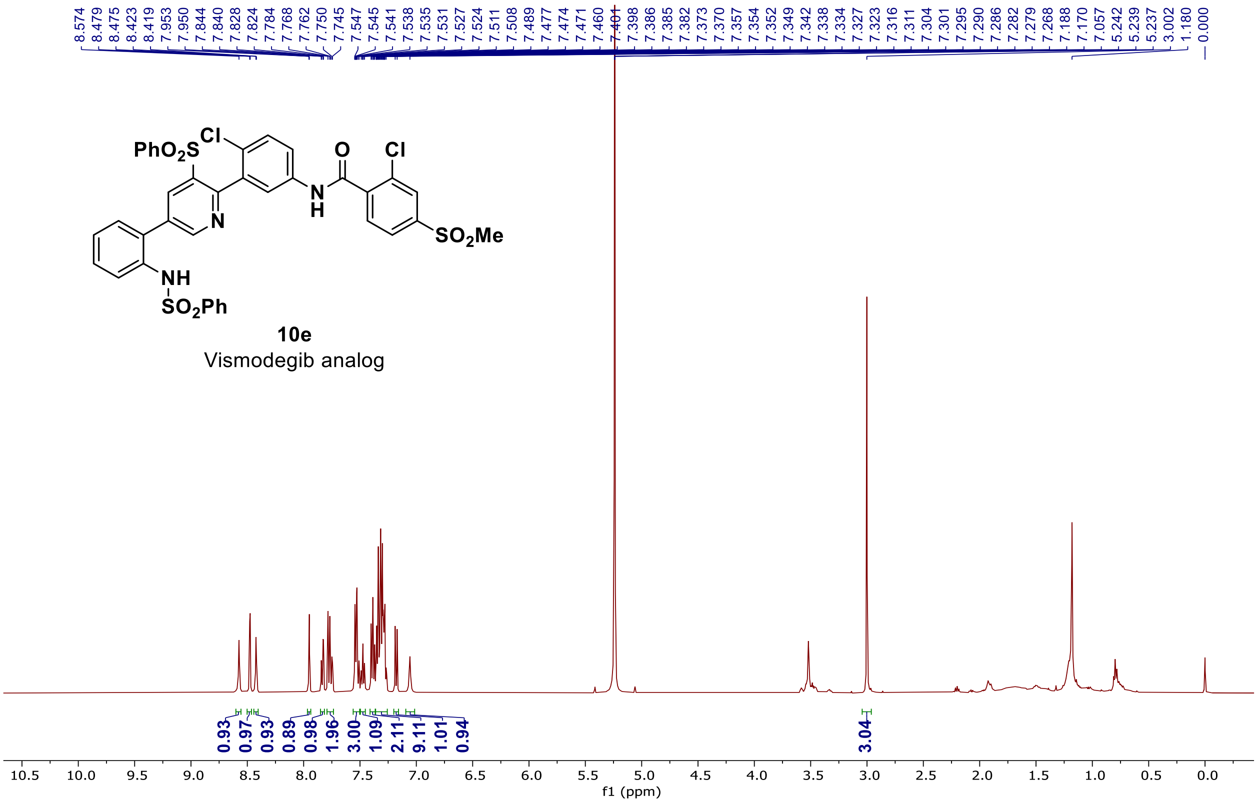

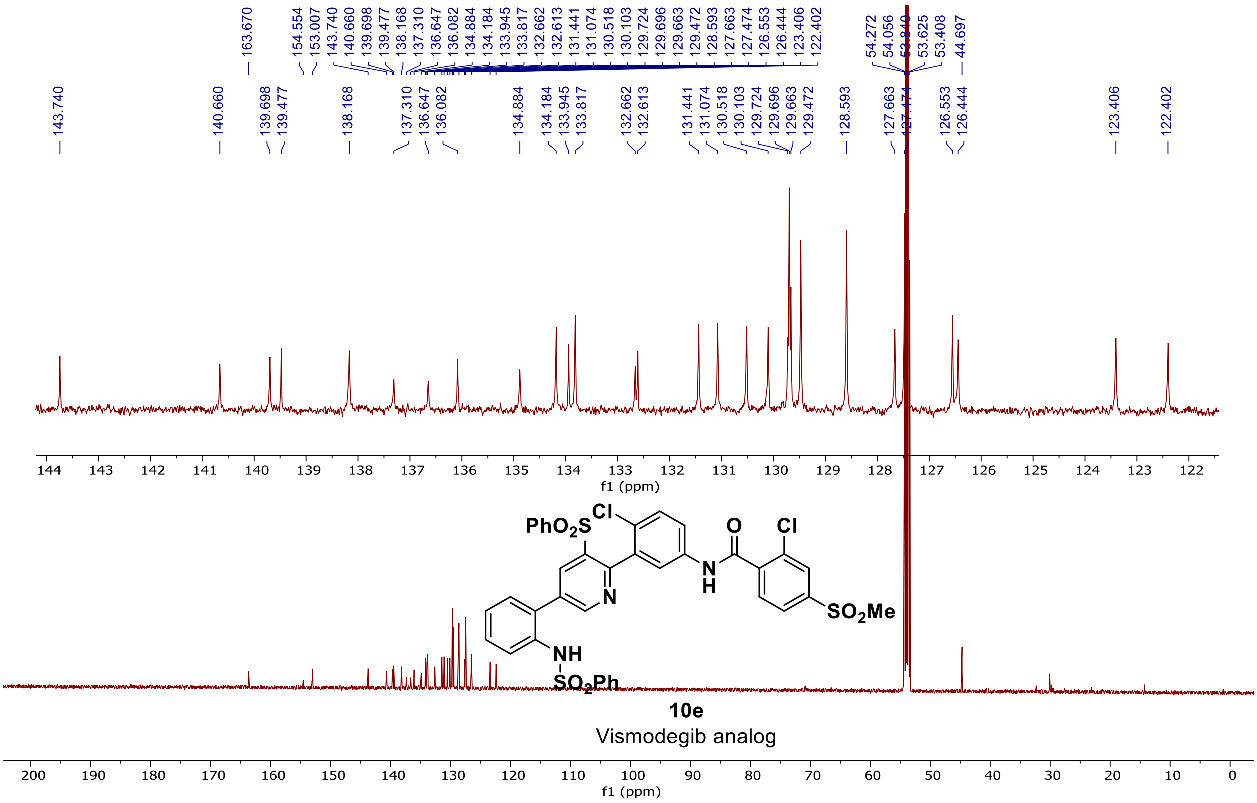

^{1}H and ^{13}C NMR Spectrum of **10e** in CD_2Cl_2

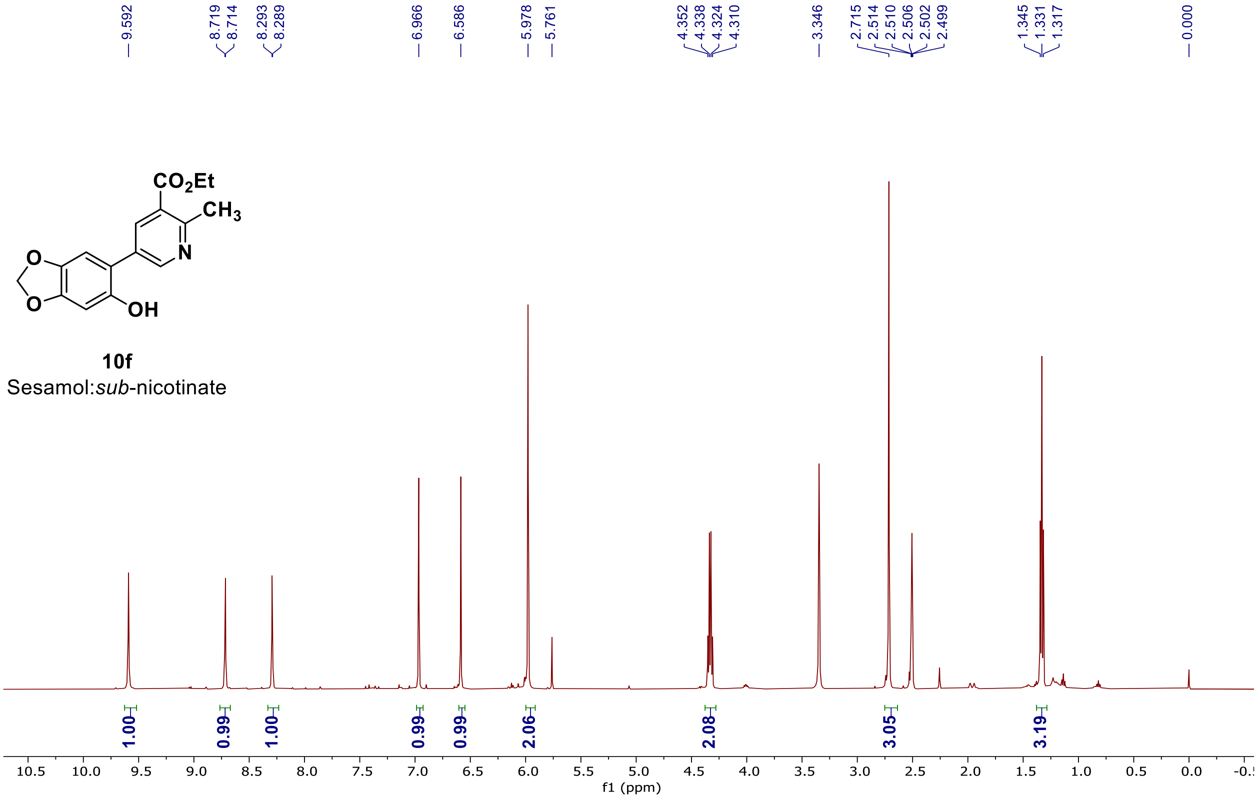

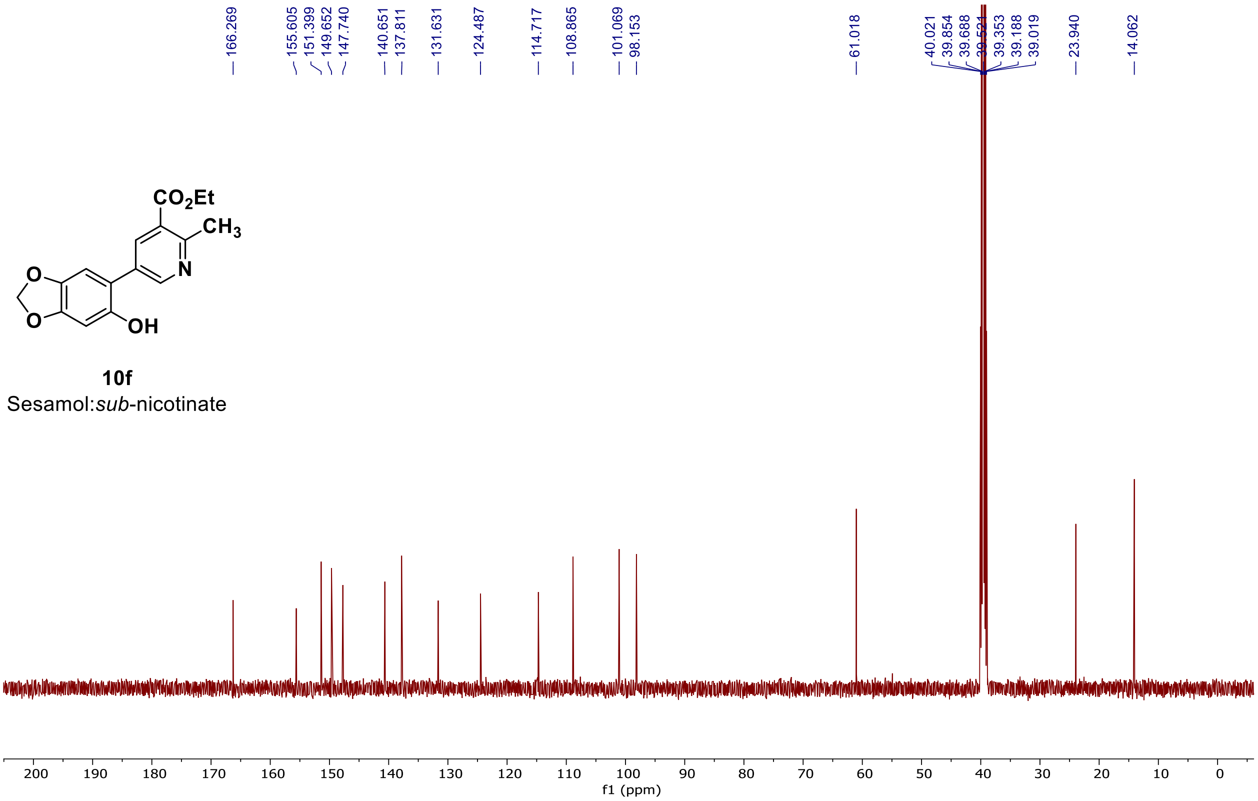

^{1}H and ^{13}C NMR Spectrum of **10f** in DMSO-d_6

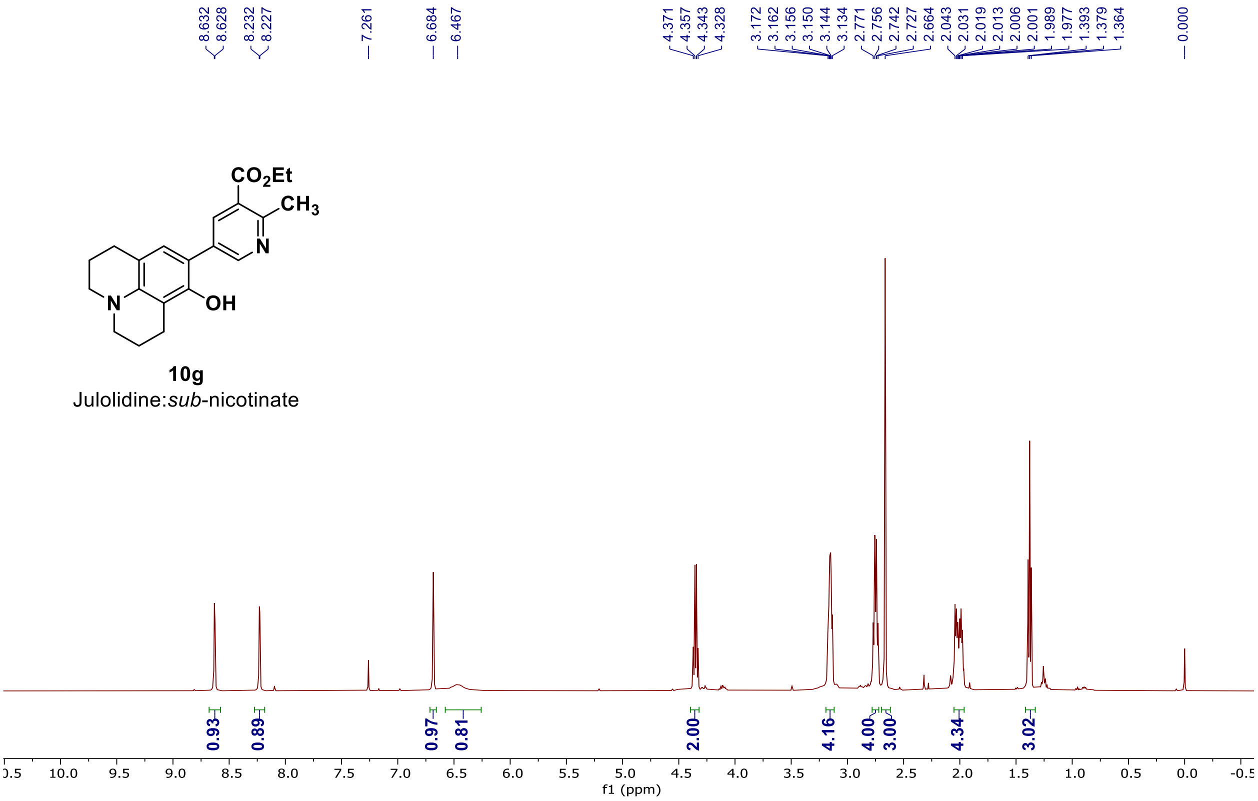

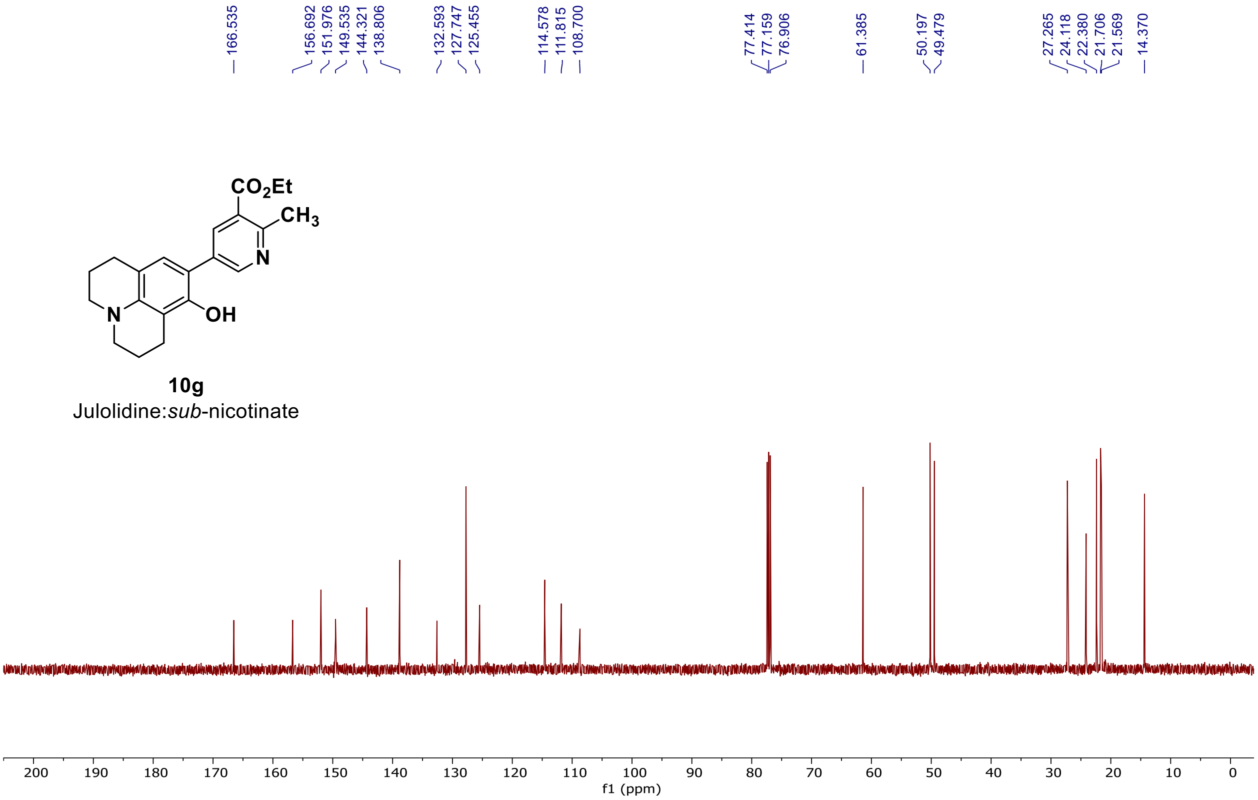

^{1}H and ^{13}C NMR Spectrum of **10g** in $CDCl_3$

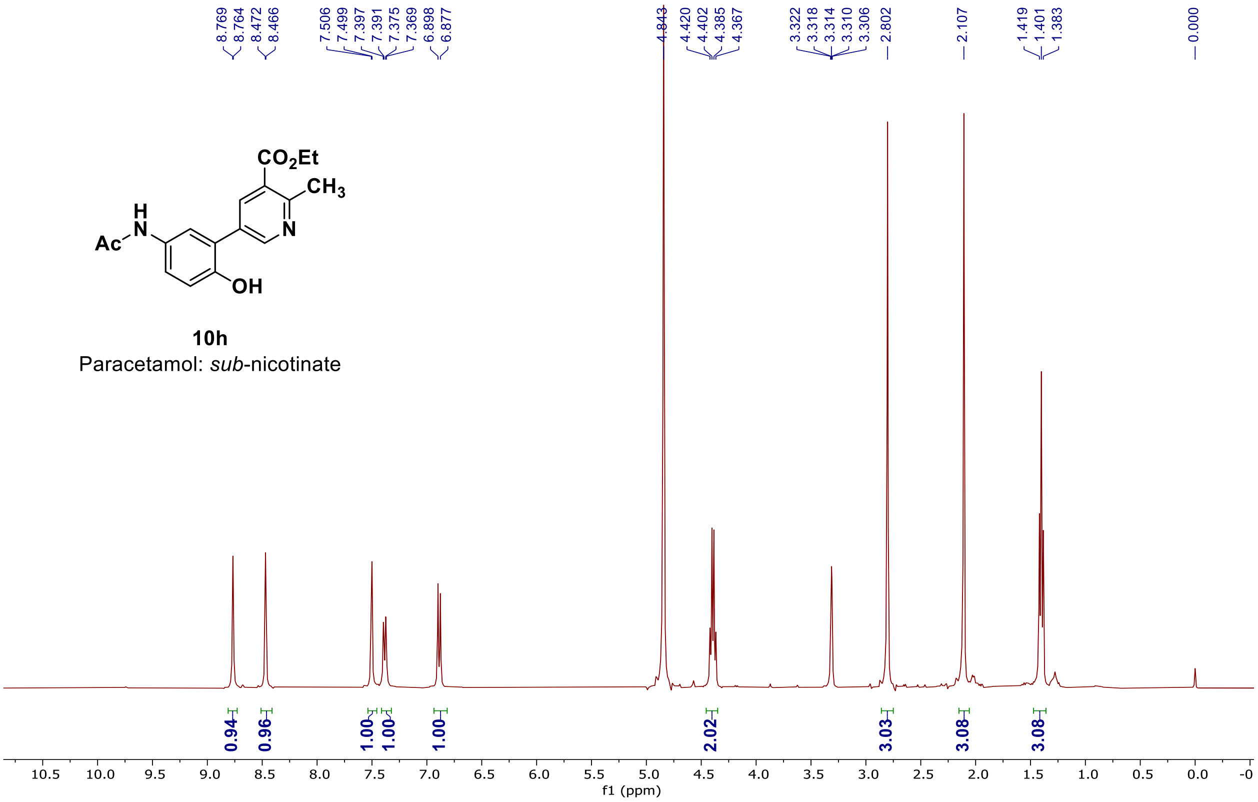

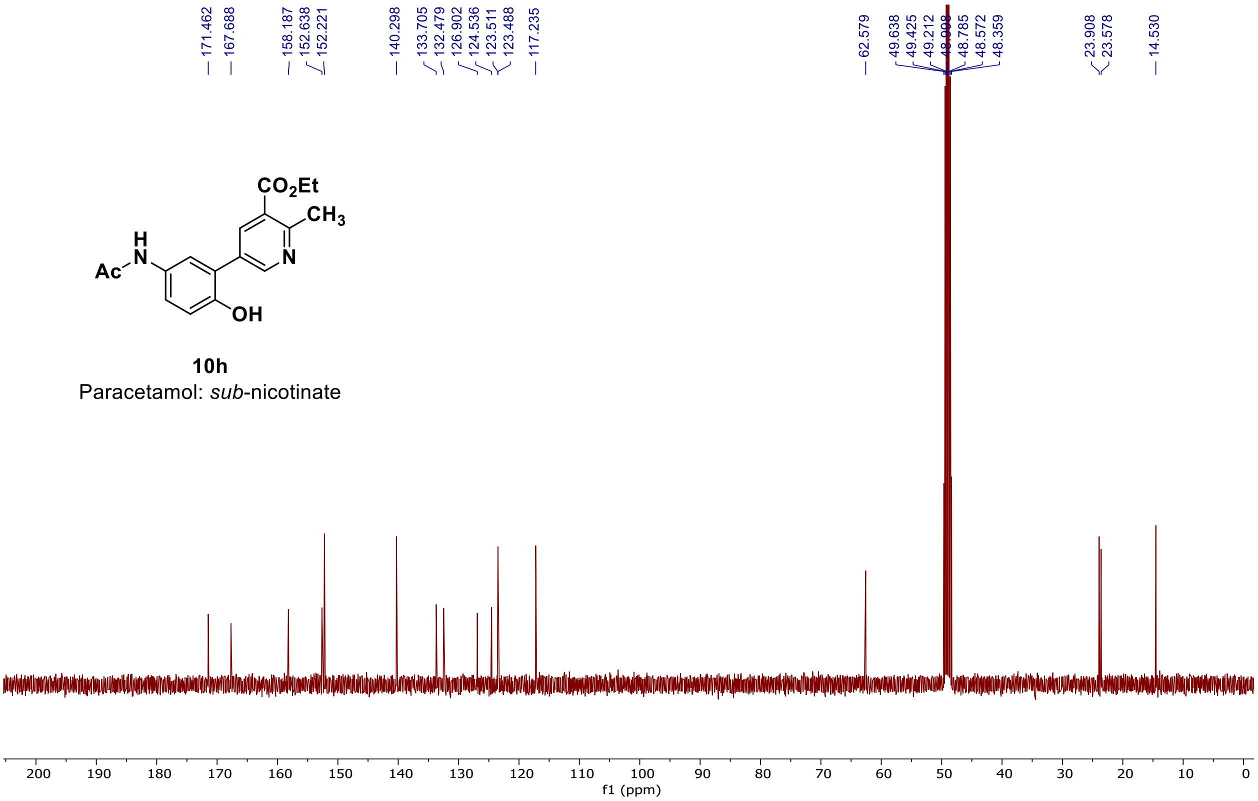

^{1}H and ^{13}C NMR Spectrum of **10h** in CD_3OD

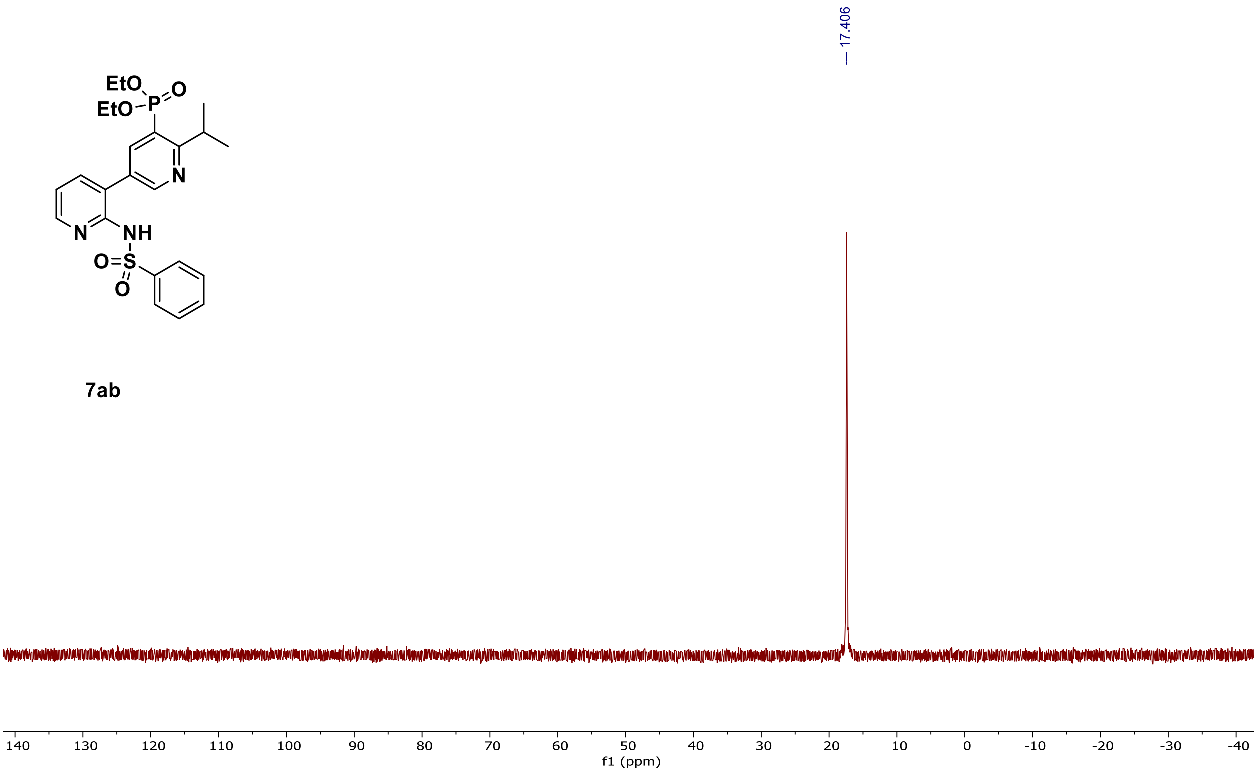

^{31}P NMR (162 MHz) Spectrum of **7ab** in $CDCl_3$

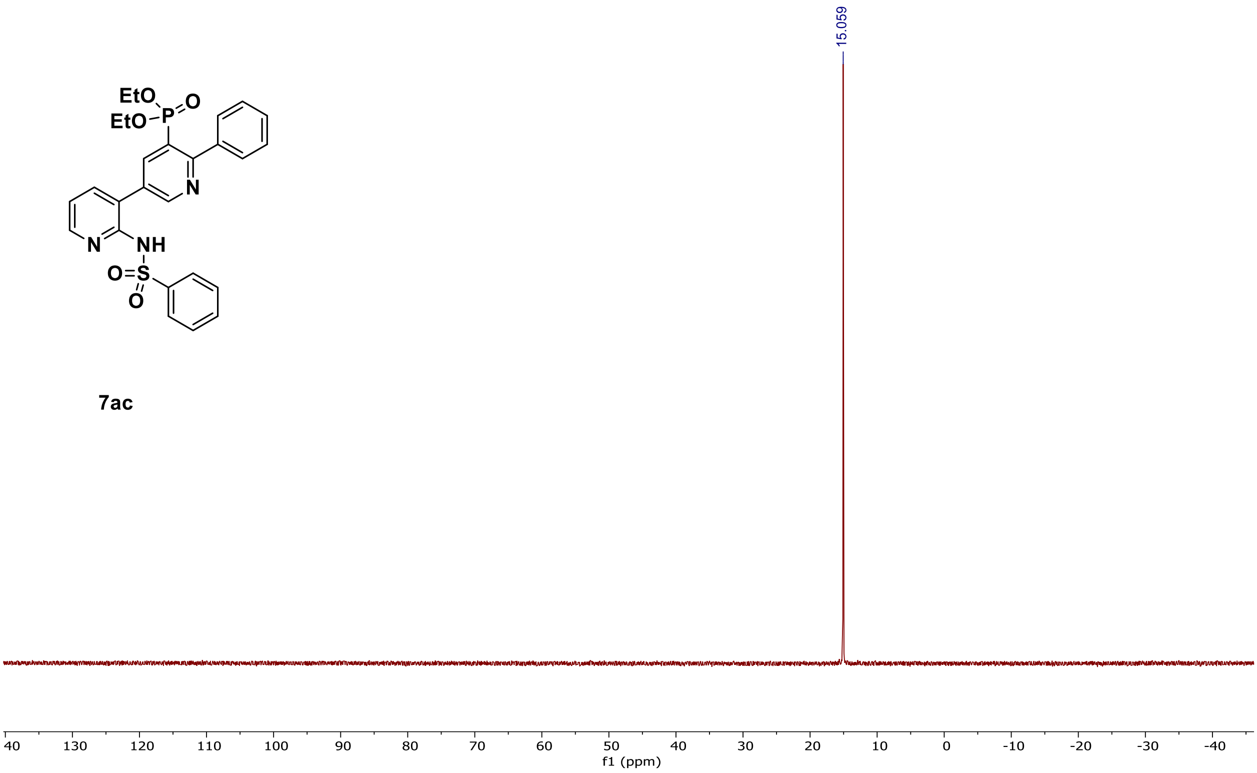

^{31}P NMR (162 MHz) Spectrum of **7ac** in $CDCl_3$

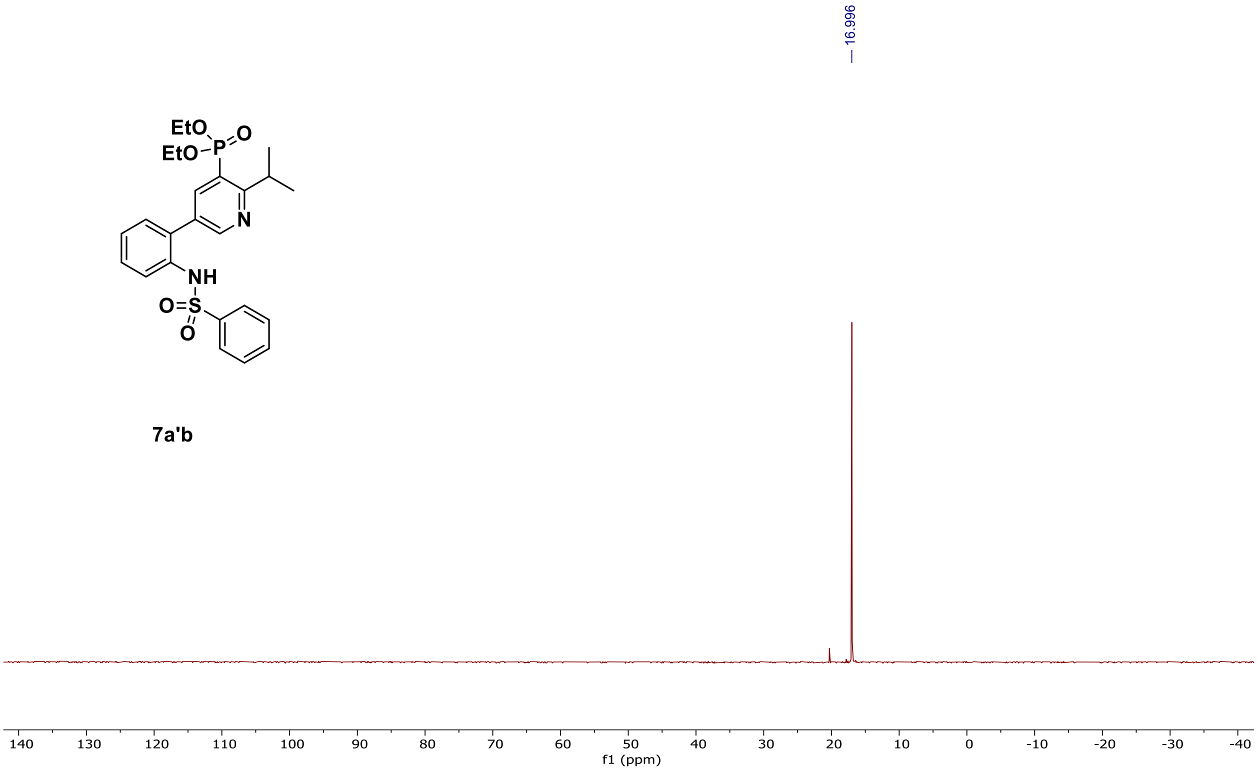

^{31}P NMR (162 MHz) Spectrum of **7a'b** in $CDCl_3$

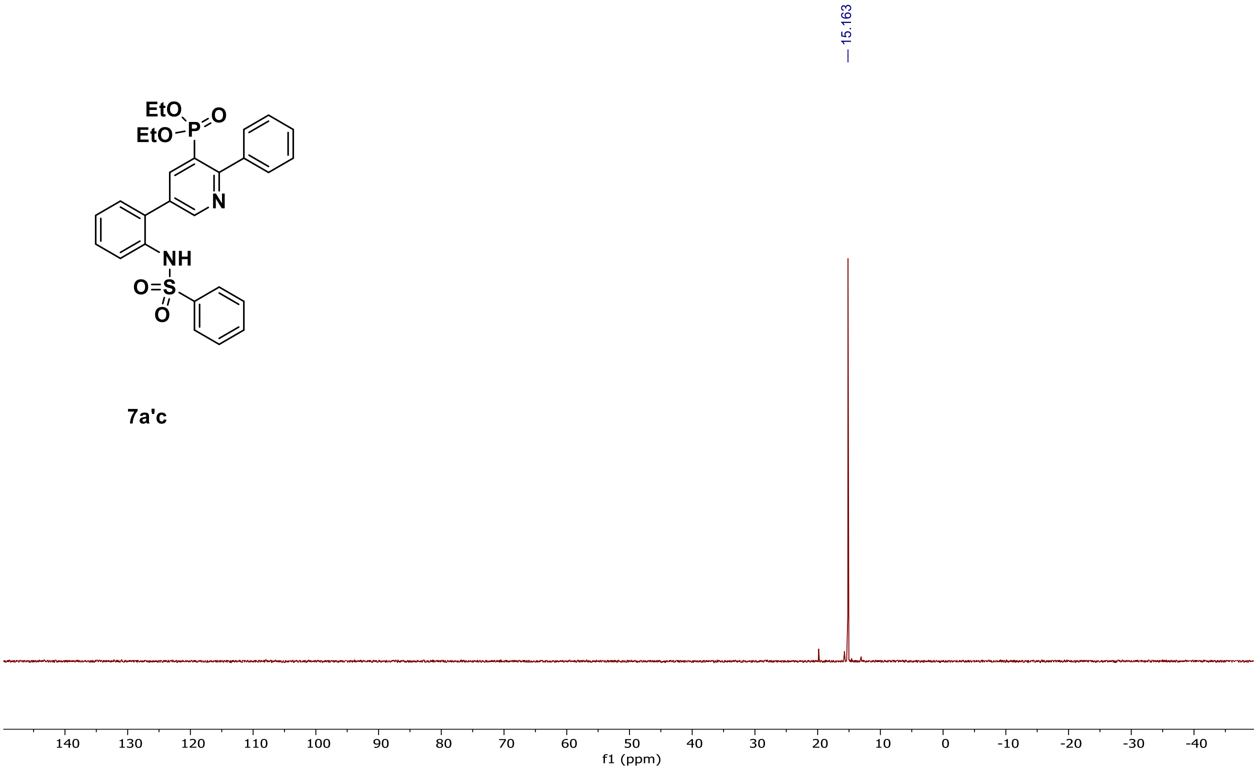

^{31}P NMR (162 MHz) Spectrum of **7a'c** in $CDCl_3$

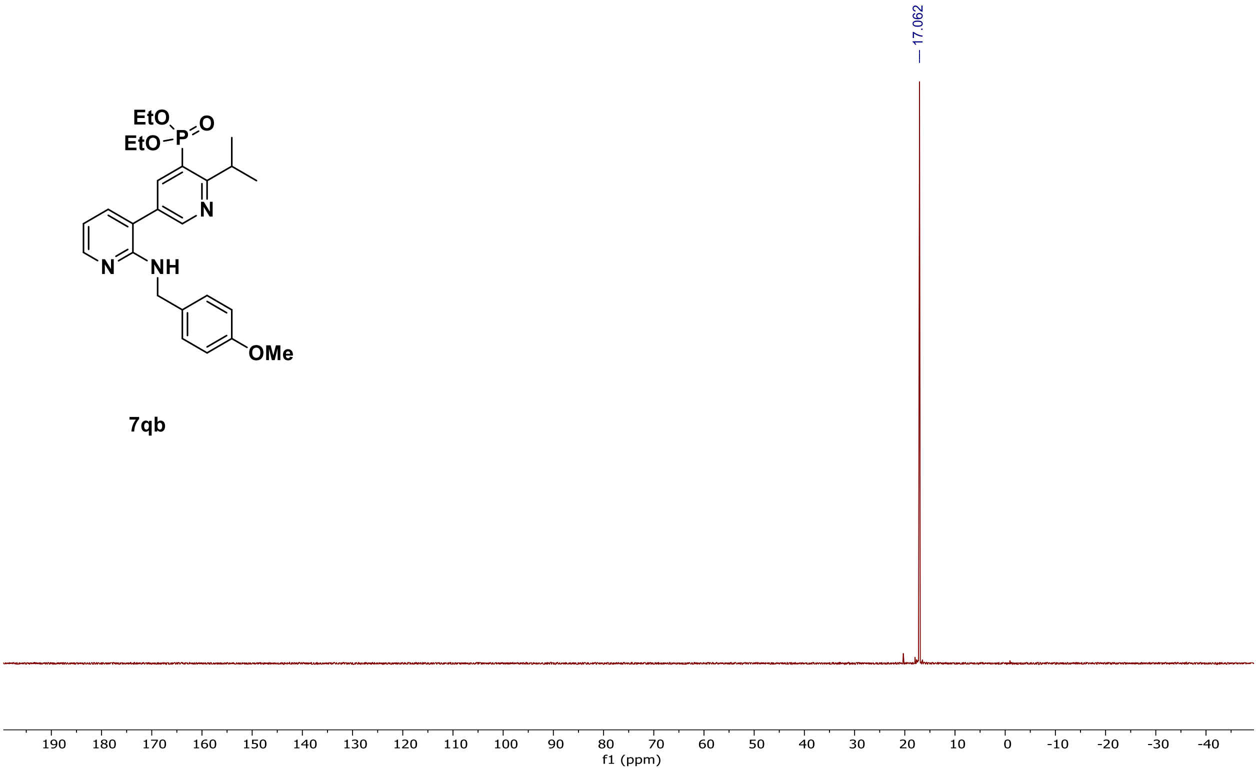

^{31}P NMR (162 MHz) Spectrum of **7qb** in $CDCl_3$

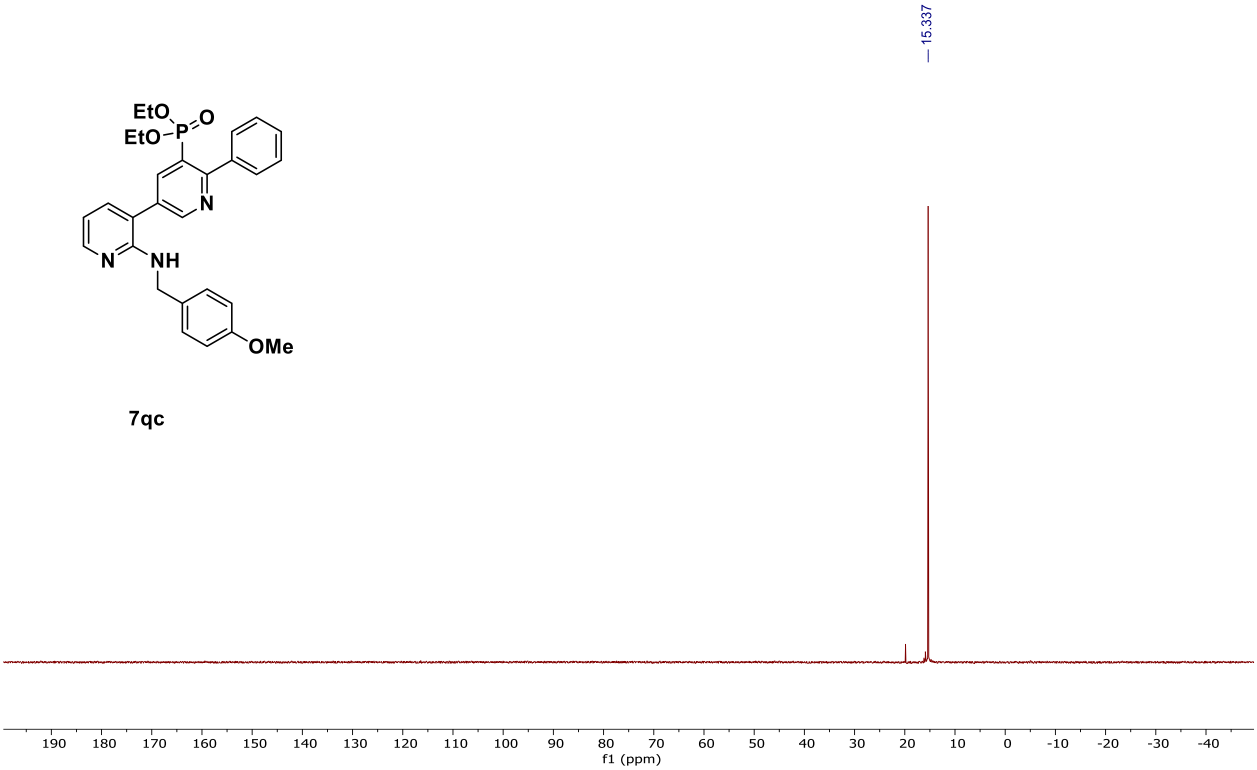

^{31}P NMR (162 MHz) Spectrum of **7qc** in $CDCl_3$

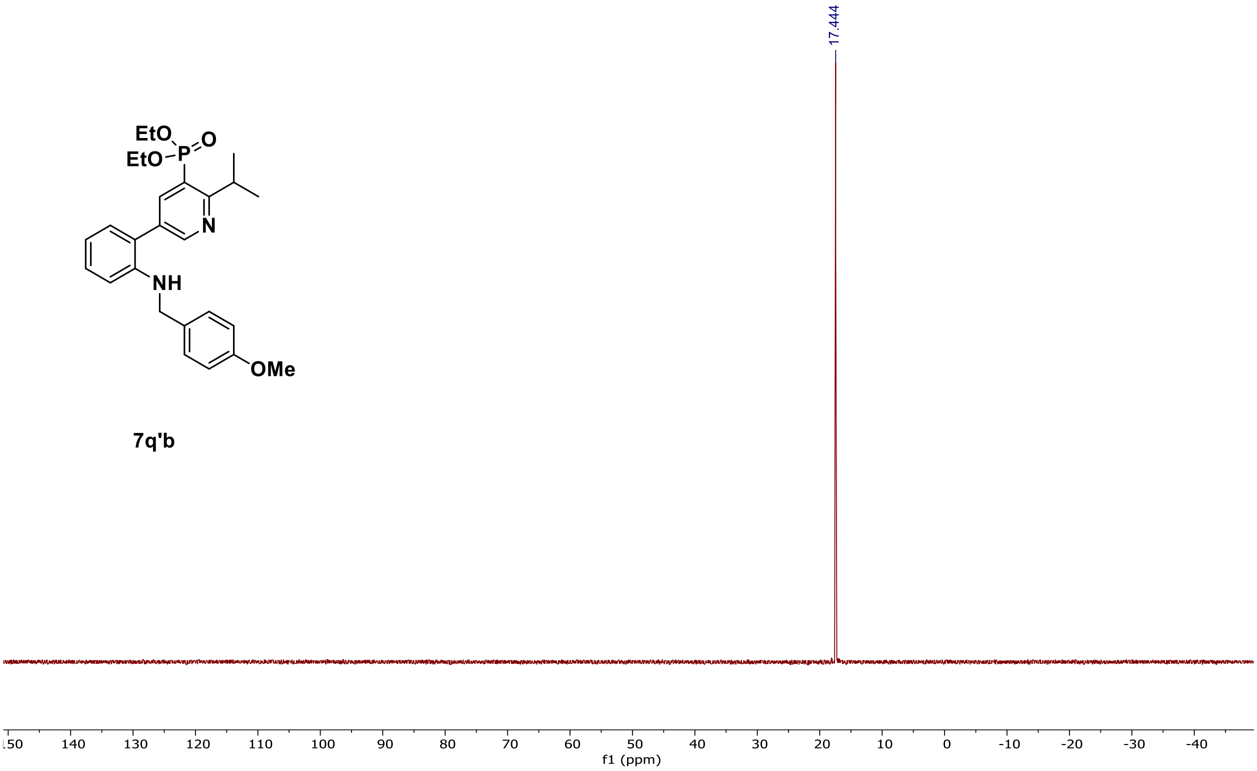

^{31}P NMR (162 MHz) Spectrum of **7q'b** in $CDCl_3$

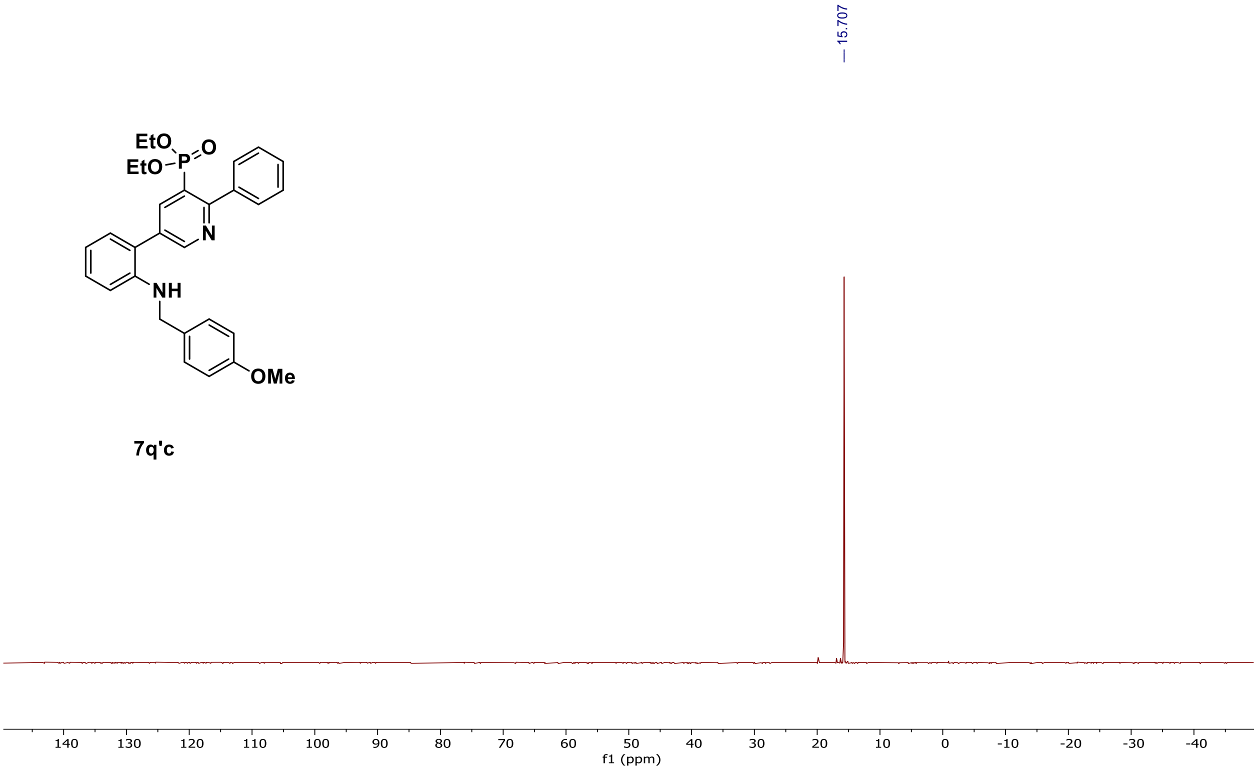

^{31}P NMR (162 MHz) Spectrum of **7q'c** in $CDCl_3$

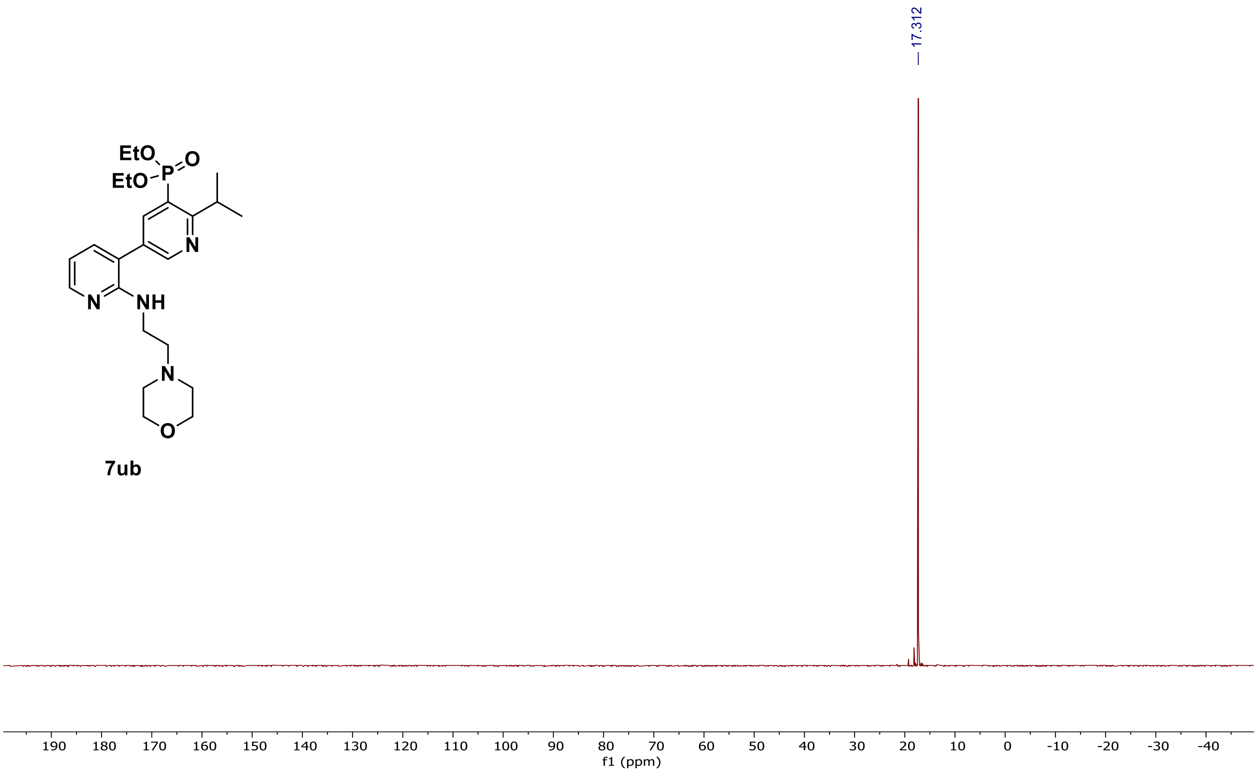

^{31}P NMR (162 MHz) Spectrum of **7ub** in $CDCl_3$

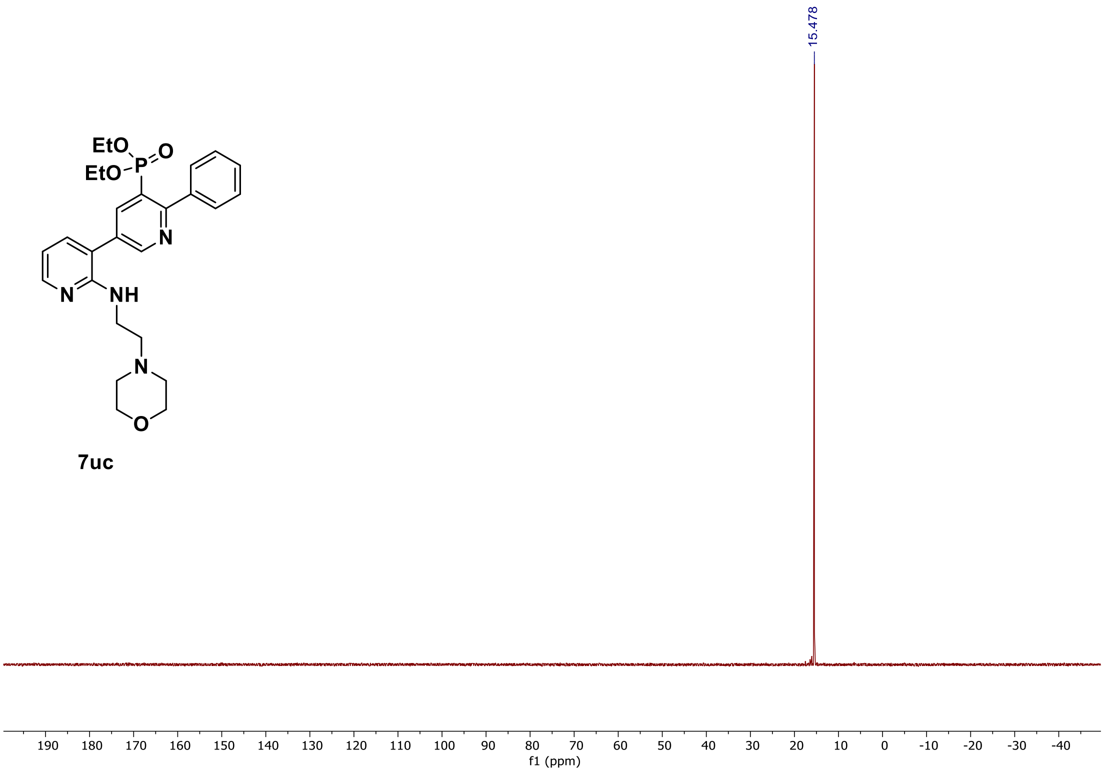

^{31}P NMR (162 MHz) Spectrum of **7uc** in $CDCl_3$

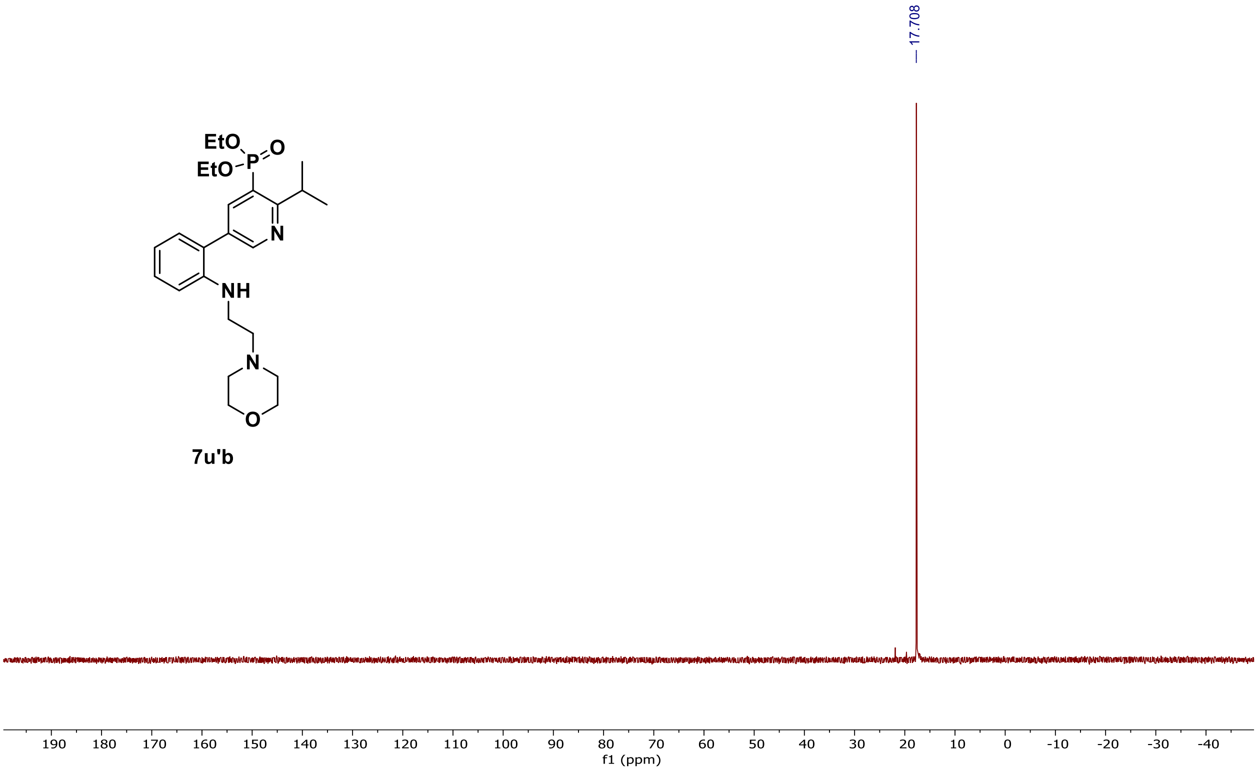

^{31}P NMR (162 MHz) Spectrum of **7u'b** in $CDCl_3$

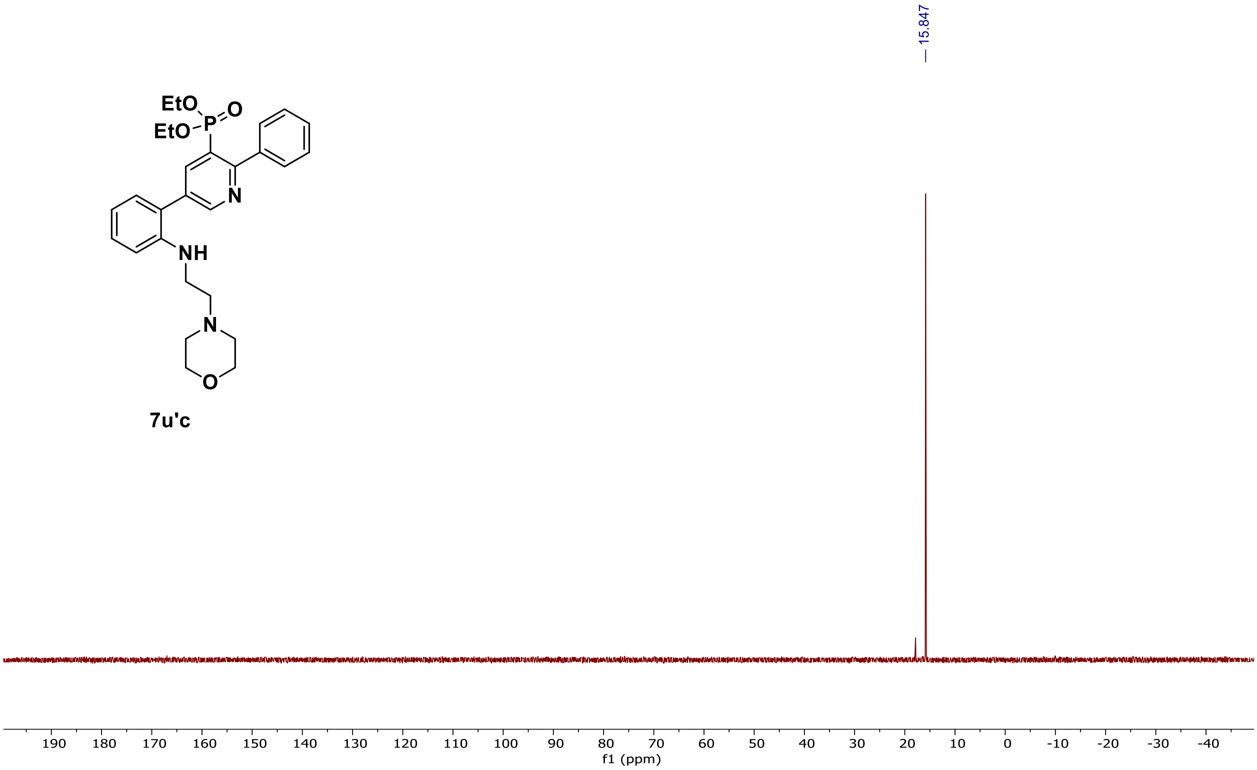

^{31}P NMR (162 MHz) Spectrum of **7u'c** in $CDCl_3$

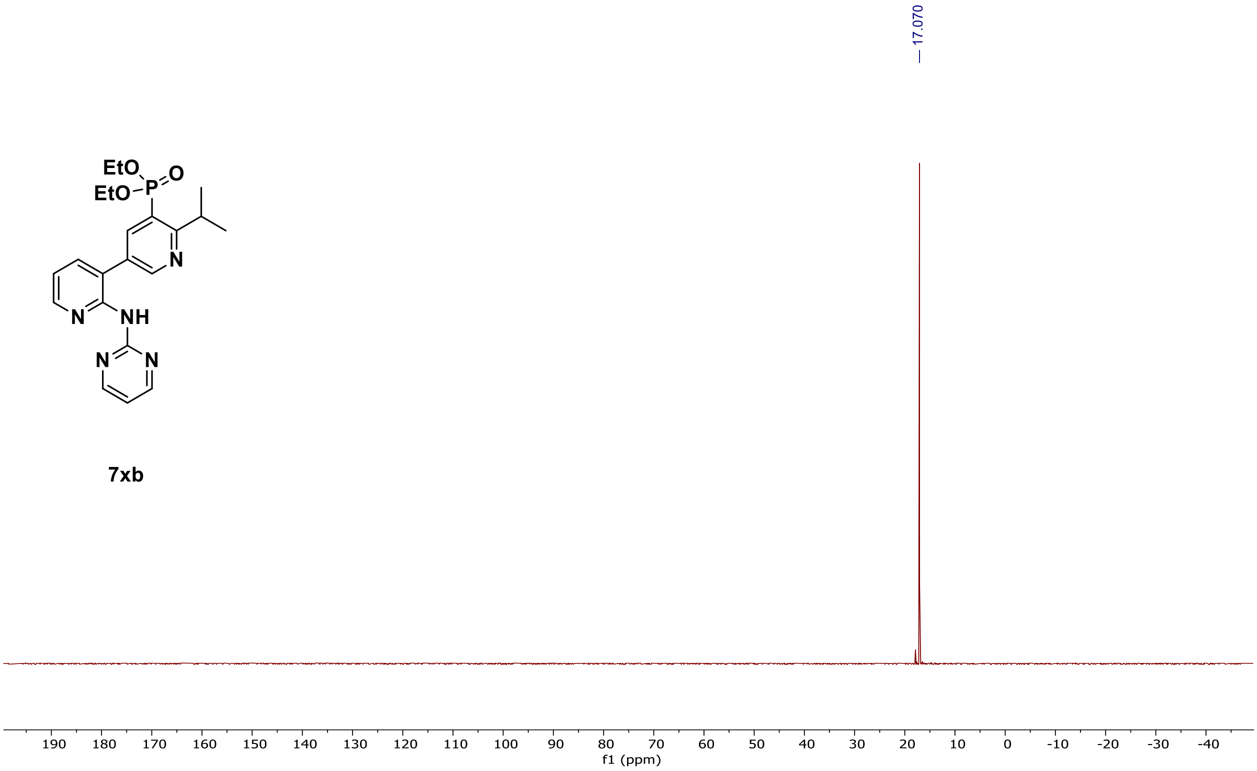

^{31}P NMR (162 MHz) Spectrum of **7xb** in $CDCl_3$

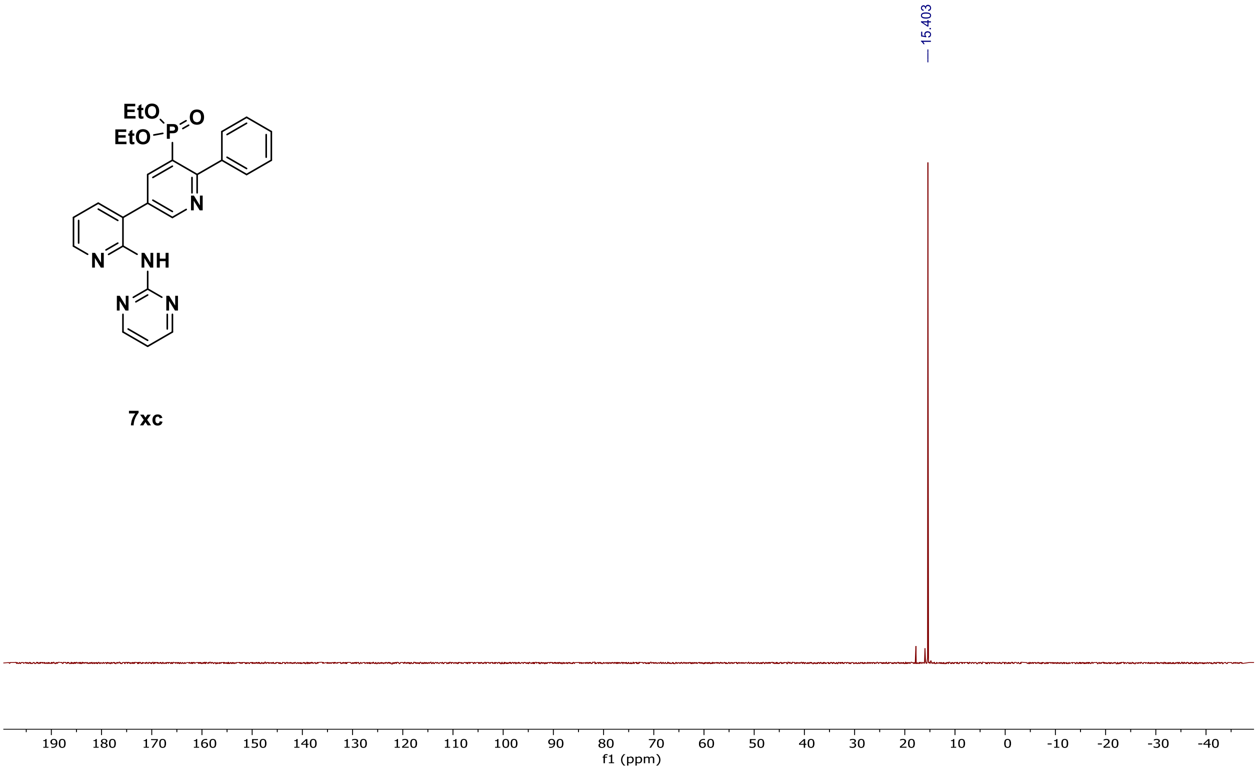

^{31}P NMR (162 MHz) Spectrum of **7xc** in $CDCl_3$

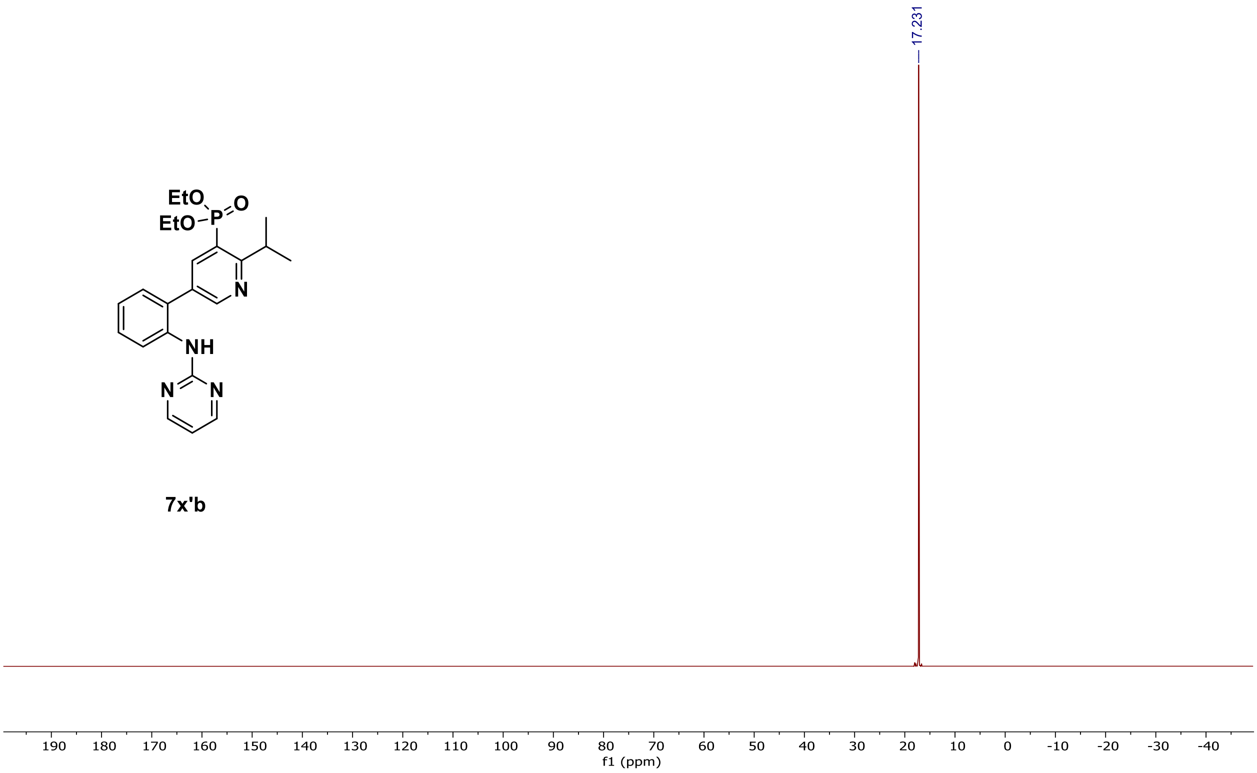

^{31}P NMR (162 MHz) Spectrum of **7x'b** in $CDCl_3$

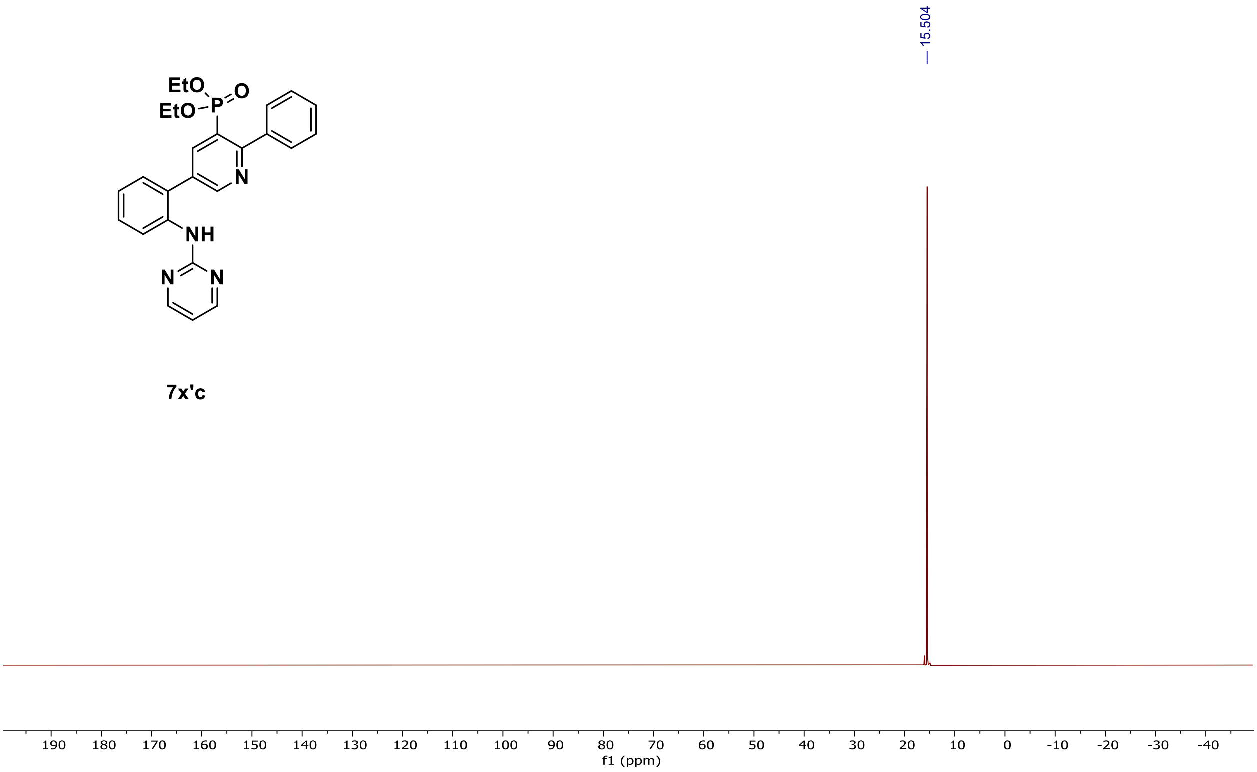

^{31}P NMR (162 MHz) Spectrum of **7x'c** in $CDCl_3$

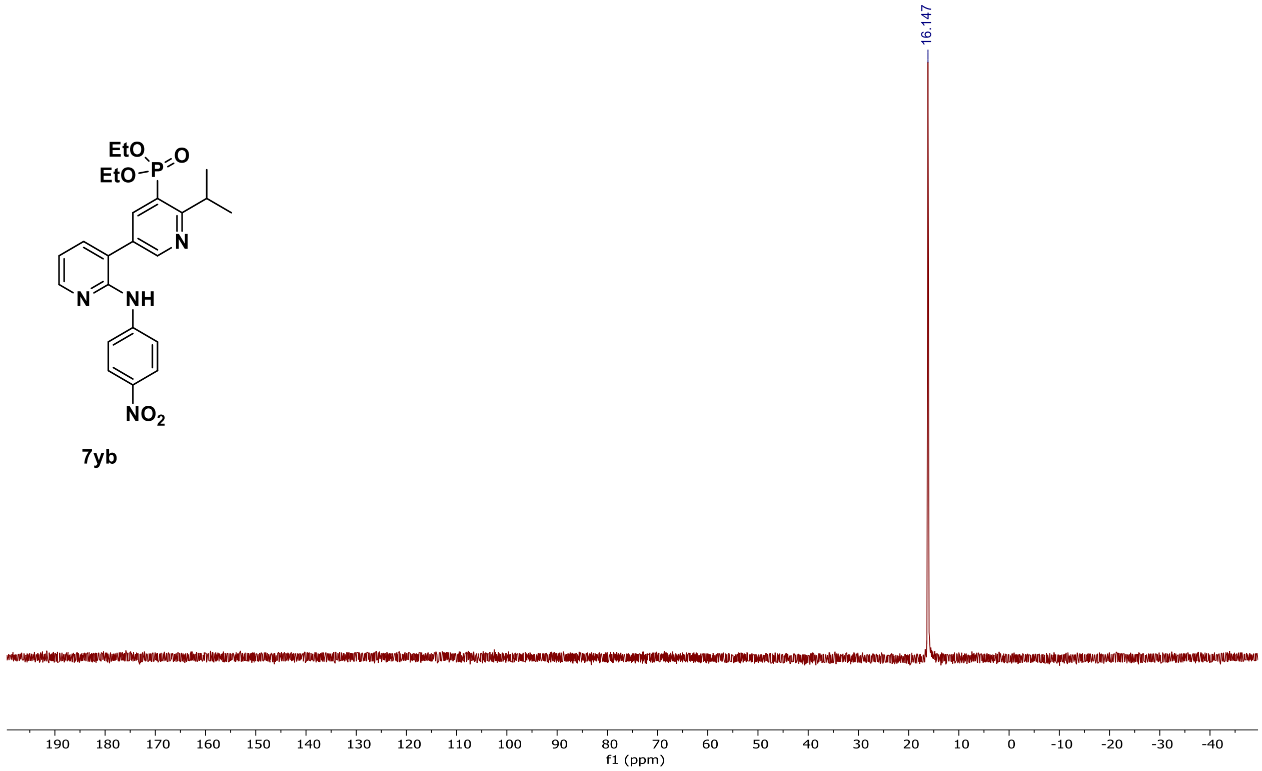

^{31}P NMR (162 MHz) Spectrum of **7yb** in $CDCl_3$

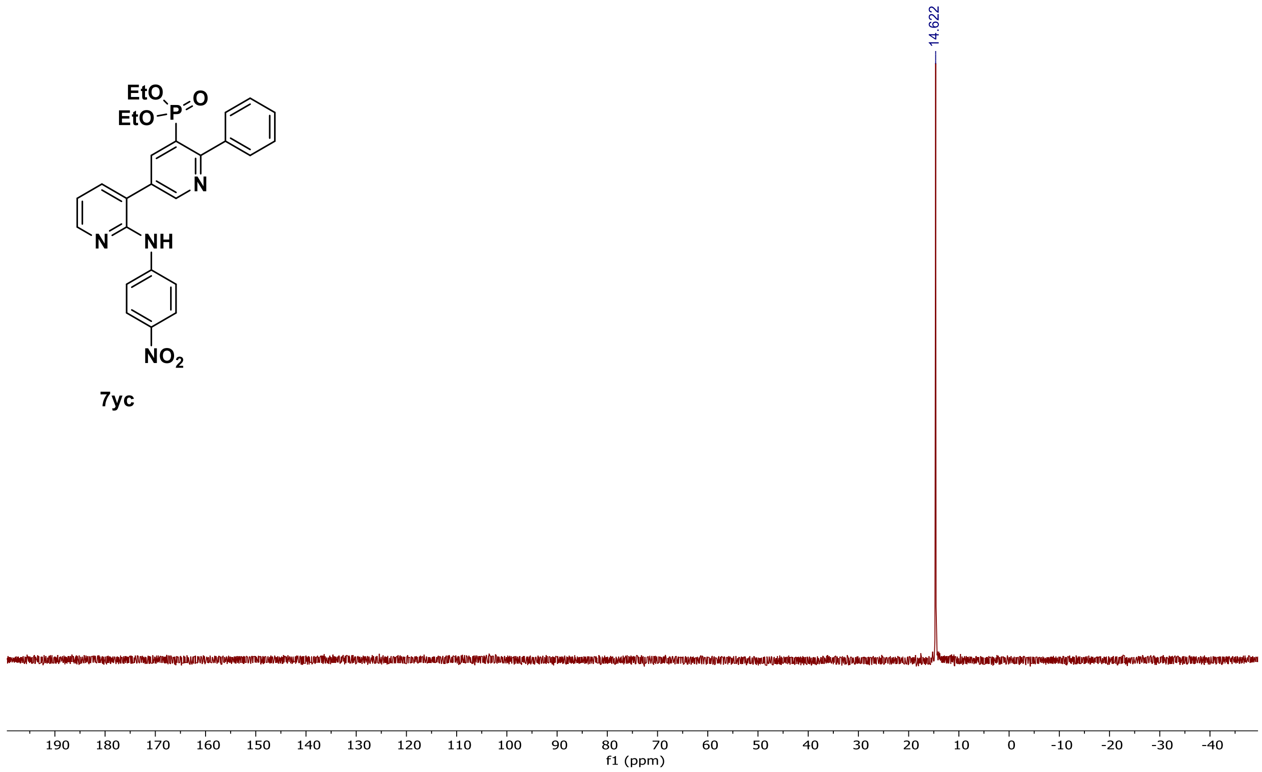

^{31}P NMR (162 MHz) Spectrum of **7yc** in $CDCl_3$

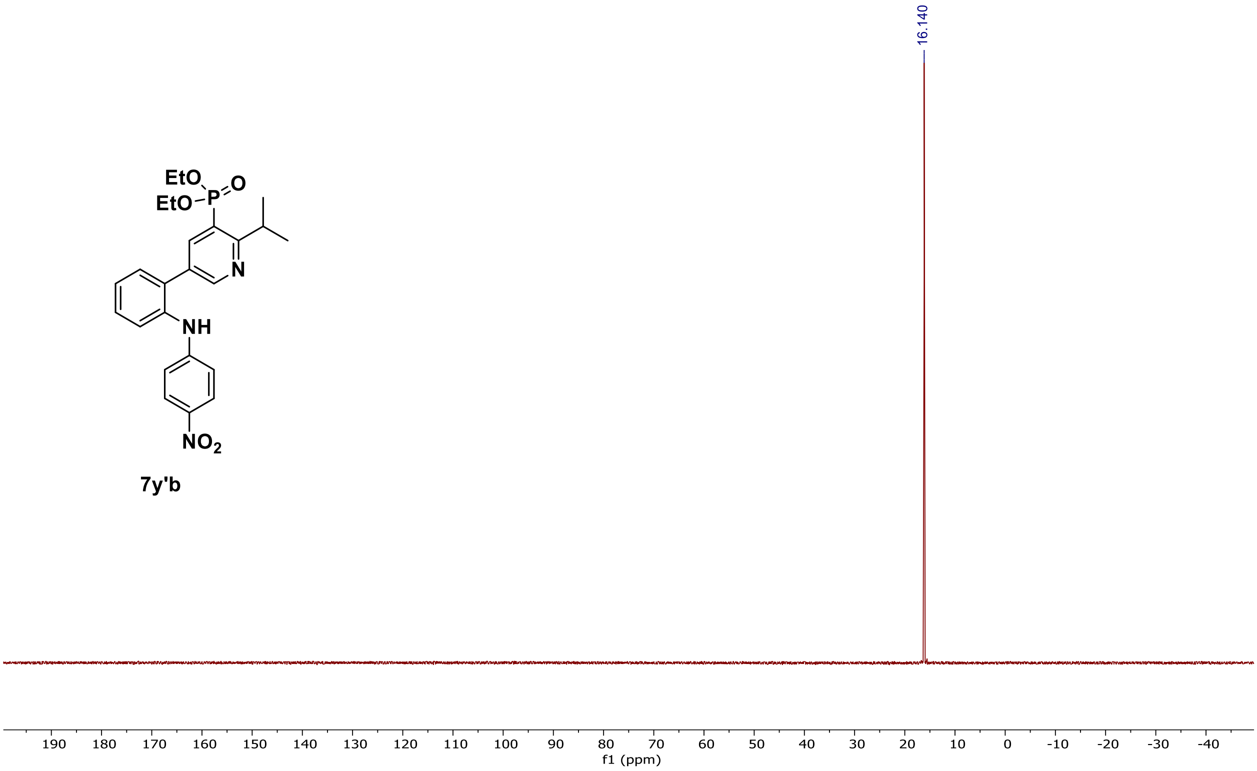

^{31}P NMR (162 MHz) Spectrum of **7y'b** in $CDCl_3$

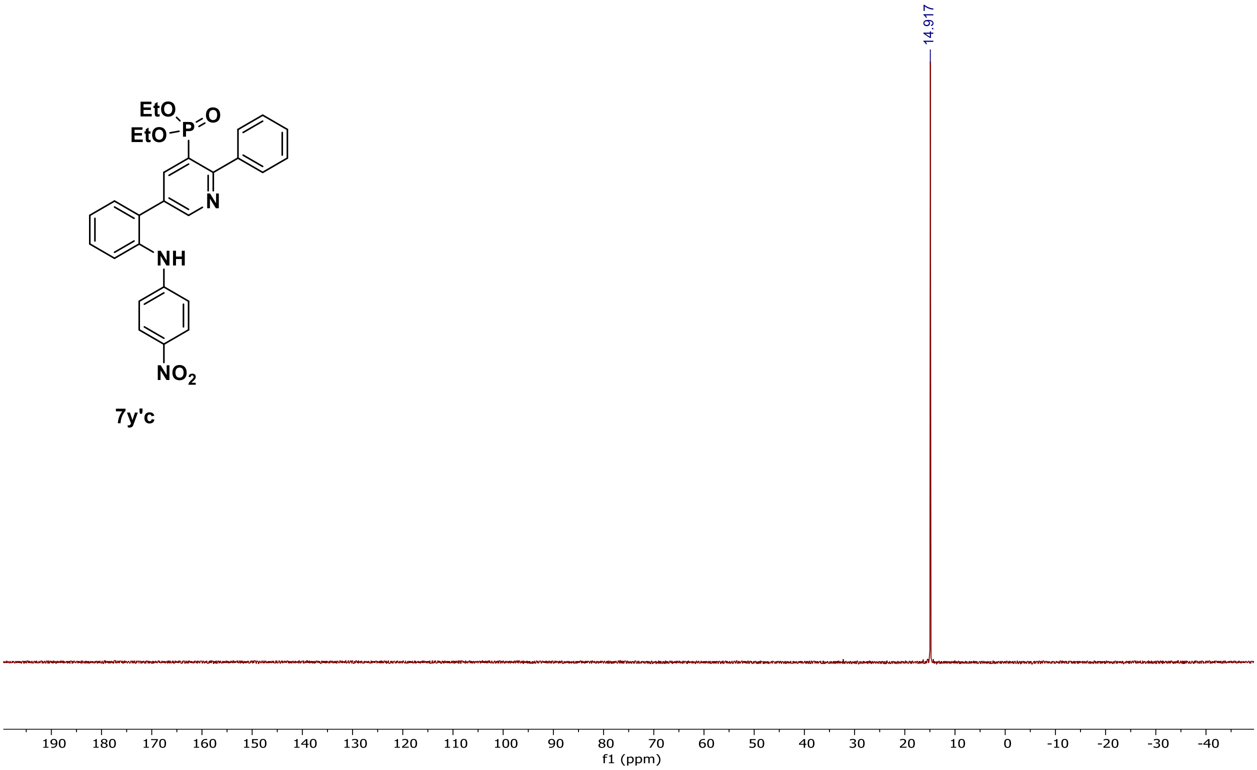

^{31}P NMR (162 MHz) Spectrum of **7y'c** in $CDCl_3$

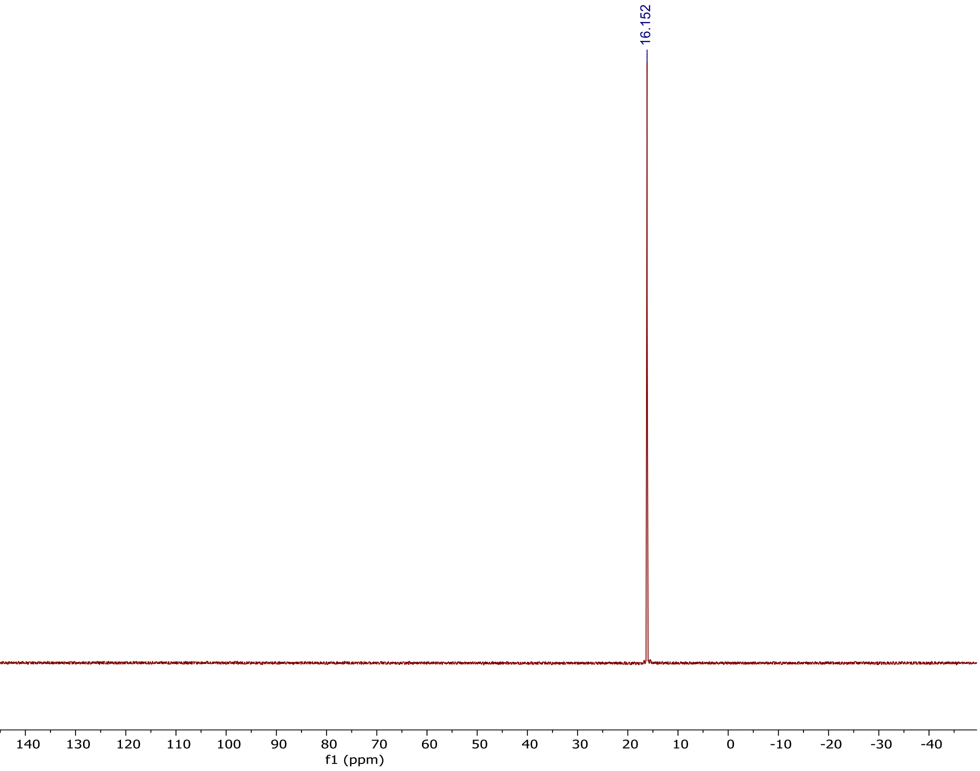

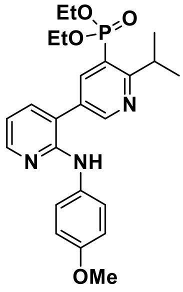

7zb

^{31}P NMR (162 MHz) Spectrum of **7zb** in $CDCl_3$

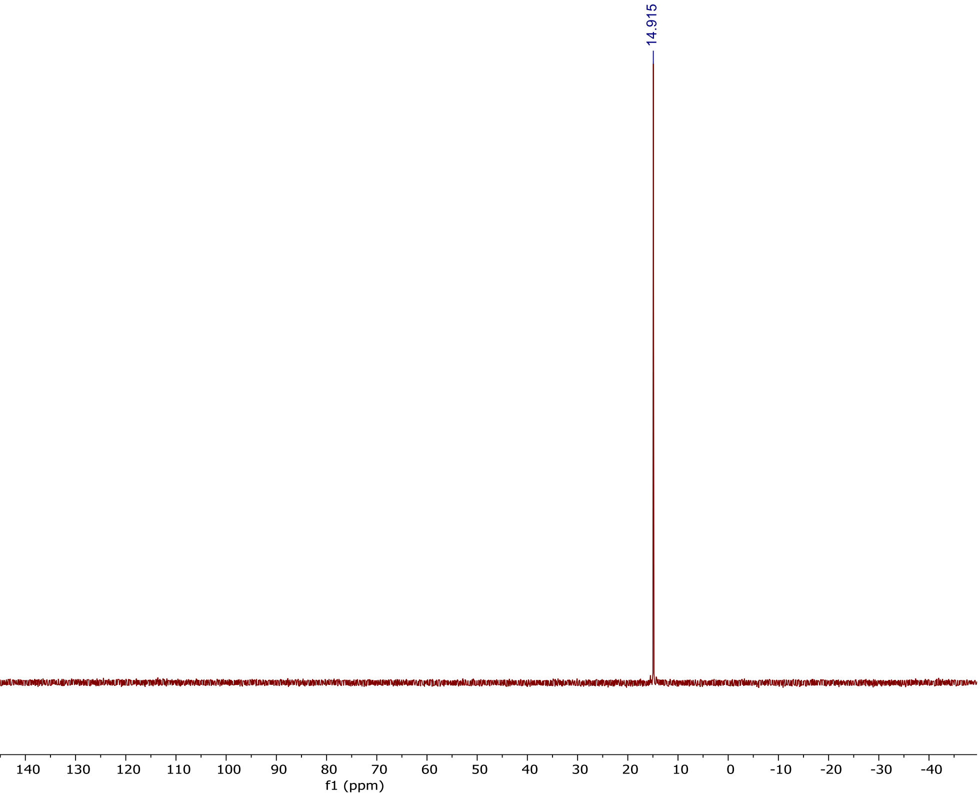

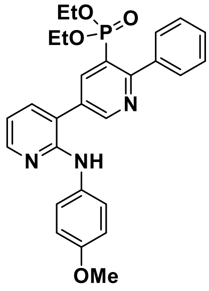

7zc

^{31}P NMR (162 MHz) Spectrum of **7zc** in $CDCl_3$

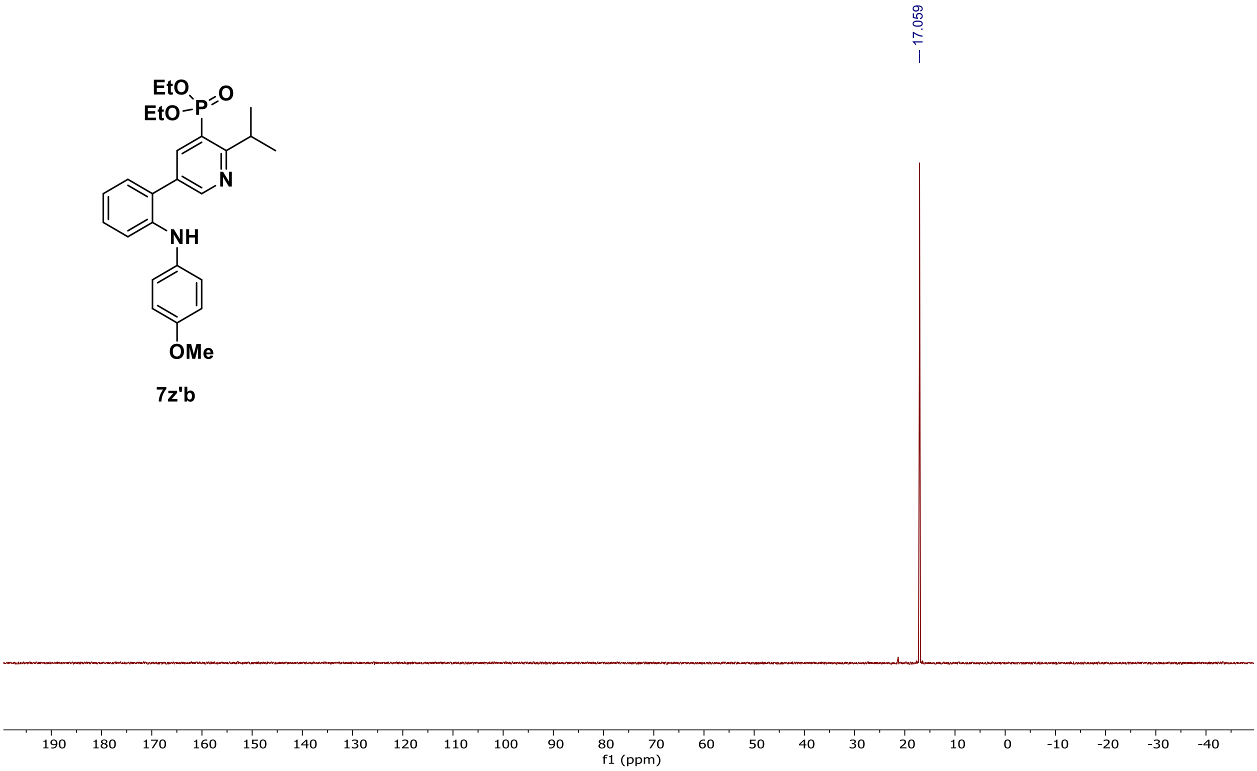

^{31}P NMR (162 MHz) Spectrum of **7z'b** in $CDCl_3$

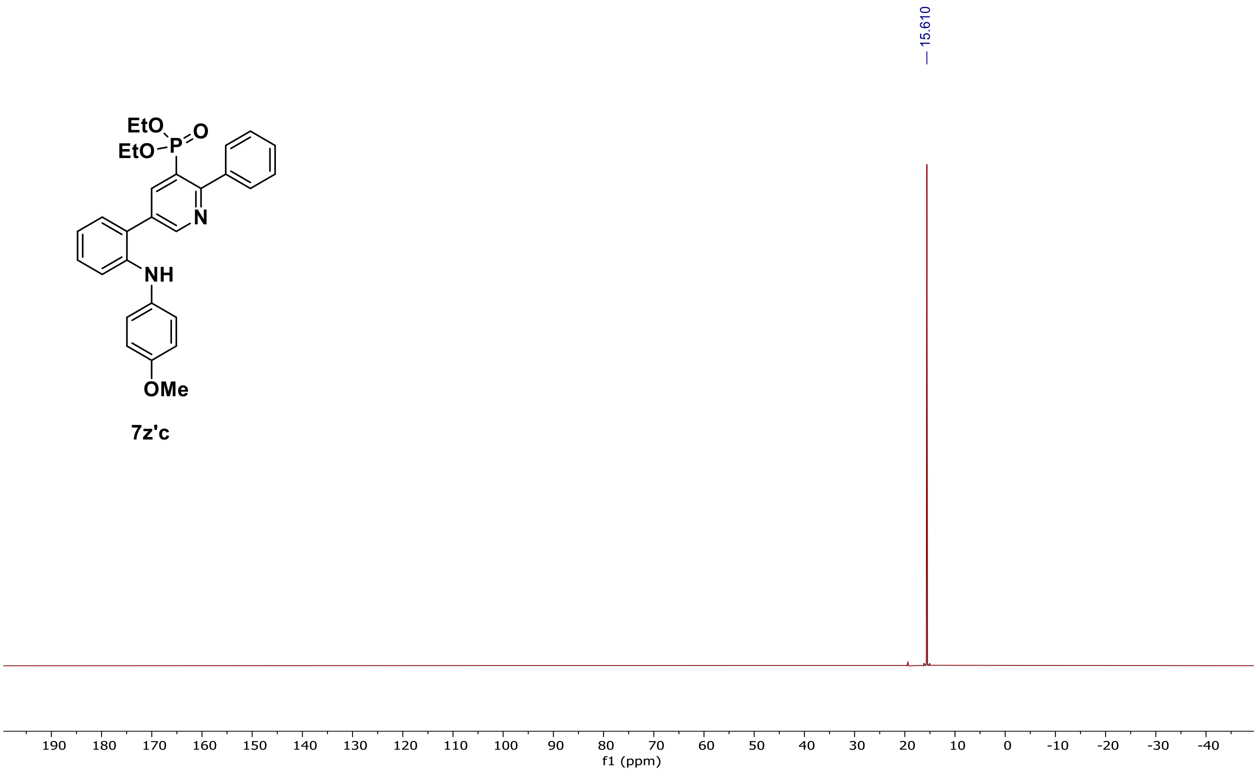

^{31}P NMR (162 MHz) Spectrum of **7z'c** in $CDCl_3$
